# Global responses to oxytetracycline treatment in tetracycline-resistant *Escherichia coli*

**DOI:** 10.1038/s41598-020-64995-1

**Published:** 2020-05-21

**Authors:** Thea S. B. Møller, Gang Liu, Hassan B. Hartman, Martin H. Rau, Sisse Mortensen, Kristian Thamsborg, Andreas E. Johansen, Morten O. A. Sommer, Luca Guardabassi, Mark G. Poolman, John E. Olsen

**Affiliations:** 1 University of Copenhagen, Department of Veterinary and Animal Sciences, 1870 Frederiksberg C, Denmark; 2 Oxford Brookes University, Department of Medical and Biological Sciences, Gipsy Lane, Headington, Oxford, OX3 OBP United Kingdom; 3 Technical University of Denmark, Department of Systems Biology, 2800 Lyngby, Denmark; 4 Technical University of Denmark, Novo Nordisk Foundation Center for Biosustainability, 2970 Hørsholm, Denmark; 5 Present Address: Public Health England, National Infections Service, Colindale, London, NW9 5EQ United Kingdom

**Keywords:** Microbiology, Antimicrobials

## Abstract

We characterized the global transcriptome of *Escherichia coli* MG1655:: *tetA* grown in the presence of ½ MIC (14 mg/L) of OTC, and for comparison WT MG1655 strain grown with 1//2 MIC of OTC (0.25 mg/L OTC). 1646 genes changed expression significantly (FDR > 0.05) in the resistant strain, the majority of which (1246) were also regulated in WT strain. Genes involved in purine synthesis and ribosome structure and function were top-enriched among up-regulated genes, and anaerobic respiration, nitrate metabolism and aromatic amino acid biosynthesis genes among down-regulated genes. Blocking of the purine-synthesis- did not affect resistance phenotypes (MIC and growth rate with OTC), while blocking of protein synthesis using low concentrations of chloramphenicol or gentamicin, lowered MIC towards OTC. Metabolic-modeling, using a novel model for MG1655 and continuous weighing factor that reflected the degree of up or down regulation of genes encoding a reaction, identified 102 metabolic reactions with significant change in flux in MG1655:: *tetA* when grown in the presence of OTC compared to growth without OTC. These pathways could not have been predicted by simply analyzing functions of the up and down regulated genes, and thus this work has provided a novel method for identification of reactions which are essential in the adaptation to growth in the presence of antimicrobials.

## Introduction

Tetracycline drugs are bacteriostatic antimicrobials which bind reversibly to the bacterial ribosome and interfere with protein translation^1^. The prevalence of resistance to this class has steadily increased in clinical isolates from both humans^2,3^ and animals^4^, roughly by 0.5% per year from 1950 to 2001 in isolates from humans^5^. While the use of tetracycline in human medicine has gradually decreased, the drug class remains among the most used in livestock production worldwide^6^.

The majority of the more than 40 genes encoding tetracycline resistance encode membrane-associated efflux proteins^7^, which selectively pumps tetracycline from the cytosol to the periplasm in exchange for a proton^8^. In *E. coli*, TetA and TetB are the most frequently observed tetracycline efflux pumps^4,9^. We have shown that a TetA-producing *E. coli* strain shows prolonged lag phase proportionally to the concentration of tetracycline in the growth medium, and that this concurs with increased expression of *tetA*^10^. This suggests that expression of the resistance mechanism or incorporation of the efflux pump into the cell-membrane constitute a burden to the bacterium. Similar observations of cellular adaptation in antimicrobial resistant bacteria grown in the presence of the drug to which they are resistant have been described for Extended Spectrum Beta-Lactamase producing (ESBL) *E. coli*^11,12^, methicillin resistant *Staphylococcus aureus*^12^ and colistin-resistant *Klebsiella pneumoniae*^13^, and interfering with these adaptive responses in certain cases re-sensitize the bacteria to the drugs^11,13^. It is therefore highly relevant to increase our understanding of the way that resistant bacteria adapt to growth in the presence of therapeutic concentrations of antimicrobials.

In the study of ESBL adaptation to cephalosporin treatment by Møller *et al*.^11^, a high number of genes encoding metabolic enzymes were shown to be significantly regulated. Understanding the importance of this for the metabolism is not straight forward, since the metabolic network contains a high number of redundant, alternative pathways^14,15^. Metabolic modeling has been a central tool for solving this problem^16^, but traditionally genome scale metabolic models (GSMs) only describe the network of enzyme-catalyzed and spontaneous reactions proceeding in the organism^17,18^, and not adaptation to changing environments^19^. Recently, however, several approaches to incorporate omic-data into modeling have been published (see for example^20^).

The aim of this study was to improve the understanding of the metabolic adaptations in an OTC-resistant, *tetA* encoding *E. coli* strain to treatment with OTC. We show that despite being highly resistant to oxytetracycline, adaptation to growth in the presence of the drug involves a high number of genes, and using GSM with incorporation of transcriptome data, we identify metabolic adaptations that could not have been identified simply from looking at the list of significantly regulated genes.

## Results

### Global gene responses in *E. coli* MG1655::*tetA* growing in the presence of therapeutic concentrations of OTC

In order to study the adaptation to OTC treatment, we selected a variant of *E. coli* MG1655, where *tetA*, together with its natural regulator, *tetR*, had been cloned into the chromosome. RNA-sequencing was used to identify significantly up and down regulated genes, when the strain was grown in MH-2-broth with 14 mg/L OTC in comparison to growth without OTC. The chosen OTC concentration corresponds to ½ MIC of the strain to OTC, and is above the peak serum concentration that is obtained in humans following treatment^21^.

Significant change in expression, based on a false discovery rate (FDR) < 0.05, was demonstrated for 1646 genes, showing that either the presence of the drug in the cell cytosol or the production and incorporation of an increasing number of TetA-pumps caused dramatic changes in cell homeostasis. Of the significantly regulated genes, 946 genes were up-regulated and 700 were down-regulated (Supplementary Material Fig. S1, and Tables S1 and S2). When cut-offs of Abs(Log_2_FC) > 2.0 alternatively Abs(Log_2_FC) > 1.5 were used, 149 and 333 genes were up-regulated and 119 and 211 genes were down regulated. The changes were generally moderate in up-regulated genes with a maximum of Log_2_FC = 3.94 (14.58 fold). The most down regulated genes showed up to Log_2_FC = −7.46 (73.78 fold) change in expression (Table 1).

**Table 1:** List of the 20 most up regulated and down regulated genes in *E. coli* MG1655::*tetA* growing in MH-2- broth with 14 mg/L oxytetracycline (OTC) compared to growth in MH-2-broth without OTC.

Genes – Up	Log FC- change^#^	Function/enzyme	Genes down	Log FC change^#^	Function/enzyme
*ydcI*	3.94	DNA-binding transcriptional regulator	*trpE*	−7.49	Anthranilate synthase component I
*prpB*	3.80	2-methyl-isocitratelyase	*trpD*	−7.17	Anthranilate synthase component II
*fadB*	3.74	Fatty acid oxidation complex	*mtr*	−6.43	Tryptophan/indole:H + symport permease
*ycdO*	3.70	Conserved protein	*tdcB*	−5.93	Threonine dehydratase
*cyoA*	3.70	Cytochrome O oxidase subunit II	*trpC*	−5.79	Part of tryptophan synthesis pathway
*bfd*	3.67	Bacterioferritin-associated ferredoxin	*dcuC*	−5.72	Dicarboxylate transporter
*ndk*	3.61	Nucleoside diphosphate kinase	*nrfC*	−5.62	Component of nitrite reductase complex
*puuA*	3.54	γ-glutamyl-putrescine synthetase	*nrfB*	−5.36	Component of nitrite reductase complex
*sdhC*	3.53	Component of succinate dehydrogenase	*nrfD*	−5.29	Component of nitrite reductase complex
*mdtQ*	3.43	Predicted outer membrane protein	*trpB*	−4.96	Tryptophan synthase, β subunit
*lldP*	3.40	L-lactate permease	*trpA*	−4.76	Tryptophan synthase, α subunit
*sdaB*	3.37	L-Serine deaminase II	*gudX*	−4.74	Predicted glucarate dehydratase
*fhuF*	3.32	Siderophore-iron reductase	*cadB*	−4.71	Lysine-cadaverine antiporter
*tnaB*	3.31	Tryptophan:H + symport permease	*nrfA*	−4.70	Component of nitrite reductase complex
*efeU*	3.29	Ferrous iron permease (annotated as pseudogene)	*tdcC*	−4.42	Serine/threonine:H + symport permease
*ddpX*	3.25	D-Ala-D-Ala dipeptidase	*garD*	−4.42	D-galactarate dehydratase
*astC*	3.24	Succinylornithine transaminase	*narH*	−4.41	Nitrate reductase A, β - subunit
*bioB*	3.23	Biotin synthase	*apaG*	−4.26	Hypothetical protein
*purK*	3.20	Part of purine synthesis	*garL*	−4.21	2-dehydro-3-deoxygalactarate aldolase
*sdhD*	3.19	Succinate dehydrogenase membrane protein	*hypB*	−4.05	Accessory protein for nickel incorporation into hydrogenase isoenzymes

**Figure 1:**
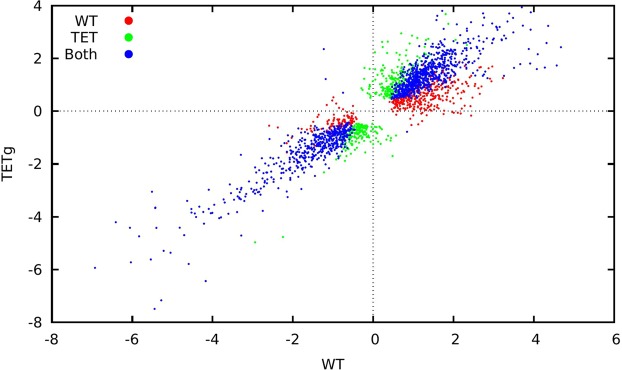
Comparison of responses by WT *E. coli* MG1655 (x-axis) and *E. coli* MG1655::*tetA* (y-axis) strains to ½ MIC OTC challenge, in which FDR was less than 0.05 in at least one strain. WT *E. coli* MG1655 was grown with and without OTC at 0.25 mg/L and *E. coli* MG1655::*tetA* was grown with and without OTC at 14 mg/L. Red points indicate genes whose change was significant only in the *E. coli* MG1655, green points genes that were significant only in *E. coli* MG1655::*tetA* and blue points genes that changed significantly in both strains.

In order to determine whether the high number of significantly regulated genes was a direct consequence of expressing the resistance mechanism or a common, general response to ½ MIC treatment with OTC, we also characterized the response in the WT MG1655 strain to growth with ½ MIC OTC (0.25 mg/L OTC). A total of 400 genes were only regulated in the resistant strain, suggesting that this was the number of genes associated with the TetA expression (see Fig. 1). On the other hand, this suggested that the majority of regulated genes were part of a common (presumably stress-related) response, rather than a consequence of expression of the resistance mechanism.

*ddpX* was among the up-regulated genes (Table 1). This gene encodes a D-alanyl-d-alanine dipeptidase and is involved in peptidoglycan synthesis. It belongs to the RpoS regulon^22^, which is the master stress-regulon in *E. coli*. Using the list of Sigma^S^ controlled genes published by Weber *et al*.^23^, we searched our data for signs of RpoS induced stress response. *rpoS* itself was not significantly regulated, and among the 16 genes listed as the first-line adaptation to stress in this regulon, only *fadB* was significantly up-regulated. Since TetA is a membrane bound pump, we specifically further looked for signs of membrane stress by analyzing regulation of the Cpx, RpoE and Psp-systems^24–26^ using the cut-off value Abs(Log_2_FC) > 1.5. None of the genes reported to belong to these systems were significantly regulated. *pspE* of the phage shock proteins was boarder line down-regulated (log_2_FC −1.44), however, this gene is believed to encode a thiosulphate sulfotransferase, and even though it is named phage shock protein E, is not considered a stress associated protein^27^. Together, the patterns of gene regulation did not indicate severe membrane stress due to incorporation of TetA pumps.

Analysis for enrichment of biological functions, molecular processes, cellular components and KEGG pathways was performed using genes that were up or down regulated more than Abs(Log_2_FC) > 2.0 to focus on the most highly regulated genes. MG1655::*tetA* was found to up-regulate biosynthesis processes related to ribonucleoside and ribonucleotide production, in particular purine biosynthesis, and molecular processes and cellular components related to ribosome function (Supplementary Table S3). Anaerobic respiration, nitrogen metabolism and phenylalanine, tyrosine and tryptophan biosynthesis were in particular enriched among the down-regulated genes (Supplementary Table S4). The down regulation of tryptophan biosynthesis was counter-acted by tryptophan uptake systems being highly upregulated (*tnaB* see Table 1).

**Figure 2:**
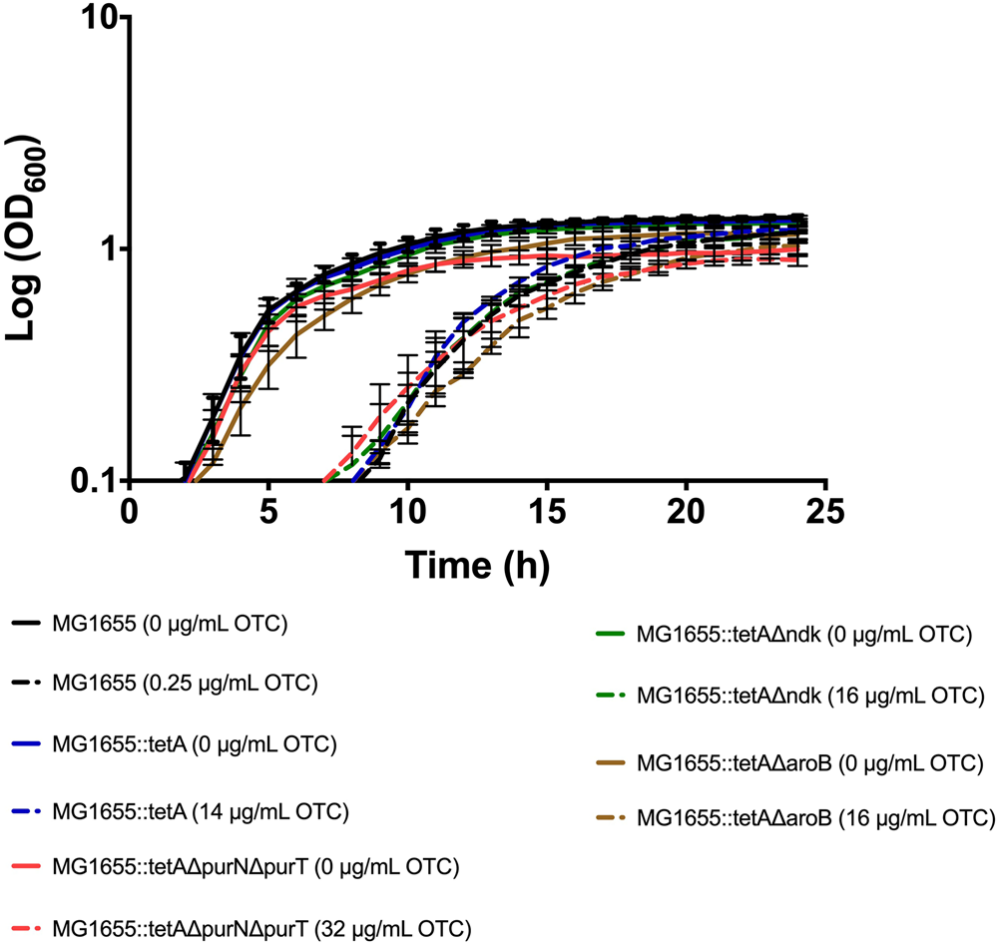
Growth curves of *E. coli* MG1655::*tetA* with *purT/purN*, *ndk* and *aroB* knock outs. MG1655::*tetA* and its mutants were grown in MH-2 media without OTC and with ½ MIC of OTC for each strain on a BioScreen CTM. Three independent replicates were performed of the growth data; the data shown represent the means.

In total, 14 purine synthesis genes (*purF, purD, purT, purN, purL, purM, purC, purE, purK, purB, purH, apt, ndk* and *prs*) were up-regulated between Log_2_FC 3.62 and Log_2_FC 1.59. To analyze the significance of this up-regulation, single and double mutants of *purN* and *purT* were constructed. These genes encode the two redundant, phosphoribosylglycinamide transformylases acting at step three in the purine biosynthesis^28^. Blockage of this step results in accumulation of glycinamide ribonucleotide (GAR) and depletion of all down-stream products, and is therefore considered essential for the purine synthesis^28^. MIC of OTC was 28–32 mg/L for MG1655::*tetA*, MG1655::*tetA*∆*purN* and MG1655::*tetA*∆*purT*, while it increased to 64 mg/L for MG1655::*tetA*∆*purN*∆*purT* (Table 2). The wild type and mutated strains grew equally well in MH-2 medium without OTC (results for MG1655::*tetA*∆*purN*∆*purT* and WT strain are shown in Fig. 2). WT and mutant strains increased significantly in lag phase when grown in the presence of ½ MIC OTC, however, to the same degree, and they did not differ significantly in growth rate, suggesting that even though the purine biosynthesis genes were highly upregulated, the pathway was not essential for growth in the presence of OTC (Fig. 2). A possible explanation for this observation is redundancy with the purine salvage pathways, and indeed, major genes involved in this pathway (*apt* and *hpt*) were significantly regulated by treatment with ½ MIC of OTC in MG1655::*tetA* as well as in WT MG1655 (Supplementary Fig. S2). In minimal media (M9), the wild type and single mutant strains showed even longer lag phases and decreased growth rate compared to growth in MH-2. As expected, the ∆*purN*∆*purT* mutant did not grow in M9 media, since no purines were available in this medium (data not shown).

**Table 2:** MIC values (mg/L) to oxytetracycline of strains investigated in the study.

Strain	Extra antimicrobials added^a^ (mg/L)	MIC
MG1655	None	0.50
MG1655	Chloramphenicol 2.0	0.50
MG1655	Chloramphenicol 1.0	0.50
MG1655	Chloramphenicol 0.5	0.50
MG1655	Gentamycin 1.0	0.50
MG1655::*tetA*	None	28
MG1655::*tetA*	Chloramphenicol 2.0	8
MG1655::*tetA*	Chloramphenicol 1.0	16
MG1655::*tetA*	Chloramphenicol 0.5	16
MG1655::*tetA*	Gentamycin 1.0	0.25
MG1655::*tetA*	Gentamycin 0.5	8
MG1655::*tetA*Δ*ndk*	None	32
MG1655::*tetA*Δ*purN*	None	32
MG1655::*tetA*Δ*purT*	None	32
MG1655::*tetA*Δ*purN*Δ*purT*	None	64
MG1655::*tetA*Δ*aroC*	None	32
MG1655*::tetAΔaroB*	None	32
MG1655:: *tetAΔndk*	None	32

^a^Additional antimicrobials to OTC added to the growth medium during the MIC testing.

*ndk* also belongs to the purine biosynthesis system. This enzyme, which was on the list of the most highly upregulated genes, encodes a nucleoside diphosphate kinase which is part of the UTP and CTP biosynthesis pathway and which also functions in pyrimidine salvage^29^. We also knocked out this gene and compared the growth phenotype and MIC to OTC to that of the MG1655::*tetA*. The two strains grew equally well with and without OTC (Fig. 2). In accordance with this, MIC of the *ndk* mutant (32 mg/L) was not significantly changed compared to MIC of the WT (28 mg/L) (Table 2).

Fifty-one ribosomal and translational genes were significantly up-regulated with Log_2_FC between 2.72 and 1.53 in the presence of 14 mg/L of OTC. This included genes encoding 18 50S ribosomal subunit proteins, 14 30S ribosomal subunit proteins and 19 tRNA synthetase genes. To investigate the importance of up-regulation of the protein synthesis, we added sub-MIC concentrations of chloramphenicol or gentamicin simultaneously with OTC. These drugs inhibit the protein synthesis by binding to the 50S and 30S ribosomal subunit, respectively^30,31^. The MIC of OTC decreased from 32 mg/L to 8 mg/L when 2 mg/L chloramphenicol was present and to 0.25 mg/L when 1 mg/L gentamicin was present (Table 2). In a checker-board assay, synergy, defined as FIC ≤ 0.5^32^ was found between 8 mg/L OTC and 0.5 mg/L gentamicin, and 0.25 mg/L OTC and 1 mg/L gentamicin (Supplementary Fig. S3). The MIC of the MG1655::*tetA* strain for chloramphenicol was 4 mg/L and for gentamicin 2 mg/L. The MICs for the WT MG1655 strain to chloramphenicol and for gentamicin were as listed for the MG1655::*tetA* strain. Combination treatment with OTC and these two drugs did not decrease MIC in the wild type MG1655 MIC to OTC (data not shown).

Genes involved in aromatic amino acid biosynthesis, as mentioned above, were highly down-regulated. In common, these amino acids are synthesized with chorismate as substrate. Chorismate is the end product of the Shikimate pathway. In order to analyse the importance of down-regulation of the amino acid biosynthesis genes, we first tried to knock-out *tyrA* and *pheA*, which both catalyzes the first step in the formation of the amino acids from chorismate. Though several optimizations were tested, it was impossible to construct this mutant, and we concluded that *tyrA* and/or *pheA* are essential for growth in LB media which we used for construction of the mutants. It should be noted, however, that others have succeeded in constructing combined *tyrA/phe* mutants in *E. coli*^33^. Instead, we knocked out *aroB* and *aroC*, which are the last enzyme in the Shimikate pathway before it branches to the aromatic amino acid biosynthesis pathways. As shown in Fig. 2 (data for MG1655::*tetAΔaroB* only) the mutant grew at comparable growth rate as the wild type strain both with and without ½ MIC of OTC. MIC towards OTC did not differ significantly between MG1655::*tetA* and the two mutants (Table 2). As expected, the *aroB* and *aroC* mutants did not grow in M9 minimal medium due to lack of aromatic amino acids (data not shown).

### Analysis of the secondary transcriptome to OTC by metabolic modeling

In total 468 genes encoding metabolic enzymes were significantly up-regulated in MG1655::*tetA* (49.5% of up regulated genes), and 319 genes were down regulated (44.3% of down regulated genes). This suggested that changes in the metabolism were a major component in the adaptation to growth in the presence of high concentrations of OTC. In order to investigate the metabolic significance of this we used a modeling approach. We built a genome scale model of *E. coli* K-12 MG1655 from the ecocyc database. It had a total of 1335 reactions encoded by 880 unique genes, 1335 internal and 64 external metabolites. The model was curated to be fully balanced to all atomic species, including protons and was demonstrated to have no violations of conservation of energy or mass. The model was able to produce all biomass components of growing *E. coli* individually and in combination in proportions corresponding to published experimental data^34^ (data not shown). Generating this biomass composition, using FBA to minimize total flux in the network and assuming growth on M9 media, the model utilized 331 of the 1351 reactions in the model.

We used the change in the expression of a metabolic gene to calculate a weighting factor for the reaction(s) associated with that gene, and when these weighting factors were applied, Flux Balance Analysis showed that 59 reaction fluxes increased significantly and 43 decreased. Inspection of this set of reactions showed a 50% increase in the flux of the proton-translocating ATP synthase reaction and concomitant increases in reactions of the electron transport chain (ETC), Oxygen and glucose uptake systems and CO_2_ excretion systems, all pointing to a substantial increased energy demand resulting from OTC exposure. Other regulated reactions were primarily associated with central carbon metabolism in addition to reactions involved with amino acid and nucleotide metabolism. These reactions were then extracted to form a regulated sub-model for further analysis.

The sub-model formed from the 102 up and down regulated reactions contained a total of 50 metabolites that were internal to the sub-model, and 75 metabolites that were exchanged with the rest of the model (i.e. those reactions whose computed fluxes did not vary in response to the weighting associated with OTC exposure). It proved infeasible to calculate elementary modes of the sub model, presumably because of the large number of exchange reactions. However, as described in the methods section, it was possible to further reduce this sub-model to a network of 33 reactions and 50 metabolites of which 20 were exchanged with the whole model. This reduced sub-model had a total 94 elementary modes, and using the method described by Schuster *et al*.^35^, the flux distributions in both the weighted and un-weighted sets could be accounted for by 8 of these elementary modes as shown in Table 3.

**Table 3:** Simplified stoichiometries of elementary modes and their associated fluxes in the reduced sub-model with and without weighting factors for MG1655::*tetA* grown in the presence of 14 mg/L OTC compared to growth without OTC.

Flux		Substrates	Products
0 mg/L OTC	14 mg/L OTC		
5.76e-6 (68)	1.12e-4	Glc, O2	AcCoA, NADPH, ATP
1.05e-5 (31)	2.28e-4	AKG NH4 Glc O2	Pyr, Glt, NADPH
1.38e-5 (67)	1.84e-5	AKG, NH4, Glc	Glt, AcCoA, NADPH, ATP
1.44e-5 (17)	1.84e-5	AKG, NH4, Glc	Glt, AcCoA, NADPH
3.53e-5 (52)	2.44e-4	Glc, O2	Pyr, ATP
5.64e-5 (81)	8.67e-5	AKG,NH4,Glc,O2	Glt, AcCoA. NADPH, ATP
6.00e-5 (79)	1.85e-4	Glc,O2	AcCoA, NADPH, ATP
6.57e-5 (82)	3.44e-5	Glc,O2	AcCoA, NADPH, ATP
5.76e-6 (68)	1.12e-4	Glc, O2	AcCoA, NADPH, ATP
1.05e-5 (31)	2.28e-4	AKG NH4 Glc O2	Pyr, Glt, NADPH
1.38e-5 (67)	1.84e-5	AKG, NH4, Glc	Glt, AcCoA, NADPH, ATP

Glc:glucose, AcCoA:acetyl-coenzyme A, AKG: alpha ketoclutarate, Glt:glutamate, Pyr:pyruvate

**Figure 3:**
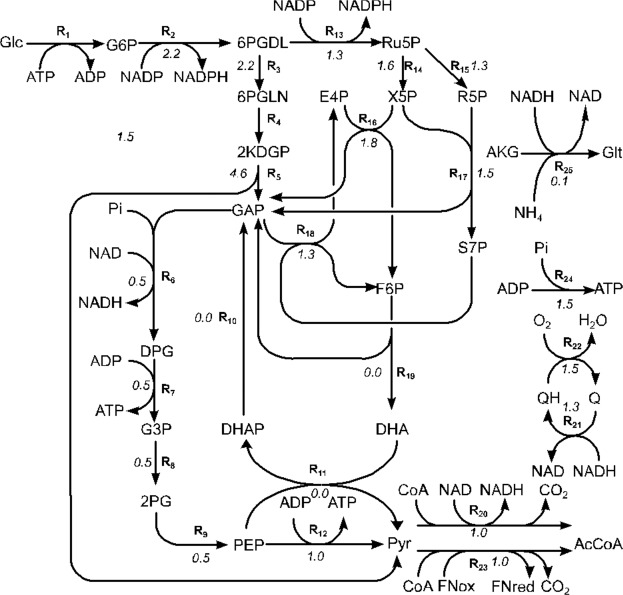
Reactions of the reduced sub-model. R1–R9, R12: E–D pathway, R13–R18: OPPP, R20–R24: OP. Italicised numbers are the ratio of weighted/reference solution values.

The reactions used by the elementary modes comprised mainly of the Entner-Doudoroff (E-D) pathway, the oxidative pentose phosphate pathway (OPPP) and oxidative phosphorylation (OP). In addition to these reactions, the network also utilized fructose-6-phosphate aldolase, PEP-glycerone phosphotransferase and glutamate synthase (R19, R11 and R25 in Fig. 3 respectively). Thus, the gene regulation pattern in response to OTC treatment caused the metabolism to shift flux away from the E-D pathway, and in to the OPPP and OP. Reactions 11 and 19 (Fig. 3) only carried fluxes in the strain reference, and not in the modeling with the weighted, solution.

## Discussion

Global expression analysis was performed to characterize adaptation to growth in the presence of OTC in an OTC resistant *E. coli* MG1655 strain. The strain was grown in the presence of therapeutically relevant concentrations of OTC and the transcriptome was used to guide metabolic modeling. Transcriptomic analysis has previously been used to characterize adaptive changes in cephalosporin resistant *E. coli* and methicillin resistant *S. aureus*^11,12^, while others have used a transposon guided sequencing (TraDis) approach to characterize adaptive changes in pan-resistant *K. pneumoniae*^13^. Together, the data has shown that even for resistant bacteria, fighting antibiotics is not just a matter of expressing the resistance mechanisms, but involves regulation of genes that are not part of the resistance mechanism, *per se*. The work by Brochmann *et al*.^12^ also showed that there are strains differences in this response, and the results of the current study should be viewed with some caution, as only one strain was analyzed.

OTC treatment of the resistant strain caused major changes in gene regulation, and genes belonging to 44 different biological functions were found to be significantly regulated. The majority of the significantly regulated genes were also significantly regulated when WT MG1655 was treated with ½ MIC of OTC, showing that the main part of the response was not directly related to expression of the TetA pump. In parallel to our study, another transcriptome study of a tetracycline resistant *E. coli*, belonging to the Extra Intestinal Pathogenic *E. coli* group APEC and grown in the presence of 32 mg/L tetracycline, was published^36^. Our study confirms the observation from that study of a general up regulation of ribosome associated genes and the purine metabolism and the down regulation of anaerobic respiration and nitrate reduction, when grown with tetracycline. Biosynthesis of aromatic amino acids was not down regulated in that study, confirming strain specific differences.

TetA strains grown in the presence of high concentrations of OTC show a significant increase in lag phase, and this has been proposed to indicate stress due to incorporation of TetA pumps in the membrane^10^. In the current study, we did not observe indications of membrane stress. It has proved technically impossible to determine the number of TetA pumps in *E. coli* by proteomics^10^, and thus it is difficult to estimate whether there is a fixed upper number of TetA pumps that can be incorporated into the membrane, thus avoiding membrane stress.

We tested whether significantly regulated pathways were essential for expression of the resistant phenotype. The purine synthesis was highly up-regulated indicating that purine *de novo* synthesis is an important part of the adaptive response to OTC treatment. Similar up-regulation of purine synthesis was observed in ESBL *E. coli* in the presence of cefotaxime^11^, and was also shown in the recent expression study with the APEC strain^36^. Despite this, an increase in MIC in the presence of OTC was observed when purine synthesis was blocked. Purines can be obtained in other ways, either by uptake from the medium or by purine salvage pathways^37,38^. In the current study, important genes in the purine salvage pathway were up-regulated in the TetA strains as well as in the WT MG1655 in the presence of ½ MIC of OTC, and it is likely that this system can fully compensate for lack of purine biosynthesis under these conditions. In minimal medium, lack of the purine synthesis pathway was costlier to the strain judged from growth responses, and it may be that the effect of blocking purine biosynthesis *in vivo* (in the patient) is more pronounced than the growth study in MH-2 media suggests.

Protein synthesis was shown to be essential for the response of MG1655::*tetA* towards OTC. The MIC towards tetracycline decreased below the epidemiology cut-off for OTC (≤8 mg/L^39^) when low concentrations of the ribosome inhibitors chloramphenicol or gentamicin were present. MIC in the WT MG1655 strain did not decrease in the same way. This suggests that the mechanism was related to operating the efflux pump in the MG1655::*tetA* strain, putting high metabolic demands (presumably energy) on the resistant strain, and that this is then overloaded by the challenge from the second antibiotic. However, an additive effect of OTC and chloramphenicol on *E. coli* has previously been observed by Ciak and Hahn^40^, and confirmed by Garrett and Brown^41^ based on studies of antibiotic sensitive *E. coli* strains, and it may also be that the synergistic effect is too small to create a significant effect on MIC at the low OTC concentrations tolerated by the WT strain. To our knowledge, the promising effect of combining OTC and chloramphenicol with the goal of reducing OTC resistance in a highly resistant strain has not been reported previously. The combination of tetracycline and chloramphenicol has previously been used to treat against *Streptococcus viridans* infections, however, it only resulted in a partial treatment effect^42^. Furthermore, the two antibiotics showed no synergy when tested on several strains of *Staphylococcus aureus*^42^. Gentamicin, like OTC, targets the 30S ribosomal subunit, but with a different mode of action compared to OTC^3,43^. This could be the reason for the observed synergy. Combining gentamicin and doxycycline has been used as an effective combination for treatment of human brucellosis^44^.

Biosynthesis genes for aromatic amino acids were significantly down-regulated. To investigate the importance of this, and the Shikimate pathway in general, we eliminated two essential enzymes in the Shikimate pathway. *aroB* encodes the 3-dehydroquinate synthase, which is the second step in the synthesis of chorismate, while *aroC* encodes the chorismate synthase, which is the last step in the Shikimate pathway. After this step, chorismate is used in the biosynthesis of the aromatic amino acids^45,46^. All the enzymes of the Shikimate pathway are attractive targets for the development of anti-metabolites and antimicrobials, as the pathway is not present in mammals^47,48^. 5-enolpyruvylshikimate-3-phosphate (EPSP) synthase, encoded by *aroA*, has been studied extensively and is the target by the active ingredient of the commercially available broad-spectrum herbicide RoundUp™ ^49^. In the current study, no effect on growth with OTC was seen from knocking out genes of the Shikimate pathway, which is in accordance with the observation that biosynthesis was downregulated. For tryptophan, we saw indication that the down regulation of biosynthesis was counter-acted by tryptophan uptake systems being highly upregulated.

The largest group of regulated genes was metabolic enzymes. In order to investigate the metabolic network on a global scale, we used a novel approach where we incorporated the transcriptome date into global metabolic modeling. We first build an *E. coli* MG1655 genome scale metabolic model based on information available in the publicly available and standardized biochemical database, BioCyc. There is already an existing *E. coli* model, EcoCyc–18.0–GEM^50^, constructed from the EcoCyc database. There are important differences between these two models; EcoCyc–18.0–GEM is a larger model with 2286 reactions and 1453 metabolites. Most of these extra reactions were removed from the model presented here, owing to more stringent criteria for reaction inclusion in terms of metabolic relevance and atomic balance. Furthermore, a small number of reactions in our model had no equivalent in EcoCyc–18.0–GEM. These reactions were manually defined in the module of extra reactions (see methods section). The EcoCyc–18.0–GEM model has a greater number of un-conserved metabolites (1170), than our model. This includes compounds consisting of carbon, nitrogen, phosphate and sulfur. The model presented here had no un-conserved metabolites.

Methods for the integration of transcriptomic data with genome scale metabolic models have been described previously. The aim with all the integration methods is to obtain improved flux predictions using transcriptomic data, and they are based upon the assumption that the expression rate of a given gene, obtained from the experimental data, is correlated with the flux through the reaction catalysed by the enzyme encoded by the gene. This assumption is not necessarily correct as illustrated by Yang *et al*.^51^. Different approaches have been used. Covert *et al*. used Boolean logic to evaluate whether sufficient expression had occurred for a given reaction to proceed in order to further constrain the FBA problem^52^. This may not be fully accurate, as many regulatory mechanisms cannot be described with Boolean logic^19^. Other methods, e.g. GIMME^53^ and iMAT^54,55^, are based on systematic removal of reactions from the model based on low transcriptomic levels. A more realistic approach was recently introduced be Yang *et al*.^20^, who simulated growth with incorporation of data on protein abundances. The advantage of the weighting system used in the current study was that it favours reactions that are associated with high levels of gene expression and select against those with low expression levels, and thus the method (in contrast to other approaches^53–55^) does not depend on setting arbitrary threshold values of expression that lead to the inclusion or exclusion of a given reaction. One problem with the integration of transcriptomic data into metabolic models consists of multi-gene and multi-reactions situations^19^ and that there is not necessarily a straight correlation between gene and protein expression.

The modelling analysis resulted in 102 reactions whose flux changed in response to the added weighting. This sub-network was reduced to a more tractable size of 25 reactions. The calculated flux changes are consistent with an increased demand for ATP when *E. coli* MG1655::*tetA* grows in the presence of high concentrations of OCT. This demand is being met by changes in flux in both the Entner-Doudoroff (E-D) and oxidative pentose pathways. However, these flux rearrangements do not follow a conventional view of the functioning of the E-D pathway; although the first steps, R3-R5 increase in flux, the subsequent steps, R6-R9, decrease. This is compensated for by additional consumption of GAP, by increased activity in the OPPP, whose fluxes also increase. The result of these changes is an increased flux in reactions of the electron transport chain (ETC) and oxidative phosphorylation (OP) utilising the increased production of reducing equivalents.

It is also of note that in addition to reactions that might be expected to be associated with increased metabolic stress, the network also contains fructose-6-phosphate aldolase (R19) and dihydroxyacetone kinase (R11). Both of the enzymes responsible for these reactions have been relatively recently discovered^56,57^ and whose metabolic role is as yet not clearly understood.

Of the reactions associated with the *pur*-genes, only one, phosphoribosyl pyrophosphate synthetase (*prs*) showed an increase in flux in the model analysis, and this response was common to both the WT and the oxytetracycling resistant MG1655::*tetA* strain. The fact that increases in expression were not reflected in changes in calculated flux are not surprising for two reasons: Firstly, even if an increase in transcript level does lead to an increase in enzyme activity, the flux catalysed by that enzyme is a function of the activity of all enzymes in the network and not that of the enzyme alone; a major motivation in the design of the model analysis was to reflect this interdependency. Secondly these reactions are ultimately contributing to nucleotide and amino acid components of the biomass, and as the proportion of these was fixed, this places additional constraints on the values that these reaction fluxes can take.

In summary, the results of the study showed that growth in the presence of ½ MIC of OTC resulted in profound changes in gene expression in the OTC resistant *E. coli* MG1655::*tetA*. This suggests that pumping out of OCT using the tetracycline specific pump TetA requires highly adaptive responses, and this may render the bacterium vulnerable in other parts of the cell than those targeted by the OTC. Indeed, we identified combinations of antibiotics (chloramphenicol and gentamycin), where even sub-therapeutic concentrations of the other drug were shown to increase the OTC susceptibility. The study investigated a novel approach to characterize the metabolic adaptation in antimicrobial resistant bacteria. It demonstrated that metabolic modeling with incorporated transcription data identified adaptive reactions that were not obvious from just looking at up and down regulated genes. On the contrary, the pathways which were identified using metabolic modeling were generally not controlled by enzymes encoded from genes which were among the most highly regulated genes. We suggest that this approach is generally applicable to studies of adaptation to antimicrobial treatment in antimicrobial resistant bacteria.

## Methods

### Bacterial strains

The strains used in this study are listed in Table 4. The OTC-resistant derivative of MG1655 was described in a previous study^58^. It has *tetA* gene and its repressor gene, *tetR*, cloned into the pseudo gene *ybeM*. Strains were maintained in Difco^TM^ Lysogeny broth (LB), Lennox (Becton, Dickinson and Company, Denmark) and on LB agar plates (Becton, Dickinson and Company, Denmark) supplemented with OTC (10 mg/L) (Sigma, Copenhagen, Denmark) when appropriate.

**Table 4:** Bacterial strains used in this study.

Strain	Genotype	Reference/source
MG1655	*E. coli* MG1655 K-12	^61^
MG1655::*tetA*	*E. coli* MG1655 ∆*ybeM::tetA*	^58^
MG1655::*tetA*∆*PurT*	*E. coli* MG1655 ∆*ybeM::tetA∆purT::*Kan	This study
MG1655::*tetA*∆*PurN*	*E. coli* MG1655 *∆ybeM::tetA ∆purN::*Chl	This study
MG1655::*tetA* ∆*PurT*∆*PurN*	*E. coli* MG1655 *∆ybeM::tetA ∆purT::*Kan *∆purN::*Chl	This study
MG1655::*tetA*∆*ndk*	*E. coli* MG1655 *∆ybeM::tetA* ∆*ndk*::Kan	This study
MG1655::*tetA*∆*aroC*	*E. coli* MG1655 ∆*ybeM::tetA ∆aroC*::Kan	This study
MG1655::*tetA*∆*aroB*	*E. coli* MG1655 ∆*ybeM::tetA* ∆*aroB*::Kan	This study

Site-specific mutants were produced by Lambda Red recombination^59^, resulting in exchange of the gene of interest with chloramphenicol or kanamycin gene cassettes. During strain construction, the medium was supplemented with kanamycin (30 mg/L) or chloramphenicol (10 mg/L) (Sigma, Copenhagen, Denmark) when appropriate. Primer sequences used for Lambda Red mediated mutagenesis and PCR verifications are listed in Table 5. Insertions were confirmed by PCR using standard procedures.

**Table 5:** Primer sequences used for Lambda Red mediated mutagenesis, PCR verifications and RT-qPCR.

Primer name	Sequence	Application
PurN-F	5′-ATGAATATTGTGGTGCTTATTTCCGGCAACGGAAG TAATTGTGTAGGCTGGAGCTGCTTC-3′	Knockout
PurN-R	5′-TTACTCGTCGGCAGCGTAGCCCTGCGGCGGCAG ACGTTGACATATGAATATCCTCCTTAG-3′	Knockout
PurN-check-F	5′-GT ATCGCACCTTCAACTGCGG-3′	Proof of knockout
PurN-check-R	5′-CGAGCAATATTGGCAGATGTCCA-3′	Proof of knockout
PurT-F	5′-ATGACGTTATTAGGCACTGCGCTGCG TCCGGCAGCAACTCGTGTAGGCTGGAGCTGCTTC-3′	Knockout
PurT-R	5′-TTAACCCTGTACTTTT ACCTGTCCGGCGGCGTGCTTCGCGCATATGAATATCCTCCTTAG-3′	Knockout
PurT-check-F	5′-TGCGCGCGGAATTAATCAGGGG-3′	Proof of knockout
PurT-check-R	5′-CGCTGGAAGCGGGCGATTAC-3′	Proof of knockout
Ndk-F	5′-ATGGCTATTGAACGTACTTTTTCCATCAT CAAACCGAACGGTGTAGGCTGGAGCTGCTTC-3′	Knockout
Ndk-R	5′-TTAACGGGTGCGCGGGCACACTTCGCCTTCGCCAAAGAAA CATATGAATATCCTCCTTAG-3′	Knockout
ndk-check-F	5′-CCTTCATCAATAGTCAACGGCCCTG-3′	Proof of knockout
ndk-check-R	5’-GGGTTGAAAAAAGAAACGCCCCGG-3’	Proof of knockout
PhoA-check-F	5′-GTGTGCGCAGGTAGAAGCTTTGGAG-3′	Proof of knockout
PhoA-check-R	5′-CATGAGCGTATGCGCCCGTGATC-3′	Proof of knockout
aroC-F	5′-ATGGCTGGAAACACAATTGGACAACTCTTTCGCGTA ACCAGTGTAGGCTGGAGCTGCTTC-3′	Knockout
aroC-R	5′-TGCCGTAACAGGTGATCCATTAAAACGATCGCC AGCATCGCATATGAATATCCTCCTTAG-3′	Knockout
aroC-check-F	5′-CGGCGGCGATGGTGTGTTTATGC-3′	Proof of knockout
aroC-check-R	5′-CTATCGATTGTGCGCTACCCGGC-3′	Proof of knockout
tyrA/pheA-F	5′-ATGGCTGGAAACACAATTGGACAACTCTTT CGCGTAACCAGTGTAGGCTGGAGCTGCTTC-3′	Knockout
tyrA/pheA-R	5′-ATGGTTGCTGAATTGACCGCATTACGCGATCAAATTGATG CATATGAATATCCTCCTTAG-3′	Knockout
tyrA/pheA-check-F	5′-GGGAGGCGTTTCGTCGTGTGAAAC-3′	Proof of knockout
tyrA/pheA-check-R	5′-CTTCCGAGCAACCGCGCAGTG-3′	Proof of knockout
aroB-F	5′-TTACGCTGATTGACAATCGGCAATGGCGTTA AGAACAAGCGTGTAGGCTGGAGCTGCTTC-3′	Knockout
aroB-R	5′-ATGGAGAGGATTGTCGTTACTCTCGGGGAACGTAG TTACCCATATGAATATCCTCCTTAG-3′	Knockout
aroB-check-F	5′-GATCTGCGGTTCGCCACGTTCAG-3′	Proof of knockout
aroB-check-R	5′-ACACCGCCGCGTGAAGTTCTGG-3′	Proof of knockout
Kanrev-R	5′-CCGCTTCAGTGACAACGTCGAGCACAGC-3′	Proof of knockout
apt-F	5′-CTTGCTGGTTGAGCGTTA-3′	RT-qPCR
apt-R	5′-CTGATCGGTGCCGTATTC-3′	RT-qPCR
hpt-F	5′-ATGAGTTTGTGGTGGGTTAC-3′	RT-qPCR
hpt-F	5′-TCGTCCAGCAGAATCACT-3′	RT-qPCR
gapA-F	5′-ACTGACTGGTATGGCGTTCC-3′	RT-qPCR
gapA-R	5′-GTTGCAGCTTTTTCCAGACG-3′	RT-qPCR
nusG-F	5′-GTCCGTTCGCAGACTTTAAC-3′	RT-qPCR
nusG-R	5′-GCTTTCTCAACCTGACTGAAG-3′	RT-qPCR

### Antimicrobial susceptibility testing

The MICs for OTC, chloramphenicol (Sigma, Copenhagen, Denmark), and gentamicin (Sigma, Copenhagen, Denmark), were determined using the broth-micro-dilution method in the range from 0 to 512 mg/L by 2-fold dilutions increase following the CLSI guidelines, When necessary, more detailed determination with more concentration steps were included, as previous described^11^.

### Checkerboard assay

Checkerboard assays were performed in Mueller-Hinton II (MH-2) (Sigma, Copenhagen, Denmark) broth with two different antibiotic combinations: OTC and chloramphenicol, and OTC and gentamicin, as previously described^11^. The panel was incubated aerobically at 37 °C for 18–22 hours, without shaking. The fractional inhibitory concentration (FIC) index was defined as; ∑FIC = FIC A + FIC B, where FIC A is the MIC of drug A in the combination divided by the MIC of drug A alone, and FIC B is the MIC of drug B in the combination divided by the MIC of drug B alone^60^. The antibiotic combinations were interpreted as synergetic when the ∑FIC ≤ 0.5, indifferent when the ∑FIC > 0.5–4, and antagonistic when the ∑FIC > 4^32^.

### Growth conditions

In order to obtain RNA for expression analysis, the strains were grown in 250 mL flasks containing 100 mL of MH-2 broth either without OTC or with OTC corresponding to ½ MIC of the OTC-resistant strain (14 mg/L) or ½ MIC of the wild type strain MG1655 (0.25 mg/L^61^). Growth was at 37 °C and with shaking (225 rpm). The flasks were inoculated with pre-cultures grown for 2 hours at 37 °C and 225 rpm to a final cell density of 10^5^ CFU/mL, as determined by use of Sensititre™ Nephelometer (Thermo Scientific™) with a McFarland 0.5 standard. The cultures were monitored by measuring optical density until samples reached the logarithmic phase (OD_600nm_ = 0.5–0.6), where aliquots were taken for RNA extraction. Biological duplicates were performed.

Growth for characterization of growth-responses to treatment were performed in Mueller-Hinton II (MH-2) on a BioScreen C^TM^ for 24 hours at 37 °C as previously described^11^. The cultures were supplemented with OTC (Sigma, Copenhagen, Denmark) representing ½ MIC of the corresponding strain when appropriate. Strains were also grown in M9 minimal media (2 mM MgSO_4_, 0.1 mM CaCl_2_, 0.4% glucose, 8.5 mM NaCl, 42 mM Na_2_HPO_4_, 22 mM KH_2_PO_4_, 18.6 mM NH_4_Cl). Lag phase of growth curves was defined as the time necessary to reach an OD_600_ of 0.1. Statistical comparison of lag phase was performed by one-way ANOVA using GraphPad Prism. Growth characterization experiments were performed with three biological replicates and with three technical repeats for each of these.

### RNA extraction

RNA extraction for RNA seq was performed essentially as previously described^11^. Briefly, 3 mL cell samples were mixed with RNAlater (Ambion®, Naerum, Denmark). Total RNA was extracted from two independently grown samples using a FastPrep system (Qbiogene, Illkirch, France) and an RNeasy Mini kit (Qiagen, Sollentuna, Sweden), and quantity and quality of RNA was determined using a NanoDrop 1000 spectrophotometer (Thermo Scientific, Hvidovre, Denmark). After DNase treatment (Thermo Scientific, Hvidovre, Denmark), rRNAs (23S and 16S) were removed by subtractive hybridization using the MICROBExpress kit (Ambion®, Naerum, Denmark). Removal of rRNAs was confirmed with an Agilent 2100 Bioanalyzer (Agilent Technologies, Glostrup, Denmark).

### RT-qPCR

Purified RNA was reverse-transcribed into cDNA using the High Capacity cDNA Reverse Transcription Kit (life Technologies, Naerum, Denmark). RT-qPCR was performed using FastStart Essential DNA Green Master (Roche, Hvidovre, Denmark). *gapA* and *nusG* were used as reference genes. RT-qPCR was performed twice by two separate biological samples and the results were caiculated by the 2^−∆∆CT^ method as described by Pfallf^62^. Primer sequences can be found in the Table 5.

### Library preparation and RNA sequencing

The library for RNA-Seq was prepared using the TruSeq RNA Sample Preparation kit (Illumina, Little Chesterford, United Kingdom), following the manufacturer’s instructions. The samples were validated after each step using an Agilent 2100 Bioanalyzer (Agilent Technologies, Glostrup, Denmark), and the final concentration of mRNA was measured using a Qubit 2.0 Fluorometer (Invitrogen, Naerum, Denmark). The libraries were sequenced using the Illumina HiSeq. 2000 platform with a single-end protocol, multiplexing and read lengths of 100 nt.

### Mapping of reads and differential expression analysis

The analysis was made on libraries consisting of 2.5–3 million unique reads with a length of 100 bp. A total of 4482 genes were considered in the analysis. Reads were trimmed for adaptor sequences using the CLC Genomics Workbench (CLC bio, Aarhus, Denmark). The trimmed reads were mapped onto the *E. coli* MG1655 genome (RefSeq Accession No. NC_000913) as previously described^11^. Briefly, hits of reads to annotated genes were counted and all rRNA read counts were removed to obtain a proper normalization. The data was normalized using the conditional quantile normalization method, while also taking into account difference in replicate library sizes and gene GC content using edgeR bioconductor software package for R^63^. The differential expression of each annotated mRNA transcript was calculated using EdgeR and significantly regulated genes were selected based on the false discovery rate (FDR) (Benjamini–Hochberg multiple testing correction)^64^ using a threshold of 0.05. The resulting datasets from analysis of MG1655::*tetA* grown with and without 14 mg/L OTC and MG1655 growin with 0.25 mg/L OTC are included as supplementary material, Tables S5 and S6. Functional enrichment analysis using gene ontology (GO) categories (biological process, molecular function, cellular process, KEGG pathway) were analysed using the Cytoscape plugin BINGO^65^. EcoCyC database^66^ was used to further analyse the functions of differentially expressed genes.

### Model construction

The *E. coli* K-12 MG1655 genome scale model was constructed in a modular way using already described techniques^67,68^. The final model consisted of (1) A top-level module which contain information about the other model modules and its prime function was to import other modules; (2) A module containing all the automatically generated reactions (see below); (3) A module containing all transport reactions importing nutrients and exporting biomass components and metabolic by-products; (4) A module containing the electron transport chain, and (5) A module containing additional and modified reactions.

The final GSM describes a metabolic network which can generate all *E. coli* biomass components from a rich medium (Mueller Hinton broth 2 (MH-2)), consisting of glucose, amino acids (aspartic acid, theronine, serine, glutamic acid, proline, glycine, alanine, cysterine, valine, methionine, isoleusine, leusine, tyrosine, phenylalanine, histidine, lysine, arginine and tryptophan)^69,70^, phosphate and O_2_.

Details on model construction can be seen in Supplementary Material, Table S7.

### Incorporation of transcriptomics data into model and analysis of model

#### Calculating the weighting factor

The weighting factor wr, for a given reaction, r, was calculated as:

$$wr=1\times \mathop{\prod }\limits_{g=0}^{g=n}{2}^{-lfcg}$$

where lfcg represents the observed log fold change for any gene that codes for enzymes that catalyse reaction, r, and which had a FDR < 0.05. As linear programs are defined as a minimization-problem in flux balance analysis, the weighing factor was negated so that up-regulated genes carry a lower weighting and vice-versa. The first multiplication in the equation ensured a default weight of one in the event that there were no significantly changed genes for a reaction.

*Defining the linear program:*

1 $$\begin{array}{c}min.:\Sigma |vi.wi|\\ {\rm{s}}.{\rm{t}}.\{\begin{array}{ll}{\rm{Nv}}=0 & \\ {{\rm{v}}}_{{\rm{i}}}\ge 0, & {\rm{\yen i}}\,{\rm{\varepsilon }}\,\{{\rm{irrevs}}\}\\ {{\rm{v}}}_{{\rm{t}}}={{\rm{bio}}}_{{\rm{t}}}, & \end{array}\end{array}$$

Stating to minimize flux, where w_i_ is the weighting factor described above and subject to three constrains: The first constraint specified stead-state, the second ensured that irreversible reactions carry positive flux and the third constraint represents the export of biomass components. The problem was solved twice, with and without w_i_, and the solutions compared to identify up or down regulated reactions. This set of reaction was then used to construct a regulated sub-model.

#### Constructing regulated sub-networks

The set of reactions identified above could not *a priori* be guaranteed to represent a balanced network as a result of constant fluxes of metabolites exchanged between the regulated and non-regulated sub-networks. However, these could be identified by constructing a stoichiometry matrix, N’ constructed from the reactions in the regulated sub-network. We could then calculate:

$${\rm{s}}{\prime} ={\rm{N}}{\prime} .{\rm{v}}{\prime} $$

The non-zero elements of s′ corresponded to unbalanced metabolites, and hence required additional transport reactions to be added to N′. Their values corresponded to the negative flux to be carried by these transporters in order to generate a regulated sub-network, such that:

$${{\rm{N}}}^{{\rm{r}}}.{{\rm{v}}}^{{\rm{r}}}=0$$

where N^r^ represents the stoichiometry matrix of the regulated sub-network with the additional transporters included, and v^r^ the corresponding flux vector. In this way we generated not only fully a balanced sub-model, but also a corresponding steady-state flux vector.

**Figure 4:**
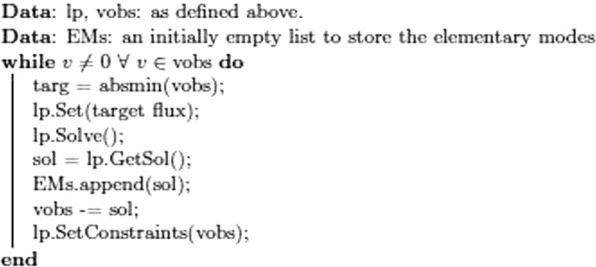
Modeling approach to identification elementary nodes. At each iteration, sol represents an EM accounting for the flux in targ, as well the fluxes of the reactions in the EM. We then subtract this solution from vobs, guaranteeing that at least one more reaction in vobs now has zero flux and use the new vobs as a new set of constraints in lp. We continue with this, until all in vobs become zero.

The sub-models proved hard to analyse, presumably due to large numbers of additional transporters included, and correlation trees with standard algorithms for flux balance analysis were inconclusive. To solve this problem, we generated a new algorithm, taking advantage of the fact that our analysis had created a valid steady-state flux vector, and based on the assumption that any Linear Programming solution in which only a single reaction is fixed will represent an elementary mode (EM). The novel algorithm also generated a set of fluxes corresponding to reactions in the newly determined EM, so as well as identifying the EMs we could assign fluxes to them. The calculations were as follows: Given an initial (observed) steady-state flux vector, vobs, define an LP problem:

2 $${\rm{lp}}=\begin{array}{l}\min \,.|v|\\ {\rm{s}}{\rm{.t}}.\{\begin{array}{ll}{\rm{Nv}}=0 & \\ 0\ge {{\rm{v}}}_{{\rm{t}}}\le {{\rm{vobs}}}_{{\rm{t}}} & {\rm{\yen }}i\,{\rm{\varepsilon }}\,{{\rm{vobs}}}_{{\rm{t}}} > 0\\ {\rm{s}}{\rm{.t}}.\,0\le {v}_{t}\ge {{\rm{vobs}}}_{{\rm{t}}} & {\rm{\yen }}i\,{\rm{\varepsilon }}\,{{\rm{vobs}}}_{{\rm{t}}} < 0\\ {{\rm{v}}}_{{\rm{targ}}}=\,{\rm{\min }}\,.(|v|) & {\rm{\yen }}i\,{\rm{\varepsilon }}\,{{\rm{vobs}}}_{{\rm{t}}}\end{array}\end{array}$$

ie, we set a target reaction, v_targ_ to the minimum (absolute) flux in vobs, and minimise the total absolute flux in the system subject to the constraint that no flux in the solution can take an absolute value greater than that in vobs, and that the sign of the reaction flux in the solution is the same as that in the observed. We can now proceed as shown in Fig. 4.

## Supplementary information

Supplementary Dataset 1.

## Data Availability

All data are included in the main document and supplementary material.
