# Supplementary material for: Global responses to oxytetracycline treatment in tetracycline-resistant Escherichia coli
Source: Sci Rep. 2020 May 21;10:8438. doi: 10.1038/s41598-020-64995-1 (PMC7242477; doi:10.1038/s41598-020-64995-1)
Supplement: Supplementary file 1 — Supplementary Dataset 1. [file 41598_2020_64995_MOESM1_ESM.pdf]

Supplementary material:

# Global responses to oxytetracycline treatment in tetracycline-resistant *Escherichia coli*

Thea S. B. Møller, Gang Liu, Hassan B. Hartman, Martin H. Rau, Sisse Mortensen, Kristian Thamsborg, Andreas E. Johansen, Morten O. A. Sommer, Luca Guardabassi Mark G. Poolman and John E. Olsen

Supplementary Figure S1.

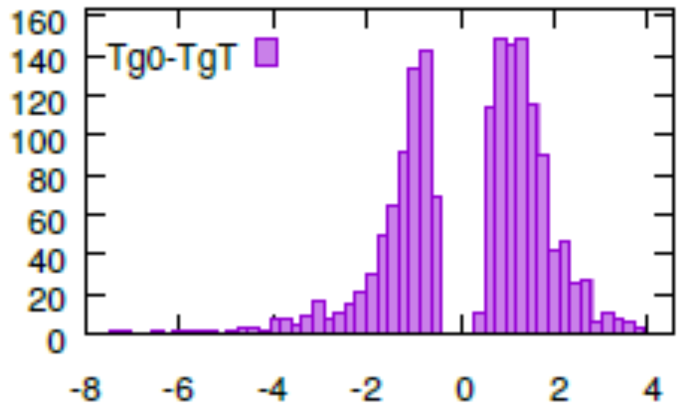


Supplementary Figure S1. Distribution of $Log_2FC$ changes among significantly regulated genes when MG1655::*tetA* was grown with 14 mg/L OTC.

Supplementary figure S2. Expression analysis of genes of the purine salvage pathway.

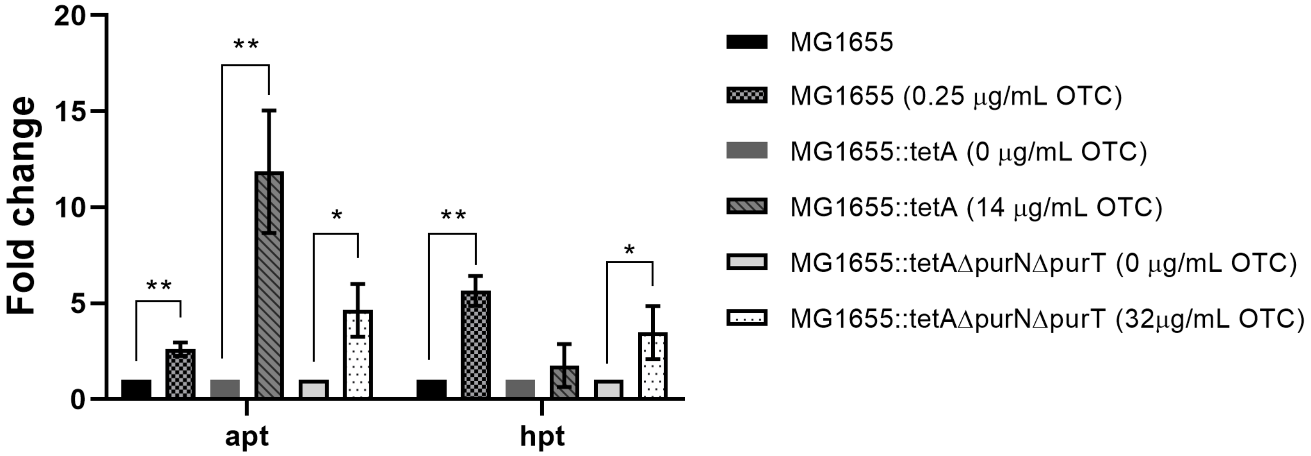

Supplementary Figure S1. Results of synergy tests.

(a)

| | | MG1655/TetA | | | | | | | | | | | |
|---|---|---|---|---|---|---|---|---|---|---|---|---|---|
| | | TET | | | | | | | | | | | |
| | Conc. | 0 | 0.25 | 0.5 | 1 | 2 | 4 | 8 | 16 | 32 | 64 | 128 | 256 |
| CHL | 0 | | | | | | | | | MIC A | | | |
| | 0.125 | | | | | | | | | ∑FIC = 1.03 | | | |
| | 0.25 | | | | | | | | | ∑FIC = 1.06 | | | |
| | 0.5 | | | | | | | | ∑FIC = 0.63 | | | | |
| | 1 | | | | | | | | ∑FIC = 0.75 | | | | |
| | 2 | | | | | | ∑FIC = 0.63 | | | | | | |
| | 4 | MIC B | | | | | | | | | | | |
| | 8 | | | | | | | | | | | | |

(b)

| | | MG1655/TetA | | | | | | | | | | | |
|---|---|---|---|---|---|---|---|---|---|---|---|---|---|
| | | TET | | | | | | | | | | | |
| | Conc. | 0 | 0.25 | 0.5 | 1 | 2 | 4 | 8 | 16 | 32 | 64 | 128 | 256 |
| GM | 0 | | | | | | | | | MIC A | | | |
| | 0.25 | | | | | | | | ∑FIC = 0.63 | | | | |
| | 0.5 | | | | | | | ∑FIC = 0.50 | | | | | |
| | 1 | | ∑FIC = 0.50 | | | | | | | | | | |
| | 2 | MIC B | | | | | | | | | | | |
| | 4 | | | | | | | | | | | | |
| | 8 | | | | | | | | | | | | |
| | 16 | | | | | | | | | | | | |

**Significantly (FDR<0.05) up-regulated genes in *E. coli* MG1655::*tetA* grow with 14 mg/L oxytetracycline, corresponding to 1/2 MIC of the strain.**

| Gene | logFC | FDR | Reaction(s) |
|---|---|---|---|
| ydcI | 3,940139206 | 6,16E-08 | |
| prpB | 3,801603072 | 9,85E-21 | METHYLISOCITRATE-LYASE-RXN |
| fadB | 3,747103648 | 0,00000149 | RXN-14393, OHBUTYRYL-COA-EPIM-RXN, OHACYL-COA-DEHYDROG-RXN, ENOYL-COA-HYDRAT-RXN, RXN0-5393, |
| efeO | 3,705585586 | 0,000000143 | |
| cyoA | 3,704541592 | 0,000000118 | RXN0-5268 |
| bfd | 3,678281898 | 0,0000473 | |
| ndk | 3,616347173 | 0,000000778 | NUCLEOSIDE-DIP-KIN-RXN, UDPKIN-RXN, CDPKIN-RXN, DUDPKIN-RXN, DCDPKIN-RXN, DTDPKIN-RXN, DADPKIN-RXN, |
| puuA | 3,546276925 | 1,75E-08 | RXN0-3901 |
| sdhC | 3,536381219 | 0,00000149 | SUCCINATE-DEHYDROGENASE-UBIQUINONE-RXN |
| mdtQ | 3,45331784 | 0,00000389 | |
| lldP | 3,40054561 | 0,000258264 | TRANS-RXN-105, TRANS-RXN0-515, TRANS-RXN-104 |
| sdaB | 3,374004892 | 0,000000162 | RXN-15125, 4.3.1.17-RXN |
| fhuF | 3,325717701 | 0,000460388 | |
| tnaB | 3,312122497 | 0,00068003 | TRANS-RXN-76 |
| efeU | 3,289641487 | 0,00000147 | TRANS-RXN-121A, TRANS-RXN-121 |
| ddpX | 3,257541441 | 0,0000247 | 3.4.13.22-RXN |
| astC | 3,244361333 | 0,0000124 | SUCCORNTRANSAM-RXN, ACETYLORNTRANSAM-RXN |
| bioB | 3,232663875 | 7,98E-14 | 2.8.1.6-RXN |
| purK | 3,201604528 | 3,61E-11 | RXN0-742 |
| sdhD | 3,192341682 | 0,00000077 | SUCCINATE-DEHYDROGENASE-UBIQUINONE-RXN |
| purM | 3,16063697 | 5,83E-09 | AIRS-RXN |
| yncE | 3,130581578 | 0,00000871 | |
| paaA | 3,120944613 | 0,0000234 | RXN0-2042 |
| carA | 3,08942406 | 2,13E-09 | CARBPSYN-RXN |
| hcaR | 3,08442948 | 4,33E-09 | |
| purH | 3,044494548 | 8,74E-08 | AICARTRANSFORM-RXN, IMPCYCLOHYDROLASE-RXN |
| nrdH | 2,988725084 | 0,000035 | |
| yjfZ | 2,948626815 | 0,000460339 | |
| codB | 2,881675044 | 1,31E-08 | TRANS-RXN-116 |
| dadA | 2,86517943 | 9,88E-08 | RXN-11193, DALADEHYDROG-RXN |
| ymdA | 2,864012926 | 0,00076728 | |
| entC | 2,841324991 | 0,0000463 | ISOCHORSYN-RXN |
| yaiS | 2,775109265 | 0,01099414 | |
| purE | 2,762873952 | 0,000000498 | RXN0-743 |
| csgE | 2,760650637 | 0,000284719 | RXN-5068 |
| putP | 2,754190795 | 0,0000356 | TRANS-RXN-118, TRANS-RXN0-505 |
| cvpA | 2,748157014 | 1,31E-08 | |
| sfmH | 2,740562323 | 0,001688553 | |
| puuD | 2,738052883 | 0,000000294 | RXN0-3942 |
| rplC | 2,723482917 | 1,74E-09 | |
| bioD | 2,721171524 | 3,06E-08 | DETHIOBIOTIN-SYN-RXN |
| dctA | 2,71077447 | 0,00000137 | TRANS-RXN0-517, TRANS-RXN0-451, TRANS-RXN-122A, TRANS-RXN-121C, TRANS-RXN-121B, |
| mglA | 2,704746941 | 0,012734777 | TRANS-RXN0-541, ABC-18-RXN |
| feoA | 2,698921457 | 0,000196883 | |

| | | | |
|---|---|---|---|
| astA | 2,692549637 | 0,0000196 | ARGININE-N-SUCCINYLTRANSFERASE-RXN |
| purF | 2,673868008 | 5,46E-10 | PRPPAMIDOTRANS-RXN |
| rsxB | 2,652351777 | 9,89E-10 | |
| yjeT | 2,652144212 | 0,006195009 | |
| purN | 2,638108 | 7,07E-13 | GART-RXN |
| fecA | 2,636498337 | 0,00000173 | RXN0-1684, RXN0-2261 |
| csgF | 2,630372192 | 0,000124675 | RXN-5068 |
| ydfU | 2,621914732 | 0,000614812 | |
| puuR | 2,596379507 | 8,53E-08 | |
| tonB | 2,589333101 | 0,00013605 | RXN0-1702, TRANS-RXN0-488, RXN0-1684, RXN0-2261, RXN0-1701, RXN0-2241, RXN0-1682, RXN0-2181, |
| pyrC | 2,588662616 | 0,000000132 | DIHYDROOROT-RXN |
| yhdV | 2,586052395 | 0,0000475 | |
| cyoB | 2,584776986 | 0,00000261 | RXN0-5268 |
| yigI | 2,583416167 | 0,000957034 | |
| yghT | 2,581308212 | 0,023992738 | |
| ymfA | 2,564956486 | 0,005653204 | |
| rpsA | 2,554601697 | 0,0000366 | |
| iscR | 2,547985342 | 0,00000437 | |
| rplD | 2,537826119 | 2,93E-08 | |
| rutC | 2,519726238 | 0,00796857 | RXN0-6461 |
| xanP | 2,512156196 | 6,15E-09 | RXN-5076 |
| agaA | 2,507228289 | 0,04863325 | |
| hofN | 2,46499312 | 0,001923884 | |
| purT | 2,458865821 | 0,00000367 | GARTRANSFORMYL2-RXN, ACETATEKIN-RXN |
| rimO | 2,45008684 | 4,52E-11 | RXN0-6366 |
| mhpR | 2,443140092 | 0,00000249 | |
| yegL | 2,440058973 | 0,001475841 | |
| alr | 2,438210243 | 0,035468997 | ALARACECAT-RXN |
| isrC | 2,431092687 | 0,00519417 | |
| plaP | 2,429626716 | 1,25E-10 | TRANS-RXN-69 |
| bioF | 2,426481921 | 0,00000101 | RXN-11484, 7KAPSYN-RXN |
| iscS | 2,42310106 | 0,00000387 | RXN-12587, RXN-12588, RXN-14382, RXN-9787, RXN0-308 |
| cycA | 2,394575466 | 6,15E-09 | RXN0-5203, RXN0-5202, RXN0-5201, RXN0-5130, TRANS-RXN-62B, TRANS-RXN-62A |
| rplW | 2,390459264 | 6,26E-08 | |
| prs | 2,387285598 | 1,76E-10 | PRPPSYN-RXN |
| gtrB | 2,38654423 | 0,000000579 | |
| fecI | 2,384874267 | 0,000226096 | |
| dadX | 2,368037846 | 0,000000639 | ALARACECAT-RXN |
| bioA | 2,35592396 | 0,00000133 | DAPASYN-RXN |
| ybeM | 2,353043676 | 2,13E-13 | |
| yjcD | 2,343145358 | 1,03E-11 | 2.6.1.57-RXN, BRANCHED-CHAINAMINOTRANSFERLEU-RXN, TYROSINE-AMINOTRANSFERASE-RXN, |
| fes | 2,32761838 | 0,00013366 | RXN0-6938, RXN0-1661 |
| purB | 2,323771041 | 7,09E-08 | AMPSYN-RXN, AICARSYN-RXN |
| yqeF | 2,316770829 | 0,000000296 | |
| trxB | 2,314191001 | 2,64E-08 | THIOREDOXIN-REDUCT-NADPH-RXN |
| fliE | 2,313849378 | 0,047649707 | |
| sdaC | 2,309630635 | 3,44E-08 | TRANS-RXN-71 |
| rpmA | 2,308405487 | 8,47E-09 | |
| cheB | 2,294231515 | 0,00221487 | MCPTAP-RXN, MCPTAR-RXN, MCPTRG-RXN, MCPTSR-RXN, CHEBTSRD-RXN, CHEBTRGD-RXN, |

| | | | | |
|---|---|---|---|---|
| rplU | 2,289297138 | 0,00000031 | | |
| fadA | 2,287939193 | 0,0000875 | RXN-14394, KETOACYLCOATHIOL-RXN | |
| kgtP | 2,283191928 | 0,000000667 | TRANS-RXN-23 | |
| prfC | 2,27959889 | 4,6E-14 | | |
| fepC | 2,255289218 | 0,000160615 | ABC-10-RXN, RXN0-2181 | |
| sthA | 2,250379082 | 0,000000376 | PYRNUTRANSHYDROGEN-RXN | |
| rpsG | 2,241794186 | 0,000000244 | | |
| mutT | 2,230323745 | 0,0000154 | RXN-14002, RXN-12816, RXN-11397, RXN-11396 | |
| rnb | 2,221537035 | 7,1E-14 | RXN0-6524, RXN0-6479, 3.1.13.1-RXN | |
| rpsJ | 2,219922569 | 6,79E-08 | | |
| accC | 2,219903192 | 1,1E-09 | BIOTIN-CARBOXYL-RXN, ACETYL-COA-CARBOXYLTRANSFER-RXN | |
| yjiH | 2,214946106 | 0,014548906 | | |
| yadM | 2,206585999 | 0,005065689 | | |
| fis | 2,204807761 | 0,00000375 | | |
| yfjY | 2,203804788 | 0,03324675 | | |
| tig | 2,203342997 | 8,28E-08 | PEPTIDYLPROLYL-ISOMERASE-RXN | |
| atpH | 2,194878174 | 0,00000101 | TRANS-RXN-249, RXN0-7041 | |
| purD | 2,185715549 | 0,0000682 | GLYRIBONUCSYN-RXN | |
| rsxC | 2,18426479 | 8,32E-12 | | |
| purR | 2,181859917 | 2,75E-09 | | |
| argT | 2,176386285 | 0,000000972 | ABC-37-RXN, ABC-4-RXN, ABC-3-RXN | |
| rpsL | 2,175531944 | 0,000000188 | | |
| aldA | 2,172924499 | 0,000101598 | GLYCOLALD-DEHYDROG-RXN, LACTALDDEHYDROG-RXN | |
| yceA | 2,168235324 | 0,000000888 | | |
| rpmG | 2,164699006 | 0,00000159 | | |
| ycgZ | 2,158697547 | 0,004395783 | | |
| yqjH | 2,15825054 | 0,0000349 | RXN0-6941, RXN0-6555 | |
| agaB | 2,155347292 | 0,037565394 | | |
| pyrF | 2,154794175 | 1,52E-08 | OROTPDECARB-RXN | |
| ybeR | 2,154680713 | 0,045034778 | | |
| flhE | 2,149462124 | 0,018315361 | | |
| prpD | 2,139092712 | 0,00000664 | 2-METHYLCITRATE-DEHYDRATASE-RXN | TRANS-RXN0-283, RXN0-1565, TRANS-RXN0-455, RXN0-2121 |
| yciH | 2,136372169 | 0,000055 | | |
| apt | 2,13607488 | 0,0000176 | ADENPRIBOSYLTRAN-RXN | |
| wcaH | 2,133815241 | 0,009211067 | | |
| efeB | 2,132442266 | 0,0000574 | RXN0-6258 | |
| yidC | 2,131835322 | 4,96E-08 | | |
| folK | 2,129021708 | 0,0000205 | H2PTERIDINEPYROPHOSPHOKIN-RXN | |
| exbD | 2,111782871 | 0,00000662 | RXN0-1702, TRANS-RXN0-488, RXN0-1684, RXN0-2261, RXN0-1701, RXN0-2241, RXN0-1682, RXN0-2181, | |
| rpsT | 2,102907404 | 0,000282861 | | |
| carB | 2,102825144 | 0,000198387 | CARBPSYN-RXN | |
| prpR | 2,087922698 | 0,00000753 | | |
| gtrA | 2,082539376 | 0,00431345 | | |
| rpsS | 2,08074578 | 0,000000439 | | |
| fliS | 2,07930304 | 0,013614267 | | |
| rplB | 2,077949789 | 0,00000121 | | |
| atpF | 2,069551354 | 0,000000212 | TRANS-RXN-249, RXN0-7041 | |
| yfaT | 2,059966771 | 0,035295171 | | |
| pgaC | 2,057756061 | 0,001078798 | TRANS-RXN0-549, RXN0-5413 | |

| | | | | | |
|---|---|---|---|---|---|
| atpE | 2,037966477 | 2,67E-08 | TRANS-RXN-249, RXN0-7041 | | |
| hofO | 2,025012541 | 0,027103336 | | | |
| sroH | 2,023632369 | 0,001533445 | | | |
| rplV | 2,020918965 | 0,00000104 | | | |
| fadD | 2,01703414 | 5,15E-08 | ACYLCOASYN-RXN | | |
| adk | 2,012585735 | 0,000000272 | ADENYL-KIN-RXN, RXN-14074 | | |
| entS | 2,001469376 | 0,000127617 | TRANS-RXN0-496, RXN0-8 | | |
| yjjZ | 1,999550381 | 0,035136797 | | | |
| phoE | 1,992680656 | 0,000658304 | RXN0-2481 | | |
| dgcZ | 1,992368681 | 0,00000172 | RXN0-5359 | | |
| sdhA | 1,985857614 | 0,00023863 | SUCCINATE-DEHYDROGENASE-UBIQUINONE-RXN | | |
| purL | 1,977194269 | 0,00000121 | FGAMSYN-RXN | | |
| typA | 1,977091373 | 1,16E-09 | | | |
| yfiM | 1,970637291 | 0,000035 | | | |
| yedE | 1,968837076 | 9,99E-08 | | | |
| pgaD | 1,958247375 | 0,002227984 | TRANS-RXN0-549, RXN0-5413 | CHEBTARD-RXN, CHEBTAPD-RXN, CHEBDEAMID-RXN, MCPMETEST-RX | |
| ddpA | 1,954628445 | 0,000998072 | | | |
| yliF | 1,951941078 | 0,002394093 | | | |
| metK | 1,950864559 | 0,001303741 | S-ADENMETSYN-RXN | | |
| ecpB | 1,946594065 | 0,018933505 | | | |
| aceB | 1,942225301 | 0,00000496 | MALSYN-RXN | | |
| rplF | 1,93381188 | 0,00000547 | | | |
| gcd | 1,922249548 | 0,0000236 | RXN0-6373 | | |
| ldrD | 1,918187331 | 0,012845082 | | | |
| feaR | 1,91596219 | 0,000521076 | | | |
| sfmC | 1,910596351 | 0,0444514 | | | |
| ygiQ | 1,901382345 | 2,62E-09 | | | |
| fliI | 1,899494931 | 0,027101503 | | | |
| rplE | 1,893729005 | 0,00000279 | | | |
| lldR | 1,892562529 | 0,022416725 | | | |
| iscU | 1,8847532 | 0,000000715 | | | |
| kefG | 1,884417123 | 0,00000371 | | | |
| yncD | 1,879790553 | 0,0000105 | | | |
| psrO | 1,873330691 | 0,001024743 | | | |
| rimM | 1,870898258 | 0,000000965 | | | |
| proV | 1,868008224 | 0,00000342 | RXN-8638, ABC-26-RXN | | |
| atpB | 1,865437567 | 0,0000048 | TRANS-RXN-249, RXN0-7041 | | |
| ycaO | 1,858818321 | 6,15E-09 | | | |
| rpsH | 1,851475897 | 0,00000194 | | | |
| ygeO | 1,846741797 | 0,04729126 | | | |
| proW | 1,846733455 | 0,00000291 | RXN-8638, ABC-26-RXN | | |
| cbdX | 1,844936813 | 0,009019944 | | | |
| dacA | 1,844240042 | 6,71E-08 | BETA-LACTAMASE-RXN, 3.4.16.4-RXN | | |
| msrB | 1,839865106 | 9,88E-08 | 1.8.4.12-RXN, 1.8.4.14-RXN | | |
| accB | 1,83512683 | 1,89E-08 | ACETYL-COA-CARBOXYLTRANSFER-RXN | | |
| hscB | 1,829691398 | 0,000866117 | | | PHEAMINOTRANS-RXN |
| yffN | 1,826499331 | 0,013355079 | | | |
| betI | 1,826145429 | 0,0000586 | | | |
| purP | 1,825633956 | 6,26E-12 | TRANS-RXN-44 | | |

| | | | |
|---|---|---|---|
| ylaC | 1,825074088 | 1,74E-09 | |
| yhgF | 1,82391496 | 6,98E-09 | |
| flgI | 1,82097845 | 0,005133098 | |
| ansP | 1,811091053 | 1,78E-08 | TRANS-RXN-262 |
| lpxT | 1,810990341 | 0,000176886 | RXN0-5383 |
| thiP | 1,810915445 | 0,000018 | ABC-32-RXN |
| emrY | 1,806429672 | 0,024665644 | TRANS-RXN-92 |
| cbdB | 1,806332286 | 0,000000291 | |
| entE | 1,805760072 | 0,007849724 | RXN0-6442, ISOCHORMAT-RXN, ENTMULTI-RXN, DHBAMPLIG-RXN, RXN0-5208 |
| lysS | 1,805549216 | 1,67E-08 | LYSINE--TRNA-LIGASE-RXN |
| fklB | 1,799251549 | 0,000000436 | PEPTIDYLPROLYL-ISOMERASE-RXN |
| rpsI | 1,797991451 | 0,00000291 | |
| glyA | 1,794723561 | 0,00120618 | GLYOHMETRANS-RXN, RXN-6321, RXN0-5240, RXN0-5234 |
| atpA | 1,791620726 | 0,0000657 | TRANS-RXN-249, RXN0-7041 |
| ygbE | 1,783199267 | 0,000918789 | |
| ymgC | 1,783034605 | 0,032742119 | TRANS-RXN0-283, RXN0-1565, TRANS-RXN0-455, RXN0-2121 |
| agaD | 1,781248238 | 0,044852952 | |
| murA | 1,779388755 | 1,78E-08 | UDPNACETYLGLUCOSAMENOLPYRTRANS-RXN |
| rpsC | 1,778943943 | 0,0000117 | |
| waaH | 1,777602619 | 0,001894608 | RXN-14361 |
| fecR | 1,77636322 | 0,000695124 | |
| fadL | 1,768749792 | 0,000295641 | RXN0-1802 |
| ugpE | 1,766505756 | 0,001642145 | TRANS-RXN0-573, ABC-34-RXN |
| codA | 1,7657278 | 0,000000781 | CYTDEAM-RXN, RXN0-6708 |
| yeaC | 1,765655658 | 0,000000778 | |
| mdtL | 1,765338434 | 0,000630168 | TRANS-RXN-44 |
| fimC | 1,756303228 | 0,003828839 | |
| rplQ | 1,755720339 | 0,00000149 | |
| yggP | 1,752282798 | 0,000815969 | |
| rplX | 1,749616815 | 0,000014 | |
| ugpB | 1,747374666 | 0,000386139 | TRANS-RXN0-573, ABC-34-RXN |
| rplM | 1,746546148 | 0,00000917 | |
| yhdT | 1,745888393 | 0,015008896 | |
| guaB | 1,740692519 | 0,000163339 | IMP-DEHYDROG-RXN |
| pyrD | 1,737081531 | 0,000131084 | RXN0-6491, RXN0-6554 |
| yzgL | 1,733712715 | 0,020583372 | |
| bioC | 1,733043579 | 0,002749112 | RXN-11475 |
| rplN | 1,730557557 | 0,00000307 | |
| yceD | 1,730271963 | 7,23E-08 | |
| yidD | 1,72927652 | 0,015039001 | |
| ydfV | 1,728269821 | 0,018610589 | |
| yqeG | 1,725676841 | 0,000000526 | |
| puuP | 1,720201359 | 0,00206469 | TRANS-RXN-69 |
| ybfP | 1,717103709 | 0,007385385 | |
| pdhR | 1,714743534 | 0,00041046 | |
| insK | 1,71351213 | 0,033514405 | |
| rfbD | 1,709663996 | 7,6E-13 | DTDPDEHYRHAMREDUCT-RXN, DTDPRHAMSYNTHMULTI-RXN |
| rsxD | 1,708874921 | 0,0000575 | |
| hsdS | 1,706599941 | 0,0000205 | 3.1.21.3-RXN |

| | | | |
|---|---|---|---|
| ybiV | 1,701787763 | 0,0000796 | SUGAR-PHOSPHATASE-RXN |
| rpmH | 1,698436249 | 0,000314856 | |
| betB | 1,69804381 | 0,00000194 | BADH-RXN |
| gltX | 1,696162683 | 1,89E-08 | GLURS-RXN |
| rclC | 1,692859127 | 0,010466719 | |
| ybhC | 1,688311188 | 7,79E-08 | |
| rpsK | 1,688247151 | 0,00000119 | |
| rpoA | 1,685612905 | 0,00000865 | DNA-DIRECTED-RNA-POLYMERASE-RXN |
| rpmB | 1,683057325 | 0,000038 | |
| rzpR | 1,682098251 | 0,024437529 | |
| can | 1,680585175 | 0,0000236 | RXN0-5224 |
| ycjM | 1,678771614 | 0,003269372 | |
| uhpA | 1,677573781 | 0,0000165 | |
| rpsD | 1,677055785 | 0,00000396 | |
| ldtC | 1,671289813 | 0,000000836 | |
| yfgJ | 1,669900483 | 0,0000151 | |
| fliL | 1,668822247 | 0,022258148 | |
| mqo | 1,666624681 | 0,000166806 | MALATE-DEHYDROGENASE-ACCEPTOR-RXN |
| atpG | 1,666151409 | 0,0000431 | TRANS-RXN-249, RXN0-7041 |
| atoD | 1,665527598 | 0,036474132 | ACECOATRANS-RXN, ACETOACETYL-COA-TRANSFER-RXN |
| fabF | 1,662688588 | 0,000000182 | 2.3.1.179-RXN, 3-OXOACYL-ACP-SYNTH-BASE-RXN, 3-OXOACYL-ACP-SYNTH-RXN |
| ygcS | 1,661767573 | 0,004156089 | |
| uhpB | 1,656800105 | 0,00000109 | |
| hpt | 1,656637713 | 1,28E-09 | GUANPRIBOSYLTRAN-RXN, HYPOXANPRIBOSYLTRAN-RXN |
| cdbA | 1,65324365 | 0,00000121 | |
| ugpA | 1,651569266 | 0,015562775 | TRANS-RXN0-573, ABC-34-RXN |
| infA | 1,643392033 | 0,000975487 | |
| yihM | 1,641962449 | 0,000528731 | |
| rho | 1,641910969 | 0,000000452 | |
| ymgA | 1,641403807 | 0,001169203 | |
| sodA | 1,638809289 | 0,001494915 | SUPEROX-DISMUT-RXN |
| hflD | 1,63829012 | 4,03E-09 | |
| fimI | 1,636765362 | 0,0000154 | |
| ynaJ | 1,636181486 | 0,000000436 | |
| tsaB | 1,633883576 | 0,00014238 | RXN0-5522 |
| rnpA | 1,632906699 | 0,002497075 | RXN0-6480, 3.1.26.5-RXN |
| glpG | 1,631852103 | 0,0000059 | RXN0-5103 |
| rsxG | 1,631602186 | 0,0000154 | |
| queA | 1,613741959 | 0,001120893 | RXN0-1342 |
| fumA | 1,611881803 | 0,004476275 | FUMHYDR-RXN, OXALOACETATE-TAUTOMERASE-RXN |
| yggW | 1,610505753 | 0,000000436 | |
| yncJ | 1,610189242 | 0,009781183 | |
| cusC | 1,608250857 | 0,034974339 | TRANS-RXN0-280, TRANS-RXN-90 |
| nfuA | 1,60629242 | 0,0000196 | |
| yeiP | 1,606178199 | 9,77E-08 | |
| fecE | 1,60329664 | 0,000000165 | ABC-9-RXN, RXN0-2261 |
| trmD | 1,602763891 | 0,0000088 | RXN-12458 |
| hcaB | 1,600950733 | 0,004287244 | RXN-12071, PHENPRODIOLDEHYDROG-RXN |
| rpsN | 1,599194677 | 0,0000829 | |

| | | | |
|---|---|---|---|
| purC | 1,597773811 | 0,000475188 | SAICARSYN-RXN |
| nrdI | 1,597287434 | 0,001257012 | |
| fimD | 1,597240733 | 0,0000028 | |
| rfbB | 1,596143977 | 3,68E-08 | DTDPGLUCDEHYDRAT-RXN |
| iscA | 1,592655944 | 0,00000917 | |
| rpsE | 1,592496511 | 0,0000611 | |
| rlmN | 1,589196273 | 5,37E-08 | RXN-11586, RXN0-7007 |
| fecC | 1,589028304 | 0,003509993 | ABC-9-RXN, RXN0-2261 |
| rpsP | 1,588366861 | 0,00000523 | |
| ymfM | 1,584984142 | 0,0444514 | |
| rplP | 1,578390155 | 0,0000391 | |
| thiI | 1,577279302 | 3,7E-09 | THIFIS-RXN, RXN-9788, TRNA-S-TRANSFERASE-RXN |
| higB | 1,574124182 | 0,034772539 | RXN0-6381 |
| rzpD | 1,572987705 | 0,017341801 | |
| potF | 1,572179473 | 0,000419027 | ABC-25-RXN |
| entH | 1,57007888 | 0,029366255 | RXN0-7046 |
| potG | 1,568930393 | 0,0000524 | ABC-25-RXN |
| ygcR | 1,557338326 | 0,005865611 | |
| fdx | 1,555524427 | 0,000000175 | |
| ykfF | 1,548890728 | 0,018385475 | |
| ydeJ | 1,54862658 | 0,000310645 | |
| glcC | 1,547917708 | 0,000431098 | |
| mrcA | 1,547457191 | 3,9E-09 | RXN-11302, RXN0-5405 |
| gltL | 1,546776484 | 0,00000952 | TRANS-RXN0-222, ABC-13-RXN |
| yraH | 1,543818927 | 0,012577183 | |
| ppa | 1,542825088 | 0,0000349 | INORGPYROPHOSPHAT-RXN, TRIPHOSPHATASE-RXN |
| mhpA | 1,53805597 | 0,000962232 | RXN-10040, MHPHYDROXY-RXN |
| fliR | 1,535817393 | 0,047984523 | |
| prpE | 1,534897961 | 0,000000151 | PROPIONATE--COA-LIGASE-RXN |
| rplK | 1,532677069 | 0,0000888 | |
| rsxE | 1,529039907 | 0,000519996 | |
| xthA | 1,518470442 | 0,0000235 | 3.1.11.2-RXN |
| fepB | 1,516703998 | 0,00238789 | ABC-10-RXN, RXN0-2181 |
| yibQ | 1,51420986 | 0,000156666 | NUCLEOSIDE-DIPHOSPHATASE-RXN |
| amiC_1 | 1,5135026 | 0,000000579 | RXN0-5190 |
| rplL | 1,512772397 | 0,000203634 | |
| yjhX | 1,509745114 | 0,001870048 | |
| rdgB | 1,508003131 | 0,000214243 | RXN0-6382, RXN0-1602, RXN0-1603 |
| bglJ | 1,507959254 | 0,012200159 | |
| folB | 1,50727344 | 0,019636905 | H2NEOPTERINALDOL-RXN, RXN-10856 |
| psuK | 1,506076179 | 0,001356699 | PSEUDOURIDINE-KINASE-RXN |
| udk | 1,502453818 | 0,000165697 | URIDINEKIN-RXN, CYTIKIN-RXN, URKI-RXN, CYTIDINEKIN-RXN |
| ypaA | 1,502355214 | 0,004682778 | |
| ssuE | 1,501873601 | 0,025197766 | RXN-12444 |
| yjhQ | 1,500503211 | 0,00000752 | |
| recF | 1,494270783 | 0,0000257 | RXN0-2606 |
| rplS | 1,493944585 | 0,0000142 | |
| mreC | 1,492642047 | 0,000000123 | |
| dtpA | 1,492618412 | 7,09E-08 | TRANS-RXN0-288, TRANS-RXN0-267 |

| | | | | |
|---|---|---|---|---|
| usg | 1,4924162 | 1,22E-11 | | PHEAMINOTRANS-RXN |
| ibaG | 1,483858332 | 0,014188838 | | |
| ugd | 1,483440321 | 0,00000496 | UGD-RXN | |
| mreB | 1,481240858 | 0,000000155 | | |
| ydcP | 1,48081714 | 5,27E-08 | | |
| greA | 1,479192274 | 0,000695124 | | |
| guaA | 1,477794984 | 0,000431261 | GMP-SYN-GLUT-RXN, GMP-SYN-NH3-RXN | |
| arpB | 1,477192051 | 0,039333973 | | |
| yjbE | 1,475820576 | 0,049060535 | | |
| tolR | 1,474283009 | 0,0000464 | | |
| efp | 1,469134397 | 0,00000676 | | |
| nrdA | 1,468898541 | 0,0000641 | RIBONUCLEOSIDE-DIP-REDUCTI-RXN, CDPREDUCT-RXN, UDPREDUCT-RXN, ADPREDUCT-RXN, GDPREDUCT-RXN | |
| rpmC | 1,468592362 | 0,0000975 | | |
| iscX | 1,468264817 | 0,0000818 | | |
| rplT | 1,466160472 | 0,000000836 | | |
| trmN | 1,466159709 | 0,000174604 | | |
| yqgB | 1,459397273 | 0,036474132 | | |
| cedA | 1,45656378 | 0,045278494 | | |
| pcnB | 1,450929661 | 2,06E-09 | POLYNUCLEOTIDE-ADENYLYLTRANSFERASE-RXN | |
| yffQ | 1,449250908 | 0,012921058 | | |
| rlmI | 1,449127794 | 1,98E-08 | RXN-11602 | |
| marB | 1,447183573 | 0,017015784 | | |
| prfB | 1,446198263 | 0,00000511 | | |
| pspG | 1,445990455 | 0,02155749 | | |
| sfmD | 1,444955677 | 0,034521966 | | |
| mepS | 1,44485677 | 0,00251642 | RXN0-5227, RXN0-3461 | |
| dusA | 1,441672313 | 0,000000778 | RXN0-1281 | DGDPKIN-RXN, GDPKIN-RXN |
| eamA | 1,441204846 | 0,0000193 | RXN0-1924, RXN0-1923 | |
| prc | 1,440147974 | 0,000000298 | 3.4.21.102-RXN | |
| rpsQ | 1,439877387 | 0,000353619 | | |
| bax | 1,43979765 | 0,000000696 | | |
| yjcH | 1,439083882 | 0,048401981 | | |
| rpsM | 1,438762412 | 0,0000266 | | |
| rplA | 1,434965453 | 0,000336225 | | |
| cyoC | 1,434649686 | 0,001533445 | RXN0-5268 | |
| ppiC | 1,434378798 | 0,0000413 | PEPTIDYLPROLYL-ISOMERASE-RXN | |
| yfaP | 1,434261116 | 0,012171873 | | |
| prmC | 1,433965852 | 0,00000307 | RXN-14992 | |
| csgG | 1,430936509 | 0,001079537 | RXN-5068 | |
| tsf | 1,430182807 | 0,000226609 | | |
| rplR | 1,426167146 | 0,0000888 | | |
| hydN | 1,425075474 | 0,047503155 | | |
| lolD | 1,424599127 | 0,0000105 | | TRANS-RXN-108C, TRANS-RXN-108B, TRANS-RXN-108A |
| yciA | 1,423950775 | 0,03563742 | ACYL-COA-HYDROLASE-RXN | |
| guaC | 1,422248567 | 0,00000424 | GMP-REDUCT-RXN | |
| cysS | 1,421773634 | 8,66E-09 | CYSTEINE--TRNA-LIGASE-RXN | |
| rpmD | 1,42142253 | 0,000054 | | |
| proS | 1,420121732 | 0,000000858 | PROLINE--TRNA-LIGASE-RXN | |
| mnmA | 1,420019939 | 0,000000806 | RXN0-2023 | |

| | | | |
|---|---|---|---|
| nupC | 1,419841792 | 0,0000104 | TRANS-RXN-108E, TRANS-RXN-108G, TRANS-RXN-108I, TRANS-RXN-108H, TRANS-RXN-108F, TRANS-RXN-108D, |
| gyrA | 1,419646061 | 0,00000389 | 5.99.1.3-RXN |
| yahA | 1,417816007 | 0,0000221 | RXN0-4181 |
| tff | 1,412726029 | 0,0000402 | |
| marA | 1,409260228 | 0,000288639 | |
| metG | 1,407708196 | 0,000000019 | METHIONINE--TRNA-LIGASE-RXN |
| corA | 1,40753902 | 0,0000518 | TRANS-RXN-141B, TRANS-RXN-141A, TRANS-RXN-141 |
| folE | 1,405257055 | 0,00000449 | GTP-CYCLOHYDRO-I-RXN |
| ssuA | 1,404066701 | 0,034266844 | ABC-56-RXN |
| yjjK | 1,404038269 | 0,00000245 | |
| sfsB | 1,403838708 | 0,001384281 | |
| proQ | 1,403421212 | 0,000137605 | TRANS-RXN0-283, RXN0-1565, TRANS-RXN0-455, RXN0-2121 |
| rsxA | 1,400887499 | 0,001032919 | |
| rpmJ | 1,399748875 | 0,0000224 | |
| rpsB | 1,398021211 | 0,000264501 | |
| yobF | 1,392148786 | 0,000147528 | |
| mgtA | 1,392017629 | 0,00000546 | TRANS-RXN-250, ABC-20-RXN |
| agaC | 1,391850494 | 0,033341607 | |
| ybiT | 1,38701826 | 1,24E-09 | |
| insZ | 1,380299515 | 0,008820548 | |
| gfcC | 1,377916167 | 0,016835902 | |
| dppB | 1,377718376 | 0,003380426 | ABC-8-RXN |
| yciW | 1,377603485 | 0,004082306 | |
| yafF | 1,377319052 | 0,022983764 | |
| yedF | 1,377305373 | 0,004754183 | |
| gpt | 1,377212756 | 0,001301249 | XANPRIBOSYLTRAN-RXN, GUANPRIBOSYLTRAN-RXN, HYPOXANPRIBOSYLTRAN-RXN |
| yccA | 1,37652501 | 0,0000168 | |
| ykfI | 1,373960949 | 0,032527651 | |
| dsbA | 1,368595805 | 0,00000105 | DISULFOXRED-RXN |
| oxc | 1,367367484 | 0,008993611 | OXALYL-COA-DECARBOXYLASE-RXN |
| glpR | 1,363591723 | 0,0000117 | |
| wcaC | 1,362321036 | 0,045478231 | |
| trmL | 1,360969013 | 0,000332887 | RXN-11865, RXN-11860 |
| yibN | 1,357946904 | 0,000038 | |
| wcaL | 1,356818283 | 0,009183137 | |
| gcvT | 1,352084964 | 0,0000371 | GCVT-RXN, GCVMULTI-RXN |
| csgD | 1,351267945 | 0,031058815 | |
| fkpA | 1,351184189 | 0,0000674 | PEPTIDYLPROLYL-ISOMERASE-RXN |
| ybbY | 1,349893056 | 0,005890111 | |
| hsdM | 1,348764737 | 0,00000115 | 3.1.21.3-RXN |
| glnQ | 1,343156371 | 0,000284719 | ABC-12-RXN |
| prpC | 1,34147533 | 0,001119536 | 2-METHYLCITRATE-SYNTHASE-RXN, CITSYN-RXN |
| metR | 1,340761364 | 0,029072905 | |
| argS | 1,340425091 | 3,08E-09 | ARGININE--TRNA-LIGASE-RXN |
| phoH | 1,340340425 | 0,006101001 | |
| aroM | 1,340213576 | 0,001598713 | |
| glsB | 1,337849985 | 0,000923625 | |
| yedV | 1,337448355 | 0,0002687 | |
| sdhB | 1,336043494 | 0,009230014 | SUCCINATE-DEHYDROGENASE-UBIQUINONE-RXN |

| | | | | |
|---|---|---|---|---|
| rpsO | 1,335838066 | 0,000023 | | |
| yfaZ | 1,335601322 | 0,001685839 | | |
| miaB | 1,335250261 | 3,08E-09 | RXN0-5063 | |
| aroA | 1,333554007 | 0,000464446 | 2.5.1.19-RXN | |
| rpmI | 1,331609411 | 0,00000433 | | |
| ygiM | 1,331279827 | 0,013187672 | | |
| fabA | 1,330756438 | 0,0000214 | RXN-9655, 3-HYDROXYDECANOYL-ACP-DEHYDR-RXN, 5.3.3.14-RXN | |
| rpmF | 1,32643594 | 0,000000831 | | |
| yheS | 1,326137055 | 0,000000379 | | |
| gltK | 1,319917243 | 0,000148043 | TRANS-RXN0-222, ABC-13-RXN | |
| rfbA | 1,317802448 | 4,88E-08 | DTDPGLUCOSEPP-RXN | |
| tolQ | 1,317154047 | 0,000279355 | | |
| pth | 1,316732624 | 0,001816057 | AMINOCYL-TRNA-HYDROLASE-RXN | |
| ispE | 1,309886193 | 1,67E-08 | 2.7.1.148-RXN | |
| rimP | 1,30938011 | 0,00000753 | | |
| rplO | 1,308443834 | 0,000373842 | | |
| prmB | 1,30756311 | 0,00000424 | RXN0-1241 | |
| lacI | 1,306325223 | 0,000476173 | | |
| casB | 1,299997558 | 0,043620458 | RXN0-5435 | |
| ubiG | 1,296424498 | 0,000143927 | 2-OCTAPRENYL-6-OHPHENOL-METHY-RXN, DHHB-METHYLTRANSFER-RXN | |
| yicG | 1,296026105 | 0,001283557 | | |
| potB | 1,293845268 | 0,0000788 | ABC-25-RXN, ABC-24-RXN | |
| ydiE | 1,290794496 | 0,004658298 | | |
| metB | 1,289952707 | 0,0000296 | O-SUCCHOMOSERLYASE-RXN, METBALT-RXN | |
| rlmL | 1,289818185 | 0,00000014 | RXN0-6950, RXN-11574 | |
| ispF | 1,289607966 | 0,00000105 | RXN0-302 | |
| yqcC | 1,287199255 | 0,022416725 | | |
| metN | 1,286868395 | 0,038595679 | TRANS-RXN0-511, TRANS-RXN0-510, RXN0-4522, TRANS-RXN0-202 | |
| amtB | 1,285806242 | 0,000891718 | TRANS-RXN0-206 | |
| nuoB | 1,285458173 | 0,0000184 | NADH-DEHYDROG-A-RXN, RXN0-5388 | |
| pldA | 1,279839382 | 0,000106232 | RXN0-6725, RXN0-6952 | |
| map | 1,279538726 | 0,00000159 | 3.4.11.18-RXN | ENOYL-COA-DELTA-ISOM-RXN, RXN0-5391 |
| wecC | 1,274729127 | 0,0000017 | | |
| yegQ | 1,274611714 | 0,000513373 | | |
| lysA | 1,271717544 | 0,0000581 | DIAMINOPIMDECARB-RXN | |
| argF | 1,269339632 | 0,013483645 | ORNCARBAMTRANSFER-RXN | |
| allD | 1,267137421 | 0,036717246 | RXN0-7024 | |
| yeaL | 1,266606895 | 0,019636905 | | |
| btuE | 1,265001022 | 0,000218208 | GLUTATHIONE-PEROXIDASE-RXN, RXN0-267 | |
| yccF | 1,264624408 | 0,004837255 | | |
| uraA | 1,262505191 | 0,022860252 | TRANS-RXN-132 | |
| hscA | 1,261814218 | 0,0000166 | RXN0-1061 | |
| lepB | 1,261340794 | 0,000000022 | 3.4.21.89-RXN | |
| nuoC | 1,259230101 | 0,0000349 | NADH-DEHYDROG-A-RXN, RXN0-5388 | |
| potC | 1,256459976 | 0,0000666 | ABC-25-RXN, ABC-24-RXN | |
| coaA | 1,255716408 | 0,003403489 | PANTOTHENATE-KIN-RXN | |
| yjhP | 1,253893204 | 0,001111155 | | |
| ycbK | 1,253260737 | 0,001101944 | | |
| yhbE | 1,252815951 | 0,000289497 | | |

| | | | |
|---|---|---|---|
| recQ | 1,251725503 | 0,001539887 | RXN0-4261 |
| lepA | 1,248303059 | 0,00000198 | |
| hisM | 1,244525731 | 0,014946756 | ABC-37-RXN, ABC-4-RXN, ABC-3-RXN, ABC-14-RXN |
| tgt | 1,243321295 | 0,00000304 | RXN0-1321 |
| speD | 1,242204117 | 0,0000055 | SAMDECARB-RXN |
| acs | 1,241275349 | 0,046264842 | ACETATE--COA-LIGASE-RXN, PROPIONATE--COA-LIGASE-RXN |
| rph | 1,239561608 | 0,003486702 | RXN0-6482, RXN0-6481, TRNA-NUCLEOTIDYLTRANSFERASE-RXN |
| ydiH | 1,239297456 | 0,039607449 | |
| cybB | 1,238751296 | 0,000077 | |
| yifE | 1,236617389 | 0,000000159 | |
| rfbC | 1,235597742 | 0,0000251 | DTDPDEHYDRHAMEPIM-RXN, DTDPRHAMSYNTHMULTI-RXN |
| yjeO | 1,235589288 | 0,036214599 | |
| wzzB | 1,235307567 | 0,000984711 | |
| rplY | 1,232852372 | 0,004947792 | |
| avtA | 1,231670452 | 0,0000374 | RXN0-5200, VALINE-PYRUVATE-AMINOTRANSFER-RXN |
| truC | 1,230268266 | 0,000756183 | RXN-11840 |
| potH | 1,229942856 | 0,036475901 | ABC-25-RXN |
| fecB | 1,229904685 | 0,013968453 | ABC-9-RXN, RXN0-2261 |
| potA | 1,229740977 | 0,000000889 | ABC-25-RXN, ABC-24-RXN |
| thrA | 1,229704015 | 0,000972839 | ASPARTATEKIN-RXN, HOMOSERDEHYDROG-RXN |
| ybiR | 1,229380794 | 0,0000368 | |
| wcaJ | 1,228596699 | 0,043835951 | |
| pncA | 1,228015038 | 0,00000477 | NICOTINAMID-RXN, PYRAZIN-RXN |
| abgR | 1,225392881 | 0,004405516 | |
| yeaJ | 1,224365232 | 0,0000128 | |
| ybfE | 1,222891962 | 0,040088983 | |
| hcaF | 1,22240533 | 0,046661652 | RXN-12072, HCAMULTI-RXN |
| priB | 1,222077096 | 0,0000865 | |
| betT | 1,22080798 | 0,000083 | TRANS-RXN-99 |
| ybiU | 1,219860478 | 0,025448977 | |
| tufB | 1,211367901 | 0,003774893 | |
| rnt | 1,208021368 | 0,001187281 | RXN0-6484, RXN0-6483, RXN0-4223 |
| yqiJ | 1,207427238 | 0,022930789 | |
| secY | 1,207389348 | 0,00072395 | |
| pncB | 1,204599132 | 0,0000395 | NICOTINATEPRIBOSYLTRANS-RXN |
| arpA | 1,203310971 | 0,009352946 | |
| dusB | 1,203274502 | 0,002868754 | RXN0-1281 |
| yddB | 1,202847999 | 0,006015805 | |
| yiaD | 1,201953213 | 0,004263132 | |
| nusA | 1,201715669 | 0,00000105 | |
| coaD | 1,19901503 | 0,000188169 | PANTEPADENYLYLTRAN-RXN |
| wecB | 1,198809704 | 0,000119915 | |
| msbA | 1,198512839 | 0,000000268 | TRANS-RXN-236, 3.6.3.39-RXN |
| livM | 1,196359813 | 0,033183198 | ABC-35-RXN, ABC-36-RXN, ABC-15-RXN |
| slmA | 1,194880704 | 0,001030108 | |
| prfA | 1,186537585 | 0,0000203 | |
| rplI | 1,186101656 | 0,000404567 | |
| dut | 1,184808748 | 0,00000787 | DUTP-PYROP-RXN |
| maeA | 1,182785832 | 0,000056 | 1.1.1.39-RXN |

| | | | |
|---|---|---|---|
| grpE | 1,181790571 | 0,003163243 | RXN-11478, RXN-9657, RXN-9658, RXN-9659, RXN-9660, RXN-9661, RXN-9662, RXN-9663 |
| tatE | 1,174534965 | 0,0000534 | TRANS-RXN0-181 |
| tdk | 1,173080278 | 0,027040814 | THYKI-RXN, DURIDKI-RXN |
| ygiS | 1,171539889 | 0,000361785 | |
| yebY | 1,171382939 | 0,000166207 | |
| amiA | 1,171253891 | 0,045006859 | 3.5.1.28-RXN |
| yecF | 1,16755695 | 0,02990695 | |
| nudE | 1,166511159 | 0,009487543 | ADPSUGPPHOSPHAT-RXN |
| citT | 1,163450781 | 0,044458346 | TRANS-RXN0-201 |
| dxs | 1,161246366 | 0,00000271 | DXS-RXN |
| accD | 1,160981791 | 0,000382125 | RXN0-5055, ACETYL-COA-CARBOXYLTRANSFER-RXN |
| parE | 1,160824643 | 0,000000544 | |
| mepA | 1,159255821 | 0,0000305 | RXN0-5407, RXN0-3461 |
| ydgC | 1,14987488 | 0,018438781 | |
| rnhB | 1,149359849 | 0,000110594 | 3.1.26.4-RXN |
| yojI | 1,149208637 | 0,014527071 | |
| yqgC | 1,148236964 | 0,032805301 | |
| yfaE | 1,14611985 | 0,006010593 | |
| rnd | 1,144919097 | 0,00099763 | RXN0-6484, RXN0-6483, 3.1.13.5-RXN |
| ddlA | 1,14478008 | 0,000017 | DALADALALIG-RXN |
| rpsF | 1,14402403 | 0,000546802 | |
| hslU | 1,1432716 | 0,000355954 | |
| speC | 1,141239118 | 0,0000522 | ORNDECARBOX-RXN |
| exbB | 1,140377179 | 0,032658501 | RXN0-1702, TRANS-RXN0-488, RXN0-1684, RXN0-2261, RXN0-1701, RXN0-2241, RXN0-1682, RXN0-2181, |
| csiD | 1,139899625 | 0,004418411 | |
| yqgF | 1,139270296 | 0,001241648 | |
| deaD | 1,137813681 | 0,000197937 | |
| yaiY | 1,137178238 | 0,034792065 | |
| alaS | 1,136037574 | 0,000793602 | ALANINE--TRNA-LIGASE-RXN |
| psrD | 1,135199218 | 0,005283355 | |
| ynbE | 1,134714392 | 0,029741729 | |
| aroC | 1,133579895 | 0,000013 | CHORISMATE-SYNTHASE-RXN |
| ygfF | 1,128484596 | 0,0000051 | |
| mnmG | 1,126604308 | 0,0000011 | RXN0-7068, RXN0-7082, RXN0-7083 |
| rlmA | 1,122206303 | 0,00578868 | RXN-11573 |
| trkG | 1,116841248 | 0,012204679 | TRANS-RXN-3 |
| thyA | 1,115758324 | 0,00000747 | THYMIDYLATESYN-RXN |
| mltD | 1,111864221 | 0,0000386 | RXN0-5190 |
| gdhA | 1,110673256 | 0,0000995 | GLUTDEHYD-RXN, GLUTAMATESYN-RXN |
| pncC | 1,10915263 | 0,005095929 | NMNAMIDOHYDRO-RXN |
| nuoA | 1,108714311 | 0,000107933 | NADH-DEHYDROG-A-RXN, RXN0-5388 |
| gspE | 1,108276452 | 0,036924122 | |
| rplJ | 1,106390096 | 0,004417036 | |
| leuA | 1,106364319 | 0,002478787 | 2-ISOPROPYLMALATESYN-RXN |
| betA | 1,10453715 | 0,002353595 | CHD-RXN |
| dedA | 1,104189682 | 0,000439244 | |
| ycbL | 1,100865011 | 0,000189897 | |
| pyrH | 1,100809483 | 0,0000179 | RXN-12002 |
| dnaX | 1,09986873 | 0,000510097 | |

| | | | |
|---|---|---|---|
| topA | 1,096518769 | 0,000109401 | 5.99.1.2-RXN |
| dapD | 1,095238002 | 0,000591783 | TETHYDPICSUCC-RXN |
| gtrS | 1,093838372 | 0,044651764 | |
| srkA | 1,092253285 | 0,000927029 | RXN-14393, OHBUTYRYL-COA-EPIM-RXN, OHACYL-COA-DEHYDROG-RXN, ENOYL-COA-HYDRAT-RXN, RXN0-5393, |
| dacB | 1,090073412 | 0,001078798 | RXN0-3461 |
| acrA | 1,088585336 | 0,00000496 | TRANS-RXN-92, TRANS-RXN0-592 |
| parC | 1,084598405 | 0,000000436 | |
| yhaM | 1,084588651 | 0,004596869 | |
| sbcD | 1,084449052 | 0,000693992 | |
| obgE | 1,081072133 | 0,0000804 | |
| rbn | 1,079120521 | 0,005076467 | RXN0-6525, RXN0-4222 |
| nuoE | 1,076520054 | 0,00064995 | NADH-DEHYDROG-A-RXN, RXN0-5388 |
| atpI | 1,075247043 | 0,003053415 | |
| fadK | 1,075241789 | 0,024304262 | ACYLCOASYN-RXN |
| ispD | 1,074363646 | 0,003450843 | 2.7.7.60-RXN |
| trxC | 1,067821801 | 0,001060442 | |
| cysM | 1,064926301 | 0,004469334 | ACSERLY-RXN, RXN-15129, LCYSDESULF-RXN |
| ispA | 1,064622013 | 0,004615045 | GPPSYN-RXN, FPPSYN-RXN |
| lolC | 1,064493891 | 0,004060205 | |
| fusA | 1,062364611 | 0,005919033 | |
| flk | 1,061653458 | 0,002347254 | |
| glnS | 1,060239769 | 0,000431261 | GLUTAMINE--TRNA-LIGASE-RXN |
| holD | 1,059244693 | 0,021969714 | |
| mrdB | 1,057042189 | 0,000035 | |
| glyQ | 1,055103533 | 0,00012374 | GLYCINE--TRNA-LIGASE-RXN |
| folD | 1,053188936 | 0,0000545 | METHENYLTHFCYCLOHYDRO-RXN, METHYLENETHFDEHYDROG-NADP-RXN |
| dppC | 1,047518091 | 0,007197848 | ABC-8-RXN |
| crl | 1,046875352 | 0,007758478 | |
| eptC | 1,046139201 | 0,000241832 | RXN-14363 |
| yeiB | 1,04586242 | 0,007983055 | |
| rlmH | 1,044337154 | 0,003947589 | RXN-11592 |
| yliE | 1,043490011 | 0,020597631 | |
| tsaC | 1,042390995 | 0,000193031 | |
| yeaX | 1,04165789 | 0,027815433 | |
| birA | 1,036354615 | 0,000203634 | BIOTINLIG-RXN |
| der | 1,035343331 | 0,00000149 | RXN0-5462 |
| yfgM | 1,03391041 | 0,00000377 | |
| yhcM | 1,033221055 | 0,000203634 | |
| ydeE | 1,026008655 | 0,00410476 | |
| chaA | 1,025393869 | 0,002175793 | TRANS-RXN-42, TRANS-RXN-101 |
| pal | 1,025035944 | 0,000953322 | ENOYL-COA-DELTA-ISOM-RXN, RXN0-5391 |
| menA | 1,024073328 | 0,024091894 | DMK-RXN |
| grxD | 1,02270956 | 0,000511402 | |
| cspC | 1,022674665 | 0,001518205 | |
| tyrB | 1,021558085 | 0,000824556 | 2.6.1.57-RXN, BRANCHED-CHAINAMINOTRANSFERLEU-RXN, TYROSINE-AMINOTRANSFERASE-RXN, |
| ybhA | 1,021509195 | 0,00064995 | 3.1.3.74-RXN, F16BDEPHOS-RXN |
| yqiK | 1,021030125 | 0,030911264 | |
| cmoB | 1,020970938 | 0,000279355 | RXN0-7067 |
| ugpC | 1,020322936 | 0,004240402 | TRANS-RXN0-573, ABC-34-RXN |

| | | | |
|---|---|---|---|
| cynR | 1,017763318 | 0,005028157 | |
| cheA | 1,016496171 | 0,035514344 | |
| yajC | 1,014471313 | 0,000106232 | |
| ydiJ | 1,014065246 | 0,000131022 | |
| thiB | 1,012154729 | 0,009272138 | ABC-32-RXN |
| yqgE | 1,010780601 | 0,00039675 | |
| serC | 1,008909322 | 0,000356027 | PSERTRANSAM-RXN, PSERTRANSAMPYR-RXN |
| pdxY | 1,008377804 | 0,0000756 | PYRIDOXKIN-RXN |
| epmB | 1,00633702 | 0,001465823 | RXN0-5192 |
| gltJ | 1,003849521 | 0,021124686 | TRANS-RXN0-222, ABC-13-RXN |
| fepG | 1,003467622 | 0,047067515 | ABC-10-RXN, RXN0-2181 |
| pheS | 1,003434677 | 0,000147484 | PHENYLALANINE--TRNA-LIGASE-RXN |
| mipA | 1,002791614 | 0,012591062 | |
| oppC | 1,002261525 | 0,002417868 | TRANS-RXN0-268, ABC-22-RXN |
| rpsR | 1,001890837 | 0,001863049 | |
| lolB | 1,000535253 | 0,003947589 | |
| rlmC | 0,99974942 | 0,006521042 | RXN-11600 |
| iadA | 0,998158387 | 0,000361785 | RXN0-3241 |
| tolB | 0,998009857 | 0,000118256 | |
| pitA | 0,997843392 | 0,00000593 | TRANS-RXN0-550, TRANS-RXN-114 |
| bcp | 0,997031453 | 0,002983507 | RXN0-5468 |
| rpe | 0,995152263 | 0,00000772 | RIBULP3EPIM-RXN |
| amiC | 0,991262275 | 0,002996873 | 3.5.1.28-RXN |
| gph | 0,990026862 | 0,000119413 | GPH-RXN |
| uup | 0,989949448 | 0,00000952 | |
| serB | 0,988416378 | 0,000303648 | RXN0-5114 |
| ilvH | 0,988140229 | 0,047493593 | ACETOLACTSYN-RXN, ACETOOHBUTSYN-RXN |
| murJ | 0,988114224 | 0,000546802 | TRANS-RXN0-286 |
| rluB | 0,988029623 | 0,0000145 | RXN-11836 |
| nfsB | 0,985387286 | 0,0000657 | RXN-13853, 1.5.1.34-RXN |
| pdxH | 0,984402842 | 0,00281669 | PNPOXI-RXN, PMPOXI-RXN |
| rffG | 0,982654229 | 0,0000132 | DTDPGLUCDEHYDRAT-RXN |
| yigB | 0,982568756 | 0,006687709 | RIBOPHOSPHAT-RXN, RXN0-5187 |
| ycgJ | 0,982350243 | 0,001701468 | |
| yfaD | 0,980371837 | 0,001931793 | |
| accA | 0,979682878 | 0,000431193 | RXN0-5055, ACETYL-COA-CARBOXYLTRANSFER-RXN |
| nusG | 0,978919901 | 0,000225462 | |
| rnk | 0,978528987 | 0,000341827 | |
| fadI | 0,976945696 | 0,016471348 | KETOACYLCOATHIOL-RXN |
| secF | 0,972739612 | 0,0000172 | |
| secD | 0,969992719 | 0,000128799 | |
| yejK | 0,965873499 | 0,000197267 | |
| sseB | 0,964705619 | 0,00150032 | |
| lptE | 0,964420123 | 0,0000198 | TRANS-RXN0-531, TRANS-RXN-237 |
| lhgO | 0,963893705 | 0,011465222 | RXN0-5364 |
| trpS | 0,963220808 | 0,0000604 | TRYPTOPHAN--TRNA-LIGASE-RXN |
| atpD | 0,962611356 | 0,044458346 | TRANS-RXN-249, RXN0-7041 |
| ispH | 0,95696686 | 0,0000384 | RXN0-884, ISPH2-RXN |
| thiQ | 0,956415257 | 0,025893291 | ABC-32-RXN |

| | | | |
|---|---|---|---|
| yigM | 0,955304437 | 0,016198609 | |
| msrC | 0,954502583 | 0,004486371 | 1.8.4.14-RXN |
| rdgC | 0,954487721 | 0,003487297 | |
| aspS | 0,953762419 | 0,000191705 | ASPARTATE--TRNA-LIGASE-RXN |
| yjiR | 0,950713679 | 0,001987969 | |
| yhjV | 0,946999218 | 0,009934401 | |
| borD | 0,945711959 | 0,024970319 | |
| rpmE | 0,941855766 | 0,003081693 | |
| yeeN | 0,940506212 | 0,015064358 | |
| slyD | 0,940146131 | 0,00049819 | PEPTIDYLPROLYL-ISOMERASE-RXN |
| yehW | 0,939175533 | 0,007242428 | |
| nudJ | 0,934589691 | 0,004062835 | GUANOSINE-DIPHOSPHATASE-RXN, RXN0-3543, RXN0-3542 |
| dfp | 0,934081389 | 0,000348862 | P-PANTOCYSLIG-RXN, P-PANTOCYSDECARB-RXN |
| fur | 0,933089364 | 0,003290886 | |
| casE | 0,931223342 | 0,029400791 | RXN0-5435 |
| fecD | 0,930532084 | 0,018697002 | ABC-9-RXN, RXN0-2261 |
| fnr | 0,927220139 | 0,000646712 | |
| fabZ | 0,926620995 | 0,000106637 | RXN-11477, RXN-11481, RXN-10656, RXN-10660, RXN0-2144, 3-HYDROXYDECANOYL-ACP-DEHYDR-RXN |
| ompX | 0,926292637 | 0,007852512 | |
| waaG | 0,924720083 | 0,000127652 | RXN0-5120 |
| erpA | 0,923956212 | 0,002117471 | |
| dnaB | 0,92358539 | 0,000140763 | RXN0-4261 |
| yfiP | 0,922650509 | 0,03355886 | |
| lon | 0,922222319 | 0,004287244 | 3.4.21.53-RXN |
| yhbW | 0,91973053 | 0,001496038 | |
| bcsA | 0,919116643 | 0,019992945 | |
| yajG | 0,91857369 | 0,043387503 | |
| yhaL | 0,918111984 | 0,010029401 | |
| rpoZ | 0,917639383 | 0,0000459 | |
| nfi | 0,917467822 | 0,000185259 | 3.1.21.7-RXN |
| ychE | 0,917127826 | 0,007758478 | |
| yjeJ | 0,916647859 | 0,013294601 | |
| yjhB | 0,910969897 | 0,048759617 | |
| xerC | 0,907821825 | 0,005209037 | |
| yacG | 0,905867522 | 0,010209742 | |
| rcnB | 0,90552959 | 0,008360732 | |
| fkpB | 0,905468085 | 0,000894618 | PEPTIDYLPROLYL-ISOMERASE-RXN |
| waaC | 0,901663392 | 0,000421158 | RXN0-5118, RXN0-5057 |
| hemB | 0,90131062 | 0,0000684 | PORPHOBILSYNTH-RXN |
| yhgN | 0,900542774 | 0,021841635 | |
| speE | 0,899862091 | 0,000143112 | SPERMIDINESYN-RXN, RXN0-5217 |
| mltB | 0,899274276 | 0,011804024 | RXN0-5190 |
| hisQ | 0,888301632 | 0,036214599 | ABC-37-RXN, ABC-4-RXN, ABC-3-RXN, ABC-14-RXN |
| yedD | 0,887002905 | 0,000559146 | |
| yadS | 0,880837682 | 0,011607606 | |
| dusC | 0,880445 | 0,023653015 | RXN0-1281 |
| yghE | 0,879732109 | 0,005577928 | ORNDECARBOX-RXN |
| ligA | 0,876209858 | 0,0000428 | DNA-LIGASE-NAD+-RXN |
| yicC | 0,874832422 | 0,001027137 | |

| | | | |
|---|---|---|---|
| rsmG | 0,873789445 | 0,010404929 | RXN-11578 |
| aer | 0,873785304 | 0,011507407 | |
| rluC | 0,872107817 | 0,002582622 | RXN-11838 |
| rpoB | 0,87197106 | 0,017909482 | DNA-DIRECTED-RNA-POLYMERASE-RXN |
| yidK | 0,870312391 | 0,025893291 | |
| purU | 0,865078326 | 0,003785997 | FORMYLTHFDEFORMYL-RXN |
| yajR | 0,864335127 | 0,006644061 | |
| mioC | 0,863162053 | 0,01427088 | |
| infB | 0,861207344 | 0,001585804 | |
| nuoF | 0,857414666 | 0,029361711 | NADH-DEHYDROG-A-RXN, RXN0-5388 |
| ycbX | 0,856561933 | 0,001768154 | |
| nhaB | 0,855109124 | 0,000523001 | TRANS-RXN-130 |
| asnS | 0,855044376 | 0,000523001 | ASPARAGINE--TRNA-LIGASE-RXN |
| ldtB | 0,854805239 | 0,003432827 | |
| yebZ | 0,852525141 | 0,006625906 | |
| fabI | 0,851998947 | 0,003119626 | 1.3.1.9-RXN, RXN-9558, ENOYL-ACP-REDUCT-NADH-RXN, RXN0-2145, RXN-10661, RXN-10657, RXN-11482, |
| pheP | 0,851929055 | 0,002262845 | TRANS-RXN-56 |
| wbbJ | 0,851577589 | 0,026387832 | |
| ytfK | 0,850364838 | 0,000691233 | |
| feoB | 0,849716499 | 0,048132318 | TRANS-RXN-8 |
| yafX | 0,849228875 | 0,012944512 | |
| ppiD | 0,84730265 | 0,001921304 | |
| nadD | 0,846957127 | 0,004067048 | NICONUCADENYLYLTRAN-RXN |
| dbpA | 0,846885623 | 0,00133924 | RXN-11109 |
| aroK | 0,844113163 | 0,002953955 | SHIKIMATE-KINASE-RXN |
| galP | 0,843778214 | 0,012665597 | RXN0-7077, TRANS-RXN-21 |
| mutS | 0,841587523 | 0,00236513 | RXN0-2625 |
| ybhB | 0,838092662 | 0,004009937 | |
| ispU | 0,837058535 | 0,001836394 | RXN-8999 |
| pnp | 0,83704128 | 0,001762129 | RXN0-6479, 2.7.7.8-RXN |
| oppB | 0,83286875 | 0,005164173 | TRANS-RXN0-268, ABC-22-RXN |
| htpX | 0,831510509 | 0,001066082 | |
| ascG | 0,83148578 | 0,012559577 | |
| glnP | 0,829904088 | 0,046759273 | ABC-12-RXN |
| dam | 0,829837743 | 0,000919633 | 2.1.1.72-RXN |
| ybgF | 0,828058834 | 0,001279439 | |
| rsmB | 0,827222172 | 0,00134875 | RXN-11591 |
| epmC | 0,822671373 | 0,019636905 | RXN0-7000 |
| cysK | 0,821323847 | 0,008585024 | ACSERLY-RXN, RXN-15129, LCYSDESULF-RXN |
| yeaY | 0,816226295 | 0,004557827 | |
| dcm | 0,815702062 | 0,004947792 | DNA-CYTOSINE-5--METHYLTRANSFERASE-RXN |
| pgm | 0,815601523 | 0,003179428 | PHOSPHOGLUCMUT-RXN |
| cyoD | 0,814309017 | 0,048404807 | RXN0-5268 |
| glrR | 0,813965684 | 0,014351889 | |
| slyX | 0,813712486 | 0,00908215 | |
| fadE | 0,813681058 | 0,036341897 | ACYLCOADEHYDROG-RXN |
| lpxA | 0,812171477 | 0,000196403 | UDPNACETYLGLUCOSAMACYLTRANS-RXN |
| mtn | 0,811759738 | 0,000245446 | ADENOSYLHOMOCYSTEINE-NUCLEOSIDASE-RXN, METHYLTHIOADENOSINE-NUCLEOSIDASE-RXN, RXN0-6550 |
| dnaE | 0,81155579 | 0,004417036 | DNA-DIRECTED-DNA-POLYMERASE-RXN |

| | | | |
|---|---|---|---|
| mlaD | 0,811293625 | 0,000145774 | |
| lptA | 0,806815282 | 0,000131942 | TRANS-RXN-237 |
| gapC | 0,806238449 | 0,01818055 | |
| pdxB | 0,806171453 | 0,00020799 | ERYTHRON4PDEHYDROG-RXN |
| ryfA | 0,804651973 | 0,021522237 | |
| hslO | 0,804515484 | 0,0001834 | |
| trmH | 0,801436563 | 0,01105036 | 2.1.1.34-RXN |
| yfgD | 0,79660079 | 0,006329411 | |
| aroE | 0,795619733 | 0,002191284 | SHIKIMATE-5-DEHYDROGENASE-RXN |
| fadH | 0,794452126 | 0,035732714 | DIENOYLCOAREDUCT-RXN |
| tcdA | 0,793000949 | 0,00908215 | |
| dnaG | 0,791640332 | 0,000927029 | RXN0-5021 |
| lpxH | 0,789834167 | 0,03851419 | LIPIDXSYNTHESIS-RXN |
| lpxM | 0,788390719 | 0,000465152 | MYRPALMTRAN-RXN, MYRISTOYLACYLTRAN-RXN |
| bamB | 0,787696926 | 0,001964681 | |
| hflC | 0,786157497 | 0,00163519 | RXN0-3221 |
| serA | 0,783907128 | 0,010734329 | PGLYCDEHYDROG-RXN, RXN-8645 |
| lpd | 0,781988982 | 0,046851983 | 1.8.1.4-RXN, RXN-8629, GCVMULTI-RXN, RXN0-1132, PYRUVDEH-RXN, RXN-7716, 2OXOGLUTARATEDEH-RXN |
| infC | 0,781526548 | 0,004210445 | |
| ychJ | 0,77591027 | 0,021709673 | |
| glyS | 0,775219217 | 0,006869619 | GLYCINE--TRNA-LIGASE-RXN |
| yajQ | 0,772739237 | 0,000614419 | |
| mntH | 0,768528729 | 0,036268822 | RXN0-2421, TRANS-RXN-241 |
| mrdA | 0,765707579 | 0,020881644 | RXN-11302 |
| yqhA | 0,765514632 | 0,026173682 | |
| dsbD | 0,762925194 | 0,007884306 | |
| recG | 0,761071046 | 0,001052583 | RXN0-2604 |
| kdsA | 0,759841726 | 0,002300446 | KDO-8PSYNTH-RXN |
| prmA | 0,758973088 | 0,004754183 | RXN0-5419 |
| yjjY | 0,758433221 | 0,003169172 | |
| hslV | 0,758317606 | 0,036507184 | |
| dapB | 0,758080316 | 0,002007203 | RXN-14014 |
| aroB | 0,754033614 | 0,00081263 | 3-DEHYDROQUINATE-SYNTHASE-RXN |
| minE | 0,751869701 | 0,006344013 | |
| intS | 0,751824556 | 0,023252376 | |
| acrB | 0,749742131 | 0,004062835 | TRANS-RXN-92, TRANS-RXN0-592 |
| nudB | 0,748836192 | 0,001294596 | H2NEOPTERINP3PYROPHOSPHOHYDRO-RXN, RXN0-384 |
| spoT | 0,748802766 | 0,00727239 | RXN0-6427, GDPPYPHOSKIN-RXN, PPGPPSYN-RXN |
| nrdB | 0,748764372 | 0,00908215 | RIBONUCLEOSIDE-DIP-REDUCTI-RXN, CDPREDUCT-RXN, UDPREDUCT-RXN, ADPREDUCT-RXN, GDPREDUCT-RXN |
| yebC | 0,748394492 | 0,003383844 | |
| ppiB | 0,744675248 | 0,011875357 | PEPTIDYLPROLYL-ISOMERASE-RXN |
| yigA | 0,740888335 | 0,003871641 | |
| mreD | 0,739211986 | 0,007856398 | |
| tatD | 0,738557059 | 0,043674665 | |
| ydgH | 0,736710429 | 0,01105036 | |
| emrA | 0,730015264 | 0,041189813 | TRANS-RXN-92 |
| ybbO | 0,727353567 | 0,006697006 | |
| ribF | 0,727179307 | 0,008945052 | RIBOFLAVINKIN-RXN, FADSYN-RXN |
| recR | 0,725057417 | 0,008270118 | RXN0-2606 |

| | | | |
|---|---|---|---|
| yfeY | 0,72432343 | 0,026851743 | |
| valS | 0,723330425 | 0,011205619 | VALINE--TRNA-LIGASE-RXN |
| tyrS | 0,718694516 | 0,001931793 | TYROSINE--TRNA-LIGASE-RXN |
| yehY | 0,714805145 | 0,030254381 | |
| yggT | 0,706398051 | 0,011875357 | |
| proY | 0,704722005 | 0,028962492 | TRANS-RXN-29 |
| diaA | 0,703626552 | 0,002722741 | |
| hupB | 0,702007128 | 0,018338891 | |
| yfiB | 0,700785271 | 0,04190505 | |
| radA | 0,698455686 | 0,004263132 | |
| envC | 0,692544931 | 0,037197134 | |
| cmoA | 0,69117983 | 0,041037356 | RXN0-7066 |
| ppiA | 0,69088081 | 0,041495005 | PEPTIDYLPROLYL-ISOMERASE-RXN |
| ydcR | 0,689558706 | 0,020576811 | |
| folC | 0,68756317 | 0,000643762 | RXN0-2921, FORMYLTHFGLUSYNTH-RXN, FOLYLPOLYGLUTAMATESYNTH-RXN, DIHYDROFOLATESYNTH-RXN |
| ygeA | 0,687496463 | 0,034661785 | |
| gsk | 0,682559216 | 0,032218792 | GUANOSINEKIN-RXN, INOSINEKIN-RXN |
| lpxK | 0,682134093 | 0,003403489 | TETRAACYLDISACC4KIN-RXN |
| cdsA | 0,682085634 | 0,000528602 | CDPDIGLYSYN-RXN, RXN0-5515 |
| yecD | 0,68135205 | 0,04743625 | |
| murB | 0,680842788 | 0,00182582 | UDPNACETYLMURAMATEDEHYDROG-RXN |
| yedW | 0,680710601 | 0,015768935 | |
| waaP | 0,68069063 | 0,017537352 | RXN0-5121 |
| truA | 0,678975649 | 0,000691233 | TRNA-PSEUDOURIDINE-SYNTHASE-I-RXN |
| ycdY | 0,675726117 | 0,023484834 | |
| lptB | 0,675406792 | 0,001384281 | TRANS-RXN-237 |
| mscK | 0,674942218 | 0,001714511 | TRANS-RXN-86 |
| ileS | 0,674205743 | 0,017817698 | ISOLEUCINE--TRNA-LIGASE-RXN |
| yoaA | 0,669837097 | 0,028355316 | |
| yciU | 0,668020815 | 0,024665644 | |
| nudC | 0,663546243 | 0,04134316 | RXN0-4401 |
| yiaJ | 0,663014761 | 0,0415851 | |
| mltC | 0,66225935 | 0,048321525 | RXN0-5190 |
| yecE | 0,662183675 | 0,035854583 | |
| yhfK | 0,660906243 | 0,011443369 | |
| pabB | 0,659227926 | 0,017883134 | PABASYN-RXN |
| sbcC | 0,658187075 | 0,002900409 | |
| secE | 0,655424111 | 0,012665597 | |
| rep | 0,654779735 | 0,003596048 | RXN0-4261 |
| ubiA | 0,653494249 | 0,019636905 | 4OHBENZOATE-OCTAPRENYLTRANSFER-RXN |
| dnaN | 0,652655566 | 0,002321928 | |
| bamD | 0,6520669 | 0,029258304 | |
| yfeZ | 0,651669616 | 0,029741729 | |
| gltI | 0,647274338 | 0,041278664 | TRANS-RXN0-222, ABC-13-RXN |
| btuD | 0,647265269 | 0,027583809 | ABC-5-RXN, TRANS-RXN0-455, RXN0-2121 |
| ruvC | 0,646615317 | 0,013294601 | 3.1.22.4-RXN |
| yniC | 0,645052522 | 0,049580596 | 3.1.3.68-RXN |
| yejM | 0,645000323 | 0,001921304 | |
| rseP | 0,644378935 | 0,005971236 | |

| | | | |
|---|---|---|---|
| queC | 0,641704291 | 0,008701642 | RXN-12093 |
| yhbY | 0,641479545 | 0,027583809 | |
| rhlB | 0,631994978 | 0,006571601 | |
| yehX | 0,628243581 | 0,024332447 | |
| bamC | 0,624568722 | 0,012831522 | |
| yraP | 0,624028522 | 0,005026755 | |
| bcr | 0,623311752 | 0,023183283 | TRANS-RXN-44 |
| yadG | 0,622744881 | 0,01841034 | |
| gluQ | 0,621107857 | 0,04559577 | RXN0-5422 |
| minD | 0,61970971 | 0,030856372 | |
| rffH | 0,615101015 | 0,01079574 | DTDPGLUCOSEPP-RXN |
| cytR | 0,614106903 | 0,042796093 | |
| rlpA | 0,613840033 | 0,014359647 | |
| alsA | 0,6136032 | 0,017571661 | ABC-42-RXN |
| mfd | 0,610627642 | 0,027850569 | |
| insG | 0,610593008 | 0,035690439 | |
| recA | 0,610429741 | 0,014890399 | |
| leuS | 0,605690876 | 0,0269463 | LEUCINE--TRNA-LIGASE-RXN |
| lrp | 0,605543959 | 0,017909482 | |
| ybaB | 0,603872762 | 0,007337509 | |
| ubiC | 0,602502244 | 0,013972454 | CHORPYRLY-RXN |
| skp | 0,601277867 | 0,02385484 | |
| mscM | 0,597605038 | 0,009487543 | |
| yfeA | 0,596133119 | 0,018214918 | |
| luxS | 0,594415448 | 0,026404419 | RIBOSYLHOMOCYSTEINASE-RXN |
| yfiH | 0,592546378 | 0,012954472 | |
| fbaB_1 | 0,591280286 | 0,011834502 | INORGPYROPHOSPHAT-RXN, TRIPHOSPHATASE-RXN |
| mpl | 0,59075873 | 0,005196962 | RXN0-7022, RXN0-2361 |
| waaQ | 0,588791942 | 0,022416725 | RXN0-5122 |
| folX | 0,581327492 | 0,024709233 | H2NTPEPIM-RXN |
| galU | 0,581072645 | 0,016662483 | GLUC1PURIDYLTRANS-RXN |
| wzxB | 0,578604115 | 0,038417271 | |
| cpxP | 0,576426473 | 0,027281442 | |
| intF | 0,575819925 | 0,013760618 | |
| ttcA | 0,574550709 | 0,025803648 | |
| zapD | 0,573228001 | 0,036200138 | |
| rsmC | 0,570731538 | 0,0440755 | RXN-11576 |
| psd | 0,570310394 | 0,009697484 | PHOSPHASERDECARB-RXN |
| lgt | 0,568714146 | 0,040088983 | RXN0-20 |
| ybbN | 0,568113479 | 0,02120565 | |
| proA | 0,5673848 | 0,022800924 | GLUTSEMIALDEHYDROG-RXN, PROLINE-MULTI |
| dsbG | 0,561934097 | 0,042217858 | 5.3.4.1-RXN, DISULFOXRED-RXN |
| bamA | 0,559392993 | 0,049319604 | |
| yjjU | 0,558500395 | 0,030896383 | |
| damX | 0,555162684 | 0,023373731 | |
| trmI | 0,553178381 | 0,018315361 | TRNA-GUANINE-N7--METHYLTRANSFERASE-RXN |
| gpp | 0,551131642 | 0,007349186 | PPPGPPHYDRO-RXN |
| cysE | 0,548949191 | 0,033341607 | SERINE-O-ACETTRAN-RXN |
| dsbC | 0,544785545 | 0,015974904 | 5.3.4.1-RXN, DISULFOXRED-RXN |

| | | | |
|---|---|---|---|
| fruB | 0,544383011 | 0,021000024 | TRANS-RXN0-582, TRANS-RXN-158 |
| yfcD | 0,543899051 | 0,016602929 | |
| hisS | 0,543325317 | 0,026474083 | HISTIDINE--TRNA-LIGASE-RXN |
| lptC | 0,542500483 | 0,035011158 | TRANS-RXN-237 |
| mukE | 0,538240977 | 0,01427088 | |
| uvrD | 0,530344617 | 0,042210583 | |
| truB | 0,519906309 | 0,038803321 | RXN-11839 |
| hldE | 0,513175903 | 0,027040645 | |
| holB | 0,511218172 | 0,047804045 | |
| mltA | 0,510350598 | 0,044433096 | RXN0-5190 |
| ydaM | 0,51021159 | 0,043992381 | RXN0-5359 |
| rcsB | 0,507843401 | 0,013399631 | |
| ychF | 0,496272048 | 0,032337616 | |
| yjaG | 0,494420732 | 0,048713081 | |
| metQ | 0,493804332 | 0,028036813 | TRANS-RXN0-511, TRANS-RXN0-510, RXN0-4522, TRANS-RXN0-202 |
| gmhA | 0,493685687 | 0,035750782 | ACYLCOADEHYDROG-RXN |
| rng | 0,484176161 | 0,02090684 | RXN0-6523 |
| ytjC | 0,482518265 | 0,035472685 | |
| mukF | 0,476718881 | 0,039300633 | |
| rbfA | 0,464294367 | 0,037866341 | |

Supplementary Table S2. Significantly down-regulated genes in E. coli treated with OTC.

**Significantly (FDR < 0.05) down-regulated genes in *E. coli MG1655::* tetA *grown in the presence of 14 mg/L oxytetracycline, corresponding to 1/2 MIC of the strain.***

| Gene | logFC | FDR | Metabolic reactions |
|---|---|---|---|
| trpE | -7,490040893 | 0,000123454 | ANTHRANSYN-RXN |
| trpD | -7,164597417 | 0,000293843 | PRTRANS-RXN, ANTHRANSYN-RXN |
| mtr | -6,435021452 | 0,00000196 | TRANS-RXN-142, TRANS-RXN-76 |
| tdcB | -5,933929415 | 1,9E-15 | RXN-15125, 4.3.1.17-RXN, RXN-15122, THREDEHYD-RXN |
| trpC | -5,788207896 | 0,00153234 | IGPSYN-RXN, PRAISOM-RXN |
| dcuC | -5,725190858 | 2,43E-10 | TRANS-RXN-106D, TRANS-RXN-106B, TRANS-RXN-106A, TRANS-RXN-106 |
| nrfC | -5,617858522 | 1,89E-08 | RXN0-5198 |
| nrfB | -5,360172401 | 1,16E-09 | RXN0-5198 |
| nrfD | -5,288687271 | 0,000000781 | RXN0-5198 |
| trpB | -4,964747999 | 0,002400803 | RXN0-2382, TRYPSYN-RXN |
| trpA | -4,764726983 | 0,002525509 | RXN0-2381, TRYPSYN-RXN |
| gudX | -4,742197273 | 1,24E-22 | |
| cadB | -4,711108238 | 1,75E-08 | TRANS-RXN0-212, TRANS-RXN-68 |
| nrfA | -4,696362402 | 0,00000105 | RXN0-5198 |
| tdcC | -4,41982805 | 2,06E-09 | TRANS-RXN-71, TRANS-RXN-72 |
| garD | -4,417510087 | 1,75E-08 | GALACTARDEHYDRA-RXN |
| narH | -4,41324081 | 0,00000955 | RXN0-3501, RXN-15119 |
| apaG | -4,261580893 | 0,00000676 | |
| garL | -4,20542075 | 1,02E-11 | KDGALDOL-RXN |
| hypB | -4,052915922 | 2,68E-11 | RXN0-5462 |
| tdcA | -4,020146815 | 0,00000108 | |
| dmsA | -4,00949196 | 9,96E-09 | DIMESULFREDUCT-RXN |
| yjjW | -3,885121622 | 6,03E-11 | |
| fdnH | -3,873516484 | 0,000000304 | FORMATEDEHYDROG-RXN |
| napA | -3,863500202 | 3,75E-09 | RXN0-6369 |
| narG | -3,852707312 | 0,0000357 | RXN0-3501, RXN-15119 |
| fdnG | -3,84069799 | 0,000000225 | FORMATEDEHYDROG-RXN |
| hcr | -3,821904611 | 1,15E-08 | RXN0-307 |
| dmsB | -3,816478429 | 0,00000135 | DIMESULFREDUCT-RXN |
| appY | -3,774778426 | 1,02E-11 | |

| | | | |
|---|---|---|---|
| gudP | -3,7043435 | 0,000000085 | TRANS-RXN0-523, TRANS-RXN0-204, TRANS-RXN0-203 |
| tdcD | -3,699620234 | 0,0000539 | PROPKIN-RXN |
| garK | -3,666383347 | 1,82E-15 | GKI-RXN |
| garP | -3,652906137 | 0,000024 | TRANS-RXN0-523, TRANS-RXN0-204, TRANS-RXN0-203 |
| hcp | -3,616083261 | 0,000420026 | HYDROXYLAMINE-REDUCTASE-RXN |
| narJ | -3,54375467 | 0,0000673 | |
| yjjI | -3,460086548 | 7,77E-08 | |
| nirD | -3,400075886 | 0,0000151 | RXN-13854 |
| gudD | -3,398723424 | 5,94E-22 | RXN0-5285, GLUCARDEHYDRA-RXN |
| hypC | -3,351916839 | 3,08E-11 | |
| adiY | -3,342003236 | 0,0000193 | |
| fdhF | -3,337642809 | 3,33E-10 | RXN-12274, RXN0-3281, FHLMULTI-RXN |
| gldA | -3,309623324 | 0,00000031 | RXN-8632, AMINOPROPDEHYDROG-RXN, GLYCDEH-RXN |
| napG | -3,275692926 | 0,000000413 | RXN0-6369 |
| preT | -3,25515538 | 3,08E-09 | DIHYDROURACIL-DEHYDROGENASE-NAD+-RXN, RXN0-6565 |
| napH | -3,230129441 | 0,0000011 | RXN0-6369 |
| narI | -3,227107044 | 0,0000941 | RXN0-3501, RXN-15119 |
| ycbJ | -3,16392026 | 0,00000326 | |
| hypA | -3,133745614 | 1,09E-13 | |
| ygeV | -3,122126001 | 2,32E-09 | |
| fdnI | -3,106160057 | 0,0000061 | FORMATEDEHYDROG-RXN |
| hybO | -3,069877585 | 0,00000174 | RXN0-5256 |
| nirB | -3,067977749 | 0,0000349 | RXN-13854 |
| hybA | -3,05412711 | 0,000000154 | RXN0-5256 |
| ycbC | -3,053983128 | 4,48E-11 | |
| garR | -3,049722158 | 1,03E-11 | TSA-REDUCT-RXN |
| preA | -3,042452556 | 0,000000008 | DIHYDROURACIL-DEHYDROGENASE-NAD+-RXN, RXN0-6565 |
| yhjA | -2,989870285 | 9,33E-10 | |
| ebgA | -2,975620468 | 1,25E-10 | |
| yqeB | -2,973505046 | 7,67E-10 | |
| nikB | -2,969793425 | 0,000303648 | ABC-20-RXN |
| cadA | -2,947978151 | 0,00000434 | LYSDECARBOX-RXN |
| dmsC | -2,945282181 | 0,00000265 | DIMESULFREDUCT-RXN |
| hypD | -2,937738654 | 1,6E-12 | |
| yqeC | -2,930739864 | 1,37E-16 | |

| | | | |
|---|---|---|---|
| sgcC | -2,915469842 | 2,39E-13 | |
| narK | -2,903766581 | 0,005140303 | TRANS-RXN-137, TRANS-RXN0-239 |
| ygcB | -2,852841164 | 2,7E-13 | SULFATE-ADENYLYLTRANS-RXN |
| yccM | -2,774204375 | 0,000143112 | |
| ttdR | -2,728332545 | 0,000000215 | |
| citB | -2,708992536 | 8,64E-10 | |
| araF | -2,703357742 | 6,57E-20 | ABC-2-RXN |
| hybB | -2,67888381 | 0,0000062 | RXN0-5256 |
| ygjH | -2,66243704 | 1,31E-08 | |
| napD | -2,643305673 | 9,24E-09 | |
| frwB | -2,600717831 | 5,16E-18 | |
| ydhV | -2,581787064 | 0,00000424 | |
| uxaB | -2,580811818 | 8,77E-10 | ALTRO-OXIDOREDUCT-RXN |
| hlyE | -2,55056778 | 0,000000118 | |
| yjfN | -2,536992013 | 6,39E-08 | |
| araG | -2,528742612 | 2,61E-14 | ABC-2-RXN |
| ynfE | -2,505468638 | 0,00000188 | RXN0-2101 |
| sgcX | -2,485670665 | 5,16E-18 | |
| yccT | -2,45244713 | 3,01E-09 | |
| agaW | -2,348410573 | 2,68E-11 | |
| yjiL | -2,346868932 | 0,000000579 | |
| srlE | -2,342423905 | 0,000000117 | TRANS-RXN-169, TRANS-RXN-156 |
| hypE | -2,329943902 | 2,06E-09 | |
| napB | -2,324550486 | 0,000541928 | RXN0-6369 |
| sgcB | -2,323873804 | 1,49E-08 | |
| nikC | -2,320595497 | 0,002441352 | ABC-20-RXN |
| nikD | -2,320314332 | 0,004377924 | ABC-20-RXN |
| ybfA | -2,315783031 | 0,000185642 | |
| fumB | -2,287909986 | 0,00000655 | FUMHYDR-RXN, D--TARTRATE-DEHYDRATASE-RXN |
| ybiW | -2,2722663 | 0,00000285 | |
| dcuB | -2,24662576 | 0,0000117 | TRANS-RXN0-499, TRANS-RXN-106D, TRANS-RXN-106B, TRANS-RXN-106A, TRANS-RXN-106 |
| cdaR | -2,235919717 | 0,001123466 | |
| yjiM | -2,232780154 | 4,97E-11 | |
| bssS | -2,210392911 | 0,0000331 | |
| cydA | -2,184600384 | 0,000000404 | RXN0-5266 |

| | | | |
|---|---|---|---|
| cydB | -2,182984112 | 0,00000287 | RXN0-5266 |
| ydeN | -2,172215579 | 1,12E-08 | |
| atoS | -2,171614872 | 1,67E-11 | |
| flu | -2,158716403 | 5,15E-08 | |
| yfbM | -2,14546424 | 0,0000101 | |
| dcuA | -2,143737097 | 0,000000833 | TRANS-RXN-106D, TRANS-RXN-106B, TRANS-RXN-106A, TRANS-RXN-106 |
| yqhD | -2,136662902 | 2,27E-09 | ALCOHOL-DEHYDROGENASE-NADPORNOP+-RXN, ALDEHYDE-DEHYDROGENASE-NADP+-RXN, RXN0-6487 |
| melA | -2,131900478 | 5,56E-11 | ALPHAGALACTOSID-RXN |
| hmp | -2,131121753 | 0,0000257 | R621-RXN, 1.5.1.34-RXN |
| caiF | -2,122709476 | 1,49E-10 | |
| pka | -2,10525819 | 2,67E-09 | |
| rhmD | -2,093454577 | 7,67E-10 | TRANS-RXN-22 |
| yfbS | -2,084463816 | 0,00000121 | |
| mocA | -2,07117402 | 0,000296448 | RXN0-6254 |
| katG | -2,058532447 | 0,000000297 | CATAL-RXN, RXN-8667 |
| uspE | -2,049418284 | 0,000000761 | |
| yniA | -2,012477012 | 1,58E-08 | |
| yjdK | -2,006019253 | 0,00000175 | |
| srlD | -2,005163786 | 0,000000329 | SORB6PDEHYDROG-RXN |
| nikA | -1,978533396 | 0,013399631 | ABC-20-RXN |
| osmB | -1,97793068 | 0,00000294 | |
| ygdI | -1,968182104 | 1,08E-09 | |
| yhcC | -1,961081233 | 0,00000787 | |
| srlA | -1,939410039 | 0,0000154 | TRANS-RXN-169, TRANS-RXN-156 |
| cadC | -1,929780009 | 0,00000355 | |
| nrfE | -1,921970421 | 0,000635773 | |
| uxaC | -1,915051415 | 5,2E-10 | GLUCUROISOM-RXN, GALACTUROISOM-RXN |
| dsdX | -1,914311916 | 0,00000578 | TRANS-RXN0-495 |
| ravA | -1,907520699 | 0,000000154 | ADENOSINETRIPHOSPHATASE-RXN |
| yeaG | -1,907002864 | 0,000000781 | PROTEIN-KINASE-RXN |
| ycgB | -1,904085012 | 0,00000419 | |
| ybiY | -1,879009114 | 0,000198512 | |
| ygjR | -1,874197765 | 1,29E-08 | |
| bssR | -1,864602964 | 0,012215103 | |
| moaA | -1,851140081 | 0,00000715 | RXN-8340 |

| | | | | |
|---|---|---|---|---|
| ccmA | -1,850695376 | 0,0000337 | TRANS-RXN0-162 | |
| frdA | -1,830298737 | 0,0000281 | R601-RXN | |
| ykgG | -1,828912149 | 0,000185718 | | |
| moaD | -1,826259809 | 0,000157585 | RXN-8342 | |
| yagH | -1,822649508 | 1,39E-08 | | |
| yecH | -1,813424888 | 0,0000035 | | |
| srlB | -1,792331784 | 0,00000366 | TRANS-RXN-169, TRANS-RXN-156 | |
| ydhY | -1,789998081 | 0,001656911 | | |
| araB | -1,788228738 | 7,56E-09 | RXN0-5116 | |
| wrbA | -1,787651841 | 0,00000183 | NQOR-RXN | |
| ynfK | -1,786702159 | 0,007231866 | | ALCOHOL-DEHYDROG-RXN, ACETALD-DEHYDROG-RXN |
| ldtE | -1,784012106 | 0,0000138 | | |
| ykgF | -1,783021176 | 0,001279439 | | |
| zraS | -1,776628964 | 1,67E-08 | | |
| yeaH | -1,76801917 | 0,00000366 | | |
| napC | -1,763592288 | 0,001202264 | RXN0-6369 | |
| malE | -1,761452376 | 0,001214425 | TRANS-RXN0-504, TRANS-RXN0-503, ABC-16-RXN | |
| tdcE | -1,760444471 | 0,020165076 | KETOBUTFORMLY-RXN, PYRUVFORMLY-RXN | |
| yibH | -1,757498429 | 0,000000526 | | |
| yccJ | -1,751494649 | 0,00000167 | | |
| fsaB | -1,747663806 | 0,00000198 | RXN0-313 | |
| cydX | -1,746897354 | 0,000388574 | RXN0-5266 | |
| pfo | -1,740456173 | 8,53E-08 | | |
| hybC | -1,73925561 | 0,000198387 | RXN0-5256 | |
| hyaB | -1,738386058 | 0,000523001 | RXN0-5256 | |
| bsmA | -1,737099023 | 0,000366151 | | |
| atoC | -1,729030915 | 0,0000093 | | |
| sgcQ | -1,709917685 | 0,00000394 | | |
| yghA | -1,709765525 | 0,000000859 | | |
| araA | -1,702922685 | 0,00000693 | ARABISOM-RXN | |
| hyaA | -1,700385684 | 0,000361785 | RXN0-5256 | |
| aaaD | -1,699956181 | 0,018858766 | | |
| yhhA | -1,695846723 | 0,0000247 | | |
| ydcS | -1,689354259 | 0,000000308 | | |
| ychH | -1,688004131 | 0,007363847 | | |

| | | | |
|---|---|---|---|
| yhfG | -1,686350415 | 0,0000148 | |
| ysaA | -1,678556635 | 0,000190738 | |
| uxaA | -1,677694295 | 0,000000858 | ALTRODEHYDRAT-RXN |
| yhbS | -1,675695832 | 0,0000403 | |
| aldB | -1,656343948 | 0,00000216 | RXN0-3962 |
| yibI | -1,655113582 | 0,000480796 | |
| ykgE | -1,651796773 | 0,036474132 | |
| ybjD | -1,632691857 | 2,41E-09 | |
| hchA | -1,630840654 | 0,0000029 | GLYOXIII-RXN |
| yehD | -1,614608353 | 0,016835902 | |
| napF | -1,614025313 | 0,026206716 | |
| nrdD | -1,611216115 | 0,000519935 | RXN0-745, RXN0-746, RXN0-724, RXN0-723, RIBONUCLEOSIDE-TRIP-REDUCT-RXN |
| ymgE | -1,605486545 | 0,0000455 | |
| pabA | -1,595210643 | 0,0000196 | PABASYN-RXN |
| yjjP | -1,593433801 | 0,000280465 | |
| yhiN | -1,589012018 | 0,0000093 | |
| ynfD | -1,587636281 | 0,0000527 | |
| melB | -1,584261811 | 7,16E-08 | TRANS-RXN-94B, TRANS-RXN-94A, TRANS-RXN0-520, TRANS-RXN0-519, TRANS-RXN-94 |
| rihC | -1,583065149 | 0,00000166 | URIDINE-NUCLEOSIDASE-RXN, RXN0-363, INOSINE-NUCLEOSIDASE-RXN, ADENOSINE-NUCLEOSIDASE-RXN, RXN0-361 |
| ybeL | -1,582878674 | 0,000000836 | |
| yqgA | -1,580444052 | 0,000601742 | |
| ybjM | -1,5735386 | 0,000303389 | |
| fucI | -1,563704081 | 0,0000983 | FUCISOM-RXN, DARABISOM-RXN |
| nirC | -1,560505623 | 0,02090684 | TRANS-RXN-137 |
| ampC | -1,556219429 | 0,000000204 | BETA-LACTAMASE-RXN |
| acnA | -1,554897395 | 0,00000375 | ACONITATEHYDR-RXN, ACONITATEDEHYDR-RXN |
| iraD | -1,551618123 | 0,006644061 | |
| osmE | -1,549052626 | 0,00000174 | |
| ccmB | -1,54822654 | 0,002404881 | TRANS-RXN0-162 |
| ebgC | -1,537070726 | 0,005499889 | |
| fic | -1,535253062 | 0,0000561 | |
| csiE | -1,534611617 | 0,0000426 | |
| sodB | -1,533102663 | 0,00000495 | SUPEROX-DISMUT-RXN |
| pliG | -1,532088776 | 0,00000193 | |
| gpr | -1,530748793 | 0,000000193 | RXN0-5256 |

| | | | |
|---|---|---|---|
| araC | -1,526474397 | 0,000000134 | |
| sseA | -1,520286171 | 0,000000216 | RXN0-6945, MERCAPYSTRANS-RXN, THIOSULFATE-SULFURTRANSFERASE-RXN |
| glpK | -1,513111368 | 3,23E-10 | GLYCEROL-KIN-RXN |
| trpR | -1,510489389 | 0,0000211 | |
| dkgA | -1,508377459 | 0,0000417 | RXN0-4281, RXN0-1941, RXN0-7020 |
| dps | -1,503708868 | 0,0000132 | |
| yibT | -1,492188642 | 0,000471142 | |
| dgoA | -1,486492477 | 0,003479042 | DEHYDDEOXPHOSGALACT-ALDOL-RXN |
| yhbV | -1,481614538 | 0,0000193 | |
| sgcA | -1,469316547 | 0,0000788 | |
| nrdG | -1,461621668 | 0,011265626 | RNTRACTIV-RXN |
| sufA | -1,457583434 | 0,000245446 | |
| yciX | -1,454247197 | 0,0000449 | |
| yqfA | -1,454120912 | 0,0000198 | |
| uspB | -1,447873363 | 0,000121858 | |
| yhbU | -1,446604939 | 0,008655766 | |
| yidE | -1,446242681 | 1,27E-09 | |
| fucO | -1,435312993 | 0,00000557 | GLYCOLALDREDUCT-RXN, LACTALDREDUCT-RXN |
| ymfE | -1,42777522 | 0,0000016 | |
| araH | -1,425735974 | 2,59E-08 | ABC-2-RXN |
| moaC | -1,42521551 | 0,002162982 | |
| yqaE | -1,424852403 | 0,0000193 | |
| ydcT | -1,419273115 | 1,75E-08 | |
| ydhW | -1,418909309 | 0,000591825 | |
| hycI | -1,418905092 | 0,0000031 | RXN0-3364 |
| cbpM | -1,414415957 | 0,0000539 | |
| qmcA | -1,408287803 | 0,001898101 | |
| ynfF | -1,407448609 | 0,001522048 | RXN0-2101 |
| ybbJ | -1,403652879 | 0,0000998 | |
| viaA | -1,40204283 | 0,00000121 | |
| yohK | -1,398219108 | 0,000256224 | |
| glpF | -1,393522575 | 3,23E-10 | TRANS-RXN0-551, TRANS-RXN0-537, TRANS-RXN0-460, TRANS-RXN0-536, TRANS-RXN-131 |
| pepT | -1,392102236 | 0,0000427 | 3.4.11.4-RXN |
| moaE | -1,391206281 | 0,00183349 | RXN-8342 |
| qorA | -1,388609643 | 0,000119818 | RXN0-1741, RXN0-1804 |

| | | | |
|---|---|---|---|
| hypF | -1,387001697 | 0,00000773 | RXN0-6435 |
| lrhA | -1,385279985 | 0,000000013 | |
| narX | -1,382546334 | 0,00069328 | |
| udp | -1,37945101 | 0,0000123 | URPHOS-RXN |
| lamB | -1,3751795 | 0,032010785 | RXN0-1741, RXN0-1804 |
| ygiW | -1,37397233 | 0,0000179 | |
| cysQ | -1,371269957 | 0,0000216 | 325-BISPHOSPHATE-NUCLEOTIDASE-RXN |
| rhaS | -1,365851875 | 0,000199088 | |
| ybaL | -1,359649042 | 0,000125143 | |
| norR | -1,35944841 | 0,00000747 | |
| yadI | -1,358364651 | 0,000244867 | |
| yfeX | -1,353804407 | 0,0000818 | RXN0-6258 |
| ada | -1,346242297 | 0,00014917 | 2.1.1.63-RXN |
| yedP | -1,339889799 | 0,00000257 | |
| xylF | -1,337228971 | 0,0000431 | ABC-33-RXN |
| gadX | -1,333497441 | 0,00000159 | |
| yihV | -1,3304768 | 0,000898939 | RXN-15297 |
| ldtD | -1,328504307 | 0,0000158 | |
| zraR | -1,327041295 | 0,00000275 | |
| ade | -1,326484045 | 0,000000021 | |
| yhbT | -1,323122317 | 0,005685453 | |
| ldhA | -1,322571179 | 0,0000736 | DLACTDEHYDROGNAD-RXN |
| nupG | -1,320254123 | 0,0000243 | TRANS-RXN-108 |
| ygcN | -1,318238839 | 0,000142971 | |
| gutQ | -1,314497096 | 0,000157585 | DARAB5PISOM-RXN |
| sufB | -1,314023302 | 0,000704823 | |
| treB | -1,313081049 | 0,000118301 | TRANS-RXN-168 |
| ygfK | -1,311812847 | 0,00000626 | |
| deoC | -1,310508934 | 0,0000473 | DEOXYRIBOSE-P-ALD-RXN |
| frdD | -1,310200727 | 0,001588207 | R601-RXN |
| dacC | -1,309224418 | 0,000000665 | 3.4.16.4-RXN |
| torD | -1,307790809 | 0,003666315 | |
| metE | -1,307682881 | 0,002682735 | HOMOCYSMET-RXN |
| yagG | -1,301728066 | 0,0000886 | |
| yfeC | -1,29421586 | 0,00000662 | |

| | | | |
|---|---|---|---|
| ymiA | -1,292582576 | 0,000185259 | |
| moaB | -1,284637267 | 0,00363503 | |
| pppA | -1,284539429 | 0,000295641 | |
| ydhF | -1,28419278 | 0,000000299 | |
| uspG | -1,281058889 | 0,001775465 | |
| ygdH | -1,271409616 | 0,00000279 | |
| yfeD | -1,263909599 | 0,0000703 | |
| srlR | -1,261684074 | 0,000190047 | |
| gadE | -1,257234643 | 0,012737192 | |
| fucP | -1,25711166 | 0,000658304 | TRANS-RXN-20 |
| hsrA | -1,253890728 | 0,003878734 | |
| gadC | -1,253732346 | 0,049580844 | TRANS-RXN-261 |
| hcaT | -1,253111507 | 0,000076 | |
| rhaR | -1,252755373 | 0,0000406 | |
| yfhM | -1,251669931 | 0,0000076 | |
| ybiB | -1,249218454 | 0,0000139 | |
| yohC | -1,247913619 | 0,017805611 | |
| slp | -1,247583203 | 0,002866923 | |
| sgbH | -1,246494839 | 0,036960432 | RXN0-705 |
| ybaE | -1,245499017 | 0,00016872 | |
| frdB | -1,244971554 | 0,00485393 | R601-RXN |
| ucpA | -1,244784335 | 0,001285354 | |
| yfjL | -1,243374481 | 0,000000579 | |
| yfjP | -1,235406667 | 0,005862076 | |
| copA | -1,232440573 | 0,000110466 | TRANS-RXN0-207 |
| treF | -1,231964633 | 0,000554915 | TREHALA-RXN |
| ybeQ | -1,22956057 | 0,000000308 | |
| aroL | -1,228956137 | 0,00078877 | SHIKIMATE-KINASE-RXN |
| cdd | -1,22689696 | 0,0000049 | CYTIDEAM2-RXN, CYTIDEAM-RXN |
| loiP | -1,226139098 | 0,0000556 | RXN0-3221 |
| yagJ | -1,226067597 | 0,012921058 | |
| clpA | -1,22462708 | 0,002289191 | 3.4.21.92-RXN |
| prlF | -1,222794034 | 0,000000162 | |
| blc | -1,220336791 | 0,00038414 | |
| yceM | -1,21777731 | 0,0000197 | |

| | | | |
|---|---|---|---|
| spy | -1,210075232 | 0,0000928 | |
| citA | -1,209832582 | 0,0000755 | |
| selA | -1,208188788 | 0,0000214 | 2.9.1.1-RXN |
| ybdK | -1,208101625 | 0,000188609 | |
| yodD | -1,202231668 | 0,002186428 | |
| dsdA | -1,196530329 | 0,0000117 | DSERDEAM-RXN |
| sodC | -1,196412622 | 0,001432665 | SUPEROX-DISMUT-RXN |
| yabI | -1,192347121 | 2,67E-09 | |
| zraP | -1,191989973 | 0,043624507 | |
| raiA | -1,190868668 | 0,003380426 | |
| ytfR | -1,184841821 | 0,002122489 | TRANS-RXN0-492, TRANS-RXN0-491, ABC-18-RXN |
| yaaJ | -1,18032431 | 0,00037148 | |
| pck | -1,176466405 | 0,002402745 | PEPCARBOXYKIN-RXN |
| ybjQ | -1,172877424 | 0,00020517 | |
| treA | -1,167241081 | 2,59E-08 | TREHALA-RXN |
| fsaA | -1,166981416 | 0,000523001 | RXN0-313 |
| yajO | -1,163983388 | 0,000124016 | |
| ccmC | -1,16334765 | 0,010705399 | TRANS-RXN0-162 |
| uspA | -1,162978782 | 0,005646758 | |
| glgS | -1,159088644 | 0,008195215 | |
| ybiI | -1,154390516 | 0,022720648 | |
| alaE | -1,152783365 | 0,000166207 | TRANS-RXN0-469 |
| ytfT | -1,149606097 | 0,02397808 | TRANS-RXN0-492, TRANS-RXN0-491, ABC-18-RXN |
| ubiF | -1,147133747 | 0,0022135 | OCTAPRENYL-METHYL-METHOXY-BENZOQ-OH-RXN |
| hyaC | -1,144054018 | 0,025576579 | RXN0-5256 |
| abrB | -1,139313643 | 0,013514459 | |
| ynjE | -1,138319045 | 0,001202264 | RXN-12473 |
| bfr | -1,136282543 | 0,000332887 | RXN0-1483 |
| ypfN | -1,129425706 | 0,002461701 | |
| exuT | -1,12838182 | 0,00000774 | TRANS-RXN-123, TRANS-RXN-35 |
| yhhT | -1,126569796 | 0,0000887 | |
| hyaD | -1,125196931 | 0,027185158 | |
| alkB | -1,12316635 | 0,00531585 | RXN-12353, RXN0-986, RXN0-985, RXN0-984 |
| cbpA | -1,123025831 | 0,000943935 | |
| gpmM | -1,120494031 | 0,000168217 | 3PGAREARR-RXN |

| | | | |
|---|---|---|---|
| dgoD | -1,11872786 | 0,001187281 | GALACTONDEHYDRAT-RXN |
| yeaO | -1,117270014 | 0,00133924 | |
| yjiY | -1,111410828 | 0,000873014 | |
| eutQ | -1,110230336 | 0,005083735 | |
| ybjP | -1,105093977 | 0,000198913 | |
| yhjD | -1,104141058 | 0,000545274 | |
| yghX | -1,103227951 | 0,0000805 | |
| ebgR | -1,095955332 | 0,000166806 | |
| yjdI | -1,095869171 | 0,000684941 | |
| rnr | -1,091564898 | 0,000107933 | RXN0-7023 |
| grxB | -1,089075126 | 0,0000818 | |
| arnF | -1,088605253 | 0,000678759 | TRANS-RXN0-276 |
| tpiA | -1,08644652 | 0,000185718 | TRIOSEPISOMERIZATION-RXN |
| ybhQ | -1,084004023 | 0,000635773 | |
| aes | -1,083749241 | 0,0000247 | ACETYLESTERASE-RXN |
| galT | -1,082617919 | 0,0000634 | GALACTURIDYLYLTRANS-RXN |
| pflB | -1,08074574 | 0,001829659 | |
| hemH | -1,08065133 | 0,000214243 | PROTOHEMEFERROCHELAT-RXN |
| ulaG | -1,079941502 | 0,008715886 | RXN0-5214 |
| sfsA | -1,077702808 | 0,0000638 | |
| rtcA | -1,077343954 | 0,00531585 | RNA-3-PHOSPHATE-CYCLASE-RXN, RXN0-6556 |
| xylB | -1,077262705 | 0,0000142 | XYLULOKIN-RXN, RXN0-382 |
| ydcH | -1,074503981 | 0,000210824 | |
| bcsC | -1,068796771 | 0,00000422 | |
| clsC | -1,065267992 | 0,0000184 | RXN0-7012 |
| yhbQ | -1,063690691 | 0,00131895 | |
| gmr | -1,056390238 | 0,0000122 | RXN0-4181 |
| ydhK | -1,051152512 | 0,00080102 | |
| ycaC | -1,051137811 | 0,000332887 | |
| xylA | -1,048358295 | 0,0000107 | XYLISOM-RXN |
| rtcB | -1,047078659 | 0,002196833 | RXN0-6566 |
| feaB | -1,0446154 | 0,000035 | PHENDEHYD-RXN |
| kdpE | -1,043691122 | 0,001425576 | |
| yjgL | -1,040544982 | 0,0036343 | |
| glgC | -1,039134491 | 0,002964958 | GLUC1PADENYLTRANS-RXN |

| | | | |
|---|---|---|---|
| yahB | -1,035451599 | 0,001562628 | |
| idnO | -1,031427484 | 0,014411284 | GLUCONATE-5-DEHYDROGENASE-RXN |
| acuI | -1,029788451 | 0,000201036 | NANE-RXN |
| yacL | -1,028423925 | 0,002966299 | |
| sbmC | -1,026587757 | 0,000841072 | |
| dtpB | -1,024252682 | 0,000129516 | TRANS-RXN0-267, TRANS-RXN0-288 |
| rhsD | -1,022267498 | 0,000943935 | |
| mrr | -1,021821471 | 0,001009029 | |
| dmlA | -1,021674617 | 0,005929576 | 1.1.1.83-RXN |
| argO | -1,021350698 | 0,021666051 | |
| yeeA | -1,021199089 | 0,00000131 | |
| dinG | -1,020913382 | 0,0000093 | |
| ydjY | -1,019975051 | 0,03393645 | |
| ilvY | -1,018705351 | 0,005639685 | |
| yfcZ | -1,017741472 | 0,013247843 | |
| yedY | -1,016473242 | 0,001406328 | |
| yahN | -1,014759188 | 0,009545383 | |
| eutH | -1,014213804 | 0,002873558 | |
| ldtA | -1,011196218 | 0,003102711 | |
| uspF | -1,009195975 | 0,009414723 | |
| yeeY | -1,005747486 | 0,014522644 | |
| fucK | -1,003804747 | 0,016547485 | DARABKIN-RXN, FUCULOKIN-RXN |
| ybaT | -1,002404027 | 0,003768161 | |
| rclA | -0,99796657 | 0,0000305 | |
| glgX | -0,996477337 | 0,001022672 | RXN0-5146 |
| yibA | -0,995271559 | 0,016630796 | |
| yiiS | -0,99310965 | 0,000250495 | |
| mtlR | -0,992619737 | 0,001257012 | |
| dsdC | -0,99143347 | 0,0000834 | |
| yihS | -0,989819522 | 0,002997352 | RXN-15296, MANNOSE-ISOMERASE-RXN |
| yccX | -0,989461403 | 0,001698199 | ACYLPHOSPHATASE-RXN |
| ytfQ | -0,988373767 | 0,00521345 | TRANS-RXN0-492, TRANS-RXN0-491, ABC-18-RXN |
| yeeP | -0,987105581 | 0,015961742 | |
| ydaN | -0,986050772 | 0,0000473 | |
| hspQ | -0,985661708 | 0,020758333 | |

| | | | |
|---|---|---|---|
| yhaH | -0,98230722 | 0,00013604 | |
| frlB | -0,978849661 | 0,027166513 | RXN0-963 |
| yceK | -0,977537834 | 0,007852512 | |
| torZ | -0,976890111 | 0,002784902 | RXN0-5264 |
| frdC | -0,974962633 | 0,024046647 | R601-RXN |
| ydbC | -0,97393286 | 0,00000396 | |
| glpT | -0,973600745 | 0,000958886 | TRANS-RXN-22 |
| yceH | -0,972804257 | 0,0000709 | |
| ybgE | -0,967452038 | 0,002964958 | |
| ldcC | -0,963483017 | 0,000135883 | LYSDECARBOX-RXN |
| ymdB | -0,962562014 | 0,001347226 | RXN0-7013 |
| cpdA | -0,955652312 | 0,000431193 | RXN0-5038 |
| ubiH | -0,955469737 | 0,000578946 | 2-OCTAPRENYL-6-METHOXYPHENOL-HYDROX-RXN |
| yhhJ | -0,955375224 | 0,000528731 | |
| ushA | -0,953620924 | 0,000237771 | UDPSUGARHYDRO-RXN, 5-NUCLEOTID-RXN, RXN0-3741 |
| yciS | -0,950599286 | 0,001588207 | PGPPHOSPHA-RXN, RXN0-4461, UNDECAPRENYL-DIPHOSPHATASE-RXN |
| pflD | -0,949623017 | 0,002508948 | |
| yceI | -0,948901316 | 0,002345828 | |
| sgcE | -0,9477213 | 0,024118622 | |
| ygbJ | -0,947249046 | 0,021522237 | |
| rhsB | -0,946039155 | 0,033532626 | |
| ydgD | -0,943839075 | 0,021522237 | |
| hdeB | -0,94321392 | 0,049481645 | |
| yfcH | -0,940734228 | 0,00011039 | |
| dctR | -0,940465569 | 0,005190851 | |
| ftsQ | -0,937150015 | 0,000539447 | |
| eno | -0,934365205 | 0,00531585 | 2PGADEHYDRAT-RXN |
| lsrA | -0,93356381 | 0,028291653 | TRANS-RXN0-454 |
| eutC | -0,930616903 | 0,005874442 | ETHAMLY-RXN |
| mmuM | -0,930298921 | 0,000431193 | MMUM-RXN |
| ygaU | -0,927758064 | 0,005887635 | |
| ydiV | -0,925558709 | 0,016394921 | |
| ylaB | -0,924493267 | 0,000482081 | |
| fucA | -0,923193966 | 0,00872118 | FUCPALDOL-RXN, DARABALDOL-RXN |
| chiP | -0,92098667 | 0,018290555 | TRANS-RXN0-445 |

| | | | |
|---|---|---|---|
| rcdA | -0,919101447 | 0,003862968 | |
| bcsE | -0,918104521 | 0,00013605 | |
| eptA | -0,918059947 | 0,003039177 | RXN-14379 |
| ybgO | -0,917344402 | 0,047730441 | |
| sufC | -0,916907049 | 0,002996873 | |
| rmuC | -0,916420665 | 0,0000363 | |
| yiiM | -0,915187071 | 0,002122489 | |
| arnT | -0,914214079 | 0,003075139 | RXN0-2001 |
| treC | -0,912267233 | 0,029366255 | TRE6PHYDRO-RXN |
| tktB | -0,910225956 | 0,011780466 | 1TRANSKETO-RXN, 2TRANSKETO-RXN |
| manZ | -0,90949193 | 0,005362279 | TRANS-RXN0-540, TRANS-RXN0-446, TRANS-RXN-167A, TRANS-RXN-157, TRANS-RXN-167, TRANS-RXN-165, TRANS-RXN-158A |
| galE | -0,908789647 | 0,000287548 | UDPGLUCEPIM-RXN |
| pgpB | -0,908516764 | 0,003487297 | PGPPHOSPHA-RXN, RXN0-4461, UNDECAPRENYL-DIPHOSPHATASE-RXN |
| bolA | -0,907779167 | 0,013508243 | |
| yejO | -0,903600335 | 0,0000107 | |
| cptA | -0,902154567 | 0,0000426 | |
| hipA | -0,89647722 | 0,001335245 | RXN0-5211 |
| waaS | -0,895208428 | 0,020597631 | RXN0-5129 |
| ycgV | -0,893988899 | 0,002511234 | |
| ypdC | -0,892255699 | 0,00443658 | |
| tabA | -0,890528249 | 0,001457364 | |
| yeiE | -0,886024838 | 0,000361495 | |
| gspO | -0,885761733 | 0,005087834 | 3.4.23.43-RXN |
| iaaA | -0,88467336 | 0,000297543 | RXN0-3241, ASPARAGHYD-RXN |
| elbB | -0,884519741 | 0,000824556 | |
| ybiA | -0,883447443 | 0,043409812 | |
| yraJ | -0,878994206 | 0,003403489 | |
| mdfA | -0,878614281 | 0,003331952 | TRANS-RXN-42, TRANS-RXN-101, TRANS-RXN-44 |
| rbbA | -0,873555965 | 0,002192238 | |
| ydcK | -0,873511946 | 0,01105036 | |
| glsA | -0,873302709 | 0,005464257 | TRANS-RXN0-207 |
| deoA | -0,870661509 | 0,00281669 | THYM-PHOSPH-RXN, URA-PHOSPH-RXN |
| frlR | -0,87007149 | 0,00833006 | |
| yddH | -0,869851681 | 0,001279439 | |
| ypjA | -0,866571146 | 0,000129516 | |

| | | | |
|---|---|---|---|
| rclR | -0,863818714 | 0,023017435 | |
| kdgT | -0,86317711 | 0,00717885 | TRANS-RXN-113 |
| yphE | -0,861105765 | 0,016090086 | |
| rssA | -0,85941111 | 0,000183668 | |
| ydjZ | -0,85831528 | 0,042907547 | |
| melR | -0,858281421 | 0,037609 | |
| pspE | -0,85729516 | 0,015964163 | THIOSULFATE-SULFURTRANSFERASE-RXN |
| tqsA | -0,856856111 | 0,02250233 | TRANS-RXN0-453 |
| malI | -0,855243449 | 0,004322001 | |
| fliY | -0,853589107 | 0,005438336 | |
| ycaI | -0,852791057 | 0,003893236 | |
| sgrR | -0,852756391 | 0,000348862 | |
| ftnB | -0,849668251 | 0,003143853 | |
| yjjL | -0,848989612 | 0,003846751 | TRANS-RXN0-227 |
| ykfB | -0,848518151 | 0,01006147 | |
| qorB | -0,844968696 | 0,0444514 | |
| yegP | -0,844491144 | 0,040426233 | |
| narL | -0,843800019 | 0,023252376 | |
| lfhA | -0,843225725 | 0,014487772 | |
| eutT | -0,841721698 | 0,019352554 | |
| yneO | -0,841571839 | 0,0000676 | |
| uspD | -0,836977642 | 0,010287156 | |
| galK | -0,835765306 | 0,006625906 | GALACTOKIN-RXN |
| narZ | -0,830678067 | 0,039729648 | RXN0-3501, RXN-15119 |
| nagE | -0,826130274 | 0,009366949 | TRANS-RXN-167, TRANS-RXN-167A |
| exuR | -0,825140895 | 0,006767293 | |
| nagA | -0,824665624 | 0,000174984 | NAG6PDEACET-RXN |
| ydhS | -0,822847478 | 0,002122489 | |
| mtlA | -0,822505151 | 0,001159319 | TRANS-RXN-156, TRANS-RXN-169 |
| tyrR | -0,821597192 | 0,001917665 | |
| yjdJ | -0,819326788 | 0,002380734 | |
| dgoK | -0,818742108 | 0,006104914 | DEHYDDEOXGALACTKIN-RXN |
| ypdA | -0,818297621 | 0,003829691 | |
| pepP | -0,816930766 | 0,005844729 | 3.4.11.9-RXN |
| ybcM | -0,816644314 | 0,014116658 | |

| | | | |
|---|---|---|---|
| ybdD | -0,814784497 | 0,004275857 | |
| adhE | -0,813920708 | 0,007219958 | PFLDEACTIV-RXN, PYRUVFORMLY-RXN, KETOBUTFORMLY-RXN, ALCOHOL-DEHYDROG-GENERIC-RXN, |
| eco | -0,811913033 | 0,002357757 | |
| dhaK | -0,809353226 | 0,015799937 | 2.7.1.121-RXN |
| znuC | -0,807836906 | 0,011577672 | ABC-63-RXN |
| potE | -0,804550188 | 0,017988376 | TRANS-RXN0-211, TRANS-RXN-69 |
| dinD | -0,803959559 | 0,007651478 | |
| uxuR | -0,801938073 | 0,004561119 | |
| yghB | -0,801762966 | 0,008824042 | |
| nagC | -0,800873779 | 0,000225696 | |
| poxB | -0,799091888 | 0,014534943 | RXN0-2022, RXN-11496 |
| ydcU | -0,798532262 | 0,001800358 | |
| manX | -0,795741021 | 0,017909482 | TRANS-RXN0-540, TRANS-RXN0-446, TRANS-RXN-167A, TRANS-RXN-157, TRANS-RXN-167, TRANS-RXN-165, TRANS-RXN-158A |
| manY | -0,794140587 | 0,017048668 | TRANS-RXN0-540, TRANS-RXN0-446, TRANS-RXN-167A, TRANS-RXN-157, TRANS-RXN-167, TRANS-RXN-165, TRANS-RXN-158A |
| ybhL | -0,794114841 | 0,022263413 | |
| rhsC | -0,791300146 | 0,032184994 | |
| yejA | -0,791212067 | 0,002400803 | ABC-22-RXN |
| ygbK | -0,790154153 | 0,043177035 | |
| ycfH | -0,78931857 | 0,004802713 | |
| yfbT | -0,78929656 | 0,001229126 | GLYCEROL-1-PHOSPHATASE-RXN, SUGAR-PHOSPHATASE-RXN |
| yghJ | -0,788443417 | 0,001024077 | |
| glgA | -0,786862614 | 0,011643336 | GLYCOGENSYN-RXN |
| yqjG | -0,781621135 | 0,005137703 | RXN0-7010 |
| basS | -0,779550647 | 0,010350653 | |
| yhaV | -0,779454463 | 0,004982247 | RXN0-6529, RXN0-6528 |
| ribB | -0,778800124 | 0,018335542 | DIOHBUTANONEPSYN-RXN |
| fadR | -0,77864737 | 0,000851678 | |
| ygcE | -0,778338217 | 0,047730441 | |
| ilvC | -0,774262775 | 0,032471182 | ACETOLACTREDUCTOISOM-RXN, ACETOOHBUTREDUCTOISOM-RXN, 2-DEHYDROPANTOATE-REDUCT-RXN |
| speB | -0,772993794 | 0,013890197 | AGMATIN-RXN |
| ydcJ | -0,771345338 | 0,012591062 | |
| chaA_1 | -0,770860031 | 0,010675659 | |
| cra | -0,768024072 | 0,00257237 | |
| yohF | -0,767026201 | 0,00615423 | ACETOINDEHYDROG-A-RXN |
| aegA | -0,766685727 | 0,023958845 | |

| | | | |
|---|---|---|---|
| gsiC | -0,766305082 | 0,007242428 | RXN0-11 |
| yafC | -0,765551593 | 0,021748644 | |
| kbl | -0,76283985 | 0,011205619 | AKBLIG-RXN |
| yfjK | -0,76172568 | 0,000136622 | |
| yieP | -0,761001053 | 0,01419391 | |
| idnD | -0,759118749 | 0,016394921 | 1.1.1.264-RXN |
| nadA | -0,757569119 | 0,014934372 | QUINOLINATE-SYNTHA-RXN |
| alsB | -0,755448386 | 0,033983993 | ABC-42-RXN |
| nanE | -0,754617306 | 0,016363598 | NANE-RXN |
| polB | -0,752941231 | 0,001123528 | RXN0-4961, DNA-DIRECTED-DNA-POLYMERASE-RXN |
| ybiO | -0,750732841 | 0,004266955 | |
| yafV | -0,748675285 | 0,001254047 | |
| ybdH | -0,747476607 | 0,025114594 | |
| yicH | -0,746213186 | 0,001816492 | |
| agp | -0,7446406 | 0,039156751 | RXN0-1001, GLUCOSE-1-PHOSPHAT-RXN |
| yjgA | -0,743865918 | 0,004449473 | |
| ftsZ | -0,743479431 | 0,006078324 | |
| mtlD | -0,742811413 | 0,001598713 | MANNPDEHYDROG-RXN |
| clcA | -0,742405888 | 0,004247191 | RXN0-2501 |
| glpX | -0,74117509 | 0,004110535 | F16BDEPHOS-RXN |
| marC | -0,741011919 | 0,002801369 | |
| dinB | -0,740023367 | 0,017885238 | DNA-DIRECTED-DNA-POLYMERASE-RXN |
| yfdC | -0,739187992 | 0,01419391 | |
| kdpD | -0,736954225 | 0,016394921 | |
| gsiA | -0,735347461 | 0,000156666 | RXN0-11 |
| chiA | -0,734306319 | 0,001271893 | RXN0-4082 |
| sugE | -0,730603366 | 0,041185196 | |
| tdh | -0,727001099 | 0,016602929 | THREODEHYD-RXN |
| ygdR | -0,725749998 | 0,001489844 | |
| ydjF | -0,719107309 | 0,02870732 | |
| adhP | -0,718782227 | 0,046023454 | ALCOHOL-DEHYDROG-RXN, ALCOHOL-DEHYDROG-GENERIC-RXN |
| yfiR | -0,715749994 | 0,040625287 | |
| zntA | -0,714901721 | 0,004210445 | RXN0-5205 |
| yaiA | -0,714175563 | 0,012200159 | |
| pbpC | -0,713806537 | 0,012830047 | RXN0-5405 |

| | | | |
|---|---|---|---|
| brnQ | -0,712307614 | 0,012926792 | TRANS-RXN-126B, TRANS-RXN-126A, TRANS-RXN-126 |
| asmA | -0,704695216 | 0,000160615 | |
| ydhZ | -0,704293366 | 0,003187797 | |
| basR | -0,702357115 | 0,002475354 | |
| sdhE | -0,701147243 | 0,005362279 | |
| pflA | -0,699449822 | 0,003590178 | TDCEACT1-RXN, KETOBUTFORMLY-RXN, PYRUVFORMLY-RXN, 1.97.1.4-A-RXN |
| glpQ | -0,697028191 | 0,007687374 | GLYCPDIESTER-RXN |
| yaeH | -0,696491777 | 0,002395585 | |
| nhoA | -0,696019593 | 0,031974612 | ARYLAMINE-N-ACETYLTRANSFERASE-RXN, 2.3.1.118-RXN |
| yfiL | -0,695394353 | 0,048402471 | |
| sbcB | -0,693051311 | 0,011229999 | 3.1.11.1-RXN |
| rssB | -0,692820239 | 0,012147273 | |
| ynfB | -0,692769033 | 0,00827684 | |
| aidB | -0,692678718 | 0,035932413 | RXN0-2301 |
| yjhG | -0,68695659 | 0,022416725 | XYLONATE-DEHYDRATASE-RXN |
| smf | -0,685003099 | 0,00708552 | |
| ygiV | -0,684678289 | 0,025869575 | |
| yhhN | -0,684507637 | 0,011419273 | |
| hyfG | -0,683017808 | 0,044991688 | RXN0-3283 |
| yacH | -0,682301461 | 0,040064096 | |
| zitB | -0,677095403 | 0,016394921 | TRANS-RXN0-200 |
| yfcG | -0,676150662 | 0,011626999 | RXN0-6256 |
| yfbV | -0,675862238 | 0,018178596 | |
| ypfJ | -0,673972608 | 0,044113528 | |
| yjbB | -0,672234102 | 0,001354592 | TRANS-RXN0-470 |
| mtgA | -0,670449368 | 0,004263132 | RXN0-5405 |
| ybeX | -0,669004888 | 0,019389828 | |
| folA | -0,668317968 | 0,030924799 | DIHYDROFOLATEREDUCT-RXN |
| yqiA | -0,663922528 | 0,004247191 | |
| ydcV | -0,657213659 | 0,040625287 | |
| xylG | -0,654830442 | 0,036200138 | ABC-33-RXN |
| tatB | -0,652489871 | 0,01105036 | TRANS-RXN0-181 |
| sapD | -0,651747746 | 0,011229999 | |
| recN | -0,649140466 | 0,025858473 | |
| yidF | -0,646651326 | 0,036200138 | |

| | | | |
|---|---|---|---|
| hrpA | -0,645896737 | 0,010637002 | |
| yccU | -0,643498831 | 0,037260653 | |
| ybeY | -0,639399036 | 0,018385475 | |
| murD | -0,639277326 | 0,014558848 | UDP-NACMURALA-GLU-LIG-RXN |
| cysN | -0,637190381 | 0,036478606 | SULFATE-ADENYLYLTRANS-RXN |
| yafS | -0,633150626 | 0,004275857 | |
| glnG | -0,631671802 | 0,028486076 | |
| ycaP | -0,629554967 | 0,005610369 | |
| rlmB | -0,629266681 | 0,006363813 | RXN-11588 |
| lpxL | -0,62675455 | 0,04330344 | LAUROYLACYLTRAN-RXN |
| hdhA | -0,623039615 | 0,043712439 | 7-ALPHA-HYDROXYSTEROID-DEH-RXN |
| yhjY | -0,622020639 | 0,012433768 | |
| uxuA | -0,620961568 | 0,013717169 | MANNONDEHYDRAT-RXN |
| nhaA | -0,618490316 | 0,030442773 | TRANS-RXN-129 |
| secM | -0,613744897 | 0,01427088 | |
| polA | -0,609687085 | 0,017725075 | RXN0-5039, RXN0-4961, DNA-DIRECTED-DNA-POLYMERASE-RXN |
| ytfB | -0,60796918 | 0,018791046 | |
| rpoE | -0,605832327 | 0,029400791 | |
| metH | -0,603502559 | 0,015980769 | HOMOCYSMETB12-RXN |
| yaeP | -0,602963798 | 0,014319757 | |
| tatA | -0,602450736 | 0,023484834 | TRANS-RXN0-181 |
| ddlB | -0,601112796 | 0,026491154 | DALADALALIG-RXN |
| yrdD | -0,598317692 | 0,021350317 | |
| murG | -0,597477189 | 0,014072659 | NACGLCTRANS-RXN |
| yneE | -0,596908566 | 0,047503155 | |
| prkB | -0,594695046 | 0,020318625 | |
| nnr | -0,594483852 | 0,029770061 | 3.1.13.3-RXN |
| yeaE | -0,593388429 | 0,038638464 | RXN0-4281 |
| yciV | -0,593063941 | 0,017248717 | |
| ygdG | -0,592082672 | 0,018891423 | |
| chaB | -0,590348566 | 0,017237419 | |
| yhfA | -0,588010887 | 0,004275857 | |
| gadW | -0,585493624 | 0,027339193 | |
| tamA | -0,583649718 | 0,004982247 | |
| ftsW | -0,582895347 | 0,018947318 | TRANS-RXN0-286 |

| | | | |
|---|---|---|---|
| yhgE | -0,582805925 | 0,014522644 | |
| setA | -0,582067893 | 0,030924799 | TRANS-RXN-82 |
| gloB | -0,575528802 | 0,014926266 | GLYOXII-RXN |
| selD | -0,572011025 | 0,027155818 | 2.7.9.3-RXN |
| yqcA | -0,568952426 | 0,03981442 | |
| ybhG | -0,568503046 | 0,019559314 | |
| yafD | -0,56722769 | 0,020758333 | |
| dcyD | -0,56513348 | 0,017341801 | DCYSDESULF-RXN, ALADEHYDCHLORO-RXN |
| yjiV | -0,564128673 | 0,02163186 | TRANS-RXN0-492, TRANS-RXN0-491, ABC-18-RXN |
| ribD | -0,562955253 | 0,023747222 | RIBOFLAVINSYNDEAM-RXN, RIBOFLAVINSYNREDUC-RXN |
| add | -0,562882137 | 0,035732714 | ADDALT-RXN, ADENODEAMIN-RXN |
| helD | -0,562370406 | 0,01565673 | |
| ftsA | -0,56134588 | 0,026904748 | |
| phoQ | -0,560938403 | 0,010353863 | |
| matP | -0,560536925 | 0,038417271 | |
| glmZ | -0,546189873 | 0,028872395 | |
| pgpC | -0,544364728 | 0,026283571 | PGPPHOSPHA-RXN |
| hemD | -0,542385411 | 0,006636421 | UROGENIIISYN-RXN |
| selB | -0,540446485 | 0,037435016 | RXN0-5462 |
| opgB | -0,540186068 | 0,015562775 | PGLYCEROLTRANSII-RXN, PGLYCEROLTRANSI-RXN |
| ypdB | -0,539067041 | 0,040952509 | |
| yecA | -0,536589992 | 0,044023136 | |
| mngB | -0,536102062 | 0,042848437 | RXN0-5216 |
| yhhX | -0,529674493 | 0,043562342 | |
| kdgR | -0,529551641 | 0,02481286 | |
| yqjA | -0,523492842 | 0,047317973 | |
| hrpB | -0,516640671 | 0,019158491 | |
| rimJ | -0,505521967 | 0,02168752 | 2.3.1.128-RXN |
| ybhF | -0,501460658 | 0,018981031 | |
| gsiB | -0,500557394 | 0,034879801 | RXN0-11 |
| arcB | -0,498197634 | 0,041037356 | |
| orn | -0,497657297 | 0,020572304 | 3.1.13.3-RXN |
| ubiI | -0,493836941 | 0,038230706 | 2-OCTAPRENYLPHENOL-HYDROX-RXN |
| iraP | -0,493832858 | 0,03513381 | |
| yfbU | -0,489750855 | 0,039333973 | |

| | | | |
|---|---|---|---|
| rof | -0,480952399 | 0,046661652 | |
| ygfZ | -0,476667671 | 0,031617414 | |
| rlmD | -0,475838886 | 0,029400791 | RXN-11601 |
| nagB | -0,44615786 | 0,043017696 | GLUCOSAMINE-6-P-DEAMIN-RXN |
| envZ | -0,424701534 | 0,037944832 | |

Supplementary Table S3. Enrichment analysis based on up-regulated genes

**Enrichement analysis of biological functions, moleclar processes, cellular components and KEGG pathways based on up regulated genes with LogFC >2**

**Biological functions**

| *Pathway ID* | *pathway description* | *count in gene set* | *false discovery rate* |
|---|---|---|---|
| GO:0006189 | de novo IMP biosynthetic process | 8 | 2.18e-08 |
| GO:0009156 | ribonucleoside monophosphate biosynthetic process | 11 | 2.23e-07 |
| GO:0046390 | ribose phosphate biosynthetic process | 12 | 4.71e-07 |
| GO:0009260 | ribonucleotide biosynthetic process | 11 | 2.68e-06 |
| GO:0009152 | purine ribonucleotide biosynthetic process | 9 | 2.56e-05 |
| GO:1901564 | organonitrogen compound metabolic process | 34 | 2.93e-05 |
| GO:0009165 | nucleotide biosynthetic process | 12 | 0.000152 |
| GO:0044281 | small molecule metabolic process | 33 | 0.00141 |
| GO:0009102 | biotin biosynthetic process | 4 | 0.00442 |
| GO:1901566 | organonitrogen compound biosynthetic process | 23 | 0.00442 |
| GO:0018130 | heterocycle biosynthetic process | 25 | 0.0176 |
| GO:0055086 | nucleobase-containing small molecule metabolic process | 14 | 0.018 |
| GO:1901137 | carbohydrate derivative biosynthetic process | 14 | 0.0227 |
| GO:0006541 | glutamine metabolic process | 4 | 0.0231 |
| GO:0019752 | carboxylic acid metabolic process | 21 | 0.0231 |
| GO:0044271 | cellular nitrogen compound biosynthetic process | 27 | 0.0268 |
| GO:0006522 | alanine metabolic process | 3 | 0.0289 |
| GO:1901605 | alpha-amino acid metabolic process | 12 | 0.0308 |
| GO:1901362 | organic cyclic compound biosynthetic process | 24 | 0.0466 |
| GO:0009064 | glutamine family amino acid metabolic process | 6 | 0.0472 |
| GO:0009447 | putrescine catabolic process | 3 | 0.0498 |
| GO:0055072 | iron ion homeostasis | 5 | 0.0498 |

**Molecular processes**

| *Pathway ID* | *pathway description* | *count in gene set* | *false discovery rate* |
|---|---|---|---|
| GO:0051537 | 2 iron, 2 sulfur cluster binding | 8 | 0.000649 |

| | | | |
|---|---|---|---|
| GO:0000104 | succinate dehydrogenase activity | 4 | 0.00123 |
| GO:0003735 | structural constituent of ribosome | 9 | 0.00242 |
| GO:0004129 | cytochrome-c oxidase activity | 3 | 0.00389 |
| GO:0019843 | rRNA binding | 8 | 0.00389 |
| GO:0008177 | succinate dehydrogenase (ubiquinone) activity | 3 | 0.00835 |
| GO:0015453 | oxidoreduction-driven active transmembrane transporter | 3 | 0.00835 |
| GO:0009055 | electron carrier activity | 9 | 0.0152 |
| GO:0048039 | ubiquinone binding | 3 | 0.0174 |

**Cellular component**

| *Pathway ID* | *pathway description* | *count in gene set* | *false discovery rate* |
|---|---|---|---|
| GO:0022625 | cytosolic large ribosomal subunit | 8 | 2.62e-05 |
| GO:0022626 | cytosolic ribosome | 9 | 0.00012 |
| GO:0044391 | ribosomal subunit | 9 | 0.00012 |
| GO:0009319 | cytochrome o ubiquinol oxidase complex | 3 | 0.00108 |
| GO:0032991 | macromolecular complex | 19 | 0.0049 |

**Kegg pathway**

| *Pathway ID* | *pathway description* | *count in gene set* | *false discovery rate* |
|---|---|---|---|
| 1100 | Metabolic pathways | 33 | 0.000217 |
| 3010 | Ribosome | 9 | 0.000217 |
| 190 | Oxidative phosphorylation | 7 | 0.000704 |
| 230 | Purine metabolism | 10 | 0.000704 |
| 1110 | Biosynthesis of secondary metabolites | 17 | 0.0032 |
| 780 | Biotin metabolism | 4 | 0.00352 |
| 650 | Butanoate metabolism | 5 | 0.00816 |
| 20 | Citrate cycle (TCA cycle) | 4 | 0.0168 |
| 473 | D-Alanine metabolism | 2 | 0.0312 |
| 670 | One carbon pool by folate | 3 | 0.0312 |
| 250 | Alanine, aspartate and glutamate metabolism | 4 | 0.0452 |

Supplementary Table S4. Enrichment analysis based on down-regulated genes.

**Enrichment analysis of biological processes, molecular functions, cellular components and KEGG pathways based on down regulated genes with LogFC < -2**

**Biological processes**

| *Pathway ID* | *pathway description* | *count in gene set* | *false discovery rate* |
|---|---|---|---|
| GO:0009061 | anaerobic respiration | 16 | 8.44e-13 |
| GO:0015980 | energy derivation by oxidation of organic compounds | 18 | 1.11e-08 |
| GO:0055114 | oxidation-reduction process | 35 | 2.12e-08 |
| GO:0042128 | nitrate assimilation | 9 | 6.18e-08 |
| GO:0000162 | tryptophan biosynthetic process | 5 | 8.86e-07 |
| GO:0019579 | aldaric acid catabolic process | 5 | 8.86e-07 |
| GO:0042838 | D-glucarate catabolic process | 4 | 2.67e-05 |
| GO:0046392 | galactarate catabolic process | 4 | 2.67e-05 |
| GO:0043436 | oxoacid metabolic process | 26 | 0.000994 |
| GO:0044710 | single-organism metabolic process | 50 | 0.002 |
| GO:0019645 | anaerobic electron transport chain | 3 | 0.00355 |
| GO:0044699 | single-organism process | 66 | 0.00483 |
| GO:0043623 | cellular protein complex assembly | 5 | 0.0132 |
| GO:0009068 | aspartate family amino acid catabolic process | 4 | 0.014 |
| GO:0046395 | carboxylic acid catabolic process | 11 | 0.021 |
| GO:0006461 | protein complex assembly | 6 | 0.0217 |
| GO:0008152 | metabolic process | 71 | 0.0217 |
| GO:0051604 | protein maturation | 5 | 0.0217 |
| GO:0070271 | protein complex biogenesis | 6 | 0.0217 |
| GO:0070689 | L-threonine catabolic process to propionate | 3 | 0.0217 |
| GO:0017004 | cytochrome complex assembly | 4 | 0.0395 |
| GO:0044282 | small molecule catabolic process | 12 | 0.0434 |

**Molecular functions**

| *Pathway ID* | *pathway description* | *count in gene set* | *false discovery rate* |
|---|---|---|---|
| GO:0051536 | iron-sulfur cluster binding | 24 | 6.29e-13 |

| | | | |
|---|---|---|---|
| GO:0051539 | 4 iron, 4 sulfur cluster binding | 20 | 1.93e-11 |
| GO:0016661 | oxidoreductase activity, acting on other nitrogenous c | 10 | 5.41e-10 |
| GO:0046872 | metal ion binding | 43 | 9.9e-09 |
| GO:0009055 | electron carrier activity | 14 | 4.61e-07 |
| GO:0016491 | oxidoreductase activity | 29 | 9.99e-07 |
| GO:0098809 | nitrite reductase activity | 5 | 9.99e-07 |
| GO:0043167 | ion binding | 52 | 9.99e-06 |
| GO:0003954 | NADH dehydrogenase activity | 8 | 7.58e-05 |
| GO:0005488 | binding | 65 | 0.000257 |
| GO:0003674 | molecular_function | 82 | 0.000284 |
| GO:0030151 | molybdenum ion binding | 6 | 0.000284 |
| GO:0008940 | nitrate reductase activity | 4 | 0.000973 |
| GO:0042279 | nitrite reductase (cytochrome, ammonia-forming) act | 3 | 0.00132 |
| GO:0008863 | formate dehydrogenase (NAD+) activity | 4 | 0.00299 |
| GO:0004049 | anthranilate synthase activity | 2 | 0.0437 |
| GO:0004159 | dihydrouracil dehydrogenase (NAD+) activity | 2 | 0.0437 |
| GO:0004834 | tryptophan synthase activity | 2 | 0.0437 |
| GO:0008942 | nitrite reductase [NAD(P)H] activity | 2 | 0.0437 |

**Cellular components**

| *Pathway ID* | *pathway description* | *count in gene set* | *false discovery rate* |
|---|---|---|---|
| GO:1990204 | oxidoreductase complex | 10 | 9.73e-06 |
| GO:0009390 | dimethyl sulfoxide reductase complex | 3 | 0.00111 |
| GO:0009326 | formate dehydrogenase complex | 3 | 0.0072 |
| GO:0009325 | nitrate reductase complex | 3 | 0.0102 |
| GO:1902494 | catalytic complex | 12 | 0.0102 |
| GO:0009344 | nitrite reductase complex [NAD(P)H] | 2 | 0.0177 |
| GO:0042597 | periplasmic space | 12 | 0.0247 |

**KEGG pathways**

| *Pathway ID* | *pathway description* | *count in gene set* | *false discovery rate* |
|---|---|---|---|
| 910 | Nitrogen metabolism | 11 | 1.55e-10 |

| | | | |
|---|---|---|---|
| 400 | Phenylalanine, tyrosine and tryptophan biosynthesis | 5 | 0.00347 |
| 1120 | Microbial metabolism in diverse environments | 14 | 0.0287 |

**Supplementary material Table S5. Results of RNA seq for analysis of gene expression in MG1655::tetA grown with 14 mg/L OTC compared to growth in MH-2 media without OTC.**

| Gene | length | gccontent | logFC | logCPM | LR | PValue | FDR |
|---|---|---|---|---|---|---|---|
| b2788 | 1341 | 0,54 | -4,742197273 | 8,555626165 | 112,5021645 | 2,77362E-26 | 1,24314E-22 |
| b2787 | 1341 | 0,53 | -3,398723424 | 8,341469808 | 108,0262076 | 2,65248E-25 | 5,9442E-22 |
| b0331 | 891 | 0,56 | 3,801603072 | 2,8334571 | 101,6593114 | 6,5941E-24 | 9,85159E-21 |
| b1901 | 990 | 0,52 | -2,703357742 | 6,419783823 | 97,33226781 | 5,86172E-23 | 6,56806E-20 |
| b4305 | 1122 | 0,52 | -2,485670665 | 5,984488962 | 88,08480822 | 6,27074E-21 | 5,16425E-18 |
| b3947 | 2502 | 0,55 | -2,600717831 | 5,452028482 | 87,89184213 | 6,91332E-21 | 5,16425E-18 |
| b2876 | 771 | 0,5 | -2,930739864 | 6,095076781 | 81,10888925 | 2,13617E-19 | 1,36776E-16 |
| b3124 | 1146 | 0,54 | -3,666383347 | 9,375975037 | 75,73489896 | 3,24423E-18 | 1,81758E-15 |
| b3117 | 990 | 0,46 | -5,933929415 | 8,257969219 | 75,41763887 | 3,80971E-18 | 1,89724E-15 |
| b1900 | 1515 | 0,52 | -2,528742612 | 5,97770165 | 70,03306442 | 5,83187E-17 | 2,61384E-14 |
| b4375 | 1590 | 0,54 | 2,27959889 | 7,027777328 | 68,73159187 | 1,12819E-16 | 4,59686E-14 |
| b1286 | 1935 | 0,53 | 2,221537035 | 7,060659441 | 67,70331766 | 1,90043E-16 | 7,09812E-14 |
| b0775 | 1041 | 0,56 | 3,232663875 | 4,721582929 | 67,31417904 | 2,3151E-16 | 7,98177E-14 |
| b2726 | 351 | 0,53 | -3,133745614 | 5,628958432 | 66,55032284 | 3,41074E-16 | 1,09192E-13 |
| b4581 | 788 | 0,54 | 2,353043676 | 7,081853365 | 65,0990488 | 7,12264E-16 | 2,12825E-13 |
| b4304 | 1314 | 0,52 | -2,915469842 | 5,470973877 | 64,74256272 | 8,53512E-16 | 2,3909E-13 |
| b2761 | 2667 | 0,45 | -2,852841164 | 5,798336838 | 64,38467302 | 1,02351E-15 | 2,69846E-13 |
| b2500 | 639 | 0,54 | 2,638108 | 6,255293716 | 62,37379376 | 2,84078E-15 | 7,07355E-13 |
| b2040 | 900 | 0,47 | 1,709663996 | 6,829590744 | 62,12546226 | 3,22258E-15 | 7,60189E-13 |
| b2729 | 1122 | 0,55 | -2,937738654 | 7,329588141 | 60,55773128 | 7,14519E-15 | 1,60124E-12 |
| b3714 | 1338 | 0,54 | 1,825633956 | 6,396265899 | 57,77990537 | 2,93149E-14 | 6,25664E-12 |
| b1629 | 2223 | 0,55 | 2,18426479 | 5,650846806 | 57,12896047 | 4,08151E-14 | 8,31515E-12 |
| b0564 | 750 | 0,32 | -3,774778426 | 6,246693599 | 56,58177297 | 5,39092E-14 | 1,01948E-11 |
| b3126 | 771 | 0,53 | -4,20542075 | 10,0676425 | 56,55708361 | 5,45904E-14 | 1,01948E-11 |
| b3125 | 885 | 0,53 | -3,049722158 | 10,51871231 | 56,39563892 | 5,92617E-14 | 1,02689E-11 |
| b4064 | 1350 | 0,56 | 2,343145358 | 6,23755281 | 56,38544198 | 5,95698E-14 | 1,02689E-11 |
| b2319 | 1014 | 0,55 | 1,4924162 | 6,558808261 | 55,96764801 | 7,36734E-14 | 1,22298E-11 |
| b2219 | 1827 | 0,49 | -2,171614872 | 6,245546429 | 55,28884354 | 1,04058E-13 | 1,66566E-11 |
| b1002 | 1242 | 0,52 | -2,348410573 | 9,998218172 | 54,23328447 | 1,78045E-13 | 2,68058E-11 |
| b2727 | 873 | 0,55 | -4,052915922 | 8,061563227 | 54,21813571 | 1,79423E-13 | 2,68058E-11 |
| b2728 | 273 | 0,55 | -3,351916839 | 5,206706812 | 53,88141159 | 2,12963E-13 | 3,07903E-11 |
| b0522 | 1068 | 0,56 | 3,201604528 | 7,086467525 | 53,50556813 | 2,57861E-13 | 3,61166E-11 |
| b0920 | 780 | 0,52 | -3,053983128 | 6,268079799 | 53,02352019 | 3,29578E-13 | 4,47627E-11 |
| b0835 | 1326 | 0,53 | 2,45008684 | 4,727503864 | 52,94505176 | 3,43011E-13 | 4,52169E-11 |
| b4335 | 1152 | 0,54 | -2,232780154 | 6,808574997 | 52,70268475 | 3,8806E-13 | 4,96939E-11 |
| b4119 | 1356 | 0,52 | -2,131900478 | 6,262013874 | 52,42535054 | 4,46917E-13 | 5,56412E-11 |
| b4379 | 864 | 0,56 | -3,885121622 | 5,694961555 | 52,21315196 | 4,97915E-13 | 6,0315E-11 |
| b3076 | 3093 | 0,55 | -2,975620468 | 8,4057857 | 50,69477445 | 1,07906E-12 | 1,2511E-10 |
| b2014 | 1359 | 0,53 | 2,429626716 | 5,15730483 | 50,67743789 | 1,08864E-12 | 1,2511E-10 |
| b0034 | 396 | 0,46 | -2,122709476 | 4,780083944 | 50,28168835 | 1,33187E-12 | 1,49236E-10 |
| b1207 | 948 | 0,53 | 2,387285598 | 8,557352135 | 49,91315354 | 1,60704E-12 | 1,75677E-10 |
| b0621 | 1386 | 0,51 | -5,725190858 | 9,122073977 | 49,2291903 | 2,27736E-12 | 2,43027E-10 |
| b3927 | 846 | 0,51 | -1,393522575 | 5,96467183 | 48,5965418 | 3,14423E-12 | 3,23073E-10 |
| b3926 | 1509 | 0,54 | -1,513111368 | 7,622237545 | 48,57953026 | 3,17162E-12 | 3,23073E-10 |
| b4079 | 2148 | 0,54 | -3,337642809 | 7,509895708 | 48,47623697 | 3,34315E-12 | 3,32978E-10 |
| b3092 | 1413 | 0,54 | -1,915051415 | 6,878181511 | 47,55825368 | 5,33935E-12 | 5,20238E-10 |
| b2312 | 1518 | 0,53 | 2,673868008 | 7,78265447 | 47,4222065 | 5,72307E-12 | 5,45761E-10 |
| b2875 | 1626 | 0,52 | -2,973505046 | 6,618311518 | 46,70457147 | 8,25362E-12 | 7,66825E-10 |
| b2247 | 1206 | 0,52 | -2,093454577 | 4,806003872 | 46,67399662 | 8,38341E-12 | 7,66825E-10 |
| b0620 | 681 | 0,51 | -2,708992536 | 5,33140105 | 46,39956796 | 9,64368E-12 | 8,64459E-10 |
| b1521 | 1452 | 0,52 | -2,580811818 | 5,478715614 | 46,33244165 | 9,97978E-12 | 8,77047E-10 |
| b3518 | 1398 | 0,48 | -2,989870285 | 5,906111691 | 46,17232066 | 1,08296E-11 | 9,3343E-10 |
| b1628 | 579 | 0,55 | 2,652351777 | 3,866485364 | 46,02083826 | 1,17002E-11 | 9,89438E-10 |
| b2809 | 228 | 0,46 | -1,968182104 | 6,763347591 | 45,80410733 | 1,30689E-11 | 1,08472E-09 |
| b3256 | 1350 | 0,53 | 2,219903192 | 8,691988665 | 45,73252652 | 1,35553E-11 | 1,10463E-09 |
| b3871 | 1824 | 0,53 | 1,977091373 | 8,612790271 | 45,57760739 | 1,46709E-11 | 1,16042E-09 |
| b4071 | 567 | 0,54 | -5,360172401 | 6,866197502 | 45,56605907 | 1,47577E-11 | 1,16042E-09 |
| b0820 | 1593 | 0,53 | 1,38701826 | 6,571681969 | 45,39570766 | 1,60987E-11 | 1,24404E-09 |
| b3685 | 1662 | 0,53 | -1,446242681 | 7,052018681 | 45,32920897 | 1,66547E-11 | 1,26519E-09 |
| b0125 | 537 | 0,51 | 1,656637713 | 6,185613602 | 45,276651 | 1,71077E-11 | 1,27794E-09 |
| b3320 | 630 | 0,51 | 2,723482917 | 10,70419117 | 44,61856988 | 2,3941E-11 | 1,74403E-09 |
| b0458 | 471 | 0,47 | 1,825074088 | 6,202703914 | 44,60354675 | 2,41254E-11 | 1,74403E-09 |
| b0143 | 1398 | 0,56 | 1,450929661 | 7,337738071 | 44,23578681 | 2,9111E-11 | 2,05661E-09 |
| b2730 | 1011 | 0,57 | -2,329943902 | 6,629332477 | 44,1954716 | 2,97168E-11 | 2,05661E-09 |
| b3116 | 1332 | 0,5 | -4,41982805 | 8,028193133 | 44,18830016 | 2,98259E-11 | 2,05661E-09 |
| b0032 | 1149 | 0,53 | 3,08942406 | 5,311350982 | 44,09031296 | 3,13569E-11 | 2,12942E-09 |
| b3011 | 1164 | 0,55 | -2,136662902 | 7,20668396 | 43,93462714 | 3,39529E-11 | 2,27129E-09 |
| b2869 | 1779 | 0,46 | -3,122126001 | 9,165419121 | 43,86404026 | 3,51997E-11 | 2,32007E-09 |
| b0876 | 1659 | 0,53 | -1,632691857 | 6,979599263 | 43,7606619 | 3,71089E-11 | 2,41047E-09 |
| b4469 | 2220 | 0,55 | 1,901382345 | 5,410539622 | 43,5691244 | 4,09245E-11 | 2,62034E-09 |
| b0065 | 765 | 0,54 | -1,192347121 | 6,177677251 | 43,47692819 | 4,28988E-11 | 2,6717E-09 |
| b2584 | 2661 | 0,54 | -2,10525819 | 9,987420774 | 43,4760081 | 4,29189E-11 | 2,6717E-09 |
| b1658 | 1026 | 0,54 | 2,181859917 | 6,982384726 | 43,39479088 | 4,47378E-11 | 2,74678E-09 |
| b0964 | 663 | 0,51 | -2,45244713 | 4,908210777 | 43,18820137 | 4,97196E-11 | 3,0114E-09 |

| | | | | | | | |
|---|---|---|---|---|---|---|---|
| b2146 | 1239 | 0,51 | -3,25515538 | 8,900591771 | 43,1183485 | 5,15268E-11 | 3,07924E-09 |
| b0661 | 1425 | 0,54 | 1,335250261 | 7,067823291 | 43,07732294 | 5,26186E-11 | 3,08202E-09 |
| b1876 | 1734 | 0,53 | 1,340425091 | 7,433041801 | 43,06509017 | 5,29486E-11 | 3,08202E-09 |
| b0423 | 1449 | 0,52 | 1,577279302 | 5,690263587 | 42,68428708 | 6,43269E-11 | 3,69632E-09 |
| b2206 | 2487 | 0,56 | -3,863500202 | 10,56045528 | 42,63188579 | 6,60735E-11 | 3,74862E-09 |
| b3396 | 2553 | 0,56 | 1,547457191 | 5,679368771 | 42,53017725 | 6,96001E-11 | 3,89934E-09 |
| b1132 | 642 | 0,57 | 1,63829012 | 5,76552635 | 42,43926742 | 7,29114E-11 | 4,03443E-09 |
| b2537 | 891 | 0,5 | 3,08442948 | 5,718374991 | 42,27576353 | 7,92688E-11 | 4,33272E-09 |
| b2499 | 1038 | 0,53 | 3,16063697 | 7,810102852 | 41,67254153 | 1,07914E-10 | 5,82736E-09 |
| b0905 | 1761 | 0,52 | 1,858818321 | 5,456532482 | 41,52531728 | 1,16354E-10 | 6,15433E-09 |
| b4208 | 1413 | 0,53 | 2,394575466 | 7,688726805 | 41,496531 | 1,1808E-10 | 6,15433E-09 |
| b3654 | 1392 | 0,55 | 2,512156196 | 6,756836098 | 41,4963967 | 1,18088E-10 | 6,15433E-09 |
| b3407 | 2322 | 0,57 | 1,82391496 | 7,287058268 | 41,22818913 | 1,35455E-10 | 6,97826E-09 |
| b0063 | 1701 | 0,58 | -1,788228738 | 5,351817639 | 41,05051839 | 1,48345E-10 | 7,55547E-09 |
| b2147 | 1236 | 0,51 | -3,042452556 | 8,604103901 | 40,91593565 | 1,5892E-10 | 8,00314E-09 |
| b3185 | 258 | 0,5 | 2,308405487 | 8,208785962 | 40,78368173 | 1,70047E-10 | 8,46835E-09 |
| b0526 | 1386 | 0,54 | 1,421773634 | 6,726306594 | 40,71912985 | 1,75758E-10 | 8,65657E-09 |
| b2207 | 264 | 0,55 | -2,643305673 | 6,100326111 | 40,57053521 | 1,89645E-10 | 9,23901E-09 |
| b0894 | 2445 | 0,53 | -4,00949196 | 9,100491403 | 40,40307997 | 2,06615E-10 | 9,95752E-09 |
| b1498 | 1683 | 0,44 | -2,172215579 | 6,836791573 | 40,14760912 | 2,3548E-10 | 1,12279E-08 |
| b0872 | 969 | 0,55 | -3,821904611 | 5,825685741 | 40,07972646 | 2,43806E-10 | 1,15025E-08 |
| b3087 | 987 | 0,53 | -1,874197765 | 8,512698865 | 39,83161823 | 2,76827E-10 | 1,29244E-08 |
| b2289 | 939 | 0,5 | -1,385279985 | 7,506484853 | 39,79437088 | 2,82157E-10 | 1,30374E-08 |
| b3074 | 333 | 0,53 | -2,66243704 | 7,390181714 | 39,75876399 | 2,87348E-10 | 1,31391E-08 |
| b0336 | 1260 | 0,54 | 2,881675044 | 4,968002144 | 39,7230514 | 2,92651E-10 | 1,31391E-08 |
| b2313 | 489 | 0,49 | 2,748157014 | 6,450262277 | 39,71969992 | 2,93154E-10 | 1,31391E-08 |
| b0271 | 1611 | 0,64 | -1,822649508 | 5,246667655 | 39,58358832 | 3,14314E-10 | 1,39481E-08 |
| b4565 | 279 | 0,48 | -2,323873804 | 3,761061474 | 39,42957106 | 3,40107E-10 | 1,49447E-08 |
| b1281 | 738 | 0,53 | 2,154794175 | 4,93710497 | 39,3824489 | 3,48415E-10 | 1,51611E-08 |
| b1725 | 861 | 0,52 | -2,012477012 | 9,666894865 | 39,27946024 | 3,67285E-10 | 1,58286E-08 |
| b1208 | 852 | 0,53 | 1,309886193 | 6,734204616 | 39,16042436 | 3,90373E-10 | 1,66634E-08 |
| b4003 | 1398 | 0,54 | -1,776628964 | 5,859074975 | 39,13754156 | 3,94976E-10 | 1,66996E-08 |
| b2890 | 1518 | 0,53 | 1,805549216 | 8,744562139 | 39,1193467 | 3,98674E-10 | 1,66996E-08 |
| b3128 | 1572 | 0,53 | -4,417510087 | 11,7737481 | 39,00677784 | 4,22337E-10 | 1,74852E-08 |
| b4132 | 1335 | 0,49 | -4,711108238 | 5,828282101 | 38,96739938 | 4,30942E-10 | 1,74852E-08 |
| b1441 | 1014 | 0,54 | -1,419273115 | 7,857538007 | 38,96671311 | 4,31094E-10 | 1,74852E-08 |
| b1297 | 1419 | 0,53 | 3,546276925 | 6,69901334 | 38,95795213 | 4,33033E-10 | 1,74852E-08 |
| b3189 | 1260 | 0,53 | 1,779388755 | 7,216877273 | 38,90715458 | 4,44449E-10 | 1,77853E-08 |
| b1767 | 1017 | 0,51 | 1,811091053 | 5,775945722 | 38,88986696 | 4,48402E-10 | 1,77853E-08 |
| b3255 | 471 | 0,52 | 1,83512683 | 7,621616308 | 38,75140349 | 4,81364E-10 | 1,88608E-08 |
| b2400 | 1416 | 0,53 | 1,696162683 | 8,425606969 | 38,7378109 | 4,84728E-10 | 1,88608E-08 |
| b4072 | 672 | 0,58 | -5,617858522 | 6,268057951 | 38,72410834 | 4,88143E-10 | 1,88608E-08 |
| b2114 | 2034 | 0,53 | 1,407708196 | 8,099601449 | 38,68942125 | 4,96895E-10 | 1,90349E-08 |
| b0967 | 1191 | 0,52 | 1,449127794 | 5,245288011 | 38,59201824 | 5,22321E-10 | 1,98393E-08 |
| b3665 | 1767 | 0,55 | -1,326484045 | 5,969487345 | 38,46851426 | 5,56442E-10 | 2,09578E-08 |
| b2568 | 975 | 0,52 | 1,261340794 | 7,082521973 | 38,35751506 | 5,89008E-10 | 2,19995E-08 |
| b1197 | 1698 | 0,52 | -1,167241081 | 7,123440284 | 38,0062851 | 7,05171E-10 | 2,59103E-08 |
| b4460 | 987 | 0,51 | -1,425735974 | 5,424275576 | 38,00598918 | 7,05278E-10 | 2,59103E-08 |
| b0888 | 966 | 0,54 | 2,314191001 | 7,401431913 | 37,95000979 | 7,25807E-10 | 2,64477E-08 |
| b3737 | 240 | 0,54 | 2,037966477 | 9,049505636 | 37,91324922 | 7,39612E-10 | 2,67334E-08 |
| b3319 | 606 | 0,53 | 2,537826119 | 10,43499936 | 37,72165327 | 8,15938E-10 | 2,92563E-08 |
| b0778 | 678 | 0,54 | 2,721171524 | 3,213702547 | 37,61961953 | 8,59754E-10 | 3,05827E-08 |
| b2796 | 1290 | 0,52 | 2,309630635 | 9,170934356 | 37,37364088 | 9,75314E-10 | 3,44201E-08 |
| b2041 | 1086 | 0,44 | 1,596143977 | 7,001836771 | 37,22772969 | 1,05108E-09 | 3,68044E-08 |
| b2039 | 882 | 0,43 | 1,317802448 | 7,290912467 | 36,6619246 | 1,40497E-09 | 4,88145E-08 |
| b3705 | 1647 | 0,54 | 2,131835322 | 8,268798906 | 36,61739555 | 1,43743E-09 | 4,95582E-08 |
| b2000 | 3120 | 0,58 | -2,158716403 | 9,022315531 | 36,51482829 | 1,51509E-09 | 5,15268E-08 |
| b1805 | 1686 | 0,51 | 2,01703414 | 6,287200579 | 36,51170523 | 1,51752E-09 | 5,15268E-08 |
| b1435 | 1962 | 0,51 | 1,48081714 | 6,008565712 | 36,45314465 | 1,56381E-09 | 5,26991E-08 |
| b2517 | 1155 | 0,53 | 1,589196273 | 6,433980037 | 36,40291715 | 1,60463E-09 | 5,36712E-08 |
| b1422 | 924 | 0,51 | 3,940139206 | 6,036386974 | 36,12014579 | 1,85519E-09 | 6,15923E-08 |
| b3318 | 303 | 0,48 | 2,390459264 | 9,603744858 | 36,07510278 | 1,89857E-09 | 6,25691E-08 |
| b4188 | 276 | 0,51 | -2,536992013 | 9,153663587 | 36,01988223 | 1,95314E-09 | 6,38978E-08 |
| b0632 | 1212 | 0,49 | 1,844240042 | 6,957186472 | 35,90955493 | 2,06693E-09 | 6,71302E-08 |
| b3321 | 312 | 0,55 | 2,219922569 | 9,270384752 | 35,87440021 | 2,10456E-09 | 6,78606E-08 |
| b1634 | 1503 | 0,52 | 1,492618412 | 6,311233557 | 35,76664391 | 2,22424E-09 | 7,09356E-08 |
| b1131 | 1371 | 0,52 | 2,323771041 | 8,104292294 | 35,76022559 | 2,23158E-09 | 7,09356E-08 |
| b4120 | 1422 | 0,46 | -1,584261811 | 5,281742065 | 35,72722466 | 2,2697E-09 | 7,16394E-08 |
| b1088 | 522 | 0,5 | 1,730271963 | 9,08855988 | 35,69548121 | 2,30699E-09 | 7,2307E-08 |
| b4380 | 1551 | 0,53 | -3,460086548 | 8,38163891 | 35,54113752 | 2,49721E-09 | 7,77255E-08 |
| b0772 | 1284 | 0,53 | 1,688311188 | 7,098489281 | 35,52290358 | 2,52069E-09 | 7,79155E-08 |
| b0436 | 1299 | 0,51 | 2,203342997 | 9,607527158 | 35,39185795 | 2,69612E-09 | 8,27671E-08 |
| b2789 | 1353 | 0,52 | -3,7043435 | 9,479890381 | 35,32592098 | 2,78895E-09 | 8,50346E-08 |
| b1299 | 558 | 0,51 | 2,596379507 | 5,220281475 | 35,30721526 | 2,81587E-09 | 8,52671E-08 |
| b1378 | 3525 | 0,53 | -1,740456173 | 9,770421686 | 35,29428372 | 2,83463E-09 | 8,52671E-08 |
| b4006 | 1590 | 0,56 | 3,044494548 | 8,0911931 | 35,23216085 | 2,92651E-09 | 8,7444E-08 |
| b2171 | 573 | 0,5 | 1,606178199 | 5,167294103 | 35,00239694 | 3,293E-09 | 9,77431E-08 |
| b1778 | 414 | 0,49 | 1,839865106 | 7,440168167 | 34,96006448 | 3,36537E-09 | 9,88357E-08 |

| | | | | | | | |
|---|---|---|---|---|---|---|---|
| b1189 | 1299 | 0,56 | 2,86517943 | 10,06079499 | 34,95513249 | 3,37391E-09 | 9,88357E-08 |
| b1929 | 1206 | 0,55 | 1,968837076 | 7,493123404 | 34,92073167 | 3,43405E-09 | 9,99442E-08 |
| b2703 | 960 | 0,55 | -2,342423905 | 5,235923908 | 34,60306814 | 4,04269E-09 | 1,16899E-07 |
| b0432 | 948 | 0,51 | 3,704541592 | 9,540231598 | 34,57198964 | 4,10775E-09 | 1,178E-07 |
| b1182 | 912 | 0,41 | -2,55056778 | 4,65285162 | 34,56315794 | 4,12643E-09 | 1,178E-07 |
| b3250 | 1104 | 0,56 | 1,492642047 | 5,934144311 | 34,46636476 | 4,33681E-09 | 1,23023E-07 |
| b1062 | 1047 | 0,53 | 2,588662616 | 6,25390999 | 34,31269801 | 4,6931E-09 | 1,32292E-07 |
| b0064 | 879 | 0,53 | -1,526474397 | 8,006113419 | 34,28043552 | 4,77155E-09 | 1,33663E-07 |
| b0948 | 2109 | 0,54 | 1,289818185 | 6,774398869 | 34,17593873 | 5,03476E-09 | 1,4016E-07 |
| b1018 | 1128 | 0,51 | 3,705585586 | 3,497243431 | 34,11823349 | 5,1863E-09 | 1,43488E-07 |
| b0335 | 1887 | 0,58 | 1,534897961 | 3,96351899 | 34,01301309 | 5,47447E-09 | 1,50531E-07 |
| b2996 | 987 | 0,55 | -3,05412711 | 8,150080437 | 33,96234748 | 5,6189E-09 | 1,5356E-07 |
| b3746 | 1497 | 0,5 | -1,907520699 | 8,278710589 | 33,94749623 | 5,66195E-09 | 1,53799E-07 |
| b3251 | 1044 | 0,54 | 1,481240858 | 7,949945182 | 33,91973984 | 5,7433E-09 | 1,55069E-07 |
| b3764 | 339 | 0,48 | 1,236617389 | 7,704378361 | 33,85928444 | 5,92457E-09 | 1,59005E-07 |
| b3129 | 336 | 0,48 | -1,222794034 | 6,200025137 | 33,80585345 | 6,08954E-09 | 1,61518E-07 |
| b2797 | 1368 | 0,55 | 3,374004892 | 7,666028335 | 33,80561729 | 6,09028E-09 | 1,61518E-07 |
| b4287 | 768 | 0,54 | 1,60329664 | 4,010703421 | 33,75482944 | 6,25137E-09 | 1,64815E-07 |
| b2525 | 336 | 0,52 | 1,555524427 | 5,701733989 | 33,63001369 | 6,66562E-09 | 1,7471E-07 |
| b1095 | 1242 | 0,53 | 1,662688588 | 9,102843096 | 33,5360099 | 6,99567E-09 | 1,82294E-07 |
| b3342 | 375 | 0,53 | 2,175531944 | 9,744744925 | 33,46714963 | 7,24779E-09 | 1,87772E-07 |
| b3001 | 1041 | 0,52 | -1,530748793 | 8,911142069 | 33,40487199 | 7,48363E-09 | 1,92768E-07 |
| b0867 | 831 | 0,56 | -1,556219429 | 7,72476841 | 33,28261891 | 7,96917E-09 | 2,04102E-07 |
| b3736 | 471 | 0,53 | 2,069551354 | 10,12804936 | 33,20032649 | 8,31366E-09 | 2,11715E-07 |
| b3060 | 933 | 0,5 | -2,728332545 | 5,782731041 | 33,16019439 | 8,48702E-09 | 2,14909E-07 |
| b2521 | 846 | 0,53 | -1,520286171 | 9,361091204 | 33,13634511 | 8,59176E-09 | 2,16339E-07 |
| b1474 | 3048 | 0,54 | -3,84069799 | 9,461838879 | 33,05045777 | 8,97979E-09 | 2,24846E-07 |
| b3341 | 540 | 0,5 | 2,241794186 | 10,1008732 | 32,88066075 | 9,79933E-09 | 2,44003E-07 |
| b0914 | 1749 | 0,51 | 1,198512839 | 6,810812561 | 32,68714425 | 1,08251E-08 | 2,68055E-07 |
| b0474 | 645 | 0,52 | 2,012585735 | 7,027167557 | 32,6511644 | 1,10273E-08 | 2,71563E-07 |
| b0979 | 1137 | 0,53 | 1,806332286 | 6,547317359 | 32,50554935 | 1,18853E-08 | 2,91092E-07 |
| b1298 | 765 | 0,52 | 2,738052883 | 5,65792718 | 32,4727275 | 1,20877E-08 | 2,94441E-07 |
| b2844 | 1182 | 0,54 | 2,316770829 | 7,035790636 | 32,45226715 | 1,22156E-08 | 2,95949E-07 |
| b3942 | 2181 | 0,56 | -2,058532447 | 11,06026994 | 32,43440129 | 1,23285E-08 | 2,97076E-07 |
| b1830 | 2049 | 0,5 | 1,440147974 | 8,574491489 | 32,41536533 | 1,24498E-08 | 2,98396E-07 |
| b1647 | 897 | 0,52 | -1,28419278 | 8,026820084 | 32,4006236 | 1,25446E-08 | 2,99069E-07 |
| b1475 | 885 | 0,55 | -3,873516484 | 7,066268928 | 32,35729895 | 1,28274E-08 | 3,04194E-07 |
| b0644 | 978 | 0,47 | -1,22956057 | 5,83774128 | 32,32006873 | 1,30756E-08 | 3,08254E-07 |
| b1440 | 1146 | 0,53 | -1,689354259 | 9,650793043 | 32,31107795 | 1,31362E-08 | 3,08254E-07 |
| b3186 | 312 | 0,48 | 2,289297138 | 8,918091964 | 32,28977951 | 1,3281E-08 | 3,09547E-07 |
| b3945 | 1104 | 0,55 | -3,309623324 | 8,067647234 | 32,2827085 | 1,33294E-08 | 3,09547E-07 |
| b2705 | 780 | 0,53 | -2,005163786 | 5,845050671 | 32,15356582 | 1,42455E-08 | 3,29115E-07 |
| b3962 | 1401 | 0,52 | 2,250379082 | 9,082424586 | 31,8873291 | 1,63379E-08 | 3,75521E-07 |
| b3352 | 1914 | 0,55 | 1,326137055 | 6,298319834 | 31,85936185 | 1,65749E-08 | 3,79024E-07 |
| b0733 | 1569 | 0,53 | -2,184600384 | 11,25813257 | 31,72353722 | 1,77755E-08 | 4,04415E-07 |
| b2205 | 696 | 0,59 | -3,275692926 | 7,421449079 | 31,67378939 | 1,82367E-08 | 4,12812E-07 |
| b1332 | 258 | 0,45 | 1,636181486 | 6,187902325 | 31,55422517 | 1,93948E-08 | 4,36242E-07 |
| b2955 | 1137 | 0,53 | 1,610505753 | 5,346420016 | 31,54010545 | 1,95363E-08 | 4,36242E-07 |
| b4207 | 621 | 0,56 | 1,799251549 | 7,153875046 | 31,53738607 | 1,95637E-08 | 4,36242E-07 |
| b3019 | 2259 | 0,55 | 1,084598405 | 7,553683376 | 31,52729188 | 1,96657E-08 | 4,36344E-07 |
| b3316 | 279 | 0,49 | 2,08074578 | 9,200286188 | 31,50509593 | 1,98918E-08 | 4,39186E-07 |
| b3783 | 1260 | 0,5 | 1,641910969 | 8,590115287 | 31,44126383 | 2,05565E-08 | 4,51639E-07 |
| b0523 | 510 | 0,59 | 2,762873952 | 5,729706917 | 31,24391555 | 2,2756E-08 | 4,97523E-07 |
| b2845 | 1230 | 0,44 | 1,725676841 | 4,907478263 | 31,12624463 | 2,4178E-08 | 5,26048E-07 |
| b3597 | 1137 | 0,53 | -1,757498429 | 6,644646724 | 31,1160108 | 2,43058E-08 | 5,26274E-07 |
| b3030 | 1893 | 0,54 | 1,160824643 | 7,188637413 | 31,0419535 | 2,52511E-08 | 5,44113E-07 |
| b2351 | 921 | 0,4 | 2,38654423 | 5,860537904 | 30,90717737 | 2,70669E-08 | 5,7939E-07 |
| b2817 | 1254 | 0,54 | 1,5135026 | 6,108927706 | 30,89820335 | 2,71923E-08 | 5,7939E-07 |
| b2628 | 1617 | 0,49 | -1,243374481 | 6,963830263 | 30,89109259 | 2,72922E-08 | 5,7939E-07 |
| b4334 | 768 | 0,57 | -2,346868932 | 4,984852343 | 30,88306166 | 2,74053E-08 | 5,7939E-07 |
| b1190 | 1071 | 0,58 | 2,368037846 | 9,291576727 | 30,6849709 | 3,03507E-08 | 6,38647E-07 |
| b0839 | 1203 | 0,52 | -1,309224418 | 9,25995311 | 30,59762172 | 3,17483E-08 | 6,64933E-07 |
| b2587 | 1299 | 0,49 | 2,283191928 | 9,213232976 | 30,58296137 | 3,19891E-08 | 6,66861E-07 |
| b3570 | 825 | 0,48 | 1,43979765 | 7,768565648 | 30,49187491 | 3,35268E-08 | 6,95681E-07 |
| b2529 | 387 | 0,5 | 1,8847532 | 7,043577302 | 30,43090202 | 3,45973E-08 | 7,14585E-07 |
| b1333 | 951 | 0,51 | -2,049418284 | 10,57055897 | 30,29986542 | 3,70152E-08 | 7,61018E-07 |
| b0722 | 348 | 0,51 | 3,192341682 | 8,300492119 | 30,26737489 | 3,76404E-08 | 7,7034E-07 |
| b2518 | 432 | 0,52 | 3,616347173 | 6,54545287 | 30,24015198 | 3,81725E-08 | 7,77677E-07 |
| b4049 | 993 | 0,54 | 1,441672313 | 4,941661399 | 30,2234386 | 3,85028E-08 | 7,77834E-07 |
| b1777 | 273 | 0,45 | 1,765655658 | 7,385226909 | 30,22220707 | 3,85273E-08 | 7,77834E-07 |
| b0337 | 1284 | 0,53 | 1,7657278 | 6,572983288 | 30,19708065 | 3,90297E-08 | 7,80647E-07 |
| b4073 | 957 | 0,58 | -5,288687271 | 6,315042471 | 30,19452791 | 3,90811E-08 | 7,80647E-07 |
| b1783 | 1935 | 0,5 | -1,907002864 | 11,86996311 | 30,18917292 | 3,91891E-08 | 7,80647E-07 |
| b1133 | 1107 | 0,54 | 1,420019939 | 6,780278353 | 30,1194193 | 4,06243E-08 | 8,05655E-07 |
| b1089 | 174 | 0,54 | 1,32643594 | 7,870029625 | 30,05116323 | 4,20796E-08 | 8,3084E-07 |
| b4138 | 1302 | 0,53 | -2,143737097 | 10,12486228 | 30,03875751 | 4,23497E-08 | 8,32505E-07 |
| b1716 | 357 | 0,48 | 1,466160472 | 10,00538121 | 30,00988257 | 4,2985E-08 | 8,35625E-07 |
| b1113 | 963 | 0,53 | 1,671289813 | 4,892593131 | 30,00711537 | 4,30464E-08 | 8,35625E-07 |

| | | | | | | | |
|---|---|---|---|---|---|---|---|
| b0643 | 483 | 0,53 | -1,582878674 | 10,08212218 | 30,00615521 | 4,30677E-08 | 8,35625E-07 |
| b3091 | 1488 | 0,54 | -1,677694295 | 6,244114845 | 29,94176815 | 4,45218E-08 | 8,58105E-07 |
| b0194 | 1719 | 0,55 | 1,420121732 | 9,035058128 | 29,93796474 | 4,46092E-08 | 8,58105E-07 |
| b3003 | 885 | 0,54 | -1,709765525 | 7,91530377 | 29,92680319 | 4,48668E-08 | 8,59371E-07 |
| b1055 | 1053 | 0,5 | 2,168235324 | 4,973868363 | 29,85483057 | 4,65635E-08 | 8,88075E-07 |
| b1126 | 1137 | 0,51 | 1,229740977 | 6,096273032 | 29,84369492 | 4,68317E-08 | 8,89406E-07 |
| b2608 | 549 | 0,49 | 1,870898258 | 10,18819593 | 29,67809879 | 5,10079E-08 | 9,6463E-07 |
| b2310 | 783 | 0,5 | 2,176386285 | 6,806004984 | 29,65564451 | 5,16022E-08 | 9,71768E-07 |
| b0776 | 1155 | 0,58 | 2,426481921 | 4,266219353 | 29,57598155 | 5,37671E-08 | 1,00823E-06 |
| b3735 | 534 | 0,52 | 2,194878174 | 9,653149457 | 29,56802813 | 5,39882E-08 | 1,00823E-06 |
| b3315 | 333 | 0,5 | 2,020918965 | 10,02346205 | 29,4914449 | 5,61641E-08 | 1,04451E-06 |
| b3169 | 1488 | 0,53 | 1,201715669 | 9,068459937 | 29,47821134 | 5,65489E-08 | 1,04732E-06 |
| b4070 | 1437 | 0,52 | -4,696362402 | 9,25510194 | 29,4636196 | 5,69762E-08 | 1,05089E-06 |
| b3860 | 627 | 0,48 | 1,368595805 | 7,846279785 | 29,44651721 | 5,74812E-08 | 1,05308E-06 |
| b2746 | 480 | 0,54 | 1,289607966 | 5,865721371 | 29,443708 | 5,75646E-08 | 1,05308E-06 |
| b3118 | 939 | 0,42 | -4,020146815 | 8,719753546 | 29,38697213 | 5,92748E-08 | 1,07996E-06 |
| b3668 | 1503 | 0,57 | 1,656800105 | 4,109106644 | 29,35782165 | 6,01732E-08 | 1,09189E-06 |
| b3741 | 1890 | 0,54 | 1,126604308 | 6,852014125 | 29,32549347 | 6,11854E-08 | 1,10458E-06 |
| b2204 | 864 | 0,56 | -3,230129441 | 6,922008258 | 29,31978827 | 6,13658E-08 | 1,10458E-06 |
| b4349 | 1590 | 0,55 | 1,348764737 | 6,206489917 | 29,23528114 | 6,41013E-08 | 1,14921E-06 |
| b3297 | 390 | 0,52 | 1,688247151 | 10,24836835 | 29,16251953 | 6,65543E-08 | 1,18843E-06 |
| b0978 | 1545 | 0,54 | 1,65324365 | 6,975775614 | 29,12241716 | 6,79462E-08 | 1,20584E-06 |
| b3317 | 822 | 0,53 | 2,077949789 | 11,1427602 | 29,10758374 | 6,84684E-08 | 1,20584E-06 |
| b2557 | 3888 | 0,57 | 1,977194269 | 9,926261831 | 29,10028345 | 6,87269E-08 | 1,20584E-06 |
| b2292 | 1833 | 0,52 | -2,084463816 | 7,400577491 | 29,09862576 | 6,87858E-08 | 1,20584E-06 |
| b3745 | 1452 | 0,53 | -1,40204283 | 6,826015355 | 29,09612976 | 6,88744E-08 | 1,20584E-06 |
| b2008 | 1059 | 0,51 | -1,021199089 | 6,488195362 | 28,92617655 | 7,51899E-08 | 1,31129E-06 |
| b0774 | 1290 | 0,56 | 2,35592396 | 3,958651447 | 28,89426978 | 7,64387E-08 | 1,3279E-06 |
| b0895 | 618 | 0,52 | -3,816478429 | 6,190401811 | 28,85070497 | 7,81774E-08 | 1,35286E-06 |
| b3528 | 1287 | 0,52 | 2,71077447 | 9,917292249 | 28,8231914 | 7,92959E-08 | 1,36694E-06 |
| b4490 | 830 | 0,54 | 3,289641487 | 2,722542714 | 28,67439317 | 8,56285E-08 | 1,47045E-06 |
| b3846 | 2190 | 0,55 | 3,747103648 | 7,29112466 | 28,62415515 | 8,78791E-08 | 1,49425E-06 |
| b2511 | 1473 | 0,52 | 1,035343331 | 7,851864151 | 28,61899645 | 8,81135E-08 | 1,49425E-06 |
| b0721 | 390 | 0,49 | 3,536381219 | 7,908558539 | 28,61405193 | 8,83388E-08 | 1,49425E-06 |
| b3294 | 384 | 0,54 | 1,755720339 | 9,485771675 | 28,61385397 | 8,83478E-08 | 1,49425E-06 |
| b3636 | 168 | 0,4 | 2,164699006 | 6,658702215 | 28,49189968 | 9,40913E-08 | 1,5854E-06 |
| b3516 | 825 | 0,41 | -1,333497441 | 9,675041832 | 28,47659617 | 9,4838E-08 | 1,58652E-06 |
| b0168 | 795 | 0,5 | 1,279538726 | 6,840221308 | 28,47603368 | 9,48655E-08 | 1,58652E-06 |
| b1138 | 705 | 0,31 | -1,42777522 | 4,130473287 | 28,45266945 | 9,60173E-08 | 1,59981E-06 |
| b0030 | 915 | 0,56 | -1,583065149 | 7,534791372 | 28,37717167 | 9,98356E-08 | 1,65727E-06 |
| b1003 | 228 | 0,47 | -1,751494649 | 9,99263474 | 28,35809902 | 1,00824E-07 | 1,6675E-06 |
| b3787 | 1263 | 0,54 | 1,274729127 | 6,303865413 | 28,3177387 | 1,02948E-07 | 1,69638E-06 |
| b1535 | 891 | 0,45 | 1,992368681 | 4,073886944 | 28,28212689 | 1,0486E-07 | 1,72154E-06 |
| b4291 | 2325 | 0,59 | 2,636498337 | 5,455548097 | 28,26351373 | 1,05873E-07 | 1,73183E-06 |
| b2997 | 1119 | 0,54 | -3,069877585 | 9,002792567 | 28,25041429 | 1,06592E-07 | 1,73725E-06 |
| b1739 | 339 | 0,5 | -1,549052626 | 10,66813495 | 28,23810814 | 1,07272E-07 | 1,742E-06 |
| b4128 | 297 | 0,35 | -2,006019253 | 4,193345582 | 28,22302807 | 1,08111E-07 | 1,74929E-06 |
| b1004 | 597 | 0,54 | -1,787651841 | 11,24557193 | 28,12942593 | 1,13467E-07 | 1,82936E-06 |
| b1587 | 2427 | 0,51 | -2,505468638 | 6,352536904 | 28,0725878 | 1,16849E-07 | 1,87713E-06 |
| b1178 | 402 | 0,43 | -1,532088776 | 5,17587848 | 28,01447344 | 1,20411E-07 | 1,92744E-06 |
| b3306 | 393 | 0,5 | 1,851475897 | 9,873600233 | 27,99758624 | 1,21467E-07 | 1,93742E-06 |
| b0312 | 1473 | 0,58 | 1,69804381 | 6,489104126 | 27,98555294 | 1,22225E-07 | 1,94259E-06 |
| b3161 | 1245 | 0,53 | -6,435021452 | 10,05184537 | 27,96050352 | 1,23817E-07 | 1,96095E-06 |
| b3946 | 663 | 0,53 | -1,747663806 | 4,142903773 | 27,93309254 | 1,25583E-07 | 1,97516E-06 |
| b2569 | 1800 | 0,54 | 1,248303059 | 8,113333582 | 27,93290651 | 1,25596E-07 | 1,97516E-06 |
| b3588 | 1539 | 0,55 | -1,656343948 | 10,06233491 | 27,75454101 | 1,37725E-07 | 2,15833E-06 |
| b4391 | 1668 | 0,55 | 1,404038269 | 9,38223288 | 27,50125647 | 1,56992E-07 | 2,45171E-06 |
| b0346 | 834 | 0,56 | 2,443140092 | 5,960138549 | 27,46825263 | 1,59695E-07 | 2,48525E-06 |
| b1955 | 816 | 0,52 | -1,339889799 | 7,545693676 | 27,39354678 | 1,65984E-07 | 2,57419E-06 |
| b0431 | 1992 | 0,53 | 2,584776986 | 11,11239408 | 27,35936485 | 1,68944E-07 | 2,61106E-06 |
| b0896 | 864 | 0,55 | -2,945282181 | 6,493276067 | 27,3277289 | 1,71731E-07 | 2,64501E-06 |
| b0420 | 1863 | 0,54 | 1,161246366 | 7,568863326 | 27,27721509 | 1,76276E-07 | 2,70572E-06 |
| b4004 | 1326 | 0,56 | -1,327041295 | 6,307630517 | 27,23881067 | 1,79813E-07 | 2,75058E-06 |
| b3308 | 540 | 0,5 | 1,893729005 | 10,82953774 | 27,20311656 | 1,83163E-07 | 2,7923E-06 |
| b2795 | 1365 | 0,51 | -1,271409616 | 9,465255531 | 27,19500251 | 1,83934E-07 | 2,79454E-06 |
| b4317 | 2637 | 0,49 | 1,597240733 | 5,056008374 | 27,18397302 | 1,84986E-07 | 2,80104E-06 |
| b0823 | 2433 | 0,55 | -2,2722663 | 5,386326625 | 27,14713551 | 1,88544E-07 | 2,84531E-06 |
| b0734 | 1140 | 0,54 | -2,182984112 | 10,49751586 | 27,12338718 | 1,90875E-07 | 2,87081E-06 |
| b1967 | 852 | 0,49 | -1,630840654 | 10,42949258 | 27,09664878 | 1,93533E-07 | 2,90106E-06 |
| b2678 | 1065 | 0,58 | 1,846733455 | 4,736133025 | 27,07965393 | 1,95242E-07 | 2,9084E-06 |
| b3230 | 393 | 0,52 | 1,797991451 | 9,732217512 | 27,07887157 | 1,95321E-07 | 2,9084E-06 |
| b1283 | 219 | 0,52 | -1,97793068 | 8,105232783 | 27,05006635 | 1,98253E-07 | 2,94229E-06 |
| b0406 | 1128 | 0,51 | 1,243321295 | 7,177553495 | 26,98342047 | 2,05208E-07 | 3,03545E-06 |
| b3310 | 372 | 0,51 | 1,730557557 | 10,2927366 | 26,95527114 | 2,08219E-07 | 3,06608E-06 |
| b1212 | 834 | 0,55 | 1,433965852 | 4,592791447 | 26,95129929 | 2,08647E-07 | 3,06608E-06 |
| b2717 | 471 | 0,54 | -1,418905092 | 5,106684334 | 26,92115913 | 2,11926E-07 | 3,10409E-06 |
| b0919 | 894 | 0,52 | -3,16392026 | 9,084760392 | 26,81974215 | 2,23344E-07 | 3,26068E-06 |
| b2677 | 1203 | 0,49 | 1,868008224 | 4,760179395 | 26,71860424 | 2,35345E-07 | 3,42472E-06 |

| | | | | | | | |
|---|---|---|---|---|---|---|---|
| b1906 | 240 | 0,47 | -1,813424888 | 5,613282995 | 26,6697346 | 2,41373E-07 | 3,50108E-06 |
| b4133 | 1539 | 0,42 | -1,929780009 | 4,341107731 | 26,63847907 | 2,45309E-07 | 3,5467E-06 |
| b2704 | 372 | 0,55 | -1,792331784 | 3,956364538 | 26,56674733 | 2,54588E-07 | 3,66266E-06 |
| b1784 | 1284 | 0,49 | -1,76801917 | 9,529170872 | 26,56389269 | 2,54965E-07 | 3,66266E-06 |
| b1849 | 1179 | 0,54 | 2,458865821 | 7,305481507 | 26,55297776 | 2,56409E-07 | 3,67165E-06 |
| b3351 | 555 | 0,57 | 1,884417123 | 4,108621144 | 26,52793224 | 2,59755E-07 | 3,70771E-06 |
| b3261 | 297 | 0,47 | 2,204807761 | 5,195153256 | 26,49858846 | 2,63731E-07 | 3,75251E-06 |
| b1276 | 2676 | 0,53 | -1,554897395 | 11,52955077 | 26,4917733 | 2,64663E-07 | 3,75386E-06 |
| b2513 | 621 | 0,5 | 1,03391041 | 7,24744341 | 26,47987459 | 2,66298E-07 | 3,76514E-06 |
| b2530 | 1215 | 0,53 | 2,42310106 | 8,382458135 | 26,41906453 | 2,74815E-07 | 3,87333E-06 |
| b2139 | 1436 | 0,55 | 3,45331784 | 1,785110323 | 26,40504701 | 2,76816E-07 | 3,88829E-06 |
| b2231 | 2628 | 0,54 | 1,419646061 | 9,357255585 | 26,39950844 | 2,77611E-07 | 3,88829E-06 |
| b4303 | 807 | 0,53 | -1,709917685 | 4,839723332 | 26,37014298 | 2,81864E-07 | 3,93556E-06 |
| b3296 | 621 | 0,51 | 1,677055785 | 11,11068219 | 26,35219049 | 2,84496E-07 | 3,95726E-06 |
| b1406 | 861 | 0,53 | -0,97393286 | 7,100344188 | 26,34752514 | 2,85184E-07 | 3,95726E-06 |
| b1188 | 1533 | 0,5 | -1,904085012 | 9,816139804 | 26,23311062 | 3,0259E-07 | 4,18582E-06 |
| b3530 | 3474 | 0,57 | -1,068796771 | 7,200495024 | 26,21187371 | 3,05936E-07 | 4,21909E-06 |
| b1673 | 2103 | 0,48 | -2,581787064 | 5,493628364 | 26,19860857 | 3,08044E-07 | 4,23514E-06 |
| b2330 | 933 | 0,53 | 1,30756311 | 6,817333176 | 26,18811657 | 3,09723E-07 | 4,2405E-06 |
| b0104 | 1044 | 0,51 | 1,422248567 | 7,421266832 | 26,18435457 | 3,10327E-07 | 4,2405E-06 |
| b1717 | 198 | 0,5 | 1,331609411 | 8,838531481 | 26,13735811 | 3,17972E-07 | 4,33177E-06 |
| b4131 | 2148 | 0,48 | -2,947978151 | 5,771522838 | 26,12674964 | 3,19724E-07 | 4,34243E-06 |
| b2531 | 489 | 0,54 | 2,547985342 | 5,9282479 | 26,10862471 | 3,22739E-07 | 4,37014E-06 |
| b2153 | 669 | 0,51 | 1,405257055 | 6,594870686 | 26,04842778 | 3,3296E-07 | 4,49496E-06 |
| b1768 | 642 | 0,53 | 1,228015038 | 5,503525869 | 25,92680694 | 3,5461E-07 | 4,77286E-06 |
| b3738 | 816 | 0,5 | 1,865437567 | 9,063115932 | 25,91043073 | 3,57631E-07 | 4,79911E-06 |
| b2143 | 885 | 0,55 | -1,22689696 | 6,960661553 | 25,86307714 | 3,66513E-07 | 4,90362E-06 |
| b1656 | 582 | 0,5 | -1,533102663 | 11,02072294 | 25,84061458 | 3,70803E-07 | 4,94625E-06 |
| b2028 | 1167 | 0,44 | 1,483440321 | 4,270596614 | 25,82950866 | 3,72943E-07 | 4,95646E-06 |
| b4014 | 1602 | 0,52 | 1,942225301 | 12,90858033 | 25,82473316 | 3,73867E-07 | 4,95646E-06 |
| b0463 | 1194 | 0,54 | 1,088585336 | 7,926467069 | 25,81947799 | 3,74886E-07 | 4,95646E-06 |
| b2902 | 744 | 0,55 | 1,128484596 | 5,853007795 | 25,75961616 | 3,86696E-07 | 5,09756E-06 |
| b2891 | 1099 | 0,54 | 1,446198263 | 8,022579897 | 25,74951602 | 3,88724E-07 | 5,10928E-06 |
| b2609 | 249 | 0,51 | 1,588366861 | 8,139814507 | 25,70026786 | 3,98772E-07 | 5,22601E-06 |
| b4242 | 2697 | 0,52 | 1,392017629 | 6,487107628 | 25,60877536 | 4,18134E-07 | 5,46377E-06 |
| b3305 | 534 | 0,52 | 1,93381188 | 10,68127636 | 25,60175595 | 4,19657E-07 | 5,46774E-06 |
| b0120 | 795 | 0,46 | 1,242204117 | 6,408882683 | 25,58380482 | 4,2358E-07 | 5,50285E-06 |
| b2799 | 1149 | 0,54 | -1,435312993 | 7,069822349 | 25,5533164 | 4,30326E-07 | 5,57434E-06 |
| b2365 | 1338 | 0,49 | -1,914311916 | 4,598413147 | 25,47738386 | 4,47599E-07 | 5,78138E-06 |
| b3424 | 831 | 0,54 | 1,631852103 | 5,198931031 | 25,43326424 | 4,57953E-07 | 5,89812E-06 |
| b3493 | 1500 | 0,54 | 0,997843392 | 7,889609972 | 25,41781849 | 4,61634E-07 | 5,9285E-06 |
| b1476 | 654 | 0,5 | -3,106160057 | 6,261069992 | 25,35591588 | 4,76688E-07 | 6,10433E-06 |
| b2995 | 1179 | 0,54 | -2,67888381 | 7,480370954 | 25,3216986 | 4,8522E-07 | 6,19588E-06 |
| b2878 | 3099 | 0,52 | -1,311812847 | 4,869090792 | 25,29779313 | 4,91271E-07 | 6,25533E-06 |
| b4122 | 1647 | 0,55 | -2,287909986 | 6,098913732 | 25,20445826 | 5,15629E-07 | 6,54688E-06 |
| b2398 | 345 | 0,5 | -1,29421586 | 6,28006593 | 25,17864626 | 5,22576E-07 | 6,61635E-06 |
| b3005 | 426 | 0,5 | 2,111782871 | 4,384621835 | 25,17147574 | 5,24523E-07 | 6,62229E-06 |
| b0334 | 1452 | 0,57 | 2,139092712 | 3,331289605 | 25,16137383 | 5,27278E-07 | 6,63837E-06 |
| b2957 | 1047 | 0,53 | -4,261580893 | 10,93586382 | 25,11812681 | 5,39236E-07 | 6,75912E-06 |
| b4147 | 567 | 0,49 | 1,469134397 | 7,991899121 | 25,11580752 | 5,39885E-07 | 6,75912E-06 |
| b0062 | 1503 | 0,55 | -1,702922685 | 5,787555299 | 25,0614623 | 5,55317E-07 | 6,93295E-06 |
| b0781 | 990 | 0,54 | -1,851140081 | 8,244808785 | 24,99666255 | 5,74296E-07 | 7,14999E-06 |
| b2709 | 1515 | 0,57 | -1,35944841 | 6,323114858 | 24,90647804 | 6,01799E-07 | 7,46546E-06 |
| b2827 | 795 | 0,51 | 1,115758324 | 6,441329161 | 24,90273954 | 6,02967E-07 | 7,46546E-06 |
| b4307 | 546 | 0,55 | 1,500503211 | 4,482530021 | 24,88347272 | 6,09023E-07 | 7,51967E-06 |
| b0330 | 1587 | 0,55 | 2,087922698 | 5,363069507 | 24,87428302 | 6,11933E-07 | 7,53351E-06 |
| b3170 | 453 | 0,49 | 1,30938011 | 6,333930228 | 24,86933641 | 6,13506E-07 | 7,53351E-06 |
| b2520 | 4962 | 0,53 | -1,251669931 | 9,485637294 | 24,84765699 | 6,20444E-07 | 7,5979E-06 |
| b3386 | 678 | 0,51 | 0,995152263 | 7,287036529 | 24,81286608 | 6,31744E-07 | 7,71519E-06 |
| b2712 | 2253 | 0,58 | -1,387001697 | 5,73112296 | 24,80396748 | 6,34667E-07 | 7,72983E-06 |
| b3093 | 1419 | 0,54 | -1,12838182 | 5,888102213 | 24,79642112 | 6,37157E-07 | 7,73912E-06 |
| b3640 | 456 | 0,55 | 1,184808748 | 5,962459481 | 24,75996113 | 6,49324E-07 | 7,86559E-06 |
| b3211 | 930 | 0,56 | -1,961081233 | 5,108362369 | 24,75401252 | 6,51331E-07 | 7,86864E-06 |
| b3295 | 990 | 0,52 | 1,685612905 | 11,73014565 | 24,56536449 | 7,18313E-07 | 8,65451E-06 |
| b1452 | 1062 | 0,51 | 3,130581578 | 4,811431288 | 24,54736302 | 7,25055E-07 | 8,71232E-06 |
| b2607 | 768 | 0,53 | 1,602763891 | 10,37115226 | 24,52184825 | 7,3472E-07 | 8,80485E-06 |
| b3231 | 429 | 0,51 | 1,746546148 | 9,583296903 | 24,43865722 | 7,67138E-07 | 9,16884E-06 |
| b2528 | 324 | 0,52 | 1,592655944 | 7,160781602 | 24,43317327 | 7,69325E-07 | 9,17052E-06 |
| b0799 | 2151 | 0,54 | -1,020913382 | 6,085562384 | 24,39586058 | 7,84371E-07 | 9,30032E-06 |
| b2220 | 1386 | 0,49 | -1,729030915 | 5,892710515 | 24,39095143 | 7,86372E-07 | 9,30032E-06 |
| b3492 | 1203 | 0,55 | -1,589012018 | 6,78764042 | 24,39078613 | 7,86439E-07 | 9,30032E-06 |
| b0949 | 1908 | 0,52 | 0,989949448 | 7,176753301 | 24,34018588 | 8,0737E-07 | 9,51851E-06 |
| b0652 | 726 | 0,52 | 1,546776484 | 6,160307778 | 24,33597456 | 8,09137E-07 | 9,51851E-06 |
| b1225 | 1539 | 0,54 | -4,41324081 | 10,86895997 | 24,32498825 | 8,13765E-07 | 9,5479E-06 |
| b2272 | 504 | 0,46 | -2,14546424 | 3,606712008 | 24,20297844 | 8,66981E-07 | 1,01457E-05 |
| b2393 | 1203 | 0,5 | 1,419841792 | 7,008578388 | 24,14631941 | 8,92867E-07 | 1,04214E-05 |
| b1117 | 702 | 0,55 | 1,424599127 | 5,487708165 | 24,13375745 | 8,9871E-07 | 1,04624E-05 |
| b1451 | 2103 | 0,51 | 1,879790553 | 4,682077249 | 24,11955063 | 9,05364E-07 | 1,05125E-05 |

| | | | | | | | |
|---|---|---|---|---|---|---|---|
| b3565 | 1323 | 0,51 | -1,048358295 | 6,32388892 | 24,0761135 | 9,26019E-07 | 1,07246E-05 |
| b2190 | 3791 | 0,48 | -0,903600335 | 6,069227265 | 24,06930119 | 9,29301E-07 | 1,07349E-05 |
| b3423 | 758 | 0,55 | 1,363591723 | 5,309962745 | 23,90605138 | 1,01153E-06 | 1,16536E-05 |
| b3314 | 702 | 0,51 | 1,778943943 | 11,16555484 | 23,89758739 | 1,01599E-06 | 1,16536E-05 |
| b4123 | 1341 | 0,52 | -2,24662576 | 5,75309337 | 23,89637228 | 1,01663E-06 | 1,16536E-05 |
| b2366 | 1329 | 0,51 | -1,196530329 | 5,535746031 | 23,88114901 | 1,0247E-06 | 1,17161E-05 |
| b1285 | 1986 | 0,48 | -1,056390238 | 8,360885706 | 23,79151028 | 1,07355E-06 | 1,22434E-05 |
| b3831 | 762 | 0,54 | -1,37945101 | 9,626487273 | 23,77589827 | 1,08229E-06 | 1,23118E-05 |
| b1748 | 1221 | 0,55 | 3,244361333 | 7,621265365 | 23,75053973 | 1,09665E-06 | 1,24435E-05 |
| b1786 | 1491 | 0,46 | 1,224365232 | 5,140640325 | 23,69444234 | 1,12908E-06 | 1,27791E-05 |
| b2329 | 1086 | 0,57 | 1,133579895 | 7,019246214 | 23,65999946 | 1,14947E-06 | 1,29771E-05 |
| b0812 | 504 | 0,49 | -1,503708868 | 12,74911534 | 23,62362515 | 1,1714E-06 | 1,31915E-05 |
| b3788 | 1068 | 0,54 | 0,982654229 | 6,26999209 | 23,61753587 | 1,17512E-06 | 1,32002E-05 |
| b1678 | 1005 | 0,52 | -1,784012106 | 9,384082738 | 23,5260704 | 1,23233E-06 | 1,38082E-05 |
| b0800 | 963 | 0,56 | -1,249218454 | 8,113554845 | 23,51245274 | 1,24108E-06 | 1,38716E-05 |
| b3309 | 315 | 0,46 | 1,749616815 | 9,599309475 | 23,4905931 | 1,25526E-06 | 1,39952E-05 |
| b3564 | 1455 | 0,55 | -1,077262705 | 6,415012637 | 23,45334307 | 1,2798E-06 | 1,42134E-05 |
| b2606 | 348 | 0,48 | 1,493944585 | 9,199663772 | 23,45128897 | 1,28117E-06 | 1,42134E-05 |
| b1269 | 876 | 0,54 | 0,988029623 | 6,089922056 | 23,40368055 | 1,31327E-06 | 1,45335E-05 |
| b3362 | 168 | 0,51 | -1,686350415 | 6,140895203 | 23,36431855 | 1,34042E-06 | 1,47974E-05 |
| b2510 | 216 | 0,48 | 1,669900483 | 3,566331063 | 23,32171537 | 1,37044E-06 | 1,50917E-05 |
| b3366 | 327 | 0,56 | -3,400075886 | 5,150401041 | 23,31482132 | 1,37536E-06 | 1,51087E-05 |
| b4315 | 540 | 0,49 | 1,636765362 | 4,967379356 | 23,27745333 | 1,40234E-06 | 1,53675E-05 |
| b1631 | 621 | 0,53 | 1,631602186 | 4,444759714 | 23,27110698 | 1,40698E-06 | 1,537E-05 |
| b0099 | 390 | 0,49 | 2,230323745 | 2,963496776 | 23,26707817 | 1,40993E-06 | 1,537E-05 |
| b2702 | 564 | 0,5 | -1,939410039 | 4,517453231 | 23,26308313 | 1,41286E-06 | 1,537E-05 |
| b0925 | 1848 | 0,53 | -1,328504307 | 8,809016518 | 23,20177681 | 1,45862E-06 | 1,58294E-05 |
| b3669 | 591 | 0,59 | 1,677573781 | 5,002337023 | 23,11787794 | 1,52367E-06 | 1,64954E-05 |
| b2526 | 1851 | 0,58 | 1,261814218 | 7,459026338 | 23,10504748 | 1,53388E-06 | 1,65659E-05 |
| b0970 | 660 | 0,51 | 1,37652501 | 8,419576871 | 23,06863676 | 1,5632E-06 | 1,6842E-05 |
| b0381 | 1095 | 0,51 | 1,14478008 | 7,414030957 | 23,04305015 | 1,58414E-06 | 1,70267E-05 |
| b0409 | 972 | 0,52 | 0,972739612 | 7,765866449 | 23,01633424 | 1,60631E-06 | 1,72236E-05 |
| b0469 | 552 | 0,54 | 2,13607488 | 5,594901691 | 22,97278092 | 1,64311E-06 | 1,75762E-05 |
| b0171 | 726 | 0,53 | 1,100809483 | 7,24428175 | 22,93320846 | 1,67729E-06 | 1,78991E-05 |
| b3024 | 393 | 0,52 | -1,37397233 | 10,117277 | 22,92657107 | 1,68309E-06 | 1,79183E-05 |
| b0067 | 1611 | 0,58 | 1,810915445 | 3,503672272 | 22,91581227 | 1,69254E-06 | 1,79762E-05 |
| b1046 | 1422 | 0,51 | -1,065267992 | 6,776727552 | 22,86226373 | 1,74035E-06 | 1,84403E-05 |
| b2287 | 663 | 0,52 | 1,285458173 | 9,035000692 | 22,85703349 | 1,7451E-06 | 1,8447E-05 |
| b1533 | 900 | 0,5 | 1,441204846 | 5,268910407 | 22,76894154 | 1,82695E-06 | 1,92667E-05 |
| b3159 | 879 | 0,56 | -1,481614538 | 4,605559872 | 22,75921188 | 1,83622E-06 | 1,93075E-05 |
| b2666 | 159 | 0,51 | -1,424852403 | 6,236361312 | 22,75443169 | 1,84079E-06 | 1,93075E-05 |
| b4116 | 762 | 0,41 | -3,342003236 | 4,546750209 | 22,75135973 | 1,84374E-06 | 1,93075E-05 |
| b3360 | 564 | 0,53 | -1,595210643 | 5,62558562 | 22,72148567 | 1,87262E-06 | 1,95643E-05 |
| b1747 | 1035 | 0,56 | 2,692549637 | 7,311079116 | 22,71084267 | 1,88302E-06 | 1,9604E-05 |
| b3414 | 576 | 0,53 | 1,60629242 | 6,701662315 | 22,70865075 | 1,88517E-06 | 1,9604E-05 |
| b1068 | 924 | 0,55 | -1,21777731 | 7,22327815 | 22,69668339 | 1,89695E-06 | 1,96809E-05 |
| b0641 | 582 | 0,52 | 0,964420123 | 7,666618955 | 22,68513747 | 1,90838E-06 | 1,97537E-05 |
| b2899 | 660 | 0,52 | -1,454120912 | 7,085626428 | 22,67180952 | 1,92167E-06 | 1,98454E-05 |
| b1211 | 1083 | 0,53 | 1,186537585 | 6,014281494 | 22,62738069 | 1,96662E-06 | 2,0263E-05 |
| b0142 | 480 | 0,5 | 2,129021708 | 3,754250785 | 22,60495591 | 1,98971E-06 | 2,04538E-05 |
| b4348 | 1395 | 0,4 | 1,706599941 | 4,783273482 | 22,59597964 | 1,99902E-06 | 2,05026E-05 |
| b4393 | 327 | 0,54 | -1,510489389 | 5,850110681 | 22,53517573 | 2,06331E-06 | 2,11136E-05 |
| b3591 | 1392 | 0,56 | -1,208188788 | 7,125533911 | 22,50087029 | 2,10048E-06 | 2,1445E-05 |
| b0954 | 519 | 0,53 | 1,330756438 | 8,210163325 | 22,49618742 | 2,10561E-06 | 2,14485E-05 |
| b4214 | 741 | 0,55 | -1,371269957 | 8,174657876 | 22,47690515 | 2,12685E-06 | 2,16158E-05 |
| b0315 | 1089 | 0,46 | 1,417816007 | 4,001043857 | 22,43054514 | 2,17881E-06 | 2,20937E-05 |
| b3299 | 117 | 0,48 | 1,399748875 | 7,793940715 | 22,40238275 | 2,211E-06 | 2,23695E-05 |
| b3165 | 270 | 0,52 | 1,335838066 | 8,275182773 | 22,34372291 | 2,27957E-06 | 2,30113E-05 |
| b1388 | 930 | 0,52 | 3,120944613 | 4,583842607 | 22,31119345 | 2,31851E-06 | 2,33518E-05 |
| b1749 | 807 | 0,53 | 1,518470442 | 5,767584829 | 22,29559145 | 2,33743E-06 | 2,34896E-05 |
| b0124 | 2391 | 0,55 | 1,922249548 | 7,705737167 | 22,27899505 | 2,35772E-06 | 2,36086E-05 |
| b0126 | 663 | 0,51 | 1,680585175 | 7,304582685 | 22,27729007 | 2,35981E-06 | 2,36086E-05 |
| b3127 | 1335 | 0,51 | -3,652906137 | 11,00322504 | 22,24367642 | 2,40148E-06 | 2,3972E-05 |
| b2964 | 1257 | 0,5 | -1,320254123 | 7,378930151 | 22,21095071 | 2,44277E-06 | 2,43299E-05 |
| b0476 | 960 | 0,52 | -1,083749241 | 4,950235024 | 22,17993142 | 2,48255E-06 | 2,46714E-05 |
| b1488 | 582 | 0,53 | 3,257541441 | 3,161026761 | 22,17482279 | 2,48917E-06 | 2,46824E-05 |
| b3448 | 441 | 0,51 | -1,695846723 | 7,89318194 | 22,17015882 | 2,49522E-06 | 2,46878E-05 |
| b2038 | 558 | 0,39 | 1,235597742 | 6,014295591 | 22,13379132 | 2,54294E-06 | 2,51045E-05 |
| b2552 | 1191 | 0,52 | -2,131121753 | 7,839493807 | 22,08492039 | 2,60851E-06 | 2,56952E-05 |
| b3700 | 1074 | 0,55 | 1,494270783 | 4,821979298 | 22,08062522 | 2,61435E-06 | 2,56963E-05 |
| b3298 | 357 | 0,52 | 1,438762412 | 10,27997973 | 22,01081516 | 2,71118E-06 | 2,65898E-05 |
| b4154 | 1809 | 0,56 | -1,830298737 | 11,25347464 | 21,90124072 | 2,87048E-06 | 2,80906E-05 |
| b3939 | 1161 | 0,55 | 1,289952707 | 5,29698621 | 21,79407681 | 3,03536E-06 | 2,96394E-05 |
| b2328 | 825 | 0,55 | 1,159255821 | 5,456465134 | 21,73550317 | 3,12946E-06 | 3,04912E-05 |
| b0304 | 1326 | 0,46 | -0,99796657 | 6,040018317 | 21,73137735 | 3,13619E-06 | 3,04912E-05 |
| b1060 | 255 | 0,45 | -2,210392911 | 8,032976987 | 21,56982454 | 3,41178E-06 | 3,30987E-05 |
| b2201 | 624 | 0,56 | -1,850695376 | 6,014738073 | 21,53377126 | 3,47652E-06 | 3,36539E-05 |
| b3365 | 2544 | 0,54 | -3,067977749 | 8,732288366 | 21,45523789 | 3,62185E-06 | 3,49075E-05 |

| | | | | | | | |
|---|---|---|---|---|---|---|---|
| b3070 | 765 | 0,56 | 2,15825054 | 3,441090975 | 21,45167704 | 3,62859E-06 | 3,49075E-05 |
| b4226 | 531 | 0,51 | 1,542825088 | 9,172735448 | 21,45125425 | 3,62939E-06 | 3,49075E-05 |
| b2286 | 1791 | 0,56 | 1,259230101 | 10,21265101 | 21,44686773 | 3,6377E-06 | 3,49126E-05 |
| b2673 | 246 | 0,53 | 2,988725084 | 2,227090059 | 21,43560569 | 3,65913E-06 | 3,4973E-05 |
| b1385 | 1500 | 0,53 | -1,0446154 | 6,104240947 | 21,43207214 | 3,66588E-06 | 3,4973E-05 |
| b2586 | 324 | 0,54 | 1,970637291 | 4,470158801 | 21,42857253 | 3,67257E-06 | 3,4973E-05 |
| b0634 | 1113 | 0,52 | 1,057042189 | 5,961413483 | 21,42719676 | 3,67521E-06 | 3,4973E-05 |
| b1015 | 1509 | 0,54 | 2,754190795 | 8,826965965 | 21,38653562 | 3,75398E-06 | 3,56469E-05 |
| b1224 | 3744 | 0,55 | -3,852707312 | 13,49970437 | 21,38205728 | 3,76276E-06 | 3,56547E-05 |
| b3832 | 1428 | 0,53 | -0,916420665 | 6,468863674 | 21,34431905 | 3,83756E-06 | 3,62868E-05 |
| b0911 | 1674 | 0,51 | 2,554601697 | 11,97811223 | 21,32625418 | 3,8739E-06 | 3,65533E-05 |
| b0818 | 1119 | 0,52 | 1,229380794 | 4,834237325 | 21,31006929 | 3,90674E-06 | 3,67857E-05 |
| b2905 | 1095 | 0,55 | 1,352084964 | 10,28186658 | 21,29168005 | 3,9444E-06 | 3,70625E-05 |
| b3572 | 1254 | 0,52 | 1,231670452 | 6,279284836 | 21,27184672 | 3,98542E-06 | 3,73696E-05 |
| b3637 | 237 | 0,53 | 1,683057325 | 8,344958938 | 21,23120037 | 4,07084E-06 | 3,80261E-05 |
| b3611 | 432 | 0,5 | 1,357946904 | 6,974946569 | 21,2304595 | 4,07241E-06 | 3,80261E-05 |
| b0029 | 951 | 0,55 | 0,95696686 | 6,838546525 | 21,20574947 | 4,12525E-06 | 3,84395E-05 |
| b0211 | 1359 | 0,52 | 1,111864221 | 8,184287229 | 21,19308974 | 4,15259E-06 | 3,86139E-05 |
| b3313 | 411 | 0,52 | 1,578390155 | 10,34880157 | 21,1644961 | 4,21501E-06 | 3,91132E-05 |
| b0931 | 1203 | 0,51 | 1,204599132 | 6,370927734 | 21,14050912 | 4,2681E-06 | 3,9524E-05 |
| b4414 | 136 | 0,51 | 1,412726029 | 6,931524077 | 21,10185103 | 4,35507E-06 | 4,02463E-05 |
| b3156 | 504 | 0,56 | -1,675695832 | 7,970172137 | 21,09706934 | 4,36595E-06 | 4,02638E-05 |
| b3906 | 849 | 0,5 | -1,252755373 | 4,034856432 | 21,07897405 | 4,40738E-06 | 4,05624E-05 |
| b3775 | 282 | 0,52 | 1,434378798 | 4,549243765 | 21,04228313 | 4,49259E-06 | 4,12618E-05 |
| b3012 | 828 | 0,51 | -1,508377459 | 9,199183325 | 21,01592941 | 4,55481E-06 | 4,17477E-05 |
| b2535 | 1281 | 0,52 | -1,534611617 | 10,14697179 | 20,96980951 | 4,66578E-06 | 4,26178E-05 |
| b2896 | 408 | 0,53 | -0,902154567 | 6,554752109 | 20,96859317 | 4,66875E-06 | 4,26178E-05 |
| b1127 | 1227 | 0,5 | -1,392102236 | 9,213342358 | 20,95913865 | 4,69184E-06 | 4,27415E-05 |
| b2411 | 2016 | 0,54 | 0,876209858 | 7,403665479 | 20,95135934 | 4,71093E-06 | 4,28284E-05 |
| b3566 | 993 | 0,44 | -1,337228971 | 5,675170221 | 20,93379965 | 4,75431E-06 | 4,31353E-05 |
| b3733 | 864 | 0,53 | 1,666151409 | 10,32184835 | 20,92933779 | 4,7654E-06 | 4,31485E-05 |
| b4523 | 168 | 0,46 | -1,454247197 | 5,550498541 | 20,84883759 | 4,96994E-06 | 4,49098E-05 |
| b1195 | 255 | 0,53 | -1,605486545 | 6,377778027 | 20,8217 | 5,04086E-06 | 4,5459E-05 |
| b3649 | 276 | 0,53 | 0,917639383 | 6,842774438 | 20,79882605 | 5,10142E-06 | 4,59128E-05 |
| b0593 | 1176 | 0,56 | 2,841324991 | 2,334972056 | 20,78045129 | 5,1506E-06 | 4,62625E-05 |
| b0738 | 429 | 0,52 | 1,474283009 | 4,365378757 | 20,76992362 | 5,179E-06 | 4,64245E-05 |
| b1342 | 984 | 0,52 | -0,986050772 | 7,336186982 | 20,73083937 | 5,28578E-06 | 4,72525E-05 |
| b3337 | 195 | 0,46 | 3,678281898 | 1,890298166 | 20,72740746 | 5,29526E-06 | 4,72525E-05 |
| b4381 | 780 | 0,55 | -1,310508934 | 7,726510007 | 20,72461338 | 5,303E-06 | 4,72525E-05 |
| b3267 | 222 | 0,54 | 2,586052395 | 2,710448455 | 20,71238841 | 5,33696E-06 | 4,74608E-05 |
| b3816 | 951 | 0,53 | 1,40753902 | 6,793819727 | 20,54097041 | 5,83685E-06 | 5,18035E-05 |
| b2965 | 2136 | 0,52 | 1,141239118 | 5,668554589 | 20,52078406 | 5,89873E-06 | 5,22492E-05 |
| b0855 | 1134 | 0,55 | 1,568930393 | 4,498490348 | 20,51221523 | 5,92519E-06 | 5,23801E-05 |
| b1586 | 306 | 0,56 | -1,587636281 | 6,023254687 | 20,4985784 | 5,96756E-06 | 5,26508E-05 |
| b0627 | 204 | 0,5 | 1,174534965 | 6,062006909 | 20,46839839 | 6,0624E-06 | 5,33824E-05 |
| b3115 | 1209 | 0,48 | -3,699620234 | 5,943170087 | 20,4460516 | 6,1336E-06 | 5,39035E-05 |
| b0999 | 306 | 0,54 | -1,414415957 | 7,934791916 | 20,44119904 | 6,14917E-06 | 5,39346E-05 |
| b3302 | 180 | 0,5 | 1,42142253 | 8,879153763 | 20,43340207 | 6,17427E-06 | 5,4049E-05 |
| b0529 | 867 | 0,54 | 1,053188936 | 6,823838257 | 20,41351124 | 6,23878E-06 | 5,45072E-05 |
| b1282 | 327 | 0,5 | 2,136372169 | 2,999222932 | 20,39307335 | 6,30576E-06 | 5,49853E-05 |
| b2936 | 759 | 0,52 | -1,226139098 | 8,220474876 | 20,36797411 | 6,38901E-06 | 5,5603E-05 |
| b1479 | 1698 | 0,52 | 1,182785832 | 8,45824785 | 20,34983392 | 6,44987E-06 | 5,60239E-05 |
| b3361 | 603 | 0,53 | -1,535253062 | 8,48175002 | 20,34198323 | 6,47639E-06 | 5,61454E-05 |
| b1019 | 1272 | 0,54 | 2,132442266 | 3,826881256 | 20,29433668 | 6,63968E-06 | 5,74499E-05 |
| b1630 | 1059 | 0,53 | 1,708874921 | 4,546522058 | 20,29055854 | 6,65281E-06 | 5,74526E-05 |
| b2838 | 1263 | 0,53 | 1,271717544 | 4,63750111 | 20,26465326 | 6,7435E-06 | 5,81238E-05 |
| b0313 | 588 | 0,57 | 1,826145429 | 5,286474074 | 20,2455804 | 6,81106E-06 | 5,85934E-05 |
| b3384 | 1005 | 0,52 | 0,963220808 | 8,234571429 | 20,18306218 | 7,03733E-06 | 6,0424E-05 |
| b3303 | 504 | 0,5 | 1,592496511 | 10,59264305 | 20,15712995 | 7,13339E-06 | 6,11316E-05 |
| b0758 | 1047 | 0,56 | -1,082617919 | 8,100562015 | 20,0837962 | 7,4122E-06 | 6,33998E-05 |
| b0146 | 705 | 0,49 | -1,077702808 | 8,491048799 | 20,06857946 | 7,47141E-06 | 6,37845E-05 |
| b2234 | 2286 | 0,54 | 1,468898541 | 8,2611288 | 20,05432673 | 7,5273E-06 | 6,41395E-05 |
| b0578 | 654 | 0,52 | 0,985387286 | 7,077614818 | 20,00444662 | 7,72623E-06 | 6,56749E-05 |
| b3734 | 1542 | 0,53 | 1,791620726 | 11,76746477 | 20,00183034 | 7,73681E-06 | 6,56749E-05 |
| b1124 | 795 | 0,5 | 1,256459976 | 5,397949121 | 19,97206898 | 7,85817E-06 | 6,6579E-05 |
| b1226 | 711 | 0,56 | -3,54375467 | 8,884629244 | 19,94797879 | 7,9578E-06 | 6,7296E-05 |
| b3347 | 813 | 0,48 | 1,351184189 | 7,675963842 | 19,94086164 | 7,98748E-06 | 6,74198E-05 |
| b4696 | 5323 | 0,51 | -0,841571839 | 6,865482377 | 19,93173611 | 8,0257E-06 | 6,7615E-05 |
| b4005 | 1290 | 0,58 | 2,185715549 | 8,304589952 | 19,91058339 | 8,11498E-06 | 6,8239E-05 |
| b0369 | 975 | 0,54 | 0,90131062 | 7,071747088 | 19,90114112 | 8,15516E-06 | 6,84484E-05 |
| b2399 | 393 | 0,46 | -1,263909599 | 5,471265461 | 19,84532966 | 8,39676E-06 | 7,03445E-05 |
| b1067 | 648 | 0,52 | -0,972804257 | 7,276516701 | 19,82731951 | 8,47625E-06 | 7,08779E-05 |
| b1380 | 990 | 0,5 | -1,322571179 | 8,091781446 | 19,75123683 | 8,82046E-06 | 7,36188E-05 |
| b0619 | 1659 | 0,5 | -1,209832582 | 5,582351588 | 19,69858937 | 9,06683E-06 | 7,55344E-05 |
| b1636 | 864 | 0,53 | 1,008377804 | 5,770090698 | 19,69244818 | 9,09601E-06 | 7,5637E-05 |
| b2536 | 1140 | 0,56 | -1,253111507 | 6,382301391 | 19,68030801 | 9,15398E-06 | 7,59781E-05 |
| b1418 | 531 | 0,48 | 1,238751296 | 5,360056023 | 19,65042134 | 9,29828E-06 | 7,70331E-05 |
| b4302 | 432 | 0,54 | -1,469316547 | 3,674238804 | 19,60472598 | 9,52333E-06 | 7,8752E-05 |

| | | | | | | | |
|---|---|---|---|---|---|---|---|
| b1125 | 828 | 0,51 | 1,293845268 | 5,053788205 | 19,60117304 | 9,54106E-06 | 7,87533E-05 |
| b0822 | 816 | 0,47 | 1,701787763 | 4,503410846 | 19,5780814 | 9,65708E-06 | 7,95644E-05 |
| b3183 | 1173 | 0,53 | 1,081072133 | 6,98755523 | 19,55488687 | 9,77504E-06 | 8,03885E-05 |
| b4658 | 836 | 0,53 | -1,103227951 | 6,650702991 | 19,54779836 | 9,81137E-06 | 8,05395E-05 |
| b1064 | 648 | 0,49 | -1,089075126 | 7,954482761 | 19,51231537 | 9,99532E-06 | 8,17526E-05 |
| b2524 | 201 | 0,5 | 1,468264817 | 4,47043483 | 19,51225621 | 9,99563E-06 | 8,17526E-05 |
| b2431 | 900 | 0,56 | -1,353804407 | 8,71766915 | 19,50749545 | 1,00206E-05 | 8,18073E-05 |
| b3307 | 306 | 0,53 | 1,599194677 | 10,12395859 | 19,47923344 | 1,01699E-05 | 8,28757E-05 |
| b0314 | 2034 | 0,55 | 1,22080798 | 5,92121651 | 19,47404353 | 1,01976E-05 | 8,29504E-05 |
| b2364 | 936 | 0,48 | -0,99143347 | 4,85764985 | 19,46022226 | 1,02717E-05 | 8,34014E-05 |
| b4201 | 315 | 0,52 | 1,222077096 | 8,949562897 | 19,38730368 | 1,06714E-05 | 8,64906E-05 |
| b3845 | 1164 | 0,58 | 2,287939193 | 6,710397151 | 19,36096402 | 1,08196E-05 | 8,75335E-05 |
| b0270 | 1383 | 0,62 | -1,301728066 | 4,336934151 | 19,33389754 | 1,09741E-05 | 8,86231E-05 |
| b3474 | 1050 | 0,51 | -1,126569796 | 6,121977884 | 19,32839627 | 1,10057E-05 | 8,87189E-05 |
| b3983 | 429 | 0,52 | 1,532677069 | 9,561116436 | 19,32011549 | 1,10536E-05 | 8,88132E-05 |
| b3304 | 354 | 0,54 | 1,426167146 | 9,799002724 | 19,31951148 | 1,10571E-05 | 8,88132E-05 |
| b1743 | 486 | 0,51 | -1,210075232 | 6,136129208 | 19,23232549 | 1,15737E-05 | 9,27966E-05 |
| b1227 | 678 | 0,56 | -3,227107044 | 8,848587224 | 19,20190475 | 1,17596E-05 | 9,41188E-05 |
| b3312 | 192 | 0,54 | 1,468592362 | 9,145676251 | 19,13133116 | 1,22025E-05 | 9,74897E-05 |
| b2802 | 1776 | 0,55 | -1,563704081 | 6,131753918 | 19,1121716 | 1,23256E-05 | 9,8298E-05 |
| b1761 | 1344 | 0,53 | 1,110673256 | 6,586044529 | 19,08601915 | 1,24957E-05 | 9,94773E-05 |
| b0488 | 459 | 0,57 | -1,403652879 | 6,391116905 | 19,07655193 | 1,25578E-05 | 9,97947E-05 |
| b1415 | 1440 | 0,51 | 2,172924499 | 10,98753515 | 19,03899849 | 1,28074E-05 | 0,000101598 |
| b0407 | 333 | 0,5 | 1,014471313 | 7,530783333 | 18,95051444 | 1,34153E-05 | 0,000106232 |
| b3821 | 870 | 0,51 | 1,279839382 | 5,256218129 | 18,9471474 | 1,3439E-05 | 0,000106232 |
| b0180 | 456 | 0,5 | 0,926620995 | 7,62872686 | 18,93652442 | 1,3514E-05 | 0,000106637 |
| b4179 | 2442 | 0,55 | -1,091564898 | 9,756909368 | 18,90723094 | 1,37232E-05 | 0,000107933 |
| b2288 | 444 | 0,53 | 1,108714311 | 7,979300632 | 18,90677236 | 1,37265E-05 | 0,000107933 |
| b1274 | 2598 | 0,52 | 1,096518769 | 8,987925069 | 18,87765892 | 1,39375E-05 | 0,000109401 |
| b2304 | 894 | 0,55 | -0,940734228 | 8,021077313 | 18,85715877 | 1,40881E-05 | 0,00011039 |
| b0484 | 2505 | 0,58 | -1,232440573 | 9,264836823 | 18,8525066 | 1,41225E-05 | 0,000110466 |
| b0183 | 597 | 0,57 | 1,149359849 | 4,857097977 | 18,84697471 | 1,41636E-05 | 0,000110594 |
| b0740 | 1293 | 0,53 | 0,998009857 | 9,195662405 | 18,71590584 | 1,51712E-05 | 0,000118256 |
| b4240 | 1422 | 0,54 | -1,313081049 | 9,010296015 | 18,7118715 | 1,52033E-05 | 0,000118301 |
| b3385 | 759 | 0,53 | 0,990026862 | 7,1631241 | 18,69072177 | 1,53729E-05 | 0,000119413 |
| b4051 | 984 | 0,53 | -1,388609643 | 9,217276733 | 18,68095624 | 1,54518E-05 | 0,000119818 |
| b3786 | 1131 | 0,53 | 1,198809704 | 5,90327685 | 18,67612701 | 1,5491E-05 | 0,000119915 |
| b3494 | 336 | 0,47 | -1,447873363 | 8,16243495 | 18,64219299 | 1,57692E-05 | 0,000121858 |
| b1264 | 1563 | 0,54 | -7,490040893 | 9,341414367 | 18,61410116 | 1,60032E-05 | 0,000123454 |
| b3560 | 912 | 0,56 | 1,055103533 | 8,427730106 | 18,60641135 | 1,60679E-05 | 0,00012374 |
| b0419 | 975 | 0,53 | -1,163983388 | 9,022985625 | 18,59887736 | 1,61316E-05 | 0,000124016 |
| b1038 | 417 | 0,42 | 2,630372192 | 2,268388546 | 18,58551499 | 1,6245E-05 | 0,000124675 |
| b0478 | 1677 | 0,55 | -1,359649042 | 8,56270276 | 18,57510764 | 1,6334E-05 | 0,000125143 |
| b0591 | 1251 | 0,58 | 2,001469376 | 2,209745733 | 18,53454366 | 1,66853E-05 | 0,000127617 |
| b3631 | 1125 | 0,45 | 0,924720083 | 6,033903549 | 18,53077201 | 1,67183E-05 | 0,000127652 |
| b0408 | 1848 | 0,53 | 0,969992719 | 8,280280421 | 18,51046693 | 1,68974E-05 | 0,000128799 |
| b2647 | 4581 | 0,49 | -0,866571146 | 6,885114133 | 18,4952912 | 1,70325E-05 | 0,000129516 |
| b3496 | 1470 | 0,52 | -1,024252682 | 9,038539224 | 18,49342801 | 1,70491E-05 | 0,000129516 |
| b1687 | 3057 | 0,54 | 1,014065246 | 8,726002234 | 18,46816313 | 1,72766E-05 | 0,000131022 |
| b0945 | 1011 | 0,47 | 1,737081531 | 3,897401872 | 18,464043 | 1,7314E-05 | 0,000131084 |
| b3200 | 558 | 0,5 | 0,806815282 | 7,280137951 | 18,44838039 | 1,74569E-05 | 0,000131942 |
| b0585 | 1125 | 0,55 | 2,32761838 | 1,637938414 | 18,42051678 | 1,7714E-05 | 0,00013366 |
| b0186 | 2142 | 0,51 | -0,963483017 | 8,458696042 | 18,38588684 | 1,80389E-05 | 0,000135883 |
| b3103 | 366 | 0,52 | -0,98230722 | 7,285691506 | 18,38048105 | 1,80902E-05 | 0,00013604 |
| b3536 | 1572 | 0,49 | -0,918104521 | 7,985708692 | 18,37684609 | 1,81247E-05 | 0,00013605 |
| b1252 | 720 | 0,54 | 2,589333101 | 4,462865279 | 18,37396085 | 1,81522E-05 | 0,00013605 |
| b2627 | 2190 | 0,49 | -0,76172568 | 7,042683639 | 18,3627897 | 1,82589E-05 | 0,000136622 |
| b1831 | 699 | 0,53 | 1,403421212 | 7,991577088 | 18,34595567 | 1,8421E-05 | 0,000137605 |
| b4052 | 1416 | 0,54 | 0,92358539 | 6,768055383 | 18,29955472 | 1,88751E-05 | 0,000140763 |
| b1807 | 696 | 0,56 | 1,633883576 | 4,858444709 | 18,27461825 | 1,91238E-05 | 0,00014238 |
| b2766 | 1272 | 0,55 | -1,318238839 | 5,50789789 | 18,26357312 | 1,9235E-05 | 0,000142971 |
| b0992 | 1074 | 0,49 | -2,774204375 | 5,550500672 | 18,25737699 | 1,92977E-05 | 0,000143112 |
| b0121 | 867 | 0,51 | 0,899862091 | 6,612593631 | 18,25538595 | 1,93179E-05 | 0,000143112 |
| b2232 | 723 | 0,54 | 1,296424498 | 5,677676518 | 18,24142259 | 1,946E-05 | 0,000143927 |
| b3193 | 552 | 0,5 | 0,811293625 | 5,93525731 | 18,21399985 | 1,97422E-05 | 0,000145774 |
| b1714 | 984 | 0,54 | 1,003434677 | 7,328474203 | 18,18865127 | 2,00067E-05 | 0,000147484 |
| b1824 | 144 | 0,42 | 1,392148786 | 9,208413032 | 18,18495726 | 2,00456E-05 | 0,000147528 |
| b0653 | 675 | 0,5 | 1,319917243 | 5,230645665 | 18,17518724 | 2,01487E-05 | 0,000148043 |
| b2213 | 1065 | 0,57 | -1,346242297 | 6,418521943 | 18,15762731 | 2,03353E-05 | 0,00014917 |
| b0829 | 1872 | 0,54 | -0,735347461 | 7,066445482 | 18,05904536 | 2,14159E-05 | 0,000156666 |
| b3614 | 960 | 0,53 | 1,51420986 | 4,43995582 | 18,05804981 | 2,14271E-05 | 0,000156666 |
| b0784 | 246 | 0,56 | -1,826259809 | 6,005837475 | 18,04311376 | 2,15958E-05 | 0,000157585 |
| b2708 | 966 | 0,57 | -1,314497096 | 6,319060405 | 18,04070912 | 2,16231E-05 | 0,000157585 |
| b2064 | 1854 | 0,53 | -0,704695216 | 7,28243993 | 17,99912021 | 2,21007E-05 | 0,000160615 |
| b0588 | 816 | 0,54 | 2,255289218 | 2,418295686 | 17,99827001 | 2,21106E-05 | 0,000160615 |
| b2508 | 1467 | 0,55 | 1,740692519 | 8,32942852 | 17,96317622 | 2,2522E-05 | 0,000163339 |
| b2066 | 642 | 0,49 | 1,502453818 | 4,66296532 | 17,93282394 | 2,2884E-05 | 0,000165697 |
| b2670 | 450 | 0,49 | -1,152783365 | 7,071741202 | 17,9209062 | 2,30278E-05 | 0,000166207 |

| | | | | | | | |
|---|---|---|---|---|---|---|---|
| b1839 | 342 | 0,46 | 1,171382939 | 5,23114792 | 17,92083217 | 2,30287E-05 | 0,000166207 |
| b3075 | 984 | 0,52 | -1,095955332 | 5,575417696 | 17,90849549 | 2,31784E-05 | 0,000166806 |
| b2210 | 1647 | 0,54 | 1,666624681 | 4,616495498 | 17,90786426 | 2,31861E-05 | 0,000166806 |
| b3612 | 1545 | 0,53 | -1,120494031 | 8,79586655 | 17,88878618 | 2,34197E-05 | 0,000168217 |
| b0445 | 1701 | 0,53 | -1,245499017 | 5,926959529 | 17,88004935 | 2,35275E-05 | 0,00016872 |
| b2575 | 738 | 0,52 | 1,466159709 | 3,599686856 | 17,81178175 | 2,43869E-05 | 0,000174604 |
| b0677 | 1149 | 0,52 | -0,824665624 | 7,735950437 | 17,80460778 | 2,4479E-05 | 0,000174984 |
| b2174 | 714 | 0,44 | 1,810990341 | 3,107423507 | 17,78100509 | 2,47845E-05 | 0,000176886 |
| b3401 | 879 | 0,53 | 0,804515484 | 6,930711438 | 17,70917336 | 2,57382E-05 | 0,0001834 |
| b1234 | 906 | 0,5 | -0,85941111 | 7,332143334 | 17,70337835 | 2,58168E-05 | 0,000183668 |
| b3998 | 672 | 0,59 | 0,917467822 | 6,11976977 | 17,68265958 | 2,60995E-05 | 0,000185259 |
| b4522 | 129 | 0,5 | -1,292582576 | 4,992320157 | 17,68093976 | 2,61231E-05 | 0,000185259 |
| b0699 | 207 | 0,5 | -2,315783031 | 5,841639118 | 17,67400384 | 2,62185E-05 | 0,000185642 |
| b3919 | 768 | 0,53 | -1,08644652 | 10,44337994 | 17,6700027 | 2,62738E-05 | 0,000185718 |
| b0308 | 696 | 0,52 | -1,828912149 | 5,978995101 | 17,66722526 | 2,63122E-05 | 0,000185718 |
| b3634 | 480 | 0,53 | 1,19901503 | 4,816186018 | 17,6392973 | 2,67014E-05 | 0,000188169 |
| b0581 | 1119 | 0,52 | -1,208101625 | 8,338620965 | 17,63186735 | 2,68059E-05 | 0,000188609 |
| b0927 | 648 | 0,52 | 1,100865011 | 6,924311696 | 17,61594401 | 2,70313E-05 | 0,000189897 |
| b2707 | 774 | 0,49 | -1,261684074 | 6,474192482 | 17,6114632 | 2,7095E-05 | 0,000190047 |
| b3573 | 474 | 0,54 | -1,678556635 | 5,974282965 | 17,60158674 | 2,72361E-05 | 0,000190738 |
| b1866 | 1773 | 0,54 | 0,953762419 | 9,084374924 | 17,58899632 | 2,7417E-05 | 0,000191705 |
| b3282 | 573 | 0,52 | 1,042390995 | 5,47245571 | 17,57292711 | 2,76497E-05 | 0,000193031 |
| b0181 | 789 | 0,53 | 0,812171477 | 8,366675278 | 17,53704205 | 2,81764E-05 | 0,000196403 |
| b3408 | 228 | 0,49 | 2,698921457 | 4,043737156 | 17,52943882 | 2,82893E-05 | 0,000196883 |
| b2186 | 1008 | 0,52 | 0,965873499 | 6,01342072 | 17,52278494 | 2,83885E-05 | 0,000197267 |
| b3162 | 1890 | 0,56 | 1,137813681 | 7,358171538 | 17,51339059 | 2,85291E-05 | 0,000197937 |
| b2994 | 1704 | 0,53 | -1,73925561 | 8,412212899 | 17,5038782 | 2,86722E-05 | 0,000198387 |
| b0033 | 3222 | 0,56 | 2,102825144 | 8,066253655 | 17,50319622 | 2,86825E-05 | 0,000198387 |
| b0824 | 900 | 0,54 | -1,879009114 | 2,810149492 | 17,49907276 | 2,87448E-05 | 0,000198512 |
| b0865 | 516 | 0,54 | -1,105093977 | 8,966938693 | 17,49230224 | 2,88473E-05 | 0,000198913 |
| b3905 | 837 | 0,52 | -1,365851875 | 4,603498164 | 17,48771405 | 2,8917E-05 | 0,000199088 |
| b3253 | 975 | 0,55 | -1,029788451 | 8,107739426 | 17,46627878 | 2,92449E-05 | 0,000201036 |
| b3986 | 366 | 0,46 | 1,512772397 | 10,57012713 | 17,43845302 | 2,96761E-05 | 0,000203634 |
| b3232 | 1128 | 0,52 | 1,033221055 | 5,228622997 | 17,43443912 | 2,97389E-05 | 0,000203634 |
| b3973 | 966 | 0,49 | 1,036354615 | 5,513890672 | 17,43314822 | 2,97591E-05 | 0,000203634 |
| b0866 | 324 | 0,54 | -1,172877424 | 6,932676531 | 17,41595959 | 3,00294E-05 | 0,00020517 |
| b2320 | 1137 | 0,53 | 0,806171453 | 7,180240352 | 17,38712212 | 3,04885E-05 | 0,00020799 |
| b1426 | 225 | 0,41 | -1,074503981 | 7,450419047 | 17,35850814 | 3,0951E-05 | 0,000210824 |
| b0475 | 963 | 0,54 | -1,08065133 | 7,015145539 | 17,32384578 | 3,15207E-05 | 0,000214243 |
| b2954 | 594 | 0,56 | 1,508003131 | 5,092899094 | 17,32216873 | 3,15485E-05 | 0,000214243 |
| b1710 | 552 | 0,49 | 1,265001022 | 6,620543594 | 17,28444814 | 3,2181E-05 | 0,000218208 |
| b3982 | 546 | 0,52 | 0,978919901 | 7,95770143 | 17,21943385 | 3,33012E-05 | 0,000225462 |
| b0676 | 1221 | 0,53 | -0,800873779 | 7,362587331 | 17,21459872 | 3,33861E-05 | 0,000225696 |
| b4293 | 522 | 0,55 | 2,384874267 | 2,743104194 | 17,2083677 | 3,34958E-05 | 0,000226096 |
| b0170 | 852 | 0,5 | 1,430182807 | 10,0721586 | 17,20120694 | 3,36223E-05 | 0,000226609 |
| b0480 | 1653 | 0,51 | -0,953620924 | 8,735978679 | 17,10702073 | 3,53315E-05 | 0,000237771 |
| b0723 | 1767 | 0,56 | 1,985857614 | 11,40339559 | 17,0973264 | 3,55123E-05 | 0,00023863 |
| b3955 | 1734 | 0,5 | 1,046139201 | 5,754078223 | 17,06916763 | 3,60427E-05 | 0,000241832 |
| b0129 | 441 | 0,49 | -1,358364651 | 5,434949686 | 17,04264278 | 3,65497E-05 | 0,000244867 |
| b1684 | 369 | 0,53 | -1,457583434 | 6,77644578 | 17,03406741 | 3,67151E-05 | 0,000245446 |
| b0159 | 699 | 0,52 | 0,811759738 | 7,564801607 | 17,03248558 | 3,67457E-05 | 0,000245446 |
| b3922 | 300 | 0,46 | -0,99310965 | 6,787420079 | 16,99100038 | 3,75574E-05 | 0,000250495 |
| b2142 | 696 | 0,53 | -1,398219108 | 5,204524958 | 16,94524493 | 3,84736E-05 | 0,000256224 |
| b3603 | 1656 | 0,55 | 3,40054561 | 6,300721173 | 16,92737055 | 3,88375E-05 | 0,000258264 |
| b0169 | 726 | 0,51 | 1,398021211 | 10,60060907 | 16,87925754 | 3,98345E-05 | 0,000264501 |
| b1968 | 1359 | 0,4 | 1,337448355 | 4,310710362 | 16,84655355 | 4,05269E-05 | 0,0002687 |
| b1871 | 972 | 0,52 | 1,020970938 | 5,993936336 | 16,76727974 | 4,22557E-05 | 0,000279355 |
| b0737 | 693 | 0,51 | 1,317154047 | 5,339658405 | 16,76715243 | 4,22585E-05 | 0,000279355 |
| b4364 | 771 | 0,52 | -1,593433801 | 4,965107353 | 16,75683209 | 4,2489E-05 | 0,000280465 |
| b0023 | 264 | 0,47 | 2,102907404 | 7,366113661 | 16,73789935 | 4,29151E-05 | 0,000282861 |
| b1039 | 390 | 0,43 | 2,760650637 | 2,186603408 | 16,7201249 | 4,3319E-05 | 0,000284719 |
| b0809 | 723 | 0,54 | 1,343156371 | 6,436261304 | 16,71990226 | 4,33241E-05 | 0,000284719 |
| b0759 | 1017 | 0,54 | -0,908789647 | 8,537661288 | 16,69836546 | 4,38187E-05 | 0,000287548 |
| b1531 | 384 | 0,46 | 1,409260228 | 3,688597067 | 16,68840655 | 4,40494E-05 | 0,000288639 |
| b3184 | 966 | 0,53 | 1,252815951 | 5,419491455 | 16,68000657 | 4,42449E-05 | 0,000289497 |
| b1263 | 1596 | 0,56 | -7,164597417 | 10,19397485 | 16,64897428 | 4,49746E-05 | 0,000293843 |
| b2972 | 810 | 0,52 | -1,284539429 | 4,159219432 | 16,63370217 | 4,53382E-05 | 0,000295641 |
| b2344 | 1341 | 0,5 | 1,768749792 | 8,001658325 | 16,63188258 | 4,53817E-05 | 0,000295641 |
| b2877 | 579 | 0,43 | -2,07117402 | 6,636901834 | 16,62395612 | 4,55718E-05 | 0,000296448 |
| b0828 | 966 | 0,57 | -0,88467336 | 6,908940736 | 16,61421196 | 4,58065E-05 | 0,000297543 |
| b0848 | 378 | 0,5 | -1,5735386 | 3,917304929 | 16,57456494 | 4,67741E-05 | 0,000303389 |
| b4388 | 969 | 0,57 | 0,988416378 | 5,675703802 | 16,56944551 | 4,69006E-05 | 0,000303648 |
| b3477 | 945 | 0,58 | -2,969793425 | 5,000491161 | 16,56746702 | 4,69495E-05 | 0,000303648 |
| b1537 | 519 | 0,49 | 1,54862658 | 3,951932354 | 16,52152883 | 4,81008E-05 | 0,000310645 |
| b3703 | 141 | 0,52 | 1,698436249 | 5,383139625 | 16,49326793 | 4,88231E-05 | 0,000314856 |
| b0897 | 627 | 0,46 | -1,051137811 | 9,380717802 | 16,38338942 | 5,17366E-05 | 0,000332887 |
| b3336 | 477 | 0,43 | -1,136282543 | 10,85461303 | 16,38161029 | 5,17852E-05 | 0,000332887 |
| b3606 | 474 | 0,55 | 1,360969013 | 3,429078589 | 16,3795367 | 5,18419E-05 | 0,000332887 |

| | | | | | | | |
|---|---|---|---|---|---|---|---|
| b3984 | 705 | 0,52 | 1,434965453 | 10,66794625 | 16,35791524 | 5,24367E-05 | 0,000336225 |
| b0610 | 411 | 0,55 | 0,978528987 | 6,189941407 | 16,32389129 | 5,33866E-05 | 0,000341827 |
| b0069 | 1656 | 0,55 | -0,852756391 | 5,244120674 | 16,28225642 | 5,45726E-05 | 0,000348862 |
| b3639 | 1221 | 0,54 | 0,934081389 | 6,613154753 | 16,27988296 | 5,4641E-05 | 0,000348862 |
| b3311 | 255 | 0,47 | 1,439877387 | 9,511542953 | 16,25152145 | 5,5465E-05 | 0,000353619 |
| b3931 | 1332 | 0,53 | 1,1432716 | 8,573845337 | 16,23636117 | 5,59106E-05 | 0,000355954 |
| b0907 | 1089 | 0,5 | 1,008909322 | 7,810281755 | 16,23328307 | 5,60015E-05 | 0,000356027 |
| b2157 | 882 | 0,54 | -0,886024838 | 6,812300983 | 16,20172167 | 5,69423E-05 | 0,000361495 |
| b4328 | 1173 | 0,57 | 0,998158387 | 5,627619939 | 16,19739059 | 5,70727E-05 | 0,000361785 |
| b0972 | 1119 | 0,56 | -1,700385684 | 5,792629944 | 16,19450469 | 5,71597E-05 | 0,000361785 |
| b3020 | 1608 | 0,52 | 1,171539889 | 6,030050185 | 16,19217038 | 5,72302E-05 | 0,000361785 |
| b4189 | 330 | 0,49 | -1,737099023 | 9,800577235 | 16,16678015 | 5,80024E-05 | 0,000366151 |
| b0007 | 1431 | 0,53 | -1,18032431 | 5,887737382 | 16,13674443 | 5,89296E-05 | 0,00037148 |
| b3301 | 435 | 0,54 | 1,308443834 | 10,9314355 | 16,12207636 | 5,93877E-05 | 0,000373842 |
| b2316 | 915 | 0,55 | 1,160981791 | 8,47959503 | 16,07791948 | 6,07887E-05 | 0,000382125 |
| b4149 | 534 | 0,53 | -1,220336791 | 7,882831546 | 16,06530534 | 6,1195E-05 | 0,00038414 |
| b3453 | 1317 | 0,54 | 1,747374666 | 7,32838999 | 16,05282574 | 6,15996E-05 | 0,000386139 |
| b4515 | 114 | 0,52 | -1,746897354 | 6,22410889 | 16,03827813 | 6,20747E-05 | 0,000388574 |
| b2948 | 564 | 0,51 | 1,010780601 | 5,738804351 | 15,99621074 | 6,34694E-05 | 0,00039675 |
| b4203 | 450 | 0,5 | 1,186101656 | 9,570985424 | 15,95663487 | 6,48102E-05 | 0,000404567 |
| b0113 | 765 | 0,56 | 1,714743534 | 4,96876055 | 15,92662715 | 6,58458E-05 | 0,00041046 |
| b0854 | 1113 | 0,51 | 1,572179473 | 5,534524111 | 15,88490383 | 6,73135E-05 | 0,000419027 |
| b0873 | 1653 | 0,55 | -3,616083261 | 7,66096822 | 15,87776862 | 6,75678E-05 | 0,000420026 |
| b3621 | 960 | 0,51 | 0,901663392 | 5,951019707 | 15,87005244 | 6,78438E-05 | 0,000421158 |
| b2980 | 765 | 0,55 | 1,547917708 | 5,280045439 | 15,82329135 | 6,95412E-05 | 0,000431098 |
| b0185 | 960 | 0,52 | 0,979682878 | 8,078742507 | 15,81785198 | 6,97414E-05 | 0,000431193 |
| b0261 | 933 | 0,6 | -0,930298921 | 5,89613621 | 15,81701885 | 6,97721E-05 | 0,000431193 |
| b3032 | 828 | 0,57 | -0,955652312 | 8,436568519 | 15,81503986 | 6,98451E-05 | 0,000431193 |
| b0680 | 1665 | 0,52 | 1,060239769 | 8,323173855 | 15,81086286 | 6,99995E-05 | 0,000431261 |
| b2507 | 1578 | 0,54 | 1,477794984 | 9,152003868 | 15,80953288 | 7,00487E-05 | 0,000431261 |
| b2317 | 660 | 0,49 | 1,104189682 | 5,556177088 | 15,77223466 | 7,14433E-05 | 0,000439244 |
| b4204 | 795 | 0,42 | 2,948626815 | 1,790136765 | 15,68090993 | 7,49771E-05 | 0,000460339 |
| b4367 | 789 | 0,53 | 3,325717701 | 4,220294968 | 15,67811869 | 7,50878E-05 | 0,000460388 |
| b0908 | 1284 | 0,51 | 1,333554007 | 6,230310145 | 15,65893821 | 7,58532E-05 | 0,000464446 |
| b1855 | 972 | 0,52 | 0,788390719 | 6,415228397 | 15,65348323 | 7,60723E-05 | 0,000465152 |
| b4554 | 210 | 0,49 | -1,492188642 | 7,928247734 | 15,62670669 | 7,71571E-05 | 0,000471142 |
| b2476 | 714 | 0,52 | 1,597773811 | 8,458513998 | 15,60796386 | 7,79257E-05 | 0,000475188 |
| b0345 | 1083 | 0,56 | 1,306325223 | 5,496779667 | 15,60147465 | 7,81936E-05 | 0,000476173 |
| b3598 | 363 | 0,52 | -1,655113582 | 5,823335007 | 15,58064088 | 7,90599E-05 | 0,000480796 |
| b0457 | 1551 | 0,48 | -0,924493267 | 6,441122412 | 15,57302969 | 7,93788E-05 | 0,000482081 |
| b3349 | 591 | 0,55 | 0,940146131 | 9,447171684 | 15,50833072 | 8,21424E-05 | 0,00049819 |
| b0470 | 1932 | 0,58 | 1,09986873 | 6,812192324 | 15,46112999 | 8,42195E-05 | 0,000510097 |
| b1654 | 348 | 0,53 | 1,02270956 | 7,203601781 | 15,45374753 | 8,45491E-05 | 0,000511402 |
| b2081 | 1362 | 0,54 | 1,274611714 | 6,005795717 | 15,44392892 | 8,49895E-05 | 0,000513373 |
| b4238 | 2139 | 0,54 | -1,611216115 | 8,371245554 | 15,4173826 | 8,61918E-05 | 0,000519935 |
| b1632 | 696 | 0,53 | 1,529039907 | 4,269973396 | 15,41461995 | 8,63179E-05 | 0,000519996 |
| b1384 | 906 | 0,49 | 1,91596219 | 4,538504594 | 15,40815957 | 8,66135E-05 | 0,000521076 |
| b0973 | 1794 | 0,57 | -1,738386058 | 6,140751583 | 15,39443096 | 8,7245E-05 | 0,000523001 |
| b0825 | 663 | 0,54 | -1,166981416 | 4,638512114 | 15,39420299 | 8,72556E-05 | 0,000523001 |
| b0930 | 1401 | 0,52 | 0,855044376 | 8,689069917 | 15,39188154 | 8,73628E-05 | 0,000523001 |
| b1186 | 1542 | 0,53 | 0,855109124 | 6,034800246 | 15,39107457 | 8,74001E-05 | 0,000523001 |
| b0175 | 858 | 0,51 | 0,682085634 | 7,19909671 | 15,36842454 | 8,84541E-05 | 0,000528602 |
| b3873 | 981 | 0,44 | 1,641962449 | 3,953778949 | 15,36421551 | 8,86514E-05 | 0,000528731 |
| b3485 | 1125 | 0,55 | -0,955375224 | 6,809740836 | 15,36293065 | 8,87117E-05 | 0,000528731 |
| b0093 | 831 | 0,53 | -0,937150015 | 8,424199311 | 15,32251452 | 9,06299E-05 | 0,000539447 |
| b2203 | 450 | 0,56 | -2,324550486 | 5,978772316 | 15,31133597 | 9,11678E-05 | 0,000541928 |
| b3522 | 1014 | 0,53 | -1,104141058 | 7,52686396 | 15,29720589 | 9,18523E-05 | 0,000545274 |
| b4200 | 396 | 0,53 | 1,14402403 | 9,380109788 | 15,28863341 | 9,22701E-05 | 0,000546802 |
| b1069 | 1536 | 0,54 | 0,988114224 | 4,883670651 | 15,28692143 | 9,23537E-05 | 0,000546802 |
| b3519 | 1650 | 0,54 | -1,231964633 | 7,578434306 | 15,25661023 | 9,38477E-05 | 0,000554915 |
| b1928 | 414 | 0,57 | 0,887002905 | 6,0680922 | 15,23977454 | 9,4688E-05 | 0,000559146 |
| b2907 | 1179 | 0,6 | -0,955469737 | 7,94860104 | 15,17157234 | 9,81703E-05 | 0,000578946 |
| b0166 | 825 | 0,53 | 1,095238002 | 8,230554769 | 15,12768477 | 0,000100479 | 0,000591783 |
| b1672 | 648 | 0,52 | -1,418909309 | 3,453231408 | 15,12507171 | 0,000100618 | 0,000591825 |
| b2966 | 708 | 0,52 | -1,580444052 | 5,062785259 | 15,09122359 | 0,000102438 | 0,000601742 |
| b0426 | 492 | 0,5 | 0,772739237 | 8,572563902 | 15,04939756 | 0,000104734 | 0,000614419 |
| b1560 | 1050 | 0,52 | 2,621914732 | 1,433373859 | 15,04572202 | 0,000104938 | 0,000614812 |
| b3710 | 1176 | 0,53 | 1,765338434 | 2,809653723 | 14,99669773 | 0,000107699 | 0,000630168 |
| b4074 | 1659 | 0,57 | -1,921970421 | 4,202836107 | 14,97723007 | 0,000108816 | 0,000635773 |
| b0791 | 411 | 0,55 | -1,084004023 | 7,627486334 | 14,97506998 | 0,000108941 | 0,000635773 |
| b2315 | 1269 | 0,56 | 0,68756317 | 6,558567922 | 14,94905262 | 0,000110454 | 0,000643762 |
| b1334 | 753 | 0,52 | 0,927220139 | 8,167327463 | 14,93797433 | 0,000111104 | 0,000646712 |
| b2285 | 501 | 0,55 | 1,076520054 | 9,03065662 | 14,92377525 | 0,000111943 | 0,00064995 |
| b0766 | 819 | 0,52 | 1,021509195 | 4,90458924 | 14,92365607 | 0,00011195 | 0,00064995 |
| b2801 | 1317 | 0,48 | -1,25711166 | 5,064263705 | 14,89518841 | 0,000113652 | 0,000658304 |
| b0241 | 1056 | 0,45 | 1,992680656 | 2,368926174 | 14,89468177 | 0,000113683 | 0,000658304 |
| b2258 | 387 | 0,49 | -1,088605253 | 4,564427061 | 14,83453008 | 0,000117367 | 0,000678759 |
| b3709 | 1248 | 0,46 | 3,312122497 | 8,393691199 | 14,82856974 | 0,000117738 | 0,00068003 |

| | | | | | | | |
|---|---|---|---|---|---|---|---|
| b4126 | 231 | 0,49 | -1,095869171 | 6,497591858 | 14,81256838 | 0,000118741 | 0,000684941 |
| b2318 | 813 | 0,55 | 0,678975649 | 6,536416747 | 14,79116364 | 0,000120097 | 0,000691233 |
| b4217 | 207 | 0,43 | 0,850364838 | 8,696675378 | 14,79047612 | 0,000120141 | 0,000691233 |
| b1222 | 1797 | 0,53 | -1,382546334 | 7,935051578 | 14,78248164 | 0,000120651 | 0,00069328 |
| b0398 | 1203 | 0,53 | 1,084449052 | 4,901781496 | 14,7781309 | 0,00012093 | 0,000693992 |
| b4292 | 954 | 0,59 | 1,77636322 | 2,491376169 | 14,7720121 | 0,000121323 | 0,000695124 |
| b3181 | 477 | 0,48 | 1,479192274 | 5,536977558 | 14,77023508 | 0,000121437 | 0,000695124 |
| b1683 | 1488 | 0,52 | -1,314023302 | 8,878311922 | 14,74170173 | 0,000123289 | 0,000704823 |
| b3300 | 1332 | 0,5 | 1,207389348 | 12,0874849 | 14,68881949 | 0,000126796 | 0,00072395 |
| b2791 | 783 | 0,54 | 1,230268266 | 4,362620792 | 14,60431708 | 0,00013261 | 0,000756183 |
| b1044 | 312 | 0,44 | 2,864012926 | 1,486774427 | 14,57446885 | 0,000134728 | 0,00076728 |
| b0388 | 525 | 0,52 | -1,228956137 | 5,963672961 | 14,52003078 | 0,000138677 | 0,00078877 |
| b2697 | 2631 | 0,54 | 1,136037574 | 9,897349828 | 14,50613693 | 0,000139704 | 0,000793602 |
| b1645 | 2013 | 0,53 | -1,051152512 | 5,874793833 | 14,48622526 | 0,000141188 | 0,00080102 |
| b3389 | 1089 | 0,54 | 0,754033614 | 8,036701094 | 14,45673766 | 0,000143416 | 0,00081263 |
| b4465 | 1278 | 0,51 | 1,752282798 | 2,488675461 | 14,44663518 | 0,000144187 | 0,000815969 |
| b3209 | 654 | 0,53 | -0,884519741 | 7,831736694 | 14,42369702 | 0,000145954 | 0,000824556 |
| b4054 | 1194 | 0,53 | 1,021558085 | 6,44955779 | 14,42216818 | 0,000146073 | 0,000824556 |
| b2009 | 474 | 0,47 | -1,026587757 | 8,040437958 | 14,38245166 | 0,000149186 | 0,000841072 |
| b1187 | 720 | 0,51 | -0,77864737 | 6,649688376 | 14,3564889 | 0,000151257 | 0,000851678 |
| b2527 | 516 | 0,52 | 1,829691398 | 4,938919928 | 14,3224762 | 0,000154015 | 0,000866117 |
| b4354 | 2151 | 0,56 | -1,111410828 | 5,610820673 | 14,30518467 | 0,000155436 | 0,000873014 |
| b0433 | 1476 | 0,54 | 1,285806242 | 4,776867113 | 14,26292606 | 0,000158965 | 0,000891718 |
| b0028 | 450 | 0,54 | 0,905468085 | 5,916492974 | 14,25446135 | 0,000159682 | 0,000894618 |
| b3883 | 897 | 0,56 | -1,3304768 | 4,270384512 | 14,24304005 | 0,000160654 | 0,000898939 |
| b2749 | 324 | 0,48 | 1,783199267 | 3,240170906 | 14,19959119 | 0,000164406 | 0,000918789 |
| b3387 | 837 | 0,5 | 0,829837743 | 6,223470471 | 14,19551788 | 0,000164763 | 0,000919633 |
| b1524 | 927 | 0,52 | 1,337849985 | 4,333200915 | 14,1850259 | 0,000165684 | 0,000923625 |
| b3066 | 1746 | 0,52 | 0,791640332 | 7,620718324 | 14,17395245 | 0,000166662 | 0,000927029 |
| b3859 | 987 | 0,49 | 1,092253285 | 5,971701188 | 14,17343011 | 0,000166708 | 0,000927029 |
| b1000 | 921 | 0,53 | -1,123025831 | 9,889968666 | 14,13585036 | 0,000170071 | 0,000943935 |
| b0497 | 4281 | 0,6 | -1,022267498 | 5,240603433 | 14,13476648 | 0,000170169 | 0,000943935 |
| b0741 | 522 | 0,51 | 1,025035944 | 10,10212776 | 14,11382493 | 0,000172074 | 0,000953322 |
| b3820 | 468 | 0,53 | 2,583416167 | 4,034633551 | 14,10419097 | 0,000172958 | 0,000957034 |
| b2240 | 1359 | 0,53 | -0,973600745 | 4,873764286 | 14,0982337 | 0,000173507 | 0,000958886 |
| b0347 | 1665 | 0,57 | 1,53805597 | 2,392932354 | 14,08936407 | 0,000174327 | 0,000962232 |
| b0002 | 2463 | 0,53 | 1,229704015 | 9,05383418 | 14,06642955 | 0,000176465 | 0,000972839 |
| b0884 | 219 | 0,48 | 1,643392033 | 5,620822134 | 14,05900571 | 0,000177163 | 0,000975487 |
| b2027 | 981 | 0,46 | 1,235307567 | 7,557554653 | 14,03899854 | 0,000179058 | 0,000984711 |
| b1804 | 1128 | 0,54 | 1,144919097 | 4,697757415 | 14,012181 | 0,00018163 | 0,00099763 |
| b1487 | 1551 | 0,5 | 1,954628445 | 5,481615835 | 14,00904522 | 0,000181933 | 0,000998072 |
| b4351 | 915 | 0,51 | -1,021821471 | 6,782504376 | 13,98621761 | 0,000184156 | 0,001009029 |
| b3431 | 1974 | 0,54 | -0,996477337 | 9,302414566 | 13,95866995 | 0,000186874 | 0,001022672 |
| b4466 | 4563 | 0,52 | -0,788443417 | 7,671725977 | 13,95379481 | 0,000187359 | 0,001024077 |
| b4449 | 174 | 0,49 | 1,873330691 | 2,420405632 | 13,95028224 | 0,000187709 | 0,001024743 |
| b3644 | 864 | 0,54 | 0,874832422 | 7,142380197 | 13,94360728 | 0,000188377 | 0,001027137 |
| b3641 | 597 | 0,53 | 1,194880704 | 5,570799603 | 13,93589239 | 0,000189152 | 0,001030108 |
| b1627 | 582 | 0,52 | 1,400887499 | 3,984648124 | 13,9284876 | 0,000189898 | 0,001032919 |
| b3652 | 2082 | 0,57 | 0,761071046 | 6,58060379 | 13,8907608 | 0,000193749 | 0,001052583 |
| b2582 | 420 | 0,5 | 1,067821801 | 4,782976323 | 13,87450358 | 0,000195432 | 0,001060442 |
| b1829 | 882 | 0,54 | 0,831510509 | 8,067120787 | 13,86226197 | 0,000196709 | 0,001066082 |
| b3182 | 1434 | 0,53 | 1,090073412 | 5,015642761 | 13,83616654 | 0,00019946 | 0,001078798 |
| b1022 | 1326 | 0,47 | 2,057756061 | 3,024097329 | 13,83544178 | 0,000199537 | 0,001078798 |
| b1037 | 834 | 0,5 | 1,430936509 | 4,255576514 | 13,83188951 | 0,000199914 | 0,001079537 |
| b0926 | 549 | 0,5 | 1,253260737 | 6,962512287 | 13,79102794 | 0,00020431 | 0,001101944 |
| b4306 | 747 | 0,6 | 1,253893204 | 4,838590456 | 13,77313156 | 0,000206265 | 0,001111155 |
| b0333 | 1170 | 0,56 | 1,34147533 | 2,78681092 | 13,75675874 | 0,000208071 | 0,001119536 |
| b0405 | 1071 | 0,54 | 1,613741959 | 4,644687172 | 13,75222888 | 0,000208573 | 0,001120893 |
| b0162 | 1158 | 0,53 | -2,235919717 | 9,589629787 | 13,74567238 | 0,000209302 | 0,001123466 |
| b0060 | 2352 | 0,55 | -0,752941231 | 6,678316295 | 13,74331889 | 0,000209565 | 0,001123528 |
| b3599 | 1914 | 0,54 | -0,822505151 | 9,280657572 | 13,68217756 | 0,000216499 | 0,001159319 |
| b1165 | 273 | 0,37 | 1,641403807 | 3,638624327 | 13,66399467 | 0,000218606 | 0,001169203 |
| b4478 | 1149 | 0,53 | -1,11872786 | 4,145538817 | 13,63123234 | 0,000222454 | 0,001187281 |
| b1652 | 648 | 0,54 | 1,208021368 | 4,116794694 | 13,63070897 | 0,000222516 | 0,001187281 |
| b1757 | 1308 | 0,54 | -1,138319045 | 6,883881716 | 13,60402798 | 0,000225701 | 0,001202264 |
| b2202 | 603 | 0,52 | -1,763592288 | 6,592740585 | 13,60269995 | 0,00022586 | 0,001202264 |
| b2551 | 1254 | 0,53 | 1,794723561 | 10,23602255 | 13,59436725 | 0,000226865 | 0,00120618 |
| b4034 | 1191 | 0,51 | -1,761452376 | 10,87158646 | 13,57935395 | 0,000228687 | 0,001214425 |
| b2293 | 651 | 0,57 | -0,78929656 | 7,608053385 | 13,55454666 | 0,000231729 | 0,001229126 |
| b2949 | 417 | 0,53 | 1,139270296 | 4,446191742 | 13,53330174 | 0,000234367 | 0,001241648 |
| b0219 | 771 | 0,55 | -0,748675285 | 6,542612429 | 13,51243792 | 0,000236987 | 0,001254047 |
| b3601 | 588 | 0,51 | -0,992619737 | 7,590717927 | 13,50434508 | 0,000238012 | 0,001257012 |
| b2674 | 411 | 0,56 | 1,597287434 | 2,720930756 | 13,50357998 | 0,000238109 | 0,001257012 |
| b3338 | 2694 | 0,5 | -0,734306319 | 5,777607331 | 13,47928848 | 0,000241211 | 0,001271893 |
| b0742 | 792 | 0,49 | 0,828058834 | 8,240053119 | 13,46538411 | 0,000243005 | 0,001279439 |
| b1462 | 570 | 0,51 | -0,869851681 | 5,340129956 | 13,46195851 | 0,000243449 | 0,001279439 |
| b0307 | 1428 | 0,5 | -1,783021176 | 7,173282336 | 13,46157826 | 0,000243499 | 0,001279439 |
| b3646 | 618 | 0,53 | 1,296026105 | 3,982794913 | 13,45335209 | 0,000244569 | 0,001283557 |

| | | | | | | | |
|---|---|---|---|---|---|---|---|
| b2426 | 792 | 0,55 | -1,244784335 | 9,099874961 | 13,4485317 | 0,000245198 | 0,001285354 |
| b1865 | 453 | 0,53 | 0,748836192 | 6,326693043 | 13,43289751 | 0,00024725 | 0,001294596 |
| b0238 | 459 | 0,52 | 1,377212756 | 5,009988352 | 13,42109195 | 0,000248811 | 0,001301249 |
| b2942 | 1155 | 0,53 | 1,950864559 | 7,748871697 | 13,41531435 | 0,000249578 | 0,001303741 |
| b3155 | 303 | 0,54 | -1,063690691 | 4,525701411 | 13,39137421 | 0,000252784 | 0,00131895 |
| b1507 | 1323 | 0,48 | -0,89647722 | 6,223514194 | 13,36616301 | 0,000256205 | 0,001335245 |
| b1343 | 1374 | 0,52 | 0,846885623 | 5,628990952 | 13,3575622 | 0,000257383 | 0,00133924 |
| b1792 | 348 | 0,53 | -1,117270014 | 5,969075299 | 13,35620457 | 0,000257569 | 0,00133924 |
| b1045 | 534 | 0,52 | -0,962562014 | 6,217637823 | 13,34287946 | 0,000259406 | 0,001347226 |
| b3289 | 1290 | 0,5 | 0,827222172 | 7,003055045 | 13,33858787 | 0,00026 | 0,00134875 |
| b4020 | 1632 | 0,55 | -0,672234102 | 6,844052419 | 13,32831468 | 0,000261428 | 0,001354592 |
| b2166 | 942 | 0,47 | 1,506076179 | 2,56287899 | 13,32323318 | 0,000262138 | 0,001356699 |
| b3201 | 726 | 0,54 | 0,675406792 | 7,191860837 | 13,28165312 | 0,000268016 | 0,001384281 |
| b3188 | 279 | 0,5 | 1,403838708 | 2,707332059 | 13,28117219 | 0,000268085 | 0,001384281 |
| b1971 | 1005 | 0,52 | -1,016473242 | 6,560041845 | 13,24939289 | 0,000272668 | 0,001406328 |
| b0694 | 678 | 0,57 | -1,043691122 | 4,847855631 | 13,22175546 | 0,000276718 | 0,001425576 |
| b1646 | 522 | 0,53 | -1,196412622 | 7,848829133 | 13,210306 | 0,000278414 | 0,001432665 |
| b4252 | 453 | 0,5 | -0,890528249 | 5,997351729 | 13,17612163 | 0,000283539 | 0,001457364 |
| b4146 | 1029 | 0,53 | 1,00633702 | 5,582373075 | 13,16312895 | 0,000285512 | 0,001465823 |
| b2073 | 660 | 0,51 | 2,440058973 | 1,920630518 | 13,14822156 | 0,000287792 | 0,001475841 |
| b2833 | 219 | 0,43 | -0,725749998 | 6,483725733 | 13,12838545 | 0,000290855 | 0,001489844 |
| b3908 | 621 | 0,53 | 1,638809289 | 8,687912174 | 13,11987929 | 0,000292179 | 0,001494915 |
| b3160 | 1008 | 0,56 | 0,91973053 | 6,447314226 | 13,11633573 | 0,000292732 | 0,001496038 |
| b2522 | 777 | 0,47 | 0,964705619 | 5,660554871 | 13,10884613 | 0,000293905 | 0,00150032 |
| b1823 | 210 | 0,47 | 1,022674665 | 9,735767938 | 13,08451462 | 0,000297747 | 0,001518205 |
| b1588 | 2424 | 0,51 | -1,407448609 | 5,659509765 | 13,07764851 | 0,00029884 | 0,001522048 |
| b1262 | 1359 | 0,53 | -5,788207896 | 10,31569177 | 13,06289887 | 0,000301203 | 0,00153234 |
| b0430 | 615 | 0,53 | 1,434649686 | 9,526949745 | 13,05842639 | 0,000301923 | 0,001533445 |
| b4691 | 161 | 0,45 | 2,023632369 | 2,605714397 | 13,05730078 | 0,000302104 | 0,001533445 |
| b3822 | 1830 | 0,55 | 1,251725503 | 5,453485763 | 13,04732939 | 0,000303717 | 0,001539887 |
| b0316 | 933 | 0,49 | -1,035451599 | 4,4418746 | 13,0177572 | 0,000308551 | 0,001562628 |
| b3168 | 2673 | 0,53 | 0,861207344 | 10,76672303 | 12,98807524 | 0,000313481 | 0,001585804 |
| b4151 | 360 | 0,54 | -1,310200727 | 7,843088639 | 12,98185084 | 0,000314525 | 0,001588207 |
| b1279 | 309 | 0,5 | -0,950599286 | 6,006852278 | 12,9810183 | 0,000314665 | 0,001588207 |
| b3600 | 1149 | 0,52 | -0,742811413 | 8,61974383 | 12,96480525 | 0,000317401 | 0,001598713 |
| b0390 | 678 | 0,46 | 1,340213576 | 4,470132844 | 12,96446109 | 0,00031746 | 0,001598713 |
| b4175 | 1005 | 0,53 | 0,786157497 | 8,93082291 | 12,92012595 | 0,000325068 | 0,00163519 |
| b3451 | 846 | 0,54 | 1,766505756 | 4,683677176 | 12,91008279 | 0,000326817 | 0,001642145 |
| b1674 | 627 | 0,49 | -1,789998081 | 4,431943145 | 12,89123064 | 0,000330125 | 0,001656911 |
| b2250 | 543 | 0,53 | 1,335601322 | 4,075311917 | 12,85674485 | 0,000336265 | 0,001685839 |
| b0533 | 984 | 0,46 | 2,740562323 | 1,103386869 | 12,85164255 | 0,000337183 | 0,001688553 |
| b0968 | 279 | 0,52 | -0,989461403 | 5,902449161 | 12,83889427 | 0,000339488 | 0,001698199 |
| b1177 | 369 | 0,42 | 0,982350243 | 4,906299951 | 12,83320806 | 0,000340521 | 0,001701468 |
| b0465 | 3363 | 0,52 | 0,674942218 | 6,907580369 | 12,8168358 | 0,000343514 | 0,001714511 |
| b3164 | 2136 | 0,54 | 0,83704128 | 10,48199194 | 12,76350511 | 0,000353448 | 0,001762129 |
| b0947 | 1110 | 0,5 | 0,856561933 | 5,670481816 | 12,75504088 | 0,000355051 | 0,001768154 |
| b0607 | 429 | 0,5 | -1,281058889 | 9,781436363 | 12,74524601 | 0,000356915 | 0,001775465 |
| b1442 | 942 | 0,53 | -0,798532262 | 6,474451923 | 12,71713214 | 0,000362321 | 0,001800358 |
| b1204 | 585 | 0,5 | 1,316732624 | 4,529948782 | 12,69882372 | 0,000365886 | 0,001816057 |
| b3655 | 1710 | 0,54 | -0,746213186 | 7,174906153 | 12,69630606 | 0,000366379 | 0,001816492 |
| b3972 | 1029 | 0,46 | 0,680842788 | 6,497256246 | 12,68466143 | 0,000368667 | 0,00182582 |
| b0903 | 2283 | 0,51 | -1,08074574 | 12,35673177 | 12,67866938 | 0,000369851 | 0,001829659 |
| b0785 | 453 | 0,57 | -1,391206281 | 7,07806388 | 12,67269576 | 0,000371034 | 0,00183349 |
| b0174 | 762 | 0,5 | 0,837058535 | 7,133132023 | 12,66767577 | 0,000372032 | 0,001836394 |
| b4202 | 228 | 0,53 | 1,001890837 | 8,318769738 | 12,63867708 | 0,000377847 | 0,001863049 |
| b4566 | 258 | 0,5 | 1,509745114 | 3,314672879 | 12,62961214 | 0,000379684 | 0,001870048 |
| b3615 | 1035 | 0,46 | 1,777602619 | 2,168265926 | 12,60316952 | 0,000385093 | 0,001894608 |
| b0489 | 918 | 0,53 | -1,408287803 | 7,767758238 | 12,597676 | 0,000386227 | 0,001898101 |
| b1323 | 1542 | 0,52 | -0,821597192 | 8,072801171 | 12,57646208 | 0,000390636 | 0,001917665 |
| b0441 | 1872 | 0,52 | 0,84730265 | 8,572167031 | 12,56899858 | 0,000392199 | 0,001921304 |
| b2188 | 1761 | 0,52 | 0,645000323 | 6,75113549 | 12,56882945 | 0,000392234 | 0,001921304 |
| b3394 | 540 | 0,55 | 2,46499312 | 1,737263185 | 12,5642807 | 0,00039319 | 0,001923884 |
| b2244 | 900 | 0,48 | 0,980371837 | 4,346556526 | 12,55315987 | 0,000395537 | 0,001931793 |
| b1637 | 1275 | 0,52 | 0,718694516 | 8,259224847 | 12,55253852 | 0,000395668 | 0,001931793 |
| b2512 | 1179 | 0,53 | 0,787696926 | 8,672567991 | 12,51896079 | 0,000402843 | 0,001964681 |
| b4340 | 1413 | 0,55 | 0,950713679 | 4,758514658 | 12,49491423 | 0,000408061 | 0,001987969 |
| b0031 | 822 | 0,53 | 0,758080316 | 6,63617783 | 12,47489746 | 0,000412457 | 0,002007203 |
| b1296 | 1386 | 0,53 | 1,720201359 | 5,178360837 | 12,42012864 | 0,000424731 | 0,00206469 |
| b0156 | 345 | 0,51 | 0,923956212 | 6,549939438 | 12,37096971 | 0,000436061 | 0,002117471 |
| b3910 | 675 | 0,53 | -0,915187071 | 7,144584397 | 12,36382237 | 0,000437734 | 0,002122489 |
| b1668 | 1605 | 0,48 | -0,822847478 | 7,671300371 | 12,3622116 | 0,000438111 | 0,002122489 |
| b4485 | 1503 | 0,56 | -1,184841821 | 6,80895906 | 12,36049202 | 0,000438515 | 0,002122489 |
| b0783 | 486 | 0,56 | -1,42521551 | 7,583903237 | 12,32319722 | 0,000447364 | 0,002162982 |
| b1216 | 1101 | 0,49 | 1,025393869 | 4,782879166 | 12,31016165 | 0,000450499 | 0,002175793 |
| b1953 | 228 | 0,49 | -1,202231668 | 7,367038812 | 12,29905074 | 0,000453189 | 0,002186428 |
| b3281 | 819 | 0,49 | 0,795619733 | 6,22934302 | 12,2929021 | 0,000454684 | 0,002191284 |
| b3486 | 2736 | 0,57 | -0,873555965 | 8,278434983 | 12,29008465 | 0,000455371 | 0,002192238 |
| b3421 | 1227 | 0,51 | -1,047078659 | 5,2560189 | 12,28417258 | 0,000456816 | 0,002196833 |

| | | | | | | | |
|---|---|---|---|---|---|---|---|
| b0662 | 1176 | 0,57 | -1,147133747 | 8,442787558 | 12,2680662 | 0,000460776 | 0,0022135 |
| b1883 | 1050 | 0,54 | 2,294231515 | 1,671096012 | 12,26491236 | 0,000461555 | 0,00221487 |
| b1021 | 414 | 0,44 | 1,958247375 | 2,617362348 | 12,25189944 | 0,000464785 | 0,002227984 |
| b0576 | 1377 | 0,53 | 0,851929055 | 5,12643269 | 12,22093669 | 0,000472562 | 0,002262845 |
| b0882 | 2277 | 0,53 | -1,22462708 | 11,47573688 | 12,19734877 | 0,000478575 | 0,002289191 |
| b1215 | 855 | 0,52 | 0,759841726 | 8,138276916 | 12,18621023 | 0,000481441 | 0,002300446 |
| b3701 | 1101 | 0,54 | 0,652655566 | 7,348768372 | 12,16688475 | 0,000486455 | 0,002321928 |
| b1056 | 576 | 0,47 | -0,948901316 | 5,391923543 | 12,14579957 | 0,000491985 | 0,002345828 |
| b2321 | 996 | 0,52 | 1,061653458 | 4,586467814 | 12,14268376 | 0,000492808 | 0,002347254 |
| b0311 | 1671 | 0,57 | 1,10453715 | 5,816664183 | 12,13567147 | 0,000494665 | 0,002353595 |
| b2209 | 489 | 0,49 | -0,811913033 | 7,551339319 | 12,13039807 | 0,000496065 | 0,002357757 |
| b2733 | 2562 | 0,56 | 0,841587523 | 6,483544846 | 12,12259886 | 0,000498144 | 0,00236513 |
| b4127 | 273 | 0,41 | -0,819326788 | 7,004700877 | 12,10836213 | 0,000501962 | 0,002380734 |
| b0592 | 957 | 0,55 | 1,516703998 | 2,927409552 | 12,10079414 | 0,000504004 | 0,00238789 |
| b0834 | 1329 | 0,43 | 1,951941078 | 2,576828234 | 12,09398678 | 0,000505847 | 0,002394093 |
| b0163 | 387 | 0,44 | -0,696491777 | 8,293967567 | 12,09085761 | 0,000506697 | 0,002395585 |
| b1261 | 1194 | 0,55 | -4,964747999 | 11,21404181 | 12,0845023 | 0,000508427 | 0,002400803 |
| b2177 | 1815 | 0,51 | -0,791212067 | 6,203356994 | 12,0828714 | 0,000508872 | 0,002400803 |
| b3403 | 1623 | 0,54 | -1,176466405 | 11,76036563 | 12,07940251 | 0,000509819 | 0,002402745 |
| b2200 | 663 | 0,56 | -1,54822654 | 4,487500007 | 12,07578614 | 0,000510809 | 0,002404881 |
| b1245 | 909 | 0,52 | 1,002261525 | 9,451615662 | 12,06378845 | 0,000514107 | 0,002417868 |
| b3478 | 834 | 0,61 | -2,320595497 | 4,344575841 | 12,04381476 | 0,000519645 | 0,002441352 |
| b4547 | 201 | 0,48 | -1,129425706 | 5,611425866 | 12,02638956 | 0,000524526 | 0,002461701 |
| b4113 | 669 | 0,53 | -0,702357115 | 6,245857489 | 12,01413054 | 0,000527987 | 0,002475354 |
| b0074 | 1572 | 0,53 | 1,106364319 | 4,814588556 | 12,00959887 | 0,000529272 | 0,002478787 |
| b3704 | 360 | 0,56 | 1,632906699 | 5,163428996 | 11,99395341 | 0,000533734 | 0,002497075 |
| b3951 | 2298 | 0,53 | -0,949623017 | 4,961075004 | 11,98316907 | 0,000536832 | 0,002508948 |
| b1202 | 2868 | 0,5 | -0,893988899 | 5,093886404 | 11,97953001 | 0,000537881 | 0,002511234 |
| b2175 | 567 | 0,47 | 1,44485677 | 8,44194978 | 11,97374613 | 0,000539554 | 0,00251642 |
| b1260 | 807 | 0,54 | -4,764726983 | 10,64536199 | 11,96509014 | 0,000542066 | 0,002525509 |
| b0080 | 1005 | 0,55 | -0,768024072 | 8,141999629 | 11,92890037 | 0,000552698 | 0,00257237 |
| b1086 | 960 | 0,51 | 0,872107817 | 5,618811289 | 11,91955714 | 0,000555477 | 0,002582622 |
| b3829 | 2262 | 0,56 | -1,307682881 | 6,641321155 | 11,84679037 | 0,000577608 | 0,002682735 |
| b3149 | 591 | 0,5 | 0,703626552 | 6,200789005 | 11,81729989 | 0,000586829 | 0,002722741 |
| b0777 | 756 | 0,56 | 1,733043579 | 3,425947215 | 11,79742796 | 0,000593126 | 0,002749112 |
| b1872 | 2430 | 0,51 | -0,976890111 | 4,856908464 | 11,77142451 | 0,000601469 | 0,002784902 |
| b1529 | 666 | 0,51 | -0,741011919 | 5,67183836 | 11,75852874 | 0,000605651 | 0,002801369 |
| b4382 | 1323 | 0,55 | -0,870661509 | 8,625847282 | 11,74492972 | 0,000610092 | 0,00281669 |
| b1638 | 657 | 0,52 | 0,984402842 | 5,708877762 | 11,74453942 | 0,00061022 | 0,00281669 |
| b3506 | 567 | 0,46 | -1,247583203 | 10,65807876 | 11,70972727 | 0,000621742 | 0,002866923 |
| b3260 | 966 | 0,52 | 1,203274502 | 6,853012915 | 11,70662538 | 0,00062278 | 0,002868754 |
| b2452 | 1227 | 0,55 | -1,014213804 | 3,767202418 | 11,70160052 | 0,000624464 | 0,002873558 |
| b0397 | 3147 | 0,54 | 0,658187075 | 6,350436928 | 11,68238584 | 0,000630946 | 0,002900409 |
| b3390 | 522 | 0,49 | 0,844113163 | 7,155828411 | 11,64644658 | 0,000643253 | 0,002953955 |
| b3430 | 1296 | 0,53 | -1,039134491 | 9,899625283 | 11,63623079 | 0,000646795 | 0,002964958 |
| b0735 | 294 | 0,5 | -0,967452038 | 6,402334328 | 11,6357225 | 0,000646972 | 0,002964958 |
| b0119 | 363 | 0,52 | -1,028423925 | 7,542768255 | 11,63298016 | 0,000647927 | 0,002966299 |
| b2480 | 471 | 0,49 | 0,997031453 | 7,300492408 | 11,62032226 | 0,000652351 | 0,002983507 |
| b2435 | 870 | 0,5 | 0,991262275 | 5,62072587 | 11,60909582 | 0,000656301 | 0,002996873 |
| b1682 | 747 | 0,5 | -0,916907049 | 7,175292361 | 11,60821686 | 0,000656611 | 0,002996873 |
| b3880 | 1242 | 0,53 | -0,989819522 | 4,27438605 | 11,60602691 | 0,000657384 | 0,002997352 |
| b4114 | 1644 | 0,49 | -0,918059947 | 4,971501563 | 11,57836544 | 0,000667236 | 0,003039177 |
| b3739 | 381 | 0,52 | 1,075247043 | 6,750705431 | 11,5677859 | 0,000671043 | 0,003053415 |
| b2257 | 1653 | 0,51 | -0,914214079 | 5,068844121 | 11,55271745 | 0,000676503 | 0,003075139 |
| b3936 | 213 | 0,49 | 0,941855766 | 8,627159094 | 11,54687434 | 0,000678632 | 0,003081693 |
| b1990 | 933 | 0,5 | -1,011196218 | 7,913822355 | 11,53235463 | 0,000683953 | 0,003102711 |
| b1288 | 789 | 0,53 | 0,851998947 | 9,276257175 | 11,52036709 | 0,000688378 | 0,003119626 |
| b1902 | 504 | 0,48 | -0,849668251 | 6,958826939 | 11,50410843 | 0,000694425 | 0,003143853 |
| b2614 | 594 | 0,5 | 1,181790571 | 8,504481652 | 11,49080298 | 0,000699414 | 0,003163243 |
| b4402 | 141 | 0,43 | 0,758433221 | 6,791089588 | 11,48544794 | 0,000701432 | 0,003169172 |
| b0688 | 1641 | 0,55 | 0,815601523 | 8,983737998 | 11,47757048 | 0,000704412 | 0,003179428 |
| b1675 | 210 | 0,47 | -0,704293366 | 6,026836884 | 11,47081443 | 0,000706977 | 0,003187797 |
| b1309 | 1680 | 0,44 | 1,678771614 | 1,845153153 | 11,42199395 | 0,000725798 | 0,003269372 |
| b0683 | 447 | 0,48 | 0,933089364 | 7,640254299 | 11,40794179 | 0,000731308 | 0,003290886 |
| b0842 | 1233 | 0,52 | -0,878614281 | 5,317478918 | 11,38303983 | 0,000741177 | 0,003331952 |
| b3543 | 1020 | 0,56 | 1,377718376 | 7,96976143 | 11,35388558 | 0,000752903 | 0,003380426 |
| b2597 | 342 | 0,46 | -1,190868668 | 12,18040722 | 11,35249085 | 0,000753468 | 0,003380426 |
| b1864 | 741 | 0,52 | 0,748394492 | 7,99569642 | 11,34875604 | 0,000754985 | 0,003383844 |
| b3974 | 951 | 0,44 | 1,255716408 | 5,281304565 | 11,33591397 | 0,000760224 | 0,003403489 |
| b3144 | 2517 | 0,48 | -0,878994206 | 6,327993015 | 11,33276894 | 0,000761513 | 0,003403489 |
| b0915 | 987 | 0,55 | 0,682134093 | 5,826942451 | 11,33244276 | 0,000761646 | 0,003403489 |
| b0819 | 921 | 0,53 | 0,854805239 | 6,758650192 | 11,31465454 | 0,000768978 | 0,003432827 |
| b2747 | 711 | 0,55 | 1,074363646 | 5,693495506 | 11,30308665 | 0,000773783 | 0,003450843 |
| b4477 | 618 | 0,59 | -1,486492477 | 3,045088151 | 11,28613026 | 0,000780883 | 0,003479042 |
| b3643 | 716 | 0,57 | 1,239561608 | 4,645989616 | 11,28020303 | 0,00078338 | 0,003486702 |
| b1278 | 765 | 0,51 | -0,908516764 | 6,260979118 | 11,2767293 | 0,000784847 | 0,003487297 |
| b0393 | 912 | 0,52 | 0,954487721 | 5,761812037 | 11,27620279 | 0,00078507 | 0,003487297 |
| b4289 | 999 | 0,61 | 1,589028304 | 3,283839432 | 11,26232177 | 0,000790962 | 0,003509993 |

| | | | | | | | |
|---|---|---|---|---|---|---|---|
| b0902 | 741 | 0,5 | -0,699449822 | 7,082276281 | 11,21856119 | 0,000809833 | 0,003590178 |
| b3778 | 2022 | 0,53 | 0,654779735 | 5,965175763 | 11,21369458 | 0,000811959 | 0,003596048 |
| b4253 | 1815 | 0,32 | -1,040544982 | 3,136874892 | 11,19222737 | 0,000821407 | 0,0036343 |
| b0782 | 513 | 0,52 | -1,284637267 | 8,184618623 | 11,19002384 | 0,000822383 | 0,00363503 |
| b0998 | 600 | 0,53 | -1,307790809 | 4,850460353 | 11,17229429 | 0,000830279 | 0,003666315 |
| b0486 | 1293 | 0,53 | -1,002404027 | 9,446925552 | 11,11963957 | 0,000854184 | 0,003768161 |
| b3980 | 1185 | 0,53 | 1,211367901 | 10,80835909 | 11,11450448 | 0,000856552 | 0,003774893 |
| b1232 | 843 | 0,47 | 0,865078326 | 6,295219646 | 11,10723448 | 0,000859916 | 0,003785997 |
| b4316 | 726 | 0,44 | 1,756303228 | 3,574802302 | 11,08454619 | 0,000870501 | 0,003828839 |
| b2380 | 1698 | 0,51 | -0,818297621 | 5,751552403 | 11,08231432 | 0,00087155 | 0,003829691 |
| b4356 | 1362 | 0,52 | -0,848989612 | 4,673846526 | 11,07225567 | 0,00087629 | 0,003846751 |
| b0846 | 537 | 0,52 | -0,919101447 | 4,680469707 | 11,06264011 | 0,000880846 | 0,003862968 |
| b3810 | 708 | 0,56 | 0,740888335 | 6,294917624 | 11,05666857 | 0,000883688 | 0,003871641 |
| b3754 | 1428 | 0,49 | -1,253890728 | 5,45987742 | 11,0514638 | 0,000886172 | 0,003878734 |
| b0913 | 2265 | 0,49 | -0,852791057 | 4,955615679 | 11,04273528 | 0,000890354 | 0,003893236 |
| b0636 | 468 | 0,58 | 1,044337154 | 4,690775716 | 11,01398589 | 0,00090427 | 0,003947589 |
| b1209 | 624 | 0,51 | 1,000535253 | 6,245835216 | 11,01342061 | 0,000904546 | 0,003947589 |
| b0773 | 477 | 0,52 | 0,838092662 | 7,510992709 | 10,98257262 | 0,000919727 | 0,004009937 |
| b1116 | 1200 | 0,54 | 1,064493891 | 5,236419011 | 10,95768446 | 0,000932162 | 0,004060205 |
| b1134 | 462 | 0,5 | 0,934589691 | 4,492892892 | 10,95299238 | 0,000934526 | 0,004062835 |
| b0462 | 3150 | 0,54 | 0,749742131 | 9,651379778 | 10,95288704 | 0,000934579 | 0,004062835 |
| b0639 | 642 | 0,5 | 0,846957127 | 5,348163469 | 10,94917018 | 0,000936455 | 0,004067048 |
| b1287 | 1128 | 0,51 | 1,377603485 | 2,915181774 | 10,94043731 | 0,00094088 | 0,004082306 |
| b1534 | 1188 | 0,5 | 1,026008655 | 4,716187426 | 10,92848195 | 0,000946971 | 0,00410476 |
| b3925 | 1011 | 0,57 | -0,74117509 | 6,036620236 | 10,92408659 | 0,00094922 | 0,004110535 |
| b2771 | 1338 | 0,53 | 1,661767573 | 2,286572948 | 10,90188063 | 0,000960667 | 0,004156089 |
| b3469 | 2199 | 0,58 | -0,714901721 | 7,424838993 | 10,87566722 | 0,000974359 | 0,004210445 |
| b1718 | 543 | 0,49 | 0,781526548 | 10,5321823 | 10,87424125 | 0,00097511 | 0,004210445 |
| b3450 | 1071 | 0,58 | 1,020322936 | 6,657282202 | 10,85932778 | 0,000982994 | 0,004240402 |
| b3031 | 582 | 0,54 | -0,663922528 | 6,228468805 | 10,85369966 | 0,000985986 | 0,004247191 |
| b0155 | 1422 | 0,54 | -0,742405888 | 7,158035994 | 10,85280401 | 0,000986463 | 0,004247191 |
| b3552 | 660 | 0,58 | 1,201953213 | 3,404656638 | 10,84262473 | 0,0009919 | 0,004263132 |
| b3208 | 729 | 0,52 | -0,670449368 | 6,445473206 | 10,84108981 | 0,000992723 | 0,004263132 |
| b4389 | 1383 | 0,58 | 0,698455686 | 6,72335276 | 10,84053832 | 0,000993019 | 0,004263132 |
| b0808 | 2226 | 0,53 | -0,750732841 | 6,482890498 | 10,83710569 | 0,000994861 | 0,004266955 |
| b4512 | 198 | 0,52 | -0,814784497 | 5,741941616 | 10,82957219 | 0,000998917 | 0,004275857 |
| b0213 | 723 | 0,49 | -0,633150626 | 5,548766368 | 10,82946215 | 0,000998977 | 0,004275857 |
| b3356 | 405 | 0,52 | -0,588010887 | 6,811035495 | 10,82793879 | 0,000999799 | 0,004275857 |
| b2541 | 813 | 0,56 | 1,600950733 | 3,131889189 | 10,82018407 | 0,001003995 | 0,004287244 |
| b0439 | 2355 | 0,52 | 0,922222319 | 9,542230552 | 10,81948456 | 0,001004374 | 0,004287244 |
| b2350 | 363 | 0,46 | 2,082539376 | 4,101400981 | 10,80644027 | 0,001011476 | 0,00431345 |
| b1620 | 1029 | 0,54 | -0,855243449 | 4,991119039 | 10,80101315 | 0,001014446 | 0,004322001 |
| b3479 | 765 | 0,59 | -2,320314332 | 4,274634459 | 10,77545657 | 0,001028548 | 0,004377924 |
| b1164 | 237 | 0,42 | 2,158697547 | 3,454558537 | 10,76616467 | 0,001033725 | 0,004395783 |
| b1339 | 909 | 0,5 | 1,225392881 | 3,70210651 | 10,76031605 | 0,001036997 | 0,004405516 |
| b0184 | 3483 | 0,55 | 0,81155579 | 7,992178846 | 10,753565 | 0,001040786 | 0,004417036 |
| b3985 | 498 | 0,52 | 1,106390096 | 10,81523709 | 10,75197764 | 0,001041679 | 0,004417036 |
| b2659 | 978 | 0,54 | 1,139899625 | 9,469444469 | 10,74965171 | 0,001042989 | 0,004418411 |
| b2382 | 858 | 0,54 | -0,892255699 | 4,740438524 | 10,74030896 | 0,001048268 | 0,00443658 |
| b4234 | 552 | 0,53 | -0,743865918 | 7,786416153 | 10,73319254 | 0,001052307 | 0,004449473 |
| b2421 | 912 | 0,56 | 1,064926301 | 4,794744788 | 10,7232064 | 0,001058002 | 0,004469334 |
| b1612 | 1647 | 0,52 | 1,611881803 | 9,562212484 | 10,71859199 | 0,001060644 | 0,004476275 |
| b1832 | 498 | 0,49 | 0,954502583 | 6,97532133 | 10,71268201 | 0,001064037 | 0,004486371 |
| b1806 | 582 | 0,54 | 0,816226295 | 5,770579063 | 10,68170849 | 0,001082001 | 0,004557827 |
| b4324 | 774 | 0,54 | -0,801938073 | 6,292365365 | 10,67863518 | 0,0010838 | 0,004561119 |
| b4470 | 1311 | 0,57 | 1,084588651 | 6,640733075 | 10,66245723 | 0,00109332 | 0,004596869 |
| b0421 | 900 | 0,54 | 1,064622013 | 6,586972236 | 10,65342433 | 0,001098673 | 0,004615045 |
| b1705 | 192 | 0,51 | 1,290794496 | 2,727746111 | 10,63443903 | 0,001110009 | 0,004658298 |
| b4543 | 186 | 0,51 | 1,502355214 | 3,189642966 | 10,62301571 | 0,001116888 | 0,004682778 |
| b3259 | 882 | 0,54 | 0,758973088 | 5,7522289 | 10,59209625 | 0,001135721 | 0,004754183 |
| b1930 | 234 | 0,48 | 1,377305373 | 5,371625569 | 10,59157812 | 0,00113604 | 0,004754183 |
| b1100 | 798 | 0,51 | -0,78931857 | 7,265741747 | 10,57107693 | 0,001148708 | 0,004802713 |
| b0961 | 447 | 0,48 | 1,264624408 | 3,547411189 | 10,55610643 | 0,001158049 | 0,004837255 |
| b4153 | 735 | 0,54 | -1,244971554 | 9,336757785 | 10,54802367 | 0,001163124 | 0,00485393 |
| b1961 | 1419 | 0,52 | 0,815702062 | 5,847890157 | 10,51030288 | 0,001187108 | 0,004947792 |
| b2185 | 285 | 0,51 | 1,232852372 | 7,816699767 | 10,50918971 | 0,001187823 | 0,004947792 |
| b4220 | 1734 | 0,53 | -0,583649718 | 7,235615947 | 10,49425181 | 0,001197465 | 0,004982247 |
| b3130 | 465 | 0,44 | -0,779454463 | 5,882132232 | 10,49293573 | 0,001198318 | 0,004982247 |
| b3150 | 576 | 0,54 | 0,624028522 | 6,673987528 | 10,47479109 | 0,001210145 | 0,005026755 |
| b0338 | 900 | 0,55 | 1,017763318 | 3,626689249 | 10,47256466 | 0,001211604 | 0,005028157 |
| b0138 | 570 | 0,43 | 2,206585999 | 2,213921007 | 10,45711695 | 0,001221778 | 0,005065689 |
| b2268 | 918 | 0,51 | 1,079120521 | 4,230443548 | 10,45148301 | 0,00122551 | 0,005076467 |
| b2460 | 702 | 0,56 | -1,110230336 | 3,625748043 | 10,44713395 | 0,001228399 | 0,005083735 |
| b3335 | 678 | 0,5 | -0,885761733 | 4,8281543 | 10,44394054 | 0,001230525 | 0,005087834 |
| b2700 | 498 | 0,57 | 1,10915263 | 5,123423398 | 10,43930095 | 0,00123362 | 0,005095929 |
| b1080 | 1098 | 0,54 | 1,82097845 | 1,877864238 | 10,42417711 | 0,001243763 | 0,005133098 |
| b3102 | 987 | 0,51 | -0,781621135 | 7,788424808 | 10,42082124 | 0,001246025 | 0,005137703 |
| b1223 | 1392 | 0,54 | -2,903766581 | 11,59984629 | 10,41818843 | 0,001247802 | 0,005140303 |

| | | | | | | | |
|---|---|---|---|---|---|---|---|
| b1244 | 921 | 0,46 | 0,83286875 | 9,127816936 | 10,40793645 | 0,001254749 | 0,005164173 |
| b3507 | 531 | 0,4 | -0,940465569 | 5,67324816 | 10,39672657 | 0,001262389 | 0,005190851 |
| b4435 | 204 | 0,43 | 2,431092687 | 1,521812253 | 10,39385293 | 0,001264355 | 0,00519417 |
| b4233 | 1374 | 0,55 | 0,59075873 | 7,491423365 | 10,39116913 | 0,001266194 | 0,005196962 |
| b3811 | 897 | 0,54 | 0,907821825 | 6,303853936 | 10,38519409 | 0,001270298 | 0,005209037 |
| b4227 | 957 | 0,5 | -0,988373767 | 8,431784374 | 10,38194189 | 0,001272538 | 0,00521345 |
| b4418 | 169 | 0,38 | 1,135199218 | 3,877885941 | 10,35566511 | 0,00129078 | 0,005283355 |
| b2212 | 651 | 0,53 | -1,12316635 | 4,340968048 | 10,34254604 | 0,001299986 | 0,00531585 |
| b2779 | 1299 | 0,5 | -0,934365205 | 12,22550402 | 10,34027823 | 0,001301584 | 0,00531585 |
| b4475 | 1017 | 0,6 | -1,077343954 | 5,121914065 | 10,33929639 | 0,001302276 | 0,00531585 |
| b2897 | 267 | 0,49 | -0,701147243 | 6,821088017 | 10,32125396 | 0,001315069 | 0,005362279 |
| b1819 | 852 | 0,53 | -0,90949193 | 10,5550099 | 10,31988689 | 0,001316044 | 0,005362279 |
| b1920 | 801 | 0,5 | -0,853589107 | 8,831591294 | 10,29221925 | 0,001335923 | 0,005438336 |
| b0485 | 933 | 0,53 | -0,873302709 | 9,895052885 | 10,28177033 | 0,00134351 | 0,005464257 |
| b3077 | 450 | 0,46 | -1,537070726 | 4,536877927 | 10,26810459 | 0,001353498 | 0,005499889 |
| b2969 | 963 | 0,57 | 0,879732109 | 4,377837587 | 10,24044099 | 0,001373947 | 0,005577928 |
| b0906 | 693 | 0,48 | -0,629554967 | 5,949910076 | 10,22807417 | 0,00138319 | 0,005610369 |
| b3773 | 894 | 0,58 | -1,018705351 | 5,946410292 | 10,21679336 | 0,001391676 | 0,005639685 |
| b3495 | 435 | 0,49 | -1,162978782 | 10,72450649 | 10,212815 | 0,001394681 | 0,005646758 |
| b1122 | 462 | 0,44 | 2,564956486 | 1,184989624 | 10,20904585 | 0,001397535 | 0,005653204 |
| b3157 | 525 | 0,52 | -1,323122317 | 8,68837713 | 10,19689201 | 0,001406776 | 0,005685453 |
| b1822 | 810 | 0,51 | 1,122206303 | 4,345480831 | 10,16205314 | 0,001433609 | 0,00578868 |
| b2908 | 1326 | 0,54 | -0,816930766 | 9,114416746 | 10,14262896 | 0,001448794 | 0,005844729 |
| b2632 | 864 | 0,54 | -1,235406667 | 3,782090672 | 10,13550771 | 0,001454402 | 0,005862076 |
| b2770 | 780 | 0,56 | 1,557338326 | 2,335226774 | 10,13273981 | 0,001456587 | 0,005865611 |
| b2440 | 888 | 0,57 | -0,930616903 | 5,71897362 | 10,1283113 | 0,001460091 | 0,005874442 |
| b2665 | 450 | 0,48 | -0,927758064 | 9,913628513 | 10,12252267 | 0,001464684 | 0,005887635 |
| b0513 | 1302 | 0,51 | 1,349893056 | 2,82444362 | 10,12009552 | 0,001466614 | 0,005890111 |
| b3340 | 2115 | 0,51 | 1,062364611 | 13,06153466 | 10,10941702 | 0,001475136 | 0,005919033 |
| b1800 | 1086 | 0,54 | -1,021674617 | 5,771732764 | 10,10448836 | 0,001479087 | 0,005929576 |
| b0176 | 1353 | 0,51 | 0,644378935 | 8,108231501 | 10,08993865 | 0,001490811 | 0,005971236 |
| b2236 | 255 | 0,56 | 1,14611985 | 4,542478802 | 10,07618776 | 0,001501978 | 0,006010593 |
| b1495 | 2373 | 0,46 | 1,202847999 | 3,36468204 | 10,07294637 | 0,001504622 | 0,006015805 |
| b0095 | 1152 | 0,54 | -0,743479431 | 10,46702141 | 10,05225553 | 0,001521615 | 0,006078324 |
| b1020 | 1065 | 0,52 | 1,340340425 | 7,110406683 | 10,0437545 | 0,001528653 | 0,006101001 |
| b3693 | 879 | 0,54 | -0,818742108 | 4,374790629 | 10,04093377 | 0,001530996 | 0,006104914 |
| b2137 | 762 | 0,55 | -0,767026201 | 6,510382938 | 10,02447562 | 0,001544736 | 0,00615423 |
| b4176 | 198 | 0,49 | 2,652144212 | 1,692697382 | 10,01067553 | 0,001556354 | 0,006195009 |
| b2495 | 360 | 0,52 | 0,79660079 | 5,880586894 | 9,969517457 | 0,001591532 | 0,006329411 |
| b1174 | 267 | 0,43 | 0,751869701 | 6,601738976 | 9,963641816 | 0,001596619 | 0,006344013 |
| b4180 | 732 | 0,56 | -0,629266681 | 6,336151564 | 9,956273443 | 0,001603022 | 0,006363813 |
| b0859 | 1128 | 0,55 | 0,99974942 | 3,864539462 | 9,909718751 | 0,001644082 | 0,006521042 |
| b3780 | 1266 | 0,53 | 0,631994978 | 8,081851319 | 9,893877587 | 0,001658296 | 0,006571601 |
| b0757 | 1149 | 0,53 | -0,835765306 | 8,817229554 | 9,875941517 | 0,001674539 | 0,006625906 |
| b1840 | 873 | 0,53 | 0,852525141 | 6,121310812 | 9,875483562 | 0,001674956 | 0,006625906 |
| b3804 | 741 | 0,53 | -0,542385411 | 7,119454944 | 9,870942722 | 0,001679095 | 0,006636421 |
| b4326 | 393 | 0,49 | -1,551618123 | 3,714775396 | 9,866734398 | 0,00168294 | 0,006644061 |
| b0427 | 1365 | 0,53 | 0,864335127 | 5,04570731 | 9,865583837 | 0,001683992 | 0,006644061 |
| b3812 | 717 | 0,56 | 0,982568756 | 5,145909209 | 9,851918691 | 0,001696547 | 0,006687709 |
| b0493 | 810 | 0,54 | 0,727353567 | 5,297841665 | 9,847745638 | 0,0017004 | 0,006697006 |
| b3094 | 777 | 0,51 | -0,825140895 | 7,001137744 | 9,826925057 | 0,001719756 | 0,006767293 |
| b3559 | 2070 | 0,55 | 0,775219217 | 9,880832105 | 9,797710635 | 0,001747293 | 0,006869619 |
| b4473 | 1125 | 0,51 | -0,685003099 | 7,024444683 | 9,739205807 | 0,001803788 | 0,00708552 |
| b3909 | 984 | 0,53 | -0,86317711 | 4,087624481 | 9,713544124 | 0,001829149 | 0,00717885 |
| b3542 | 903 | 0,59 | 1,047518091 | 7,876069647 | 9,707078722 | 0,001835596 | 0,007197848 |
| b1241 | 2676 | 0,51 | -0,813920708 | 11,66601905 | 9,699835332 | 0,001842845 | 0,007219958 |
| b1593 | 696 | 0,52 | -1,786702159 | 8,246478847 | 9,695201552 | 0,001847498 | 0,007231866 |
| b0831 | 921 | 0,54 | -0,766305082 | 5,098701617 | 9,689916638 | 0,001852819 | 0,007242428 |
| b2128 | 732 | 0,58 | 0,939175533 | 4,960160541 | 9,689313141 | 0,001853428 | 0,007242428 |
| b3650 | 2109 | 0,54 | 0,748802766 | 8,087708758 | 9,680126247 | 0,001862718 | 0,00727239 |
| b0471 | 330 | 0,53 | 0,603872762 | 7,276236647 | 9,662148858 | 0,001881035 | 0,007337509 |
| b3779 | 1485 | 0,54 | 0,551131642 | 6,963539317 | 9,657629328 | 0,001885668 | 0,007349186 |
| b1205 | 279 | 0,53 | -1,688004131 | 10,04947724 | 9,652371647 | 0,001891073 | 0,007363847 |
| b0689 | 495 | 0,45 | 1,717103709 | 1,684197338 | 9,645411562 | 0,001898252 | 0,007385385 |
| b3645 | 825 | 0,46 | -0,803959559 | 4,780002219 | 9,578816874 | 0,001968352 | 0,007651478 |
| b2239 | 1077 | 0,46 | -0,697028191 | 5,91561528 | 9,568632553 | 0,001979302 | 0,007687374 |
| b1242 | 648 | 0,48 | 0,917127826 | 4,145819002 | 9,549147505 | 0,002000423 | 0,007758478 |
| b0240 | 1178 | 0,5 | 1,046875352 | 7,432264996 | 9,548553038 | 0,002001071 | 0,007758478 |
| b0594 | 1611 | 0,56 | 1,805760072 | 3,245724683 | 9,525506279 | 0,002026357 | 0,007849724 |
| b0814 | 516 | 0,51 | 0,926292637 | 10,29043284 | 9,523177984 | 0,002028929 | 0,007852512 |
| b1050 | 228 | 0,5 | -0,977537834 | 8,588368097 | 9,521685172 | 0,00203058 | 0,007852512 |
| b3249 | 489 | 0,51 | 0,739211986 | 4,883874074 | 9,519194509 | 0,002033338 | 0,007856398 |
| b4136 | 1698 | 0,54 | 0,762925194 | 6,048417595 | 9,511105967 | 0,00204232 | 0,007884306 |
| b1010 | 387 | 0,54 | 2,519726238 | 1,251498585 | 9,490020137 | 0,002065926 | 0,00796857 |
| b2152 | 1158 | 0,54 | 1,04586242 | 4,457148918 | 9,485109876 | 0,002071462 | 0,007983055 |
| b3049 | 201 | 0,45 | -1,159088644 | 9,19028205 | 9,435421008 | 0,002128342 | 0,008195215 |
| b0472 | 606 | 0,58 | 0,725057417 | 6,899363912 | 9,417161474 | 0,00214964 | 0,008270118 |
| b1583 | 342 | 0,51 | -0,692769033 | 5,951902443 | 9,414098305 | 0,002153234 | 0,00827684 |

| | | | | | | | |
|---|---|---|---|---|---|---|---|
| b3375 | 732 | 0,51 | -0,87007149 | 5,101593233 | 9,400774561 | 0,002168938 | 0,00833006 |
| b2107 | 339 | 0,5 | 0,90552959 | 4,992977775 | 9,392465765 | 0,00217879 | 0,008360732 |
| b2414 | 972 | 0,5 | 0,821323847 | 8,277459147 | 9,342371974 | 0,002239155 | 0,008585024 |
| b3158 | 996 | 0,57 | -1,446604939 | 5,710600468 | 9,32576686 | 0,002259537 | 0,008655766 |
| b0444 | 696 | 0,52 | 0,641704291 | 5,692438786 | 9,314515811 | 0,002273455 | 0,008701642 |
| b4192 | 1065 | 0,52 | -1,079941502 | 4,116750096 | 9,309955087 | 0,002279121 | 0,008715886 |
| b2800 | 648 | 0,49 | -0,923193966 | 4,445253735 | 9,307280157 | 0,002282451 | 0,00872118 |
| b4573 | 897 | 0,55 | 1,380299515 | 2,705172838 | 9,284963963 | 0,002310425 | 0,008820548 |
| b3009 | 660 | 0,51 | -0,801762966 | 6,624774233 | 9,282678813 | 0,002313309 | 0,008824042 |
| b0025 | 942 | 0,54 | 0,727179307 | 7,129915368 | 9,25617392 | 0,002347028 | 0,008945052 |
| b2373 | 1695 | 0,47 | 1,367367484 | 2,149080003 | 9,244702974 | 0,002361776 | 0,008993611 |
| b4592 | 93 | 0,46 | 1,844936813 | 2,248735059 | 9,237794103 | 0,002370704 | 0,009019944 |
| b2235 | 1131 | 0,51 | 0,748764372 | 7,669392312 | 9,223388426 | 0,002389429 | 0,00908215 |
| b3348 | 219 | 0,53 | 0,813712486 | 4,583978177 | 9,220970371 | 0,002392587 | 0,00908215 |
| b2812 | 807 | 0,55 | 0,793000949 | 5,263273165 | 9,220553149 | 0,002393132 | 0,00908215 |
| b2044 | 1221 | 0,55 | 1,356818283 | 2,645930508 | 9,198760006 | 0,002421791 | 0,009183137 |
| b2051 | 480 | 0,55 | 2,133815241 | 1,217731061 | 9,191653342 | 0,002431212 | 0,009211067 |
| b0724 | 717 | 0,52 | 1,336043494 | 10,34604763 | 9,186345477 | 0,002438272 | 0,009230014 |
| b0068 | 984 | 0,53 | 1,012154729 | 4,03756849 | 9,17646617 | 0,002451469 | 0,009272138 |
| b4017 | 2187 | 0,38 | 1,203310971 | 4,776701171 | 9,159042243 | 0,002474921 | 0,009352946 |
| b0679 | 1947 | 0,55 | -0,826130274 | 9,317017038 | 9,154762485 | 0,002480716 | 0,009366949 |
| b1376 | 435 | 0,49 | -1,009195975 | 9,661592915 | 9,143912869 | 0,002495469 | 0,009414723 |
| b4159 | 3324 | 0,56 | 0,597605038 | 6,51075454 | 9,128194844 | 0,002516999 | 0,009487543 |
| b3397 | 561 | 0,51 | 1,166511159 | 5,293588203 | 9,126738395 | 0,002519004 | 0,009487543 |
| b0328 | 672 | 0,49 | -1,014759188 | 4,383786267 | 9,114083032 | 0,002536491 | 0,009545383 |
| b4160 | 969 | 0,53 | 0,570310394 | 7,07172528 | 9,083632829 | 0,002579072 | 0,009697484 |
| b1436 | 231 | 0,49 | 1,610189242 | 2,34177184 | 9,066383695 | 0,002603514 | 0,009781183 |
| b3539 | 1272 | 0,45 | 0,946999218 | 5,986377114 | 9,036433906 | 0,002646514 | 0,009934401 |
| b3107 | 165 | 0,52 | 0,918111984 | 4,235212662 | 9,01750653 | 0,002674059 | 0,010029401 |
| b0250 | 468 | 0,53 | -0,848518151 | 5,537381652 | 9,010142704 | 0,002684855 | 0,01006147 |
| b0101 | 198 | 0,52 | 0,905867522 | 4,274818848 | 8,981881033 | 0,002726698 | 0,010209742 |
| b3923 | 429 | 0,43 | -0,836977642 | 8,168879079 | 8,96655334 | 0,002749668 | 0,010287156 |
| b4112 | 1092 | 0,54 | -0,779550647 | 5,704542045 | 8,953787003 | 0,00276895 | 0,010350653 |
| b1129 | 1461 | 0,51 | -0,560938403 | 7,032842431 | 8,951697607 | 0,002772119 | 0,010353863 |
| b3740 | 624 | 0,49 | 0,873789445 | 4,713638473 | 8,941188673 | 0,002788112 | 0,010404929 |
| b0301 | 594 | 0,45 | 1,692859127 | 1,800690739 | 8,928853417 | 0,002807005 | 0,010466719 |
| b1413 | 3903 | 0,53 | -0,645896737 | 8,68780842 | 8,897863311 | 0,002855045 | 0,010637002 |
| b1217 | 231 | 0,45 | -0,770860031 | 5,119650706 | 8,88972248 | 0,002867803 | 0,010675659 |
| b2199 | 738 | 0,55 | -1,16334765 | 5,39110054 | 8,883127565 | 0,002878181 | 0,010705399 |
| b2913 | 1233 | 0,53 | 0,783907128 | 7,405959612 | 8,876686052 | 0,002888354 | 0,010734329 |
| b3789 | 882 | 0,55 | 0,615101015 | 6,260475975 | 8,864758809 | 0,002907287 | 0,01079574 |
| b0364 | 558 | 0,43 | 2,775109265 | 0,908702253 | 8,830009406 | 0,002963169 | 0,01099414 |
| b1604 | 945 | 0,51 | 0,736710429 | 7,56987236 | 8,818577003 | 0,002981791 | 0,01105036 |
| b3651 | 690 | 0,55 | 0,801436563 | 5,32777461 | 8,817143792 | 0,002984134 | 0,01105036 |
| b1428 | 981 | 0,48 | -0,873511946 | 6,822904694 | 8,815241692 | 0,002987246 | 0,01105036 |
| b3838 | 516 | 0,55 | -0,652489871 | 7,521507661 | 8,814669656 | 0,002988183 | 0,01105036 |
| b3617 | 1197 | 0,54 | -0,76283985 | 10,19079119 | 8,786968447 | 0,0030339 | 0,011205619 |
| b4258 | 2856 | 0,55 | 0,723330425 | 9,798924112 | 8,786206421 | 0,003035168 | 0,011205619 |
| b1291 | 993 | 0,51 | -0,651747746 | 6,435764547 | 8,780648243 | 0,00304443 | 0,011229999 |
| b2011 | 1428 | 0,5 | -0,693051311 | 6,653039394 | 8,779239268 | 0,003046782 | 0,011229999 |
| b4237 | 465 | 0,51 | -1,461621668 | 3,956947653 | 8,771962325 | 0,003058962 | 0,011265626 |
| b3468 | 627 | 0,57 | -0,684507637 | 6,301324552 | 8,745758741 | 0,00310323 | 0,011419273 |
| b3358 | 2103 | 0,53 | 0,660906243 | 6,471019428 | 8,740418552 | 0,003112331 | 0,011443369 |
| b2660 | 1269 | 0,58 | 0,963893705 | 9,121869145 | 8,73544439 | 0,003120832 | 0,011465222 |
| b3072 | 1521 | 0,55 | 0,873785304 | 4,668155329 | 8,727254068 | 0,003134883 | 0,011507407 |
| b1858 | 756 | 0,49 | -0,807836906 | 5,299556611 | 8,714662981 | 0,003156608 | 0,011577672 |
| b0157 | 624 | 0,54 | 0,880837682 | 3,66145827 | 8,708464497 | 0,003167359 | 0,011607606 |
| b2302 | 648 | 0,51 | -0,676150662 | 6,613997244 | 8,703931544 | 0,003175245 | 0,011626999 |
| b3429 | 1434 | 0,55 | -0,786862614 | 9,589656741 | 8,699883354 | 0,003182304 | 0,011643336 |
| b2465 | 2004 | 0,55 | -0,910225956 | 10,78373257 | 8,677055397 | 0,003222412 | 0,011780466 |
| b2701 | 1086 | 0,55 | 0,899274276 | 5,575368009 | 8,671928823 | 0,00323149 | 0,011804024 |
| b4232 | 999 | 0,51 | 0,591280286 | 8,224457415 | 8,665745139 | 0,003242474 | 0,011834502 |
| b0525 | 495 | 0,48 | 0,744675248 | 8,685750774 | 8,657660908 | 0,003256891 | 0,011875357 |
| b2952 | 567 | 0,51 | 0,706398051 | 5,465388677 | 8,656499985 | 0,003258967 | 0,011875357 |
| b1235 | 1014 | 0,49 | -0,692820239 | 7,80683214 | 8,613776072 | 0,003336299 | 0,012147273 |
| b2225 | 777 | 0,53 | 1,434261116 | 2,445348643 | 8,60861224 | 0,003345771 | 0,012171873 |
| b0389 | 192 | 0,48 | -0,714175563 | 6,4181121 | 8,601645542 | 0,003358594 | 0,012200159 |
| b4366 | 678 | 0,45 | 1,507959254 | 1,966094774 | 8,601430424 | 0,003358991 | 0,012200159 |
| b1363 | 1458 | 0,38 | 1,116841248 | 3,924719808 | 8,599280634 | 0,003362958 | 0,012204679 |
| b0836 | 384 | 0,49 | -1,864602964 | 8,564019202 | 8,596251737 | 0,003368556 | 0,012215103 |
| b3548 | 699 | 0,5 | -0,622020639 | 7,418770232 | 8,562472169 | 0,003431631 | 0,012433768 |
| b2714 | 1011 | 0,53 | 0,83148578 | 5,146699991 | 8,542674233 | 0,003469156 | 0,012559577 |
| b3142 | 585 | 0,45 | 1,543818927 | 2,142991416 | 8,538654739 | 0,003476825 | 0,012577183 |
| b1782 | 747 | 0,52 | 1,002791614 | 8,331935238 | 8,533860249 | 0,003485996 | 0,012591062 |
| b1423 | 1344 | 0,53 | -0,771345338 | 8,423513388 | 8,533711586 | 0,00348628 | 0,012591062 |
| b3981 | 384 | 0,55 | 0,655424111 | 7,355334469 | 8,520662344 | 0,003511367 | 0,012665597 |
| b2943 | 1395 | 0,53 | 0,843778214 | 6,286944279 | 8,520039039 | 0,00351257 | 0,012665597 |
| b2149 | 1521 | 0,45 | 2,704746941 | 7,57299067 | 8,508662915 | 0,003534597 | 0,012734777 |

| | | | | | | | |
|---|---|---|---|---|---|---|---|
| b3512 | 528 | 0,33 | -1,257234643 | 9,569141951 | 8,506855783 | 0,003538109 | 0,012737192 |
| b2519 | 2313 | 0,57 | -0,713806537 | 5,018565799 | 8,492178643 | 0,003566764 | 0,012830047 |
| b2477 | 1035 | 0,55 | 0,624568722 | 8,394928893 | 8,490509899 | 0,003570037 | 0,012831522 |
| b4453 | 108 | 0,53 | 1,918187331 | 1,675705838 | 8,487129901 | 0,003576676 | 0,012845082 |
| b0276 | 828 | 0,49 | -1,226067597 | 4,843274094 | 8,47421962 | 0,003602149 | 0,012921058 |
| b2448 | 378 | 0,43 | 1,449250908 | 2,369320137 | 8,47348833 | 0,003603597 | 0,012921058 |
| b0401 | 1320 | 0,56 | -0,712307614 | 7,391284808 | 8,471226658 | 0,00360808 | 0,012926792 |
| b0248 | 459 | 0,56 | 0,849228875 | 3,989238785 | 8,467281295 | 0,003615915 | 0,012944512 |
| b2593 | 732 | 0,55 | 0,592546378 | 6,064515683 | 8,464430048 | 0,003621587 | 0,012954472 |
| b3055 | 621 | 0,53 | 1,331279827 | 5,397958013 | 8,430532124 | 0,003689724 | 0,013187672 |
| b2343 | 285 | 0,52 | -1,017741472 | 7,98246176 | 8,420805444 | 0,003709514 | 0,013247843 |
| b1863 | 522 | 0,56 | 0,646615317 | 5,963485107 | 8,412813476 | 0,003725856 | 0,013294601 |
| b4145 | 870 | 0,45 | 0,916647859 | 4,145017546 | 8,411504749 | 0,003728539 | 0,013294601 |
| b2445 | 369 | 0,47 | 1,826499331 | 1,484917059 | 8,401807851 | 0,00374848 | 0,013355079 |
| b3476 | 1575 | 0,53 | -1,978533396 | 6,86467463 | 8,394117543 | 0,003764372 | 0,013399631 |
| b2217 | 651 | 0,48 | 0,507843401 | 7,992667586 | 8,392866233 | 0,003766964 | 0,013399631 |
| b0273 | 1005 | 0,59 | 1,269339632 | 4,566361945 | 8,380063648 | 0,003793591 | 0,013483645 |
| b0435 | 318 | 0,48 | -0,907779167 | 9,489702852 | 8,375310481 | 0,003803526 | 0,013508243 |
| b0715 | 1047 | 0,56 | -1,139313643 | 4,722718484 | 8,373034774 | 0,003808291 | 0,013514459 |
| b1925 | 411 | 0,5 | 2,07930304 | 1,345301038 | 8,35822502 | 0,003839454 | 0,013614267 |
| b4322 | 1185 | 0,54 | -0,620961568 | 6,389537022 | 8,343106294 | 0,003871535 | 0,013717169 |
| b0281 | 1401 | 0,44 | 0,575819925 | 6,266196333 | 8,335925086 | 0,003886868 | 0,013760618 |
| b2937 | 921 | 0,55 | -0,772993794 | 8,070462037 | 8,317464839 | 0,003926568 | 0,013890197 |
| b4290 | 903 | 0,58 | 1,229904685 | 4,257217425 | 8,305827692 | 0,003951807 | 0,013968453 |
| b4039 | 498 | 0,53 | 0,602502244 | 6,065620724 | 8,303875831 | 0,003956056 | 0,013972454 |
| b0090 | 1068 | 0,56 | -0,597477189 | 7,504682017 | 8,289467799 | 0,003987567 | 0,014072659 |
| b0546 | 798 | 0,36 | -0,816644314 | 4,733376521 | 8,282369987 | 0,004003184 | 0,014116658 |
| b3190 | 255 | 0,51 | 1,483858332 | 4,429129768 | 8,271681767 | 0,004026819 | 0,014188838 |
| b3755 | 693 | 0,49 | -0,761001053 | 6,956529269 | 8,268306692 | 0,004034312 | 0,01419391 |
| b2347 | 933 | 0,49 | -0,739187992 | 5,720460053 | 8,268180502 | 0,004034592 | 0,01419391 |
| b0097 | 513 | 0,54 | -0,613744897 | 6,492931654 | 8,256740956 | 0,004060095 | 0,01427088 |
| b3742 | 444 | 0,5 | 0,863162053 | 6,080218056 | 8,254742473 | 0,004064567 | 0,01427088 |
| b0923 | 705 | 0,52 | 0,538240977 | 6,359810417 | 8,254092544 | 0,004066023 | 0,01427088 |
| b4406 | 201 | 0,51 | -0,602963798 | 6,204630361 | 8,246465249 | 0,004083144 | 0,014319757 |
| b2554 | 1335 | 0,55 | 0,813965684 | 5,344328726 | 8,240977096 | 0,004095508 | 0,014351889 |
| b0633 | 1089 | 0,57 | 0,613840033 | 7,393609254 | 8,238577745 | 0,004100925 | 0,014359647 |
| b4266 | 765 | 0,49 | -1,031427484 | 3,410590187 | 8,230645481 | 0,004118888 | 0,014411284 |
| b0229 | 1713 | 0,58 | -0,843225725 | 3,994190139 | 8,219622819 | 0,004143981 | 0,014487772 |
| b2015 | 930 | 0,5 | -1,005747486 | 5,982860121 | 8,212905745 | 0,004159349 | 0,014522644 |
| b3402 | 1725 | 0,5 | -0,582805925 | 5,685884164 | 8,212431611 | 0,004160436 | 0,014522644 |
| b2211 | 1644 | 0,53 | 1,149208637 | 4,149374039 | 8,210465873 | 0,004164946 | 0,014527071 |
| b0871 | 1719 | 0,54 | -0,799091888 | 9,683433045 | 8,208071429 | 0,004170445 | 0,014534943 |
| b4330 | 684 | 0,53 | 2,214946106 | 1,333601407 | 8,204918772 | 0,004177698 | 0,014548906 |
| b0088 | 1317 | 0,55 | -0,639277326 | 8,403218853 | 8,202270094 | 0,004183801 | 0,014558848 |
| b2699 | 1062 | 0,54 | 0,610429741 | 9,155679725 | 8,160015015 | 0,004282402 | 0,014890399 |
| b0212 | 756 | 0,4 | -0,575528802 | 6,208329962 | 8,154245453 | 0,004296047 | 0,014926266 |
| b0750 | 1044 | 0,5 | -0,757569119 | 4,719857953 | 8,151855578 | 0,004301712 | 0,014934372 |
| b2307 | 717 | 0,5 | 1,244525731 | 3,94172949 | 8,148948309 | 0,004308614 | 0,014946756 |
| b3257 | 243 | 0,54 | 1,745888393 | 1,543199163 | 8,140022119 | 0,004329876 | 0,015008896 |
| b4557 | 258 | 0,55 | 1,72927652 | 3,598548127 | 8,134986986 | 0,004341916 | 0,015039001 |
| b1983 | 717 | 0,4 | 0,940506212 | 5,955986999 | 8,130531856 | 0,004352598 | 0,015064358 |
| b3452 | 888 | 0,56 | 1,651569266 | 4,207237761 | 8,06989518 | 0,004500672 | 0,015562775 |
| b4359 | 2292 | 0,52 | -0,540186068 | 7,406575618 | 8,068736201 | 0,004503552 | 0,015562775 |
| b0962 | 2055 | 0,53 | -0,562370406 | 7,76734246 | 8,056434151 | 0,004534234 | 0,01565673 |
| b1969 | 672 | 0,45 | 0,680710601 | 4,7054904 | 8,042102187 | 0,004570247 | 0,015768935 |
| b1200 | 1071 | 0,53 | -0,809353226 | 6,675858825 | 8,037150445 | 0,004582757 | 0,015799937 |
| b1999 | 552 | 0,58 | -0,987105581 | 3,558898163 | 8,017303677 | 0,00463325 | 0,015961742 |
| b1308 | 315 | 0,51 | -0,85729516 | 7,853107695 | 8,015637513 | 0,004637515 | 0,015964163 |
| b2893 | 711 | 0,48 | 0,544785545 | 6,554767768 | 8,013029071 | 0,004644199 | 0,015974904 |
| b4019 | 3684 | 0,56 | -0,603502559 | 8,22484542 | 8,010975026 | 0,00464947 | 0,015980769 |
| b2547 | 1512 | 0,56 | -0,861105765 | 3,509609589 | 7,997242368 | 0,004684865 | 0,016090086 |
| b3827 | 900 | 0,56 | 0,955304437 | 3,716993541 | 7,983685099 | 0,004720076 | 0,016198609 |
| b3223 | 690 | 0,58 | -0,754617306 | 4,932099356 | 7,963955008 | 0,004771803 | 0,016363598 |
| b0752 | 942 | 0,53 | -0,677095403 | 5,83326841 | 7,958582623 | 0,004785988 | 0,016394921 |
| b1707 | 714 | 0,42 | -0,925558709 | 4,697971127 | 7,956889 | 0,004790468 | 0,016394921 |
| b4267 | 1032 | 0,47 | -0,759118749 | 3,66659702 | 7,955235867 | 0,004794846 | 0,016394921 |
| b0695 | 2685 | 0,58 | -0,736954225 | 7,489016562 | 7,954962916 | 0,004795569 | 0,016394921 |
| b2342 | 1311 | 0,56 | 0,976945696 | 6,072803881 | 7,945166077 | 0,004821599 | 0,016471348 |
| b2803 | 1449 | 0,51 | -1,003804747 | 5,402864179 | 7,935441883 | 0,004847579 | 0,016547485 |
| b3616 | 1026 | 0,51 | -0,727001099 | 10,19699539 | 7,926864172 | 0,004870614 | 0,016602929 |
| b2299 | 543 | 0,53 | 0,543899051 | 6,507501543 | 7,926635338 | 0,00487123 | 0,016602929 |
| b3594 | 843 | 0,34 | -0,995271559 | 3,900485052 | 7,922225739 | 0,004883116 | 0,016630796 |
| b1236 | 909 | 0,47 | 0,581072645 | 8,725450302 | 7,917407653 | 0,004896138 | 0,016662483 |
| b2111 | 543 | 0,41 | -1,614608353 | 3,459130519 | 7,896029641 | 0,004954343 | 0,016835902 |
| b0985 | 747 | 0,52 | 1,377916167 | 1,652262376 | 7,895932614 | 0,004954608 | 0,016835902 |
| b1532 | 219 | 0,5 | 1,447183573 | 2,73628563 | 7,875341069 | 0,005011342 | 0,017015784 |
| b1818 | 801 | 0,52 | -0,794140587 | 10,37614273 | 7,870480315 | 0,00502483 | 0,017048668 |
| b1218 | 696 | 0,55 | -0,590348566 | 6,297217074 | 7,849204308 | 0,005084308 | 0,017237419 |

| | | | | | | | |
|---|---|---|---|---|---|---|---|
| b1266 | 882 | 0,54 | -0,593063941 | 6,183220473 | 7,846652793 | 0,005091489 | 0,017248717 |
| b1919 | 987 | 0,54 | -0,56513348 | 7,003849505 | 7,835074658 | 0,005124204 | 0,017341801 |
| b0556 | 462 | 0,51 | 1,572987705 | 2,432730282 | 7,834192917 | 0,005126704 | 0,017341801 |
| b3630 | 798 | 0,43 | 0,68069063 | 5,269527154 | 7,812562956 | 0,005188427 | 0,017537352 |
| b4053 | 1080 | 0,56 | 0,6136032 | 5,926041721 | 7,807668768 | 0,005202498 | 0,017571661 |
| b3863 | 2787 | 0,52 | -0,609687085 | 9,319377827 | 7,79060039 | 0,005251874 | 0,017725075 |
| b2135 | 588 | 0,54 | -1,247913619 | 6,908347119 | 7,781050334 | 0,005279709 | 0,017805611 |
| b0026 | 2817 | 0,56 | 0,674205743 | 10,0391352 | 7,778465561 | 0,005287269 | 0,017817698 |
| b1812 | 1362 | 0,51 | 0,659227926 | 5,311521023 | 7,770485849 | 0,005310676 | 0,017883134 |
| b0231 | 1056 | 0,54 | -0,740023367 | 5,770721523 | 7,768916748 | 0,005315292 | 0,017885238 |
| b3987 | 4029 | 0,53 | 0,87197106 | 11,96639742 | 7,763548309 | 0,005331113 | 0,017909482 |
| b0889 | 495 | 0,48 | 0,605543959 | 7,738794094 | 7,763218722 | 0,005332085 | 0,017909482 |
| b1817 | 972 | 0,5 | -0,795741021 | 10,50581922 | 7,762406304 | 0,005334484 | 0,017909482 |
| b0692 | 1320 | 0,51 | -0,804550188 | 4,567247415 | 7,75311526 | 0,005361997 | 0,017988376 |
| b2295 | 456 | 0,51 | -0,675862238 | 6,304671753 | 7,732768505 | 0,005422754 | 0,018178596 |
| b4493 | 1001 | 0,48 | 0,806238449 | 8,382354563 | 7,731224425 | 0,005427393 | 0,01818055 |
| b2395 | 2190 | 0,49 | 0,596133119 | 5,553032966 | 7,726465628 | 0,005441717 | 0,018214918 |
| b0681 | 1407 | 0,51 | -0,92098667 | 3,728348996 | 7,717636391 | 0,005468394 | 0,018290555 |
| b1878 | 393 | 0,53 | 2,149462124 | 1,22436253 | 7,713722125 | 0,005480264 | 0,018315361 |
| b2960 | 720 | 0,53 | 0,553178381 | 7,104533558 | 7,712497279 | 0,005483983 | 0,018315361 |
| b3041 | 654 | 0,52 | -0,778800124 | 8,519529803 | 7,709164594 | 0,005494117 | 0,018335542 |
| b0440 | 273 | 0,49 | 0,702007128 | 10,00654427 | 7,707491366 | 0,005499212 | 0,018338891 |
| b0659 | 468 | 0,52 | -0,639399036 | 7,312665653 | 7,700740571 | 0,005519817 | 0,018385475 |
| b0249 | 240 | 0,59 | 1,548890728 | 2,201784972 | 7,700227928 | 0,005521385 | 0,018385475 |
| b0127 | 927 | 0,5 | 0,622744881 | 5,75762411 | 7,696447965 | 0,00553296 | 0,01841034 |
| b1607 | 336 | 0,47 | 1,14987488 | 3,31093393 | 7,692322539 | 0,005545622 | 0,018438781 |
| b1565 | 306 | 0,46 | 1,728269821 | 2,047201678 | 7,674246848 | 0,005601447 | 0,018610589 |
| b4288 | 957 | 0,6 | 0,930532084 | 3,315613901 | 7,664551051 | 0,005631627 | 0,018697002 |
| b4206 | 639 | 0,56 | -0,60796918 | 7,214508679 | 7,654163053 | 0,005664146 | 0,018791046 |
| b4634 | 309 | 0,53 | -1,699956181 | 2,621449979 | 7,646338612 | 0,005688767 | 0,018858766 |
| b2798 | 756 | 0,53 | -0,592082672 | 5,085393588 | 7,641883885 | 0,005702833 | 0,018891423 |
| b0292 | 669 | 0,55 | 1,946594065 | 1,500286766 | 7,636537671 | 0,00571976 | 0,018933505 |
| b0089 | 1245 | 0,53 | -0,582895347 | 7,749058358 | 7,633890664 | 0,00572816 | 0,018947318 |
| b0794 | 1737 | 0,56 | -0,501460658 | 7,099804214 | 7,62935371 | 0,005742588 | 0,018981031 |
| b0148 | 2430 | 0,57 | -0,516640671 | 6,709205866 | 7,611242061 | 0,005800552 | 0,019158491 |
| b2459 | 804 | 0,59 | -0,841721698 | 3,620452422 | 7,591742341 | 0,005863625 | 0,019352554 |
| b0658 | 879 | 0,52 | -0,669004888 | 8,200647956 | 7,586946387 | 0,005879245 | 0,019389828 |
| b0795 | 999 | 0,57 | -0,568503046 | 7,091627324 | 7,569932886 | 0,005934999 | 0,019559314 |
| b2326 | 549 | 0,5 | 0,822671373 | 4,81699714 | 7,560594998 | 0,005965829 | 0,019636905 |
| b1789 | 447 | 0,55 | 1,266606895 | 2,685994231 | 7,559659288 | 0,005968928 | 0,019636905 |
| b4040 | 873 | 0,52 | 0,653494249 | 6,382712926 | 7,558473077 | 0,005972858 | 0,019636905 |
| b3058 | 369 | 0,51 | 1,50727344 | 2,789759644 | 7,5575046 | 0,005976068 | 0,019636905 |
| b3533 | 2619 | 0,55 | 0,919116643 | 3,817092617 | 7,523808245 | 0,006088882 | 0,019992945 |
| b3114 | 2295 | 0,53 | -1,760444471 | 5,856421438 | 7,507047298 | 0,006145804 | 0,020165076 |
| b3355 | 870 | 0,53 | -0,594695046 | 6,35986078 | 7,492067873 | 0,006197135 | 0,020318625 |
| b4162 | 546 | 0,54 | -0,497657297 | 6,041213061 | 7,468410376 | 0,006279097 | 0,020572304 |
| b1439 | 1407 | 0,51 | 0,689558706 | 5,265200674 | 7,466700494 | 0,006285064 | 0,020576811 |
| b3427 | 219 | 0,44 | 1,733712715 | 1,848390201 | 7,464812112 | 0,00629166 | 0,020583372 |
| b0833 | 2349 | 0,42 | 1,043490011 | 3,427704638 | 7,460984325 | 0,006305052 | 0,020597631 |
| b3629 | 936 | 0,27 | -0,895208428 | 4,901063997 | 7,460939376 | 0,00630521 | 0,020597631 |
| b0209 | 801 | 0,53 | -0,56722769 | 7,168643648 | 7,445460055 | 0,006359666 | 0,020758333 |
| b0966 | 318 | 0,56 | -0,985661708 | 8,156208912 | 7,444328554 | 0,006363666 | 0,020758333 |
| b0635 | 1902 | 0,53 | 0,765707579 | 5,418346575 | 7,432360156 | 0,006406127 | 0,020881644 |
| b3247 | 1470 | 0,52 | 0,484176161 | 7,372932899 | 7,428698797 | 0,006419174 | 0,02090684 |
| b3367 | 807 | 0,55 | -1,560505623 | 2,783262821 | 7,427574585 | 0,006423186 | 0,02090684 |
| b2169 | 1131 | 0,56 | 0,544383011 | 6,671876777 | 7,418266228 | 0,0064565 | 0,021000024 |
| b0654 | 741 | 0,52 | 1,003849521 | 5,496853615 | 7,406312492 | 0,006499541 | 0,021124686 |
| b0492 | 855 | 0,56 | 0,568113479 | 7,43958207 | 7,39812695 | 0,006529183 | 0,02120565 |
| b3283 | 543 | 0,5 | -0,598317692 | 7,234446088 | 7,384594773 | 0,006578489 | 0,021350317 |
| b4440 | 304 | 0,53 | 0,804651973 | 4,356017406 | 7,366973983 | 0,006643263 | 0,021522237 |
| b1598 | 822 | 0,52 | -0,943839075 | 8,013374577 | 7,366867808 | 0,006643655 | 0,021522237 |
| b2736 | 909 | 0,53 | -0,947249046 | 3,885423672 | 7,366269359 | 0,006645867 | 0,021522237 |
| b4050 | 243 | 0,5 | 1,445990455 | 2,437446203 | 7,362027651 | 0,006661563 | 0,02155749 |
| b4486 | 2937 | 0,5 | -0,564128673 | 6,359956315 | 7,354537828 | 0,00668937 | 0,02163186 |
| b2923 | 636 | 0,51 | -1,021350698 | 3,629215272 | 7,350401629 | 0,006704778 | 0,021666051 |
| b1066 | 585 | 0,49 | -0,505521967 | 6,639244159 | 7,347325357 | 0,00671626 | 0,02168752 |
| b1233 | 459 | 0,46 | 0,77591027 | 4,712704509 | 7,344195279 | 0,006727964 | 0,021709673 |
| b0208 | 915 | 0,5 | -0,765551593 | 5,301409752 | 7,339677435 | 0,006744894 | 0,021748644 |
| b3434 | 594 | 0,5 | 0,900542774 | 3,793094629 | 7,330715455 | 0,006778607 | 0,021841635 |
| b4372 | 414 | 0,58 | 1,059244693 | 4,540394213 | 7,318915371 | 0,006823258 | 0,021969714 |
| b1944 | 465 | 0,53 | 1,668822247 | 1,577891465 | 7,294187527 | 0,006917805 | 0,022258148 |
| b0786 | 705 | 0,49 | -0,794114841 | 8,644012291 | 7,292473348 | 0,006924408 | 0,022263413 |
| b4297 | 1968 | 0,55 | -0,68695659 | 5,869694571 | 7,276707829 | 0,006985445 | 0,022416725 |
| b3604 | 777 | 0,54 | 1,892562529 | 5,392404001 | 7,275653144 | 0,006989547 | 0,022416725 |
| b2792 | 330 | 0,52 | 1,287199255 | 3,050184064 | 7,275209379 | 0,006991274 | 0,022416725 |
| b3632 | 1035 | 0,45 | 0,588791942 | 6,087045691 | 7,274997877 | 0,006992098 | 0,022416725 |
| b1601 | 1035 | 0,5 | -0,856856111 | 4,764405411 | 7,266867561 | 0,00702382 | 0,02250233 |
| b0803 | 267 | 0,52 | -1,154390516 | 4,80740523 | 7,248245023 | 0,007097034 | 0,022720648 |

| | | | | | | | |
|---|---|---|---|---|---|---|---|
| b0243 | 1254 | 0,54 | 0,5673848 | 7,979696177 | 7,240630048 | 0,007127197 | 0,022800924 |
| b2497 | 1290 | 0,52 | 1,262505191 | 4,329373537 | 7,234683394 | 0,007150842 | 0,022860252 |
| b3050 | 630 | 0,43 | 1,207427238 | 2,154316872 | 7,22787243 | 0,007178023 | 0,022930789 |
| b4503 | 273 | 0,47 | 1,377319052 | 1,769420866 | 7,222451021 | 0,007199733 | 0,022983764 |
| b0305 | 855 | 0,51 | -0,863818714 | 4,149213429 | 7,218545165 | 0,007215416 | 0,023017435 |
| b2182 | 1191 | 0,53 | 0,623311752 | 5,808775786 | 7,204382109 | 0,007272578 | 0,023183283 |
| b1221 | 651 | 0,52 | -0,843800019 | 8,215825258 | 7,196931811 | 0,007302833 | 0,023252376 |
| b2349 | 1158 | 0,48 | 0,751824556 | 4,109237553 | 7,196490698 | 0,007304629 | 0,023252376 |
| b3388 | 1287 | 0,55 | 0,555162684 | 8,614474756 | 7,185876057 | 0,007347967 | 0,023373731 |
| b3836 | 270 | 0,46 | -0,602450736 | 7,427388323 | 7,174877061 | 0,007393151 | 0,023484834 |
| b1035 | 555 | 0,54 | 0,675726117 | 6,36733279 | 7,174823123 | 0,007393374 | 0,023484834 |
| b2140 | 948 | 0,54 | 0,880445 | 3,573864724 | 7,160751551 | 0,007451597 | 0,023653015 |
| b0414 | 1104 | 0,55 | -0,562955253 | 7,544420094 | 7,152352085 | 0,007486574 | 0,023747222 |
| b0178 | 486 | 0,5 | 0,601277867 | 9,003844555 | 7,142974123 | 0,007525824 | 0,02385484 |
| b2468 | 1980 | 0,56 | -0,766685727 | 4,826167661 | 7,133905041 | 0,007563982 | 0,023958845 |
| b4230 | 1026 | 0,58 | -1,149606097 | 5,469311696 | 7,131199287 | 0,007575404 | 0,02397808 |
| b2986 | 693 | 0,47 | 2,581308212 | 0,872108468 | 7,128837702 | 0,007585388 | 0,023992738 |
| b4152 | 396 | 0,49 | -0,974962633 | 7,890586783 | 7,123548821 | 0,007607797 | 0,024046647 |
| b3930 | 927 | 0,53 | 1,024073328 | 4,753652421 | 7,118914588 | 0,007627487 | 0,024091894 |
| b4301 | 633 | 0,55 | -0,9477213 | 4,163055643 | 7,115663785 | 0,007641331 | 0,024118622 |
| b1701 | 1647 | 0,5 | 1,075241789 | 3,150743986 | 7,100657478 | 0,007705568 | 0,024304262 |
| b2129 | 927 | 0,55 | 0,628243581 | 5,766062518 | 7,097319233 | 0,007719933 | 0,024332447 |
| b1362 | 444 | 0,52 | 1,682098251 | 1,812035546 | 7,08833621 | 0,007758724 | 0,024437529 |
| b2367 | 1539 | 0,42 | 1,806429672 | 1,38208051 | 7,069483797 | 0,007840784 | 0,024665644 |
| b1248 | 330 | 0,47 | 0,668020815 | 5,489378419 | 7,06917039 | 0,007842156 | 0,024665644 |
| b2303 | 363 | 0,45 | 0,581327492 | 6,46157306 | 7,064750197 | 0,007861527 | 0,024709233 |
| b1827 | 792 | 0,5 | -0,529551641 | 7,704407388 | 7,055996816 | 0,007900034 | 0,02481286 |
| b0557 | 294 | 0,41 | 0,945711959 | 5,280431938 | 7,04341097 | 0,007955737 | 0,024970319 |
| b0599 | 1089 | 0,55 | -0,747476607 | 6,682260198 | 7,03183906 | 0,008007308 | 0,025114594 |
| b0937 | 576 | 0,54 | 1,501873601 | 2,261093362 | 7,024665682 | 0,008039448 | 0,025197766 |
| b0821 | 1266 | 0,56 | 1,219860478 | 5,659050019 | 7,005652403 | 0,008125276 | 0,025448977 |
| b0974 | 708 | 0,52 | -1,144054018 | 4,527510371 | 6,9954486 | 0,008171723 | 0,025576579 |
| b1344 | 936 | 0,49 | 0,574550709 | 5,69149116 | 6,978379377 | 0,008250028 | 0,025803648 |
| b2616 | 1662 | 0,52 | -0,649140466 | 6,866949194 | 6,973332745 | 0,008273327 | 0,025858473 |
| b3023 | 483 | 0,53 | -0,684678289 | 6,010497377 | 6,971317222 | 0,00828265 | 0,025869575 |
| b3679 | 1716 | 0,53 | 0,870312391 | 2,837019089 | 6,967235909 | 0,008301563 | 0,025893291 |
| b0066 | 699 | 0,57 | 0,956415257 | 3,135675659 | 6,96718531 | 0,008301798 | 0,025893291 |
| b3002 | 495 | 0,47 | 0,765514632 | 5,715489752 | 6,946671038 | 0,008397536 | 0,026173682 |
| b2208 | 495 | 0,55 | -1,614025313 | 7,870333985 | 6,943171192 | 0,008413981 | 0,026206716 |
| b2560 | 636 | 0,52 | -0,544364728 | 6,029216247 | 6,936690617 | 0,008444521 | 0,026283571 |
| b2033 | 591 | 0,41 | 0,851577589 | 6,479287386 | 6,92836822 | 0,008483906 | 0,026387832 |
| b2687 | 516 | 0,52 | 0,594415448 | 9,021920652 | 6,926003767 | 0,00849513 | 0,026404419 |
| b2514 | 1275 | 0,55 | 0,543325317 | 8,442003239 | 6,920052004 | 0,00852345 | 0,026474083 |
| b0092 | 921 | 0,52 | -0,601112796 | 8,123280176 | 6,917660455 | 0,008534856 | 0,026491154 |
| b2432 | 576 | 0,51 | 0,72432343 | 5,672621742 | 6,892250507 | 0,008657021 | 0,026851743 |
| b0094 | 1263 | 0,52 | -0,56134588 | 8,997959652 | 6,887488738 | 0,008680113 | 0,026904748 |
| b0642 | 2583 | 0,53 | 0,605690876 | 9,929683208 | 6,883494582 | 0,008699531 | 0,0269463 |
| b3052 | 1434 | 0,56 | 0,513175903 | 7,842463723 | 6,876013 | 0,008736023 | 0,027040645 |
| b1238 | 618 | 0,42 | 1,173080278 | 4,328326585 | 6,874767998 | 0,008742111 | 0,027040814 |
| b1941 | 1374 | 0,59 | 1,899494931 | 1,642457166 | 6,869528619 | 0,008767778 | 0,027101503 |
| b3393 | 441 | 0,55 | 2,025012541 | 1,274054697 | 6,86817569 | 0,008774418 | 0,027103336 |
| b1764 | 1044 | 0,55 | -0,572011025 | 8,735668858 | 6,863487642 | 0,008797467 | 0,027155818 |
| b3371 | 1023 | 0,54 | -0,978849661 | 3,243252171 | 6,861553795 | 0,008806993 | 0,027166513 |
| b0975 | 588 | 0,59 | -1,125196931 | 4,463214227 | 6,859098532 | 0,008819103 | 0,027185158 |
| b4484 | 501 | 0,5 | 0,576426473 | 7,451931097 | 6,851553073 | 0,008856425 | 0,027281442 |
| b3515 | 729 | 0,42 | -0,585493624 | 7,996866896 | 6,846547628 | 0,008881273 | 0,027339193 |
| b1709 | 750 | 0,55 | 0,647265269 | 6,268286812 | 6,829154635 | 0,00896817 | 0,027583809 |
| b3180 | 294 | 0,49 | 0,641479545 | 6,72900523 | 6,828183781 | 0,008973046 | 0,027583809 |
| b1803 | 966 | 0,5 | 1,04165789 | 2,992188569 | 6,812025529 | 0,0090546 | 0,027815433 |
| b1114 | 3447 | 0,55 | 0,610627642 | 7,901943713 | 6,80854784 | 0,009072251 | 0,027850569 |
| b0197 | 816 | 0,49 | 0,493804332 | 7,325252635 | 6,795425217 | 0,009139175 | 0,028036813 |
| b1513 | 1536 | 0,53 | -0,93356381 | 7,241550378 | 6,778051512 | 0,009228558 | 0,028291653 |
| b1808 | 1911 | 0,55 | 0,669837097 | 5,53260292 | 6,772819302 | 0,009255651 | 0,028355316 |
| b3868 | 1410 | 0,57 | -0,631671802 | 5,551243454 | 6,76338893 | 0,009304689 | 0,028486076 |
| b1770 | 759 | 0,42 | -0,719107309 | 5,670606116 | 6,748365455 | 0,009383361 | 0,02870732 |
| b4456 | 207 | 0,47 | -0,546189873 | 8,518784542 | 6,736918771 | 0,00944376 | 0,028872395 |
| b0402 | 1374 | 0,53 | 0,704722005 | 5,156640864 | 6,730144669 | 0,009479691 | 0,028962492 |
| b3828 | 954 | 0,56 | 1,340761364 | 4,143700305 | 6,722142501 | 0,009522317 | 0,029072905 |
| b2595 | 738 | 0,51 | 0,6520669 | 7,731288674 | 6,709591502 | 0,009589569 | 0,029258304 |
| b2284 | 1338 | 0,56 | 0,857414666 | 10,21966362 | 6,70208709 | 0,009630012 | 0,029361711 |
| b4239 | 1656 | 0,53 | -0,912267233 | 7,735817406 | 6,699564424 | 0,009643647 | 0,029366255 |
| b0597 | 414 | 0,57 | 1,57007888 | 2,565134194 | 6,699386938 | 0,009644607 | 0,029366255 |
| b2785 | 1302 | 0,51 | -0,475838886 | 7,101104229 | 6,696069573 | 0,009662568 | 0,029400791 |
| b2756 | 600 | 0,45 | 0,931223342 | 3,199564539 | 6,694804869 | 0,009669425 | 0,029400791 |
| b2573 | 576 | 0,49 | -0,605832327 | 8,303136905 | 6,69366147 | 0,009675628 | 0,029400791 |
| b2433 | 450 | 0,53 | 0,651669616 | 4,56840037 | 6,671827938 | 0,009794868 | 0,029741729 |
| b1382 | 186 | 0,43 | 1,134714392 | 2,861581339 | 6,670694138 | 0,009801101 | 0,029741729 |
| b4167 | 1548 | 0,57 | -0,594483852 | 8,12513768 | 6,667790982 | 0,009817079 | 0,029770061 |

| | | | | | | | |
|---|---|---|---|---|---|---|---|
| b1915 | 225 | 0,48 | 1,16755695 | 3,733124144 | 6,658410031 | 0,009868893 | 0,02990695 |
| b2130 | 1158 | 0,57 | 0,714805145 | 5,793270697 | 6,636627009 | 0,009990291 | 0,030254381 |
| b0019 | 1167 | 0,51 | -0,618490316 | 8,473991089 | 6,624366395 | 0,010059292 | 0,030442773 |
| b1175 | 813 | 0,5 | 0,61970971 | 8,607226416 | 6,599133072 | 0,010202843 | 0,030856372 |
| b4377 | 1074 | 0,54 | 0,558500395 | 7,080163002 | 6,595624931 | 0,010222967 | 0,030896383 |
| b3051 | 1662 | 0,5 | 1,021030125 | 3,068199616 | 6,593567558 | 0,010234787 | 0,030911264 |
| b0048 | 480 | 0,53 | -0,668317968 | 6,146007104 | 6,59124371 | 0,010248155 | 0,030924799 |
| b0070 | 1179 | 0,49 | -0,582067893 | 5,249773389 | 6,590390479 | 0,010253068 | 0,030924799 |
| b1040 | 651 | 0,42 | 1,351267945 | 3,671624743 | 6,581495107 | 0,010304431 | 0,031058815 |
| b2898 | 981 | 0,54 | -0,476667671 | 8,690800285 | 6,548576236 | 0,010496812 | 0,031617414 |
| b1463 | 846 | 0,51 | -0,696019593 | 4,455210657 | 6,527396258 | 0,010622534 | 0,031974612 |
| b4036 | 1341 | 0,52 | -1,3751795 | 10,94087993 | 6,524191121 | 0,010641693 | 0,032010785 |
| b0700 | 4194 | 0,59 | -0,791300146 | 4,504846207 | 6,513345573 | 0,010706788 | 0,032184994 |
| b0477 | 1305 | 0,52 | 0,682559216 | 5,752115943 | 6,510286907 | 0,01072522 | 0,032218792 |
| b1203 | 1092 | 0,51 | 0,496272048 | 7,642577795 | 6,502549754 | 0,01077199 | 0,032337616 |
| b3774 | 1476 | 0,54 | -0,774262775 | 5,534015617 | 6,494031036 | 0,010823727 | 0,032471182 |
| b0245 | 342 | 0,51 | 1,373960949 | 1,973344124 | 6,489752579 | 0,010849808 | 0,032527651 |
| b3006 | 735 | 0,53 | 1,140377179 | 5,699741042 | 6,481427505 | 0,01090074 | 0,032658501 |
| b1167 | 249 | 0,36 | 1,783034605 | 2,42468535 | 6,475694805 | 0,010935955 | 0,032742119 |
| b2940 | 216 | 0,48 | 1,148236964 | 5,378836195 | 6,471081721 | 0,010964378 | 0,032805301 |
| b3456 | 1278 | 0,57 | 1,196359813 | 2,844885001 | 6,449543703 | 0,011098084 | 0,033183198 |
| b2644 | 483 | 0,54 | 2,203804788 | 1,318516229 | 6,444959652 | 0,011126757 | 0,03324675 |
| b3607 | 822 | 0,54 | 0,548949191 | 7,064064071 | 6,438022971 | 0,01117029 | 0,033341607 |
| b3140 | 792 | 0,49 | 1,391850494 | 1,908718957 | 6,437531453 | 0,011173381 | 0,033341607 |
| b3558 | 852 | 0,43 | 1,71351213 | 1,826447386 | 6,427167591 | 0,011238766 | 0,033514405 |
| b3482 | 4236 | 0,59 | -0,946039155 | 3,021779011 | 6,425021 | 0,011252358 | 0,033532626 |
| b2583 | 699 | 0,52 | 0,922650509 | 3,198765305 | 6,422451664 | 0,011268649 | 0,03355886 |
| b1751 | 678 | 0,48 | -1,019975051 | 4,815148264 | 6,40140469 | 0,011403011 | 0,03393645 |
| b4088 | 936 | 0,49 | -0,755448386 | 4,187564587 | 6,397740843 | 0,011426568 | 0,033983993 |
| b0936 | 960 | 0,52 | 1,404066701 | 1,475145249 | 6,381850293 | 0,011529318 | 0,034266844 |
| b0532 | 2604 | 0,48 | 1,444955677 | 2,281426243 | 6,367509994 | 0,011622857 | 0,034521966 |
| b2840 | 693 | 0,51 | 0,687496463 | 4,904264336 | 6,359162502 | 0,011677665 | 0,034661785 |
| b3083 | 315 | 0,38 | 1,574124182 | 2,115709292 | 6,352327989 | 0,011722737 | 0,034772539 |
| b0379 | 309 | 0,52 | 1,137178238 | 2,717130648 | 6,350158333 | 0,011737082 | 0,034792065 |
| b0830 | 1539 | 0,52 | -0,500557394 | 6,96525996 | 6,344517672 | 0,011774462 | 0,034879801 |
| b0572 | 1374 | 0,51 | 1,608250857 | 2,080994749 | 6,338544484 | 0,011814179 | 0,034974339 |
| b3199 | 576 | 0,46 | 0,542500483 | 6,572124576 | 6,335507116 | 0,011834427 | 0,035011158 |
| b0382 | 261 | 0,46 | -0,493832858 | 7,120889489 | 6,328134553 | 0,011883725 | 0,03513381 |
| b4567 | 237 | 0,48 | 1,999550381 | 1,096203434 | 6,326814387 | 0,011892575 | 0,035136797 |
| b2229 | 624 | 0,51 | 2,059966771 | 1,084506272 | 6,317670959 | 0,011954054 | 0,035295171 |
| b2624 | 213 | 0,47 | 2,438210243 | 0,955965534 | 6,307792764 | 0,01202084 | 0,035468997 |
| b4395 | 648 | 0,56 | 0,482518265 | 5,715338932 | 6,306441663 | 0,012030005 | 0,035472685 |
| b1888 | 1965 | 0,53 | 1,016496171 | 2,844406625 | 6,303194961 | 0,012052056 | 0,035514344 |
| b1253 | 399 | 0,48 | 1,423950775 | 3,622628996 | 6,295897253 | 0,012101774 | 0,03563742 |
| b4278 | 1329 | 0,54 | 0,610593008 | 4,466642818 | 6,292097945 | 0,012127742 | 0,035690439 |
| b1623 | 1002 | 0,54 | -0,562882137 | 6,351564941 | 6,288258377 | 0,012154042 | 0,035732714 |
| b3081 | 2019 | 0,57 | 0,794452126 | 6,682154619 | 6,287673759 | 0,012158052 | 0,035732714 |
| b0222 | 579 | 0,51 | 0,493685687 | 7,754026926 | 6,285616018 | 0,012172176 | 0,035750782 |
| b1868 | 819 | 0,51 | 0,662183675 | 4,870941767 | 6,279316993 | 0,012215517 | 0,035854583 |
| b4269 | 1020 | 0,56 | -0,692678718 | 8,088073526 | 6,274314405 | 0,012250051 | 0,035932413 |
| b0102 | 744 | 0,51 | 0,573228001 | 5,896725313 | 6,258247159 | 0,012361645 | 0,036200138 |
| b3567 | 1542 | 0,47 | -0,654830442 | 5,248759197 | 6,257998702 | 0,012363379 | 0,036200138 |
| b3674 | 498 | 0,54 | -0,646651326 | 4,835671786 | 6,257687132 | 0,012365554 | 0,036200138 |
| b2308 | 687 | 0,53 | 0,888301632 | 5,067326465 | 6,254952331 | 0,012384658 | 0,036214599 |
| b4158 | 315 | 0,48 | 1,235589288 | 2,338502467 | 6,254666969 | 0,012386653 | 0,036214599 |
| b2392 | 1239 | 0,53 | 0,768528729 | 5,802413553 | 6,250861566 | 0,012413291 | 0,036268822 |
| b0221 | 2445 | 0,57 | 0,813681058 | 8,246923303 | 6,246142039 | 0,01244641 | 0,036341897 |
| b0306 | 720 | 0,51 | -1,651796773 | 6,201246144 | 6,237112635 | 0,012510027 | 0,036474132 |
| b2221 | 663 | 0,54 | 1,665527598 | 1,284865975 | 6,236893792 | 0,012511573 | 0,036474132 |
| b2939 | 132 | 0,41 | 1,459397273 | 2,58977356 | 6,236251344 | 0,012516112 | 0,036474132 |
| b0856 | 954 | 0,51 | 1,229942856 | 3,329217944 | 6,235014392 | 0,012524858 | 0,036475901 |
| b2751 | 1428 | 0,55 | -0,637190381 | 5,069165494 | 6,233732776 | 0,012533925 | 0,036478606 |
| b3932 | 531 | 0,53 | 0,758317606 | 6,831509428 | 6,231196467 | 0,01255189 | 0,036507184 |
| b0517 | 1050 | 0,52 | 1,267137421 | 1,794045988 | 6,219888666 | 0,012632306 | 0,036717246 |
| b3326 | 1482 | 0,52 | 1,108276452 | 2,62782854 | 6,20879429 | 0,012711718 | 0,036924122 |
| b3581 | 663 | 0,59 | -1,246494839 | 2,344374608 | 6,205907612 | 0,012732465 | 0,036960432 |
| b3613 | 1260 | 0,57 | 0,692544931 | 5,68899626 | 6,193462746 | 0,012822305 | 0,037197134 |
| b0965 | 414 | 0,54 | -0,643498831 | 7,622509113 | 6,189298249 | 0,012852514 | 0,037260653 |
| b3590 | 1845 | 0,56 | -0,540446485 | 7,563813595 | 6,179892782 | 0,012921011 | 0,037435016 |
| b3139 | 804 | 0,51 | 2,155347292 | 1,144446555 | 6,172598117 | 0,012974393 | 0,037565394 |
| b4118 | 909 | 0,5 | -0,858281421 | 6,653257491 | 6,169403186 | 0,012997845 | 0,037609 |
| b3167 | 402 | 0,53 | 0,464294367 | 6,63470114 | 6,156198983 | 0,013095232 | 0,037866341 |
| b3404 | 1353 | 0,57 | -0,424701534 | 7,238339611 | 6,151395919 | 0,013130842 | 0,037944832 |
| b2906 | 1203 | 0,55 | -0,493836941 | 6,84529453 | 6,1369831 | 0,013238299 | 0,038230706 |
| b2037 | 1248 | 0,35 | 0,578604115 | 6,564048511 | 6,126926348 | 0,013313814 | 0,038417271 |
| b0956 | 453 | 0,46 | -0,560536925 | 5,956227122 | 6,126099159 | 0,013320045 | 0,038417271 |
| b0524 | 723 | 0,54 | 0,789834167 | 4,600446488 | 6,120507781 | 0,013362241 | 0,03851419 |
| b0199 | 1032 | 0,53 | 1,286868395 | 5,826166132 | 6,115635312 | 0,013399124 | 0,038595679 |

| | | | | | | | |
|---|---|---|---|---|---|---|---|
| b1781 | 855 | 0,51 | -0,593388429 | 5,159067022 | 6,112541404 | 0,013422599 | 0,038638464 |
| b3166 | 945 | 0,52 | 0,519906309 | 7,203381778 | 6,103881909 | 0,013488526 | 0,038803321 |
| b3135 | 555 | 0,58 | -0,7446406 | 3,537964462 | 6,086726636 | 0,013620119 | 0,039156751 |
| b0922 | 1323 | 0,53 | 0,476718881 | 6,863597348 | 6,079114092 | 0,013678935 | 0,039300633 |
| b4494 | 1900 | 0,39 | 1,477192051 | 1,62154013 | 6,075745248 | 0,013705047 | 0,039333973 |
| b2294 | 495 | 0,48 | -0,489750855 | 7,618488091 | 6,075352852 | 0,013708092 | 0,039333973 |
| b1685 | 189 | 0,44 | 1,239297456 | 3,281655793 | 6,061984819 | 0,013812236 | 0,039607449 |
| b1468 | 3741 | 0,55 | -0,830678067 | 9,256980982 | 6,055415298 | 0,013863715 | 0,039729648 |
| b2790 | 450 | 0,53 | -0,568952426 | 6,404968698 | 6,050522914 | 0,013902179 | 0,03981442 |
| b0117 | 1854 | 0,54 | -0,682301461 | 4,362110929 | 6,038357944 | 0,013998298 | 0,040064096 |
| b2828 | 876 | 0,53 | 0,568714146 | 5,925782083 | 6,035694207 | 0,014019437 | 0,040088983 |
| b0685 | 294 | 0,54 | 1,222891962 | 2,479702509 | 6,035008541 | 0,014024883 | 0,040088983 |
| b2080 | 333 | 0,48 | -0,844491144 | 8,044016285 | 6,019096781 | 0,014151888 | 0,040426233 |
| b1443 | 795 | 0,54 | -0,657213659 | 7,240807462 | 6,008646161 | 0,014235946 | 0,040625287 |
| b2603 | 519 | 0,46 | -0,715749994 | 4,478679993 | 6,008181183 | 0,014239698 | 0,040625287 |
| b2381 | 735 | 0,47 | -0,539067041 | 5,856759289 | 5,992904771 | 0,014363531 | 0,040952509 |
| b1870 | 744 | 0,51 | 0,69117983 | 5,656440251 | 5,987246635 | 0,014409677 | 0,041037356 |
| b3210 | 2337 | 0,51 | -0,498197634 | 8,193723252 | 5,987011086 | 0,014411602 | 0,041037356 |
| b4148 | 318 | 0,53 | -0,730603366 | 5,277586554 | 5,979548153 | 0,01447271 | 0,041185196 |
| b2685 | 1173 | 0,53 | 0,730015264 | 4,788354375 | 5,978231071 | 0,014483522 | 0,041189813 |
| b0655 | 909 | 0,49 | 0,647274338 | 8,935418507 | 5,973312332 | 0,014523974 | 0,041278664 |
| b3996 | 774 | 0,53 | 0,663546243 | 6,124690449 | 5,969441348 | 0,014555892 | 0,04134316 |
| b3363 | 573 | 0,51 | 0,69088081 | 6,617309136 | 5,961860046 | 0,014618611 | 0,041495005 |
| b3574 | 849 | 0,52 | 0,663014761 | 4,995226473 | 5,95691997 | 0,014659629 | 0,0415851 |
| b2605 | 483 | 0,51 | 0,700785271 | 4,210860121 | 5,942293914 | 0,014781768 | 0,04190505 |
| b3813 | 2163 | 0,58 | 0,530344617 | 7,13345073 | 5,928376618 | 0,014898961 | 0,042210583 |
| b0604 | 747 | 0,47 | 0,561934097 | 5,593056589 | 5,926959425 | 0,014910948 | 0,042217858 |
| b3934 | 1026 | 0,52 | 0,614106903 | 6,368039228 | 5,901881026 | 0,015124724 | 0,042796093 |
| b0732 | 2634 | 0,49 | -0,536102062 | 5,039572369 | 5,898616502 | 0,015152783 | 0,042848437 |
| b1752 | 708 | 0,52 | -0,85831528 | 4,511725669 | 5,895077721 | 0,015183259 | 0,042907547 |
| b0678 | 801 | 0,49 | -0,44615786 | 7,709815506 | 5,889452553 | 0,015231835 | 0,043017696 |
| b2737 | 1167 | 0,55 | -0,790154153 | 4,06897767 | 5,881833026 | 0,015297887 | 0,043177035 |
| b1054 | 921 | 0,53 | -0,62675455 | 6,024619767 | 5,875577634 | 0,015352335 | 0,04330344 |
| b0434 | 579 | 0,51 | 0,91857369 | 5,500640551 | 5,87105575 | 0,015391818 | 0,043387503 |
| b0798 | 483 | 0,5 | -0,883447443 | 3,91384465 | 5,869043995 | 0,015409418 | 0,043409812 |
| b3440 | 1038 | 0,51 | -0,529674493 | 7,698921327 | 5,861763686 | 0,015473282 | 0,043562342 |
| b2759 | 483 | 0,46 | 1,299997558 | 2,198682468 | 5,858311889 | 0,015503657 | 0,043620458 |
| b4002 | 426 | 0,54 | -1,191989973 | 6,755035152 | 5,857044008 | 0,015514829 | 0,043624507 |
| b4483 | 783 | 0,54 | 0,738557059 | 3,980120608 | 5,853917817 | 0,015542412 | 0,043674665 |
| b1619 | 768 | 0,47 | -0,623039615 | 9,189995903 | 5,851293319 | 0,015565608 | 0,043712439 |
| b2047 | 1395 | 0,55 | 1,228596699 | 1,983862279 | 5,845225815 | 0,015619369 | 0,043835951 |
| b1341 | 1233 | 0,48 | 0,51021159 | 6,368483076 | 5,837856556 | 0,015684923 | 0,043992381 |
| b1908 | 666 | 0,53 | -0,536589992 | 7,942702944 | 5,835526336 | 0,01570571 | 0,044023136 |
| b4371 | 1032 | 0,56 | 0,570731538 | 5,462570592 | 5,832334998 | 0,015734226 | 0,0440755 |
| b2475 | 864 | 0,54 | -0,673972608 | 7,775048819 | 5,829718634 | 0,015757644 | 0,044113528 |
| b2813 | 1098 | 0,53 | 0,510350598 | 5,881738224 | 5,815923836 | 0,015881709 | 0,044433096 |
| b0531 | 693 | 0,43 | 1,910596351 | 0,839333586 | 5,813165029 | 0,015906641 | 0,0444514 |
| b1148 | 339 | 0,5 | 1,584984142 | 1,657365469 | 5,812615636 | 0,015911611 | 0,0444514 |
| b4211 | 861 | 0,54 | -0,844968696 | 4,441059625 | 5,811909103 | 0,015918005 | 0,0444514 |
| b0612 | 1464 | 0,51 | 1,163450781 | 2,557890852 | 5,809696703 | 0,015938043 | 0,044458346 |
| b3732 | 1383 | 0,55 | 0,962611356 | 11,59678094 | 5,809444276 | 0,015940331 | 0,044458346 |
| b2352 | 1332 | 0,28 | 1,093838372 | 5,041234105 | 5,800716842 | 0,016019642 | 0,044651764 |
| b3141 | 756 | 0,5 | 1,781248238 | 1,626386454 | 5,79171979 | 0,01610183 | 0,044852952 |
| b2487 | 1668 | 0,54 | -0,683017808 | 4,263007946 | 5,785198506 | 0,016161673 | 0,044991688 |
| b3088 | 966 | 0,54 | 1,171253891 | 3,061283991 | 5,783514411 | 0,016177164 | 0,045006859 |
| b0645 | 708 | 0,46 | 2,154680713 | 0,908266736 | 5,781333636 | 0,016197247 | 0,045034778 |
| b1731 | 243 | 0,52 | 1,45656378 | 3,308872766 | 5,770757994 | 0,016295005 | 0,045278494 |
| b2057 | 1218 | 0,54 | 1,362321036 | 1,985961821 | 5,761934239 | 0,016377034 | 0,045478231 |
| b0144 | 927 | 0,54 | 0,621107857 | 5,101255243 | 5,756310786 | 0,016429533 | 0,04559577 |
| b1478 | 1011 | 0,52 | -0,718782227 | 8,279975337 | 5,738822915 | 0,01659391 | 0,046023454 |
| b4069 | 1959 | 0,57 | 1,241275349 | 10,06493691 | 5,728549382 | 0,016691265 | 0,046264842 |
| b0189 | 255 | 0,48 | -0,480952399 | 6,4079888 | 5,711450484 | 0,016854608 | 0,046661652 |
| b2539 | 519 | 0,52 | 1,22240533 | 2,646636905 | 5,711383945 | 0,016855246 | 0,046661652 |
| b0810 | 660 | 0,53 | 0,829904088 | 6,147307935 | 5,706631264 | 0,016900942 | 0,046759273 |
| b0116 | 1425 | 0,53 | 0,781988982 | 11,08155679 | 5,702071233 | 0,016944905 | 0,046851983 |
| b0589 | 993 | 0,58 | 1,003467622 | 2,679701297 | 5,692933489 | 0,017033358 | 0,047067515 |
| b2859 | 399 | 0,36 | 1,846741797 | 1,225453711 | 5,683529963 | 0,01712488 | 0,04729126 |
| b3095 | 663 | 0,51 | -0,523492842 | 5,934235919 | 5,681458344 | 0,017145111 | 0,047317973 |
| b1867 | 567 | 0,53 | 0,68135205 | 5,78784846 | 5,675998246 | 0,017198551 | 0,04743625 |
| b0078 | 492 | 0,5 | 0,988140229 | 3,188766237 | 5,672799513 | 0,017229938 | 0,047493593 |
| b1520 | 915 | 0,48 | -0,596908566 | 5,508753547 | 5,671256027 | 0,017245104 | 0,047503155 |
| b2713 | 528 | 0,52 | 1,425075474 | 1,807703369 | 5,670289903 | 0,017254604 | 0,047503155 |
| b1937 | 315 | 0,54 | 2,313849378 | 1,169860285 | 5,663809447 | 0,017318468 | 0,047649707 |
| b2776 | 1479 | 0,49 | -0,778338217 | 4,039092833 | 5,659380899 | 0,017362251 | 0,047730441 |
| b0716 | 1062 | 0,45 | -0,917344402 | 3,843140175 | 5,65868818 | 0,017369109 | 0,047730441 |
| b1099 | 1005 | 0,54 | 0,511218172 | 5,585013045 | 5,654910816 | 0,01740656 | 0,047804045 |
| b1950 | 786 | 0,47 | 1,535817393 | 1,788289255 | 5,64722855 | 0,017482982 | 0,047984523 |
| b3409 | 2322 | 0,55 | 0,849716499 | 7,44487707 | 5,640762914 | 0,01754757 | 0,048132318 |

| | | | | | | | |
|---|---|---|---|---|---|---|---|
| b2963 | 1080 | 0,51 | 0,66225935 | 4,909937386 | 5,632812228 | 0,01762733 | 0,048321525 |
| b4068 | 315 | 0,49 | 1,439083882 | 5,844273956 | 5,628824117 | 0,017667479 | 0,048401981 |
| b2602 | 366 | 0,46 | -0,695394353 | 5,025636699 | 5,627735262 | 0,017678457 | 0,048402471 |
| b0429 | 330 | 0,5 | 0,814309017 | 9,334364349 | 5,626580222 | 0,01769011 | 0,048404807 |
| b3138 | 477 | 0,44 | 2,507228289 | 0,816767825 | 5,617258369 | 0,017784448 | 0,04863325 |
| b3999 | 591 | 0,49 | 0,494420732 | 6,040965856 | 5,613315172 | 0,01782451 | 0,048713081 |
| b4279 | 1218 | 0,36 | 0,910969897 | 3,798666191 | 5,610573767 | 0,017852417 | 0,048759617 |
| b4026 | 243 | 0,6 | 1,475820576 | 2,33599809 | 5,598726446 | 0,017973538 | 0,049060535 |
| b0177 | 2433 | 0,51 | 0,559392993 | 10,10545554 | 5,588433923 | 0,018079453 | 0,049319604 |
| b3509 | 327 | 0,4 | -0,94321392 | 11,36556386 | 5,581623108 | 0,018149894 | 0,049481645 |
| b1727 | 669 | 0,52 | 0,645052522 | 4,921417257 | 5,577059503 | 0,018197251 | 0,049580596 |
| b1492 | 1536 | 0,48 | -1,253732346 | 12,4752756 | 5,575986516 | 0,018208404 | 0,049580844 |
| b0629 | 954 | 0,45 | 0,731679289 | 3,512362159 | 5,560037725 | 0,018375018 | 0,050004148 |
| b2412 | 987 | 0,55 | -0,537367982 | 8,766603222 | 5,554203437 | 0,018436361 | 0,050128318 |
| b2151 | 1041 | 0,52 | 1,126495935 | 5,518525506 | 5,553571686 | 0,018443016 | 0,050128318 |
| b0731 | 1977 | 0,55 | 1,183894981 | 1,953367711 | 5,529424023 | 0,01869926 | 0,050722977 |
| b2104 | 789 | 0,54 | 0,907986277 | 2,696036653 | 5,529360585 | 0,018699938 | 0,050722977 |
| b1650 | 1098 | 0,53 | 0,617538533 | 6,042920725 | 5,528476584 | 0,018709388 | 0,050722977 |
| b0792 | 1107 | 0,52 | -0,571442922 | 5,407573697 | 5,52768751 | 0,018717828 | 0,050722977 |
| b2306 | 774 | 0,52 | 0,87696492 | 4,460930137 | 5,527635405 | 0,018718386 | 0,050722977 |
| b4319 | 504 | 0,52 | 0,93761226 | 3,359862015 | 5,523964731 | 0,0187577 | 0,050798797 |
| b2274 | 423 | 0,4 | 1,736289281 | 1,46340006 | 5,521149524 | 0,018787909 | 0,050849884 |
| b1828 | 1374 | 0,52 | 1,30529324 | 2,614338869 | 5,520017231 | 0,018800074 | 0,0508521 |
| b0696 | 573 | 0,54 | 1,453296797 | 4,719721519 | 5,512132036 | 0,018885013 | 0,051051041 |
| b4255 | 417 | 0,49 | 0,491916949 | 8,323490874 | 5,508173623 | 0,018927802 | 0,05113587 |
| b0246 | 318 | 0,57 | 1,477405664 | 1,685593894 | 5,502501595 | 0,01898929 | 0,051271082 |
| b0797 | 1365 | 0,57 | 0,742452367 | 3,881812045 | 5,472007311 | 0,019323419 | 0,052141821 |
| b2819 | 1827 | 0,55 | -0,535916652 | 6,475653832 | 5,467792761 | 0,019370074 | 0,052236265 |
| b3028 | 582 | 0,51 | 0,628781964 | 5,639000984 | 5,457600852 | 0,01948338 | 0,052510229 |
| b2223 | 1323 | 0,53 | 1,254860041 | 2,033187289 | 5,44098398 | 0,019669586 | 0,052980218 |
| b3766 | 99 | 0,54 | 1,118319347 | 2,830795143 | 5,435712278 | 0,019729043 | 0,053108451 |
| b0115 | 1893 | 0,55 | -0,776126291 | 9,768464933 | 5,434284341 | 0,01974518 | 0,053119986 |
| b2046 | 1479 | 0,54 | 1,4269003 | 1,996710368 | 5,432200868 | 0,01976875 | 0,053151492 |
| b1651 | 408 | 0,51 | -0,480062873 | 7,730219911 | 5,429140568 | 0,019803423 | 0,053212794 |
| b1746 | 1479 | 0,58 | 1,11337228 | 7,693250741 | 5,426511838 | 0,019833256 | 0,053261028 |
| b1453 | 1500 | 0,52 | 0,895051078 | 3,009282494 | 5,424881605 | 0,019851781 | 0,053278853 |
| b2558 | 1557 | 0,5 | 0,775060833 | 3,946788368 | 5,423293426 | 0,019869846 | 0,053295421 |
| b1874 | 747 | 0,52 | 0,597171594 | 6,012984016 | 5,416307246 | 0,01994951 | 0,053477095 |
| b3618 | 858 | 0,37 | 0,718854434 | 5,521128561 | 5,404764538 | 0,020081857 | 0,053791968 |
| b3622 | 1260 | 0,32 | 0,592992253 | 5,738307792 | 5,403972144 | 0,020090976 | 0,053791968 |
| b4342 | 1503 | 0,44 | -0,647429269 | 7,372298752 | 5,399381864 | 0,020143885 | 0,053901428 |
| b1090 | 1071 | 0,52 | 0,500180327 | 7,060262243 | 5,396787885 | 0,020173847 | 0,053949393 |
| b0288 | 330 | 0,5 | 1,208180165 | 2,332072299 | 5,387392009 | 0,020282763 | 0,054208315 |
| b0018 | 210 | 0,51 | -0,873893941 | 3,493923309 | 5,373754744 | 0,020441928 | 0,054580273 |
| b1797 | 360 | 0,48 | -1,078357024 | 3,570402032 | 5,373382467 | 0,020446291 | 0,054580273 |
| b1493 | 1401 | 0,53 | -1,301622738 | 11,61063792 | 5,36918552 | 0,020495546 | 0,054650509 |
| b0849 | 258 | 0,45 | 0,670290985 | 4,067772906 | 5,369062681 | 0,020496989 | 0,054650509 |
| b2436 | 900 | 0,55 | 0,543001057 | 5,017811587 | 5,365436814 | 0,020539643 | 0,054731678 |
| b2485 | 651 | 0,53 | 1,32214162 | 1,561333306 | 5,363490837 | 0,020562574 | 0,054760223 |
| b2106 | 825 | 0,52 | 1,111545701 | 2,029929298 | 5,359902106 | 0,02060493 | 0,054840438 |
| b0817 | 468 | 0,54 | 0,506107817 | 5,240086332 | 5,341266609 | 0,020826336 | 0,055396817 |
| b1853 | 870 | 0,49 | -0,495400573 | 6,712239897 | 5,336209221 | 0,020886845 | 0,055524817 |
| b4385 | 1332 | 0,54 | -0,54770007 | 6,291743524 | 5,333193281 | 0,020923016 | 0,055588002 |
| b1704 | 1047 | 0,53 | -1,258276323 | 5,782006583 | 5,321042798 | 0,021069399 | 0,055943748 |
| b1596 | 1254 | 0,53 | 0,790774232 | 4,903455769 | 5,313982186 | 0,021154948 | 0,056137642 |
| b1076 | 1209 | 0,52 | 1,038463086 | 2,97305559 | 5,312375275 | 0,021174468 | 0,056156193 |
| b1121 | 789 | 0,43 | 2,076875779 | 1,581832527 | 5,310562021 | 0,021196517 | 0,056181426 |
| b3272 | 120 | 0,62 | -0,74177498 | 9,03816011 | 5,30909276 | 0,021214401 | 0,056195594 |
| b3596 | 462 | 0,32 | 2,182916796 | 0,859535428 | 5,296659535 | 0,021366363 | 0,0565647 |
| b3153 | 519 | 0,5 | -0,519103968 | 6,357161435 | 5,295075877 | 0,021385799 | 0,056582734 |
| b2971 | 411 | 0,44 | 1,656260526 | 1,788868827 | 5,287729431 | 0,021476203 | 0,056788404 |
| b3033 | 423 | 0,48 | 0,565716873 | 5,557392996 | 5,283431992 | 0,02152927 | 0,056895159 |
| b1976 | 798 | 0,46 | -0,588661494 | 8,388507271 | 5,280165863 | 0,021569693 | 0,056968394 |
| b1482 | 432 | 0,5 | -0,70868822 | 10,20300823 | 5,268325012 | 0,021716899 | 0,057304307 |
| b3078 | 1434 | 0,5 | -1,212893584 | 5,372419859 | 5,267880221 | 0,021722449 | 0,057304307 |
| b0442 | 372 | 0,55 | -0,769119841 | 3,735418176 | 5,266567835 | 0,021738833 | 0,057313794 |
| b4182 | 699 | 0,51 | 1,433677702 | 1,582053066 | 5,263470188 | 0,021777555 | 0,057382129 |
| b2061 | 444 | 0,55 | 2,307662837 | 0,698755622 | 5,262420414 | 0,021790694 | 0,057383014 |
| b1681 | 1272 | 0,53 | -0,721708306 | 8,199580767 | 5,259074132 | 0,02183263 | 0,057458256 |
| b3422 | 1599 | 0,56 | 0,673702408 | 3,950384299 | 5,257345797 | 0,021854323 | 0,057458256 |
| b3480 | 807 | 0,56 | -1,562671591 | 4,095061454 | 5,25707485 | 0,021857726 | 0,057458256 |
| b1312 | 843 | 0,53 | 1,133370905 | 1,988432425 | 5,255760972 | 0,021874234 | 0,057467947 |
| b1163 | 1212 | 0,44 | 0,768177204 | 3,905093017 | 5,245316978 | 0,022005919 | 0,05777497 |
| b3432 | 2187 | 0,53 | -0,589258969 | 9,981565206 | 5,243971619 | 0,022022942 | 0,05777497 |
| b1856 | 1323 | 0,52 | 0,516461833 | 6,746239311 | 5,243432349 | 0,022029769 | 0,05777497 |
| b0697 | 2049 | 0,56 | 1,408256593 | 6,25408794 | 5,234384044 | 0,022144648 | 0,058010829 |
| b0329 | 276 | 0,39 | -0,693546411 | 8,889065544 | 5,234310203 | 0,022145589 | 0,058010829 |
| b2784 | 2235 | 0,54 | -0,440200558 | 8,780141041 | 5,229321996 | 0,022209188 | 0,058143447 |

| | | | | | | | |
|---|---|---|---|---|---|---|---|
| b3100 | 300 | 0,57 | -0,559503789 | 9,861186903 | 5,221770925 | 0,022305824 | 0,058355877 |
| b1198 | 1419 | 0,5 | -0,571441555 | 6,726384559 | 5,220888962 | 0,02231714 | 0,058355877 |
| b2377 | 243 | 0,48 | -0,851516252 | 5,792452512 | 5,219934614 | 0,022329391 | 0,058355877 |
| b0847 | 1686 | 0,52 | -0,464483505 | 6,108505591 | 5,218534866 | 0,022347372 | 0,058368834 |
| b0027 | 495 | 0,54 | 0,472856226 | 6,673693959 | 5,209374897 | 0,022465411 | 0,058642964 |
| b1320 | 999 | 0,45 | 1,093693862 | 3,302882142 | 5,190825503 | 0,022706428 | 0,059207507 |
| b0863 | 732 | 0,5 | -0,527031055 | 9,557777359 | 5,190697535 | 0,0227081 | 0,059207507 |
| b1292 | 891 | 0,57 | -0,580156163 | 5,140062772 | 5,184915005 | 0,022783787 | 0,059370309 |
| b3715 | 666 | 0,48 | 0,599665145 | 4,111267445 | 5,183111211 | 0,02280745 | 0,059397437 |
| b2889 | 549 | 0,5 | -0,626072856 | 5,940938675 | 5,17925648 | 0,022858103 | 0,059494784 |
| b2764 | 1800 | 0,56 | 0,873586857 | 3,323772603 | 5,169763245 | 0,022983348 | 0,059786051 |
| b1444 | 1425 | 0,53 | -0,778353393 | 9,804566896 | 5,156225201 | 0,023163188 | 0,060218915 |
| b4057 | 357 | 0,5 | -0,579246343 | 7,434498286 | 5,153456453 | 0,023200148 | 0,060257696 |
| b2638 | 153 | 0,44 | 1,524940638 | 1,541107001 | 5,153093731 | 0,023204994 | 0,060257696 |
| b2407 | 834 | 0,52 | 1,474045107 | 1,795707879 | 5,146390416 | 0,023294748 | 0,06045574 |
| b0262 | 1047 | 0,56 | 1,005109156 | 2,410330091 | 5,142538442 | 0,023346487 | 0,060554951 |
| b2214 | 1056 | 0,53 | 0,995472202 | 3,757510533 | 5,133858052 | 0,023463518 | 0,0608233 |
| b3369 | 168 | 0,42 | 1,863485453 | 1,308469091 | 5,130526765 | 0,023508592 | 0,060904919 |
| b3553 | 975 | 0,52 | 0,687906392 | 7,592281449 | 5,129159755 | 0,023527114 | 0,060917693 |
| b1034 | 738 | 0,48 | 0,452559717 | 6,857808933 | 5,117833971 | 0,023681158 | 0,061281148 |
| b2534 | 855 | 0,51 | 1,186113406 | 2,128191957 | 5,100350255 | 0,023921013 | 0,061866116 |
| b2561 | 849 | 0,52 | 0,741390624 | 3,990811129 | 5,090061284 | 0,024063341 | 0,062198325 |
| b2870 | 1191 | 0,5 | -1,319256182 | 2,614821791 | 5,088460412 | 0,024085565 | 0,062219887 |
| b2725 | 462 | 0,5 | 2,018394494 | 0,91640815 | 5,086992933 | 0,024105956 | 0,062236691 |
| b2981 | 1101 | 0,51 | 0,962582531 | 3,079139391 | 5,08390119 | 0,024148975 | 0,062258802 |
| b4376 | 606 | 0,52 | -0,671062045 | 11,55308333 | 5,083402776 | 0,024155917 | 0,062258802 |
| b1649 | 600 | 0,52 | 0,84744596 | 3,683743679 | 5,083382995 | 0,024156193 | 0,062258802 |
| b2716 | 1425 | 0,52 | -0,584250663 | 4,660435336 | 5,08144989 | 0,024183139 | 0,06229243 |
| b4187 | 1626 | 0,54 | -0,853805379 | 9,931910069 | 5,080439398 | 0,024197237 | 0,062292944 |
| b3656 | 2319 | 0,52 | -0,59190223 | 4,775378824 | 5,074876076 | 0,024275007 | 0,06245728 |
| b3501 | 354 | 0,51 | 1,426799541 | 1,821306931 | 5,072110345 | 0,024313766 | 0,062521113 |
| b3084 | 1137 | 0,54 | 0,60550154 | 4,03507279 | 5,07097747 | 0,024329661 | 0,062526112 |
| b0052 | 990 | 0,56 | 0,46608615 | 6,790145931 | 5,0681923 | 0,024368783 | 0,062590767 |
| b2562 | 261 | 0,45 | 1,740713065 | 1,897342579 | 5,057551799 | 0,024518851 | 0,062940142 |
| b2113 | 1110 | 0,54 | 0,430914542 | 8,478562461 | 5,056417595 | 0,024534903 | 0,062945298 |
| b1554 | 534 | 0,51 | 2,259510405 | 0,815103739 | 5,051138089 | 0,024609769 | 0,063101249 |
| b4060 | 282 | 0,52 | 1,094477552 | 2,768161221 | 5,049374614 | 0,024634828 | 0,063129388 |
| b4559 | 174 | 0,35 | -1,367674113 | 2,478734952 | 5,047995084 | 0,02465445 | 0,063143569 |
| b0755 | 753 | 0,51 | -0,57211112 | 10,79708721 | 5,040446876 | 0,024762101 | 0,06338306 |
| b3867 | 1374 | 0,53 | 0,382058246 | 7,432969003 | 5,034904605 | 0,024841455 | 0,063549886 |
| b3137 | 861 | 0,52 | 1,301525292 | 2,017517839 | 5,032993927 | 0,024868873 | 0,063583735 |
| b2786 | 2757 | 0,5 | -0,397606275 | 6,499041742 | 5,013309063 | 0,025153184 | 0,064273985 |
| b3038 | 1161 | 0,5 | 0,445438836 | 8,162641203 | 5,01158285 | 0,025178276 | 0,064287397 |
| b1680 | 1221 | 0,55 | -0,714711585 | 7,612221011 | 5,010974906 | 0,025187119 | 0,064287397 |
| b4115 | 1338 | 0,54 | -0,828907425 | 3,540299105 | 5,005772478 | 0,025262927 | 0,064444188 |
| b1787 | 504 | 0,51 | 0,586426575 | 5,485844558 | 4,999613862 | 0,025352974 | 0,064637105 |
| b0110 | 552 | 0,54 | -0,48932821 | 5,572116233 | 4,998623953 | 0,025367479 | 0,064637318 |
| b1614 | 1509 | 0,49 | -0,571020613 | 10,01576976 | 4,996747295 | 0,025395001 | 0,064670679 |
| b4694 | 339 | 0,5 | 0,890535595 | 2,597547241 | 4,98581063 | 0,025556009 | 0,065043743 |
| b1780 | 885 | 0,5 | -0,726997899 | 7,319805011 | 4,984376453 | 0,025577201 | 0,065060735 |
| b3916 | 963 | 0,53 | -0,528180353 | 9,600136265 | 4,966253241 | 0,025846579 | 0,065708659 |
| b2121 | 1137 | 0,54 | 1,646444737 | 1,887762226 | 4,963117517 | 0,025893485 | 0,06579059 |
| b2029 | 1407 | 0,5 | 0,727611339 | 10,44049934 | 4,958684308 | 0,025959952 | 0,065877006 |
| b4086 | 981 | 0,52 | 0,883768894 | 2,661801257 | 4,958503378 | 0,025962668 | 0,065877006 |
| b2132 | 2298 | 0,54 | 0,43507094 | 7,20780943 | 4,957909213 | 0,025971591 | 0,065877006 |
| b4150 | 1134 | 0,5 | 0,705294206 | 4,468982682 | 4,956662216 | 0,025990328 | 0,065887244 |
| b3428 | 2448 | 0,51 | -0,531139327 | 10,02237429 | 4,945467325 | 0,026159166 | 0,066277774 |
| b2154 | 837 | 0,54 | 0,489056493 | 6,561256968 | 4,939921878 | 0,026243223 | 0,066453178 |
| b0980 | 1299 | 0,54 | 0,658729702 | 6,453906154 | 4,933218477 | 0,026345206 | 0,066673752 |
| b4539 | 255 | 0,44 | 0,621952757 | 4,486389417 | 4,925208447 | 0,026467609 | 0,066945724 |
| b3497 | 753 | 0,58 | 0,582660644 | 5,681350073 | 4,905764218 | 0,026767205 | 0,06766532 |
| b0082 | 942 | 0,55 | -0,422397845 | 7,702333111 | 4,898652908 | 0,026877654 | 0,067906227 |
| b2374 | 1251 | 0,47 | 1,068393848 | 2,176188688 | 4,892719896 | 0,026970165 | 0,068101567 |
| b2754 | 285 | 0,46 | 0,954629791 | 2,644497913 | 4,880680997 | 0,027158902 | 0,068539526 |
| b0698 | 1674 | 0,55 | 1,655335797 | 4,971286493 | 4,875826598 | 0,027235394 | 0,068693886 |
| b0649 | 1452 | 0,48 | 0,812832822 | 3,553601007 | 4,871827639 | 0,027298574 | 0,068779998 |
| b2778 | 873 | 0,41 | 1,592177042 | 1,038694837 | 4,871723173 | 0,027300227 | 0,068779998 |
| b2406 | 1257 | 0,48 | -0,896733396 | 4,423932012 | 4,867621874 | 0,027365187 | 0,068904926 |
| b0150 | 2244 | 0,51 | 0,82266314 | 6,235643124 | 4,85652242 | 0,027541797 | 0,069310688 |
| b0005 | 297 | 0,54 | 0,943821032 | 2,782404582 | 4,852468871 | 0,027606591 | 0,069434759 |
| b1692 | 867 | 0,48 | 1,368232104 | 1,827597047 | 4,8503144 | 0,027641094 | 0,069482547 |
| b3866 | 510 | 0,52 | -0,455110835 | 7,39692116 | 4,8436229 | 0,02774854 | 0,069701704 |
| b1927 | 1488 | 0,5 | 0,488542918 | 8,974267117 | 4,842949321 | 0,02775938 | 0,069701704 |
| b0111 | 855 | 0,52 | -0,4264514 | 6,321616295 | 4,83933967 | 0,027817545 | 0,069772133 |
| b2553 | 339 | 0,51 | 0,470292204 | 6,041442885 | 4,839276522 | 0,027818564 | 0,069772133 |
| b2238 | 243 | 0,41 | 1,730754045 | 1,220520055 | 4,83580225 | 0,027874669 | 0,06987375 |
| b3268 | 1025 | 0,5 | -0,759929255 | 4,693817028 | 4,830521788 | 0,027960167 | 0,070023516 |
| b1835 | 1440 | 0,52 | 0,774431792 | 4,478386982 | 4,830183054 | 0,027965661 | 0,070023516 |

| | | | | | | | |
|---|---|---|---|---|---|---|---|
| b1057 | 567 | 0,51 | 0,970339052 | 2,790441957 | 4,815822328 | 0,028199614 | 0,070569887 |
| b1491 | 1320 | 0,52 | 0,461563862 | 5,554174025 | 4,803073643 | 0,028409011 | 0,071054233 |
| b4579 | 2029 | 0,45 | 1,411567884 | 1,630703026 | 4,789538428 | 0,028633099 | 0,071574762 |
| b3455 | 768 | 0,55 | 0,997551129 | 2,424839191 | 4,78513224 | 0,028706443 | 0,071718104 |
| b4516 | 276 | 0,53 | 1,735825848 | 0,863557416 | 4,782123635 | 0,028756637 | 0,071798673 |
| b2636 | 444 | 0,51 | 1,668885466 | 1,312416138 | 4,781279778 | 0,028770731 | 0,071798673 |
| b4481 | 1080 | 0,53 | 0,432020963 | 5,504709169 | 4,770469872 | 0,028951923 | 0,072210639 |
| b1014 | 3963 | 0,57 | 1,183204449 | 11,70851826 | 4,766668791 | 0,029015917 | 0,072330001 |
| b2224 | 1185 | 0,53 | 0,924346084 | 2,738549556 | 4,758300994 | 0,029157316 | 0,072642073 |
| b0385 | 1116 | 0,54 | 0,785506988 | 3,014510195 | 4,753879108 | 0,029232326 | 0,072788492 |
| b4659 | 796 | 0,43 | 0,620323083 | 4,883445636 | 4,749572934 | 0,029305567 | 0,072930344 |
| b4225 | 351 | 0,54 | -0,581828012 | 4,961522609 | 4,745827022 | 0,029369434 | 0,073034656 |
| b2325 | 279 | 0,53 | 0,795664622 | 4,131102091 | 4,74520386 | 0,029380072 | 0,073034656 |
| b2846 | 633 | 0,37 | 1,359422958 | 1,761918702 | 4,740095756 | 0,029467431 | 0,073211212 |
| b3719 | 1170 | 0,5 | 1,020100637 | 2,588266084 | 4,73882394 | 0,029489224 | 0,073224765 |
| b4480 | 840 | 0,48 | -0,540266098 | 5,252608028 | 4,732650039 | 0,029595252 | 0,073447354 |
| b4139 | 1437 | 0,49 | -0,86398151 | 12,53720448 | 4,727503854 | 0,029683935 | 0,073626672 |
| b0416 | 420 | 0,54 | 0,435501971 | 6,927080824 | 4,72280389 | 0,02976517 | 0,07378733 |
| b3374 | 786 | 0,57 | -0,783267141 | 3,728136786 | 4,719987896 | 0,029813953 | 0,073867406 |
| b1092 | 930 | 0,55 | 0,44580662 | 9,069889549 | 4,714011547 | 0,029917761 | 0,074083648 |
| b4018 | 825 | 0,54 | 0,47758771 | 7,108586155 | 4,712911675 | 0,029936906 | 0,074090124 |
| b0386 | 810 | 0,55 | 0,432722353 | 7,101266668 | 4,704472408 | 0,030084234 | 0,074393601 |
| b1519 | 759 | 0,54 | -0,431631142 | 7,129399963 | 4,703987304 | 0,030092726 | 0,074393601 |
| b0984 | 2097 | 0,52 | 0,704721505 | 3,696629412 | 4,700414574 | 0,030155343 | 0,074507302 |
| b3418 | 2706 | 0,54 | -0,641942593 | 10,6872312 | 4,697462254 | 0,030207188 | 0,07459428 |
| b1199 | 633 | 0,54 | -0,632649676 | 5,726652873 | 4,692008889 | 0,030303199 | 0,074790164 |
| b3073 | 1380 | 0,55 | -0,522780355 | 8,320526311 | 4,680249436 | 0,030511318 | 0,07526237 |
| b3731 | 420 | 0,55 | 0,833831549 | 9,713664534 | 4,670008969 | 0,030693767 | 0,075670773 |
| b4041 | 2424 | 0,55 | -0,512389225 | 8,736793924 | 4,664473064 | 0,030792871 | 0,075873363 |
| b4360 | 495 | 0,51 | 0,639371568 | 5,030892439 | 4,660142187 | 0,030870635 | 0,076021275 |
| b0140 | 741 | 0,44 | 2,018080252 | 1,090598565 | 4,65924205 | 0,030886823 | 0,076021275 |
| b1841 | 375 | 0,5 | 0,563038188 | 5,453810876 | 4,654342409 | 0,030975095 | 0,076196693 |
| b1144 | 309 | 0,47 | 0,764218177 | 3,257918303 | 4,644593881 | 0,031151507 | 0,076553688 |
| b0928 | 1191 | 0,52 | 0,67177816 | 9,428113594 | 4,644435667 | 0,031154379 | 0,076553688 |
| b1389 | 288 | 0,5 | 1,809971106 | 3,736108468 | 4,640200403 | 0,031231355 | 0,076700786 |
| b2439 | 660 | 0,56 | -0,649671613 | 5,558871211 | 4,636740757 | 0,031294382 | 0,076781204 |
| b0161 | 1425 | 0,54 | 0,76705024 | 8,623809947 | 4,63652251 | 0,031298362 | 0,076781204 |
| b2222 | 651 | 0,54 | 1,92639575 | 0,842146059 | 4,62990532 | 0,031419297 | 0,077035717 |
| b2458 | 1017 | 0,58 | -0,707414909 | 3,776338054 | 4,625005245 | 0,031509165 | 0,07721382 |
| b3858 | 270 | 0,46 | -0,534660675 | 6,371870196 | 4,623367614 | 0,031539259 | 0,077245332 |
| b4059 | 537 | 0,58 | -0,47945586 | 8,545646564 | 4,620806969 | 0,031586375 | 0,077318477 |
| b4329 | 462 | 0,53 | 0,925390721 | 2,432918385 | 4,615236305 | 0,031689129 | 0,077527661 |
| b0739 | 1266 | 0,52 | 0,410174498 | 6,511647951 | 4,613908394 | 0,031713674 | 0,077528791 |
| b3344 | 360 | 0,5 | 0,662530311 | 4,153850327 | 4,613340014 | 0,031724186 | 0,077528791 |
| b2938 | 1977 | 0,54 | 0,510322404 | 9,382747373 | 4,611396437 | 0,03176016 | 0,077574408 |
| b3280 | 258 | 0,49 | 0,71399991 | 4,467715682 | 4,608154388 | 0,031820262 | 0,077678876 |
| b2894 | 897 | 0,53 | -0,381048266 | 6,283855482 | 4,604268833 | 0,03189245 | 0,077812718 |
| b3346 | 723 | 0,47 | 0,423583134 | 6,950662745 | 4,602339954 | 0,03192835 | 0,077857923 |
| b2363 | 306 | 0,43 | 1,377716052 | 1,411733735 | 4,597672869 | 0,032015385 | 0,078018904 |
| b3147 | 2037 | 0,54 | 0,387655677 | 7,74046947 | 4,596934397 | 0,03202918 | 0,078018904 |
| b0963 | 459 | 0,53 | -0,430720945 | 7,902254078 | 4,592725375 | 0,032107921 | 0,078168225 |
| b2423 | 876 | 0,57 | 0,972655918 | 2,588079667 | 4,590411361 | 0,032151297 | 0,078226684 |
| b3967 | 858 | 0,52 | -0,434797644 | 6,378925348 | 4,589582954 | 0,03216684 | 0,078226684 |
| b0251 | 444 | 0,51 | 0,995212084 | 2,399591008 | 4,586681962 | 0,032221333 | 0,07831671 |
| b1667 | 306 | 0,45 | 0,465969279 | 7,549268064 | 4,579027066 | 0,032365587 | 0,078624694 |
| b1450 | 666 | 0,44 | 0,916631155 | 4,86022252 | 4,568131514 | 0,032572073 | 0,079083442 |
| b0624 | 384 | 0,48 | 0,631746408 | 4,965601317 | 4,561985868 | 0,032689149 | 0,079324723 |
| b4318 | 531 | 0,5 | 0,80818891 | 3,348478248 | 4,555841231 | 0,032806644 | 0,079566763 |
| b1154 | 630 | 0,5 | 1,908889179 | 1,19411108 | 4,547811048 | 0,032960859 | 0,07989755 |
| b1156 | 603 | 0,48 | 1,538843186 | 1,868532056 | 4,54307423 | 0,033052182 | 0,08007561 |
| b2567 | 681 | 0,51 | -0,491892634 | 7,16161985 | 4,541356985 | 0,033085354 | 0,080095055 |
| b1931 | 669 | 0,52 | -0,700866648 | 4,862749202 | 4,540808923 | 0,033095948 | 0,080095055 |
| b2505 | 519 | 0,47 | 1,33038368 | 1,347911103 | 4,532800264 | 0,033251166 | 0,080427267 |
| b0073 | 1092 | 0,55 | 0,775274228 | 4,9110525 | 4,527545115 | 0,03335343 | 0,080631108 |
| b1051 | 375 | 0,5 | -0,667616292 | 9,917815497 | 4,523311797 | 0,033436049 | 0,080787261 |
| b1686 | 411 | 0,52 | 0,570696933 | 5,984537563 | 4,516032216 | 0,033578619 | 0,081088022 |
| b0994 | 1029 | 0,53 | -0,587108468 | 4,344167208 | 4,511192035 | 0,033673765 | 0,081273998 |
| b0228 | 498 | 0,45 | -0,560324933 | 4,295181061 | 4,505731452 | 0,033781445 | 0,081483971 |
| b1094 | 237 | 0,48 | 0,593019624 | 10,51639528 | 4,504937962 | 0,033797122 | 0,081483971 |
| b2929 | 510 | 0,48 | 0,443285 | 5,222085828 | 4,501660364 | 0,033861959 | 0,081596398 |
| b4012 | 444 | 0,55 | -0,659979241 | 4,570100401 | 4,500134603 | 0,033892186 | 0,081598229 |
| b3593 | 4134 | 0,6 | -0,809103976 | 3,660329884 | 4,499784238 | 0,033899131 | 0,081598229 |
| b1399 | 951 | 0,5 | -0,415216555 | 6,932844442 | 4,49828193 | 0,033928927 | 0,081626113 |
| b3105 | 897 | 0,55 | 0,596854357 | 5,37031077 | 4,491048308 | 0,034072778 | 0,081928213 |
| b2069 | 1353 | 0,56 | 0,62839799 | 3,961132063 | 4,487729255 | 0,034138995 | 0,082043419 |
| b1916 | 723 | 0,47 | 0,462746554 | 5,202675335 | 4,481188165 | 0,034269888 | 0,082313848 |
| b0317 | 498 | 0,53 | -1,290773489 | 2,070179962 | 4,478876651 | 0,034316269 | 0,082381103 |
| b0239 | 1245 | 0,54 | -0,436142986 | 7,093506618 | 4,47652101 | 0,034363603 | 0,082450573 |

| | | | | | | | |
|---|---|---|---|---|---|---|---|
| b3568 | 1182 | 0,54 | -0,846028277 | 4,499535553 | 4,474643527 | 0,034401378 | 0,082497044 |
| b3398 | 2136 | 0,53 | 0,447806464 | 6,144637449 | 4,462893764 | 0,03463877 | 0,083021907 |
| b2579 | 384 | 0,48 | -0,86784789 | 7,844073954 | 4,457533492 | 0,034747637 | 0,083238326 |
| b1184 | 1269 | 0,51 | 0,638096502 | 4,06683399 | 4,447928408 | 0,034943611 | 0,08366307 |
| b0468 | 378 | 0,52 | 0,756041664 | 3,734503498 | 4,445026289 | 0,035003051 | 0,08374923 |
| b2042 | 894 | 0,52 | 0,536789569 | 8,638977388 | 4,444347491 | 0,035016969 | 0,08374923 |
| b2060 | 2163 | 0,53 | 0,975505636 | 2,38133212 | 4,434623579 | 0,035216985 | 0,08418268 |
| b4546 | 219 | 0,52 | 0,928559897 | 3,509432264 | 4,431429135 | 0,035282953 | 0,084295413 |
| b0224 | 741 | 0,48 | 0,581857129 | 6,173906281 | 4,42371128 | 0,035442868 | 0,084632358 |
| b1473 | 882 | 0,5 | 0,80462124 | 3,522267094 | 4,420961581 | 0,035500026 | 0,084723703 |
| b3332 | 984 | 0,5 | -0,761542247 | 3,481930382 | 4,406406675 | 0,035804185 | 0,085374447 |
| b1243 | 1632 | 0,48 | -0,586549365 | 12,54392148 | 4,406092035 | 0,03581079 | 0,085374447 |
| b0605 | 564 | 0,5 | -0,545846541 | 11,58779597 | 4,401823679 | 0,035900521 | 0,085542868 |
| b1584 | 561 | 0,47 | -0,456098449 | 6,076964273 | 4,392518905 | 0,036096946 | 0,085965202 |
| b4336 | 1281 | 0,53 | -0,50355291 | 6,183633736 | 4,388122461 | 0,036190146 | 0,086141388 |
| b1237 | 414 | 0,47 | 0,486296613 | 10,01916362 | 4,377548968 | 0,036415327 | 0,086598181 |
| b3467 | 360 | 0,51 | 0,720222353 | 4,153751183 | 4,377297552 | 0,036420699 | 0,086598181 |
| b4425 | 171 | 0,58 | 1,15215273 | 2,726702906 | 4,362787005 | 0,036732164 | 0,08729245 |
| b4347 | 342 | 0,56 | 1,090616857 | 2,011983018 | 4,361008343 | 0,036770534 | 0,087337326 |
| b1485 | 897 | 0,53 | 1,089942003 | 3,844517201 | 4,346814749 | 0,037078232 | 0,088021523 |
| b3529 | 1989 | 0,53 | -0,389804118 | 6,505203644 | 4,341175979 | 0,03720122 | 0,088266738 |
| b3881 | 879 | 0,54 | -0,675727124 | 3,528351687 | 4,334007056 | 0,0373582 | 0,088592303 |
| b0128 | 771 | 0,51 | 0,445884278 | 5,828269096 | 4,332120213 | 0,037399632 | 0,088643654 |
| b3571 | 2031 | 0,53 | -0,526728351 | 4,975170415 | 4,329475442 | 0,037457788 | 0,08871824 |
| b1798 | 639 | 0,45 | 0,986688934 | 3,753503259 | 4,32888932 | 0,037470689 | 0,08871824 |
| b1905 | 498 | 0,43 | -0,816770922 | 8,178151332 | 4,326264807 | 0,037528514 | 0,088808236 |
| b1254 | 540 | 0,46 | 0,523681486 | 5,084281893 | 4,320463728 | 0,037656659 | 0,089045805 |
| b3324 | 816 | 0,42 | 1,010679094 | 2,287453605 | 4,319922372 | 0,037668641 | 0,089045805 |
| b3197 | 987 | 0,52 | 0,352919934 | 7,092245864 | 4,318907823 | 0,037691106 | 0,089051945 |
| b2540 | 321 | 0,55 | 1,179203188 | 1,92170569 | 4,314779077 | 0,037782677 | 0,089221263 |
| b1461 | 234 | 0,43 | -0,659988839 | 4,304152267 | 4,302130101 | 0,038064669 | 0,089839834 |
| b3438 | 996 | 0,54 | -0,460509271 | 7,104755195 | 4,301131339 | 0,038087029 | 0,089845296 |
| b0706 | 762 | 0,4 | 1,860872341 | 1,011972915 | 4,29598498 | 0,038202461 | 0,09007019 |
| b2676 | 960 | 0,48 | 0,945626502 | 3,047635912 | 4,292403313 | 0,038283014 | 0,090212655 |
| b0614 | 552 | 0,58 | 1,692024833 | 0,987000186 | 4,289201574 | 0,038355173 | 0,090335201 |
| b2863 | 1137 | 0,42 | 0,819635194 | 3,786177759 | 4,284402032 | 0,03846361 | 0,090543015 |
| b3192 | 636 | 0,52 | 0,417513393 | 7,749782935 | 4,275287871 | 0,038670413 | 0,090982043 |
| b3411 | 879 | 0,48 | 0,709937445 | 3,694398362 | 4,273578266 | 0,038709334 | 0,090987943 |
| b1381 | 2640 | 0,52 | -0,412404805 | 6,355702473 | 4,273394399 | 0,038713523 | 0,090987943 |
| b2270 | 1728 | 0,47 | 0,749182528 | 3,358559871 | 4,27036751 | 0,038782542 | 0,091102386 |
| b0487 | 408 | 0,59 | -0,614381929 | 7,003337369 | 4,269337688 | 0,038806053 | 0,091109864 |
| b3034 | 630 | 0,51 | 0,554200046 | 5,76630787 | 4,259559096 | 0,03903005 | 0,091587793 |
| b1419 | 174 | 0,44 | -0,670941301 | 3,817695461 | 4,242778914 | 0,039417598 | 0,092448809 |
| b2333 | 540 | 0,49 | 1,464446842 | 1,573444848 | 4,240583247 | 0,039468606 | 0,092520027 |
| b3262 | 885 | 0,47 | 0,951816597 | 2,401764979 | 4,236803914 | 0,039556567 | 0,092677748 |
| b4544 | 336 | 0,54 | -0,740273603 | 3,824677079 | 4,232931008 | 0,039646919 | 0,092840903 |
| b4046 | 516 | 0,52 | -0,720369222 | 6,300226099 | 4,23190103 | 0,039670984 | 0,092848746 |
| b2098 | 1278 | 0,5 | 0,698846927 | 3,596725498 | 4,229536376 | 0,039726291 | 0,092929663 |
| b4363 | 474 | 0,51 | -0,749493678 | 3,265889849 | 4,222774203 | 0,039884899 | 0,093252018 |
| b0091 | 1476 | 0,55 | -0,414270034 | 8,087564071 | 4,219052849 | 0,039972468 | 0,093408029 |
| b3417 | 2394 | 0,52 | -0,493608184 | 9,681284562 | 4,214302242 | 0,040084549 | 0,093621131 |
| b1530 | 435 | 0,49 | 1,02623121 | 2,622576395 | 4,198562864 | 0,040458253 | 0,094444734 |
| b4409 | 126 | 0,43 | -0,849464113 | 3,867674367 | 4,187777913 | 0,040716433 | 0,094997945 |
| b2148 | 1011 | 0,5 | 1,779193211 | 7,3334746 | 4,184643883 | 0,040791782 | 0,095124228 |
| b0210 | 624 | 0,49 | -0,771216791 | 4,626586749 | 4,177631016 | 0,040960918 | 0,095439711 |
| b2322 | 1179 | 0,57 | 0,945544122 | 2,958073446 | 4,177269481 | 0,040969657 | 0,095439711 |
| b4627 | 222 | 0,55 | 1,510277353 | 1,336857368 | 4,168391705 | 0,041184877 | 0,095891229 |
| b3626 | 1017 | 0,34 | -0,446090277 | 5,554438887 | 4,164015184 | 0,041291411 | 0,096089358 |
| b0039 | 1143 | 0,54 | -0,691881842 | 5,312519305 | 4,156765423 | 0,041468524 | 0,096451439 |
| b2872 | 1212 | 0,51 | -0,703801558 | 3,02572191 | 4,15487852 | 0,041514752 | 0,096508878 |
| b4400 | 1353 | 0,5 | 0,699310121 | 3,391741521 | 4,150344481 | 0,041626055 | 0,096694337 |
| b2464 | 951 | 0,5 | -0,565719094 | 9,527491711 | 4,149871748 | 0,041637677 | 0,096694337 |
| b2478 | 879 | 0,52 | 0,415253296 | 8,631011742 | 4,146739823 | 0,041714767 | 0,096823192 |
| b4609 | 143 | 0,41 | 0,52602013 | 5,351399478 | 4,145452324 | 0,041746501 | 0,096846695 |
| b4043 | 609 | 0,52 | -0,448751117 | 7,681761898 | 4,141239424 | 0,041850516 | 0,097037773 |
| b3244 | 1446 | 0,55 | -0,449044374 | 8,154434174 | 4,139552129 | 0,041892252 | 0,097084319 |
| b1875 | 567 | 0,51 | 0,620598187 | 4,724098777 | 4,137859546 | 0,041934162 | 0,097131222 |
| b2807 | 396 | 0,55 | -0,518202729 | 4,521886802 | 4,130924708 | 0,042106337 | 0,097479649 |
| b3670 | 291 | 0,48 | 0,827871573 | 4,963189332 | 4,127979973 | 0,042179671 | 0,097599013 |
| b0574 | 1224 | 0,56 | 1,162177362 | 2,433406763 | 4,126492284 | 0,042216772 | 0,097634453 |
| b2793 | 546 | 0,48 | -0,468817378 | 5,337984531 | 4,120437185 | 0,042368128 | 0,097933961 |
| b4174 | 1260 | 0,56 | 0,462616369 | 9,311388338 | 4,106773231 | 0,04271178 | 0,098677421 |
| b2924 | 861 | 0,5 | 0,880304229 | 8,241575744 | 4,103210187 | 0,042801872 | 0,098834617 |
| b1467 | 1545 | 0,54 | -0,781154783 | 7,989944136 | 4,096932353 | 0,042961095 | 0,099151199 |
| b0096 | 918 | 0,51 | -0,483210265 | 10,52255019 | 4,094571056 | 0,043021145 | 0,099238688 |
| b2509 | 1371 | 0,53 | 0,54523303 | 5,323948905 | 4,091502974 | 0,043099301 | 0,099367833 |
| b4000 | 273 | 0,48 | 0,489753367 | 9,165310407 | 4,082063833 | 0,043340691 | 0,099872995 |
| b1641 | 468 | 0,51 | -0,471634471 | 9,697099834 | 4,073885753 | 0,043550981 | 0,100286236 |

| | | | | | | | |
|---|---|---|---|---|---|---|---|
| b2892 | 1734 | 0,57 | 0,397949531 | 7,007187423 | 4,07335093 | 0,04356477 | 0,100286236 |
| b4166 | 1140 | 0,53 | -0,428694111 | 7,092783604 | 4,069581428 | 0,043662092 | 0,100458675 |
| b2881 | 2871 | 0,53 | -0,491159985 | 4,889803409 | 4,064141717 | 0,04380294 | 0,100705354 |
| b4535 | 108 | 0,48 | 1,882718827 | 0,890989461 | 4,063705942 | 0,043814244 | 0,100705354 |
| b0768 | 954 | 0,45 | 0,853020554 | 2,898174987 | 4,056993371 | 0,043988755 | 0,101000452 |
| b3800 | 1236 | 0,53 | 0,715078463 | 3,396013896 | 4,056965906 | 0,043989471 | 0,101000452 |
| b1884 | 861 | 0,54 | 1,356907259 | 1,747804196 | 4,056168999 | 0,044010237 | 0,101000452 |
| b3224 | 1491 | 0,55 | -0,695572725 | 5,985733173 | 4,050289179 | 0,044163779 | 0,101300951 |
| b4686 | 111 | 0,51 | 0,669897203 | 5,009912748 | 4,04939565 | 0,044187161 | 0,10130274 |
| b0285 | 957 | 0,61 | 0,780487543 | 4,212872631 | 4,046638619 | 0,044259391 | 0,101394644 |
| b2424 | 834 | 0,56 | 1,079692206 | 2,409113277 | 4,046138964 | 0,044272494 | 0,101394644 |
| b3152 | 636 | 0,55 | -0,55887496 | 5,682667548 | 4,031745808 | 0,044651712 | 0,102210916 |
| b3824 | 621 | 0,52 | 0,855580155 | 3,291230288 | 4,025882647 | 0,044807168 | 0,102508753 |
| b2136 | 579 | 0,54 | -0,448046669 | 5,692436215 | 4,025114874 | 0,044827567 | 0,102508753 |
| b3475 | 588 | 0,56 | -0,545180988 | 5,026132401 | 4,017779442 | 0,045022955 | 0,102903052 |
| b3425 | 327 | 0,54 | 1,115256916 | 3,534392423 | 4,01686865 | 0,045047278 | 0,102906167 |
| b2490 | 414 | 0,53 | 1,026664265 | 1,880425401 | 4,00189984 | 0,045449007 | 0,103770989 |
| b4321 | 1344 | 0,53 | -0,585385388 | 4,793607211 | 3,979816305 | 0,046048585 | 0,105086435 |
| b1355 | 135 | 0,47 | 1,863287733 | 0,700377371 | 3,974955642 | 0,04618167 | 0,105336511 |
| b3333 | 1164 | 0,49 | -0,621908679 | 4,317488984 | 3,971001689 | 0,046290228 | 0,105530418 |
| b0528 | 213 | 0,55 | 0,764242015 | 3,730443759 | 3,967769275 | 0,046379175 | 0,105679443 |
| b0527 | 522 | 0,53 | 0,934991442 | 2,19532896 | 3,965476745 | 0,046442369 | 0,105769664 |
| b0349 | 867 | 0,55 | 0,738199428 | 2,616132524 | 3,961683732 | 0,046547123 | 0,105954396 |
| b3941 | 891 | 0,52 | 1,132070133 | 4,088123693 | 3,949123723 | 0,046895782 | 0,106693855 |
| b2501 | 2067 | 0,49 | -0,344105325 | 7,937538202 | 3,945611541 | 0,04699377 | 0,106862546 |
| b4392 | 1938 | 0,55 | -0,414746021 | 8,330111115 | 3,940020243 | 0,04715021 | 0,107163916 |
| b3653 | 1206 | 0,54 | -0,450279766 | 6,917464996 | 3,937862801 | 0,04721072 | 0,10724706 |
| b2543 | 423 | 0,5 | 0,493763755 | 6,533974258 | 3,934024068 | 0,047318589 | 0,107437647 |
| b3819 | 891 | 0,51 | 1,029131875 | 3,387839676 | 3,929792758 | 0,04743779 | 0,107653759 |
| b3793 | 1353 | 0,52 | 0,449189284 | 5,054561423 | 3,923604732 | 0,047612685 | 0,107995978 |
| b0350 | 810 | 0,55 | 0,966277576 | 2,384751058 | 3,922698473 | 0,047638356 | 0,10799955 |
| b1070 | 417 | 0,48 | 0,893483591 | 2,61399412 | 3,920041058 | 0,047713715 | 0,108115708 |
| b2383 | 2496 | 0,55 | 0,625448277 | 3,730527652 | 3,919140906 | 0,04773927 | 0,108118953 |
| b1460 | 1137 | 0,42 | 1,844591621 | 1,081872346 | 3,910716824 | 0,047979129 | 0,1086073 |
| b4390 | 1233 | 0,51 | -0,502229669 | 7,689184511 | 3,909397997 | 0,048016794 | 0,108637694 |
| b3862 | 933 | 0,45 | 0,822697499 | 2,841390025 | 3,905882491 | 0,04811735 | 0,108810275 |
| b2481 | 618 | 0,56 | 1,614583879 | 1,222645466 | 3,903697256 | 0,048179968 | 0,108896932 |
| b1185 | 531 | 0,53 | 0,577105604 | 5,038732166 | 3,902474216 | 0,048215051 | 0,1089213 |
| b2372 | 945 | 0,45 | 1,546362752 | 1,336914019 | 3,885671323 | 0,048699789 | 0,109960935 |
| b0648 | 708 | 0,48 | 1,203340479 | 1,840967183 | 3,882904233 | 0,048780108 | 0,110086829 |
| b3817 | 381 | 0,35 | 1,33844528 | 1,63954647 | 3,879793077 | 0,04887058 | 0,110235501 |
| b1801 | 1446 | 0,51 | 0,726460981 | 2,732733156 | 3,876965088 | 0,048952972 | 0,110365805 |
| b3400 | 402 | 0,53 | 0,486787733 | 5,410542763 | 3,872990702 | 0,049069011 | 0,110571799 |
| b1694 | 1596 | 0,47 | 1,217932667 | 1,512156761 | 3,868398415 | 0,049203453 | 0,110819033 |
| b4221 | 3780 | 0,55 | -0,454343377 | 8,334968435 | 3,864367611 | 0,049321777 | 0,111029737 |
| b2259 | 267 | 0,47 | -0,607042894 | 5,439085079 | 3,858684012 | 0,049489131 | 0,111296711 |
| b3869 | 1050 | 0,55 | -0,531848184 | 4,885431997 | 3,858653293 | 0,049490037 | 0,111296711 |
| b1102 | 2190 | 0,5 | 0,681104693 | 3,722439245 | 3,856694139 | 0,049547864 | 0,111301045 |
| b0195 | 708 | 0,55 | 0,667533339 | 4,105864069 | 3,856583321 | 0,049551137 | 0,111301045 |
| b2233 | 3753 | 0,51 | -0,361599937 | 5,854824137 | 3,85574605 | 0,049575874 | 0,111301045 |
| b3466 | 270 | 0,46 | 0,860916327 | 3,150682777 | 3,855224296 | 0,049591296 | 0,111301045 |
| b0348 | 945 | 0,58 | 0,979086644 | 1,956254056 | 3,852192887 | 0,049680996 | 0,111441608 |
| b0805 | 2283 | 0,54 | 0,878614353 | 3,680437972 | 3,851428088 | 0,049703653 | 0,111441608 |
| b2683 | 336 | 0,54 | 0,668019166 | 4,326797108 | 3,845210095 | 0,049888271 | 0,111799616 |
| b0153 | 1983 | 0,6 | 0,820971532 | 4,129531778 | 3,828521535 | 0,05038736 | 0,112861643 |
| b3751 | 891 | 0,53 | -0,610890889 | 9,798330286 | 3,822970056 | 0,05055455 | 0,113179568 |
| b1633 | 636 | 0,49 | 0,769274834 | 4,053139139 | 3,820936856 | 0,050615929 | 0,113260407 |
| b4511 | 219 | 0,56 | 1,976465665 | 0,690381672 | 3,816649853 | 0,050745606 | 0,113493915 |
| b4178 | 426 | 0,48 | -0,436405183 | 6,305171396 | 3,809221589 | 0,050971133 | 0,113941456 |
| b2502 | 1542 | 0,51 | 0,35741147 | 7,243716535 | 3,806554504 | 0,051052366 | 0,114066154 |
| b2311 | 570 | 0,53 | 0,815778756 | 3,903082153 | 3,793245641 | 0,051459773 | 0,114919135 |
| b2425 | 1017 | 0,54 | 0,806179664 | 3,189227648 | 3,789723429 | 0,051568169 | 0,11510385 |
| b3826 | 801 | 0,52 | 0,424714597 | 5,743810834 | 3,784926233 | 0,05171619 | 0,115376786 |
| b3997 | 1065 | 0,56 | 0,358825954 | 7,203163773 | 3,781984275 | 0,051807189 | 0,115522298 |
| b4410 | 126 | 0,54 | 1,112441264 | 1,699063902 | 3,773346031 | 0,052075362 | 0,116062541 |
| b4271 | 1266 | 0,46 | -0,399178275 | 6,03173071 | 3,77060262 | 0,052160837 | 0,116195265 |
| b0827 | 1236 | 0,56 | -0,478873178 | 7,35121301 | 3,759248195 | 0,052516186 | 0,116873004 |
| b3222 | 876 | 0,58 | -0,453472674 | 5,829519828 | 3,759214923 | 0,052517231 | 0,116873004 |
| b0294 | 591 | 0,39 | 1,588055349 | 0,77205985 | 3,755875127 | 0,052622243 | 0,117048582 |
| b3865 | 633 | 0,52 | 0,344965367 | 6,71944735 | 3,754703641 | 0,05265913 | 0,11707253 |
| b0343 | 1254 | 0,46 | -0,671142662 | 3,31831189 | 3,748794911 | 0,052845598 | 0,117419775 |
| b3743 | 459 | 0,49 | 0,793843894 | 2,981330692 | 3,748095509 | 0,052867716 | 0,117419775 |
| b3872 | 711 | 0,49 | 0,810524973 | 3,361701367 | 3,735012687 | 0,053283262 | 0,118284091 |
| b0983 | 1140 | 0,51 | 0,688698726 | 3,434822701 | 3,731307978 | 0,053401561 | 0,118488018 |
| b2474 | 2016 | 0,55 | -0,388670749 | 6,615758905 | 3,728821388 | 0,053481119 | 0,118605826 |
| b2675 | 2145 | 0,52 | 0,546280748 | 4,926422996 | 3,727751125 | 0,0535154 | 0,118623157 |
| b1499 | 762 | 0,4 | 1,639216917 | 1,176420917 | 3,724152881 | 0,053630826 | 0,118820248 |
| b3620 | 1047 | 0,54 | 0,367921431 | 7,093736943 | 3,716972159 | 0,05386196 | 0,119260796 |

| | | | | | | | |
|---|---|---|---|---|---|---|---|
| b0499 | 708 | 0,45 | 0,973897775 | 2,17217486 | 3,716323547 | 0,05388289 | 0,119260796 |
| b2577 | 882 | 0,5 | -0,54483601 | 4,257510349 | 3,709920758 | 0,054089959 | 0,119660018 |
| b2450 | 810 | 0,5 | 0,562534042 | 6,665123481 | 3,702444747 | 0,054332804 | 0,120137952 |
| b3235 | 1068 | 0,53 | 0,451247928 | 5,481273416 | 3,698821041 | 0,054450929 | 0,120339776 |
| b0087 | 1083 | 0,53 | -0,42126728 | 7,279567158 | 3,693376174 | 0,054628933 | 0,12067367 |
| b3729 | 1830 | 0,54 | 0,426658911 | 9,996332625 | 3,692245749 | 0,054665966 | 0,12069599 |
| b2935 | 1992 | 0,56 | 0,604790393 | 10,18706215 | 3,689241245 | 0,054764524 | 0,120787745 |
| b3647 | 1683 | 0,53 | -0,530941877 | 4,029936559 | 3,688909869 | 0,054775405 | 0,120787745 |
| b1194 | 735 | 0,51 | 1,221765534 | 1,713170657 | 3,688515081 | 0,054788373 | 0,120787745 |
| b4386 | 1017 | 0,58 | -0,49643806 | 6,022946232 | 3,681974142 | 0,055003689 | 0,121202819 |
| b1719 | 1929 | 0,5 | -0,483502259 | 10,94909299 | 3,67596746 | 0,055202208 | 0,12158049 |
| b2871 | 1197 | 0,54 | -0,653561686 | 3,26158905 | 3,668281416 | 0,055457339 | 0,122082414 |
| b0628 | 966 | 0,53 | 0,448916825 | 7,599646738 | 3,666918478 | 0,055502711 | 0,122122313 |
| b3416 | 2085 | 0,55 | -0,443757778 | 8,836818993 | 3,66027734 | 0,055724357 | 0,122549836 |
| b3576 | 468 | 0,45 | 1,551640946 | 0,963617165 | 3,654246131 | 0,055926459 | 0,122933983 |
| b3686 | 429 | 0,49 | -1,955047998 | 5,150069585 | 3,650869441 | 0,05603995 | 0,123123067 |
| b1294 | 1644 | 0,54 | -0,422161097 | 6,928918414 | 3,649619099 | 0,056082036 | 0,123155162 |
| b1563 | 288 | 0,47 | -0,431518476 | 6,213724342 | 3,643792264 | 0,056278607 | 0,123526307 |
| b2741 | 993 | 0,52 | 0,56131361 | 12,03370605 | 3,641194992 | 0,056366463 | 0,1236557 |
| b4022 | 873 | 0,52 | -0,367973988 | 5,69893569 | 3,640419074 | 0,056392738 | 0,1236557 |
| b0230 | 756 | 0,53 | 0,902807644 | 2,573380906 | 3,637302269 | 0,056498412 | 0,123826838 |
| b3713 | 567 | 0,53 | -0,384982393 | 7,135364229 | 3,634198069 | 0,056603868 | 0,123997329 |
| b0647 | 555 | 0,43 | 1,3895917 | 0,971341199 | 3,633149392 | 0,05663954 | 0,124014861 |
| b2183 | 696 | 0,53 | 0,56627732 | 5,907454334 | 3,629019232 | 0,056780268 | 0,124262286 |
| b2744 | 762 | 0,55 | -0,323637635 | 7,372174947 | 3,626572791 | 0,056863801 | 0,124384361 |
| b1280 | 1170 | 0,52 | -0,388018967 | 6,528172017 | 3,623246065 | 0,0569776 | 0,12457249 |
| b1486 | 1023 | 0,55 | 1,131658482 | 3,913628104 | 3,604910927 | 0,057609155 | 0,125891873 |
| b4190 | 750 | 0,53 | -0,516855396 | 5,192460724 | 3,599908685 | 0,057782745 | 0,12620968 |
| b0326 | 816 | 0,39 | 1,772278017 | 0,908231328 | 3,598277899 | 0,057839457 | 0,126272015 |
| b0460 | 219 | 0,41 | -0,714838023 | 5,60745493 | 3,597440608 | 0,057868598 | 0,126274127 |
| b0152 | 891 | 0,55 | 0,94865589 | 3,295845307 | 3,595102412 | 0,057950058 | 0,12635765 |
| b0187 | 390 | 0,51 | -0,502677788 | 5,615908432 | 3,594723809 | 0,057963259 | 0,12635765 |
| b3065 | 216 | 0,52 | 0,790552973 | 7,219729254 | 3,570771261 | 0,058804993 | 0,128130276 |
| b3540 | 1005 | 0,56 | 0,548252124 | 8,763879188 | 3,569227185 | 0,058859698 | 0,128178944 |
| b0719 | 567 | 0,47 | 1,376876022 | 1,1797869 | 3,568526917 | 0,058884526 | 0,128178944 |
| b4013 | 930 | 0,5 | 1,049093799 | 4,588090309 | 3,556797159 | 0,059302059 | 0,129025159 |
| b2168 | 939 | 0,53 | 0,321788812 | 7,206996634 | 3,546985859 | 0,05965372 | 0,129727303 |
| b2327 | 810 | 0,53 | 0,481846186 | 4,76723161 | 3,524292662 | 0,060475625 | 0,131450897 |
| b0704 | 570 | 0,37 | 1,540162665 | 2,093878987 | 3,523322493 | 0,06051103 | 0,131464099 |
| b2216 | 2673 | 0,49 | 0,343044518 | 7,892911389 | 3,514727298 | 0,060825666 | 0,132083641 |
| b1617 | 1812 | 0,52 | -0,659677137 | 4,330346808 | 3,511904218 | 0,060929387 | 0,132244801 |
| b2489 | 759 | 0,56 | -0,732799528 | 2,98935926 | 3,5095399 | 0,061016398 | 0,132369554 |
| b0684 | 531 | 0,49 | 0,432288395 | 7,375200781 | 3,501088564 | 0,061328505 | 0,132982274 |
| b3900 | 447 | 0,5 | 1,740582368 | 0,868310776 | 3,499917636 | 0,061371881 | 0,133011979 |
| b1073 | 417 | 0,53 | 1,466742609 | 1,201780274 | 3,496855969 | 0,061485453 | 0,133193716 |
| b1041 | 456 | 0,42 | 1,217170252 | 1,347709083 | 3,495188652 | 0,061547395 | 0,133263491 |
| b1942 | 444 | 0,52 | 1,966897535 | 0,891939326 | 3,490230074 | 0,061732005 | 0,13359867 |
| b1012 | 1149 | 0,55 | 1,671943604 | 1,295343514 | 3,487805157 | 0,0618225 | 0,133729944 |
| b4243 | 387 | 0,53 | -0,484198273 | 9,048244357 | 3,484201131 | 0,061957259 | 0,133956795 |
| b3089 | 1245 | 0,55 | 0,431149225 | 6,807119574 | 3,482059137 | 0,062037499 | 0,134027217 |
| b2516 | 1014 | 0,55 | -0,488119922 | 8,296450252 | 3,481735364 | 0,062049637 | 0,134027217 |
| b3510 | 333 | 0,43 | -0,700879545 | 12,37645367 | 3,471084891 | 0,062450339 | 0,13479822 |
| b4087 | 1533 | 0,48 | 0,730476145 | 2,566439659 | 3,470650672 | 0,062466734 | 0,13479822 |
| b1155 | 414 | 0,42 | 1,520313932 | 1,330814219 | 3,467964083 | 0,062568275 | 0,134952362 |
| b3145 | 1092 | 0,48 | -0,601666037 | 4,034995113 | 3,467134161 | 0,062599678 | 0,134955149 |
| b3040 | 774 | 0,53 | -0,412716898 | 6,654482859 | 3,462823931 | 0,062763039 | 0,13524228 |
| b0569 | 2238 | 0,53 | -0,472623521 | 5,349861237 | 3,461644847 | 0,062807807 | 0,135273709 |
| b3541 | 984 | 0,57 | 0,572732854 | 8,657416715 | 3,459269513 | 0,062898097 | 0,135395007 |
| b4556 | 815 | 0,53 | 0,871509959 | 2,474581554 | 3,45857446 | 0,062924543 | 0,135395007 |
| b2254 | 969 | 0,5 | 0,751492026 | 4,800234026 | 3,450834839 | 0,06321983 | 0,135965105 |
| b0481 | 480 | 0,51 | 0,498062535 | 4,429971996 | 3,442870135 | 0,063525248 | 0,136491182 |
| b2734 | 657 | 0,42 | 1,219673029 | 1,712928537 | 3,442867561 | 0,063525347 | 0,136491182 |
| b2631 | 372 | 0,46 | -0,48628792 | 5,527073167 | 3,432134118 | 0,063939428 | 0,137315053 |
| b2998 | 288 | 0,45 | 1,180197139 | 1,534800938 | 3,429664636 | 0,064035103 | 0,137454661 |
| b3043 | 552 | 0,44 | 1,272032989 | 1,363575398 | 3,423789301 | 0,064263347 | 0,137828488 |
| b1851 | 1812 | 0,54 | -0,352774397 | 6,385398075 | 3,423598893 | 0,064270759 | 0,137828488 |
| b3823 | 621 | 0,52 | 0,587984784 | 5,181405114 | 3,422498731 | 0,064313599 | 0,1378544 |
| b3082 | 417 | 0,48 | 0,534800408 | 4,054860603 | 3,419833143 | 0,064417524 | 0,138011158 |
| b2437 | 1053 | 0,53 | -0,564829883 | 5,24074437 | 3,418088813 | 0,064485628 | 0,138091058 |
| b0263 | 348 | 0,56 | 1,362746109 | 1,457773336 | 3,414451056 | 0,064627905 | 0,138329642 |
| b2868 | 480 | 0,52 | -0,619046585 | 3,673033827 | 3,412314837 | 0,064711611 | 0,138442692 |
| b3441 | 489 | 0,52 | 0,537526186 | 4,017961301 | 3,398857262 | 0,065241602 | 0,139500453 |
| b1201 | 1920 | 0,51 | 0,45356401 | 5,107608129 | 3,398182843 | 0,065268284 | 0,139500453 |
| b3697 | 813 | 0,51 | 0,37267202 | 5,846604178 | 3,396104985 | 0,065350563 | 0,139609735 |
| b0286 | 690 | 0,55 | 0,705067693 | 3,843590364 | 3,390603283 | 0,065568954 | 0,140009554 |
| b4620 | 279 | 0,49 | 1,113195256 | 1,649833399 | 3,38855991 | 0,065650265 | 0,140116422 |
| b1249 | 1461 | 0,52 | 0,367681025 | 6,995068543 | 3,386721118 | 0,065723527 | 0,140206019 |
| b0880 | 225 | 0,48 | -0,522237685 | 10,19633214 | 3,383472392 | 0,065853177 | 0,140384111 |

| | | | | | | | |
|---|---|---|---|---|---|---|---|
| b1814 | 1365 | 0,54 | 0,42147166 | 8,729827468 | 3,383060022 | 0,065869653 | 0,140384111 |
| b2120 | 2280 | 0,56 | 0,910165781 | 2,423158173 | 3,381510783 | 0,065931593 | 0,140449334 |
| b4137 | 339 | 0,5 | 0,545612208 | 4,220065041 | 3,376854391 | 0,066118134 | 0,140779798 |
| b0959 | 630 | 0,48 | -0,393994703 | 5,373323023 | 3,368808696 | 0,066441784 | 0,141401745 |
| b4660 | 1607 | 0,35 | 1,179052864 | 1,593296981 | 3,365766593 | 0,066564597 | 0,141595883 |
| b3098 | 306 | 0,55 | -0,438247748 | 10,01517067 | 3,361706696 | 0,066728879 | 0,141878005 |
| b4186 | 1164 | 0,53 | 0,801245655 | 2,49271216 | 3,359783248 | 0,066806861 | 0,141976459 |
| b1847 | 357 | 0,49 | 0,48139496 | 8,567415429 | 3,357790291 | 0,066887764 | 0,142081023 |
| b1459 | 1054 | 0,38 | 1,423979506 | 2,001036601 | 3,352252898 | 0,067113102 | 0,142492148 |
| b1300 | 1488 | 0,57 | 0,705954167 | 7,048297319 | 3,347926606 | 0,06728972 | 0,142799492 |
| b3405 | 720 | 0,55 | -0,344281301 | 8,503762459 | 3,347016443 | 0,06732694 | 0,14281086 |
| b1091 | 954 | 0,55 | 0,393848409 | 8,360645834 | 3,340892614 | 0,06757794 | 0,143275462 |
| b2438 | 501 | 0,58 | -0,613716255 | 5,093919146 | 3,339348919 | 0,067641369 | 0,143312367 |
| b2498 | 627 | 0,53 | 0,507367579 | 6,756168045 | 3,338912897 | 0,067659297 | 0,143312367 |
| b3086 | 693 | 0,52 | 1,11207587 | 1,712213757 | 3,336943134 | 0,067740349 | 0,14341627 |
| b4692 | 234 | 0,54 | 1,387793933 | 1,331754408 | 3,333174846 | 0,067895696 | 0,143677295 |
| b2698 | 501 | 0,52 | 0,965664177 | 2,75524911 | 3,325575197 | 0,068210151 | 0,144274609 |
| b3330 | 378 | 0,48 | 1,160374871 | 1,189085766 | 3,322489256 | 0,068338284 | 0,144470401 |
| b0577 | 1248 | 0,48 | 0,42134498 | 5,575754963 | 3,321794075 | 0,068367184 | 0,144470401 |
| b4251 | 594 | 0,49 | 0,54046543 | 3,651891469 | 3,315687651 | 0,068621607 | 0,144898715 |
| b2968 | 537 | 0,53 | 0,952639735 | 2,057617514 | 3,315378072 | 0,068634532 | 0,144898715 |
| b0450 | 339 | 0,51 | 1,255614467 | 1,345462008 | 3,308698231 | 0,068914062 | 0,145395486 |
| b1139 | 894 | 0,37 | -0,660510231 | 4,494874674 | 3,308205774 | 0,068934718 | 0,145395486 |
| b2842 | 762 | 0,52 | -0,432473689 | 4,919711696 | 3,29504462 | 0,06948922 | 0,146496088 |
| b1548 | 528 | 0,53 | 1,368325252 | 1,083363538 | 3,291428484 | 0,069642409 | 0,146750012 |
| b2910 | 330 | 0,47 | -0,43327288 | 7,838713234 | 3,287196297 | 0,069822154 | 0,14705963 |
| b0638 | 612 | 0,51 | 0,5729118 | 3,92248418 | 3,285809107 | 0,069881177 | 0,147086415 |
| b3029 | 315 | 0,5 | -0,374585524 | 7,867649148 | 3,285355131 | 0,069900505 | 0,147086415 |
| b1890 | 888 | 0,51 | 1,10266969 | 1,683543054 | 3,279429654 | 0,070153304 | 0,147549089 |
| b0172 | 558 | 0,51 | 0,380261382 | 9,054468118 | 3,276966732 | 0,070258668 | 0,147701384 |
| b4030 | 411 | 0,49 | 1,158169733 | 2,676893099 | 3,274847615 | 0,07034946 | 0,147776301 |
| b3887 | 438 | 0,55 | -0,333524037 | 6,864835885 | 3,274596026 | 0,070360247 | 0,147776301 |
| b0003 | 933 | 0,56 | -0,440267139 | 8,244227085 | 3,270636072 | 0,070530272 | 0,148064019 |
| b3623 | 1074 | 0,31 | -0,441917192 | 5,33367 | 3,268058854 | 0,070641164 | 0,148227387 |
| b2904 | 390 | 0,53 | 0,412671548 | 10,13119397 | 3,259727827 | 0,07100091 | 0,148843279 |
| b3010 | 957 | 0,5 | -0,497844512 | 4,7850323 | 3,259723444 | 0,0710011 | 0,148843279 |
| b3888 | 990 | 0,55 | -0,324398182 | 7,605484774 | 3,253450458 | 0,071273273 | 0,149343996 |
| b0367 | 828 | 0,57 | 1,177345812 | 1,574892643 | 3,244457501 | 0,071665412 | 0,150095502 |
| b3675 | 363 | 0,5 | 0,775135192 | 3,135529757 | 3,241021409 | 0,071815853 | 0,150324472 |
| b3538 | 1680 | 0,53 | -0,36266319 | 6,64121229 | 3,239918287 | 0,071864222 | 0,150324472 |
| b2442 | 1209 | 0,54 | 0,521797546 | 4,823784823 | 3,239664498 | 0,071875355 | 0,150324472 |
| b4065 | 1650 | 0,56 | -0,329926743 | 6,77019617 | 3,229716158 | 0,072313221 | 0,15116971 |
| b3855 | 120 | 0,62 | -0,545269503 | 10,27783023 | 3,223370699 | 0,072594004 | 0,151685932 |
| b1152 | 780 | 0,58 | 1,066809965 | 1,576507453 | 3,22030863 | 0,072729916 | 0,151899107 |
| b2781 | 792 | 0,51 | -0,342030817 | 6,825760577 | 3,214810384 | 0,072974646 | 0,152339247 |
| b1661 | 1149 | 0,48 | 0,473336495 | 9,783024107 | 3,206561289 | 0,073343476 | 0,153037924 |
| b2625 | 1410 | 0,38 | 1,667305046 | 1,238026558 | 3,20446567 | 0,073437493 | 0,153162793 |
| b2769 | 861 | 0,56 | 0,724188644 | 2,607465218 | 3,191776686 | 0,074009535 | 0,154256801 |
| b2091 | 1041 | 0,49 | -0,616228764 | 11,64728036 | 3,191305365 | 0,074030875 | 0,154256801 |
| b2017 | 252 | 0,48 | 0,441189762 | 5,349437713 | 3,185332252 | 0,074301891 | 0,15474957 |
| b2566 | 906 | 0,52 | 0,386881399 | 7,322221182 | 3,182362777 | 0,07443702 | 0,154943043 |
| b2989 | 867 | 0,53 | -0,318945985 | 7,934675749 | 3,181772219 | 0,074463926 | 0,154943043 |
| b1001 | 1257 | 0,34 | 1,135738729 | 1,77072871 | 3,180193917 | 0,074535884 | 0,155020803 |
| b1424 | 1656 | 0,51 | 0,355680979 | 6,852649765 | 3,179421414 | 0,074571131 | 0,155022174 |
| b4157 | 315 | 0,5 | 0,826404362 | 1,855992845 | 3,174857415 | 0,074779739 | 0,155383768 |
| b2389 | 1257 | 0,54 | -0,416098004 | 5,616625048 | 3,172067818 | 0,074907552 | 0,155577223 |
| b4023 | 273 | 0,49 | -0,549052252 | 3,637608072 | 3,166419169 | 0,07516708 | 0,156033311 |
| b0600 | 1161 | 0,54 | 0,949057894 | 4,02555104 | 3,16577416 | 0,075196777 | 0,156033311 |
| b2379 | 1239 | 0,55 | -0,374432902 | 7,591272679 | 3,163102021 | 0,075319937 | 0,156216547 |
| b4308 | 1241 | 0,44 | 0,763969711 | 2,958766141 | 3,161026981 | 0,075415727 | 0,156342871 |
| b2626 | 627 | 0,4 | 1,191975919 | 1,361869615 | 3,158798727 | 0,075518735 | 0,156484035 |
| b0539 | 279 | 0,5 | 1,873492997 | 0,948764816 | 3,155669953 | 0,075663627 | 0,15671182 |
| b2470 | 3114 | 0,54 | 0,472604344 | 4,956742359 | 3,151017762 | 0,075879622 | 0,157086588 |
| b3609 | 468 | 0,51 | 0,386014343 | 9,161758111 | 3,147982894 | 0,076020883 | 0,157275437 |
| b1857 | 933 | 0,49 | -0,414235176 | 6,186525555 | 3,147550662 | 0,076041025 | 0,157275437 |
| b0085 | 1488 | 0,57 | -0,363487785 | 8,332498377 | 3,143401649 | 0,076234658 | 0,1576032 |
| b2991 | 342 | 0,55 | -0,779134036 | 3,742606562 | 3,140995131 | 0,076347213 | 0,15776312 |
| b3290 | 1377 | 0,5 | 0,337841021 | 7,247211376 | 3,13955426 | 0,076414689 | 0,157829785 |
| b2134 | 933 | 0,51 | 0,414670239 | 5,155376471 | 3,137817971 | 0,076496084 | 0,157925127 |
| b1074 | 405 | 0,53 | 1,382775052 | 1,173728093 | 3,136814116 | 0,076543187 | 0,157949614 |
| b0622 | 561 | 0,47 | -0,747739257 | 4,339319822 | 3,134158389 | 0,076667948 | 0,158134257 |
| b3198 | 567 | 0,53 | 0,357464711 | 6,550565817 | 3,13315264 | 0,076715253 | 0,158135494 |
| b3279 | 555 | 0,49 | 0,311613623 | 6,437890985 | 3,132645633 | 0,076739112 | 0,158135494 |
| b1214 | 810 | 0,46 | 0,35671697 | 6,29293686 | 3,119872915 | 0,077342815 | 0,159306295 |
| b4580 | 4168 | 0,45 | -0,409839205 | 6,033013925 | 3,116440476 | 0,077505919 | 0,159568916 |
| b2882 | 1401 | 0,49 | -0,515840083 | 3,911491889 | 3,113350198 | 0,077653081 | 0,15979849 |
| b1959 | 921 | 0,54 | 0,911530281 | 2,465619288 | 3,11173769 | 0,07772999 | 0,159860043 |
| b0841 | 597 | 0,52 | 0,666682349 | 3,921444437 | 3,111227781 | 0,077754327 | 0,159860043 |

| | | | | | | | |
|---|---|---|---|---|---|---|---|
| b3391 | 1239 | 0,54 | 0,664751181 | 3,113061501 | 3,110213552 | 0,077802759 | 0,159886274 |
| b2035 | 1167 | 0,32 | -0,470064401 | 6,869571551 | 3,108696432 | 0,077875265 | 0,159961934 |
| b4261 | 1101 | 0,55 | -0,353311664 | 7,646036686 | 3,102914057 | 0,078152286 | 0,16045742 |
| b2065 | 582 | 0,6 | -0,316696378 | 7,26357772 | 3,095535328 | 0,078507326 | 0,161112563 |
| b0858 | 489 | 0,52 | 0,728458084 | 3,575591851 | 3,093456918 | 0,078607646 | 0,161244608 |
| b0932 | 2613 | 0,53 | 0,473783429 | 9,946408958 | 3,089252727 | 0,078810993 | 0,161587773 |
| b2961 | 1053 | 0,55 | 0,394840121 | 5,569872019 | 3,082954513 | 0,079116685 | 0,162140367 |
| b3187 | 972 | 0,53 | 0,374550316 | 7,643423545 | 3,080463941 | 0,07923792 | 0,162274095 |
| b2538 | 1362 | 0,53 | 0,475479844 | 4,801537204 | 3,079126614 | 0,079303101 | 0,162274095 |
| b0660 | 1041 | 0,54 | -0,437928489 | 8,709418159 | 3,078673793 | 0,079325184 | 0,162274095 |
| b4518 | 174 | 0,51 | 0,608782152 | 7,861811402 | 3,078641465 | 0,079326761 | 0,162274095 |
| b1546 | 576 | 0,48 | 1,953187442 | 0,734949631 | 3,070121641 | 0,079743505 | 0,163052184 |
| b1963 | 366 | 0,5 | 1,032957333 | 3,02956863 | 3,066380991 | 0,079927222 | 0,163345192 |
| b1653 | 4617 | 0,57 | -0,381455589 | 6,6193901 | 3,065720781 | 0,079959694 | 0,163345192 |
| b3556 | 213 | 0,48 | 0,734133853 | 4,10759347 | 3,063583974 | 0,080064892 | 0,163485578 |
| b2690 | 567 | 0,53 | -0,34631031 | 6,034400663 | 3,058587946 | 0,080311434 | 0,163844614 |
| b2592 | 2574 | 0,51 | -0,886344661 | 10,70226429 | 3,058539327 | 0,080313837 | 0,163844614 |
| b0498 | 369 | 0,31 | 1,089631551 | 1,739585229 | 3,054401759 | 0,080518642 | 0,164187695 |
| b2094 | 453 | 0,43 | 0,613201295 | 10,98514974 | 3,049586101 | 0,080757721 | 0,164600322 |
| b3708 | 1416 | 0,51 | 0,943101033 | 12,34398307 | 3,027615467 | 0,081858238 | 0,166767555 |
| b3071 | 624 | 0,54 | -0,54509671 | 5,065473995 | 3,021905684 | 0,082146884 | 0,16727957 |
| b1722 | 759 | 0,49 | 0,920797044 | 3,1631141 | 3,019352366 | 0,082276317 | 0,167467054 |
| b4441 | 184 | 0,5 | 0,612679846 | 8,820956132 | 3,016737604 | 0,082409093 | 0,167661168 |
| b3712 | 750 | 0,52 | -0,420155169 | 5,540747598 | 3,012962048 | 0,082601221 | 0,167975805 |
| b3415 | 1317 | 0,53 | -0,46595446 | 5,605198199 | 3,011436331 | 0,082678998 | 0,168057719 |
| b2993 | 495 | 0,55 | -0,671099358 | 6,091408535 | 3,009722328 | 0,082766468 | 0,168159252 |
| b4396 | 870 | 0,53 | -0,421788064 | 9,140436806 | 3,004423194 | 0,083037528 | 0,168633531 |
| b4532 | 177 | 0,49 | 0,790542399 | 2,874670216 | 2,999455676 | 0,083292496 | 0,169048234 |
| b3899 | 1452 | 0,56 | 0,99602111 | 1,957849068 | 2,998975866 | 0,083317168 | 0,169048234 |
| b4056 | 417 | 0,52 | -0,479867874 | 8,185492591 | 2,995918756 | 0,083474552 | 0,169290923 |
| b2390 | 327 | 0,54 | 0,574063883 | 3,973216866 | 2,993851517 | 0,083581157 | 0,169430459 |
| b4508 | 312 | 0,51 | 1,610519966 | 0,706820788 | 2,989002704 | 0,083831783 | 0,169815453 |
| b3706 | 1365 | 0,56 | 0,347934441 | 6,725191348 | 2,98871164 | 0,083846853 | 0,169815453 |
| b2688 | 1557 | 0,51 | 0,366748976 | 8,86109326 | 2,985124635 | 0,084032818 | 0,170115216 |
| b1907 | 1212 | 0,51 | 0,63011652 | 4,106629579 | 2,982164247 | 0,084186631 | 0,170349653 |
| b4183 | 660 | 0,47 | 1,220135113 | 1,290781287 | 2,974057064 | 0,084609419 | 0,17094959 |
| b0760 | 1473 | 0,53 | 0,417081778 | 5,35574069 | 2,973964205 | 0,084614274 | 0,17094959 |
| b4177 | 1299 | 0,54 | -0,489558838 | 11,01229932 | 2,973706297 | 0,084627763 | 0,17094959 |
| b4472 | 3801 | 0,53 | -0,340891541 | 7,468111957 | 2,973554837 | 0,084635685 | 0,17094959 |
| b4461 | 1287 | 0,51 | 0,363858946 | 5,69713292 | 2,962087067 | 0,085237852 | 0,172088313 |
| b2979 | 1500 | 0,57 | 1,908869272 | 9,188328915 | 2,944990723 | 0,086144194 | 0,173839836 |
| b2471 | 357 | 0,47 | -0,453706855 | 5,272461149 | 2,936585621 | 0,086593599 | 0,174668096 |
| b2070 | 1947 | 0,51 | -0,522650454 | 4,338395606 | 2,934271372 | 0,086717782 | 0,174839901 |
| b1251 | 297 | 0,49 | 0,410006209 | 6,246134434 | 2,932586418 | 0,086808319 | 0,174943743 |
| b0813 | 888 | 0,53 | 0,838773811 | 3,071506611 | 2,92834916 | 0,08703645 | 0,175324659 |
| b1390 | 747 | 0,53 | 1,278645939 | 5,100303421 | 2,926546051 | 0,087133724 | 0,175441758 |
| b2264 | 1671 | 0,58 | -0,346215395 | 7,081517572 | 2,921502058 | 0,087406465 | 0,17591189 |
| b0590 | 1005 | 0,61 | 0,807784055 | 2,132689783 | 2,918980711 | 0,087543147 | 0,176107893 |
| b2542 | 1203 | 0,54 | 0,617916246 | 4,134264275 | 2,916773504 | 0,08766299 | 0,176269861 |
| b4058 | 2823 | 0,56 | 0,352101686 | 8,431951206 | 2,908597923 | 0,088108442 | 0,177086115 |
| b3610 | 252 | 0,52 | 0,306973597 | 7,066249244 | 2,906831112 | 0,088205029 | 0,177140248 |
| b0977 | 858 | 0,55 | -0,678455685 | 3,969766726 | 2,90665944 | 0,088214421 | 0,177140248 |
| b1826 | 144 | 0,47 | 0,685350949 | 3,044479062 | 2,903498433 | 0,088387532 | 0,177341035 |
| b4300 | 783 | 0,5 | -0,493536918 | 4,52392198 | 2,903388746 | 0,088393546 | 0,177341035 |
| b1914 | 657 | 0,5 | -0,309973941 | 7,483848419 | 2,900257188 | 0,088565421 | 0,177606361 |
| b0637 | 318 | 0,51 | 0,542776249 | 5,084763931 | 2,893769887 | 0,088922628 | 0,178242943 |
| b3099 | 405 | 0,53 | -0,430889934 | 10,31095681 | 2,882224643 | 0,089562209 | 0,17939411 |
| b2959 | 327 | 0,49 | 0,431337832 | 8,186429761 | 2,88195909 | 0,089576979 | 0,17939411 |
| b2823 | 324 | 0,55 | 0,781454077 | 2,701084642 | 2,881051748 | 0,089627464 | 0,179415048 |
| b4320 | 903 | 0,5 | 0,454352485 | 4,587379577 | 2,877103031 | 0,089847531 | 0,179775283 |
| b1176 | 696 | 0,51 | 0,340553081 | 7,334756735 | 2,864682117 | 0,090543597 | 0,181087193 |
| b3795 | 1386 | 0,53 | 0,464589136 | 5,432056495 | 2,863586352 | 0,090605283 | 0,181129741 |
| b3667 | 1320 | 0,55 | 0,62063436 | 3,325828746 | 2,854029893 | 0,091145208 | 0,182127874 |
| b1662 | 642 | 0,48 | 0,36412691 | 6,829639831 | 2,852490497 | 0,091232508 | 0,182221078 |
| b3582 | 861 | 0,56 | -0,70148203 | 2,697958337 | 2,851309399 | 0,09129955 | 0,182273756 |
| b1465 | 681 | 0,53 | -0,56455942 | 6,507238405 | 2,850063666 | 0,091370318 | 0,182333823 |
| b3690 | 1065 | 0,51 | 0,808057918 | 2,123847413 | 2,847130055 | 0,091537209 | 0,182585568 |
| b1973 | 651 | 0,38 | -0,736209922 | 2,329264262 | 2,845375868 | 0,091637161 | 0,182634062 |
| b3893 | 903 | 0,55 | -0,579147981 | 10,01346792 | 2,844773387 | 0,091671518 | 0,182634062 |
| b0182 | 1149 | 0,54 | 0,346043379 | 7,216697793 | 2,844272913 | 0,091700068 | 0,182634062 |
| b4067 | 1650 | 0,56 | 1,010049853 | 8,814363544 | 2,843844508 | 0,091724514 | 0,182634062 |
| b1310 | 1293 | 0,5 | 0,831014753 | 1,785444408 | 2,842450125 | 0,091804132 | 0,182711421 |
| b0763 | 774 | 0,51 | -0,401222783 | 5,733870365 | 2,840219645 | 0,091931647 | 0,182883995 |
| b0707 | 510 | 0,5 | -0,436764708 | 5,586471923 | 2,835086744 | 0,092225821 | 0,18331445 |
| b1496 | 1686 | 0,44 | 1,231425565 | 1,468450155 | 2,835016965 | 0,092229827 | 0,18331445 |
| b1573 | 219 | 0,48 | 1,063635984 | 1,758046693 | 2,831841425 | 0,092412343 | 0,183595798 |
| b2572 | 651 | 0,52 | -0,431628742 | 9,06257174 | 2,828564694 | 0,092601086 | 0,183886088 |
| b1799 | 924 | 0,52 | -0,47745781 | 6,559214084 | 2,8278811 | 0,092640515 | 0,183886088 |

| | | | | | | | |
|---|---|---|---|---|---|---|---|
| b2230 | 1689 | 0,51 | -0,498016517 | 3,485105519 | 2,825548513 | 0,092775192 | 0,184071894 |
| b3331 | 588 | 0,5 | 0,95727684 | 1,898250968 | 2,824736807 | 0,092822107 | 0,184083489 |
| b1147 | 558 | 0,5 | 0,554282419 | 3,351562767 | 2,822403446 | 0,092957116 | 0,184269701 |
| b3449 | 744 | 0,56 | 0,406911469 | 6,501222632 | 2,817972011 | 0,093214108 | 0,18469745 |
| b2472 | 1128 | 0,53 | -0,376020257 | 6,974730547 | 2,811298308 | 0,093602595 | 0,185385254 |
| b0879 | 1947 | 0,54 | -0,339526239 | 6,484799355 | 2,81048317 | 0,093650166 | 0,185397546 |
| b0691 | 651 | 0,44 | 1,637819375 | 0,867732076 | 2,80682731 | 0,093863843 | 0,185738519 |
| b2622 | 1242 | 0,48 | 0,356688821 | 6,441139493 | 2,805338755 | 0,093950998 | 0,185804363 |
| b0323 | 951 | 0,56 | 0,728954732 | 2,28214386 | 2,804843258 | 0,093980029 | 0,185804363 |
| b4671 | 102 | 0,28 | -1,33809994 | 1,009207618 | 2,802667656 | 0,094107612 | 0,185974566 |
| b1375 | 234 | 0,36 | 1,773679031 | 0,632250521 | 2,800592471 | 0,094229481 | 0,186074836 |
| b4033 | 1545 | 0,53 | -0,912033031 | 8,216047271 | 2,80038997 | 0,094241383 | 0,186074836 |
| b1547 | 963 | 0,53 | 1,061976827 | 2,126908264 | 2,796537358 | 0,094468122 | 0,186440389 |
| b2245 | 804 | 0,54 | 0,669015702 | 3,327213927 | 2,785524883 | 0,095119524 | 0,187643357 |
| b3242 | 204 | 0,48 | 0,687700149 | 2,50470342 | 2,784248264 | 0,095195353 | 0,187710327 |
| b3549 | 564 | 0,5 | 0,42308562 | 5,118858079 | 2,776770069 | 0,095640872 | 0,188505887 |
| b3896 | 1056 | 0,46 | 0,970042847 | 2,002647051 | 2,770088423 | 0,096040856 | 0,18921104 |
| b3958 | 1005 | 0,54 | -0,527092844 | 4,17823652 | 2,765276344 | 0,096330051 | 0,189657737 |
| b0613 | 879 | 0,55 | 1,12625918 | 1,747391671 | 2,764908035 | 0,096352224 | 0,189657737 |
| b1043 | 333 | 0,43 | 1,856774466 | 0,762358857 | 2,763353175 | 0,096445894 | 0,189758778 |
| b4397 | 474 | 0,49 | 0,428702 | 5,271957831 | 2,761951913 | 0,096530395 | 0,189841699 |
| b2933 | 1389 | 0,5 | 1,023143188 | 1,895492448 | 2,760576262 | 0,096613431 | 0,189911391 |
| b4641 | 159 | 0,4 | 0,923161904 | 2,346096234 | 2,759961284 | 0,096650576 | 0,189911391 |
| b4032 | 891 | 0,55 | -0,858777986 | 7,648073741 | 2,757576737 | 0,096794754 | 0,190111345 |
| b1577 | 765 | 0,49 | 1,110569917 | 1,661378309 | 2,753631542 | 0,09703381 | 0,190459791 |
| b1071 | 294 | 0,53 | 0,977144427 | 2,095633666 | 2,753246855 | 0,097057154 | 0,190459791 |
| b1624 | 1041 | 0,53 | 0,305828346 | 6,930248059 | 2,751299856 | 0,097175398 | 0,190608375 |
| b2196 | 1944 | 0,57 | -0,766992866 | 6,063304367 | 2,748120814 | 0,097368805 | 0,190857661 |
| b0571 | 684 | 0,54 | 0,480147475 | 4,767028092 | 2,747811339 | 0,097387655 | 0,190857661 |
| b2275 | 849 | 0,43 | 0,656183288 | 3,496811627 | 2,73481899 | 0,098182625 | 0,192331524 |
| b1345 | 1236 | 0,43 | -0,624763069 | 3,87554026 | 2,728124806 | 0,098594987 | 0,193054929 |
| b0010 | 567 | 0,53 | -0,393947105 | 5,335041043 | 2,724715179 | 0,098805746 | 0,193383124 |
| b2715 | 1458 | 0,56 | -0,505856563 | 4,222841648 | 2,720246201 | 0,099082731 | 0,193746885 |
| b0826 | 750 | 0,55 | -0,41105027 | 6,162142797 | 2,720054204 | 0,09909465 | 0,193746885 |
| b0279 | 855 | 0,36 | 1,39932217 | 1,246242471 | 2,719625215 | 0,099121287 | 0,193746885 |
| b0832 | 912 | 0,53 | -0,416199354 | 4,36185407 | 2,718451194 | 0,099194224 | 0,19380493 |
| b4224 | 252 | 0,5 | -0,55967488 | 4,773156277 | 2,714650255 | 0,099430762 | 0,194182428 |
| b2532 | 741 | 0,55 | -0,49469023 | 7,210432934 | 2,712209749 | 0,099582962 | 0,194394964 |
| b2265 | 1296 | 0,53 | 0,39159755 | 6,885332588 | 2,707734451 | 0,099862723 | 0,194856215 |
| b4384 | 720 | 0,52 | -0,378292217 | 9,902935653 | 2,691919293 | 0,100858262 | 0,196713111 |
| b3550 | 441 | 0,52 | 0,546521652 | 3,665531013 | 2,686341563 | 0,101211953 | 0,197317083 |
| b0862 | 717 | 0,55 | -0,295718929 | 7,68512025 | 2,681048189 | 0,101548868 | 0,197887837 |
| b4398 | 690 | 0,55 | 0,404192519 | 5,431608574 | 2,669570188 | 0,102283643 | 0,199233067 |
| b1815 | 1599 | 0,51 | -0,423195852 | 5,143128065 | 2,667023921 | 0,102447432 | 0,199407444 |
| b0009 | 588 | 0,54 | -0,374025178 | 6,900772553 | 2,666716842 | 0,102467204 | 0,199407444 |
| b3284 | 474 | 0,46 | -0,383054626 | 8,917687699 | 2,66610459 | 0,102506638 | 0,199407444 |
| b1821 | 567 | 0,52 | 0,985821023 | 2,687704476 | 2,662707425 | 0,102725746 | 0,199746982 |
| b2556 | 1428 | 0,53 | -0,3365263 | 5,767900297 | 2,660238108 | 0,102885332 | 0,199970537 |
| b3898 | 1071 | 0,53 | 0,58538841 | 3,032912092 | 2,6590464 | 0,102962446 | 0,200033673 |
| b1753 | 549 | 0,56 | -0,645431652 | 4,266902001 | 2,657656506 | 0,103052465 | 0,200080348 |
| b0443 | 399 | 0,46 | 0,496529374 | 4,469785992 | 2,657297153 | 0,103075753 | 0,200080348 |
| b1246 | 1014 | 0,52 | 0,619728287 | 9,999212738 | 2,656229248 | 0,103144993 | 0,200119134 |
| b2155 | 1992 | 0,53 | 0,758356051 | 4,227802133 | 2,655612049 | 0,103185033 | 0,200119134 |
| b1706 | 1437 | 0,53 | 0,320017024 | 6,708257537 | 2,654841452 | 0,10323505 | 0,200129538 |
| b0751 | 720 | 0,48 | -0,373794683 | 5,947936084 | 2,651971114 | 0,103421585 | 0,200404472 |
| b3392 | 405 | 0,52 | 1,088039067 | 1,238520741 | 2,649646252 | 0,103572942 | 0,200611031 |
| b1978 | 7077 | 0,5 | -0,454746616 | 6,197936287 | 2,63546628 | 0,104501377 | 0,202321889 |
| b1112 | 258 | 0,47 | 1,094426133 | 2,383715657 | 2,631173872 | 0,104784217 | 0,202781892 |
| b2158 | 1050 | 0,53 | -0,437167316 | 4,993902137 | 2,62873415 | 0,104945352 | 0,203006072 |
| b1421 | 1641 | 0,51 | 0,524283182 | 3,930405882 | 2,622585376 | 0,105352662 | 0,203706053 |
| b1429 | 993 | 0,53 | -0,425527121 | 5,26365105 | 2,618545812 | 0,105621196 | 0,204137214 |
| b1975 | 90 | 0,63 | -0,730520872 | 2,647470529 | 2,617584107 | 0,105685236 | 0,204172944 |
| b3265 | 1158 | 0,52 | 0,914418183 | 2,016109256 | 2,61048402 | 0,106159356 | 0,205000532 |
| b1338 | 1311 | 0,55 | 0,594174978 | 3,097107832 | 2,605726726 | 0,106478337 | 0,205527953 |
| b2173 | 987 | 0,55 | 0,54722766 | 4,705973897 | 2,60302808 | 0,106659751 | 0,205789499 |
| b4185 | 639 | 0,49 | 1,419686899 | 1,146261342 | 2,595982005 | 0,107135018 | 0,206516893 |
| b2469 | 1701 | 0,52 | -0,443394154 | 5,431865755 | 2,595812197 | 0,1071465 | 0,206516893 |
| b0686 | 765 | 0,51 | 0,418297893 | 5,204656041 | 2,595391002 | 0,107174987 | 0,206516893 |
| b0899 | 1431 | 0,47 | 0,558820533 | 4,650734981 | 2,589561635 | 0,107570104 | 0,207024056 |
| b4542 | 108 | 0,49 | 0,974694341 | 2,173109851 | 2,588591106 | 0,107636042 | 0,207024056 |
| b1023 | 2019 | 0,44 | 0,750844687 | 3,492153192 | 2,588009401 | 0,107675584 | 0,207024056 |
| b1305 | 225 | 0,48 | 0,514395668 | 3,837810532 | 2,587730174 | 0,107694571 | 0,207024056 |
| b0997 | 2547 | 0,55 | -0,446616033 | 3,950533934 | 2,587147808 | 0,107734182 | 0,207024056 |
| b0595 | 858 | 0,55 | 1,157388209 | 3,147531728 | 2,586905205 | 0,107750688 | 0,207024056 |
| b1618 | 591 | 0,51 | -0,385904901 | 6,141583858 | 2,586746048 | 0,107761518 | 0,207024056 |
| b3635 | 810 | 0,54 | 0,650314877 | 4,25252992 | 2,582036416 | 0,108082532 | 0,207551804 |
| b2123 | 462 | 0,42 | 1,040812374 | 1,762057176 | 2,581229662 | 0,108137627 | 0,20756867 |
| b2794 | 849 | 0,49 | 0,31603876 | 5,878445223 | 2,579053162 | 0,108286418 | 0,207765293 |

| | | | | | | | |
|---|---|---|---|---|---|---|---|
| b3678 | 1494 | 0,52 | -0,516613759 | 3,079785573 | 2,576771222 | 0,108442658 | 0,207976036 |
| b0234 | 453 | 0,43 | 0,82192127 | 2,252052792 | 2,575246332 | 0,108547203 | 0,208087495 |
| b2052 | 966 | 0,56 | 0,852897593 | 2,232708677 | 2,574216188 | 0,108617891 | 0,208094678 |
| b3659 | 1185 | 0,46 | 1,136842302 | 1,475765806 | 2,573838683 | 0,108643808 | 0,208094678 |
| b0857 | 846 | 0,55 | 0,530965583 | 3,693779607 | 2,562649068 | 0,109415104 | 0,209482485 |
| b2290 | 1218 | 0,5 | 0,31718182 | 6,065114666 | 2,560954919 | 0,109532405 | 0,209617523 |
| b0013 | 405 | 0,49 | 0,957526717 | 1,719680841 | 2,5581416 | 0,109727501 | 0,209901264 |
| b4062 | 324 | 0,51 | 0,569174322 | 3,962875815 | 2,556871657 | 0,109815693 | 0,209980349 |
| b4222 | 342 | 0,51 | -0,349815803 | 6,27855383 | 2,555507018 | 0,109910549 | 0,210072102 |
| b3915 | 903 | 0,52 | -0,276680932 | 6,771122675 | 2,543291123 | 0,110763694 | 0,21161248 |
| b3602 | 363 | 0,51 | 0,423752619 | 4,944160577 | 2,533818807 | 0,111430246 | 0,212784317 |
| b1591 | 615 | 0,53 | 0,523939151 | 4,234160088 | 2,533227287 | 0,111472016 | 0,212784317 |
| b2427 | 858 | 0,52 | -0,568680402 | 3,915370627 | 2,530462593 | 0,111667475 | 0,213066676 |
| b3172 | 1344 | 0,53 | 0,404492049 | 7,872174673 | 2,528975182 | 0,111772788 | 0,213176867 |
| b2172 | 1467 | 0,52 | 0,349105606 | 4,662790645 | 2,524159395 | 0,112114512 | 0,213731556 |
| b4156 | 1503 | 0,47 | 0,551015609 | 4,298571237 | 2,523511166 | 0,112160597 | 0,213731556 |
| b3525 | 768 | 0,53 | 1,081818401 | 1,562724689 | 2,522863237 | 0,112206682 | 0,213731556 |
| b1690 | 1215 | 0,45 | 1,102798893 | 1,606862189 | 2,522078492 | 0,112262527 | 0,213747088 |
| b4650 | 252 | 0,44 | 1,773559508 | 0,809543651 | 2,518726709 | 0,112501392 | 0,214110929 |
| b1049 | 2544 | 0,55 | 0,334554552 | 8,21566362 | 2,514811637 | 0,112781109 | 0,214552177 |
| b4676 | 99 | 0,42 | 1,094301377 | 1,344444834 | 2,510583754 | 0,113084035 | 0,215016411 |
| b2719 | 768 | 0,56 | 0,539314137 | 3,026249463 | 2,510067536 | 0,113121084 | 0,215016411 |
| b0344 | 3075 | 0,56 | -0,446337017 | 4,878541339 | 2,508913685 | 0,113203942 | 0,215082692 |
| b4401 | 717 | 0,51 | 0,455484372 | 10,34626147 | 2,501500677 | 0,113737873 | 0,216005099 |
| b2479 | 573 | 0,5 | 0,327001258 | 6,274847822 | 2,500836881 | 0,113785818 | 0,216005099 |
| b0020 | 906 | 0,47 | 0,304279023 | 6,048717944 | 2,499474543 | 0,11388429 | 0,216100502 |
| b2441 | 1362 | 0,56 | -0,465029436 | 8,054262651 | 2,498572899 | 0,113949513 | 0,216132763 |
| b3406 | 477 | 0,5 | -0,357789459 | 4,895351573 | 2,492815479 | 0,114366968 | 0,216832805 |
| b0573 | 333 | 0,45 | 1,173095825 | 1,612163784 | 2,490516936 | 0,1145341 | 0,217057859 |
| b1431 | 669 | 0,45 | 0,339065188 | 7,663311466 | 2,478299843 | 0,115426965 | 0,218613022 |
| b0568 | 2973 | 0,55 | -0,332938239 | 6,303238971 | 2,477810055 | 0,11546292 | 0,218613022 |
| b2296 | 1203 | 0,52 | -0,407440233 | 8,866679324 | 2,477291033 | 0,115501034 | 0,218613022 |
| b3063 | 1464 | 0,55 | 0,95664964 | 3,006580846 | 2,474410614 | 0,115712811 | 0,21892141 |
| b2053 | 1122 | 0,55 | 0,884260475 | 2,46714775 | 2,468799145 | 0,116126616 | 0,2196116 |
| b0611 | 807 | 0,51 | -0,311545566 | 6,421730604 | 2,458950046 | 0,116856871 | 0,220813656 |
| b1555 | 312 | 0,44 | 1,328211584 | 1,090278152 | 2,458897585 | 0,116860775 | 0,220813656 |
| b3133 | 474 | 0,51 | -0,648580562 | 3,520911998 | 2,45611532 | 0,117067989 | 0,22111198 |
| b4286 | 330 | 0,54 | 0,713304367 | 2,380017988 | 2,454529562 | 0,117186273 | 0,221242155 |
| b3578 | 1278 | 0,51 | 1,141978565 | 1,510497143 | 2,452673706 | 0,117324872 | 0,221410559 |
| b3801 | 1656 | 0,53 | -0,417078688 | 4,487231879 | 2,444681027 | 0,117923854 | 0,22244727 |
| b0713 | 735 | 0,58 | -0,43006216 | 6,730485113 | 2,442071934 | 0,118120114 | 0,222723749 |
| b1048 | 1536 | 0,5 | 0,3543512 | 8,363445884 | 2,428957432 | 0,119112094 | 0,224499751 |
| b3064 | 1014 | 0,58 | 0,294518267 | 6,121239794 | 2,422497866 | 0,119604081 | 0,225205465 |
| b0154 | 1281 | 0,56 | 0,277182711 | 7,679183408 | 2,422137364 | 0,119631604 | 0,225205465 |
| b3090 | 552 | 0,5 | 0,785993968 | 2,697097639 | 2,422063258 | 0,119637263 | 0,225205465 |
| b4108 | 336 | 0,46 | 0,38197719 | 4,833925457 | 2,418884394 | 0,119880277 | 0,225568179 |
| b0059 | 2907 | 0,55 | 0,354867084 | 7,714487508 | 2,411525714 | 0,120444926 | 0,226535526 |
| b3977 | 85 | 0,59 | 1,312910169 | 1,239571988 | 2,40372168 | 0,121046968 | 0,227572362 |
| b4464 | 1368 | 0,53 | -0,416506811 | 4,769386229 | 2,397353835 | 0,121540686 | 0,22840476 |
| b3721 | 1413 | 0,49 | 0,792567656 | 1,908295807 | 2,393553266 | 0,121836418 | 0,228864554 |
| b2581 | 1038 | 0,56 | 0,31846519 | 7,140069375 | 2,392370322 | 0,121928629 | 0,228941816 |
| b2072 | 762 | 0,52 | 0,964399717 | 1,426485484 | 2,390372218 | 0,122084557 | 0,229138603 |
| b3664 | 1335 | 0,48 | -0,634682321 | 2,693454022 | 2,389061129 | 0,122186992 | 0,229234868 |
| b0672 | 85 | 0,6 | 1,729577278 | 0,680029614 | 2,387321636 | 0,122323045 | 0,229394096 |
| b0024 | 219 | 0,49 | -0,703411387 | 2,690928714 | 2,384560331 | 0,122539364 | 0,229703651 |
| b0587 | 1134 | 0,45 | 1,713654365 | 1,04891031 | 2,383186677 | 0,122647133 | 0,229809553 |
| b0145 | 456 | 0,53 | 0,321652183 | 9,559413376 | 2,382292368 | 0,122717352 | 0,229845037 |
| b0508 | 777 | 0,45 | 1,419655555 | 3,443364985 | 2,375929417 | 0,123218244 | 0,230686789 |
| b1956 | 1695 | 0,51 | -0,270584655 | 6,958550438 | 2,373792012 | 0,12338701 | 0,230906296 |
| b3517 | 1401 | 0,54 | -0,8305618 | 11,22477758 | 2,372541647 | 0,123485855 | 0,230949665 |
| b0236 | 501 | 0,54 | 1,159195127 | 1,382432691 | 2,372195418 | 0,123513241 | 0,230949665 |
| b2371 | 1146 | 0,45 | -0,624984887 | 3,65382269 | 2,369886931 | 0,12369601 | 0,231194962 |
| b0196 | 405 | 0,53 | 0,470166249 | 5,100233056 | 2,367806364 | 0,123860992 | 0,231406822 |
| b2062 | 1140 | 0,54 | 1,199634175 | 1,335648124 | 2,360256233 | 0,124461744 | 0,232344534 |
| b3627 | 1020 | 0,36 | -0,338268825 | 5,788052574 | 2,359855355 | 0,124493732 | 0,232344534 |
| b1119 | 912 | 0,53 | -0,282728572 | 7,036471654 | 2,35925166 | 0,12454192 | 0,232344534 |
| b4439 | 93 | 0,35 | 1,632435769 | 0,601003774 | 2,358896722 | 0,124570262 | 0,232344534 |
| b0077 | 1725 | 0,53 | 0,41541225 | 4,493002373 | 2,358082456 | 0,124635308 | 0,232369156 |
| b2059 | 840 | 0,52 | 0,914161071 | 1,854533186 | 2,356332181 | 0,124775253 | 0,23253334 |
| b3178 | 1935 | 0,54 | 0,366987739 | 10,40493022 | 2,346646913 | 0,125552809 | 0,233727744 |
| b1087 | 585 | 0,5 | 0,29885357 | 6,977457266 | 2,346454565 | 0,125568305 | 0,233727744 |
| b3364 | 1182 | 0,52 | 0,490839895 | 3,568856349 | 2,34578118 | 0,125622574 | 0,233727744 |
| b4697 | 143 | 0,42 | -0,895539367 | 2,279421784 | 2,345754167 | 0,125624751 | 0,233727744 |
| b2944 | 498 | 0,55 | 0,785951655 | 2,669689545 | 2,339247934 | 0,126150445 | 0,23460842 |
| b0869 | 1431 | 0,55 | 0,307416876 | 6,330688966 | 2,336480314 | 0,126374805 | 0,234928194 |
| b1642 | 435 | 0,48 | 0,288374159 | 6,947862111 | 2,327155304 | 0,127134021 | 0,236241576 |
| b4084 | 930 | 0,55 | -0,440022201 | 4,128272888 | 2,320392621 | 0,127687793 | 0,237172271 |
| b3264 | 663 | 0,44 | 1,321407963 | 1,538045895 | 2,318243931 | 0,127864304 | 0,237401744 |

| | | | | | | | |
|---|---|---|---|---|---|---|---|
| b0061 | 696 | 0,55 | -0,670614736 | 3,101552543 | 2,316603847 | 0,127999216 | 0,237553824 |
| b4383 | 1224 | 0,54 | -0,405596558 | 10,4051098 | 2,313049579 | 0,128292132 | 0,23799446 |
| b0608 | 1239 | 0,5 | 0,416494912 | 5,61982732 | 2,312435194 | 0,12834284 | 0,23799446 |
| b4231 | 996 | 0,52 | -0,614918683 | 5,381611235 | 2,310982912 | 0,128462794 | 0,23811838 |
| b4369 | 87 | 0,6 | 1,421134455 | 1,261694515 | 2,301150763 | 0,129278188 | 0,239530731 |
| b1745 | 1344 | 0,56 | 0,717125085 | 7,332819027 | 2,299761927 | 0,12939383 | 0,239645929 |
| b4661 | 2646 | 0,52 | -0,45128005 | 5,016866831 | 2,294541165 | 0,129829572 | 0,240353632 |
| b3068 | 507 | 0,54 | -0,283248395 | 7,167234063 | 2,291209438 | 0,130108504 | 0,240770567 |
| b1171 | 330 | 0,45 | 0,542659949 | 3,737178859 | 2,288610537 | 0,130326547 | 0,240993877 |
| b4096 | 681 | 0,6 | -0,583591757 | 3,796431036 | 2,288489464 | 0,130336715 | 0,240993877 |
| b1145 | 675 | 0,44 | 0,382509437 | 5,826609131 | 2,286621371 | 0,13049371 | 0,241184664 |
| b4101 | 453 | 0,63 | 1,276855439 | 0,956470812 | 2,280584182 | 0,131002522 | 0,242025269 |
| b1433 | 1176 | 0,53 | -0,453508394 | 3,735003029 | 2,278010761 | 0,131220081 | 0,242256604 |
| b0303 | 237 | 0,35 | 1,411359553 | 0,897706758 | 2,277824541 | 0,13123584 | 0,242256604 |
| b2482 | 2019 | 0,55 | 0,702460323 | 2,706946132 | 2,276018477 | 0,131388787 | 0,242439088 |
| b1754 | 1167 | 0,56 | -0,628836521 | 5,032968783 | 2,263015436 | 0,132495846 | 0,244381227 |
| b3017 | 1413 | 0,56 | -0,266212258 | 7,158176671 | 2,258502872 | 0,13288247 | 0,244993514 |
| b0358 | 774 | 0,5 | -0,543721972 | 3,531035987 | 2,257521797 | 0,132966693 | 0,245047992 |
| b3966 | 1845 | 0,5 | 0,3448833 | 6,883461719 | 2,255741438 | 0,133119684 | 0,245135678 |
| b1896 | 1425 | 0,49 | -0,407677148 | 9,207017544 | 2,255695206 | 0,133123659 | 0,245135678 |
| b2832 | 714 | 0,5 | 0,522988558 | 4,229446908 | 2,254012234 | 0,133268467 | 0,245301548 |
| b0874 | 900 | 0,48 | 0,686193534 | 2,713076919 | 2,252663671 | 0,133384629 | 0,245414576 |
| b0112 | 1374 | 0,53 | -0,355210529 | 5,786348992 | 2,251196299 | 0,133511153 | 0,245501877 |
| b2745 | 1050 | 0,52 | 0,271845151 | 7,829736128 | 2,250843092 | 0,133541628 | 0,245501877 |
| b1952 | 189 | 0,51 | -0,42262246 | 5,002561798 | 2,243599593 | 0,134168326 | 0,246538459 |
| b0425 | 912 | 0,52 | -0,332895037 | 6,482792661 | 2,243055952 | 0,134215493 | 0,246538459 |
| b2873 | 1386 | 0,53 | 0,527663346 | 3,031812954 | 2,239439817 | 0,13452971 | 0,246936226 |
| b3134 | 375 | 0,58 | -0,607034277 | 2,646280356 | 2,239295956 | 0,134542227 | 0,246936226 |
| b2874 | 933 | 0,49 | -0,534456401 | 3,85433139 | 2,235280392 | 0,134892152 | 0,247477129 |
| b1058 | 141 | 0,38 | 1,671905113 | 0,651800074 | 2,227205444 | 0,135598907 | 0,248671973 |
| b3825 | 1023 | 0,54 | -0,290627359 | 6,757209208 | 2,217326359 | 0,136469211 | 0,250165646 |
| b2099 | 1005 | 0,56 | 0,440592482 | 4,09521378 | 2,212104984 | 0,136931717 | 0,250910856 |
| b2420 | 735 | 0,39 | 0,505745254 | 3,863108624 | 2,194624064 | 0,138492994 | 0,253668002 |
| b0247 | 477 | 0,58 | 0,747684672 | 2,052265847 | 2,193499598 | 0,138594105 | 0,253749501 |
| b1845 | 2061 | 0,5 | -0,275273674 | 7,264226075 | 2,191364189 | 0,138786347 | 0,253997716 |
| b1179 | 294 | 0,44 | 0,458238954 | 4,678361193 | 2,190184241 | 0,138892701 | 0,254088606 |
| b3882 | 897 | 0,55 | -0,491652036 | 2,815334554 | 2,185694793 | 0,139298191 | 0,254726435 |
| b2043 | 1395 | 0,47 | 0,474604638 | 3,731016392 | 2,18374721 | 0,139474511 | 0,254944845 |
| b4246 | 135 | 0,5 | 1,648337281 | 0,884366313 | 2,1817952 | 0,139651484 | 0,255164268 |
| b0296 | 264 | 0,46 | 0,947870521 | 1,356611412 | 2,1794153 | 0,13986759 | 0,255454987 |
| b0904 | 858 | 0,49 | -0,384483602 | 8,007002096 | 2,177323313 | 0,140057863 | 0,255698306 |
| b1410 | 1758 | 0,51 | 0,583246725 | 3,461617483 | 2,175879364 | 0,140189364 | 0,255834173 |
| b1957 | 183 | 0,53 | 0,346585713 | 7,564791905 | 2,174925342 | 0,140276323 | 0,255888678 |
| b2063 | 1584 | 0,53 | -0,353009944 | 5,96356021 | 2,169016761 | 0,140816243 | 0,256677611 |
| b2835 | 1194 | 0,56 | -0,314898671 | 5,988679536 | 2,168765883 | 0,140839219 | 0,256677611 |
| b3221 | 465 | 0,47 | -0,34487358 | 5,357927127 | 2,168313989 | 0,140880617 | 0,256677611 |
| b0076 | 945 | 0,46 | 0,600200432 | 3,234622594 | 2,165043135 | 0,141180661 | 0,257050334 |
| b1615 | 1266 | 0,47 | 0,948650837 | 1,972907065 | 2,164833748 | 0,141199893 | 0,257050334 |
| b1836 | 237 | 0,49 | 0,402556777 | 7,412506928 | 2,16303911 | 0,141364851 | 0,257246147 |
| b1357 | 297 | 0,43 | 0,968792939 | 2,101600313 | 2,161423125 | 0,141513572 | 0,257412269 |
| b4651 | 365 | 0,36 | 1,636191406 | 0,886264261 | 2,145413649 | 0,14299649 | 0,260004166 |
| b1365 | 264 | 0,48 | 1,628401767 | 0,890028206 | 2,140176107 | 0,143485419 | 0,260787367 |
| b4327 | 912 | 0,51 | -0,357826487 | 4,681890546 | 2,138113398 | 0,143678491 | 0,261009658 |
| b4417 | 79 | 0,46 | 1,773792344 | 0,443993366 | 2,13762558 | 0,143724194 | 0,261009658 |
| b3624 | 852 | 0,33 | -0,344721726 | 4,816221797 | 2,132776023 | 0,144179433 | 0,261730344 |
| b2300 | 555 | 0,52 | 0,32587131 | 6,684653911 | 2,130938583 | 0,144352342 | 0,261866119 |
| b4325 | 831 | 0,4 | 1,284573829 | 0,834143942 | 2,130739615 | 0,14437108 | 0,261866119 |
| b1699 | 1290 | 0,55 | 0,558852856 | 2,826527619 | 2,129964907 | 0,144444063 | 0,261892512 |
| b0355 | 834 | 0,49 | 0,407506385 | 4,602490648 | 2,124473628 | 0,144962574 | 0,2626528 |
| b2760 | 1509 | 0,44 | 0,613897117 | 2,882962142 | 2,124283172 | 0,144980595 | 0,2626528 |
| b1414 | 801 | 0,51 | 0,337385037 | 6,195118773 | 2,121531143 | 0,145241279 | 0,263000419 |
| b2671 | 345 | 0,47 | -0,47703393 | 4,311460718 | 2,121019164 | 0,145289834 | 0,263000419 |
| b1078 | 783 | 0,54 | 0,832911585 | 2,392087657 | 2,11918657 | 0,145463784 | 0,263208994 |
| b2826 | 471 | 0,54 | 0,770801752 | 1,996512968 | 2,117074149 | 0,145664586 | 0,26346597 |
| b3995 | 477 | 0,49 | 0,379239937 | 6,694445735 | 2,111585561 | 0,146187781 | 0,264305621 |
| b3146 | 861 | 0,55 | 0,427728394 | 4,871048335 | 2,107930499 | 0,146537372 | 0,264830848 |
| b2456 | 288 | 0,53 | 0,872146455 | 1,328836562 | 2,106571826 | 0,146667564 | 0,264959299 |
| b2180 | 1590 | 0,51 | 0,319860712 | 6,063072034 | 2,099846504 | 0,147313927 | 0,265912358 |
| b2097 | 1053 | 0,51 | -0,373908629 | 10,54212423 | 2,099698947 | 0,147328144 | 0,265912358 |
| b2645 | 318 | 0,54 | 0,948667257 | 1,382113995 | 2,099232333 | 0,147373114 | 0,265912358 |
| b1816 | 1557 | 0,54 | 0,311645274 | 6,796424665 | 2,0939089 | 0,147887256 | 0,266732668 |
| b2637 | 468 | 0,51 | -0,491421162 | 3,879426114 | 2,092905378 | 0,147984403 | 0,266800521 |
| b3270 | 1104 | 0,52 | 0,643345529 | 2,587674476 | 2,090203161 | 0,148246355 | 0,267165325 |
| b1398 | 1314 | 0,52 | 0,636606323 | 6,65159522 | 2,087198941 | 0,148538197 | 0,267583681 |
| b1432 | 1149 | 0,52 | -0,337191213 | 5,883312047 | 2,086412428 | 0,148614709 | 0,267613952 |
| b2267 | 462 | 0,48 | 0,310770007 | 5,457850392 | 2,083338654 | 0,148914154 | 0,267958758 |
| b4488 | 1645 | 0,55 | 0,369934914 | 6,307192423 | 2,08312942 | 0,148934562 | 0,267958758 |
| b2025 | 777 | 0,52 | 0,555035519 | 5,528625796 | 2,082606837 | 0,148985548 | 0,267958758 |

| | | | | | | | |
|---|---|---|---|---|---|---|---|
| b2118 | 3633 | 0,51 | -0,396275334 | 4,192869763 | 2,080996752 | 0,149142758 | 0,268133912 |
| b1257 | 507 | 0,41 | -0,669698835 | 5,875504933 | 2,075274865 | 0,149702969 | 0,268993462 |
| b2281 | 543 | 0,54 | 0,631795098 | 8,847788552 | 2,074493643 | 0,14977964 | 0,268993462 |
| b3673 | 1185 | 0,57 | 0,348016535 | 5,866715696 | 2,074276992 | 0,149800911 | 0,268993462 |
| b1676 | 1413 | 0,5 | -0,387499111 | 10,12461437 | 2,069599341 | 0,150260992 | 0,269711559 |
| b2565 | 729 | 0,54 | 0,34647401 | 5,004699687 | 2,068056097 | 0,150413131 | 0,269876563 |
| b1594 | 1221 | 0,52 | -0,372144908 | 7,278057456 | 2,06614146 | 0,150602126 | 0,270038957 |
| b1366 | 453 | 0,49 | 1,051683552 | 1,577572992 | 2,065918633 | 0,150624139 | 0,270038957 |
| b1514 | 1029 | 0,54 | -0,584928363 | 6,237058958 | 2,061945293 | 0,151017274 | 0,270635514 |
| b0558 | 411 | 0,32 | -1,007770832 | 1,78338217 | 2,05549866 | 0,151657597 | 0,271598298 |
| b2068 | 849 | 0,56 | -0,385960059 | 4,783507093 | 2,05531673 | 0,151675712 | 0,271598298 |
| b0630 | 642 | 0,48 | 0,465415844 | 4,82597873 | 2,046145595 | 0,152592078 | 0,273130069 |
| b3473 | 1218 | 0,57 | 0,417870316 | 4,427088027 | 2,044913468 | 0,152715668 | 0,273158736 |
| b4184 | 399 | 0,44 | 1,486995889 | 0,860536901 | 2,044770805 | 0,152729985 | 0,273158736 |
| b0139 | 2598 | 0,43 | 0,687249303 | 2,623226064 | 2,039437349 | 0,153266328 | 0,273918454 |
| b3463 | 669 | 0,56 | 0,25767669 | 6,789456089 | 2,039331512 | 0,153276993 | 0,273918454 |
| b0459 | 552 | 0,48 | 0,485365498 | 5,480693849 | 2,035228637 | 0,153691072 | 0,274459048 |
| b2067 | 3318 | 0,52 | 0,324479199 | 6,604753622 | 2,035120866 | 0,153701966 | 0,274459048 |
| b3498 | 2043 | 0,55 | 0,331708268 | 8,840076487 | 2,033713107 | 0,153844346 | 0,274603887 |
| b4586 | 480 | 0,31 | 1,508519905 | 0,571074127 | 2,025245683 | 0,154703902 | 0,27602822 |
| b2006 | 168 | 0,49 | 1,389214547 | 1,112195891 | 2,019865047 | 0,155252939 | 0,276897602 |
| b3699 | 2415 | 0,55 | 0,290407581 | 8,954618385 | 2,017822523 | 0,155461936 | 0,277029658 |
| b2663 | 1401 | 0,51 | 0,55065852 | 8,04252483 | 2,017663146 | 0,155478257 | 0,277029658 |
| b1101 | 1434 | 0,53 | 0,363821746 | 9,784524133 | 2,017329712 | 0,155512409 | 0,277029658 |
| b4355 | 1656 | 0,55 | -0,449299051 | 4,354305604 | 2,013756449 | 0,155878939 | 0,277572271 |
| b2762 | 735 | 0,51 | 0,566384704 | 3,21811147 | 2,013094988 | 0,155946897 | 0,277582999 |
| b1923 | 1497 | 0,47 | -0,32541073 | 6,25305358 | 2,011691659 | 0,156091184 | 0,27772953 |
| b3511 | 573 | 0,47 | -0,584599538 | 9,851899267 | 2,010997135 | 0,156162649 | 0,277746426 |
| b2443 | 642 | 0,44 | -0,455379817 | 3,908630343 | 2,007281763 | 0,156545587 | 0,278317066 |
| b0837 | 1116 | 0,52 | 0,339826354 | 5,870144171 | 2,005056653 | 0,156775437 | 0,27861519 |
| b4571 | 1994 | 0,35 | -0,474993173 | 6,806000962 | 1,998766018 | 0,157427325 | 0,279662811 |
| b2162 | 942 | 0,52 | -0,762086553 | 1,8904813 | 1,997978639 | 0,157509137 | 0,279697286 |
| b3410 | 237 | 0,55 | 0,579614893 | 3,545213188 | 1,992488841 | 0,158080892 | 0,28060141 |
| b4482 | 765 | 0,48 | 0,568759396 | 2,396249397 | 1,988226999 | 0,158526386 | 0,281280784 |
| b0941 | 1071 | 0,48 | -0,437305945 | 3,647030214 | 1,987535056 | 0,15859885 | 0,281297999 |
| b2903 | 2874 | 0,56 | 0,396432413 | 11,76111308 | 1,986426544 | 0,158715018 | 0,281392686 |
| b1794 | 1026 | 0,48 | 0,39112163 | 5,448786347 | 1,982519141 | 0,159125272 | 0,281921623 |
| b1608 | 720 | 0,49 | 0,486499786 | 4,66725717 | 1,982387087 | 0,159139158 | 0,281921623 |
| b2742 | 1140 | 0,52 | 0,380656778 | 11,40925614 | 1,98140673 | 0,159242289 | 0,281992864 |
| b1504 | 531 | 0,45 | 1,69381342 | 0,727073523 | 1,980050246 | 0,159385114 | 0,282134312 |
| b4471 | 1365 | 0,53 | -0,744096754 | 4,069271061 | 1,97386215 | 0,160038513 | 0,283179081 |
| b2953 | 291 | 0,48 | 0,453509204 | 3,62940222 | 1,972977231 | 0,1601322 | 0,283233039 |
| b3206 | 273 | 0,49 | -0,40869478 | 4,38077151 | 1,970110997 | 0,160436081 | 0,283658585 |
| b3628 | 1080 | 0,4 | 0,292632443 | 6,025759335 | 1,969156266 | 0,160537448 | 0,283725884 |
| b2696 | 186 | 0,49 | -0,328307822 | 8,622825811 | 1,967761458 | 0,160685671 | 0,283814298 |
| b4213 | 1944 | 0,49 | -0,403346976 | 7,713274314 | 1,967493911 | 0,160714121 | 0,283814298 |
| b1711 | 981 | 0,55 | -0,39088188 | 5,013572225 | 1,962600317 | 0,161235492 | 0,284622873 |
| b0452 | 861 | 0,52 | 0,381222993 | 6,06093585 | 1,960856488 | 0,161421748 | 0,284752103 |
| b1077 | 756 | 0,59 | 0,649000707 | 2,069097286 | 1,96072536 | 0,161435764 | 0,284752103 |
| b2457 | 294 | 0,58 | -0,498285041 | 3,711997002 | 1,958442377 | 0,161680001 | 0,285070718 |
| b3457 | 927 | 0,56 | 0,705501107 | 1,767095734 | 1,957625977 | 0,161767444 | 0,285112734 |
| b3057 | 822 | 0,52 | 0,320759911 | 5,266040314 | 1,950732482 | 0,162507941 | 0,286305263 |
| b3433 | 1104 | 0,54 | 0,314895957 | 8,989812884 | 1,949711619 | 0,16261793 | 0,286386468 |
| b3067 | 1842 | 0,53 | 0,354036831 | 9,697933313 | 1,947362701 | 0,162871329 | 0,286720069 |
| b0278 | 699 | 0,37 | 0,759400144 | 1,792694425 | 1,945196337 | 0,163105433 | 0,287019454 |
| b1644 | 858 | 0,5 | 0,666209261 | 3,219009925 | 1,932825328 | 0,164449671 | 0,28927136 |
| b3886 | 873 | 0,51 | -0,257400737 | 7,229436019 | 1,931085374 | 0,164639748 | 0,289492096 |
| b0494 | 627 | 0,53 | 0,280347342 | 5,81321847 | 1,930133656 | 0,164743823 | 0,289515334 |
| b2187 | 228 | 0,5 | 0,434909545 | 4,485530398 | 1,929783304 | 0,164782154 | 0,289515334 |
| b2992 | 489 | 0,54 | -0,472184914 | 5,528944485 | 1,929138221 | 0,164852759 | 0,289525887 |
| b4024 | 1350 | 0,54 | -0,377729116 | 4,725087542 | 1,926426808 | 0,165149901 | 0,289934138 |
| b0559 | 207 | 0,4 | -0,991729476 | 2,452819496 | 1,922633402 | 0,165566646 | 0,290551961 |
| b0141 | 585 | 0,45 | 1,667019118 | 0,747050641 | 1,921446195 | 0,16569732 | 0,290667472 |
| b0793 | 1134 | 0,55 | -0,346997557 | 5,26586742 | 1,916402703 | 0,166253767 | 0,291529493 |
| b0047 | 1863 | 0,56 | 0,282837822 | 6,491751858 | 1,913832636 | 0,166538144 | 0,291839348 |
| b0950 | 1254 | 0,52 | -0,286713026 | 5,721849037 | 1,913079737 | 0,166621557 | 0,291839348 |
| b1833 | 1284 | 0,5 | -0,283649762 | 5,65654074 | 1,912960077 | 0,166634819 | 0,291839348 |
| b2837 | 1032 | 0,53 | -0,364625123 | 5,601544051 | 1,912453936 | 0,166690926 | 0,291839348 |
| b4028 | 738 | 0,59 | -1,092294523 | 1,09751133 | 1,908996247 | 0,1670748 | 0,292324214 |
| b1360 | 747 | 0,53 | 0,688777165 | 2,602869484 | 1,908784757 | 0,167098312 | 0,292324214 |
| b1464 | 894 | 0,52 | 0,306238061 | 5,729296233 | 1,908074001 | 0,167177359 | 0,292348389 |
| b3382 | 363 | 0,52 | 0,517381144 | 3,162096862 | 1,906830581 | 0,167315748 | 0,292476281 |
| b4106 | 789 | 0,59 | 1,166249453 | 0,994659239 | 1,9053459 | 0,167481161 | 0,292651292 |
| b2585 | 1356 | 0,49 | -0,3443482 | 8,467003392 | 1,903494004 | 0,16768775 | 0,292898088 |
| b4111 | 1503 | 0,5 | -0,319848674 | 9,145122512 | 1,902417169 | 0,16780801 | 0,292993963 |
| b4378 | 780 | 0,56 | 0,320046613 | 6,60496173 | 1,901309922 | 0,16793177 | 0,29309587 |
| b2564 | 732 | 0,56 | -0,264193263 | 6,874118708 | 1,900723579 | 0,16799735 | 0,293096194 |
| b0779 | 2022 | 0,54 | 0,262312067 | 8,344130346 | 1,896035979 | 0,168522691 | 0,293898327 |

| | | | | | | | |
|---|---|---|---|---|---|---|---|
| b3179 | 630 | 0,5 | -0,325825402 | 8,627966086 | 1,885368144 | 0,169725275 | 0,295880468 |
| b1671 | 669 | 0,5 | -0,602851733 | 3,211398326 | 1,880750375 | 0,170248887 | 0,296677882 |
| b3258 | 1452 | 0,54 | 0,301616434 | 4,895063948 | 1,877740596 | 0,170591166 | 0,297158805 |
| b3228 | 498 | 0,55 | 0,279206493 | 7,378129707 | 1,876939814 | 0,170682366 | 0,297202161 |
| b0650 | 1671 | 0,5 | -0,432923594 | 3,92553662 | 1,871199965 | 0,171337709 | 0,298227423 |
| b1075 | 696 | 0,55 | 0,708021555 | 1,97343146 | 1,869451917 | 0,171537865 | 0,298459904 |
| b2615 | 879 | 0,5 | -0,263702681 | 6,100236253 | 1,864232686 | 0,172137081 | 0,2992771 |
| b4582 | 1840 | 0,46 | -0,363874684 | 4,179174862 | 1,864197844 | 0,17214109 | 0,2992771 |
| b0646 | 1428 | 0,47 | 0,651315603 | 2,268894264 | 1,860286109 | 0,172591783 | 0,299944308 |
| b4100 | 585 | 0,61 | 1,100531522 | 1,0959482 | 1,857434793 | 0,172921154 | 0,300328054 |
| b2854 | 503 | 0,34 | 1,520070221 | 0,561126135 | 1,857214682 | 0,17294661 | 0,300328054 |
| b1130 | 672 | 0,48 | -0,266846322 | 7,325709188 | 1,855755771 | 0,173115445 | 0,300448491 |
| b2056 | 1218 | 0,42 | 0,891478333 | 1,518282893 | 1,855015817 | 0,17320115 | 0,300448491 |
| b1361 | 500 | 0,48 | 0,650879544 | 2,497957945 | 1,85487843 | 0,173217069 | 0,300448491 |
| b3805 | 942 | 0,56 | -0,24494422 | 7,833990871 | 1,852935605 | 0,173442352 | 0,300722871 |
| b1370 | 981 | 0,55 | -0,476006616 | 4,320965083 | 1,849310188 | 0,173863645 | 0,301336758 |
| b1954 | 87 | 0,44 | 1,639955915 | 0,868265292 | 1,846663419 | 0,174171958 | 0,301754431 |
| b2252 | 603 | 0,43 | 1,048539153 | 1,835047348 | 1,84497683 | 0,174368751 | 0,301978648 |
| b0235 | 207 | 0,51 | 1,509243584 | 0,561069634 | 1,831685911 | 0,175928544 | 0,304562276 |
| b2574 | 1623 | 0,53 | 0,467178733 | 3,296852038 | 1,829078139 | 0,17623647 | 0,304977551 |
| b3874 | 1266 | 0,45 | 0,917288199 | 3,436603384 | 1,826420198 | 0,17655096 | 0,30540386 |
| b0086 | 1359 | 0,55 | -0,299045958 | 8,017211865 | 1,822883208 | 0,176970464 | 0,306011427 |
| b1503 | 504 | 0,45 | 1,340790215 | 0,988106488 | 1,821830631 | 0,177095526 | 0,306109583 |
| b1093 | 735 | 0,52 | 0,29175332 | 9,940594572 | 1,817812555 | 0,177573875 | 0,306818083 |
| b2970 | 849 | 0,51 | 0,810759872 | 1,582846819 | 1,816198028 | 0,177766503 | 0,30703255 |
| b2251 | 426 | 0,48 | 0,527961942 | 3,364865259 | 1,813687252 | 0,178066542 | 0,307432296 |
| b1525 | 1389 | 0,53 | -0,360421758 | 5,51485864 | 1,810825452 | 0,178409241 | 0,307905359 |
| b0560 | 546 | 0,53 | 1,052883251 | 1,334627424 | 1,808745582 | 0,178658782 | 0,308133708 |
| b4082 | 1032 | 0,56 | 0,737109618 | 1,904652571 | 1,808058019 | 0,178741364 | 0,308133708 |
| b3671 | 1689 | 0,56 | 0,392589388 | 7,239046014 | 1,80800445 | 0,1787478 | 0,308133708 |
| b0570 | 1443 | 0,52 | 0,365602944 | 4,578236373 | 1,807140803 | 0,178851598 | 0,308194104 |
| b3353 | 1023 | 0,54 | 0,283172897 | 5,860707769 | 1,802833344 | 0,179370334 | 0,308969193 |
| b0726 | 2802 | 0,56 | 0,573693102 | 12,25930049 | 1,798719923 | 0,179867327 | 0,309656933 |
| b0392 | 342 | 0,47 | 0,677890243 | 2,930667401 | 1,798385717 | 0,179907776 | 0,309656933 |
| b2323 | 1221 | 0,57 | -0,395345608 | 10,215598 | 1,797167382 | 0,180055322 | 0,309791921 |
| b2026 | 612 | 0,55 | 0,37281919 | 5,639983426 | 1,792831615 | 0,180581539 | 0,310484737 |
| b2127 | 732 | 0,53 | -0,292076709 | 6,685792553 | 1,79224125 | 0,180653327 | 0,310484737 |
| b0815 | 1584 | 0,48 | 0,428493692 | 3,977569718 | 1,792138564 | 0,180665817 | 0,310484737 |
| b1053 | 1227 | 0,52 | 0,449751558 | 4,004285773 | 1,791366741 | 0,180759728 | 0,31052706 |
| b0272 | 759 | 0,63 | 0,41571912 | 4,718899433 | 1,789646481 | 0,180969242 | 0,31076787 |
| b0901 | 591 | 0,43 | 0,580307818 | 2,615473877 | 1,787667457 | 0,181210619 | 0,310992947 |
| b0354 | 540 | 0,48 | 0,308728943 | 5,581824458 | 1,787079673 | 0,181282381 | 0,310992947 |
| b1115 | 1074 | 0,5 | 0,638790409 | 2,173205012 | 1,786309217 | 0,181376496 | 0,310992947 |
| b3046 | 3805 | 0,42 | 0,649596503 | 2,933672633 | 1,786298061 | 0,181377859 | 0,310992947 |
| b3368 | 1374 | 0,55 | -0,294701543 | 6,05286083 | 1,785353198 | 0,181493356 | 0,311071978 |
| b1391 | 498 | 0,56 | 1,006204492 | 4,36332828 | 1,782718056 | 0,181815919 | 0,311403565 |
| b2218 | 2850 | 0,51 | 0,275040488 | 6,69480728 | 1,782637611 | 0,181825776 | 0,311403565 |
| b4021 | 690 | 0,54 | -0,361947258 | 6,12423201 | 1,78072557 | 0,182060256 | 0,31154511 |
| b4501 | 201 | 0,44 | 0,654186041 | 2,296134933 | 1,780458608 | 0,182093022 | 0,31154511 |
| b1713 | 2388 | 0,54 | 0,298624159 | 10,19940956 | 1,780263658 | 0,182116954 | 0,31154511 |
| b4403 | 687 | 0,52 | 0,362409218 | 4,661629705 | 1,77808714 | 0,18238439 | 0,31188357 |
| b0987 | 306 | 0,58 | 0,652598221 | 2,196718738 | 1,776546853 | 0,182573925 | 0,311975324 |
| b1373 | 576 | 0,48 | 1,037104844 | 1,105249358 | 1,77600954 | 0,182640096 | 0,311975324 |
| b4219 | 639 | 0,54 | -0,331194458 | 7,542223189 | 1,77595459 | 0,182646865 | 0,311975324 |
| b4274 | 336 | 0,46 | -0,4621865 | 5,872726342 | 1,774228579 | 0,182859624 | 0,312219746 |
| b1484 | 987 | 0,54 | 0,834157075 | 4,355865334 | 1,764758205 | 0,184032125 | 0,31410205 |
| b1550 | 174 | 0,3 | 0,56519184 | 4,895427669 | 1,763847883 | 0,184145288 | 0,314111998 |
| b2359 | 363 | 0,5 | 0,920653672 | 1,338378334 | 1,763551556 | 0,184182142 | 0,314111998 |
| b1762 | 1041 | 0,41 | 0,580112219 | 2,108372319 | 1,762699517 | 0,184288158 | 0,314111998 |
| b1837 | 192 | 0,46 | -0,560117623 | 4,404431809 | 1,76245749 | 0,184318285 | 0,314111998 |
| b4496 | 1193 | 0,46 | -0,46469025 | 6,150842627 | 1,761860927 | 0,18439257 | 0,314119155 |
| b1545 | 591 | 0,45 | 1,016025022 | 1,715555633 | 1,761170517 | 0,184478583 | 0,31414628 |
| b1084 | 3186 | 0,56 | 0,320417713 | 9,689308636 | 1,755708346 | 0,18516072 | 0,315173329 |
| b2900 | 312 | 0,44 | -0,325136313 | 5,007918107 | 1,755216041 | 0,185222345 | 0,315173329 |
| b3894 | 3051 | 0,55 | -0,457306319 | 11,91365862 | 1,75379505 | 0,185400352 | 0,315313121 |
| b3378 | 354 | 0,47 | 0,703955194 | 2,320368363 | 1,753437291 | 0,1854452 | 0,315313121 |
| b2987 | 1500 | 0,52 | 0,743766809 | 1,923246617 | 1,750732031 | 0,185784732 | 0,315676028 |
| b4241 | 948 | 0,54 | -0,339813777 | 4,761071742 | 1,750148116 | 0,185858113 | 0,315676028 |
| b4445 | 82 | 0,48 | 1,595453693 | 0,454834111 | 1,750054089 | 0,185869933 | 0,315676028 |
| b4117 | 2268 | 0,53 | -0,377004777 | 4,458148132 | 1,749067335 | 0,185994024 | 0,315732865 |
| b1788 | 105 | 0,43 | 1,116653316 | 1,026051021 | 1,748667867 | 0,186044288 | 0,315732865 |
| b3323 | 1470 | 0,49 | 0,590640796 | 2,600218198 | 1,747190772 | 0,186230282 | 0,315928888 |
| b1982 | 1455 | 0,5 | 0,33906824 | 7,534895571 | 1,745330166 | 0,186464874 | 0,316176657 |
| b1852 | 1476 | 0,52 | 0,274503627 | 8,252781443 | 1,744913779 | 0,186517421 | 0,316176657 |
| b2004 | 369 | 0,57 | 0,94920563 | 1,709601903 | 1,741175835 | 0,18698991 | 0,316813683 |
| b4538 | 186 | 0,43 | 0,805660008 | 1,481632624 | 1,740822975 | 0,187034584 | 0,316813683 |
| b1861 | 612 | 0,53 | -0,282532948 | 5,703117711 | 1,739492834 | 0,187203101 | 0,316979334 |
| b1688 | 1113 | 0,55 | -0,271302463 | 7,020044797 | 1,737370846 | 0,187472303 | 0,31731528 |

| | | | | | | | |
|---|---|---|---|---|---|---|---|
| b2533 | 804 | 0,54 | 0,486367561 | 5,362521213 | 1,733759895 | 0,187931436 | 0,317972327 |
| b3491 | 1053 | 0,47 | -0,534811147 | 7,703387681 | 1,7299523 | 0,188416989 | 0,318673565 |
| b1740 | 828 | 0,5 | 0,253842205 | 7,405107466 | 1,727178643 | 0,188771612 | 0,319152911 |
| b3359 | 1221 | 0,55 | 0,32973914 | 5,091497096 | 1,726244538 | 0,188891216 | 0,319234702 |
| b2298 | 1521 | 0,52 | 0,604503652 | 3,569266076 | 1,723183241 | 0,189283807 | 0,319718064 |
| b2768 | 576 | 0,47 | -0,557833468 | 3,04905482 | 1,722902259 | 0,189319889 | 0,319718064 |
| b2246 | 1290 | 0,53 | 0,44973473 | 3,109179362 | 1,721743576 | 0,189468763 | 0,319848963 |
| b4311 | 717 | 0,43 | 0,667821609 | 2,124027352 | 1,719731411 | 0,189727622 | 0,320165362 |
| b0727 | 1218 | 0,56 | 0,595375883 | 11,65757003 | 1,717691796 | 0,189990433 | 0,32048819 |
| b3488 | 1623 | 0,37 | -0,468671315 | 3,560698816 | 1,712564637 | 0,19065296 | 0,321484788 |
| b1527 | 1116 | 0,4 | 0,851631145 | 1,143945064 | 1,711461094 | 0,190795911 | 0,321604841 |
| b2131 | 918 | 0,54 | 0,28353691 | 7,356848504 | 1,709697493 | 0,191024624 | 0,32186931 |
| b3471 | 666 | 0,51 | 0,488101124 | 2,977111592 | 1,70808563 | 0,191233939 | 0,322100907 |
| b3439 | 696 | 0,52 | -0,292063722 | 6,177283868 | 1,706982304 | 0,19137737 | 0,322221401 |
| b3785 | 1047 | 0,53 | 0,225952095 | 6,666753792 | 1,705705243 | 0,191543543 | 0,322295568 |
| b3978 | 75 | 0,57 | 1,358727874 | 0,582092028 | 1,705538613 | 0,191565237 | 0,322295568 |
| b0134 | 795 | 0,55 | -0,259121144 | 7,953792716 | 1,704903952 | 0,191647894 | 0,322313644 |
| b2599 | 1161 | 0,49 | -0,31843477 | 6,609499983 | 1,70343169 | 0,191839798 | 0,322515369 |
| b2765 | 366 | 0,49 | 0,719377489 | 2,700957127 | 1,701686231 | 0,192067602 | 0,322777276 |
| b4637 | 87 | 0,4 | 1,357331274 | 0,582591734 | 1,700633752 | 0,192205117 | 0,322887307 |
| b2834 | 1041 | 0,54 | 0,251162693 | 7,973515164 | 1,699429623 | 0,192362587 | 0,323030766 |
| b4209 | 663 | 0,53 | -0,770584394 | 6,012966398 | 1,688433499 | 0,193807597 | 0,32533545 |
| b2110 | 720 | 0,39 | 1,348802156 | 1,108582787 | 1,684208533 | 0,194366175 | 0,326150953 |
| b4685 | 81 | 0,4 | 1,568346656 | 0,4442836 | 1,680468072 | 0,194862268 | 0,326861035 |
| b1820 | 459 | 0,45 | 0,520568773 | 3,844200881 | 1,677501809 | 0,195256734 | 0,327400181 |
| b1059 | 1119 | 0,52 | 0,265075292 | 6,032161985 | 1,674149734 | 0,195703632 | 0,328026806 |
| b0708 | 1419 | 0,53 | -0,271817462 | 6,513023183 | 1,673090247 | 0,195845132 | 0,328141264 |
| b2915 | 321 | 0,53 | -0,70369989 | 1,725079625 | 1,671194369 | 0,196098635 | 0,328443229 |
| b2504 | 192 | 0,49 | -0,416435519 | 4,038998518 | 1,668538477 | 0,196454408 | 0,328916196 |
| b0640 | 1032 | 0,52 | 0,29856871 | 6,582177994 | 1,66466594 | 0,196974514 | 0,329663843 |
| b0217 | 786 | 0,43 | 0,835989283 | 1,527731621 | 1,653007361 | 0,198550107 | 0,332176775 |
| b2473 | 699 | 0,54 | -0,277094642 | 6,460672762 | 1,647153915 | 0,199346742 | 0,33338511 |
| b3039 | 789 | 0,54 | 0,414311438 | 3,859173721 | 1,646283612 | 0,199465507 | 0,333459307 |
| b0893 | 1293 | 0,53 | -0,320034488 | 9,869385214 | 1,644124256 | 0,199760542 | 0,333828019 |
| b3531 | 1107 | 0,55 | -0,444817777 | 3,413794002 | 1,643001434 | 0,199914156 | 0,333960212 |
| b4640 | 533 | 0,41 | -0,52000794 | 2,952598867 | 1,632269231 | 0,201389469 | 0,336299404 |
| b1157 | 501 | 0,47 | 1,31786372 | 0,735811882 | 1,629343417 | 0,201793887 | 0,336849237 |
| b1567 | 183 | 0,44 | 1,180431267 | 0,877802184 | 1,627747877 | 0,202014832 | 0,337092508 |
| b3595 | 945 | 0,48 | -0,565526616 | 3,19819563 | 1,624882349 | 0,202412355 | 0,337630137 |
| b3056 | 1239 | 0,56 | 0,29380443 | 7,189180042 | 1,615565433 | 0,203711235 | 0,339670296 |
| b0158 | 801 | 0,53 | 0,41759 | 4,445706782 | 1,614088275 | 0,203918068 | 0,339888724 |
| b3013 | 927 | 0,47 | -0,424229692 | 3,564208386 | 1,610014236 | 0,204489801 | 0,340714977 |
| b2841 | 1419 | 0,51 | -0,357816569 | 5,036968097 | 1,609191006 | 0,204605559 | 0,340781165 |
| b0505 | 483 | 0,51 | 0,493731682 | 3,387201845 | 1,603872005 | 0,205355353 | 0,341902932 |
| b0853 | 477 | 0,48 | 0,332372076 | 5,623806417 | 1,601128433 | 0,205743369 | 0,342421752 |
| b4537 | 252 | 0,48 | 0,752617751 | 2,565621825 | 1,597729426 | 0,206225281 | 0,343096403 |
| b3357 | 633 | 0,49 | 0,272675147 | 9,524156209 | 1,596620828 | 0,206382747 | 0,343230973 |
| b0058 | 660 | 0,54 | 0,32051659 | 5,533955686 | 1,592221485 | 0,207009033 | 0,344101339 |
| b3835 | 1641 | 0,5 | -0,230838086 | 7,057175424 | 1,591866673 | 0,207059641 | 0,344101339 |
| b3680 | 894 | 0,44 | -0,433604395 | 3,054509874 | 1,587973609 | 0,207615888 | 0,344848014 |
| b1582 | 327 | 0,52 | 0,53960008 | 3,266165093 | 1,587645656 | 0,207662827 | 0,344848014 |
| b2493 | 1062 | 0,54 | -0,288279057 | 5,493889971 | 1,587014911 | 0,207753139 | 0,34487021 |
| b2958 | 720 | 0,52 | 0,302334403 | 6,15804923 | 1,585764306 | 0,207932342 | 0,345039895 |
| b0147 | 531 | 0,56 | 0,493359023 | 3,673788298 | 1,578755176 | 0,208940092 | 0,346583823 |
| b2416 | 1728 | 0,5 | -0,30543414 | 11,11542489 | 1,578160388 | 0,209025874 | 0,346597842 |
| b1314 | 789 | 0,54 | 0,561840033 | 2,738766795 | 1,576539833 | 0,209259807 | 0,346857418 |
| b0368 | 852 | 0,54 | 0,452322515 | 2,867037806 | 1,575333015 | 0,209434218 | 0,347018175 |
| b2335 | 513 | 0,49 | 0,892239419 | 1,577960724 | 1,574350023 | 0,209576408 | 0,347125447 |
| b3447 | 1743 | 0,55 | 0,290464008 | 7,944178583 | 1,572562123 | 0,209835321 | 0,347425899 |
| b1270 | 591 | 0,5 | 0,335720661 | 4,873718882 | 1,567157124 | 0,210620349 | 0,3485969 |
| b2194 | 1053 | 0,54 | -0,596723435 | 5,504474637 | 1,564934727 | 0,210944142 | 0,349003929 |
| b4457 | 245 | 0,49 | -0,384113645 | 11,29099423 | 1,562034649 | 0,211367557 | 0,349575421 |
| b2780 | 1638 | 0,52 | 0,275814655 | 9,48098391 | 1,561304429 | 0,211474329 | 0,349622996 |
| b1873 | 1101 | 0,47 | 0,760931009 | 1,802067845 | 1,558666648 | 0,211860555 | 0,350132378 |
| b2546 | 999 | 0,56 | 0,621882842 | 2,604507656 | 1,55704573 | 0,212098307 | 0,350287305 |
| b2550 | 1194 | 0,51 | -0,386542866 | 4,66338153 | 1,556961925 | 0,212110608 | 0,350287305 |
| b3849 | 1452 | 0,53 | -0,245284035 | 6,647901898 | 1,549599131 | 0,21319463 | 0,35194782 |
| b2339 | 564 | 0,46 | 1,487641679 | 0,772133512 | 1,546973867 | 0,213582737 | 0,352458699 |
| b2927 | 1020 | 0,51 | 0,24961337 | 8,116773748 | 1,542015295 | 0,214318082 | 0,35354201 |
| b0891 | 612 | 0,51 | -0,230081684 | 6,95866851 | 1,541356563 | 0,214415996 | 0,353572889 |
| b0424 | 591 | 0,57 | -0,291423128 | 5,917864209 | 1,540521099 | 0,214540257 | 0,353572889 |
| b0394 | 909 | 0,56 | -0,237894366 | 7,609665606 | 1,540243044 | 0,214581632 | 0,353572889 |
| b2721 | 1710 | 0,57 | -0,470408818 | 3,085762616 | 1,539078564 | 0,214755011 | 0,353572889 |
| b1962 | 696 | 0,54 | 0,390219068 | 4,83997915 | 1,538798298 | 0,214796765 | 0,353572889 |
| b1349 | 810 | 0,47 | -0,506534376 | 3,249831863 | 1,538708638 | 0,214810124 | 0,353572889 |
| b3940 | 2433 | 0,58 | 0,236086187 | 6,850410564 | 1,536195994 | 0,215184913 | 0,354059758 |
| b0160 | 1518 | 0,48 | 0,29260857 | 6,110440893 | 1,53556682 | 0,215278883 | 0,354084387 |
| b1480 | 138 | 0,44 | 0,32994129 | 8,14241633 | 1,533813614 | 0,21554099 | 0,354385444 |

| | | | | | | | |
|---|---|---|---|---|---|---|---|
| b4263 | 1503 | 0,54 | -0,229963686 | 7,84520171 | 1,533116561 | 0,215645306 | 0,354426939 |
| b3458 | 1110 | 0,54 | -0,529312607 | 3,033369423 | 1,532385909 | 0,215754715 | 0,354476772 |
| b2735 | 768 | 0,52 | -0,303075051 | 5,999149602 | 1,530329507 | 0,216062998 | 0,354853191 |
| b0072 | 1401 | 0,56 | 0,456144569 | 5,549313883 | 1,528041595 | 0,216406604 | 0,355287326 |
| b0551 | 384 | 0,43 | 0,877435359 | 1,131662791 | 1,525278365 | 0,216822463 | 0,355839722 |
| b0151 | 798 | 0,57 | 0,653307059 | 3,833633982 | 1,523879013 | 0,217033425 | 0,356055568 |
| b3379 | 879 | 0,53 | 0,4918804 | 2,473157835 | 1,522528059 | 0,217237323 | 0,356259672 |
| b0365 | 963 | 0,56 | 0,733776068 | 1,42026865 | 1,52048569 | 0,217546009 | 0,35663541 |
| b3772 | 1545 | 0,59 | -0,311679157 | 6,080618661 | 1,519517555 | 0,217692516 | 0,356745103 |
| b4323 | 1461 | 0,58 | -0,441295145 | 6,504338861 | 1,517504601 | 0,217997512 | 0,356999909 |
| b0602 | 1221 | 0,48 | 0,691875373 | 2,719381893 | 1,517440013 | 0,218007307 | 0,356999909 |
| b0561 | 438 | 0,49 | 1,133881675 | 1,334182608 | 1,511267353 | 0,218945801 | 0,3584058 |
| b3110 | 1332 | 0,51 | 0,446247846 | 6,009028656 | 1,506963684 | 0,219602987 | 0,35930835 |
| b4090 | 450 | 0,5 | 0,388297619 | 3,432137144 | 1,506607439 | 0,219657492 | 0,35930835 |
| b1502 | 915 | 0,45 | 1,031472882 | 1,033743904 | 1,506001471 | 0,219750242 | 0,359328925 |
| b3870 | 1410 | 0,53 | -0,477657439 | 7,636436096 | 1,504087902 | 0,220043441 | 0,359677135 |
| b4314 | 549 | 0,52 | 0,306342034 | 9,340268856 | 1,502545526 | 0,220280106 | 0,359932714 |
| b3952 | 879 | 0,55 | -0,490851753 | 3,199185929 | 1,501863343 | 0,220384879 | 0,359972677 |
| b4296 | 1350 | 0,49 | 0,373587331 | 3,837111676 | 1,500720067 | 0,220560601 | 0,360128457 |
| b2619 | 477 | 0,47 | 0,279834889 | 5,935148243 | 1,499416677 | 0,220761138 | 0,360209741 |
| b0226 | 261 | 0,47 | -0,2937941 | 5,634082473 | 1,499351834 | 0,22077112 | 0,360209741 |
| b0753 | 381 | 0,5 | -0,461529058 | 8,702643764 | 1,498546365 | 0,220895163 | 0,360280975 |
| b0135 | 1239 | 0,4 | 0,544408054 | 2,577583595 | 1,49263549 | 0,221807998 | 0,361638214 |
| b2672 | 330 | 0,54 | -0,374974882 | 9,459886473 | 1,49171829 | 0,221950049 | 0,361738225 |
| b1466 | 696 | 0,56 | -0,449591094 | 6,326002041 | 1,487976776 | 0,22253064 | 0,362552646 |
| b1079 | 699 | 0,54 | 0,69882823 | 1,684278634 | 1,486757097 | 0,222720297 | 0,362729786 |
| b4339 | 921 | 0,49 | -0,353070025 | 4,496109348 | 1,486009958 | 0,222836571 | 0,362787327 |
| b0321 | 1419 | 0,58 | -0,339456324 | 4,021622086 | 1,480847135 | 0,223642027 | 0,363863964 |
| b1639 | 330 | 0,46 | 0,412768382 | 3,468588315 | 1,480730617 | 0,223660246 | 0,363863964 |
| b3487 | 1068 | 0,58 | -0,275603153 | 8,036124088 | 1,480137525 | 0,223753006 | 0,363882792 |
| b2415 | 258 | 0,51 | 0,266337992 | 8,697561049 | 1,477436724 | 0,224176002 | 0,364438462 |
| b1028 | 342 | 0,44 | 1,456515031 | 0,582543843 | 1,476412204 | 0,224336711 | 0,364567491 |
| b2124 | 471 | 0,49 | 0,343340459 | 4,964040585 | 1,474643037 | 0,224614553 | 0,364886708 |
| b4468 | 1053 | 0,58 | 1,392490497 | 8,636836606 | 1,472785956 | 0,224906646 | 0,365228837 |
| b1580 | 1020 | 0,45 | 0,506212597 | 2,814005602 | 1,465625994 | 0,226037083 | 0,366931622 |
| b4085 | 696 | 0,49 | 0,473059023 | 2,895042491 | 1,462344213 | 0,2265575 | 0,367643271 |
| b2928 | 714 | 0,5 | -0,289762003 | 5,203323212 | 1,459111692 | 0,227071513 | 0,368344017 |
| b1958 | 918 | 0,53 | 0,257100935 | 6,507510661 | 1,45804557 | 0,227241347 | 0,36848615 |
| b4504 | 222 | 0,56 | 0,975323225 | 1,097779127 | 1,456899001 | 0,227424167 | 0,368649229 |
| b3472 | 558 | 0,52 | 0,237449428 | 7,467333512 | 1,455574001 | 0,227635659 | 0,368855976 |
| b1449 | 1038 | 0,52 | -0,24329333 | 8,086552855 | 1,45506913 | 0,227716306 | 0,368855976 |
| b0037 | 1554 | 0,51 | -0,438409978 | 3,843005265 | 1,448536118 | 0,22876299 | 0,37032319 |
| b2280 | 555 | 0,54 | 0,533716994 | 9,089930214 | 1,448384485 | 0,228787353 | 0,37032319 |
| b3527 | 1497 | 0,53 | 0,357726252 | 6,024848211 | 1,444335116 | 0,229439111 | 0,371244077 |
| b4280 | 1119 | 0,39 | -0,464668158 | 5,831664525 | 1,439665262 | 0,230193516 | 0,372330328 |
| b2336 | 753 | 0,51 | 0,932572382 | 1,288819324 | 1,437243068 | 0,230585993 | 0,3728306 |
| b4098 | 846 | 0,58 | 0,873644463 | 1,326559188 | 1,431681216 | 0,231490259 | 0,374067074 |
| b4077 | 1314 | 0,53 | 0,26269322 | 6,383024995 | 1,431299788 | 0,231552429 | 0,374067074 |
| b1340 | 564 | 0,54 | 0,274238465 | 4,996798454 | 1,431001272 | 0,2316011 | 0,374067074 |
| b4533 | 234 | 0,44 | 1,237358615 | 0,926357129 | 1,428158542 | 0,232065201 | 0,374681639 |
| b1258 | 501 | 0,43 | -0,556131839 | 5,513731656 | 1,422280427 | 0,23302842 | 0,376101325 |
| b4037 | 921 | 0,53 | -0,682808465 | 8,491603058 | 1,421587852 | 0,233142227 | 0,376149554 |
| b0043 | 1287 | 0,55 | -0,701126229 | 2,494908663 | 1,413636851 | 0,234453592 | 0,377994103 |
| b3106 | 702 | 0,53 | -0,359183822 | 3,898534846 | 1,413633339 | 0,234454174 | 0,377994103 |
| b1895 | 429 | 0,45 | -0,310080897 | 5,355832677 | 1,41158045 | 0,234794207 | 0,378406197 |
| b0437 | 624 | 0,51 | 0,237651756 | 8,855515035 | 1,409408565 | 0,2351546 | 0,378850796 |
| b0631 | 264 | 0,46 | 0,462684924 | 6,194896627 | 1,404483105 | 0,235974393 | 0,380034936 |
| b0953 | 168 | 0,54 | -0,383885724 | 10,82856788 | 1,402046225 | 0,236381266 | 0,380553461 |
| b3992 | 756 | 0,58 | 0,591620691 | 2,812825048 | 1,399079844 | 0,236877696 | 0,381215738 |
| b1353 | 489 | 0,37 | -0,771128878 | 2,137882368 | 1,395692208 | 0,237446169 | 0,381993442 |
| b0014 | 1917 | 0,51 | -0,613199417 | 11,66227831 | 1,394220848 | 0,237693591 | 0,382254278 |
| b4527 | 156 | 0,49 | -1,182332224 | 0,954620004 | 1,393141829 | 0,237875236 | 0,382409185 |
| b4093 | 435 | 0,58 | 0,400389413 | 4,412079987 | 1,389665799 | 0,238461546 | 0,383214288 |
| b1592 | 1257 | 0,54 | -0,395840448 | 3,967996814 | 1,388296884 | 0,238692926 | 0,383448636 |
| b3237 | 471 | 0,49 | 0,223422912 | 6,419434085 | 1,385860527 | 0,239105403 | 0,383973636 |
| b3499 | 843 | 0,54 | 0,421244771 | 5,373904829 | 1,382719945 | 0,239638385 | 0,384691705 |
| b0555 | 498 | 0,48 | -0,464950094 | 3,265236512 | 1,381101862 | 0,239913549 | 0,384995535 |
| b2297 | 2145 | 0,56 | 0,335067694 | 9,339587603 | 1,380338609 | 0,240043478 | 0,385066167 |
| b2449 | 393 | 0,44 | -0,376039667 | 5,118404716 | 1,372471499 | 0,2413877 | 0,38708396 |
| b2419 | 375 | 0,46 | 0,760099577 | 2,65481592 | 1,370796909 | 0,241675011 | 0,38740608 |
| b2078 | 1404 | 0,54 | 0,361021694 | 4,070952846 | 1,36774335 | 0,242199986 | 0,388108809 |
| b1346 | 216 | 0,45 | 1,209339386 | 0,642934573 | 1,366643926 | 0,242389342 | 0,38827342 |
| b2007 | 330 | 0,48 | -0,236354613 | 8,143147617 | 1,365860362 | 0,242524406 | 0,388350978 |
| b4436 | 143 | 0,48 | -0,349230242 | 4,146047579 | 1,361353009 | 0,243303131 | 0,389432153 |
| b0040 | 1515 | 0,52 | 0,643450221 | 1,749672015 | 1,3609473 | 0,243373374 | 0,389432153 |
| b4450 | 121 | 0,52 | 0,494058293 | 2,557311161 | 1,358291408 | 0,243833815 | 0,390029679 |
| b4558 | 204 | 0,52 | -0,279069685 | 5,563512521 | 1,355176591 | 0,244375173 | 0,390756163 |
| b2156 | 1470 | 0,53 | -0,212904313 | 7,009870233 | 1,352850653 | 0,244780379 | 0,391256451 |

| | | | | | | | |
|---|---|---|---|---|---|---|---|
| b0935 | 1146 | 0,57 | 0,556383204 | 2,378216691 | 1,352379051 | 0,244862638 | 0,391256451 |
| b0864 | 729 | 0,53 | -0,277791103 | 8,116974514 | 1,350579449 | 0,245176842 | 0,391618891 |
| b4102 | 726 | 0,62 | 1,132272687 | 1,12542998 | 1,347699701 | 0,245680661 | 0,392165566 |
| b1779 | 996 | 0,5 | -0,326171282 | 12,30147782 | 1,347623046 | 0,245694089 | 0,392165566 |
| b4256 | 504 | 0,52 | 0,370836557 | 4,183091454 | 1,346561151 | 0,245880202 | 0,392322914 |
| b3464 | 1494 | 0,53 | 0,220690671 | 7,853701897 | 1,34509833 | 0,246136865 | 0,392592679 |
| b0878 | 1116 | 0,53 | -0,263328793 | 5,8613038 | 1,339407766 | 0,247138437 | 0,394049973 |
| b2548 | 984 | 0,46 | -0,498929204 | 3,095903833 | 1,332777794 | 0,248311646 | 0,3957798 |
| b4458 | 110 | 0,49 | 1,383607292 | 0,509330308 | 1,331472217 | 0,248543478 | 0,395982414 |
| b0693 | 2199 | 0,49 | 0,470664957 | 2,995816877 | 1,330659046 | 0,248688007 | 0,395982414 |
| b0616 | 909 | 0,58 | -0,580938767 | 2,572852678 | 1,330570141 | 0,248703814 | 0,395982414 |
| b1358 | 423 | 0,51 | 0,621836188 | 2,372464241 | 1,32633525 | 0,249458229 | 0,397042536 |
| b4657 | 246 | 0,42 | 0,937599052 | 1,022910579 | 1,325340441 | 0,249635853 | 0,3971842 |
| b0297 | 872 | 0,48 | 1,05826841 | 0,735205744 | 1,323265762 | 0,250006789 | 0,397633224 |
| b1136 | 1251 | 0,5 | -0,332986482 | 11,83672726 | 1,319695219 | 0,250646757 | 0,398509671 |
| b1445 | 174 | 0,45 | 0,733839383 | 1,787464203 | 1,318761869 | 0,250814377 | 0,398613092 |
| b2664 | 663 | 0,54 | 0,364485382 | 7,507051955 | 1,318342832 | 0,250889677 | 0,398613092 |
| b2034 | 993 | 0,36 | 0,325732984 | 6,921159766 | 1,316967262 | 0,251137058 | 0,39886474 |
| b0511 | 1455 | 0,47 | 0,60832137 | 1,956885507 | 1,308838941 | 0,252604979 | 0,400904928 |
| b2732 | 354 | 0,51 | -0,744019373 | 3,918179232 | 1,307989307 | 0,252759025 | 0,400904928 |
| b1730 | 804 | 0,35 | 0,893975586 | 1,154067173 | 1,307980647 | 0,252760596 | 0,400904928 |
| b2822 | 3369 | 0,54 | -0,196168268 | 7,107837895 | 1,307876911 | 0,252779412 | 0,400904928 |
| b4487 | 330 | 0,56 | 0,482160752 | 3,305343833 | 1,306045087 | 0,253111964 | 0,401290351 |
| b4027 | 639 | 0,55 | 0,956876042 | 1,000592966 | 1,300601576 | 0,254103368 | 0,402719694 |
| b4169 | 1338 | 0,55 | -0,265286176 | 7,14339981 | 1,29946447 | 0,254311068 | 0,4029064 |
| b3979 | 76 | 0,54 | 1,363901216 | 0,509185644 | 1,292039205 | 0,255672491 | 0,404920178 |
| b1862 | 603 | 0,44 | 0,881601052 | 1,850465154 | 1,289790135 | 0,25608663 | 0,405432806 |
| b1110 | 540 | 0,54 | 0,428226923 | 4,035567809 | 1,288159545 | 0,2563874 | 0,405547999 |
| b1540 | 687 | 0,5 | 0,354016012 | 5,09084026 | 1,288122253 | 0,256394284 | 0,405547999 |
| b3889 | 213 | 0,46 | 1,172738486 | 0,727665819 | 1,287924231 | 0,256430841 | 0,405547999 |
| b1259 | 180 | 0,51 | 0,580050196 | 4,896170921 | 1,285716696 | 0,256838812 | 0,406049931 |
| b0507 | 1782 | 0,53 | 0,619911445 | 5,024844232 | 1,278621121 | 0,258155572 | 0,407987755 |
| b1775 | 1380 | 0,49 | 0,558662445 | 2,615282908 | 1,276162841 | 0,258613711 | 0,408567731 |
| b2985 | 714 | 0,5 | 0,917977068 | 1,210475094 | 1,275398738 | 0,258756318 | 0,408648985 |
| b2743 | 627 | 0,53 | -0,196382154 | 6,986978037 | 1,27335432 | 0,259138354 | 0,409108172 |
| b0227 | 750 | 0,52 | 0,414855305 | 3,646540957 | 1,269213073 | 0,259914359 | 0,409990289 |
| b2757 | 675 | 0,48 | -0,391344704 | 3,604375159 | 1,269010067 | 0,259952473 | 0,409990289 |
| b4143 | 1647 | 0,53 | -0,59043187 | 12,10022416 | 1,268908572 | 0,259971531 | 0,409990289 |
| b3339 | 1185 | 0,53 | 0,293936614 | 11,60382575 | 1,256333394 | 0,262346272 | 0,413589866 |
| b3239 | 273 | 0,43 | -0,314470921 | 5,641179533 | 1,251478333 | 0,263270311 | 0,414900681 |
| b2429 | 1425 | 0,57 | -0,326686494 | 5,193739757 | 1,250907383 | 0,263379243 | 0,414926456 |
| b3769 | 264 | 0,49 | 0,938657301 | 1,120451073 | 1,248315667 | 0,26387442 | 0,415560488 |
| b4541 | 318 | 0,39 | 1,338827926 | 0,700248254 | 1,247040099 | 0,264118556 | 0,415667032 |
| b1061 | 246 | 0,48 | 0,416511571 | 3,859067124 | 1,246993104 | 0,264127556 | 0,415667032 |
| b0958 | 510 | 0,48 | -0,294473515 | 6,865760306 | 1,24601735 | 0,264314507 | 0,415732949 |
| b3688 | 333 | 0,54 | -0,272001093 | 6,70710693 | 1,245806358 | 0,264354954 | 0,415732949 |
| b0415 | 471 | 0,52 | 0,230210321 | 6,806322632 | 1,244603788 | 0,264585633 | 0,415949774 |
| b4194 | 306 | 0,51 | -0,500446134 | 2,660974731 | 1,240877738 | 0,265301959 | 0,416929657 |
| b4636 | 150 | 0,47 | 1,33370492 | 0,455242862 | 1,239352738 | 0,265595833 | 0,417245189 |
| b0496 | 2415 | 0,58 | 0,239912909 | 5,917093323 | 1,235524472 | 0,266335344 | 0,41826034 |
| b2353 | 291 | 0,54 | -0,383748115 | 3,708918875 | 1,234369849 | 0,266558887 | 0,418464774 |
| b3328 | 438 | 0,49 | 0,819192477 | 1,245211996 | 1,231567773 | 0,26710236 | 0,419171141 |
| b2983 | 1068 | 0,53 | 0,524784419 | 2,298907328 | 1,223605636 | 0,268654203 | 0,421458922 |
| b3104 | 357 | 0,45 | -0,735652079 | 1,856901618 | 1,219932762 | 0,269373848 | 0,422440023 |
| b4693 | 1134 | 0,54 | 0,481675456 | 2,620126921 | 1,211808168 | 0,270974314 | 0,424709441 |
| b1042 | 456 | 0,51 | -0,419089684 | 3,472675445 | 1,211625238 | 0,271010486 | 0,424709441 |
| b1457 | 483 | 0,27 | 0,897803396 | 1,471687468 | 1,208370572 | 0,271655066 | 0,425570782 |
| b1846 | 660 | 0,53 | -0,2962279 | 5,64922782 | 1,205448311 | 0,272235449 | 0,426330987 |
| b1892 | 351 | 0,46 | 0,49094027 | 3,358332947 | 1,204349382 | 0,272454107 | 0,426524382 |
| b3724 | 726 | 0,53 | -0,430000583 | 6,648167578 | 1,200786888 | 0,273164461 | 0,427351719 |
| b4502 | 255 | 0,56 | 0,581773854 | 2,831465914 | 1,200742697 | 0,273173288 | 0,427351719 |
| b1756 | 654 | 0,51 | 0,448600098 | 3,341311833 | 1,198737706 | 0,273574116 | 0,427811088 |
| b0886 | 1722 | 0,54 | -0,244930947 | 7,063109116 | 1,198143837 | 0,273692981 | 0,427811088 |
| b2049 | 1437 | 0,55 | 0,604168609 | 2,350299628 | 1,197842699 | 0,27375328 | 0,427811088 |
| b0989 | 213 | 0,47 | 1,128701771 | 0,933561727 | 1,195674213 | 0,274187981 | 0,42834107 |
| b0509 | 879 | 0,51 | 0,822741852 | 4,270595559 | 1,191522551 | 0,275022655 | 0,429357308 |
| b2706 | 360 | 0,55 | -0,621658281 | 2,492363766 | 1,191485674 | 0,275030083 | 0,429357308 |
| b3963 | 648 | 0,53 | -0,19394313 | 7,03820165 | 1,19076611 | 0,275175077 | 0,429434086 |
| b1559 | 753 | 0,48 | 0,471253042 | 2,87338149 | 1,187863376 | 0,275760963 | 0,430088788 |
| b4587 | 1912 | 0,52 | -0,313027456 | 4,801791813 | 1,187359945 | 0,275862734 | 0,430088788 |
| b2641 | 1016 | 0,55 | 0,327224446 | 3,806104409 | 1,186860004 | 0,275963847 | 0,430088788 |
| b3120 | 540 | 0,36 | 1,007040951 | 1,159233945 | 1,186787885 | 0,275978437 | 0,430088788 |
| b0981 | 2181 | 0,51 | -0,243964492 | 5,295305925 | 1,180419337 | 0,277270643 | 0,431952389 |
| b0242 | 1104 | 0,56 | 0,206150838 | 7,451340255 | 1,178500099 | 0,277661557 | 0,432411084 |
| b0179 | 1026 | 0,53 | 0,249052238 | 9,062711263 | 1,177372038 | 0,277891646 | 0,43261909 |
| b1729 | 1392 | 0,52 | -0,267383496 | 5,252795262 | 1,176841125 | 0,278000019 | 0,43263753 |
| b2242 | 1260 | 0,56 | 0,541394348 | 2,24830739 | 1,168978151 | 0,279611302 | 0,43499405 |
| b1774 | 1044 | 0,47 | 0,519483109 | 2,720453217 | 1,165978528 | 0,280229088 | 0,435645931 |

| | | | | | | | |
|---|---|---|---|---|---|---|---|
| b1972 | 636 | 0,48 | -0,346951653 | 4,425764861 | 1,164959834 | 0,280439284 | 0,435645931 |
| b4104 | 788 | 0,62 | -0,896182629 | 1,585378167 | 1,164643514 | 0,280504594 | 0,435645931 |
| b3892 | 636 | 0,53 | -0,355485072 | 9,117048851 | 1,164594625 | 0,28051469 | 0,435645931 |
| b0575 | 3144 | 0,56 | 0,291191388 | 4,473815204 | 1,164301781 | 0,280575172 | 0,435645931 |
| b1960 | 471 | 0,56 | 0,475874738 | 3,248185797 | 1,164116143 | 0,280613521 | 0,435645931 |
| b4061 | 1587 | 0,45 | -0,255391103 | 5,87374105 | 1,162117735 | 0,281026773 | 0,436136426 |
| b3273 | 76 | 0,57 | 1,291473088 | 0,478185471 | 1,161445816 | 0,281165893 | 0,436201291 |
| b1611 | 1404 | 0,54 | 0,322696345 | 7,590684476 | 1,158278108 | 0,281822932 | 0,437069336 |
| b2385 | 1086 | 0,57 | 0,522660226 | 2,227618072 | 1,155083253 | 0,282487569 | 0,43794856 |
| b1120 | 729 | 0,52 | 0,215232462 | 6,76263751 | 1,153131368 | 0,282894603 | 0,438427943 |
| b3437 | 528 | 0,5 | -0,392409409 | 3,786659395 | 1,149750828 | 0,283601316 | 0,439371275 |
| b3154 | 444 | 0,54 | -0,341683316 | 4,203070314 | 1,147335978 | 0,284107518 | 0,440003419 |
| b2839 | 936 | 0,53 | -0,292103019 | 4,375725626 | 1,146130914 | 0,284360552 | 0,440243176 |
| b0035 | 591 | 0,55 | -0,466610672 | 2,572903229 | 1,142695046 | 0,28508357 | 0,441210139 |
| b0319 | 864 | 0,47 | 0,746989229 | 1,455638475 | 1,138907795 | 0,285883234 | 0,442295014 |
| b1790 | 822 | 0,5 | -0,304419321 | 4,287760349 | 1,13538488 | 0,286629642 | 0,443296776 |
| b4083 | 1986 | 0,53 | 0,538116054 | 2,274908447 | 1,133500026 | 0,287030006 | 0,443762845 |
| b1677 | 237 | 0,51 | 0,298165693 | 13,13960455 | 1,131453662 | 0,287465482 | 0,444282859 |
| b0898 | 1149 | 0,52 | -0,285502076 | 4,687656503 | 1,130381164 | 0,287694051 | 0,444482845 |
| b1065 | 1209 | 0,55 | -0,442568361 | 3,44042464 | 1,127966511 | 0,288209502 | 0,445125771 |
| b0910 | 684 | 0,54 | 0,256831012 | 6,643433788 | 1,125772163 | 0,288678944 | 0,445605423 |
| b4268 | 564 | 0,43 | -0,366919997 | 3,207478761 | 1,125585565 | 0,288718908 | 0,445605423 |
| b3131 | 810 | 0,52 | 0,239035894 | 6,375180146 | 1,122403837 | 0,289401434 | 0,446505069 |
| b2783 | 249 | 0,49 | -0,277919806 | 5,012937539 | 1,120647298 | 0,289779117 | 0,44693393 |
| b2488 | 546 | 0,55 | 0,465021598 | 2,359551454 | 1,11862013 | 0,29021577 | 0,44740643 |
| b1621 | 1593 | 0,53 | -0,30910124 | 5,879223058 | 1,118137946 | 0,290319756 | 0,44740643 |
| b3949 | 1080 | 0,54 | -0,380258503 | 3,323156094 | 1,117723101 | 0,290409258 | 0,44740643 |
| b0418 | 519 | 0,53 | -0,260090837 | 6,371981862 | 1,117373256 | 0,290484764 | 0,44740643 |
| b2282 | 978 | 0,54 | 0,481023433 | 9,791934984 | 1,110960587 | 0,291873233 | 0,449317097 |
| b2629 | 264 | 0,45 | 0,859755331 | 1,792262118 | 1,110718624 | 0,291925789 | 0,449317097 |
| b2263 | 759 | 0,56 | -0,219640041 | 5,422971524 | 1,106902995 | 0,292756161 | 0,450059602 |
| b4577 | 227 | 0,48 | -0,399553202 | 3,723957714 | 1,106656983 | 0,292809802 | 0,450059602 |
| b2109 | 2481 | 0,46 | 0,419628193 | 2,682157737 | 1,106449573 | 0,292855037 | 0,450059602 |
| b3850 | 546 | 0,52 | 0,217928444 | 5,592182731 | 1,106250447 | 0,292898473 | 0,450059602 |
| b3380 | 1227 | 0,54 | 0,48248226 | 2,466338641 | 1,106196343 | 0,292910276 | 0,450059602 |
| b3790 | 675 | 0,56 | 0,25284547 | 5,314231806 | 1,103391332 | 0,293523051 | 0,450791117 |
| b2662 | 1281 | 0,59 | -0,36795038 | 10,46964411 | 1,103096653 | 0,293587521 | 0,450791117 |
| b3248 | 594 | 0,54 | 0,242466654 | 5,836999071 | 1,099556774 | 0,294363394 | 0,451827649 |
| b4244 | 462 | 0,48 | 0,529823228 | 6,287674575 | 1,094911778 | 0,295385474 | 0,453241252 |
| b0404 | 582 | 0,53 | 0,317734577 | 4,231493981 | 1,093451682 | 0,295707691 | 0,453579052 |
| b4500 | 4605 | 0,54 | -0,297466698 | 4,646363338 | 1,09265132 | 0,295884508 | 0,453579052 |
| b0483 | 342 | 0,45 | 0,295021147 | 5,185659087 | 1,092539485 | 0,295909225 | 0,453579052 |
| b1081 | 942 | 0,55 | 0,6703075 | 1,967110502 | 1,090149401 | 0,296438109 | 0,454234394 |
| b1047 | 1158 | 0,44 | 0,321145747 | 3,771882673 | 1,088780487 | 0,296741572 | 0,454543993 |
| b4198 | 687 | 0,57 | -0,322136328 | 3,242296555 | 1,085806455 | 0,297402234 | 0,455400346 |
| b2141 | 399 | 0,49 | -0,347892362 | 3,607491667 | 1,085163986 | 0,297545203 | 0,455449465 |
| b0289 | 711 | 0,53 | 0,365465423 | 3,415945764 | 1,084749223 | 0,297637547 | 0,455449465 |
| b3207 | 633 | 0,51 | 0,25289212 | 8,984352309 | 1,08124697 | 0,298418768 | 0,456388567 |
| b1842 | 231 | 0,48 | 0,310497029 | 3,86852241 | 1,080743533 | 0,298531282 | 0,456388567 |
| b1316 | 2268 | 0,54 | 0,405674494 | 3,463349593 | 1,080629688 | 0,298556733 | 0,456388567 |
| b3765 | 1521 | 0,51 | 0,293037058 | 3,521477302 | 1,079984366 | 0,298701054 | 0,456453502 |
| b0850 | 288 | 0,5 | 0,276353096 | 4,17150171 | 1,077696268 | 0,299213489 | 0,457080728 |
| b3175 | 333 | 0,5 | 0,401920548 | 7,506069006 | 1,076994204 | 0,299370947 | 0,457165447 |
| b3994 | 1896 | 0,56 | 0,307312219 | 4,186531103 | 1,075845411 | 0,299628828 | 0,457392322 |
| b0164 | 813 | 0,51 | 0,422848721 | 3,045692596 | 1,075423473 | 0,299723617 | 0,457392322 |
| b3481 | 402 | 0,57 | -0,363140962 | 4,356495557 | 1,069506349 | 0,301056977 | 0,459270718 |
| b1970 | 414 | 0,42 | 0,352645082 | 3,009917656 | 1,067951868 | 0,301408529 | 0,459497344 |
| b3592 | 609 | 0,53 | 0,310133592 | 5,598137451 | 1,067942833 | 0,301410574 | 0,459497344 |
| b4649 | 192 | 0,53 | 0,803807228 | 1,140179318 | 1,067123988 | 0,301595974 | 0,45962365 |
| b0051 | 822 | 0,53 | 0,206556349 | 7,388726629 | 1,064859075 | 0,302109553 | 0,460111598 |
| b2249 | 1203 | 0,54 | 0,195980433 | 6,471592865 | 1,064806576 | 0,302121471 | 0,460111598 |
| b0567 | 891 | 0,52 | 0,390124151 | 3,896621847 | 1,063994856 | 0,302305816 | 0,46023596 |
| b3884 | 786 | 0,52 | -0,265477102 | 5,794865062 | 1,062002045 | 0,302759009 | 0,460769398 |
| b4332 | 1179 | 0,54 | 0,336916019 | 4,00280238 | 1,060704168 | 0,303054636 | 0,461062755 |
| b2825 | 564 | 0,52 | 0,399579689 | 2,781467532 | 1,059484474 | 0,303332794 | 0,461329346 |
| b0428 | 891 | 0,54 | 0,387944943 | 10,43142282 | 1,055241574 | 0,304302986 | 0,462634576 |
| b2886 | 489 | 0,55 | 0,508501096 | 2,280950383 | 1,0548294 | 0,304397448 | 0,462634576 |
| b4015 | 1305 | 0,55 | 0,304194525 | 13,99158794 | 1,053460103 | 0,304711538 | 0,46288461 |
| b3194 | 783 | 0,55 | 0,200334835 | 6,19778217 | 1,053211902 | 0,304768515 | 0,46288461 |
| b4066 | 1293 | 0,33 | 0,574808023 | 1,561334135 | 1,052638455 | 0,304900209 | 0,462927756 |
| b2806 | 1101 | 0,52 | 0,20142377 | 6,044572843 | 1,049745143 | 0,30556579 | 0,463781196 |
| b3924 | 747 | 0,54 | -0,179592597 | 6,442081921 | 1,048373726 | 0,30588193 | 0,464103862 |
| b4048 | 708 | 0,37 | 0,860362773 | 1,061282385 | 1,047317401 | 0,306125723 | 0,464194039 |
| b1477 | 285 | 0,51 | 0,325520053 | 4,011821193 | 1,047218766 | 0,306148501 | 0,464194039 |
| b3803 | 1182 | 0,53 | -0,202737173 | 8,377519983 | 1,045929562 | 0,306446411 | 0,464488608 |
| b4333 | 861 | 0,43 | -0,460800026 | 3,297320706 | 1,043564362 | 0,306993942 | 0,465161206 |
| b0295 | 228 | 0,44 | 0,754428765 | 1,332243584 | 1,038119198 | 0,3082593 | 0,466920644 |
| b3918 | 756 | 0,49 | 0,385256951 | 3,272567202 | 1,032202996 | 0,309641808 | 0,468856279 |

| | | | | | | | |
|---|---|---|---|---|---|---|---|
| b3243 | 930 | 0,53 | -0,270694255 | 5,244065315 | 1,030910268 | 0,309944968 | 0,469156821 |
| b2947 | 951 | 0,53 | 0,191656192 | 7,232484561 | 1,027416805 | 0,310766159 | 0,470241028 |
| b1771 | 981 | 0,48 | 0,344458898 | 3,346421984 | 1,026221105 | 0,311047877 | 0,470508466 |
| b1744 | 969 | 0,53 | 0,415097293 | 6,438979029 | 1,025076873 | 0,31131778 | 0,470757858 |
| b1708 | 465 | 0,5 | 0,204231303 | 6,612195374 | 1,022646022 | 0,311892186 | 0,471300131 |
| b0728 | 1167 | 0,54 | 0,43624274 | 11,88029305 | 1,022564753 | 0,311911414 | 0,471300131 |
| b3707 | 75 | 0,35 | 1,215989525 | 0,530974521 | 1,02222483 | 0,311991854 | 0,471300131 |
| b1319 | 906 | 0,5 | 1,038342631 | 0,932046231 | 1,020323331 | 0,312442326 | 0,471821599 |
| b1256 | 639 | 0,5 | -0,493473567 | 8,282133834 | 1,018622469 | 0,312845986 | 0,472272048 |
| b4196 | 651 | 0,57 | -0,350929544 | 3,925748648 | 1,017074166 | 0,313214032 | 0,472668448 |
| b2215 | 1104 | 0,51 | -0,289777479 | 14,73654399 | 1,014837078 | 0,313746807 | 0,47331309 |
| b2830 | 531 | 0,49 | 0,198428446 | 7,603456077 | 1,010221676 | 0,314849738 | 0,474817136 |
| b4648 | 102 | 0,44 | 1,127667849 | 0,531570847 | 1,004334555 | 0,316263941 | 0,476789432 |
| b3329 | 510 | 0,56 | 0,753294761 | 1,112063637 | 1,000391397 | 0,31721582 | 0,478063653 |
| b2850 | 490 | 0,32 | 0,974411922 | 0,613437756 | 0,999636457 | 0,317398491 | 0,478178163 |
| b4705 | 129 | 0,41 | 0,446927978 | 2,833415756 | 0,999112503 | 0,317525351 | 0,478208543 |
| b2865 | 756 | 0,51 | -0,183889097 | 6,239632038 | 0,996239359 | 0,318222187 | 0,479097025 |
| b3014 | 258 | 0,48 | 0,670258132 | 1,719420409 | 0,993652447 | 0,31885132 | 0,479883014 |
| b3395 | 780 | 0,58 | -0,351130304 | 3,234208399 | 0,992800317 | 0,319058914 | 0,480034258 |
| b4408 | 369 | 0,51 | 0,289759373 | 15,07898474 | 0,990078622 | 0,319723159 | 0,480872215 |
| b4042 | 369 | 0,52 | -0,211087266 | 5,302154695 | 0,989222778 | 0,319932409 | 0,481025513 |
| b3792 | 1251 | 0,54 | -0,222936957 | 6,086436496 | 0,9860283 | 0,320715034 | 0,482040504 |
| b2279 | 303 | 0,55 | 0,450157397 | 7,405798231 | 0,984231699 | 0,321156296 | 0,482495072 |
| b2119 | 1089 | 0,54 | 0,769745933 | 1,220187778 | 0,983764771 | 0,321271109 | 0,482495072 |
| b1936 | 162 | 0,5 | 1,184513007 | 0,593512072 | 0,983482974 | 0,321340426 | 0,482495072 |
| b2831 | 690 | 0,54 | -0,306922975 | 4,134220073 | 0,981231968 | 0,321894842 | 0,483046791 |
| b4262 | 1083 | 0,54 | 0,182698807 | 7,228238784 | 0,98111608 | 0,321923419 | 0,483046791 |
| b2255 | 1983 | 0,51 | 0,264598801 | 6,400513597 | 0,977518265 | 0,322812275 | 0,484218413 |
| b3950 | 321 | 0,49 | -0,479445193 | 2,27099773 | 0,976651763 | 0,323026832 | 0,48437814 |
| b1696 | 912 | 0,42 | -0,266178466 | 4,164314411 | 0,97160394 | 0,324280486 | 0,485955753 |
| b0955 | 1761 | 0,52 | -0,206511647 | 7,809943101 | 0,971542545 | 0,324295774 | 0,485955753 |
| b3236 | 939 | 0,52 | -0,252087812 | 12,08113307 | 0,970688237 | 0,324508594 | 0,486112139 |
| b1568 | 279 | 0,51 | 1,013791464 | 1,197053981 | 0,967224527 | 0,325373351 | 0,487145918 |
| b1247 | 1005 | 0,52 | 0,482314758 | 10,03633785 | 0,967053687 | 0,325416082 | 0,487145918 |
| b2226 | 1650 | 0,55 | 0,322977493 | 3,069671876 | 0,964207615 | 0,326129044 | 0,488050209 |
| b2668 | 525 | 0,46 | -0,37750904 | 4,584480562 | 0,96153157 | 0,326801301 | 0,488893001 |
| b4684 | 126 | 0,34 | 1,178674266 | 0,583305618 | 0,960493387 | 0,327062601 | 0,489120646 |
| b3839 | 777 | 0,52 | 0,348061493 | 5,937932207 | 0,959052075 | 0,327425822 | 0,489432594 |
| b2434 | 426 | 0,52 | 0,243771552 | 5,971641467 | 0,95879925 | 0,327489592 | 0,489432594 |
| b2122 | 2001 | 0,54 | 0,443890942 | 3,418267444 | 0,95828982 | 0,327618133 | 0,48946149 |
| b1733 | 750 | 0,51 | -0,277030776 | 4,294495942 | 0,957688196 | 0,327770023 | 0,489525239 |
| b3350 | 1806 | 0,55 | 0,232831429 | 5,76836372 | 0,956413724 | 0,328092094 | 0,489843027 |
| b1336 | 1527 | 0,49 | 0,358048722 | 3,037354208 | 0,955006181 | 0,328448282 | 0,490211522 |
| b1412 | 606 | 0,52 | 0,287074812 | 4,944074638 | 0,950636287 | 0,329557389 | 0,491703135 |
| b2023 | 591 | 0,57 | 0,276893015 | 5,500493526 | 0,949121924 | 0,329942905 | 0,492114509 |
| b4193 | 1398 | 0,53 | -0,300982678 | 3,739383056 | 0,948421113 | 0,330121515 | 0,492217109 |
| b1834 | 2634 | 0,54 | -0,182264179 | 7,212262739 | 0,944881046 | 0,331025715 | 0,493245296 |
| b4399 | 1425 | 0,54 | 0,24930216 | 4,893447193 | 0,944859599 | 0,331031203 | 0,493245296 |
| b1536 | 393 | 0,48 | 0,318688082 | 5,71042251 | 0,943165374 | 0,331465116 | 0,4937277 |
| b3216 | 2382 | 0,46 | -0,269545236 | 4,886755725 | 0,941333531 | 0,331935127 | 0,494248041 |
| b0543 | 333 | 0,41 | 0,449586084 | 2,808758606 | 0,940944746 | 0,332034996 | 0,494248041 |
| b0705 | 1137 | 0,41 | 0,78434464 | 1,019693711 | 0,938315738 | 0,332711368 | 0,495090421 |
| b0131 | 381 | 0,52 | 0,269381489 | 6,482848381 | 0,9348569 | 0,333604035 | 0,496253994 |
| b0687 | 546 | 0,5 | 0,21983059 | 7,045354716 | 0,933819718 | 0,333872337 | 0,496488325 |
| b2623 | 957 | 0,37 | 0,386211037 | 3,391799169 | 0,932670071 | 0,334170069 | 0,496732038 |
| b0881 | 321 | 0,45 | -0,21468227 | 6,087217488 | 0,932331254 | 0,334257882 | 0,496732038 |
| b1917 | 753 | 0,54 | -0,219336864 | 5,747408062 | 0,930624557 | 0,334700687 | 0,496970853 |
| b1407 | 2307 | 0,43 | 0,416124017 | 2,931497047 | 0,93032205 | 0,334779254 | 0,496970853 |
| b2682 | 738 | 0,53 | 0,204617585 | 6,109188933 | 0,930099397 | 0,334837098 | 0,496970853 |
| b3935 | 2199 | 0,57 | 0,222031007 | 5,450360102 | 0,930003137 | 0,33486211 | 0,496970853 |
| b1843 | 657 | 0,52 | -0,205241911 | 5,543270829 | 0,928301237 | 0,335304737 | 0,497463036 |
| b3749 | 1506 | 0,51 | -0,295278098 | 7,092508339 | 0,924266627 | 0,336357184 | 0,49885933 |
| b0283 | 957 | 0,6 | -0,337864288 | 4,473273292 | 0,919290337 | 0,337661376 | 0,500627948 |
| b3227 | 1368 | 0,51 | 0,416039567 | 2,745838747 | 0,915975575 | 0,338533878 | 0,50172569 |
| b3191 | 294 | 0,53 | -0,229148919 | 6,169281752 | 0,915627548 | 0,338625661 | 0,50172569 |
| b2620 | 483 | 0,52 | -0,200147195 | 6,51637082 | 0,91501779 | 0,338786548 | 0,501798184 |
| b1083 | 954 | 0,51 | -0,276186742 | 4,169328605 | 0,914281755 | 0,33898089 | 0,501920168 |
| b4594 | 186 | 0,38 | 0,847200319 | 0,996738367 | 0,913521643 | 0,339181747 | 0,502051714 |
| b2012 | 228 | 0,5 | 0,407340572 | 2,901056953 | 0,911162348 | 0,339806202 | 0,502809969 |
| b2911 | 183 | 0,55 | 0,405536021 | 11,6235428 | 0,908755502 | 0,340444836 | 0,503588699 |
| b2160 | 1089 | 0,52 | -0,214999637 | 5,620788398 | 0,904037054 | 0,34170153 | 0,505280851 |
| b1483 | 927 | 0,55 | 0,353628498 | 4,7175645 | 0,901689106 | 0,342329203 | 0,506042048 |
| b4257 | 1197 | 0,37 | 0,721646649 | 1,556514852 | 0,900585165 | 0,342624855 | 0,506312101 |
| b4679 | 84 | 0,35 | 1,137056395 | 0,520473757 | 0,899293052 | 0,342971341 | 0,506588939 |
| b0549 | 291 | 0,53 | 1,129687742 | 0,572881136 | 0,899043743 | 0,343038248 | 0,506588939 |
| b1625 | 216 | 0,47 | 0,483473219 | 3,060565895 | 0,898264432 | 0,343247507 | 0,506731004 |
| b1508 | 267 | 0,45 | -0,340840367 | 3,99251547 | 0,897411033 | 0,343476858 | 0,50677897 |
| b0986 | 645 | 0,48 | 0,50629443 | 1,792996602 | 0,89730214 | 0,343506138 | 0,50677897 |

| | | | | | | | |
|---|---|---|---|---|---|---|---|
| b1539 | 747 | 0,51 | 0,197855974 | 8,093201092 | 0,896603869 | 0,343693974 | 0,506889237 |
| b0516 | 1236 | 0,51 | 0,322437521 | 3,415536538 | 0,896048689 | 0,343843417 | 0,506942827 |
| b2576 | 1335 | 0,55 | 0,167919427 | 6,484544044 | 0,895426328 | 0,344011048 | 0,507023189 |
| b1938 | 1659 | 0,55 | 0,59630716 | 1,80720272 | 0,894566474 | 0,344242829 | 0,507036715 |
| b2684 | 531 | 0,49 | 0,292883035 | 6,596399431 | 0,894552938 | 0,34424648 | 0,507036715 |
| b3744 | 993 | 0,56 | 0,258208334 | 6,329769829 | 0,89030438 | 0,345394837 | 0,508548696 |
| b4248 | 396 | 0,47 | 0,389171198 | 3,908337574 | 0,889916448 | 0,345499951 | 0,508548696 |
| b4170 | 1848 | 0,56 | -0,177017385 | 7,240621356 | 0,889074985 | 0,345728101 | 0,508693778 |
| b4249 | 714 | 0,54 | 0,39790363 | 2,764083582 | 0,888669527 | 0,345838108 | 0,508693778 |
| b4309 | 981 | 0,49 | 0,451479768 | 3,346265391 | 0,888297788 | 0,345939008 | 0,508693778 |
| b3203 | 288 | 0,45 | 0,176580858 | 8,117894668 | 0,885634422 | 0,346663086 | 0,509591326 |
| b1141 | 246 | 0,44 | 0,529824003 | 2,365321726 | 0,883665797 | 0,347199609 | 0,510098538 |
| b0325 | 1050 | 0,53 | -0,218605556 | 8,568704262 | 0,883298822 | 0,347299748 | 0,510098538 |
| b1732 | 2262 | 0,52 | -0,254578691 | 10,30895004 | 0,883116321 | 0,347349562 | 0,510098538 |
| b0490 | 678 | 0,45 | 0,218657164 | 5,012509358 | 0,881310485 | 0,347842999 | 0,510655854 |
| b4105 | 1017 | 0,55 | 0,792587306 | 1,451955332 | 0,879964143 | 0,348211501 | 0,511029452 |
| b1481 | 216 | 0,45 | 0,833853033 | 1,992861899 | 0,87900097 | 0,348475452 | 0,511249419 |
| b0580 | 249 | 0,51 | -0,340287898 | 3,960844343 | 0,878022738 | 0,348743808 | 0,511475703 |
| b4568 | 162 | 0,52 | -0,313017933 | 8,967608928 | 0,874402743 | 0,34973932 | 0,512527482 |
| b2197 | 480 | 0,54 | -0,437800155 | 4,681105095 | 0,87417862 | 0,349801081 | 0,512527482 |
| b1724 | 291 | 0,43 | 0,221985888 | 7,373194828 | 0,874168 | 0,349804008 | 0,512527482 |
| b1541 | 204 | 0,52 | -0,504894121 | 4,135699587 | 0,872444848 | 0,350279359 | 0,513056238 |
| b2848 | 483 | 0,32 | 0,860175548 | 1,018642382 | 0,871480268 | 0,350545834 | 0,513278806 |
| b4462 | 2253 | 0,37 | 0,585649159 | 1,427671372 | 0,86933131 | 0,351140497 | 0,513981616 |
| b2601 | 1071 | 0,52 | -0,278111742 | 5,952647607 | 0,863809692 | 0,352674768 | 0,516058867 |
| b1301 | 1281 | 0,58 | 0,354547479 | 6,751827631 | 0,86223892 | 0,353112903 | 0,516364142 |
| b3956 | 2652 | 0,56 | 0,217663197 | 9,052023789 | 0,862235673 | 0,35311381 | 0,516364142 |
| b4001 | 696 | 0,53 | 0,230944515 | 5,433450632 | 0,860839908 | 0,353503756 | 0,51662828 |
| b3589 | 1152 | 0,51 | 0,596638108 | 1,583560677 | 0,860764019 | 0,353524975 | 0,51662828 |
| b3877 | 1386 | 0,52 | 0,436139663 | 2,765055618 | 0,85507189 | 0,355121473 | 0,518792191 |
| b2301 | 645 | 0,52 | 0,20505292 | 5,48873259 | 0,853707824 | 0,355505524 | 0,519061998 |
| b1172 | 345 | 0,52 | 0,35936539 | 3,332214232 | 0,853593347 | 0,355537781 | 0,519061998 |
| b0193 | 825 | 0,49 | 0,35482225 | 3,086143049 | 0,853110493 | 0,355673881 | 0,51909161 |
| b0584 | 2241 | 0,54 | 0,399581555 | 4,482847378 | 0,850021196 | 0,356546342 | 0,520195541 |
| b0188 | 1299 | 0,58 | 0,211381711 | 5,919416395 | 0,848771548 | 0,356900093 | 0,520542212 |
| b2867 | 879 | 0,54 | -0,311546703 | 3,742130237 | 0,848181333 | 0,35706734 | 0,520591463 |
| b0081 | 459 | 0,52 | -0,167270366 | 7,331312505 | 0,847744676 | 0,357191142 | 0,520591463 |
| b0482 | 795 | 0,54 | -0,21789869 | 4,970850393 | 0,847423233 | 0,357282316 | 0,520591463 |
| b3546 | 1692 | 0,5 | -0,226195104 | 5,561688082 | 0,846146957 | 0,357644635 | 0,520950034 |
| b3608 | 1020 | 0,57 | 0,192002736 | 8,886401788 | 0,84241363 | 0,358707383 | 0,522328294 |
| b3563 | 342 | 0,42 | 0,787061423 | 1,148462004 | 0,841685514 | 0,358915157 | 0,522461103 |
| b0122 | 348 | 0,5 | -0,381736206 | 4,124611483 | 0,839739049 | 0,359471414 | 0,523100934 |
| b0618 | 1059 | 0,51 | -0,490852619 | 3,132708843 | 0,838062874 | 0,359951378 | 0,523629366 |
| b1295 | 246 | 0,48 | 0,477179008 | 2,794362667 | 0,837566602 | 0,360093652 | 0,523666369 |
| b2570 | 480 | 0,55 | 0,273117093 | 5,008135405 | 0,836278472 | 0,360463303 | 0,524033904 |
| b1887 | 504 | 0,52 | 0,384545448 | 2,589002659 | 0,835831383 | 0,360591725 | 0,52405062 |
| b1290 | 807 | 0,51 | -0,238041672 | 5,507309899 | 0,833795927 | 0,361177189 | 0,524731332 |
| b4181 | 402 | 0,39 | 1,021123824 | 0,932488963 | 0,832171591 | 0,361645342 | 0,525241225 |
| b1011 | 693 | 0,55 | 0,579496703 | 1,718557742 | 0,829146516 | 0,362519439 | 0,526065024 |
| b2646 | 330 | 0,48 | 0,57122599 | 1,984204591 | 0,829001066 | 0,36256154 | 0,526065024 |
| b4623 | 2597 | 0,51 | 0,368238327 | 3,384695771 | 0,828990249 | 0,362564672 | 0,526065024 |
| b4589 | 195 | 0,44 | -0,744334738 | 1,513305521 | 0,825417763 | 0,363600886 | 0,52739779 |
| b0512 | 1362 | 0,52 | -0,313755707 | 4,834626923 | 0,823791136 | 0,364074053 | 0,527913267 |
| b3716 | 468 | 0,44 | 0,409344002 | 2,524806191 | 0,822967025 | 0,364314103 | 0,527967906 |
| b3662 | 1191 | 0,53 | 0,441600748 | 2,381798669 | 0,822853014 | 0,36434733 | 0,527967906 |
| b4459 | 140 | 0,52 | -0,378007183 | 3,636000985 | 0,822039881 | 0,364584427 | 0,528140724 |
| b4525 | 138 | 0,41 | 0,668714789 | 1,403981781 | 0,821555705 | 0,364725707 | 0,528174674 |
| b3586 | 1137 | 0,51 | 0,364632354 | 2,913918411 | 0,819705424 | 0,365266307 | 0,528786689 |
| b0098 | 2706 | 0,52 | -0,198954451 | 9,495341106 | 0,817145141 | 0,366016184 | 0,529701174 |
| b4089 | 891 | 0,47 | -0,196403542 | 6,033652372 | 0,815074763 | 0,366624136 | 0,530409741 |
| b0084 | 1767 | 0,54 | -0,181522887 | 7,679260801 | 0,812285008 | 0,367445549 | 0,531426573 |
| b1351 | 276 | 0,39 | -0,933609376 | 0,957682519 | 0,81105407 | 0,367808798 | 0,531780333 |
| b2600 | 1122 | 0,52 | -0,247096796 | 6,019175923 | 0,805453086 | 0,369467971 | 0,533910647 |
| b4265 | 1320 | 0,51 | -0,279497854 | 4,219684611 | 0,805276372 | 0,369520488 | 0,533910647 |
| b0790 | 762 | 0,56 | -0,229688346 | 5,802460689 | 0,804604401 | 0,369720285 | 0,534027173 |
| b2271 | 972 | 0,45 | 0,548755997 | 1,632206815 | 0,802894266 | 0,370229438 | 0,534590316 |
| b1562 | 156 | 0,52 | -0,303265331 | 4,761754646 | 0,801542204 | 0,370632676 | 0,53496496 |
| b3327 | 1197 | 0,51 | -0,364779904 | 3,523611266 | 0,801224172 | 0,370727614 | 0,53496496 |
| b2919 | 786 | 0,49 | 0,452756071 | 2,135278146 | 0,799989066 | 0,371096639 | 0,535325116 |
| b1356 | 477 | 0,42 | 0,216877327 | 5,510991873 | 0,795843812 | 0,372338914 | 0,536944341 |
| b4442 | 78 | 0,41 | 0,879043221 | 0,622200346 | 0,794976112 | 0,372599687 | 0,537147571 |
| b0042 | 942 | 0,57 | 0,342553324 | 2,400592092 | 0,793548437 | 0,373029308 | 0,537550804 |
| b3322 | 420 | 0,46 | 0,337988976 | 2,767440146 | 0,793249789 | 0,373119266 | 0,537550804 |
| b1104 | 378 | 0,49 | 0,17668581 | 7,231827004 | 0,792455186 | 0,373358762 | 0,537722999 |
| b3907 | 1035 | 0,52 | 0,491746721 | 2,89143728 | 0,78912221 | 0,374365682 | 0,538999996 |
| b1265 | 45 | 0,44 | 1,194064458 | 0,410376926 | 0,787221508 | 0,374941605 | 0,539241738 |
| b4668 | 57 | 0,44 | 1,194064458 | 0,410376926 | 0,787221508 | 0,374941605 | 0,539241738 |
| b4678 | 63 | 0,44 | 1,194064458 | 0,410376926 | 0,787221508 | 0,374941605 | 0,539241738 |

| | | | | | | | |
|---|---|---|---|---|---|---|---|
| b3920 | 600 | 0,49 | 0,318185478 | 4,506890749 | 0,786980117 | 0,375014837 | 0,539241738 |
| b4427 | 109 | 0,39 | 1,197216516 | 0,421896389 | 0,78599119 | 0,375315061 | 0,539500354 |
| b2686 | 1539 | 0,57 | 0,192668985 | 5,27232457 | 0,782778192 | 0,376292814 | 0,540720272 |
| b4350 | 3513 | 0,54 | -0,201514908 | 9,286395957 | 0,782410261 | 0,376405009 | 0,540720272 |
| b1579 | 1158 | 0,42 | 0,404004891 | 2,846227733 | 0,779997834 | 0,377141802 | 0,541605113 |
| b1379 | 423 | 0,47 | 0,183898774 | 6,057941668 | 0,778073895 | 0,377730859 | 0,542277294 |
| b1063 | 561 | 0,47 | 0,194596322 | 5,313771527 | 0,773298184 | 0,379198663 | 0,544210184 |
| b1108 | 543 | 0,47 | -0,180714018 | 8,274307155 | 0,771931137 | 0,379620302 | 0,544640907 |
| b0269 | 1968 | 0,67 | 0,19684659 | 6,036892814 | 0,770603166 | 0,380030523 | 0,545054977 |
| b2048 | 1371 | 0,55 | 0,46488342 | 2,811811521 | 0,769967242 | 0,380227186 | 0,545162588 |
| b2559 | 504 | 0,55 | -0,24913417 | 4,578433534 | 0,768953611 | 0,380540956 | 0,54543798 |
| b3720 | 1617 | 0,45 | 0,596925282 | 2,076944608 | 0,766290392 | 0,381367101 | 0,546447362 |
| b0167 | 2673 | 0,54 | -0,158984013 | 6,938486184 | 0,764596065 | 0,381894012 | 0,547027473 |
| b0861 | 669 | 0,52 | 0,181687702 | 7,032702152 | 0,761558265 | 0,382841307 | 0,548079985 |
| b1438 | 417 | 0,46 | -0,25705874 | 4,091924178 | 0,761455641 | 0,382873367 | 0,548079985 |
| b3562 | 438 | 0,41 | 0,56489398 | 1,464852782 | 0,757817701 | 0,384012332 | 0,549534888 |
| b4212 | 381 | 0,56 | -0,313613013 | 3,985962669 | 0,755884152 | 0,384619644 | 0,550228294 |
| b2150 | 999 | 0,51 | 0,585139157 | 9,841401037 | 0,753290934 | 0,385436298 | 0,55122064 |
| b1517 | 876 | 0,5 | 0,470821036 | 7,11135962 | 0,75220522 | 0,385778942 | 0,551399497 |
| b2305 | 891 | 0,46 | -0,325250368 | 3,987405761 | 0,751928482 | 0,385866348 | 0,551399497 |
| b4097 | 759 | 0,61 | 0,577706392 | 1,533088968 | 0,75160542 | 0,385968421 | 0,551399497 |
| b0890 | 3990 | 0,54 | -0,190767574 | 9,139575598 | 0,751336353 | 0,386053463 | 0,551399497 |
| b2195 | 558 | 0,54 | -0,48680002 | 4,570117574 | 0,749895766 | 0,386509234 | 0,551736109 |
| b4578 | 432 | 0,46 | 0,336033847 | 3,725249444 | 0,749331635 | 0,386687922 | 0,551736109 |
| b3633 | 1278 | 0,54 | 0,187904829 | 6,020996813 | 0,748842869 | 0,386842833 | 0,551736109 |
| b4467 | 1224 | 0,56 | 0,756429863 | 9,062609332 | 0,748725634 | 0,386880003 | 0,551736109 |
| b4639 | 87 | 0,41 | 1,165065115 | 0,410376926 | 0,748259828 | 0,387027739 | 0,551736109 |
| b4670 | 54 | 0,41 | 1,165065115 | 0,410376926 | 0,748259828 | 0,387027739 | 0,551736109 |
| b2093 | 285 | 0,42 | -0,301290947 | 10,55208995 | 0,746687236 | 0,387527102 | 0,552154918 |
| b2917 | 2145 | 0,55 | -0,269913722 | 3,590421907 | 0,74629765 | 0,387650954 | 0,552154918 |
| b2021 | 1071 | 0,55 | -0,279699136 | 5,84524893 | 0,746171395 | 0,387691103 | 0,552154918 |
| b1180 | 660 | 0,52 | 0,199657034 | 6,157386423 | 0,744801814 | 0,388127014 | 0,552600152 |
| b4171 | 951 | 0,53 | -0,214877922 | 9,943046646 | 0,743426016 | 0,388565608 | 0,553048922 |
| b1981 | 1317 | 0,5 | 0,243582559 | 5,278160004 | 0,741770559 | 0,389094295 | 0,553514574 |
| b4698 | 98 | 0,42 | 1,15833189 | 0,488793819 | 0,741355511 | 0,389227005 | 0,553514574 |
| b1387 | 2046 | 0,54 | 0,511997932 | 3,872566733 | 0,741242157 | 0,389263261 | 0,553514574 |
| b0109 | 894 | 0,53 | 0,172640315 | 5,696186983 | 0,737516919 | 0,39045746 | 0,55503658 |
| b4310 | 1107 | 0,44 | 0,276281148 | 5,367827375 | 0,736900619 | 0,390655533 | 0,555142073 |
| b3555 | 291 | 0,44 | -0,216132731 | 8,713940535 | 0,736384012 | 0,390821676 | 0,55520214 |
| b2494 | 1464 | 0,54 | 0,163859157 | 7,159382956 | 0,734496016 | 0,391429725 | 0,555889743 |
| b1250 | 1254 | 0,42 | 0,216284567 | 5,699444969 | 0,733123085 | 0,391872744 | 0,556342617 |
| b3547 | 1209 | 0,51 | 0,288116866 | 3,392428632 | 0,730286963 | 0,392790188 | 0,55717845 |
| b4413 | 55 | 0,47 | 1,144823263 | 0,432982471 | 0,729767811 | 0,39295846 | 0,55717845 |
| b0075 | 87 | 0,47 | 1,14453146 | 0,421833148 | 0,729767391 | 0,392958597 | 0,55717845 |
| b4545 | 51 | 0,47 | 1,144823305 | 0,410376926 | 0,729766943 | 0,392958742 | 0,55717845 |
| b4312 | 603 | 0,43 | 0,625626996 | 4,870884771 | 0,729256336 | 0,393124345 | 0,557236975 |
| b2578 | 588 | 0,51 | -0,289010433 | 3,221994596 | 0,728311006 | 0,393431205 | 0,557355269 |
| b4526 | 171 | 0,37 | -0,905519728 | 0,612157427 | 0,728233104 | 0,393456509 | 0,557355269 |
| b0136 | 597 | 0,41 | 0,465745784 | 1,786394362 | 0,724257223 | 0,394751004 | 0,559004089 |
| b2108 | 1035 | 0,39 | 0,669831096 | 1,142345105 | 0,723892951 | 0,394869912 | 0,559004089 |
| b2758 | 1092 | 0,43 | 0,325376687 | 3,874490524 | 0,721884952 | 0,395526306 | 0,559756521 |
| b1271 | 759 | 0,52 | 0,190379194 | 5,547549665 | 0,720330958 | 0,39603537 | 0,560300041 |
| b4431 | 106 | 0,39 | 1,141066147 | 0,410376926 | 0,717623349 | 0,396924602 | 0,561380898 |
| b1008 | 591 | 0,57 | 0,554506662 | 1,480471895 | 0,716935297 | 0,397151031 | 0,56152395 |
| b2100 | 966 | 0,57 | 0,248907658 | 4,034231189 | 0,716440941 | 0,397313832 | 0,561576978 |
| b2804 | 423 | 0,49 | -0,20892276 | 5,652618414 | 0,715826771 | 0,397516225 | 0,561685914 |
| b1372 | 3363 | 0,53 | -0,294930641 | 4,562919421 | 0,712473738 | 0,398623812 | 0,563073409 |
| b0378 | 1095 | 0,53 | 0,185619393 | 5,871594578 | 0,710955122 | 0,399126917 | 0,56360644 |
| b3053 | 2841 | 0,56 | -0,20804284 | 8,39369776 | 0,705365566 | 0,400986638 | 0,565909903 |
| b0417 | 978 | 0,54 | 0,151685642 | 6,174089895 | 0,705293563 | 0,401010677 | 0,565909903 |
| b3718 | 723 | 0,5 | 0,277433762 | 3,170273829 | 0,704081318 | 0,401415701 | 0,56630317 |
| b2763 | 1713 | 0,56 | 0,299280485 | 3,613204055 | 0,702629885 | 0,401901423 | 0,56681 |
| b0767 | 996 | 0,55 | -0,174910725 | 9,059259394 | 0,702229198 | 0,402035664 | 0,566820964 |
| b1096 | 810 | 0,51 | -0,178615191 | 5,325970087 | 0,70177275 | 0,402188665 | 0,566858364 |
| b3929 | 486 | 0,52 | -0,213869347 | 8,720308825 | 0,699504842 | 0,402950124 | 0,567753052 |
| b0504 | 927 | 0,52 | -0,32914999 | 2,758287266 | 0,698622127 | 0,403247067 | 0,567989344 |
| b0356 | 1110 | 0,54 | -0,17095708 | 7,184890981 | 0,697456653 | 0,403639618 | 0,567989344 |
| b1811 | 180 | 0,56 | 0,311155271 | 4,067180486 | 0,696487802 | 0,403966366 | 0,567989344 |
| b4438 | 87 | 0,43 | 1,119697699 | 0,433445674 | 0,696374455 | 0,404004618 | 0,567989344 |
| b4675 | 75 | 0,43 | 1,119612439 | 0,421858274 | 0,696374021 | 0,404004764 | 0,567989344 |
| b2018 | 51 | 0,43 | 1,119612442 | 0,410376926 | 0,696373576 | 0,404004915 | 0,567989344 |
| b4601 | 84 | 0,43 | 1,119612442 | 0,410376926 | 0,696373576 | 0,404004915 | 0,567989344 |
| b1317 | 660 | 0,57 | 0,530892254 | 1,891627327 | 0,695760204 | 0,404212005 | 0,568102291 |
| b4561 | 267 | 0,46 | 0,453944428 | 1,878583746 | 0,694988168 | 0,404472884 | 0,568290742 |
| b4295 | 987 | 0,5 | -0,182929036 | 7,600002843 | 0,692078649 | 0,405458254 | 0,569496676 |
| b4505 | 249 | 0,47 | 0,227843651 | 3,914008471 | 0,688140966 | 0,406797428 | 0,571198644 |
| b1430 | 594 | 0,46 | 0,210593968 | 5,33038084 | 0,686985218 | 0,407191717 | 0,571573215 |
| b4613 | 129 | 0,47 | -0,365937756 | 3,536409403 | 0,68509157 | 0,407838954 | 0,572302503 |

| | | | | | | | |
|---|---|---|---|---|---|---|---|
| b0603 | 903 | 0,39 | -0,426200032 | 1,907227249 | 0,68363239 | 0,408338723 | 0,572824462 |
| b2331 | 552 | 0,53 | -0,188156201 | 5,687060354 | 0,680913336 | 0,409272397 | 0,573954595 |
| b3524 | 2061 | 0,53 | -0,209924348 | 8,112125853 | 0,679023286 | 0,409923255 | 0,574563587 |
| b4016 | 1737 | 0,53 | 0,271194887 | 10,81141346 | 0,678907892 | 0,409963041 | 0,574563587 |
| b4667 | 60 | 0,4 | 1,106078911 | 0,410376926 | 0,677213198 | 0,410548004 | 0,575203549 |
| b3666 | 1392 | 0,53 | 0,443920081 | 2,8754814 | 0,67243712 | 0,412203204 | 0,577342113 |
| b4608 | 77 | 0,42 | 1,099375956 | 0,410376926 | 0,671646237 | 0,412478242 | 0,577546855 |
| b3864 | 109 | 0,42 | 0,774013544 | 0,728032521 | 0,670858424 | 0,412752481 | 0,57775035 |
| b1657 | 1170 | 0,53 | 0,30500979 | 3,497134308 | 0,668490867 | 0,413578255 | 0,578725488 |
| b1854 | 1443 | 0,53 | -0,232216442 | 9,928270337 | 0,667123348 | 0,41405634 | 0,579126071 |
| b0383 | 1416 | 0,53 | -0,208876312 | 5,62749173 | 0,666933006 | 0,414122949 | 0,579126071 |
| b1635 | 606 | 0,5 | 0,187980874 | 7,973063145 | 0,666019082 | 0,414442989 | 0,579260614 |
| b1552 | 213 | 0,4 | 0,798749963 | 0,918635112 | 0,665920188 | 0,414477642 | 0,579260614 |
| b1151 | 412 | 0,56 | 0,661180541 | 1,326769129 | 0,665332373 | 0,414683703 | 0,579367942 |
| b3696 | 657 | 0,43 | 0,330468101 | 3,545278124 | 0,664253987 | 0,415062131 | 0,579715946 |
| b1918 | 669 | 0,53 | -0,186308631 | 5,480953548 | 0,663148443 | 0,41545062 | 0,579928732 |
| b1350 | 2601 | 0,49 | -0,244308076 | 4,105866488 | 0,663084059 | 0,415473261 | 0,579928732 |
| b1494 | 2796 | 0,47 | 0,26824509 | 4,215851698 | 0,662466766 | 0,415690431 | 0,580051217 |
| b4216 | 555 | 0,52 | 0,161548794 | 6,777902978 | 0,662018212 | 0,415848342 | 0,580090964 |
| b3027 | 333 | 0,34 | 0,969234372 | 0,680958034 | 0,660134737 | 0,41651238 | 0,580836493 |
| b0943 | 516 | 0,47 | 0,453763854 | 2,197620545 | 0,65902803 | 0,416903295 | 0,581200799 |
| b1585 | 711 | 0,44 | 0,184672669 | 5,890379487 | 0,657681985 | 0,417379484 | 0,581683722 |
| b0729 | 870 | 0,54 | 0,328511759 | 11,38589116 | 0,656346511 | 0,417852733 | 0,582162247 |
| b3834 | 606 | 0,56 | -0,147410911 | 6,300104403 | 0,654798699 | 0,418402226 | 0,582746668 |
| b2722 | 924 | 0,58 | 0,480535508 | 1,457705854 | 0,653756507 | 0,418772823 | 0,583081638 |
| b1556 | 216 | 0,46 | 0,787811437 | 0,652617069 | 0,65087765 | 0,419799072 | 0,584224996 |
| b0788 | 957 | 0,52 | -0,268227497 | 5,133270225 | 0,650721932 | 0,419854688 | 0,584224996 |
| b2341 | 2145 | 0,52 | 0,188452334 | 7,767254864 | 0,650076061 | 0,420085487 | 0,584364728 |
| b3750 | 966 | 0,53 | 0,181499146 | 7,140652679 | 0,648716821 | 0,420571823 | 0,584717873 |
| b0982 | 447 | 0,52 | 0,327367243 | 2,863130924 | 0,648637382 | 0,420600273 | 0,584717873 |
| b1153 | 585 | 0,57 | 0,708110912 | 1,098316828 | 0,647688849 | 0,42094019 | 0,585008971 |
| b0083 | 366 | 0,5 | -0,174583786 | 5,854958865 | 0,646343248 | 0,421423106 | 0,585498562 |
| b0342 | 612 | 0,43 | 0,414733987 | 2,21088617 | 0,645153553 | 0,421850759 | 0,585911094 |
| b2388 | 966 | 0,52 | 0,146451799 | 7,414722897 | 0,642472081 | 0,422817036 | 0,587024949 |
| b2340 | 486 | 0,53 | -0,196128211 | 6,899404711 | 0,642201645 | 0,422914672 | 0,587024949 |
| b2446 | 417 | 0,49 | 0,477813812 | 2,392345175 | 0,641312207 | 0,423236027 | 0,587212528 |
| b3904 | 1470 | 0,55 | 0,48525537 | 1,910439629 | 0,641102515 | 0,423311842 | 0,587212528 |
| b2711 | 1134 | 0,54 | 0,337620088 | 2,788219792 | 0,63908822 | 0,424041159 | 0,588025132 |
| b3830 | 816 | 0,55 | -0,205982635 | 7,661179406 | 0,638760404 | 0,424160029 | 0,588025132 |
| b3345 | 387 | 0,55 | 0,171144148 | 5,412648249 | 0,638276 | 0,424335773 | 0,588086869 |
| b4346 | 1380 | 0,4 | 0,191859372 | 5,176748105 | 0,637068216 | 0,424774437 | 0,588415239 |
| b3648 | 624 | 0,49 | 0,137089283 | 7,22708058 | 0,636900851 | 0,424835277 | 0,588415239 |
| b3976 | 76 | 0,51 | 0,886137402 | 0,7277354 | 0,631437542 | 0,426828504 | 0,590993313 |
| b4129 | 1518 | 0,47 | 0,21626727 | 10,45267457 | 0,630199778 | 0,427282044 | 0,591438579 |
| b1255 | 744 | 0,49 | 0,20850708 | 5,431279177 | 0,628901568 | 0,427758512 | 0,591915298 |
| b2054 | 549 | 0,48 | 0,876324701 | 0,709595694 | 0,626673437 | 0,428578151 | 0,592866442 |
| b0351 | 951 | 0,54 | 0,270188416 | 3,129156541 | 0,62503802 | 0,429181264 | 0,593517564 |
| b4099 | 1065 | 0,63 | 0,477137103 | 1,366633107 | 0,621856664 | 0,430358174 | 0,594947924 |
| b2956 | 1008 | 0,45 | -0,394444175 | 2,591352162 | 0,62152524 | 0,430481061 | 0,594947924 |
| b2909 | 579 | 0,51 | 0,139638922 | 7,665449814 | 0,620348804 | 0,430917698 | 0,595367793 |
| b3878 | 2037 | 0,54 | 0,39501775 | 2,94257946 | 0,619439863 | 0,431255512 | 0,595650911 |
| b1697 | 765 | 0,51 | -0,439280272 | 2,103022769 | 0,618924779 | 0,431447126 | 0,595731983 |
| b1516 | 1023 | 0,5 | 0,407301788 | 7,308881906 | 0,61819956 | 0,431717129 | 0,59592121 |
| b0046 | 531 | 0,55 | 0,251994474 | 4,309528486 | 0,617484247 | 0,431983695 | 0,596105579 |
| b3809 | 825 | 0,55 | 0,157736354 | 7,235324776 | 0,614938354 | 0,432934468 | 0,59681333 |
| b4704 | 69 | 0,39 | 1,05161739 | 0,433172695 | 0,614330122 | 0,433162085 | 0,59681333 |
| b4630 | 90 | 0,39 | 1,051052695 | 0,421875775 | 0,614329746 | 0,433162226 | 0,59681333 |
| b4663 | 87 | 0,39 | 1,051437126 | 0,410376926 | 0,614329351 | 0,433162374 | 0,59681333 |
| b4680 | 72 | 0,39 | 1,051437126 | 0,410376926 | 0,614329351 | 0,433162374 | 0,59681333 |
| b2462 | 336 | 0,51 | 0,445790693 | 1,801662573 | 0,612863928 | 0,433711524 | 0,597386309 |
| b0988 | 504 | 0,55 | 0,257863412 | 4,24339363 | 0,610967069 | 0,434423924 | 0,598166317 |
| b1572 | 129 | 0,47 | 0,870350532 | 0,894911129 | 0,610645847 | 0,434544741 | 0,598166317 |
| b0341 | 1155 | 0,59 | -0,297969539 | 2,804686204 | 0,608770887 | 0,435250969 | 0,598954511 |
| b4076 | 597 | 0,52 | 0,424688369 | 2,138633319 | 0,602492706 | 0,437628523 | 0,602041449 |
| b0651 | 936 | 0,51 | 0,198044364 | 7,407786331 | 0,599531452 | 0,438756854 | 0,603122042 |
| b4298 | 906 | 0,53 | -0,177763264 | 4,438080146 | 0,599393817 | 0,438809406 | 0,603122042 |
| b1109 | 1305 | 0,53 | -0,229138397 | 5,850822557 | 0,599364293 | 0,43882068 | 0,603122042 |
| b2261 | 963 | 0,59 | -0,176994103 | 7,659720686 | 0,598585931 | 0,439118067 | 0,603122042 |
| b4599 | 96 | 0,39 | 0,353030032 | 3,948996488 | 0,598466602 | 0,439163686 | 0,603122042 |
| b3642 | 642 | 0,53 | -0,315583128 | 2,618571122 | 0,598171394 | 0,439276574 | 0,603122042 |
| b2332 | 822 | 0,45 | -0,182183782 | 5,87737814 | 0,597963833 | 0,439355972 | 0,603122042 |
| b2463 | 2280 | 0,54 | 0,228132513 | 10,20183416 | 0,595213584 | 0,44041011 | 0,604383991 |
| b2466 | 1044 | 0,55 | -0,279918935 | 4,445879475 | 0,594100561 | 0,440837823 | 0,604641951 |
| b0291 | 2526 | 0,55 | 0,22497639 | 4,173091559 | 0,593705486 | 0,440989797 | 0,604641951 |
| b0712 | 933 | 0,56 | -0,228113085 | 7,962423668 | 0,593671699 | 0,441002797 | 0,604641951 |
| b3698 | 399 | 0,51 | -0,202343409 | 5,343805534 | 0,591999941 | 0,441646794 | 0,605337956 |
| b2748 | 312 | 0,52 | 0,174449108 | 5,759116359 | 0,591653177 | 0,441780556 | 0,605337956 |
| b0537 | 1164 | 0,47 | -0,147869247 | 6,110970348 | 0,589951779 | 0,442437766 | 0,606053199 |

| | | | | | | | |
|---|---|---|---|---|---|---|---|
| b1489 | 2400 | 0,5 | 0,262853775 | 5,521021962 | 0,58854187 | 0,442983523 | 0,606615384 |
| b0940 | 2601 | 0,48 | -0,265349946 | 3,645030944 | 0,587644253 | 0,44333152 | 0,606906497 |
| b2977 | 405 | 0,55 | -0,616008934 | 9,850468809 | 0,584766896 | 0,444449892 | 0,608204509 |
| b4144 | 354 | 0,55 | 0,168114702 | 7,4662727 | 0,584507094 | 0,444551087 | 0,608204509 |
| b1885 | 1602 | 0,54 | 0,485509936 | 2,040057454 | 0,581549925 | 0,44570544 | 0,609597737 |
| b1515 | 993 | 0,53 | -0,373811982 | 5,688206677 | 0,579534807 | 0,44649472 | 0,61049095 |
| b1670 | 786 | 0,49 | -0,268818188 | 3,586127258 | 0,578631735 | 0,446849137 | 0,610789214 |
| b3025 | 660 | 0,52 | -0,202539986 | 4,084116633 | 0,577630409 | 0,447242627 | 0,611140687 |
| b2849 | 426 | 0,33 | 0,69699746 | 0,689733074 | 0,573363276 | 0,448925527 | 0,61317433 |
| b1609 | 1302 | 0,51 | 0,180169381 | 4,782426098 | 0,573163655 | 0,449004496 | 0,61317433 |
| b0991 | 231 | 0,42 | 0,842009526 | 0,753379523 | 0,571898049 | 0,449505666 | 0,613671763 |
| b1368 | 933 | 0,46 | -0,292177033 | 3,408512001 | 0,570374917 | 0,450109973 | 0,614217905 |
| b2484 | 1440 | 0,55 | 0,43420213 | 2,133732962 | 0,570199151 | 0,45017979 | 0,614217905 |
| b1570 | 408 | 0,44 | -0,191549671 | 5,444693565 | 0,568486415 | 0,450861002 | 0,61488806 |
| b0190 | 546 | 0,52 | -0,177976529 | 5,13363319 | 0,568274628 | 0,450945349 | 0,61488806 |
| b1272 | 1050 | 0,51 | 0,136486943 | 7,22997387 | 0,563663013 | 0,452788113 | 0,617212993 |
| b1932 | 480 | 0,47 | 0,238991605 | 3,504472482 | 0,563076415 | 0,453023357 | 0,617345906 |
| b2324 | 2007 | 0,53 | 0,1565343 | 5,915788659 | 0,561918325 | 0,453488351 | 0,617791729 |
| b1924 | 1407 | 0,52 | 0,372977889 | 2,35462327 | 0,560620837 | 0,454010206 | 0,618202506 |
| b0586 | 3882 | 0,57 | 0,340868053 | 4,730377911 | 0,560482899 | 0,45406574 | 0,618202506 |
| b0456 | 354 | 0,49 | 0,28339415 | 5,125799362 | 0,559801142 | 0,454340377 | 0,618388572 |
| b4606 | 60 | 0,38 | 0,999909005 | 0,487890955 | 0,558618854 | 0,454817263 | 0,618475407 |
| b2598 | 48 | 0,38 | 1,000709988 | 0,410376926 | 0,55861666 | 0,454818149 | 0,618475407 |
| b4666 | 60 | 0,38 | 1,000709988 | 0,410376926 | 0,55861666 | 0,454818149 | 0,618475407 |
| b3292 | 426 | 0,49 | 0,183449375 | 5,531751322 | 0,555090209 | 0,456245264 | 0,620227866 |
| b3503 | 426 | 0,52 | 0,335240276 | 2,934637637 | 0,552943538 | 0,457117454 | 0,621225115 |
| b1949 | 270 | 0,5 | 0,652260785 | 0,849926179 | 0,551432351 | 0,457733026 | 0,62187312 |
| b1643 | 237 | 0,49 | 0,245046779 | 3,586049367 | 0,550839158 | 0,457975017 | 0,622013342 |
| b3537 | 192 | 0,48 | -0,262403352 | 3,558576254 | 0,54999139 | 0,458321212 | 0,622294963 |
| b2001 | 1533 | 0,5 | -0,286445534 | 4,54613417 | 0,547232602 | 0,459450661 | 0,623639571 |
| b2604 | 1227 | 0,48 | -0,182088477 | 4,552406115 | 0,54123225 | 0,46192249 | 0,626804905 |
| b0845 | 1209 | 0,55 | -0,193600129 | 4,562216357 | 0,540297004 | 0,462309664 | 0,62714041 |
| b0132 | 903 | 0,49 | -0,20306694 | 4,718018456 | 0,539925745 | 0,462463502 | 0,627159278 |
| b0544 | 1527 | 0,36 | 0,43482836 | 1,755127978 | 0,536489941 | 0,46389106 | 0,62875474 |
| b3202 | 1434 | 0,53 | 0,155518775 | 8,907637378 | 0,536419132 | 0,463920555 | 0,62875474 |
| b3454 | 714 | 0,57 | 0,292186278 | 2,774786524 | 0,534171038 | 0,464858528 | 0,629835527 |
| b1159 | 834 | 0,38 | 0,351816749 | 2,491472426 | 0,53347529 | 0,465149429 | 0,630039208 |
| b4358 | 1023 | 0,51 | 0,367482889 | 2,073145565 | 0,531007699 | 0,46618351 | 0,631249091 |
| b2361 | 537 | 0,46 | 0,68206699 | 0,850711241 | 0,529297229 | 0,466902469 | 0,632031672 |
| b3583 | 696 | 0,57 | -0,262516559 | 3,028572464 | 0,527236455 | 0,467771034 | 0,632980513 |
| b3442 | 1179 | 0,34 | 0,860791973 | 0,87587061 | 0,526964469 | 0,467885863 | 0,632980513 |
| b1219 | 354 | 0,52 | -0,143027543 | 6,867765481 | 0,526555804 | 0,468058481 | 0,633022967 |
| b3037 | 672 | 0,56 | 0,141221446 | 8,000898767 | 0,525576649 | 0,468472488 | 0,633257715 |
| b0503 | 1095 | 0,56 | 0,216359598 | 4,257089902 | 0,525477052 | 0,468514633 | 0,633257715 |
| b4353 | 204 | 0,5 | 0,226024835 | 3,423214462 | 0,524384198 | 0,468977476 | 0,633692206 |
| b2002 | 447 | 0,58 | 0,329006448 | 2,703514405 | 0,521720113 | 0,470108852 | 0,634900217 |
| b2430 | 1305 | 0,52 | -0,239789817 | 4,919194395 | 0,521612132 | 0,470154801 | 0,634900217 |
| b1326 | 729 | 0,55 | -0,159052983 | 6,605729065 | 0,516682207 | 0,4722604 | 0,63755154 |
| b2334 | 489 | 0,47 | 0,411228113 | 1,617765144 | 0,516102336 | 0,472509067 | 0,637695163 |
| b2976 | 2172 | 0,53 | -0,548643242 | 12,67856685 | 0,514522395 | 0,473187672 | 0,638418767 |
| b3444 | 276 | 0,53 | -0,232989226 | 3,891240537 | 0,513493711 | 0,473630354 | 0,638823727 |
| b1879 | 2079 | 0,55 | 0,236888249 | 4,017846983 | 0,510814402 | 0,47478652 | 0,640190488 |
| b2753 | 1038 | 0,49 | 0,198626412 | 4,305752783 | 0,509781444 | 0,475233483 | 0,640459163 |
| b1107 | 1026 | 0,53 | -0,127016379 | 7,1555178 | 0,509693495 | 0,47527157 | 0,640459163 |
| b1143 | 342 | 0,39 | 0,19603972 | 4,327796331 | 0,509139841 | 0,47551145 | 0,640589816 |
| b3625 | 699 | 0,35 | 0,167670241 | 4,93024121 | 0,50386206 | 0,477808064 | 0,643490308 |
| b4652 | 199 | 0,42 | -0,660861444 | 1,509241466 | 0,502175776 | 0,478545662 | 0,644290074 |
| b0657 | 1539 | 0,56 | 0,163134111 | 6,249079089 | 0,501243418 | 0,478954284 | 0,644646577 |
| b2013 | 1059 | 0,5 | 0,286838171 | 2,887734064 | 0,499945423 | 0,479524104 | 0,645070685 |
| b4045 | 210 | 0,42 | -0,223641068 | 10,04243795 | 0,499870034 | 0,479557234 | 0,645070685 |
| b1948 | 738 | 0,53 | 0,484527255 | 1,280519542 | 0,499060242 | 0,479913335 | 0,645356006 |
| b0252 | 822 | 0,58 | -0,303780295 | 2,840202761 | 0,497188504 | 0,480738081 | 0,646271169 |
| b3343 | 288 | 0,5 | 0,257111968 | 4,143715178 | 0,496819563 | 0,480900922 | 0,646296231 |
| b2453 | 1188 | 0,59 | -0,286403566 | 2,985779659 | 0,493246273 | 0,482482779 | 0,648227763 |
| b0357 | 276 | 0,53 | 0,174889298 | 4,782113083 | 0,491049526 | 0,483459508 | 0,649345375 |
| b3054 | 1302 | 0,55 | 0,122092181 | 7,067136152 | 0,485100117 | 0,48612121 | 0,652625019 |
| b3683 | 1617 | 0,52 | 0,275442797 | 2,661683283 | 0,484566588 | 0,486361089 | 0,652625019 |
| b1118 | 1245 | 0,52 | -0,125776963 | 7,030644018 | 0,484381197 | 0,486444488 | 0,652625019 |
| b2077 | 1416 | 0,56 | -0,234115105 | 4,231356152 | 0,484293928 | 0,486483754 | 0,652625019 |
| b2369 | 615 | 0,4 | 0,263378458 | 6,161453147 | 0,482454265 | 0,487312734 | 0,653541494 |
| b1664 | 1257 | 0,51 | 0,145226708 | 9,206310189 | 0,481274871 | 0,487845419 | 0,653962979 |
| b3213 | 1419 | 0,56 | -0,36406384 | 7,178178867 | 0,481112501 | 0,487918831 | 0,653962979 |
| b4424 | 68 | 0,49 | 0,917530364 | 0,421994664 | 0,479111198 | 0,488825183 | 0,654786156 |
| b2348 | 75 | 0,49 | 0,917530364 | 0,421875775 | 0,479111195 | 0,488825185 | 0,654786156 |
| b4433 | 121 | 0,46 | 0,916300196 | 0,410376926 | 0,47716574 | 0,489708933 | 0,655676822 |
| b1386 | 2274 | 0,51 | -0,257131103 | 3,741996276 | 0,476559738 | 0,48998476 | 0,655676822 |
| b2610 | 1362 | 0,54 | -0,159214712 | 8,379753678 | 0,476468924 | 0,490026118 | 0,655676822 |
| b0130 | 1230 | 0,5 | -0,209205718 | 4,726754896 | 0,476361013 | 0,490075268 | 0,655676822 |

| | | | | | | | |
|---|---|---|---|---|---|---|---|
| b2821 | 2889 | 0,51 | -0,134610693 | 6,890385824 | 0,475454972 | 0,490488273 | 0,656033553 |
| b3376 | 1086 | 0,57 | -0,237207394 | 3,821808935 | 0,472541117 | 0,491820453 | 0,657492589 |
| b3937 | 609 | 0,52 | 0,265380257 | 3,905380574 | 0,471999946 | 0,492068536 | 0,657492589 |
| b2946 | 732 | 0,53 | 0,15818829 | 5,693342294 | 0,471827187 | 0,492147776 | 0,657492589 |
| b3753 | 993 | 0,51 | -0,161143665 | 5,672448193 | 0,471787645 | 0,492165916 | 0,657492589 |
| b0339 | 660 | 0,57 | 0,295342907 | 2,498822572 | 0,471313672 | 0,492383438 | 0,657587178 |
| b4517 | 174 | 0,4 | -0,313191421 | 3,322568024 | 0,470654572 | 0,492686189 | 0,657795502 |
| b0703 | 1521 | 0,55 | -0,404350638 | 2,196876676 | 0,470291102 | 0,492853279 | 0,657822631 |
| b2019 | 900 | 0,54 | 0,193396254 | 5,317617981 | 0,469554112 | 0,493192369 | 0,65807925 |
| b0756 | 1041 | 0,56 | 0,153143738 | 9,011989665 | 0,468381905 | 0,493732511 | 0,658348373 |
| b2950 | 981 | 0,55 | 0,254775496 | 3,206635983 | 0,468281559 | 0,493778796 | 0,658348373 |
| b4669 | 51 | 0,37 | 0,911208861 | 0,421787673 | 0,466873077 | 0,494429228 | 0,658348373 |
| b4585 | 84 | 0,37 | 0,911208862 | 0,410376926 | 0,46687277 | 0,494429369 | 0,658348373 |
| b4614 | 30 | 0,37 | 0,911208862 | 0,410376926 | 0,46687277 | 0,494429369 | 0,658348373 |
| b4664 | 60 | 0,37 | 0,911208862 | 0,410376926 | 0,46687277 | 0,494429369 | 0,658348373 |
| b4687 | 81 | 0,37 | 0,911208862 | 0,410376926 | 0,46687277 | 0,494429369 | 0,658348373 |
| b2820 | 3543 | 0,55 | -0,126335124 | 7,340706031 | 0,466570468 | 0,494569159 | 0,658348373 |
| b0601 | 630 | 0,47 | 0,238831997 | 3,541099185 | 0,466158538 | 0,494759749 | 0,658406531 |
| b1741 | 888 | 0,52 | 0,220799279 | 4,108947047 | 0,464770642 | 0,495402807 | 0,659066601 |
| b4191 | 756 | 0,51 | 0,189971136 | 4,82113624 | 0,463496238 | 0,495994519 | 0,65949781 |
| b1313 | 1053 | 0,56 | -0,294965695 | 2,890606462 | 0,463438787 | 0,496021222 | 0,65949781 |
| b0933 | 768 | 0,56 | 0,214608293 | 3,612917171 | 0,462017525 | 0,496682587 | 0,660181303 |
| b1723 | 930 | 0,54 | 0,139097582 | 8,414399909 | 0,461064019 | 0,497127122 | 0,660576271 |
| b1082 | 1644 | 0,52 | -0,220192633 | 3,911358138 | 0,460277265 | 0,497494421 | 0,660868405 |
| b2085 | 318 | 0,36 | -0,378571771 | 2,206344639 | 0,457773209 | 0,49866651 | 0,662040586 |
| b3569 | 1179 | 0,49 | -0,162223591 | 4,885106633 | 0,457513132 | 0,498788514 | 0,662040586 |
| b2237 | 651 | 0,49 | 0,15619175 | 5,468544185 | 0,457082527 | 0,498990624 | 0,662040586 |
| b4699 | 122 | 0,44 | 0,720450453 | 0,939721141 | 0,456387649 | 0,499317067 | 0,662040586 |
| b2738 | 639 | 0,54 | -0,264868845 | 3,016099478 | 0,456121638 | 0,499442131 | 0,662040586 |
| b0380 | 213 | 0,49 | 0,169194902 | 6,067559957 | 0,4558305 | 0,499579069 | 0,662040586 |
| b0038 | 1218 | 0,53 | -0,204542464 | 3,744871777 | 0,455811736 | 0,499587897 | 0,662040586 |
| b0868 | 1014 | 0,54 | -0,148615498 | 5,853796647 | 0,455618362 | 0,499678889 | 0,662040586 |
| b1825 | 288 | 0,43 | -0,186496635 | 5,061085899 | 0,455352492 | 0,49980404 | 0,662040586 |
| b2755 | 918 | 0,51 | -0,212500008 | 4,134720489 | 0,455246524 | 0,499853936 | 0,662040586 |
| b4548 | 201 | 0,55 | 0,542937393 | 0,900747212 | 0,453115509 | 0,50085915 | 0,663175985 |
| b4374 | 678 | 0,54 | 0,145742142 | 6,030210956 | 0,449806175 | 0,502427013 | 0,665055485 |
| b3722 | 1878 | 0,51 | 0,233933569 | 4,358148099 | 0,447809699 | 0,503376933 | 0,66597967 |
| b2866 | 2259 | 0,53 | -0,152149556 | 5,221229911 | 0,446190594 | 0,504149554 | 0,66597967 |
| b4702 | 54 | 0,5 | 0,882630277 | 0,443827977 | 0,444295455 | 0,505056475 | 0,66597967 |
| b0743 | 76 | 0,5 | 0,882517253 | 0,410376926 | 0,444294628 | 0,505056872 | 0,66597967 |
| b0745 | 76 | 0,5 | 0,882517253 | 0,410376926 | 0,444294628 | 0,505056872 | 0,66597967 |
| b0747 | 76 | 0,5 | 0,882517253 | 0,410376926 | 0,444294628 | 0,505056872 | 0,66597967 |
| b0748 | 76 | 0,5 | 0,882517253 | 0,410376926 | 0,444294628 | 0,505056872 | 0,66597967 |
| b0749 | 76 | 0,5 | 0,882517253 | 0,410376926 | 0,444294628 | 0,505056872 | 0,66597967 |
| b2404 | 76 | 0,5 | 0,882517253 | 0,410376926 | 0,444294628 | 0,505056872 | 0,66597967 |
| b4422 | 66 | 0,5 | 0,882517253 | 0,410376926 | 0,444294628 | 0,505056872 | 0,66597967 |
| b4444 | 88 | 0,5 | 0,882517253 | 0,410376926 | 0,444294628 | 0,505056872 | 0,66597967 |
| b4597 | 64 | 0,5 | 0,882517253 | 0,410376926 | 0,444294628 | 0,505056872 | 0,66597967 |
| b4706 | 66 | 0,5 | 0,882517253 | 0,410376926 | 0,444294628 | 0,505056872 | 0,66597967 |
| b1763 | 1962 | 0,55 | -0,117683766 | 7,525716042 | 0,443830099 | 0,505279599 | 0,666077401 |
| b2918 | 996 | 0,54 | 0,318115425 | 2,450913681 | 0,443381856 | 0,505494678 | 0,666164995 |
| b3214 | 765 | 0,45 | 0,286989161 | 2,647565498 | 0,443027554 | 0,505664792 | 0,666193298 |
| b1750 | 711 | 0,48 | 0,235863368 | 3,882622609 | 0,440020919 | 0,507112359 | 0,667745355 |
| b2921 | 897 | 0,45 | 0,212388936 | 3,229930279 | 0,43977952 | 0,507228891 | 0,667745355 |
| b0806 | 261 | 0,52 | -0,206100384 | 4,429177601 | 0,439185058 | 0,507516056 | 0,667745355 |
| b1659 | 933 | 0,54 | -0,150809349 | 4,674130881 | 0,439143849 | 0,507535972 | 0,667745355 |
| b1994 | 1017 | 0,55 | -0,307734686 | 2,711010745 | 0,439036674 | 0,507587778 | 0,667745355 |
| b1703 | 834 | 0,51 | 0,126231263 | 6,797183511 | 0,438598201 | 0,507799821 | 0,667828286 |
| b1315 | 1056 | 0,55 | 0,299294637 | 2,810063327 | 0,436235672 | 0,508944955 | 0,669104856 |
| b4443 | 206 | 0,43 | 0,179683883 | 11,62984544 | 0,435980166 | 0,509069067 | 0,669104856 |
| b0118 | 2598 | 0,56 | 0,222997631 | 12,30576546 | 0,434828158 | 0,509629305 | 0,669549298 |
| b0553 | 2297 | 0,44 | 0,256971155 | 10,76771852 | 0,434670661 | 0,509705981 | 0,669549298 |
| b1383 | 327 | 0,45 | 0,225296465 | 3,986051795 | 0,433828035 | 0,510116543 | 0,669892278 |
| b1551 | 156 | 0,44 | 0,872940476 | 0,455059768 | 0,433289295 | 0,51037934 | 0,670041066 |
| b0473 | 1875 | 0,53 | -0,329971307 | 9,657962218 | 0,432894839 | 0,510571903 | 0,67009759 |
| b1329 | 1614 | 0,48 | 0,158220165 | 7,619852679 | 0,432483756 | 0,510772717 | 0,670136191 |
| b4588 | 102 | 0,49 | 0,869830953 | 0,488116829 | 0,432222627 | 0,510900349 | 0,670136191 |
| b4421 | 108 | 0,58 | 0,639102411 | 0,671649966 | 0,430596003 | 0,511696644 | 0,670984305 |
| b0710 | 744 | 0,53 | 0,146667223 | 8,042061881 | 0,428515535 | 0,512718253 | 0,671727099 |
| b4426 | 96 | 0,5 | 0,866546511 | 0,611506391 | 0,428453221 | 0,512748907 | 0,671727099 |
| b2667 | 300 | 0,53 | 0,395894137 | 3,081508202 | 0,428231864 | 0,512857824 | 0,671727099 |
| b0454 | 390 | 0,54 | -0,218993514 | 4,269557294 | 0,42789458 | 0,513023858 | 0,671727099 |
| b2376 | 276 | 0,4 | -0,332943384 | 2,626577952 | 0,427812927 | 0,513064068 | 0,671727099 |
| b3747 | 1869 | 0,51 | 0,138260398 | 6,179166223 | 0,42743278 | 0,51325134 | 0,671727099 |
| b4646 | 394 | 0,44 | 0,326882466 | 2,489069387 | 0,427309276 | 0,513312208 | 0,671727099 |
| b1590 | 855 | 0,53 | 0,229986827 | 4,414515776 | 0,426745351 | 0,513590291 | 0,67189483 |
| b0770 | 1434 | 0,5 | 0,371406848 | 1,61079253 | 0,426162371 | 0,513878048 | 0,672075112 |
| b1146 | 201 | 0,43 | 0,335556797 | 2,505095927 | 0,425516502 | 0,514197173 | 0,672296304 |

| | | | | | | | |
|---|---|---|---|---|---|---|---|
| b1715 | 45 | 0,36 | 0,867994799 | 0,410376926 | 0,424702424 | 0,514599904 | 0,672430545 |
| b4673 | 66 | 0,36 | 0,867994799 | 0,410376926 | 0,424702424 | 0,514599904 | 0,672430545 |
| b4654 | 163 | 0,42 | 0,862712382 | 0,488557894 | 0,422732047 | 0,515576944 | 0,67351089 |
| b2945 | 708 | 0,55 | -0,273673053 | 2,959342047 | 0,422422667 | 0,515730649 | 0,673515376 |
| b0912 | 285 | 0,49 | -0,145924527 | 9,141384401 | 0,421006911 | 0,516435043 | 0,673879958 |
| b0244 | 76 | 0,51 | 0,859449201 | 0,478209824 | 0,420956554 | 0,516460128 | 0,673879958 |
| b4629 | 49 | 0,51 | 0,859249668 | 0,410376926 | 0,420955049 | 0,516460878 | 0,673879958 |
| b2086 | 900 | 0,5 | 0,156328473 | 6,684688205 | 0,420413245 | 0,516730915 | 0,673897317 |
| b1268 | 1896 | 0,47 | -0,15866894 | 5,304484255 | 0,420314392 | 0,516780211 | 0,673897317 |
| b3993 | 636 | 0,58 | 0,343620032 | 2,663498803 | 0,420023636 | 0,516925251 | 0,673897317 |
| b3354 | 219 | 0,5 | -0,176171811 | 4,445168085 | 0,41922379 | 0,517324612 | 0,674221841 |
| b1523 | 360 | 0,53 | 0,276893507 | 3,340138018 | 0,418229151 | 0,517821989 | 0,674673882 |
| b4337 | 1233 | 0,54 | -0,201117301 | 4,096896768 | 0,417110773 | 0,518382244 | 0,675159329 |
| b0300 | 684 | 0,43 | 0,222914189 | 3,471567096 | 0,416884249 | 0,518495852 | 0,675159329 |
| b1501 | 2280 | 0,5 | -0,222393085 | 4,399163665 | 0,416491288 | 0,518693036 | 0,675219921 |
| b2378 | 921 | 0,51 | 0,348243386 | 2,738802915 | 0,415312142 | 0,519285512 | 0,675753663 |
| b0237 | 1458 | 0,51 | 0,162651817 | 9,546847737 | 0,415075439 | 0,519404589 | 0,675753663 |
| b2354 | 441 | 0,46 | 0,345118957 | 2,085807368 | 0,414405269 | 0,519741991 | 0,675996402 |
| b2818 | 1332 | 0,53 | 0,180146343 | 4,803937212 | 0,413983273 | 0,519954646 | 0,676076798 |
| b3219 | 717 | 0,44 | -0,159114925 | 4,655119366 | 0,413119622 | 0,52039034 | 0,676447072 |
| b0352 | 1014 | 0,57 | -0,219433094 | 3,553857655 | 0,408987479 | 0,522483869 | 0,678870423 |
| b3875 | 693 | 0,41 | 0,501295956 | 1,07695031 | 0,408581379 | 0,522690421 | 0,678870423 |
| b4168 | 462 | 0,52 | -0,157908894 | 5,765193816 | 0,408544825 | 0,52270902 | 0,678870423 |
| b4432 | 249 | 0,46 | 0,247370758 | 6,725646663 | 0,405796372 | 0,524110849 | 0,679254387 |
| b0001 | 66 | 0,52 | 0,84295981 | 0,477334582 | 0,405441549 | 0,52429231 | 0,679254387 |
| b4420 | 67 | 0,52 | 0,843166091 | 0,433254521 | 0,405440642 | 0,524292775 | 0,679254387 |
| b4429 | 56 | 0,52 | 0,842975591 | 0,421554886 | 0,405440378 | 0,52429291 | 0,679254387 |
| b0666 | 77 | 0,52 | 0,843166097 | 0,410376926 | 0,405440116 | 0,524293043 | 0,679254387 |
| b0673 | 77 | 0,52 | 0,843166097 | 0,410376926 | 0,405440116 | 0,524293043 | 0,679254387 |
| b4625 | 77 | 0,52 | 0,843166097 | 0,410376926 | 0,405440116 | 0,524293043 | 0,679254387 |
| b4701 | 56 | 0,52 | 0,843166097 | 0,410376926 | 0,405440116 | 0,524293043 | 0,679254387 |
| b0464 | 648 | 0,45 | 0,124768504 | 5,607443957 | 0,40529239 | 0,524368625 | 0,679254387 |
| b0993 | 2745 | 0,54 | 0,166313063 | 4,56297516 | 0,404838001 | 0,524601229 | 0,67935935 |
| b3059 | 618 | 0,54 | -0,149704162 | 4,925559997 | 0,403941366 | 0,525060759 | 0,679758036 |
| b0617 | 297 | 0,56 | -0,692062483 | 0,901680034 | 0,402066016 | 0,526024205 | 0,680779991 |
| b1273 | 252 | 0,47 | -0,131996903 | 6,543732922 | 0,401813984 | 0,526153924 | 0,680779991 |
| b1738 | 321 | 0,44 | 0,181252302 | 4,264290126 | 0,401238594 | 0,526450288 | 0,680966866 |
| b2843 | 837 | 0,5 | 0,154478443 | 4,506173762 | 0,400799552 | 0,526676623 | 0,681063077 |
| b1759 | 408 | 0,55 | 0,257465672 | 3,031260017 | 0,398500134 | 0,527864868 | 0,682402752 |
| b2926 | 1164 | 0,52 | -0,171594397 | 11,23594593 | 0,398074354 | 0,52808542 | 0,68246777 |
| b4662 | 132 | 0,47 | 0,833555872 | 0,410376926 | 0,397795343 | 0,528230036 | 0,68246777 |
| b0736 | 405 | 0,5 | 0,188597792 | 5,239772591 | 0,396013017 | 0,529155518 | 0,68246777 |
| b1471 | 1331 | 0,33 | -0,706376838 | 0,7740629 | 0,395968639 | 0,529178598 | 0,68246777 |
| b3234 | 1368 | 0,52 | -0,130427838 | 7,661429399 | 0,395399729 | 0,529474643 | 0,68246777 |
| b1576 | 192 | 0,43 | 0,830778509 | 0,603413059 | 0,393799062 | 0,530309183 | 0,68246777 |
| b1796 | 183 | 0,46 | 0,316294597 | 2,169112567 | 0,393643438 | 0,530390446 | 0,68246777 |
| b0565 | 954 | 0,43 | 0,204426689 | 7,692189566 | 0,393602023 | 0,530412076 | 0,68246777 |
| b4451 | 90 | 0,53 | 0,829257363 | 0,44467613 | 0,39273462 | 0,530865461 | 0,68246777 |
| b1665 | 77 | 0,53 | 0,829257377 | 0,410376926 | 0,392733873 | 0,530865851 | 0,68246777 |
| b1977 | 76 | 0,53 | 0,829257377 | 0,410376926 | 0,392733873 | 0,530865851 | 0,68246777 |
| b1984 | 76 | 0,53 | 0,829257377 | 0,410376926 | 0,392733873 | 0,530865851 | 0,68246777 |
| b1986 | 76 | 0,53 | 0,829257377 | 0,410376926 | 0,392733873 | 0,530865851 | 0,68246777 |
| b1989 | 76 | 0,53 | 0,829257377 | 0,410376926 | 0,392733873 | 0,530865851 | 0,68246777 |
| b4430 | 68 | 0,53 | 0,829257377 | 0,410376926 | 0,392733873 | 0,530865851 | 0,68246777 |
| b4700 | 59 | 0,53 | 0,829257377 | 0,410376926 | 0,392733873 | 0,530865851 | 0,68246777 |
| b1655 | 816 | 0,48 | 0,216285455 | 5,383417798 | 0,392466243 | 0,53100588 | 0,68246777 |
| b2005 | 375 | 0,53 | 0,491331609 | 1,277320323 | 0,392391703 | 0,531044893 | 0,68246777 |
| b0664 | 75 | 0,59 | 0,829183256 | 0,410376926 | 0,391099779 | 0,531721877 | 0,68246777 |
| b0665 | 75 | 0,59 | 0,829183256 | 0,410376926 | 0,391099779 | 0,531721877 | 0,68246777 |
| b1230 | 85 | 0,59 | 0,829183256 | 0,410376926 | 0,391099779 | 0,531721877 | 0,68246777 |
| b1231 | 85 | 0,59 | 0,829183256 | 0,410376926 | 0,391099779 | 0,531721877 | 0,68246777 |
| b2967 | 76 | 0,59 | 0,829183256 | 0,410376926 | 0,391099779 | 0,531721877 | 0,68246777 |
| b3069 | 76 | 0,59 | 0,829183256 | 0,410376926 | 0,391099779 | 0,531721877 | 0,68246777 |
| b4134 | 76 | 0,59 | 0,829183256 | 0,410376926 | 0,391099779 | 0,531721877 | 0,68246777 |
| b0885 | 705 | 0,54 | -0,164374685 | 5,128994644 | 0,389209171 | 0,532715388 | 0,6835472 |
| b1791 | 1182 | 0,53 | 0,254489893 | 3,436628458 | 0,388887803 | 0,532884599 | 0,683568625 |
| b4708 | 174 | 0,41 | 0,825004268 | 0,410376926 | 0,387419631 | 0,533658881 | 0,684226019 |
| b4688 | 205 | 0,43 | 0,823326947 | 0,454907773 | 0,387131906 | 0,533810859 | 0,684226019 |
| b3728 | 1041 | 0,51 | 0,227201206 | 5,749888332 | 0,387048249 | 0,533855062 | 0,684226019 |
| b0615 | 1533 | 0,56 | 0,316320046 | 1,931115201 | 0,385304907 | 0,534777725 | 0,685212625 |
| b1640 | 1110 | 0,55 | -0,131675033 | 6,015165595 | 0,382706426 | 0,53615835 | 0,686785289 |
| b4536 | 240 | 0,44 | 0,295406836 | 3,887553137 | 0,382309968 | 0,536369566 | 0,686859542 |
| b1162 | 732 | 0,49 | -0,123207063 | 5,935145047 | 0,380605631 | 0,537279291 | 0,687827987 |
| b4270 | 85 | 0,54 | 0,811935399 | 0,421843572 | 0,377287252 | 0,539058641 | 0,68990886 |
| b1946 | 414 | 0,55 | 0,480388931 | 1,070931442 | 0,376949048 | 0,539240594 | 0,689944717 |
| b3585 | 975 | 0,48 | -0,149517358 | 4,861594707 | 0,376324421 | 0,539576938 | 0,690178036 |
| b0015 | 1131 | 0,55 | 0,264931878 | 8,235919164 | 0,375322441 | 0,540117279 | 0,690672082 |
| b2914 | 660 | 0,52 | 0,138207932 | 8,534281955 | 0,374670756 | 0,540469249 | 0,690925035 |

| | | | | | | | |
|---|---|---|---|---|---|---|---|
| b3123 | 377 | 0,62 | -0,174519703 | 12,88633405 | 0,374064327 | 0,540797154 | 0,691057711 |
| b4491 | 2648 | 0,43 | -0,2114471 | 3,957593087 | 0,373908624 | 0,540881404 | 0,691057711 |
| b1427 | 540 | 0,43 | 0,166461979 | 5,432226568 | 0,372180951 | 0,541817857 | 0,691948087 |
| b4235 | 1353 | 0,54 | -0,121096778 | 8,062952724 | 0,372053502 | 0,541887056 | 0,691948087 |
| b0071 | 606 | 0,51 | 0,206855563 | 5,319310884 | 0,371505664 | 0,542184694 | 0,692090423 |
| b2045 | 1281 | 0,54 | 0,219813295 | 2,63426861 | 0,371280053 | 0,542307355 | 0,692090423 |
| b3711 | 960 | 0,52 | -0,170193181 | 4,457822792 | 0,369929649 | 0,543042618 | 0,692831487 |
| b3933 | 960 | 0,57 | -0,128399499 | 7,217448593 | 0,369390704 | 0,543336574 | 0,693009256 |
| b0256 | 1152 | 0,46 | -0,18861727 | 3,96366879 | 0,36864692 | 0,543742738 | 0,69333 |
| b1766 | 1857 | 0,52 | 0,105597434 | 6,374401764 | 0,367632658 | 0,544297509 | 0,693788386 |
| b1881 | 645 | 0,53 | 0,266185569 | 3,178550588 | 0,367423915 | 0,544411814 | 0,693788386 |
| b4357 | 915 | 0,47 | 0,19014799 | 5,427696749 | 0,364934545 | 0,545778406 | 0,695332238 |
| b4387 | 645 | 0,56 | -0,143353966 | 5,293332976 | 0,363623748 | 0,546500554 | 0,695906938 |
| b0506 | 816 | 0,53 | -0,166388619 | 9,028601985 | 0,363552184 | 0,546540032 | 0,695906938 |
| b3195 | 810 | 0,55 | -0,119048348 | 6,469198386 | 0,363245959 | 0,546709016 | 0,695924399 |
| b3334 | 462 | 0,48 | 0,32981147 | 2,201191898 | 0,358549253 | 0,549313049 | 0,699040626 |
| b4454 | 66 | 0,55 | 0,787155722 | 0,421896389 | 0,355436094 | 0,551051903 | 0,700855624 |
| b1574 | 53 | 0,55 | 0,787155728 | 0,410376926 | 0,355435859 | 0,551052034 | 0,700855624 |
| b1106 | 825 | 0,53 | 0,126077832 | 5,535278719 | 0,354611549 | 0,551514181 | 0,701244414 |
| b2036 | 1104 | 0,36 | 0,143208629 | 7,876501193 | 0,353399439 | 0,55219507 | 0,701911033 |
| b4595 | 174 | 0,51 | 0,195899702 | 4,559309441 | 0,351613743 | 0,553201048 | 0,702990388 |
| b4569 | 1745 | 0,46 | 0,254892898 | 2,820030462 | 0,350612533 | 0,553766595 | 0,703509603 |
| b1569 | 231 | 0,47 | 0,502472612 | 1,064150299 | 0,350212127 | 0,553993074 | 0,703597892 |
| b2417 | 510 | 0,47 | -0,141904047 | 10,76820591 | 0,349802935 | 0,554224705 | 0,703692671 |
| b1912 | 549 | 0,52 | -0,124790451 | 6,057432991 | 0,348771975 | 0,55480911 | 0,704067749 |
| b0310 | 669 | 0,37 | -0,387844431 | 1,239347084 | 0,348727599 | 0,554834291 | 0,704067749 |
| b1648 | 240 | 0,52 | -0,261163259 | 4,193949656 | 0,346685524 | 0,555995398 | 0,705341459 |
| b4254 | 1005 | 0,52 | 0,261657216 | 3,114252018 | 0,345805961 | 0,55649693 | 0,705648199 |
| b2082 | 219 | 0,51 | 0,173040421 | 3,908758147 | 0,345515287 | 0,556662863 | 0,705648199 |
| b1025 | 1359 | 0,39 | 0,211670096 | 3,836257152 | 0,345433598 | 0,556709512 | 0,705648199 |
| b1549 | 411 | 0,32 | 0,455855337 | 1,055985126 | 0,344847994 | 0,557044146 | 0,705710305 |
| b0804 | 678 | 0,53 | 0,223285384 | 3,781984456 | 0,3447968 | 0,557073418 | 0,705710305 |
| b1891 | 579 | 0,49 | -0,217531588 | 3,698343144 | 0,342135491 | 0,558599162 | 0,707443189 |
| b1769 | 1359 | 0,45 | 0,31105176 | 2,56029869 | 0,340761105 | 0,559390227 | 0,708244914 |
| b0108 | 441 | 0,53 | 0,49383869 | 0,865387284 | 0,339829916 | 0,559927414 | 0,708724843 |
| b1581 | 1215 | 0,51 | 0,181845803 | 3,465920105 | 0,33826058 | 0,560834973 | 0,709673165 |
| b4313 | 597 | 0,47 | 0,308332001 | 2,119228719 | 0,336943521 | 0,561598815 | 0,710439145 |
| b1737 | 1359 | 0,49 | 0,159489309 | 4,083122066 | 0,336589142 | 0,561804682 | 0,710440397 |
| b0877 | 993 | 0,5 | -0,152187973 | 6,090546879 | 0,336396204 | 0,561916824 | 0,710440397 |
| b2283 | 2727 | 0,56 | 0,245245389 | 11,39881105 | 0,335269789 | 0,562572398 | 0,711068666 |
| b2071 | 462 | 0,41 | 0,528865911 | 1,32547886 | 0,33438533 | 0,563088186 | 0,711519946 |
| b4509 | 141 | 0,48 | 0,761178224 | 0,455283754 | 0,333856594 | 0,563396962 | 0,711709466 |
| b0268 | 909 | 0,62 | 0,126338905 | 5,253888297 | 0,332556713 | 0,564157467 | 0,712469362 |
| b0971 | 88 | 0,6 | 0,760494709 | 0,410376926 | 0,330633753 | 0,565286149 | 0,712726279 |
| b2691 | 77 | 0,6 | 0,760494709 | 0,410376926 | 0,330633753 | 0,565286149 | 0,712726279 |
| b2692 | 77 | 0,6 | 0,760494709 | 0,410376926 | 0,330633753 | 0,565286149 | 0,712726279 |
| b2693 | 77 | 0,6 | 0,760494709 | 0,410376926 | 0,330633753 | 0,565286149 | 0,712726279 |
| b2694 | 77 | 0,6 | 0,760494709 | 0,410376926 | 0,330633753 | 0,565286149 | 0,712726279 |
| b3857 | 585 | 0,5 | -0,15133807 | 4,7710006 | 0,330584662 | 0,565315021 | 0,712726279 |
| b4690 | 75 | 0,56 | 0,75718883 | 0,444137069 | 0,32942731 | 0,565996503 | 0,713384794 |
| b0951 | 1641 | 0,5 | -0,1158897 | 6,644985591 | 0,32736492 | 0,567214854 | 0,71459516 |
| b3723 | 837 | 0,43 | 0,398154971 | 1,798251224 | 0,326745128 | 0,567581989 | 0,71459516 |
| b3461 | 855 | 0,54 | -0,116207575 | 9,011025903 | 0,326600018 | 0,567668013 | 0,71459516 |
| b0449 | 1782 | 0,56 | 0,1521608 | 4,878081748 | 0,326594366 | 0,567671364 | 0,71459516 |
| b1838 | 657 | 0,48 | -0,211287646 | 4,283129082 | 0,32643408 | 0,567766414 | 0,71459516 |
| b3856 | 528 | 0,49 | 0,174513449 | 4,163225482 | 0,326186277 | 0,567913423 | 0,71459516 |
| b0324 | 1383 | 0,56 | 0,123001094 | 5,315959044 | 0,324973901 | 0,568633734 | 0,7153007 |
| b1669 | 813 | 0,53 | 0,197770655 | 3,766317501 | 0,324597861 | 0,568857513 | 0,715381418 |
| b1772 | 948 | 0,45 | 0,26208107 | 2,666747567 | 0,324182827 | 0,569104696 | 0,715491514 |
| b3842 | 489 | 0,5 | 0,172945788 | 4,439973689 | 0,323522562 | 0,569498365 | 0,715762685 |
| b3148 | 396 | 0,55 | 0,160790036 | 4,952594777 | 0,322920889 | 0,569857563 | 0,715762685 |
| b0004 | 1287 | 0,53 | 0,147357772 | 8,665248679 | 0,322775286 | 0,569944554 | 0,715762685 |
| b2752 | 909 | 0,53 | 0,247899561 | 3,006290466 | 0,322750818 | 0,569959175 | 0,715762685 |
| b3725 | 774 | 0,52 | -0,243423332 | 6,278552761 | 0,321968759 | 0,570426888 | 0,716039997 |
| b0277 | 627 | 0,45 | -0,18611385 | 3,541186104 | 0,321847432 | 0,570499516 | 0,716039997 |
| b4633 | 222 | 0,53 | 0,227183915 | 2,838506367 | 0,321236669 | 0,570865398 | 0,716298632 |
| b1220 | 1395 | 0,51 | -0,148075593 | 4,793040766 | 0,320449485 | 0,571337646 | 0,716690548 |
| b2720 | 543 | 0,56 | 0,335139459 | 1,74041694 | 0,320051735 | 0,571576556 | 0,716789626 |
| b3794 | 741 | 0,53 | 0,135940503 | 5,308498689 | 0,31929816 | 0,57202973 | 0,717157273 |
| b1033 | 939 | 0,51 | 0,134265565 | 7,200892756 | 0,318241482 | 0,572666371 | 0,717754663 |
| b0875 | 696 | 0,52 | -0,220911853 | 4,131513512 | 0,315124693 | 0,574552363 | 0,719917163 |
| b1404 | 1152 | 0,46 | 0,731246902 | 0,410376926 | 0,314453625 | 0,574960035 | 0,720025392 |
| b4284 | 1152 | 0,46 | 0,731246902 | 0,410376926 | 0,314453625 | 0,574960035 | 0,720025392 |
| b3561 | 996 | 0,47 | 0,249231351 | 3,158636889 | 0,313456217 | 0,575567016 | 0,720584181 |
| b2451 | 1404 | 0,59 | -0,195165463 | 2,899103447 | 0,313089743 | 0,575790356 | 0,72066249 |
| b2912 | 549 | 0,52 | 0,232899786 | 4,038483431 | 0,31190807 | 0,576511671 | 0,721363849 |
| b0233 | 399 | 0,44 | 0,227416021 | 3,240072858 | 0,311426091 | 0,576806394 | 0,721531192 |
| b3938 | 318 | 0,52 | -0,206633914 | 6,602672887 | 0,310221406 | 0,577544353 | 0,722252732 |

| | | | | | | | |
|---|---|---|---|---|---|---|---|
| b1988 | 918 | 0,48 | 0,209002886 | 3,444126523 | 0,308554701 | 0,578568436 | 0,723331584 |
| b2058 | 489 | 0,54 | 0,402798363 | 1,629533626 | 0,307683111 | 0,579105413 | 0,723801021 |
| b3136 | 1155 | 0,57 | 0,221325529 | 2,632024093 | 0,306989008 | 0,579533755 | 0,724134455 |
| b3605 | 1191 | 0,56 | -0,362259638 | 7,550368336 | 0,305489514 | 0,58046128 | 0,72455316 |
| b0396 | 1185 | 0,51 | -0,139235729 | 4,547471768 | 0,305002952 | 0,580762886 | 0,72455316 |
| b1085 | 321 | 0,45 | 0,126293213 | 6,294781267 | 0,30450774 | 0,581070176 | 0,72455316 |
| b1571 | 156 | 0,48 | 0,724350958 | 0,933005088 | 0,303263905 | 0,581843444 | 0,72455316 |
| b3269 | 1182 | 0,5 | -0,214532946 | 3,006377319 | 0,302965709 | 0,582029134 | 0,72455316 |
| b1193 | 612 | 0,52 | 0,139153379 | 5,182880938 | 0,302902415 | 0,582068563 | 0,72455316 |
| b3174 | 87 | 0,57 | 0,724688561 | 0,488249675 | 0,302655752 | 0,582222273 | 0,72455316 |
| b0202 | 77 | 0,57 | 0,724783896 | 0,410376926 | 0,302654524 | 0,582223039 | 0,72455316 |
| b0668 | 75 | 0,57 | 0,724783896 | 0,410376926 | 0,302654524 | 0,582223039 | 0,72455316 |
| b0670 | 75 | 0,57 | 0,724783896 | 0,410376926 | 0,302654524 | 0,582223039 | 0,72455316 |
| b2652 | 76 | 0,57 | 0,724783896 | 0,410376926 | 0,302654524 | 0,582223039 | 0,72455316 |
| b2864 | 74 | 0,57 | 0,724783896 | 0,410376926 | 0,302654524 | 0,582223039 | 0,72455316 |
| b3277 | 77 | 0,57 | 0,724783896 | 0,410376926 | 0,302654524 | 0,582223039 | 0,72455316 |
| b3852 | 77 | 0,57 | 0,724783896 | 0,410376926 | 0,302654524 | 0,582223039 | 0,72455316 |
| b1909 | 87 | 0,6 | 0,608747703 | 0,572426975 | 0,302541134 | 0,582293726 | 0,72455316 |
| b3173 | 1626 | 0,43 | -0,16149257 | 3,710353396 | 0,301824376 | 0,582740955 | 0,724908398 |
| b4437 | 136 | 0,54 | 0,295657145 | 2,277390157 | 0,301315426 | 0,583058939 | 0,72510271 |
| b0843 | 285 | 0,53 | 0,176280006 | 3,175987295 | 0,300980711 | 0,583268255 | 0,725161809 |
| b3215 | 675 | 0,42 | 0,499778171 | 1,16215952 | 0,300452391 | 0,583598951 | 0,725371741 |
| b1335 | 516 | 0,53 | -0,18825161 | 4,536067925 | 0,296140476 | 0,586312174 | 0,728542047 |
| b0762 | 150 | 0,41 | 0,127001054 | 5,575507568 | 0,294360071 | 0,587439947 | 0,729741087 |
| b3263 | 180 | 0,41 | 0,338220967 | 3,076551808 | 0,293817812 | 0,58778431 | 0,72996655 |
| b0223 | 768 | 0,54 | 0,102291286 | 6,286333909 | 0,292962199 | 0,588328507 | 0,730375272 |
| b2633 | 822 | 0,56 | 0,251487898 | 2,35443237 | 0,292669681 | 0,588514794 | 0,730375272 |
| b3288 | 948 | 0,53 | -0,129135404 | 8,367318464 | 0,292532348 | 0,588602294 | 0,730375272 |
| b1728 | 591 | 0,51 | -0,241766539 | 2,913867704 | 0,291899479 | 0,589005863 | 0,730673755 |
| b4299 | 789 | 0,41 | -0,219921221 | 3,243129811 | 0,291353526 | 0,589354462 | 0,730903901 |
| b1542 | 1461 | 0,49 | 0,214798573 | 3,10190283 | 0,290516061 | 0,589890016 | 0,731365713 |
| b1945 | 1005 | 0,53 | 0,212813075 | 2,647653404 | 0,289554793 | 0,59050597 | 0,731926925 |
| b4520 | 219 | 0,4 | -0,222175116 | 2,752723587 | 0,289283771 | 0,590679873 | 0,731940058 |
| b2357 | 495 | 0,54 | 0,702457683 | 0,5530997 | 0,289008606 | 0,59085654 | 0,731956609 |
| b3504 | 2423 | 0,34 | 0,491850796 | 1,025264079 | 0,287995222 | 0,591508112 | 0,732374675 |
| b1311 | 882 | 0,54 | 0,329580147 | 1,787554464 | 0,287975479 | 0,591520821 | 0,732374675 |
| b2739 | 777 | 0,51 | 0,181432102 | 3,276537244 | 0,286559115 | 0,592434015 | 0,733302749 |
| b0944 | 711 | 0,52 | 0,169543191 | 3,464071124 | 0,285576428 | 0,593069305 | 0,733810719 |
| b4277 | 330 | 0,58 | 0,517153971 | 0,763740132 | 0,285235705 | 0,593289905 | 0,733810719 |
| b3151 | 1041 | 0,58 | 0,12141729 | 4,946193993 | 0,285165199 | 0,593335575 | 0,733810719 |
| b3912 | 699 | 0,51 | -0,116810304 | 7,981678247 | 0,283774757 | 0,594237714 | 0,73461685 |
| b1328 | 900 | 0,47 | 0,149651378 | 4,440293989 | 0,283655542 | 0,594315194 | 0,73461685 |
| b1869 | 396 | 0,51 | 0,120654164 | 5,766915246 | 0,280687884 | 0,596250708 | 0,736806085 |
| b0860 | 732 | 0,45 | 0,173171418 | 5,028130536 | 0,280182516 | 0,596581614 | 0,736826007 |
| b4331 | 555 | 0,51 | 0,192886712 | 3,490820729 | 0,280161135 | 0,596595622 | 0,736826007 |
| b2545 | 1062 | 0,55 | -0,16365653 | 4,236586148 | 0,278905418 | 0,597419543 | 0,73698032 |
| b3748 | 420 | 0,48 | 0,148076142 | 8,276909724 | 0,277976248 | 0,598030734 | 0,73698032 |
| b4513 | 90 | 0,49 | 0,556321383 | 0,661053533 | 0,27727518 | 0,598492748 | 0,73698032 |
| b4603 | 91 | 0,35 | 0,694911579 | 0,421808067 | 0,275441834 | 0,599704484 | 0,73698032 |
| b4665 | 60 | 0,35 | 0,694462139 | 0,410376926 | 0,275441651 | 0,599704605 | 0,73698032 |
| b4463 | 1455 | 0,51 | 0,199236288 | 3,133964861 | 0,275264533 | 0,599821942 | 0,73698032 |
| b3545 | 77 | 0,61 | 0,690926823 | 0,444415631 | 0,274982532 | 0,600008862 | 0,73698032 |
| b0536 | 77 | 0,61 | 0,691230351 | 0,410376926 | 0,274982038 | 0,60000919 | 0,73698032 |
| b0883 | 88 | 0,61 | 0,691230351 | 0,410376926 | 0,274982038 | 0,60000919 | 0,73698032 |
| b1032 | 88 | 0,61 | 0,691230351 | 0,410376926 | 0,274982038 | 0,60000919 | 0,73698032 |
| b1666 | 77 | 0,61 | 0,691230351 | 0,410376926 | 0,274982038 | 0,60000919 | 0,73698032 |
| b1911 | 76 | 0,61 | 0,691230351 | 0,410376926 | 0,274982038 | 0,60000919 | 0,73698032 |
| b2396 | 76 | 0,61 | 0,691230351 | 0,410376926 | 0,274982038 | 0,60000919 | 0,73698032 |
| b2397 | 76 | 0,61 | 0,691230351 | 0,410376926 | 0,274982038 | 0,60000919 | 0,73698032 |
| b3798 | 87 | 0,61 | 0,691230351 | 0,410376926 | 0,274982038 | 0,60000919 | 0,73698032 |
| b4163 | 76 | 0,61 | 0,691230351 | 0,410376926 | 0,274982038 | 0,60000919 | 0,73698032 |
| b4164 | 76 | 0,61 | 0,691230351 | 0,410376926 | 0,274982038 | 0,60000919 | 0,73698032 |
| b4165 | 76 | 0,61 | 0,691230351 | 0,410376926 | 0,274982038 | 0,60000919 | 0,73698032 |
| b4368 | 87 | 0,61 | 0,691230351 | 0,410376926 | 0,274982038 | 0,60000919 | 0,73698032 |
| b4370 | 87 | 0,61 | 0,691230351 | 0,410376926 | 0,274982038 | 0,60000919 | 0,73698032 |
| b2413 | 762 | 0,5 | 0,133267499 | 4,908848025 | 0,27465201 | 0,600228098 | 0,737047215 |
| b1135 | 654 | 0,51 | 0,210574657 | 4,410549283 | 0,273016448 | 0,601315454 | 0,737461704 |
| b2782 | 336 | 0,46 | -0,14895698 | 4,760473685 | 0,272221876 | 0,601845198 | 0,737461704 |
| b2092 | 1358 | 0,51 | -0,162670516 | 13,2420452 | 0,272120279 | 0,601913004 | 0,737461704 |
| b3791 | 1131 | 0,54 | 0,112468074 | 6,435872684 | 0,271969548 | 0,602013633 | 0,737461704 |
| b0021 | 504 | 0,55 | 0,680600911 | 0,410376926 | 0,271920339 | 0,602046492 | 0,737461704 |
| b0264 | 504 | 0,55 | 0,680600911 | 0,410376926 | 0,271920339 | 0,602046492 | 0,737461704 |
| b0274 | 504 | 0,55 | 0,680600911 | 0,410376926 | 0,271920339 | 0,602046492 | 0,737461704 |
| b1893 | 504 | 0,55 | 0,680600911 | 0,410376926 | 0,271920339 | 0,602046492 | 0,737461704 |
| b3445 | 504 | 0,55 | 0,680600911 | 0,410376926 | 0,271920339 | 0,602046492 | 0,737461704 |
| b1006 | 1329 | 0,54 | -0,145724387 | 4,018421984 | 0,271183324 | 0,602539091 | 0,737830172 |
| b0453 | 573 | 0,54 | 0,175199608 | 9,885345091 | 0,270709263 | 0,602856389 | 0,737830172 |
| b0214 | 468 | 0,5 | -0,152165461 | 4,413681506 | 0,269844173 | 0,603436321 | 0,737830172 |

| | | | | | | | |
|---|---|---|---|---|---|---|---|
| b3989 | 540 | 0,42 | 0,196000233 | 3,374510947 | 0,269819275 | 0,60345303 | 0,737830172 |
| b4419 | 108 | 0,56 | 0,683170328 | 0,410376926 | 0,269778028 | 0,603480712 | 0,737830172 |
| b4423 | 108 | 0,56 | 0,683170328 | 0,410376926 | 0,269778028 | 0,603480712 | 0,737830172 |
| b1538 | 2046 | 0,5 | 0,126585385 | 8,269807944 | 0,269749819 | 0,603499645 | 0,737830172 |
| b3176 | 1338 | 0,54 | 0,097480946 | 8,446761313 | 0,268950041 | 0,604036965 | 0,738285704 |
| b3446 | 285 | 0,46 | -0,381294715 | 1,323234068 | 0,268046749 | 0,604645049 | 0,738827456 |
| b1974 | 531 | 0,42 | -0,268676065 | 1,95703941 | 0,267090805 | 0,605289995 | 0,739413943 |
| b2934 | 444 | 0,42 | 0,356778111 | 1,296481073 | 0,265942667 | 0,606066544 | 0,739987877 |
| b0542 | 1564 | 0,5 | 0,303523155 | 2,681414635 | 0,26585498 | 0,606125939 | 0,739987877 |
| b3903 | 1260 | 0,56 | 0,242153791 | 2,12651615 | 0,265335988 | 0,606477732 | 0,739987877 |
| b2880 | 780 | 0,54 | 0,233535196 | 2,361713198 | 0,265155714 | 0,60660003 | 0,739987877 |
| b3554 | 711 | 0,5 | -0,100245428 | 8,394262047 | 0,264816413 | 0,606830356 | 0,739987877 |
| b3079 | 1071 | 0,52 | 0,326654196 | 2,4759283 | 0,264571742 | 0,60699656 | 0,739987877 |
| b3225 | 894 | 0,52 | -0,155790407 | 5,425396098 | 0,264503578 | 0,607042882 | 0,739987877 |
| b3660 | 924 | 0,49 | 0,12633359 | 5,173992266 | 0,264448024 | 0,607080639 | 0,739987877 |
| b4647 | 185 | 0,38 | 0,677102373 | 0,521047258 | 0,263199762 | 0,607930348 | 0,74072904 |
| b1397 | 1206 | 0,56 | 0,275468182 | 6,128516085 | 0,26306143 | 0,60802467 | 0,74072904 |
| b2750 | 606 | 0,52 | 0,178034811 | 3,074919245 | 0,262755393 | 0,608233451 | 0,74072904 |
| b4161 | 1053 | 0,54 | 0,10287024 | 7,019585103 | 0,262585011 | 0,608349754 | 0,74072904 |
| b2387 | 327 | 0,51 | 0,432475075 | 1,001473966 | 0,261041671 | 0,609405415 | 0,741812892 |
| b4452 | 105 | 0,5 | 0,259338466 | 2,559116473 | 0,260658066 | 0,609668415 | 0,741931532 |
| b2635 | 237 | 0,53 | 0,562249789 | 0,87377153 | 0,259945459 | 0,610157628 | 0,74212742 |
| b0413 | 450 | 0,51 | -0,120234478 | 6,550793774 | 0,259896819 | 0,61019105 | 0,74212742 |
| b2103 | 801 | 0,55 | 0,188305904 | 3,202965485 | 0,259558501 | 0,610423633 | 0,74212742 |
| b3035 | 1482 | 0,51 | -0,112279411 | 9,831615109 | 0,259459543 | 0,610491699 | 0,74212742 |
| b3876 | 1404 | 0,45 | 0,284683682 | 2,116269001 | 0,258653135 | 0,611046983 | 0,742601024 |
| b1939 | 996 | 0,56 | 0,257621538 | 2,254057646 | 0,257121906 | 0,612104375 | 0,743684415 |
| b2984 | 759 | 0,53 | 0,428506691 | 1,239995307 | 0,256155951 | 0,612773454 | 0,744295561 |
| b3895 | 834 | 0,53 | 0,129686884 | 5,359061284 | 0,255680532 | 0,61310334 | 0,744494492 |
| b4135 | 576 | 0,53 | 0,106676115 | 7,764353539 | 0,253121158 | 0,614885898 | 0,746219181 |
| b4598 | 96 | 0,33 | 0,149453804 | 5,330454157 | 0,253036057 | 0,614945363 | 0,746219181 |
| b0501 | 491 | 0,33 | 0,489663959 | 1,016657229 | 0,252888683 | 0,615048372 | 0,746219181 |
| b1528 | 1191 | 0,51 | 0,189962625 | 3,510774653 | 0,252686688 | 0,615189619 | 0,746219181 |
| b1161 | 405 | 0,37 | 0,530519712 | 0,955958833 | 0,252341821 | 0,615430935 | 0,746309916 |
| b0887 | 1767 | 0,54 | 0,093165016 | 6,624547716 | 0,251848804 | 0,615776276 | 0,746526716 |
| b4616 | 140 | 0,51 | -0,552859081 | 0,642638323 | 0,25109374 | 0,616305992 | 0,746811898 |
| b2266 | 306 | 0,52 | -0,127374754 | 10,36244186 | 0,251038537 | 0,616344759 | 0,746811898 |
| b0376 | 1158 | 0,55 | 0,171949392 | 5,504883942 | 0,25064815 | 0,616619065 | 0,746942337 |
| b0548 | 171 | 0,49 | 0,655889909 | 0,582927828 | 0,249983396 | 0,617086769 | 0,747306917 |
| b0900 | 909 | 0,47 | -0,160920009 | 3,571758123 | 0,249641219 | 0,61732782 | 0,74739689 |
| b0360 | 366 | 0,53 | 0,651024604 | 0,410376926 | 0,248074976 | 0,618433819 | 0,747524374 |
| b1403 | 366 | 0,53 | 0,651024604 | 0,410376926 | 0,248074976 | 0,618433819 | 0,747524374 |
| b1997 | 366 | 0,53 | 0,651024604 | 0,410376926 | 0,248074976 | 0,618433819 | 0,747524374 |
| b2861 | 366 | 0,53 | 0,651024604 | 0,410376926 | 0,248074976 | 0,618433819 | 0,747524374 |
| b3044 | 366 | 0,53 | 0,651024604 | 0,410376926 | 0,248074976 | 0,618433819 | 0,747524374 |
| b4272 | 366 | 0,53 | 0,651024604 | 0,410376926 | 0,248074976 | 0,618433819 | 0,747524374 |
| b3325 | 1953 | 0,5 | -0,181357211 | 3,533575356 | 0,247661814 | 0,618726299 | 0,747572679 |
| b2611 | 792 | 0,53 | -0,151388655 | 5,105109115 | 0,247547367 | 0,618807371 | 0,747572679 |
| b1097 | 1023 | 0,51 | 0,14166848 | 5,06179291 | 0,247255614 | 0,619014147 | 0,747620967 |
| b1844 | 663 | 0,52 | 0,117597893 | 5,491596025 | 0,246696261 | 0,619411009 | 0,747841928 |
| b4415 | 153 | 0,48 | 0,463335109 | 0,917936749 | 0,246527567 | 0,619530807 | 0,747841928 |
| b3752 | 930 | 0,52 | 0,129537479 | 7,279781493 | 0,245121844 | 0,620531078 | 0,748847682 |
| b0606 | 1566 | 0,53 | 0,108514436 | 9,092480777 | 0,243618263 | 0,621604942 | 0,749891382 |
| b2022 | 1068 | 0,53 | -0,126396498 | 6,574043139 | 0,243357029 | 0,621791936 | 0,749891382 |
| b0946 | 543 | 0,49 | -0,124604169 | 6,234294696 | 0,243209111 | 0,621897873 | 0,749891382 |
| b4345 | 1047 | 0,37 | 0,190198647 | 3,06756815 | 0,242738359 | 0,622235285 | 0,750024305 |
| b4631 | 129 | 0,36 | 0,650147046 | 0,499178999 | 0,242588489 | 0,622342791 | 0,750024305 |
| b1192 | 915 | 0,53 | -0,118007143 | 5,307717959 | 0,241336715 | 0,623242331 | 0,750275656 |
| b1622 | 1173 | 0,51 | -0,138344377 | 5,83956399 | 0,240609725 | 0,623766086 | 0,750275656 |
| b2710 | 1440 | 0,53 | 0,173854523 | 4,20816282 | 0,240528565 | 0,623824618 | 0,750275656 |
| b0377 | 1221 | 0,51 | 0,135933315 | 4,988910525 | 0,24039587 | 0,623920343 | 0,750275656 |
| b0298 | 300 | 0,5 | 0,640547255 | 0,410376926 | 0,240205224 | 0,62405793 | 0,750275656 |
| b0373 | 300 | 0,5 | 0,640547255 | 0,410376926 | 0,240205224 | 0,62405793 | 0,750275656 |
| b0540 | 300 | 0,5 | 0,640547255 | 0,410376926 | 0,240205224 | 0,62405793 | 0,750275656 |
| b1027 | 300 | 0,5 | 0,640547255 | 0,410376926 | 0,240205224 | 0,62405793 | 0,750275656 |
| b2088 | 300 | 0,5 | 0,640547255 | 0,410376926 | 0,240205224 | 0,62405793 | 0,750275656 |
| b4689 | 255 | 0,49 | 0,636575509 | 0,410376926 | 0,236880559 | 0,626468274 | 0,752844165 |
| b3890 | 219 | 0,46 | -0,234379793 | 2,347148433 | 0,236605917 | 0,62666832 | 0,752844165 |
| b1578 | 693 | 0,55 | 0,634386127 | 0,410376926 | 0,236564836 | 0,626698255 | 0,752844165 |
| b1511 | 1593 | 0,52 | -0,152034666 | 7,12267256 | 0,236096347 | 0,627039866 | 0,752868887 |
| b1776 | 1077 | 0,5 | 0,166101971 | 3,233388362 | 0,236075897 | 0,627054787 | 0,752868887 |
| b0969 | 330 | 0,47 | 0,136151294 | 4,661482221 | 0,234492406 | 0,6282126 | 0,75405701 |
| b2277 | 1530 | 0,55 | 0,220643659 | 9,984974692 | 0,23295633 | 0,629340366 | 0,755208439 |
| b3885 | 600 | 0,5 | -0,101147392 | 7,727301831 | 0,231673814 | 0,630285489 | 0,756029535 |
| b0266 | 309 | 0,52 | -0,159853687 | 3,486014905 | 0,231570213 | 0,630361975 | 0,756029535 |
| b2055 | 747 | 0,46 | 0,504685554 | 0,809848627 | 0,23052658 | 0,631133652 | 0,756752549 |
| b1943 | 1128 | 0,55 | 0,232551479 | 2,148196808 | 0,230000492 | 0,631523464 | 0,757017428 |
| b3532 | 2340 | 0,55 | 0,128909936 | 4,559974764 | 0,229457594 | 0,631926307 | 0,757113136 |

| | | | | | | | |
|---|---|---|---|---|---|---|---|
| b2241 | 1629 | 0,57 | 0,230427427 | 2,110590666 | 0,229437603 | 0,631941152 | 0,757113136 |
| b3061 | 912 | 0,54 | -0,315437214 | 4,188543819 | 0,226829421 | 0,633884796 | 0,758582465 |
| b3807 | 321 | 0,54 | 0,164144387 | 4,915300737 | 0,225897525 | 0,634582579 | 0,758582465 |
| b2178 | 1095 | 0,51 | -0,119324224 | 4,303248054 | 0,225872471 | 0,634601363 | 0,758582465 |
| b2930 | 966 | 0,57 | -0,171684486 | 3,166735767 | 0,225871834 | 0,634601841 | 0,758582465 |
| b3726 | 891 | 0,56 | 0,283088288 | 4,744552341 | 0,225667844 | 0,63475483 | 0,758582465 |
| b0412 | 540 | 0,48 | -0,184301139 | 3,64926744 | 0,225647589 | 0,634770026 | 0,758582465 |
| b0391 | 285 | 0,52 | -0,115666994 | 6,300787571 | 0,225582236 | 0,63481906 | 0,758582465 |
| b4653 | 132 | 0,55 | 0,622169378 | 0,541362752 | 0,224826069 | 0,635387046 | 0,758582465 |
| b0976 | 399 | 0,57 | -0,206087349 | 3,379756322 | 0,224736409 | 0,63545447 | 0,758582465 |
| b3799 | 77 | 0,62 | 0,623549993 | 0,443499419 | 0,224402096 | 0,63570602 | 0,758582465 |
| b0744 | 76 | 0,62 | 0,62310681 | 0,410376926 | 0,224401694 | 0,635706322 | 0,758582465 |
| b0746 | 76 | 0,62 | 0,62310681 | 0,410376926 | 0,224401694 | 0,635706322 | 0,758582465 |
| b2401 | 76 | 0,62 | 0,62310681 | 0,410376926 | 0,224401694 | 0,635706322 | 0,758582465 |
| b2402 | 76 | 0,62 | 0,62310681 | 0,410376926 | 0,224401694 | 0,635706322 | 0,758582465 |
| b2403 | 76 | 0,62 | 0,62310681 | 0,410376926 | 0,224401694 | 0,635706322 | 0,758582465 |
| b3960 | 1374 | 0,56 | 0,118068626 | 6,766786432 | 0,223750385 | 0,636197052 | 0,75896598 |
| b2165 | 939 | 0,5 | 0,183536217 | 3,811742118 | 0,222252226 | 0,637329167 | 0,760114244 |
| b4626 | 63 | 0,33 | 0,622971999 | 0,410376926 | 0,221821482 | 0,637655532 | 0,760301169 |
| b0387 | 459 | 0,56 | -0,14608494 | 4,501064907 | 0,22124327 | 0,638094238 | 0,76046208 |
| b2079 | 723 | 0,54 | -0,1094459 | 5,210864474 | 0,221106934 | 0,638197781 | 0,76046208 |
| b1850 | 642 | 0,56 | 0,105571872 | 7,84983348 | 0,220973056 | 0,638299497 | 0,76046208 |
| b3535 | 189 | 0,46 | -0,17769869 | 4,291221191 | 0,220502863 | 0,638657029 | 0,760685837 |
| b1987 | 951 | 0,48 | -0,173660903 | 3,313025942 | 0,220090997 | 0,638970592 | 0,760857119 |
| b3177 | 849 | 0,52 | 0,102357771 | 5,790470506 | 0,219717766 | 0,639255051 | 0,760993662 |
| b2975 | 1683 | 0,53 | -0,451945766 | 8,408179175 | 0,219019654 | 0,63978791 | 0,761280773 |
| b0053 | 1287 | 0,54 | 0,102727177 | 9,124900288 | 0,218956798 | 0,639835938 | 0,761280773 |
| b0579 | 369 | 0,47 | -0,136749765 | 4,110538328 | 0,218436835 | 0,640233562 | 0,761551705 |
| b4550 | 219 | 0,43 | -0,165306477 | 3,87338179 | 0,217837146 | 0,640692869 | 0,761895845 |
| b4215 | 939 | 0,36 | -0,278155172 | 2,562306052 | 0,216283757 | 0,641886216 | 0,763112472 |
| b4394 | 513 | 0,56 | 0,143189155 | 4,192290475 | 0,215687078 | 0,642345984 | 0,763456563 |
| b1755 | 1536 | 0,57 | 0,205887672 | 4,151040584 | 0,215048653 | 0,642838775 | 0,763839711 |
| b3687 | 414 | 0,47 | -0,326443421 | 7,045929275 | 0,214718478 | 0,643093981 | 0,763887671 |
| b4555 | 294 | 0,43 | 0,191431663 | 2,929846567 | 0,214480135 | 0,643278354 | 0,763887671 |
| b2422 | 1098 | 0,59 | 0,161034315 | 3,911006825 | 0,214335317 | 0,643390442 | 0,763887671 |
| b0934 | 792 | 0,56 | -0,359650014 | 1,563875088 | 0,213933469 | 0,643701706 | 0,764054832 |
| b4264 | 999 | 0,47 | 0,130257418 | 4,333128916 | 0,213642424 | 0,643927366 | 0,764120322 |
| b4011 | 384 | 0,45 | -0,194387357 | 2,804358666 | 0,213365485 | 0,644142263 | 0,764173008 |
| b3844 | 702 | 0,53 | 0,084091731 | 7,944333831 | 0,212976876 | 0,644444098 | 0,764328777 |
| b4412 | 153 | 0,53 | 0,605014263 | 0,410376926 | 0,212611793 | 0,644727964 | 0,764463158 |
| b3911 | 1374 | 0,55 | -0,090899853 | 7,84574084 | 0,212271069 | 0,644993157 | 0,764541672 |
| b0045 | 1332 | 0,53 | -0,242265332 | 1,963171097 | 0,212088524 | 0,645135342 | 0,764541672 |
| b0787 | 714 | 0,42 | 0,215429944 | 2,610523907 | 0,211635924 | 0,645488194 | 0,764731234 |
| b3901 | 315 | 0,51 | 0,328993742 | 1,477662938 | 0,211445812 | 0,645636544 | 0,764731234 |
| b0502 | 422 | 0,49 | 0,182878416 | 2,90572635 | 0,210475509 | 0,64639496 | 0,765427268 |
| b2718 | 411 | 0,53 | 0,178570828 | 3,032847715 | 0,20869458 | 0,647792508 | 0,76579795 |
| b2916 | 894 | 0,54 | -0,095635212 | 5,949782655 | 0,208421709 | 0,648007274 | 0,76579795 |
| b4632 | 93 | 0,66 | 0,603299508 | 0,509980899 | 0,208333265 | 0,648076922 | 0,76579795 |
| b2189 | 77 | 0,66 | 0,602715763 | 0,410376926 | 0,208332194 | 0,648077765 | 0,76579795 |
| b2814 | 77 | 0,66 | 0,602715763 | 0,410376926 | 0,208332194 | 0,648077765 | 0,76579795 |
| b2815 | 77 | 0,66 | 0,602715763 | 0,410376926 | 0,208332194 | 0,648077765 | 0,76579795 |
| b2816 | 77 | 0,66 | 0,602715763 | 0,410376926 | 0,208332194 | 0,648077765 | 0,76579795 |
| b0754 | 1053 | 0,52 | -0,088443195 | 7,369196647 | 0,208014038 | 0,648328453 | 0,76579795 |
| b1500 | 198 | 0,37 | 0,600133731 | 0,444315403 | 0,207915237 | 0,64840635 | 0,76579795 |
| b1490 | 1383 | 0,44 | 0,158637042 | 5,749785032 | 0,207902233 | 0,648416604 | 0,76579795 |
| b0198 | 654 | 0,52 | 0,145392479 | 4,994486176 | 0,206947584 | 0,649170449 | 0,76635313 |
| b1735 | 843 | 0,42 | 0,12101581 | 3,779841518 | 0,206873982 | 0,649228656 | 0,76635313 |
| b2648 | 240 | 0,5 | 0,593872303 | 0,603859661 | 0,206378048 | 0,649621186 | 0,766614575 |
| b1605 | 1383 | 0,54 | 0,142092669 | 4,259180541 | 0,20461832 | 0,651018614 | 0,767883488 |
| b1660 | 1212 | 0,53 | 0,197784201 | 3,74661753 | 0,204592589 | 0,651039102 | 0,767883488 |
| b1289 | 354 | 0,5 | -0,161386083 | 3,391468933 | 0,203995041 | 0,651515306 | 0,768242989 |
| b2076 | 3078 | 0,56 | -0,128395595 | 5,135918468 | 0,20236244 | 0,652820672 | 0,769579761 |
| b0916 | 1233 | 0,52 | 0,120477101 | 3,938588854 | 0,202103377 | 0,653028391 | 0,769622205 |
| b3833 | 756 | 0,52 | 0,074224817 | 7,385762203 | 0,20140201 | 0,653591556 | 0,770083427 |
| b4141 | 1257 | 0,52 | -0,117883678 | 4,361215844 | 0,200626376 | 0,654215728 | 0,770616266 |
| b3370 | 1338 | 0,55 | -0,196274688 | 2,600208609 | 0,198747302 | 0,655733893 | 0,77206398 |
| b2384 | 1038 | 0,58 | 0,236273742 | 1,997103559 | 0,198678944 | 0,655789284 | 0,77206398 |
| b0036 | 786 | 0,54 | -0,228524988 | 2,320528736 | 0,197588034 | 0,6566748 | 0,772735281 |
| b2375 | 636 | 0,46 | 0,393908971 | 1,278566862 | 0,197551749 | 0,656704303 | 0,772735281 |
| b2260 | 1356 | 0,56 | 0,09763481 | 6,562498678 | 0,196538538 | 0,657529467 | 0,773503168 |
| b3196 | 978 | 0,52 | 0,097493174 | 5,757506541 | 0,19539054 | 0,658467482 | 0,774403373 |
| b4498 | 2035 | 0,46 | 0,138588177 | 6,971746585 | 0,193686541 | 0,659865891 | 0,775844418 |
| b3383 | 906 | 0,52 | 0,134097665 | 5,064975053 | 0,193161029 | 0,66029864 | 0,776149621 |
| b2679 | 993 | 0,53 | 0,142698529 | 6,106801417 | 0,192362416 | 0,66095763 | 0,776720529 |
| b1304 | 669 | 0,49 | 0,108234441 | 6,569096631 | 0,191865243 | 0,661368705 | 0,776797086 |
| b2962 | 276 | 0,47 | -0,127436496 | 6,05977206 | 0,191846118 | 0,66138453 | 0,776797086 |
| b0106 | 1203 | 0,52 | -0,171658606 | 3,189617022 | 0,191655011 | 0,661542721 | 0,776797086 |
| b0022 | 276 | 0,53 | 0,5702412 | 0,410376926 | 0,190483845 | 0,662514222 | 0,776920132 |

| | | | | | | | |
|---|---|---|---|---|---|---|---|
| b0265 | 276 | 0,53 | 0,5702412 | 0,410376926 | 0,190483845 | 0,662514222 | 0,776920132 |
| b0275 | 276 | 0,53 | 0,5702412 | 0,410376926 | 0,190483845 | 0,662514222 | 0,776920132 |
| b1894 | 276 | 0,53 | 0,5702412 | 0,410376926 | 0,190483845 | 0,662514222 | 0,776920132 |
| b4294 | 276 | 0,53 | 0,5702412 | 0,410376926 | 0,190483845 | 0,662514222 | 0,776920132 |
| b0720 | 1284 | 0,51 | -0,129352172 | 11,99355945 | 0,190189066 | 0,662759306 | 0,77700424 |
| b2851 | 492 | 0,33 | -0,496355985 | 0,681867475 | 0,189782 | 0,663098119 | 0,777198162 |
| b1036 | 492 | 0,54 | 0,129765384 | 4,950299311 | 0,189403281 | 0,663413726 | 0,77736479 |
| b1610 | 930 | 0,51 | -0,112700577 | 5,449179483 | 0,18869973 | 0,664001031 | 0,777849614 |
| b3500 | 1353 | 0,54 | 0,084499443 | 8,068301601 | 0,187890756 | 0,664677952 | 0,778439138 |
| b0438 | 1275 | 0,52 | 0,101219151 | 10,23221554 | 0,187565047 | 0,664950981 | 0,778555459 |
| b2356 | 270 | 0,53 | 0,56526439 | 0,489143704 | 0,187167581 | 0,665284545 | 0,778742578 |
| b1007 | 495 | 0,59 | 0,242087681 | 1,755645076 | 0,186625859 | 0,665739849 | 0,779072064 |
| b2643 | 459 | 0,54 | 0,246205763 | 2,651891283 | 0,185134633 | 0,666997252 | 0,780339777 |
| b0149 | 2535 | 0,55 | 0,081468803 | 7,506957737 | 0,184442545 | 0,667582861 | 0,780821081 |
| b3727 | 960 | 0,55 | 0,176947284 | 4,686348419 | 0,182631581 | 0,669121388 | 0,782416399 |
| b1325 | 966 | 0,55 | -0,085850421 | 6,267444683 | 0,181914044 | 0,669733475 | 0,782927865 |
| b3544 | 1608 | 0,52 | 0,153912701 | 11,61094679 | 0,181078228 | 0,670448261 | 0,783559089 |
| b3988 | 4224 | 0,54 | -0,133916145 | 12,55801865 | 0,180197088 | 0,671203921 | 0,783792401 |
| b0924 | 4461 | 0,56 | 0,099719196 | 9,507527838 | 0,179514656 | 0,671790669 | 0,783792401 |
| b1448 | 519 | 0,53 | -0,100142353 | 5,092124635 | 0,179391193 | 0,671896963 | 0,783792401 |
| b1347 | 210 | 0,35 | -0,483677885 | 0,61282542 | 0,179145068 | 0,672108987 | 0,783792401 |
| b0939 | 702 | 0,45 | 0,368492892 | 1,231353096 | 0,179060356 | 0,672182002 | 0,783792401 |
| b0203 | 76 | 0,63 | 0,555148896 | 0,410376926 | 0,179001606 | 0,672232652 | 0,783792401 |
| b3276 | 76 | 0,63 | 0,555148896 | 0,410376926 | 0,179001606 | 0,672232652 | 0,783792401 |
| b3853 | 76 | 0,63 | 0,555148896 | 0,410376926 | 0,179001606 | 0,672232652 | 0,783792401 |
| b0299 | 867 | 0,55 | 0,549394318 | 0,410376926 | 0,178198084 | 0,672926374 | 0,783792401 |
| b0372 | 867 | 0,55 | 0,549394318 | 0,410376926 | 0,178198084 | 0,672926374 | 0,783792401 |
| b0541 | 867 | 0,55 | 0,549394318 | 0,410376926 | 0,178198084 | 0,672926374 | 0,783792401 |
| b1026 | 867 | 0,55 | 0,549394318 | 0,410376926 | 0,178198084 | 0,672926374 | 0,783792401 |
| b2089 | 867 | 0,55 | 0,549394318 | 0,410376926 | 0,178198084 | 0,672926374 | 0,783792401 |
| b0491 | 780 | 0,46 | -0,110941488 | 4,665367782 | 0,177818481 | 0,673254745 | 0,783792401 |
| b2492 | 849 | 0,51 | -0,13529075 | 3,482384868 | 0,177623809 | 0,673423304 | 0,783792401 |
| b3204 | 492 | 0,52 | -0,073888599 | 7,561593225 | 0,17759772 | 0,673445902 | 0,783792401 |
| b4109 | 2229 | 0,51 | 0,189516576 | 4,06586129 | 0,177174489 | 0,67381277 | 0,783891759 |
| b1793 | 255 | 0,53 | 0,118342319 | 3,867575093 | 0,177095763 | 0,673881068 | 0,783891759 |
| b2836 | 2160 | 0,53 | 0,076299734 | 6,908270643 | 0,176333691 | 0,674543127 | 0,784409068 |
| b1348 | 195 | 0,41 | 0,442302268 | 0,582020033 | 0,176181203 | 0,674675805 | 0,784409068 |
| b2444 | 246 | 0,49 | -0,294455776 | 1,612102068 | 0,175085627 | 0,675631042 | 0,785310752 |
| b0918 | 747 | 0,53 | 0,07175924 | 7,342352066 | 0,17489023 | 0,675801778 | 0,785310752 |
| b1330 | 1032 | 0,43 | 0,097429643 | 5,34511495 | 0,173638701 | 0,67689802 | 0,786304978 |
| b1395 | 1428 | 0,53 | -0,312610514 | 5,533367247 | 0,173513165 | 0,677008235 | 0,786304978 |
| b4373 | 447 | 0,52 | 0,119112896 | 5,396690112 | 0,173104712 | 0,677367164 | 0,786518039 |
| b3520 | 603 | 0,48 | -0,155047854 | 3,645939674 | 0,172135787 | 0,6782206 | 0,787305032 |
| b1526 | 882 | 0,54 | 0,130287646 | 4,302023752 | 0,171739294 | 0,678570646 | 0,787337728 |
| b2410 | 999 | 0,52 | -0,105276571 | 6,046374157 | 0,17121253 | 0,679036434 | 0,787337728 |
| b0016 | 1113 | 0,52 | 0,536733416 | 0,410376926 | 0,171110108 | 0,679127099 | 0,787337728 |
| b0582 | 1113 | 0,52 | 0,536733416 | 0,410376926 | 0,171110108 | 0,679127099 | 0,787337728 |
| b2394 | 1113 | 0,52 | 0,536733416 | 0,410376926 | 0,171110108 | 0,679127099 | 0,787337728 |
| b3293 | 369 | 0,48 | 0,097848194 | 5,661135697 | 0,169203258 | 0,68082087 | 0,788174622 |
| b0361 | 906 | 0,55 | 0,534476606 | 0,410376926 | 0,168912694 | 0,681079944 | 0,788174622 |
| b1402 | 906 | 0,55 | 0,534476606 | 0,410376926 | 0,168912694 | 0,681079944 | 0,788174622 |
| b1996 | 906 | 0,55 | 0,534476606 | 0,410376926 | 0,168912694 | 0,681079944 | 0,788174622 |
| b2860 | 906 | 0,55 | 0,534476606 | 0,410376926 | 0,168912694 | 0,681079944 | 0,788174622 |
| b3045 | 906 | 0,55 | 0,534476606 | 0,410376926 | 0,168912694 | 0,681079944 | 0,788174622 |
| b4273 | 906 | 0,55 | 0,534476606 | 0,410376926 | 0,168912694 | 0,681079944 | 0,788174622 |
| b4124 | 720 | 0,49 | -0,089972054 | 5,631675159 | 0,16745847 | 0,682380494 | 0,789439386 |
| b1544 | 234 | 0,35 | 0,451246612 | 0,603421754 | 0,1672972 | 0,682525128 | 0,789439386 |
| b3677 | 450 | 0,48 | 0,184227612 | 2,83872589 | 0,166742007 | 0,683023671 | 0,789608979 |
| b1589 | 618 | 0,53 | 0,209753088 | 3,115296235 | 0,166620315 | 0,683133075 | 0,789608979 |
| b3080 | 2352 | 0,54 | -0,166304115 | 4,271721386 | 0,166545597 | 0,683200272 | 0,789608979 |
| b1726 | 537 | 0,44 | -0,086311821 | 5,926883052 | 0,166094763 | 0,683606096 | 0,789874329 |
| b3026 | 1350 | 0,53 | 0,108366275 | 4,835051086 | 0,165698927 | 0,683962944 | 0,790082968 |
| b2887 | 1920 | 0,53 | -0,127431575 | 3,631691114 | 0,165412287 | 0,684221661 | 0,790178172 |
| b3730 | 1371 | 0,53 | 0,084043647 | 9,098504806 | 0,160447827 | 0,688744548 | 0,795196564 |
| b1985 | 1488 | 0,49 | 0,109335028 | 5,02410246 | 0,159327056 | 0,689776837 | 0,796183307 |
| b1810 | 300 | 0,45 | 0,099807303 | 6,194664739 | 0,157476183 | 0,691490844 | 0,797874457 |
| b0534 | 516 | 0,5 | 0,414846502 | 0,868654985 | 0,157360878 | 0,691598007 | 0,797874457 |
| b0114 | 2664 | 0,53 | -0,111490731 | 10,21142689 | 0,156747895 | 0,692168473 | 0,798228055 |
| b1411 | 1293 | 0,51 | 0,121362223 | 3,806398856 | 0,156648926 | 0,692260698 | 0,798228055 |
| b0917 | 183 | 0,48 | -0,104361231 | 5,16360834 | 0,154616196 | 0,69416242 | 0,799904505 |
| b0838 | 627 | 0,52 | -0,123089048 | 7,319365824 | 0,154423527 | 0,694343419 | 0,799904505 |
| b1303 | 978 | 0,52 | -0,095773684 | 4,827445698 | 0,154283236 | 0,694475294 | 0,799904505 |
| b4643 | 15 | 0,67 | 0,517779472 | 0,410376926 | 0,154069144 | 0,694676677 | 0,799904505 |
| b4645 | 14 | 0,79 | 0,517779472 | 0,410376926 | 0,154069144 | 0,694676677 | 0,799904505 |
| b2486 | 1581 | 0,54 | 0,14991434 | 3,149101436 | 0,153953614 | 0,694785417 | 0,799904505 |
| b2176 | 1557 | 0,51 | 0,087889264 | 4,885671494 | 0,153747847 | 0,694979205 | 0,799922136 |
| b3399 | 669 | 0,52 | 0,073757871 | 6,133220372 | 0,151993952 | 0,696637103 | 0,800109507 |
| b2181 | 345 | 0,49 | 0,099171808 | 5,623379882 | 0,15170339 | 0,696912824 | 0,800109507 |

| | | | | | | | |
|---|---|---|---|---|---|---|---|
| b0366 | 768 | 0,56 | -0,256784463 | 1,620796237 | 0,151410852 | 0,697190727 | 0,800109507 |
| b0259 | 1017 | 0,55 | 0,504533947 | 0,410376926 | 0,151312542 | 0,697284189 | 0,800109507 |
| b0552 | 1017 | 0,55 | 0,504533947 | 0,410376926 | 0,151312542 | 0,697284189 | 0,800109507 |
| b0656 | 1017 | 0,55 | 0,504533947 | 0,410376926 | 0,151312542 | 0,697284189 | 0,800109507 |
| b1331 | 1017 | 0,55 | 0,504533947 | 0,410376926 | 0,151312542 | 0,697284189 | 0,800109507 |
| b2030 | 1017 | 0,55 | 0,504533947 | 0,410376926 | 0,151312542 | 0,697284189 | 0,800109507 |
| b2192 | 1017 | 0,55 | 0,504533947 | 0,410376926 | 0,151312542 | 0,697284189 | 0,800109507 |
| b2982 | 1017 | 0,55 | 0,504533947 | 0,410376926 | 0,151312542 | 0,697284189 | 0,800109507 |
| b3218 | 1017 | 0,55 | 0,504533947 | 0,410376926 | 0,151312542 | 0,697284189 | 0,800109507 |
| b3505 | 1017 | 0,55 | 0,504533947 | 0,410376926 | 0,151312542 | 0,697284189 | 0,800109507 |
| b2774 | 786 | 0,46 | 0,263877784 | 1,525170045 | 0,150613928 | 0,697949356 | 0,800667779 |
| b3426 | 1506 | 0,55 | -0,111223121 | 6,682176185 | 0,149746593 | 0,698777641 | 0,801370618 |
| b3085 | 504 | 0,51 | 0,127255697 | 3,98884219 | 0,149598207 | 0,698919622 | 0,801370618 |
| b3465 | 597 | 0,54 | 0,092274694 | 4,76990164 | 0,149129886 | 0,699368261 | 0,801679935 |
| b2810 | 1206 | 0,56 | 0,076166928 | 5,899956801 | 0,148381984 | 0,700086412 | 0,802297954 |
| b4703 | 90 | 0,42 | 0,235207513 | 1,507428318 | 0,147940753 | 0,700511067 | 0,80254456 |
| b3523 | 1323 | 0,56 | 0,114285197 | 3,954718745 | 0,147786476 | 0,700659719 | 0,80254456 |
| b0730 | 723 | 0,48 | -0,101426148 | 4,021793315 | 0,146655682 | 0,701752017 | 0,803240807 |
| b2010 | 1167 | 0,49 | 0,097316063 | 4,494942569 | 0,146508209 | 0,701894825 | 0,803240807 |
| b3848 | 615 | 0,56 | -0,090883423 | 6,217955862 | 0,14643546 | 0,701965303 | 0,803240807 |
| b1029 | 987 | 0,41 | -0,311863298 | 1,133733513 | 0,146415714 | 0,701984436 | 0,803240807 |
| b0340 | 471 | 0,51 | -0,139933982 | 2,5309864 | 0,146030817 | 0,702357682 | 0,803460578 |
| b0455 | 114 | 0,64 | 0,420705376 | 0,710329757 | 0,145848136 | 0,70253503 | 0,803460578 |
| b0718 | 2448 | 0,5 | 0,198970229 | 2,34132169 | 0,145542739 | 0,702831796 | 0,803506573 |
| b2133 | 1716 | 0,52 | -0,0814045 | 9,050190852 | 0,145316435 | 0,703051934 | 0,803506573 |
| b4245 | 936 | 0,54 | 0,209777941 | 6,976495195 | 0,145253623 | 0,70311307 | 0,803506573 |
| b3047 | 750 | 0,39 | 0,255211324 | 1,578456176 | 0,144080154 | 0,704258014 | 0,80429846 |
| b1128 | 1122 | 0,54 | 0,074317439 | 8,01056799 | 0,144040795 | 0,704296508 | 0,80429846 |
| b4455 | 153 | 0,35 | 0,498380934 | 0,410376926 | 0,143991869 | 0,704344368 | 0,80429846 |
| b4407 | 201 | 0,56 | 0,296656132 | 1,348319566 | 0,143712046 | 0,704618272 | 0,80440629 |
| b3771 | 1851 | 0,56 | 0,081936085 | 6,381880779 | 0,142805313 | 0,705507927 | 0,804475297 |
| b1394 | 789 | 0,54 | -0,282435469 | 4,737908996 | 0,142794245 | 0,705518806 | 0,804475297 |
| b4615 | 439 | 0,33 | 0,414724793 | 0,661602276 | 0,142534307 | 0,705774455 | 0,804475297 |
| b0852 | 903 | 0,56 | 0,070270038 | 5,821760859 | 0,142529019 | 0,705779658 | 0,804475297 |
| b0200 | 576 | 0,5 | -0,090216046 | 6,221074824 | 0,142478566 | 0,70582931 | 0,804475297 |
| b2345 | 1059 | 0,36 | 0,182236874 | 2,14038667 | 0,142058735 | 0,706242869 | 0,804475297 |
| b0403 | 1815 | 0,55 | 0,094147011 | 5,577255963 | 0,141755896 | 0,706541616 | 0,804475297 |
| b0055 | 816 | 0,53 | -0,084673631 | 5,211522915 | 0,141632779 | 0,706663175 | 0,804475297 |
| b1506 | 203 | 0,46 | 0,393663106 | 0,754962849 | 0,140749294 | 0,707537246 | 0,804475297 |
| b0761 | 789 | 0,53 | 0,082733829 | 5,38635965 | 0,140507172 | 0,707777334 | 0,804475297 |
| b3205 | 855 | 0,52 | 0,070724146 | 7,790856454 | 0,140352503 | 0,707930828 | 0,804475297 |
| b2145 | 240 | 0,43 | 0,217920802 | 2,002334448 | 0,139949645 | 0,708331078 | 0,804475297 |
| b0467 | 528 | 0,59 | -0,100850362 | 4,839398352 | 0,139844509 | 0,70843564 | 0,804475297 |
| b0851 | 723 | 0,53 | 0,077997115 | 6,370935994 | 0,139652378 | 0,70862684 | 0,804475297 |
| b2661 | 1449 | 0,58 | -0,129811128 | 10,15618334 | 0,138844591 | 0,709432357 | 0,804475297 |
| b4519 | 165 | 0,47 | 0,391041053 | 0,583237089 | 0,138842077 | 0,709434868 | 0,804475297 |
| b4695 | 78 | 0,64 | 0,48797292 | 0,4220575 | 0,138794953 | 0,709481943 | 0,804475297 |
| b0206 | 77 | 0,64 | 0,487808305 | 0,410376926 | 0,13879486 | 0,709482036 | 0,804475297 |
| b0216 | 77 | 0,64 | 0,487808305 | 0,410376926 | 0,13879486 | 0,709482036 | 0,804475297 |
| b1229 | 90 | 0,64 | 0,487808305 | 0,410376926 | 0,13879486 | 0,709482036 | 0,804475297 |
| b2590 | 76 | 0,64 | 0,487808305 | 0,410376926 | 0,13879486 | 0,709482036 | 0,804475297 |
| b3757 | 76 | 0,64 | 0,487808305 | 0,410376926 | 0,13879486 | 0,709482036 | 0,804475297 |
| b3760 | 77 | 0,64 | 0,487808305 | 0,410376926 | 0,13879486 | 0,709482036 | 0,804475297 |
| b3969 | 76 | 0,64 | 0,487808305 | 0,410376926 | 0,13879486 | 0,709482036 | 0,804475297 |
| b4008 | 76 | 0,64 | 0,487808305 | 0,410376926 | 0,13879486 | 0,709482036 | 0,804475297 |
| b4635 | 45 | 0,64 | 0,487808305 | 0,410376926 | 0,13879486 | 0,709482036 | 0,804475297 |
| b0293 | 588 | 0,53 | 0,128333281 | 3,404649803 | 0,138751903 | 0,709524955 | 0,804475297 |
| b2879 | 1329 | 0,53 | 0,245528584 | 2,343459213 | 0,13806215 | 0,710215149 | 0,805054197 |
| b1813 | 579 | 0,55 | 0,09058321 | 4,839280147 | 0,137624993 | 0,7106536 | 0,8050796 |
| b0123 | 1551 | 0,55 | 0,077510928 | 7,190540346 | 0,137369025 | 0,710910695 | 0,8050796 |
| b2853 | 219 | 0,34 | 0,359886307 | 1,018015445 | 0,137350552 | 0,710929259 | 0,8050796 |
| b0400 | 1296 | 0,53 | -0,119435013 | 4,700758428 | 0,136883552 | 0,711399052 | 0,8050796 |
| b2075 | 3123 | 0,57 | -0,089001427 | 5,318180373 | 0,136780832 | 0,711502509 | 0,8050796 |
| b2083 | 213 | 0,48 | -0,260082518 | 1,271726786 | 0,136766521 | 0,711516926 | 0,8050796 |
| b1947 | 366 | 0,55 | 0,342850364 | 0,82323717 | 0,136653613 | 0,711630701 | 0,8050796 |
| b0563 | 183 | 0,47 | 0,305336914 | 1,611628608 | 0,136610104 | 0,711674559 | 0,8050796 |
| b2650 | 1374 | 0,34 | 0,320822635 | 0,957815314 | 0,135305017 | 0,712993807 | 0,806188406 |
| b2102 | 819 | 0,48 | 0,123113567 | 3,477555025 | 0,135284635 | 0,713014467 | 0,806188406 |
| b0996 | 1173 | 0,51 | 0,223071912 | 2,012406034 | 0,134760416 | 0,713546453 | 0,806586432 |
| b2491 | 2013 | 0,53 | 0,118070303 | 3,695215679 | 0,133733135 | 0,71459237 | 0,807565053 |
| b3526 | 930 | 0,57 | -0,088187836 | 6,385810419 | 0,133031863 | 0,715308981 | 0,808171125 |
| b2105 | 273 | 0,48 | 0,146237133 | 2,804251327 | 0,132610991 | 0,715740087 | 0,808454403 |
| b2695 | 93 | 0,65 | 0,145089507 | 4,613841816 | 0,131914555 | 0,716455163 | 0,809058212 |
| b0232 | 294 | 0,42 | 0,139716235 | 3,770290769 | 0,130806251 | 0,717597551 | 0,810041809 |
| b4210 | 966 | 0,56 | 0,131626199 | 3,805391016 | 0,13071907 | 0,717687645 | 0,810041809 |
| b1910 | 74 | 0,57 | 0,378944119 | 0,611356134 | 0,130200083 | 0,718224676 | 0,810443857 |
| b0375 | 624 | 0,39 | -0,20806911 | 1,536617337 | 0,130022288 | 0,718408931 | 0,810447729 |
| b4055 | 714 | 0,49 | 0,112626467 | 6,455989061 | 0,129203086 | 0,719259742 | 0,811192668 |

| | | | | | | | |
|---|---|---|---|---|---|---|---|
| b2095 | 1263 | 0,49 | 0,102452839 | 13,07188015 | 0,129038305 | 0,719431248 | 0,811192668 |
| b0702 | 327 | 0,31 | -0,424357729 | 0,972123685 | 0,126521564 | 0,72206622 | 0,813958953 |
| b0353 | 1212 | 0,57 | -0,140236006 | 3,716777292 | 0,124236747 | 0,724484062 | 0,816479147 |
| b4197 | 855 | 0,57 | -0,130745629 | 3,716276271 | 0,124029881 | 0,724704203 | 0,81652193 |
| b4510 | 183 | 0,57 | 0,459035676 | 0,410376926 | 0,123858412 | 0,724886831 | 0,816522437 |
| b1773 | 837 | 0,45 | 0,177423576 | 2,837132976 | 0,123631998 | 0,725128199 | 0,816589092 |
| b4528 | 186 | 0,57 | 0,456181005 | 0,410376926 | 0,122362188 | 0,726486495 | 0,817913206 |
| b3959 | 777 | 0,55 | 0,11791381 | 5,024415344 | 0,122011917 | 0,726862565 | 0,818106862 |
| b0451 | 1287 | 0,56 | 0,094117185 | 4,694559208 | 0,121862131 | 0,727023568 | 0,818106862 |
| b2858 | 707 | 0,31 | -0,35935454 | 0,80718238 | 0,121541543 | 0,727368538 | 0,818289606 |
| b3096 | 384 | 0,54 | -0,112161256 | 4,471542269 | 0,12102959 | 0,727920488 | 0,818705051 |
| b2689 | 429 | 0,49 | -0,072311579 | 5,74001655 | 0,119361346 | 0,729728196 | 0,820362032 |
| b4094 | 558 | 0,6 | 0,17967545 | 2,174770733 | 0,119332296 | 0,729759799 | 0,820362032 |
| b0909 | 765 | 0,5 | -0,13934057 | 2,898091387 | 0,118967194 | 0,730157361 | 0,820603133 |
| b4524 | 1082 | 0,49 | 0,261151085 | 1,468905929 | 0,118017624 | 0,731194561 | 0,82153495 |
| b0717 | 729 | 0,48 | -0,211695638 | 2,033404506 | 0,117872884 | 0,731353068 | 0,82153495 |
| b0107 | 1386 | 0,53 | -0,129374005 | 4,071142751 | 0,116401975 | 0,732970084 | 0,82313516 |
| b3802 | 1197 | 0,55 | 0,072834637 | 8,248888272 | 0,116243548 | 0,733144926 | 0,82313516 |
| b3443 | 1272 | 0,28 | 0,332426962 | 0,859153919 | 0,115921772 | 0,733500454 | 0,823137839 |
| b2032 | 1119 | 0,32 | -0,107966265 | 6,730152972 | 0,115908961 | 0,73351462 | 0,823137839 |
| b3691 | 1293 | 0,53 | -0,1635308 | 2,80666878 | 0,115543672 | 0,733918918 | 0,823385379 |
| b1809 | 345 | 0,52 | 0,081203446 | 6,735421918 | 0,11532137 | 0,734165308 | 0,823455683 |
| b2549 | 3282 | 0,55 | -0,099613115 | 3,880616742 | 0,114225663 | 0,73538363 | 0,824615819 |
| b4031 | 1476 | 0,5 | 0,114710971 | 3,800443093 | 0,113717795 | 0,73595054 | 0,825045102 |
| b2408 | 765 | 0,45 | -0,128386294 | 3,686318926 | 0,112483894 | 0,737333787 | 0,826178677 |
| b2642 | 1704 | 0,31 | -0,106589814 | 4,386174582 | 0,11221177 | 0,737639982 | 0,826178677 |
| b3971 | 120 | 0,63 | 0,437596526 | 0,410376926 | 0,112159333 | 0,737699032 | 0,826178677 |
| b4010 | 120 | 0,63 | 0,437596526 | 0,410376926 | 0,112159333 | 0,737699032 | 0,826178677 |
| b1626 | 441 | 0,49 | 0,155480428 | 4,707534503 | 0,111818537 | 0,738083181 | 0,826402402 |
| b3921 | 441 | 0,49 | 0,10632259 | 3,853682291 | 0,111045225 | 0,738957285 | 0,827174464 |
| b2256 | 891 | 0,53 | -0,109226939 | 4,901552344 | 0,110179312 | 0,739940084 | 0,828067779 |
| b4218 | 1344 | 0,51 | -0,083402527 | 5,93780125 | 0,109790994 | 0,740382212 | 0,828355734 |
| b0254 | 894 | 0,48 | -0,107195061 | 3,355710787 | 0,109074284 | 0,741200521 | 0,829046553 |
| b4362 | 540 | 0,57 | 0,09910594 | 5,152530207 | 0,108926515 | 0,741369608 | 0,829046553 |
| b1802 | 1125 | 0,52 | 0,144961641 | 2,921922668 | 0,108616494 | 0,741724769 | 0,829132231 |
| b2901 | 1440 | 0,51 | 0,070221403 | 7,867094996 | 0,108536755 | 0,741816208 | 0,829132231 |
| b1712 | 300 | 0,5 | -0,080870852 | 9,806352034 | 0,108292047 | 0,742097057 | 0,829239344 |
| b1396 | 423 | 0,52 | -0,258513201 | 3,830333562 | 0,107340917 | 0,743192013 | 0,830255883 |
| b4352 | 957 | 0,55 | 0,056953938 | 6,329952957 | 0,106974058 | 0,743615782 | 0,830519199 |
| b1897 | 801 | 0,51 | -0,080641013 | 7,908213216 | 0,106700614 | 0,743932168 | 0,830519199 |
| b0952 | 564 | 0,52 | -0,085753933 | 5,681646048 | 0,106656183 | 0,743983619 | 0,830519199 |
| b4707 | 252 | 0,58 | 0,424050312 | 0,410376926 | 0,106307708 | 0,744387563 | 0,830763212 |
| b2775 | 1278 | 0,5 | 0,108528545 | 3,005033998 | 0,106069323 | 0,744664317 | 0,830856309 |
| b3661 | 819 | 0,47 | 0,080548026 | 5,253071982 | 0,105916658 | 0,744841733 | 0,830856309 |
| b2571 | 957 | 0,51 | -0,088761951 | 7,513450025 | 0,104763768 | 0,746186132 | 0,832148854 |
| b0530 | 543 | 0,45 | 0,189054986 | 1,810859458 | 0,104244664 | 0,746794134 | 0,832619729 |
| b0320 | 1548 | 0,56 | -0,121640071 | 2,87745445 | 0,10406389 | 0,747006259 | 0,832649105 |
| b4035 | 1116 | 0,55 | -0,180763632 | 8,838909717 | 0,103159268 | 0,748070832 | 0,833628411 |
| b2405 | 885 | 0,51 | 0,090433707 | 4,376672283 | 0,10276214 | 0,748539803 | 0,833943674 |
| b1889 | 927 | 0,53 | 0,191995593 | 1,797769317 | 0,101949357 | 0,749502753 | 0,834628657 |
| b1160 | 324 | 0,34 | 0,416186051 | 0,572203825 | 0,101928874 | 0,749527074 | 0,834628657 |
| b0041 | 771 | 0,53 | 0,160843942 | 2,152513228 | 0,101095119 | 0,750519385 | 0,835526052 |
| b3240 | 1968 | 0,53 | 0,088572854 | 3,996322708 | 0,10072227 | 0,750964598 | 0,835564354 |
| b2681 | 1178 | 0,54 | 0,101810362 | 3,315500238 | 0,100692397 | 0,751000307 | 0,835564354 |
| b3220 | 1128 | 0,49 | 0,125445583 | 2,816445099 | 0,100016039 | 0,751810387 | 0,835564354 |
| b0205 | 120 | 0,64 | 0,413279988 | 0,410376926 | 0,099975717 | 0,751858777 | 0,835564354 |
| b2588 | 120 | 0,64 | 0,413279988 | 0,410376926 | 0,099975717 | 0,751858777 | 0,835564354 |
| b3274 | 120 | 0,64 | 0,413279988 | 0,410376926 | 0,099975717 | 0,751858777 | 0,835564354 |
| b3759 | 120 | 0,64 | 0,413279988 | 0,410376926 | 0,099975717 | 0,751858777 | 0,835564354 |
| b2455 | 1404 | 0,57 | 0,108106951 | 3,048736258 | 0,098913212 | 0,753137741 | 0,836778224 |
| b0562 | 655 | 0,41 | -0,11773715 | 3,354108887 | 0,098187476 | 0,754015672 | 0,837546033 |
| b1597 | 309 | 0,51 | -0,130592577 | 3,309168262 | 0,097712742 | 0,754591892 | 0,837802618 |
| b1196 | 441 | 0,41 | -0,171064517 | 2,778739599 | 0,097689189 | 0,75462052 | 0,837802618 |
| b2386 | 1248 | 0,54 | 0,218698847 | 1,911104599 | 0,096516948 | 0,756050161 | 0,839181976 |
| b0714 | 792 | 0,52 | 0,10546216 | 4,635398289 | 0,095782533 | 0,756950694 | 0,839973511 |
| b1663 | 1374 | 0,53 | 0,065673257 | 6,822692414 | 0,095325321 | 0,757513235 | 0,840389684 |
| b3902 | 825 | 0,56 | 0,163663479 | 2,051436777 | 0,094841233 | 0,758110458 | 0,840836836 |
| b1613 | 1176 | 0,51 | -0,066478953 | 7,744072598 | 0,094694754 | 0,758291497 | 0,840836836 |
| b3212 | 4461 | 0,57 | 0,183526147 | 8,441744403 | 0,094332825 | 0,758739482 | 0,84112549 |
| b3513 | 1158 | 0,55 | -0,122017199 | 8,140225351 | 0,094080527 | 0,759052325 | 0,841264224 |
| b1518 | 291 | 0,46 | 0,172054119 | 5,287311103 | 0,092813669 | 0,760630176 | 0,842804561 |
| b0479 | 1221 | 0,52 | 0,089227702 | 4,180864236 | 0,091512497 | 0,762263064 | 0,844405104 |
| b1734 | 1353 | 0,47 | -0,099455451 | 3,521945115 | 0,091268208 | 0,762571041 | 0,844537536 |
| b0011 | 714 | 0,51 | -0,110573779 | 2,852819881 | 0,090281282 | 0,763819867 | 0,845711622 |
| b4281 | 231 | 0,48 | -0,254330869 | 1,590560044 | 0,089822215 | 0,764403295 | 0,846148572 |
| b2370 | 3594 | 0,41 | 0,084906085 | 6,340794435 | 0,089626887 | 0,764652028 | 0,846214912 |
| b1616 | 1374 | 0,52 | -0,166553236 | 2,575118932 | 0,088349659 | 0,766285801 | 0,847813616 |
| b0682 | 327 | 0,48 | -0,138044083 | 2,716507449 | 0,087643769 | 0,767194263 | 0,848609251 |

| | | | | | | | |
|---|---|---|---|---|---|---|---|
| b4029 | 2097 | 0,54 | 0,10032723 | 4,441244251 | 0,087382506 | 0,76753151 | 0,848765327 |
| b1166 | 267 | 0,35 | 0,147188805 | 4,260817322 | 0,087241225 | 0,767714108 | 0,848765327 |
| b1408 | 606 | 0,47 | 0,361152106 | 0,562121748 | 0,086948116 | 0,768093451 | 0,848975301 |
| b1284 | 750 | 0,49 | -0,084268414 | 5,012250163 | 0,086780598 | 0,768310564 | 0,849005905 |
| b4063 | 465 | 0,52 | 0,113188034 | 3,391076963 | 0,08615111 | 0,769128465 | 0,849700217 |
| b2126 | 1686 | 0,51 | -0,068713373 | 5,831664698 | 0,085393003 | 0,7701178 | 0,850556155 |
| b4572 | 1260 | 0,57 | 0,117934941 | 2,756147924 | 0,085266934 | 0,770282783 | 0,850556155 |
| b0422 | 243 | 0,55 | -0,095484715 | 5,323480932 | 0,08491994 | 0,770737568 | 0,850848714 |
| b3132 | 1281 | 0,55 | -0,093728215 | 3,929708232 | 0,084031847 | 0,771906152 | 0,851928927 |
| b4081 | 2052 | 0,55 | 0,110782924 | 3,124681327 | 0,083687313 | 0,772361304 | 0,851965702 |
| b3957 | 1152 | 0,52 | -0,062894963 | 6,48721797 | 0,083646831 | 0,77241485 | 0,851965702 |
| b4173 | 1281 | 0,54 | 0,063534973 | 9,011789843 | 0,083575125 | 0,772509731 | 0,851965702 |
| b4130 | 1458 | 0,51 | 0,105333477 | 3,303563242 | 0,083022199 | 0,773242843 | 0,852553156 |
| b3238 | 264 | 0,48 | -0,118551999 | 4,045383749 | 0,082886752 | 0,773422832 | 0,852553156 |
| b4618 | 90 | 0,42 | -0,130865642 | 2,321782415 | 0,082618898 | 0,77377924 | 0,852736306 |
| b0801 | 1086 | 0,56 | 0,07241581 | 7,205666441 | 0,082338928 | 0,774152439 | 0,852937864 |
| b2314 | 663 | 0,57 | 0,050180778 | 6,276288321 | 0,081855708 | 0,774798187 | 0,853439537 |
| b4447 | 143 | 0,48 | -0,099560113 | 3,974048321 | 0,081000983 | 0,775945465 | 0,854415638 |
| b2184 | 1761 | 0,53 | 0,08107724 | 4,704216153 | 0,080896794 | 0,776085762 | 0,854415638 |
| b4121 | 630 | 0,55 | -0,10403846 | 3,365164325 | 0,0806592 | 0,776406064 | 0,854415638 |
| b4474 | 831 | 0,55 | -0,102816257 | 2,792491609 | 0,080628954 | 0,776446875 | 0,854415638 |
| b0811 | 747 | 0,49 | -0,06744553 | 8,660532613 | 0,079739207 | 0,777651141 | 0,855465818 |
| b3119 | 219 | 0,29 | 0,33630843 | 0,530905159 | 0,079642142 | 0,777782956 | 0,855465818 |
| b1420 | 168 | 0,51 | -0,105490569 | 3,577865334 | 0,079351423 | 0,778178274 | 0,855473388 |
| b4612 | 156 | 0,33 | 0,310144823 | 0,499612806 | 0,079257707 | 0,778305875 | 0,855473388 |
| b1168 | 1524 | 0,45 | -0,142900351 | 3,087445171 | 0,079216179 | 0,778362444 | 0,855473388 |
| b1293 | 966 | 0,51 | -0,067456935 | 5,710367565 | 0,079029408 | 0,77861706 | 0,855543433 |
| b4611 | 142 | 0,46 | -0,096104775 | 3,836589063 | 0,077727812 | 0,780400555 | 0,857292962 |
| b1698 | 939 | 0,54 | -0,113631047 | 3,20695984 | 0,076977455 | 0,781436043 | 0,858220129 |
| b3521 | 900 | 0,53 | -0,106600358 | 3,782463948 | 0,076327278 | 0,782337686 | 0,8588516 |
| b4446 | 140 | 0,47 | 0,139130342 | 2,692019674 | 0,076040859 | 0,782736195 | 0,8588516 |
| b1913 | 1833 | 0,52 | 0,04809991 | 7,34917587 | 0,075996683 | 0,78279773 | 0,8588516 |
| b0399 | 690 | 0,54 | -0,067811521 | 4,858423386 | 0,075950038 | 0,782862727 | 0,8588516 |
| b0050 | 378 | 0,52 | -0,059390254 | 6,364420178 | 0,07587371 | 0,782969129 | 0,8588516 |
| b4038 | 1581 | 0,33 | 0,182622579 | 1,25597849 | 0,075701453 | 0,783209473 | 0,85890503 |
| b4142 | 294 | 0,49 | -0,137480769 | 8,934161799 | 0,075441864 | 0,783572222 | 0,858946788 |
| b2805 | 732 | 0,48 | 0,064920638 | 6,581444407 | 0,075396106 | 0,783636234 | 0,858946788 |
| b0623 | 210 | 0,44 | -0,086647575 | 10,25180498 | 0,075263054 | 0,783822482 | 0,858946788 |
| b4260 | 1512 | 0,55 | 0,058289903 | 9,421889618 | 0,074834906 | 0,784423013 | 0,859394756 |
| b3843 | 1494 | 0,54 | 0,052941763 | 8,495446009 | 0,074443096 | 0,784974194 | 0,85939747 |
| b2024 | 738 | 0,57 | 0,079940774 | 5,680832992 | 0,074435063 | 0,784985512 | 0,85939747 |
| b2346 | 756 | 0,52 | 0,050300407 | 7,403948525 | 0,074424266 | 0,785000723 | 0,85939747 |
| b0870 | 1002 | 0,56 | -0,052628179 | 7,432470054 | 0,073919125 | 0,785713738 | 0,859929653 |
| b2925 | 1080 | 0,51 | 0,074181063 | 11,34727256 | 0,073808269 | 0,785870562 | 0,859929653 |
| b3579 | 987 | 0,51 | -0,115185898 | 2,365321887 | 0,073469028 | 0,786351264 | 0,86014778 |
| b1700 | 294 | 0,48 | -0,206278366 | 0,979319435 | 0,073396826 | 0,786453726 | 0,86014778 |
| b1691 | 1266 | 0,45 | 0,242750605 | 1,205744971 | 0,073070615 | 0,786917335 | 0,860444863 |
| b2467 | 576 | 0,49 | -0,06509342 | 4,764253925 | 0,072553831 | 0,787654063 | 0,860658767 |
| b4506 | 141 | 0,42 | -0,302153019 | 0,670264745 | 0,072547268 | 0,787663436 | 0,860658767 |
| b2016 | 825 | 0,52 | -0,050951532 | 7,558792198 | 0,072529347 | 0,787689037 | 0,860658767 |
| b1327 | 921 | 0,48 | 0,084788745 | 4,335003442 | 0,0721791 | 0,788190041 | 0,860996287 |
| b3928 | 246 | 0,48 | -0,055790194 | 7,635078861 | 0,071871854 | 0,788630608 | 0,861062967 |
| b2159 | 858 | 0,52 | 0,049476354 | 6,612866653 | 0,071849115 | 0,788663255 | 0,861062967 |
| b4682 | 141 | 0,24 | -0,345849296 | 0,444471786 | 0,071734821 | 0,78882743 | 0,861062967 |
| b3460 | 1104 | 0,54 | -0,075191997 | 4,246030319 | 0,071531833 | 0,789119352 | 0,861142664 |
| b1469 | 1389 | 0,49 | -0,099317673 | 6,987640176 | 0,071416989 | 0,789284709 | 0,861142664 |
| b3508 | 648 | 0,46 | -0,089658454 | 6,992334134 | 0,070962198 | 0,789940939 | 0,86164889 |
| b3252 | 1941 | 0,51 | -0,056736132 | 6,84674245 | 0,070696747 | 0,790325005 | 0,861858071 |
| b2125 | 720 | 0,49 | 0,068965257 | 5,091174412 | 0,068704578 | 0,793232334 | 0,864651737 |
| b1472 | 279 | 0,42 | 0,322276568 | 0,488017012 | 0,068677178 | 0,793272634 | 0,864651737 |
| b4092 | 759 | 0,59 | -0,079811051 | 4,172260891 | 0,068147288 | 0,794053676 | 0,865292627 |
| b4080 | 1467 | 0,56 | -0,177359414 | 1,786189376 | 0,067999435 | 0,794272185 | 0,865320353 |
| b2808 | 918 | 0,49 | 0,061460041 | 4,997798149 | 0,067019946 | 0,795726203 | 0,866502583 |
| b2895 | 522 | 0,51 | 0,047645598 | 6,247936746 | 0,067008001 | 0,795744005 | 0,866502583 |
| b0207 | 804 | 0,46 | -0,060161398 | 5,336167602 | 0,065431964 | 0,798107804 | 0,868865479 |
| b0892 | 1344 | 0,54 | 0,053352684 | 6,287170244 | 0,065107143 | 0,798598742 | 0,86918882 |
| b0840 | 759 | 0,51 | -0,064587253 | 4,954516099 | 0,064955393 | 0,798828545 | 0,869227856 |
| b4282 | 249 | 0,49 | 0,208375903 | 0,971014599 | 0,064689289 | 0,799232211 | 0,869456012 |
| b1736 | 351 | 0,5 | 0,124179664 | 2,874905826 | 0,063717078 | 0,800714573 | 0,870857247 |
| b4195 | 465 | 0,54 | 0,103078633 | 2,97262061 | 0,063513886 | 0,801025903 | 0,870984497 |
| b4656 | 261 | 0,51 | 0,066899521 | 4,928091858 | 0,063201759 | 0,801505177 | 0,871294252 |
| b4428 | 150 | 0,53 | -0,095943585 | 3,480426385 | 0,062844881 | 0,802054711 | 0,871680217 |
| b3241 | 933 | 0,54 | 0,081385184 | 3,680651003 | 0,062246285 | 0,802980187 | 0,872378059 |
| b3638 | 669 | 0,48 | -0,062865806 | 4,211618603 | 0,062162392 | 0,803110269 | 0,872378059 |
| b0674 | 1665 | 0,52 | 0,062973594 | 7,052428135 | 0,062052546 | 0,803280734 | 0,872378059 |
| b3008 | 1188 | 0,51 | -0,06391632 | 5,439808317 | 0,061765263 | 0,803727315 | 0,872651605 |
| b4499 | 3796 | 0,5 | 0,096062618 | 3,819840067 | 0,061088333 | 0,804783982 | 0,873587263 |
| b0990 | 213 | 0,44 | 0,150857122 | 1,70055907 | 0,060807341 | 0,805224426 | 0,873853723 |

| | | | | | | | |
|---|---|---|---|---|---|---|---|
| b3797 | 77 | 0,53 | 0,302345353 | 0,563142894 | 0,060449243 | 0,805787297 | 0,874252884 |
| b4590 | 258 | 0,44 | 0,301398767 | 0,68022837 | 0,060276833 | 0,806058928 | 0,874335943 |
| b3761 | 76 | 0,59 | 0,300993447 | 0,531478586 | 0,059743619 | 0,80690162 | 0,875038244 |
| b4172 | 309 | 0,49 | 0,049088369 | 8,961042853 | 0,059601993 | 0,807126116 | 0,875069969 |
| b3965 | 1101 | 0,51 | -0,049468588 | 7,072951457 | 0,059328391 | 0,807560611 | 0,875329301 |
| b1602 | 1389 | 0,53 | -0,058137852 | 9,097732695 | 0,058443805 | 0,808972693 | 0,876647875 |
| b4553 | 300 | 0,48 | 0,123176558 | 3,068477374 | 0,057648522 | 0,810251908 | 0,877821864 |
| b3097 | 369 | 0,49 | -0,053561806 | 9,019576932 | 0,057075291 | 0,811179749 | 0,878614701 |
| b4621 | 204 | 0,28 | -0,307091583 | 0,432868771 | 0,056774176 | 0,811669112 | 0,878932341 |
| b3943 | 906 | 0,54 | -0,082549698 | 3,549493581 | 0,056395909 | 0,812285807 | 0,879387678 |
| b1599 | 330 | 0,52 | 0,098667573 | 3,00151813 | 0,056264306 | 0,812500874 | 0,879408093 |
| b1267 | 621 | 0,48 | -0,048156643 | 6,720575462 | 0,055232037 | 0,814197113 | 0,881031255 |
| b3048 | 1065 | 0,4 | 0,084709519 | 3,641629694 | 0,055050807 | 0,814496634 | 0,881056492 |
| b4576 | 504 | 0,53 | 0,101266019 | 3,19716441 | 0,054980127 | 0,814613588 | 0,881056492 |
| b1352 | 222 | 0,42 | 0,287868299 | 0,718533939 | 0,054858655 | 0,814814775 | 0,881061477 |
| b1848 | 291 | 0,49 | -0,054724977 | 5,707226351 | 0,054604251 | 0,81523689 | 0,881305292 |
| b3961 | 918 | 0,55 | 0,046169431 | 7,653569566 | 0,053623172 | 0,816874514 | 0,882862689 |
| b0535 | 633 | 0,37 | 0,208350056 | 0,884420457 | 0,053440946 | 0,817180422 | 0,882980388 |
| b2360 | 825 | 0,46 | -0,135096168 | 2,318527572 | 0,05183402 | 0,819902129 | 0,885457834 |
| b1606 | 723 | 0,51 | 0,06177028 | 5,236527508 | 0,051784388 | 0,819986893 | 0,885457834 |
| b1693 | 759 | 0,5 | 0,062375149 | 4,943779098 | 0,051707485 | 0,820118319 | 0,885457834 |
| b0466 | 162 | 0,48 | 0,099919016 | 2,796995893 | 0,051622613 | 0,820263483 | 0,885457834 |
| b0550 | 363 | 0,53 | -0,260403373 | 0,727597516 | 0,051416124 | 0,820617182 | 0,885626344 |
| b4638 | 51 | 0,31 | -0,273333152 | 0,433177521 | 0,051027028 | 0,821285709 | 0,886029797 |
| b3233 | 399 | 0,54 | 0,044511852 | 7,530342378 | 0,050968563 | 0,821386392 | 0,886029797 |
| b0802 | 261 | 0,51 | -0,074638646 | 3,770880375 | 0,050380265 | 0,822402901 | 0,886772926 |
| b1393 | 768 | 0,55 | 0,169419182 | 5,019735302 | 0,050340977 | 0,822471007 | 0,886772926 |
| b3672 | 99 | 0,59 | -0,056146439 | 5,332352379 | 0,049582251 | 0,823791789 | 0,887983357 |
| b3580 | 1497 | 0,57 | 0,122978519 | 2,533593404 | 0,049349971 | 0,824198257 | 0,888172111 |
| b1181 | 462 | 0,48 | 0,060514107 | 4,679222324 | 0,049195287 | 0,824469497 | 0,888172111 |
| b4199 | 276 | 0,46 | 0,114852857 | 2,543080388 | 0,049142939 | 0,824561391 | 0,888172111 |
| b3163 | 885 | 0,48 | 0,059158367 | 9,64767828 | 0,048865118 | 0,825049952 | 0,888189681 |
| b3684 | 717 | 0,51 | 0,140742495 | 1,844445649 | 0,048809983 | 0,825147082 | 0,888189681 |
| b0596 | 747 | 0,58 | -0,097673795 | 3,669203532 | 0,048795727 | 0,825172207 | 0,888189681 |
| b4365 | 726 | 0,45 | 0,193222747 | 0,806656899 | 0,048388858 | 0,825890877 | 0,888749799 |
| b3990 | 1134 | 0,55 | 0,072429811 | 3,784945771 | 0,04760685 | 0,827281122 | 0,890032163 |
| b2503 | 2244 | 0,47 | -0,065467535 | 4,004087055 | 0,047385899 | 0,827676092 | 0,890243399 |
| b4514 | 240 | 0,38 | -0,248738107 | 0,520185518 | 0,046345589 | 0,829548822 | 0,892043623 |
| b2523 | 1284 | 0,57 | 0,049946144 | 8,10558709 | 0,045803934 | 0,830532603 | 0,892887293 |
| b3796 | 77 | 0,68 | 0,224832433 | 1,401598026 | 0,045267811 | 0,831512344 | 0,893726218 |
| b0363 | 1197 | 0,5 | -0,076773178 | 3,105833131 | 0,045056381 | 0,831900389 | 0,8937698 |
| b1072 | 660 | 0,52 | 0,087485326 | 2,756610585 | 0,045028452 | 0,831951719 | 0,8937698 |
| b4521 | 344 | 0,5 | -0,10663378 | 2,819147409 | 0,044762515 | 0,832441317 | 0,894081472 |
| b4259 | 444 | 0,54 | 0,039930137 | 6,24506186 | 0,044591859 | 0,832756301 | 0,894205496 |
| b3689 | 1248 | 0,55 | -0,049160895 | 5,92881873 | 0,044317621 | 0,83326379 | 0,894536121 |
| b4593 | 174 | 0,32 | -0,21637189 | 0,763520589 | 0,043920686 | 0,834001246 | 0,895113407 |
| b1277 | 591 | 0,5 | -0,044120885 | 6,936152793 | 0,043729648 | 0,834357412 | 0,895281283 |
| b4642 | 129 | 0,53 | -0,244703786 | 2,28658406 | 0,043357507 | 0,835053559 | 0,895651548 |
| b4361 | 738 | 0,51 | 0,041172283 | 5,585389473 | 0,043331596 | 0,835102146 | 0,895651548 |
| b4600 | 21837 | 0,47 | 0,095220773 | 2,842117325 | 0,043145145 | 0,835452212 | 0,895812635 |
| b1882 | 390 | 0,49 | -0,089862495 | 2,694844341 | 0,042916702 | 0,835882197 | 0,896059318 |
| b2276 | 1458 | 0,57 | 0,091700015 | 10,06242525 | 0,042687535 | 0,836314748 | 0,896308632 |
| b4602 | 90 | 0,5 | -0,112897786 | 2,347871209 | 0,041134892 | 0,839277768 | 0,899073991 |
| b3021 | 396 | 0,42 | -0,049978994 | 5,914213089 | 0,041125326 | 0,839296203 | 0,899073991 |
| b3584 | 741 | 0,49 | -0,075043184 | 3,519314988 | 0,040721226 | 0,840076983 | 0,89969535 |
| b3777 | 449 | 0,43 | 0,071656839 | 3,933967772 | 0,040319102 | 0,840857957 | 0,900316618 |
| b1446 | 234 | 0,54 | -0,043359062 | 5,755289367 | 0,039545201 | 0,842372455 | 0,901412523 |
| b4476 | 1341 | 0,55 | -0,060548589 | 3,93958419 | 0,039399611 | 0,842659084 | 0,901412523 |
| b0008 | 954 | 0,52 | 0,04936613 | 10,53301056 | 0,039292514 | 0,842870284 | 0,901412523 |
| b3484 | 1137 | 0,42 | -0,10551567 | 1,752192288 | 0,039027368 | 0,843394454 | 0,901412523 |
| b0410 | 348 | 0,47 | 0,059982969 | 4,446429191 | 0,039020448 | 0,843408159 | 0,901412523 |
| b2618 | 291 | 0,51 | 0,067007136 | 4,437195143 | 0,039012179 | 0,843424538 | 0,901412523 |
| b3042 | 291 | 0,49 | -0,043487765 | 5,904120332 | 0,038980812 | 0,843486681 | 0,901412523 |
| b0780 | 909 | 0,51 | 0,047507154 | 5,355968403 | 0,038978919 | 0,843490433 | 0,901412523 |
| b4155 | 978 | 0,53 | 0,055838115 | 4,559597966 | 0,038529882 | 0,844382952 | 0,90161657 |
| b3101 | 393 | 0,49 | -0,077817341 | 3,113916186 | 0,038494913 | 0,844452685 | 0,90161657 |
| b0192 | 711 | 0,52 | 0,046604847 | 5,991037998 | 0,03847935 | 0,844483728 | 0,90161657 |
| b0218 | 357 | 0,38 | 0,240263789 | 0,641947053 | 0,0384782 | 0,844486024 | 0,90161657 |
| b1926 | 366 | 0,52 | 0,145226026 | 1,253316261 | 0,038277684 | 0,8448866 | 0,90170837 |
| b3381 | 1164 | 0,55 | 0,123880883 | 2,027925193 | 0,038233818 | 0,844974376 | 0,90170837 |
| b3964 | 360 | 0,49 | -0,042085935 | 6,810418802 | 0,037757798 | 0,845930292 | 0,902513585 |
| b2731 | 2079 | 0,53 | -0,040826489 | 6,296971767 | 0,037156076 | 0,847147625 | 0,903597252 |
| b3470 | 246 | 0,54 | 0,063680102 | 3,947784315 | 0,037027035 | 0,847410014 | 0,90366207 |
| b2669 | 405 | 0,48 | -0,088577685 | 4,809743046 | 0,036539697 | 0,84840526 | 0,904508176 |
| b3676 | 348 | 0,52 | 0,069557557 | 3,741962441 | 0,036328555 | 0,848838589 | 0,904754948 |
| b2951 | 705 | 0,51 | -0,041014126 | 5,303923661 | 0,035953974 | 0,849610572 | 0,905296912 |
| b1409 | 897 | 0,44 | 0,190712159 | 0,613245279 | 0,035886038 | 0,849751029 | 0,905296912 |
| b2829 | 2247 | 0,54 | 0,03698402 | 8,551219968 | 0,035709891 | 0,850115852 | 0,905333165 |

| | | | | | | | |
|---|---|---|---|---|---|---|---|
| b3897 | 1749 | 0,5 | 0,094312554 | 2,438093649 | 0,035674607 | 0,850189043 | 0,905333165 |
| b3806 | 2547 | 0,52 | -0,039038445 | 8,64797763 | 0,035466472 | 0,850621542 | 0,905510604 |
| b1785 | 1476 | 0,38 | -0,088705486 | 3,074128515 | 0,035400099 | 0,85075974 | 0,905510604 |
| b4624 | 90 | 0,56 | 0,229957245 | 0,509944558 | 0,035235323 | 0,851103408 | 0,905661319 |
| b0929 | 1089 | 0,48 | -0,068943304 | 10,72841959 | 0,034751965 | 0,852116358 | 0,906315295 |
| b0318 | 606 | 0,45 | 0,067070901 | 3,363627065 | 0,034749087 | 0,852122412 | 0,906315295 |
| b0220 | 474 | 0,5 | -0,04399891 | 8,799354768 | 0,034534844 | 0,85257374 | 0,906580191 |
| b1922 | 720 | 0,54 | -0,104040492 | 1,900376506 | 0,033712668 | 0,854319342 | 0,908220894 |
| b2544 | 873 | 0,55 | -0,054722726 | 4,077715724 | 0,033402846 | 0,854982839 | 0,908710715 |
| b2594 | 981 | 0,53 | -0,042088914 | 7,063825902 | 0,033078287 | 0,855681317 | 0,909039266 |
| b0675 | 753 | 0,53 | 0,035855929 | 6,417093131 | 0,032988355 | 0,855875485 | 0,909039266 |
| b3271 | 759 | 0,47 | -0,068407649 | 3,26619427 | 0,032976813 | 0,855900424 | 0,909039266 |
| b1904 | 324 | 0,5 | 0,10912429 | 1,989886975 | 0,032405073 | 0,857141506 | 0,910141727 |
| b2454 | 837 | 0,59 | -0,086465568 | 2,322724939 | 0,032309129 | 0,857350877 | 0,91014842 |
| b2824 | 408 | 0,53 | 0,071443707 | 2,735502609 | 0,031977005 | 0,858078134 | 0,910704759 |
| b1760 | 273 | 0,51 | -0,083937963 | 2,717338309 | 0,031742824 | 0,85859327 | 0,910983683 |
| b0521 | 894 | 0,57 | -0,069818301 | 2,565768499 | 0,031555819 | 0,859006042 | 0,910983683 |
| b3717 | 588 | 0,45 | -0,070598079 | 3,328632828 | 0,031549817 | 0,859019311 | 0,910983683 |
| b3847 | 1332 | 0,54 | 0,0402649 | 8,806023404 | 0,031472733 | 0,859189839 | 0,910983683 |
| b1359 | 858 | 0,48 | 0,063598317 | 2,933002647 | 0,031397172 | 0,859357209 | 0,910983683 |
| b1886 | 1662 | 0,53 | 0,085254946 | 2,312137749 | 0,031277756 | 0,859622142 | 0,911049052 |
| b4551 | 201 | 0,48 | -0,055638164 | 3,865181188 | 0,031074933 | 0,860073317 | 0,911311727 |
| b4622 | 276 | 0,47 | 0,21396475 | 0,55160108 | 0,030771788 | 0,860750497 | 0,911687164 |
| b0942 | 543 | 0,47 | 0,121081857 | 1,953539114 | 0,030734305 | 0,860834467 | 0,911687164 |
| b4552 | 351 | 0,35 | -0,15172188 | 1,080148335 | 0,030496969 | 0,861367377 | 0,911879642 |
| b3291 | 411 | 0,45 | -0,038789369 | 8,623681238 | 0,0304722 | 0,861423115 | 0,911879642 |
| b4411 | 147 | 0,48 | 0,053072462 | 9,518575704 | 0,030314137 | 0,861779365 | 0,91204135 |
| b3879 | 927 | 0,48 | 0,075408584 | 2,669502683 | 0,029998232 | 0,862494241 | 0,912582433 |
| b1742 | 576 | 0,52 | 0,096832071 | 2,138331268 | 0,029869025 | 0,862787747 | 0,912677527 |
| b4095 | 1137 | 0,61 | 0,092824094 | 2,002457468 | 0,029553209 | 0,863507917 | 0,913007689 |
| b1795 | 249 | 0,46 | 0,054165164 | 8,357466132 | 0,029486635 | 0,863660232 | 0,913007689 |
| b1695 | 1152 | 0,52 | 0,119067697 | 2,008561096 | 0,029445085 | 0,863755388 | 0,913007689 |
| b2355 | 579 | 0,51 | -0,16527811 | 0,833646772 | 0,029375593 | 0,863914683 | 0,913007689 |
| b2811 | 444 | 0,55 | -0,040386984 | 5,159635857 | 0,029108718 | 0,864528254 | 0,913440744 |
| b2418 | 852 | 0,52 | -0,039632499 | 6,358029634 | 0,028884739 | 0,865045442 | 0,913771782 |
| b3657 | 1383 | 0,49 | -0,060925541 | 2,948961117 | 0,028663405 | 0,865558552 | 0,913921977 |
| b0598 | 2106 | 0,57 | -0,061257767 | 10,39429409 | 0,028647523 | 0,865595447 | 0,913921977 |
| b3944 | 618 | 0,53 | 0,107260581 | 1,612828549 | 0,028274113 | 0,866465999 | 0,91437558 |
| b0411 | 885 | 0,51 | -0,043147325 | 8,428883616 | 0,028235334 | 0,866556745 | 0,91437558 |
| b0771 | 2262 | 0,51 | -0,052562841 | 3,707872605 | 0,028188796 | 0,866665731 | 0,91437558 |
| b1456 | 2037 | 0,58 | -0,091421882 | 2,328180428 | 0,028106769 | 0,866858054 | 0,91437558 |
| b1337 | 1446 | 0,53 | 0,045176863 | 4,009816669 | 0,028027104 | 0,867045117 | 0,91437558 |
| b1558 | 213 | 0,48 | 0,126598344 | 1,840698351 | 0,027887167 | 0,867374366 | 0,914507624 |
| b2163 | 660 | 0,45 | 0,081880362 | 2,387885136 | 0,027488814 | 0,868316306 | 0,915285439 |
| b3143 | 696 | 0,44 | 0,161823551 | 1,049451216 | 0,027109892 | 0,869218831 | 0,915943329 |
| b2740 | 1365 | 0,52 | 0,063885333 | 3,36328308 | 0,027055398 | 0,869349157 | 0,915943329 |
| b3784 | 1104 | 0,49 | 0,034144362 | 6,851700133 | 0,026612358 | 0,87041376 | 0,91676287 |
| b1600 | 366 | 0,42 | 0,103263319 | 1,761039669 | 0,026561689 | 0,870536095 | 0,91676287 |
| b2050 | 1224 | 0,56 | 0,085691786 | 2,56737855 | 0,026394582 | 0,870940401 | 0,916973191 |
| b3018 | 738 | 0,49 | 0,032730889 | 5,660171806 | 0,025578812 | 0,872933244 | 0,918657634 |
| b2362 | 363 | 0,47 | 0,160559929 | 0,773057423 | 0,025571921 | 0,872950214 | 0,918657634 |
| b0191 | 423 | 0,54 | 0,044668273 | 4,557742809 | 0,025480138 | 0,873176492 | 0,918680055 |
| b1206 | 1680 | 0,55 | 0,046727108 | 5,564323008 | 0,025369121 | 0,873450743 | 0,918752929 |
| b1880 | 1149 | 0,55 | -0,108088348 | 1,581180234 | 0,02513256 | 0,874037197 | 0,919083578 |
| b3891 | 930 | 0,55 | 0,042265498 | 8,023259013 | 0,025077054 | 0,874175211 | 0,919083578 |
| b2723 | 1827 | 0,58 | 0,0683758 | 2,312070064 | 0,024810598 | 0,874839931 | 0,919566738 |
| b2922 | 741 | 0,53 | 0,034658998 | 8,353289177 | 0,024687205 | 0,875148994 | 0,919655514 |
| b1522 | 948 | 0,5 | -0,05223693 | 3,704132737 | 0,024613188 | 0,875334767 | 0,919655514 |
| b0253 | 864 | 0,57 | -0,043762487 | 4,626115591 | 0,024210853 | 0,876349609 | 0,920326739 |
| b3412 | 771 | 0,56 | -0,036991105 | 4,855494027 | 0,024017921 | 0,876839321 | 0,920326739 |
| b0957 | 1041 | 0,54 | -0,044171831 | 14,05413377 | 0,024008461 | 0,876863385 | 0,920326739 |
| b1859 | 786 | 0,52 | -0,03585574 | 4,663660784 | 0,024002832 | 0,876877706 | 0,920326739 |
| b0446 | 819 | 0,51 | 0,043433728 | 4,035998442 | 0,023954659 | 0,877000335 | 0,920326739 |
| b2273 | 717 | 0,39 | 0,117613339 | 1,288695704 | 0,02374497 | 0,877535596 | 0,920672879 |
| b2428 | 897 | 0,54 | -0,044434276 | 5,238706755 | 0,02361359 | 0,877872195 | 0,920806821 |
| b1679 | 417 | 0,49 | 0,051075609 | 5,013515073 | 0,023534942 | 0,878074153 | 0,920806821 |
| b2888 | 1449 | 0,49 | -0,074174162 | 2,247170955 | 0,023400131 | 0,878421135 | 0,920955211 |
| b1392 | 1071 | 0,53 | 0,103270279 | 5,658784769 | 0,023251697 | 0,878804364 | 0,921141525 |
| b4075 | 384 | 0,52 | 0,088387902 | 2,045062723 | 0,022480635 | 0,880815553 | 0,922933622 |
| b3557 | 522 | 0,46 | 0,073541113 | 2,441242157 | 0,022438702 | 0,880925934 | 0,922933622 |
| b1105 | 642 | 0,56 | 0,031364517 | 8,034152405 | 0,021993216 | 0,882105148 | 0,92395309 |
| b2555 | 714 | 0,54 | 0,042696192 | 4,660627926 | 0,02170101 | 0,882885267 | 0,924554151 |
| b1765 | 552 | 0,56 | -0,032220829 | 7,46146624 | 0,021590491 | 0,883181722 | 0,924648559 |
| b0545 | 552 | 0,42 | -0,05514375 | 3,529721 | 0,02144675 | 0,883568456 | 0,924743203 |
| b2193 | 648 | 0,52 | -0,030116281 | 5,926748089 | 0,021403614 | 0,88368477 | 0,924743203 |
| b3954 | 852 | 0,53 | -0,039845606 | 4,273507486 | 0,021235614 | 0,884138922 | 0,925002486 |
| b2461 | 480 | 0,5 | 0,064420523 | 2,798767378 | 0,021024159 | 0,884713162 | 0,925387256 |
| b3226 | 792 | 0,52 | 0,034771866 | 6,671872867 | 0,020932678 | 0,884962505 | 0,925432092 |

| | | | | | | | |
|---|---|---|---|---|---|---|---|
| b3781 | 330 | 0,51 | 0,031683696 | 8,882105621 | 0,02028605 | 0,886741074 | 0,927075693 |
| b1877 | 489 | 0,39 | 0,112397647 | 1,387227058 | 0,019642045 | 0,888541412 | 0,92874128 |
| b4047 | 255 | 0,29 | -0,178882114 | 0,443810318 | 0,019254162 | 0,889640304 | 0,929673081 |
| b4570 | 1596 | 0,57 | -0,095951634 | 1,635370068 | 0,018994469 | 0,890382344 | 0,930231623 |
| b0280 | 441 | 0,44 | 0,035812237 | 5,198865406 | 0,018779337 | 0,891000978 | 0,930661008 |
| b2167 | 1692 | 0,56 | -0,029706156 | 8,500983018 | 0,018641559 | 0,891399073 | 0,93085989 |
| b2563 | 381 | 0,51 | 0,038282247 | 4,677112769 | 0,018462306 | 0,891919254 | 0,93118614 |
| b2243 | 1191 | 0,54 | -0,057019249 | 2,870087059 | 0,018348926 | 0,892249605 | 0,931314097 |
| b4044 | 1380 | 0,56 | -0,038659944 | 4,818321605 | 0,018183495 | 0,892733486 | 0,931602208 |
| b1447 | 450 | 0,52 | -0,049287172 | 4,094601272 | 0,017784823 | 0,893908877 | 0,932611636 |
| b0287 | 615 | 0,45 | 0,050569776 | 5,47230614 | 0,017672413 | 0,894242708 | 0,932742802 |
| b1564 | 240 | 0,47 | 0,031618392 | 5,845165286 | 0,017543474 | 0,894626962 | 0,932926487 |
| b1557 | 216 | 0,35 | -0,087745692 | 2,031226626 | 0,017301337 | 0,895352459 | 0,933465858 |
| b2112 | 282 | 0,44 | 0,029811254 | 5,360269669 | 0,017067376 | 0,896058385 | 0,933984577 |
| b0583 | 621 | 0,48 | -0,062731408 | 2,066644423 | 0,016992282 | 0,896286004 | 0,93400462 |
| b1951 | 624 | 0,38 | -0,056162695 | 2,713716187 | 0,01681704 | 0,896819188 | 0,934230401 |
| b1150 | 183 | 0,6 | -0,155979245 | 0,682239883 | 0,016784157 | 0,896919548 | 0,934230401 |
| b2291 | 600 | 0,49 | 0,035818234 | 4,306909543 | 0,016636707 | 0,897370816 | 0,934247762 |
| b0448 | 1773 | 0,54 | -0,034818548 | 5,067834372 | 0,016604892 | 0,897468452 | 0,934247762 |
| b4205 | 402 | 0,39 | -0,051520013 | 3,24854438 | 0,016574584 | 0,897561549 | 0,934247762 |
| b2856 | 633 | 0,36 | 0,154738197 | 0,736669423 | 0,016095055 | 0,899046231 | 0,935460184 |
| b0215 | 732 | 0,5 | 0,022616987 | 6,604485945 | 0,015966413 | 0,899448332 | 0,935460184 |
| b4479 | 690 | 0,55 | 0,02717247 | 5,039502079 | 0,015893389 | 0,899677319 | 0,935460184 |
| b4655 | 126 | 0,31 | 0,163951865 | 0,499644527 | 0,015831363 | 0,899872238 | 0,935460184 |
| b2358 | 708 | 0,52 | 0,152545904 | 0,573539868 | 0,015763621 | 0,900085563 | 0,935460184 |
| b2883 | 1320 | 0,49 | -0,029767911 | 4,647881289 | 0,015737396 | 0,900168273 | 0,935460184 |
| b2409 | 927 | 0,54 | -0,041700302 | 3,867990992 | 0,015731345 | 0,900187366 | 0,935460184 |
| b0844 | 816 | 0,46 | 0,033265389 | 5,071313458 | 0,015520866 | 0,900853873 | 0,935935804 |
| b0520 | 816 | 0,57 | 0,060788715 | 2,0208475 | 0,015009739 | 0,90249177 | 0,937309917 |
| b3991 | 771 | 0,58 | -0,057484111 | 3,333029818 | 0,014977898 | 0,902594735 | 0,937309917 |
| b1137 | 666 | 0,35 | 0,056093661 | 3,04139619 | 0,01464314 | 0,903684032 | 0,938223727 |
| b0807 | 927 | 0,54 | -0,03205225 | 4,530032552 | 0,013852528 | 0,906307932 | 0,940337802 |
| b1940 | 687 | 0,57 | -0,069264108 | 1,859761388 | 0,013851128 | 0,906312645 | 0,940337802 |
| b1140 | 1128 | 0,45 | 0,036124443 | 4,034392395 | 0,013840126 | 0,906349689 | 0,940337802 |
| b0495 | 687 | 0,57 | -0,026969221 | 4,977700372 | 0,013750205 | 0,906653016 | 0,940434811 |
| b0514 | 1146 | 0,56 | 0,038119375 | 4,258674735 | 0,013655765 | 0,906972673 | 0,940548709 |
| b4604 | 165 | 0,4 | -0,110641984 | 0,710121935 | 0,012957224 | 0,909372712 | 0,942625332 |
| b1603 | 1533 | 0,52 | 0,02661245 | 9,149805568 | 0,012950597 | 0,909395791 | 0,942625332 |
| b3121 | 1188 | 0,32 | 0,12345592 | 0,901575944 | 0,012847876 | 0,90975429 | 0,942778896 |
| b0327 | 246 | 0,47 | -0,080575908 | 1,417160527 | 0,012710008 | 0,910237743 | 0,942863438 |
| b1322 | 1062 | 0,56 | -0,043519925 | 5,889666407 | 0,012704644 | 0,910256603 | 0,942863438 |
| b0769 | 1053 | 0,49 | 0,099799051 | 0,689297873 | 0,01251699 | 0,910919067 | 0,943331621 |
| b3266 | 3105 | 0,52 | 0,02876113 | 4,557804282 | 0,012235183 | 0,911923428 | 0,94415357 |
| b1302 | 1266 | 0,58 | 0,046426194 | 6,710696008 | 0,011990099 | 0,912806467 | 0,944470601 |
| b2634 | 702 | 0,57 | 0,047236906 | 2,246232975 | 0,011925918 | 0,913039221 | 0,944470601 |
| b1191 | 1737 | 0,52 | -0,026474152 | 5,038001255 | 0,011878769 | 0,913210607 | 0,944470601 |
| b3770 | 930 | 0,55 | -0,026769199 | 6,01886506 | 0,011810687 | 0,913458696 | 0,944470601 |
| b2483 | 948 | 0,54 | -0,068103775 | 1,619991572 | 0,011807073 | 0,913471887 | 0,944470601 |
| b0049 | 843 | 0,56 | 0,019554679 | 6,478218276 | 0,011801018 | 0,913493988 | 0,944470601 |
| b1158 | 555 | 0,49 | 0,083146036 | 1,053407592 | 0,011614388 | 0,914178087 | 0,944959913 |
| b3587 | 324 | 0,47 | -0,123539208 | 0,700393069 | 0,011410342 | 0,914932413 | 0,945521576 |
| b2777 | 672 | 0,52 | 0,025741041 | 6,703004864 | 0,011228913 | 0,915608878 | 0,945796096 |
| b1991 | 1080 | 0,51 | -0,022703417 | 6,110890745 | 0,01120942 | 0,915681889 | 0,945796096 |
| b3229 | 639 | 0,54 | -0,021951036 | 8,426199633 | 0,01116963 | 0,915831115 | 0,945796096 |
| b3287 | 510 | 0,46 | -0,02275671 | 7,932709072 | 0,011085233 | 0,91614853 | 0,945905946 |
| b1512 | 954 | 0,51 | 0,026536933 | 6,857883546 | 0,010860134 | 0,917001137 | 0,946568194 |
| b4025 | 1650 | 0,51 | -0,027966027 | 10,29104153 | 0,010754279 | 0,917405172 | 0,946663599 |
| b4596 | 174 | 0,48 | 0,038510872 | 3,147347294 | 0,010725337 | 0,917515992 | 0,946663599 |
| b0137 | 606 | 0,43 | -0,050502965 | 2,446688606 | 0,0105789 | 0,918079021 | 0,947026507 |
| b3413 | 684 | 0,56 | 0,024045831 | 5,445536439 | 0,010308597 | 0,919128741 | 0,947891168 |
| b1505 | 1227 | 0,45 | 0,1004923 | 1,167119405 | 0,010076625 | 0,920040746 | 0,94861344 |
| b4672 | 105 | 0,3 | 0,1299937 | 0,467128363 | 0,009940141 | 0,920582296 | 0,948614417 |
| b1454 | 618 | 0,47 | -0,02642557 | 5,219997381 | 0,009932893 | 0,920611163 | 0,948614417 |
| b0921 | 786 | 0,52 | 0,020666236 | 6,186342607 | 0,00991646 | 0,920676643 | 0,948614417 |
| b3113 | 390 | 0,53 | -0,051072383 | 3,157339898 | 0,009312842 | 0,923121037 | 0,950914384 |
| b0938 | 540 | 0,42 | 0,04546101 | 2,482729578 | 0,009126343 | 0,923892356 | 0,951322493 |
| b3917 | 990 | 0,51 | 0,036105665 | 2,824070669 | 0,009114471 | 0,923941725 | 0,951322493 |
| b3462 | 1059 | 0,53 | -0,018850837 | 7,244953106 | 0,008879916 | 0,924923828 | 0,952095695 |
| b3953 | 342 | 0,56 | 0,04243866 | 2,517232763 | 0,008781333 | 0,925340506 | 0,952095695 |
| b0796 | 672 | 0,54 | -0,023681406 | 5,318244061 | 0,008736225 | 0,92553195 | 0,952095695 |
| b0103 | 621 | 0,53 | 0,033273498 | 5,503475135 | 0,008733771 | 0,925542379 | 0,952095695 |
| b0447 | 459 | 0,52 | 0,02463492 | 4,240883203 | 0,00866537 | 0,925833674 | 0,952176807 |
| b4628 | 192 | 0,58 | -0,108105309 | 0,709365343 | 0,008038228 | 0,928560451 | 0,954762088 |
| b3502 | 1290 | 0,54 | 0,030294887 | 3,575309481 | 0,007646992 | 0,930316145 | 0,956279673 |
| b3818 | 381 | 0,33 | 0,080474569 | 1,040351031 | 0,007572902 | 0,930653691 | 0,956279673 |
| b2278 | 1842 | 0,57 | 0,043778632 | 10,6393466 | 0,007567915 | 0,930676469 | 0,956279673 |
| b3171 | 77 | 0,65 | -0,104894222 | 0,478086535 | 0,007377433 | 0,931552285 | 0,956960197 |
| b1993 | 546 | 0,51 | -0,023625649 | 4,453723604 | 0,00715836 | 0,932573763 | 0,957667572 |

| | | | | | | | |
|---|---|---|---|---|---|---|---|
| b0789 | 1242 | 0,54 | -0,023860799 | 6,275003114 | 0,00713827 | 0,932668218 | 0,957667572 |
| b1374 | 591 | 0,45 | 0,03893653 | 2,572319305 | 0,00705063 | 0,93308185 | 0,957872847 |
| b2447 | 594 | 0,52 | 0,030530578 | 3,073816434 | 0,006812712 | 0,934217983 | 0,958819556 |
| b0518 | 1668 | 0,56 | 0,050776799 | 1,779400431 | 0,006690071 | 0,93481144 | 0,95915649 |
| b3459 | 384 | 0,57 | -0,023777301 | 4,460642206 | 0,006653815 | 0,934987925 | 0,95915649 |
| b4529 | 267 | 0,55 | 0,017215715 | 6,501502719 | 0,00658898 | 0,935304742 | 0,95915649 |
| b1992 | 744 | 0,53 | 0,017717021 | 5,26884156 | 0,006569084 | 0,93540228 | 0,95915649 |
| b2170 | 1182 | 0,53 | 0,033551603 | 3,114821827 | 0,006454403 | 0,935967405 | 0,959516448 |
| b2309 | 783 | 0,49 | -0,01923899 | 7,308118912 | 0,006331015 | 0,936581104 | 0,959926026 |
| b3062 | 606 | 0,54 | 0,062800366 | 3,444802975 | 0,006218678 | 0,937145099 | 0,960060508 |
| b3534 | 753 | 0,54 | -0,027827697 | 4,237043122 | 0,006202529 | 0,937226592 | 0,960060508 |
| b1497 | 1158 | 0,39 | -0,0416707 | 2,514882494 | 0,006177142 | 0,937354927 | 0,960060508 |
| b1009 | 801 | 0,58 | -0,056311917 | 1,557179477 | 0,006051313 | 0,937994951 | 0,960496544 |
| b1377 | 1134 | 0,47 | -0,046254446 | 2,250007419 | 0,005488066 | 0,940945548 | 0,96329784 |
| b1306 | 360 | 0,5 | 0,025217627 | 4,487591072 | 0,005441843 | 0,941194316 | 0,963332479 |
| b2020 | 1305 | 0,58 | 0,022926835 | 5,977973734 | 0,00533259 | 0,941786552 | 0,963718568 |
| b3577 | 474 | 0,44 | 0,08427351 | 0,727399424 | 0,005184833 | 0,942597307 | 0,964328037 |
| b0384 | 321 | 0,48 | 0,015827837 | 6,682716443 | 0,005143902 | 0,942823945 | 0,964339781 |
| b0515 | 786 | 0,46 | 0,024698579 | 4,484123522 | 0,004960171 | 0,943852622 | 0,965171675 |
| b0764 | 690 | 0,54 | 0,016651547 | 4,838146496 | 0,00487007 | 0,94436408 | 0,965474408 |
| b1566 | 333 | 0,43 | 0,034071234 | 2,674902672 | 0,004707296 | 0,945300265 | 0,966211126 |
| b0995 | 693 | 0,52 | 0,017152655 | 4,297045525 | 0,004657866 | 0,945587774 | 0,966284634 |
| b1860 | 1011 | 0,53 | 0,012384462 | 6,385278147 | 0,004608492 | 0,945876481 | 0,966359332 |
| b0554 | 216 | 0,48 | 0,05314634 | 1,448385101 | 0,004412974 | 0,947035309 | 0,967322757 |
| b4125 | 1632 | 0,5 | 0,014052479 | 5,94675934 | 0,004193225 | 0,948368979 | 0,968464289 |
| b4078 | 690 | 0,5 | 0,013591681 | 5,943942111 | 0,004148262 | 0,948646156 | 0,968526667 |
| b2101 | 747 | 0,51 | 0,018385862 | 4,314902984 | 0,004005647 | 0,949535436 | 0,969010302 |
| b0133 | 852 | 0,53 | -0,011761081 | 8,257191887 | 0,004002972 | 0,949552264 | 0,969010302 |
| b0461 | 375 | 0,41 | 0,026028964 | 6,77309165 | 0,003940858 | 0,949944678 | 0,969190086 |
| b0258 | 1108 | 0,55 | -0,018169786 | 4,536453133 | 0,00388232 | 0,950317344 | 0,969349644 |
| b0044 | 288 | 0,57 | 0,049387889 | 1,133340627 | 0,003662947 | 0,951739665 | 0,970579563 |
| b1689 | 357 | 0,46 | -0,031988717 | 2,079912426 | 0,003558418 | 0,952432424 | 0,971065087 |
| b3377 | 1305 | 0,53 | 0,026872128 | 3,075090152 | 0,00352374 | 0,952664499 | 0,971080801 |
| b0006 | 777 | 0,5 | 0,012740878 | 5,490482011 | 0,00347199 | 0,953012965 | 0,971215123 |
| b2164 | 1251 | 0,51 | 0,017668694 | 4,424882393 | 0,003407809 | 0,953448783 | 0,971438382 |
| b3658 | 95 | 0,64 | -0,06939598 | 0,642083604 | 0,003263308 | 0,954445325 | 0,972054548 |
| b1013 | 639 | 0,51 | -0,014831812 | 4,654466522 | 0,003257291 | 0,954487297 | 0,972054548 |
| b2724 | 612 | 0,57 | -0,056262137 | 1,066285174 | 0,003196123 | 0,954916202 | 0,972270427 |
| b1275 | 975 | 0,48 | -0,010288932 | 6,700430411 | 0,003119916 | 0,955456355 | 0,972443903 |
| b4236 | 366 | 0,49 | -0,013090905 | 4,912395817 | 0,003110926 | 0,955520515 | 0,972443903 |
| b4107 | 444 | 0,52 | 0,016158275 | 4,680919676 | 0,003062776 | 0,955865718 | 0,972574381 |
| b0054 | 2355 | 0,51 | -0,011805114 | 9,71617805 | 0,00297557 | 0,956497942 | 0,972996771 |
| b1024 | 2424 | 0,47 | -0,016238815 | 4,770697952 | 0,002653732 | 0,958915638 | 0,975138037 |
| b4674 | 66 | 0,14 | -0,066040569 | 0,410376926 | 0,002564427 | 0,959612252 | 0,975138037 |
| b4677 | 66 | 0,2 | -0,066040569 | 0,410376926 | 0,002564427 | 0,959612252 | 0,975138037 |
| b4683 | 81 | 0,26 | -0,066040569 | 0,410376926 | 0,002564427 | 0,959612252 | 0,975138037 |
| b2920 | 1479 | 0,53 | 0,016245135 | 3,300189517 | 0,002554461 | 0,959690736 | 0,975138037 |
| b2580 | 690 | 0,52 | -0,009398601 | 5,525674079 | 0,002417906 | 0,960782062 | 0,975989238 |
| b4110 | 879 | 0,57 | 0,02177453 | 2,672922674 | 0,002378052 | 0,961106354 | 0,975989238 |
| b0960 | 2154 | 0,52 | 0,009770866 | 6,017194786 | 0,002368837 | 0,961181726 | 0,975989238 |
| b2988 | 1860 | 0,52 | 0,009977128 | 7,254133006 | 0,002261218 | 0,962073075 | 0,976673051 |
| b0566 | 762 | 0,46 | 0,02367408 | 2,493468198 | 0,002134096 | 0,963153812 | 0,977548774 |
| b4495 | 1129 | 0,34 | -0,047074562 | 0,940474755 | 0,002074471 | 0,963671825 | 0,977821437 |
| b0173 | 1197 | 0,53 | 0,009933596 | 6,873706491 | 0,002036086 | 0,964009258 | 0,977821437 |
| b2262 | 858 | 0,56 | 0,010218384 | 8,216791054 | 0,002018703 | 0,96416312 | 0,977821437 |
| b0547 | 456 | 0,5 | -0,032098355 | 1,377241453 | 0,002003849 | 0,964295125 | 0,977821437 |
| b1921 | 552 | 0,47 | 0,017352201 | 3,253106479 | 0,001913103 | 0,965112427 | 0,978428839 |
| b2630 | 1074 | 0,44 | -0,008171056 | 7,089611727 | 0,001790627 | 0,96624695 | 0,97929812 |
| b0765 | 1059 | 0,54 | -0,009441768 | 5,556490532 | 0,001773689 | 0,966406869 | 0,97929812 |
| b2515 | 1119 | 0,53 | -0,008650709 | 8,46681329 | 0,001726051 | 0,966860802 | 0,979420135 |
| b3575 | 999 | 0,53 | 0,016376361 | 2,829536428 | 0,001715278 | 0,966964323 | 0,979420135 |
| b1758 | 621 | 0,53 | -0,012566627 | 4,288406382 | 0,001670157 | 0,967401479 | 0,979442491 |
| b2506 | 540 | 0,49 | 0,017568467 | 2,91771122 | 0,001667906 | 0,967423451 | 0,979442491 |
| b1400 | 591 | 0,51 | -0,007915169 | 6,636538129 | 0,001624217 | 0,967852702 | 0,979655784 |
| b4140 | 477 | 0,47 | -0,018759114 | 5,590254511 | 0,001587694 | 0,968215999 | 0,979752258 |
| b0267 | 1155 | 0,62 | -0,025149721 | 1,530682806 | 0,001570825 | 0,968385208 | 0,979752258 |
| b0260 | 1404 | 0,55 | 0,012493706 | 4,208658267 | 0,001417443 | 0,969967587 | 0,981131736 |
| b2144 | 720 | 0,48 | -0,010145814 | 4,11164429 | 0,001374778 | 0,970422813 | 0,981370724 |
| b2617 | 342 | 0,49 | 0,008178214 | 6,938332182 | 0,001320485 | 0,971012473 | 0,981745523 |
| b1434 | 537 | 0,49 | 0,011122268 | 3,476872401 | 0,001280747 | 0,971451775 | 0,981968167 |
| b3551 | 2334 | 0,54 | -0,005931664 | 6,616768942 | 0,001256614 | 0,971721914 | 0,982019756 |
| b0225 | 279 | 0,39 | 0,011713074 | 4,099528206 | 0,001217863 | 0,972161162 | 0,982242184 |
| b1098 | 642 | 0,54 | -0,006875126 | 5,394093719 | 0,001109125 | 0,973432541 | 0,983305082 |
| b2847 | 810 | 0,4 | -0,017092793 | 2,551135379 | 0,001066913 | 0,973942824 | 0,98359886 |
| b0711 | 657 | 0,58 | 0,009069318 | 7,583991567 | 0,001033854 | 0,974349565 | 0,983787959 |
| b1103 | 360 | 0,51 | 0,006588535 | 7,607333812 | 0,00096778 | 0,975182484 | 0,983952342 |
| b1455 | 213 | 0,31 | 0,034022341 | 0,572244338 | 0,000965336 | 0,975213832 | 0,983952342 |
| b3663 | 453 | 0,53 | -0,009927627 | 3,735320065 | 0,000946236 | 0,97546018 | 0,983952342 |

| | | | | | | | |
|---|---|---|---|---|---|---|---|
| b1183 | 420 | 0,48 | -0,012025257 | 3,298956901 | 0,000936264 | 0,975589793 | 0,983952342 |
| b3702 | 1404 | 0,54 | 0,006408041 | 7,732399094 | 0,000934711 | 0,975610042 | 0,983952342 |
| b2852 | 1377 | 0,35 | 0,022233345 | 1,380739401 | 0,000904109 | 0,976012492 | 0,984136781 |
| b2269 | 1212 | 0,35 | -0,014369985 | 1,868635129 | 0,00085983 | 0,976607098 | 0,984158615 |
| b1213 | 393 | 0,48 | 0,009600196 | 3,852219756 | 0,000850821 | 0,976729933 | 0,984158615 |
| b1561 | 252 | 0,4 | -0,015496888 | 2,005128858 | 0,00084996 | 0,976741715 | 0,984158615 |
| b1123 | 1047 | 0,51 | 0,008639355 | 8,948729203 | 0,000837522 | 0,976912467 | 0,984158615 |
| b4492 | 8622 | 0,5 | 0,004868414 | 6,990215883 | 0,000678734 | 0,979215444 | 0,986100539 |
| b1111 | 633 | 0,51 | 0,006609073 | 5,79678408 | 0,000674516 | 0,97928012 | 0,986100539 |
| b1553 | 498 | 0,46 | 0,029699237 | 0,773724827 | 0,000650092 | 0,979658617 | 0,98626009 |
| b3861 | 1431 | 0,42 | 0,010926493 | 2,663384863 | 0,000588511 | 0,980645827 | 0,987032247 |
| b2990 | 249 | 0,52 | -0,009042917 | 3,652437825 | 0,000566029 | 0,981019023 | 0,987186184 |
| b2074 | 1248 | 0,55 | 0,007388325 | 4,461261725 | 0,00052224 | 0,981767878 | 0,987512365 |
| b1702 | 2379 | 0,55 | -0,005560737 | 10,79946592 | 0,000521326 | 0,981783824 | 0,987512365 |
| b1321 | 1398 | 0,54 | 0,009152058 | 6,112201052 | 0,000452632 | 0,983026176 | 0,988197054 |
| b1210 | 1257 | 0,54 | 0,004830185 | 6,7816948 | 0,000447505 | 0,983122562 | 0,988197054 |
| b3483 | 384 | 0,33 | 0,017158681 | 1,015579495 | 0,000441297 | 0,983240024 | 0,988197054 |
| b1595 | 894 | 0,53 | 0,006320554 | 4,275010238 | 0,000435708 | 0,983346466 | 0,988197054 |
| b0290 | 1644 | 0,52 | 0,005466051 | 4,692713126 | 0,000382436 | 0,984397587 | 0,988838115 |
| b2496 | 702 | 0,54 | 0,004411102 | 5,601686006 | 0,000381062 | 0,984425629 | 0,988838115 |
| b2003 | 222 | 0,53 | 0,012765217 | 1,456608912 | 0,000296994 | 0,986250332 | 0,990449023 |
| b4497 | 1053 | 0,39 | -0,010777664 | 1,715138206 | 0,000267324 | 0,986955123 | 0,990789209 |
| b1307 | 222 | 0,55 | -0,006186572 | 3,184369866 | 0,000253748 | 0,987290661 | 0,990789209 |
| b2767 | 261 | 0,55 | 0,007978168 | 2,260822588 | 0,000250717 | 0,987366776 | 0,990789209 |
| b2161 | 1251 | 0,5 | 0,006938061 | 2,611198632 | 0,00024617 | 0,987481872 | 0,990789209 |
| b3022 | 297 | 0,4 | -0,003626153 | 5,589187515 | 0,000237882 | 0,987694374 | 0,990789209 |
| b1324 | 507 | 0,51 | 0,003201735 | 10,03638189 | 0,000153637 | 0,99011045 | 0,99299061 |
| b2179 | 1026 | 0,53 | -0,004422725 | 4,325456534 | 0,000146369 | 0,990347181 | 0,993005831 |
| b4341 | 165 | 0,56 | -0,013809667 | 0,583291903 | 0,00013008 | 0,990900101 | 0,993338012 |
| b2248 | 783 | 0,51 | 0,002656064 | 3,038016918 | 4,93032E-05 | 0,994397602 | 0,99662121 |
| b2253 | 1140 | 0,51 | 0,001897414 | 5,515139964 | 2,51197E-05 | 0,996001051 | 0,997886505 |
| b0284 | 2199 | 0,59 | 0,001370301 | 5,713223908 | 2,38264E-05 | 0,99610536 | 0,997886505 |
| b2198 | 210 | 0,57 | 0,002085443 | 3,257496979 | 1,71418E-05 | 0,996696559 | 0,998255637 |
| b2368 | 1164 | 0,42 | -0,001677159 | 2,414175757 | 1,1506E-05 | 0,997293538 | 0,998390614 |
| b4644 | 272 | 0,55 | 0,003600035 | 0,651648342 | 8,86955E-06 | 0,99762376 | 0,998390614 |
| b0709 | 1482 | 0,52 | -0,000708316 | 3,964793215 | 7,30398E-06 | 0,997843649 | 0,998390614 |
| b3619 | 933 | 0,51 | 0,000497783 | 8,467880535 | 7,15833E-06 | 0,997865258 | 0,998390614 |
| b3514 | 3114 | 0,53 | -0,001122877 | 9,324100439 | 6,63286E-06 | 0,997945103 | 0,998390614 |
| b1575 | 189 | 0,36 | -0,000557336 | 0,603672359 | 2,09765E-07 | 0,999634568 | 0,999857651 |
| b2096 | 855 | 0,53 | -3,27087E-05 | 12,32258535 | 1,58834E-08 | 0,999899443 | 0,999899443 |

**Suppplementary material Table S6. Results of RNA seq for analysis of gene expression in WT MG1655 grown with 0.25 mg/L OTC compared to growth in MH-2 media without OTC.**

| Gene | length | gccontent | logFC | logCPM | LR | PValue | FDR |
|---|---|---|---|---|---|---|---|
| b0978 | 1545 | 0,54 | 4,271841609 | 6,975775614 | 178,8082726 | 8,82323E-41 | 3,95457E-37 |
| b2787 | 1341 | 0,53 | -4,633209659 | 8,341469808 | 175,1401858 | 5,57948E-40 | 1,25036E-36 |
| b0775 | 1041 | 0,56 | 4,356085437 | 4,721582929 | 151,064665 | 1,01451E-34 | 1,34981E-31 |
| b2788 | 1341 | 0,54 | -5,827824689 | 8,555626165 | 150,7233197 | 1,20465E-34 | 1,34981E-31 |
| b0979 | 1137 | 0,53 | 3,907374788 | 6,547317359 | 149,0935063 | 2,73591E-34 | 2,45247E-31 |
| b3125 | 885 | 0,53 | -5,50909628 | 10,51871231 | 145,3022215 | 1,84454E-33 | 1,37787E-30 |
| b3124 | 1146 | 0,54 | -5,427225419 | 9,375975037 | 139,3168012 | 3,75505E-32 | 2,40431E-29 |
| b3126 | 771 | 0,53 | -6,408322464 | 10,0676425 | 105,9372585 | 7,61071E-25 | 4,2639E-22 |
| b0776 | 1155 | 0,58 | 4,673064214 | 4,266219353 | 102,7380788 | 3,82521E-24 | 1,90495E-21 |
| b4242 | 2697 | 0,52 | 2,754462868 | 6,487107628 | 96,83515716 | 7,53463E-23 | 3,37702E-20 |
| b4307 | 546 | 0,55 | 2,788241089 | 4,482530021 | 96,15841147 | 1,06049E-22 | 4,32103E-20 |
| b1901 | 990 | 0,52 | -2,702388499 | 6,419783823 | 93,92961588 | 3,26945E-22 | 1,22114E-19 |
| b3117 | 990 | 0,46 | -6,927698446 | 8,257969219 | 93,24819941 | 4,61309E-22 | 1,59045E-19 |
| b1197 | 1698 | 0,52 | -1,857409588 | 7,123440284 | 92,6954687 | 6,09931E-22 | 1,95265E-19 |
| b0774 | 1290 | 0,56 | 3,873008572 | 3,958651447 | 89,34002511 | 3,32464E-21 | 9,93403E-19 |
| b0897 | 627 | 0,46 | -2,581581387 | 9,380717802 | 88,25498892 | 5,75379E-21 | 1,61178E-18 |
| b0778 | 678 | 0,54 | 3,197935321 | 3,213702547 | 86,87712191 | 1,15477E-20 | 3,04453E-18 |
| b3076 | 3093 | 0,55 | -4,094655056 | 8,4057857 | 86,58426024 | 1,33909E-20 | 3,33433E-18 |
| b0980 | 1299 | 0,54 | 2,651486116 | 6,453906154 | 79,61519994 | 4,54912E-19 | 1,07311E-16 |
| b2876 | 771 | 0,5 | -2,799651283 | 6,095076781 | 78,55704623 | 7,77211E-19 | 1,74173E-16 |
| b4306 | 747 | 0,6 | 2,845336783 | 4,838590456 | 73,2283941 | 1,15482E-17 | 2,46472E-15 |
| b0777 | 756 | 0,56 | 4,567644854 | 3,425947215 | 71,53371653 | 2,72561E-17 | 5,5528E-15 |
| b1002 | 1242 | 0,52 | -2,731052529 | 9,998218172 | 70,99983035 | 3,57256E-17 | 6,96182E-15 |
| b2674 | 411 | 0,56 | 4,07208592 | 2,720930756 | 66,96903657 | 2,75805E-16 | 5,15066E-14 |
| b2726 | 351 | 0,53 | -3,163228919 | 5,628958432 | 65,52383025 | 5,74155E-16 | 1,02934E-13 |
| b3129 | 336 | 0,48 | -1,702506786 | 6,200025137 | 64,61310007 | 9,11473E-16 | 1,5161E-13 |
| b2729 | 1122 | 0,55 | -3,034749336 | 7,329588141 | 64,60912437 | 9,13314E-16 | 1,5161E-13 |
| b2809 | 228 | 0,46 | -2,409247244 | 6,763347591 | 64,16714653 | 1,14299E-15 | 1,8296E-13 |
| b0032 | 1149 | 0,53 | 3,919174837 | 5,311350982 | 64,0917427 | 1,18758E-15 | 1,83543E-13 |
| b1647 | 897 | 0,52 | -1,829707025 | 8,026820084 | 63,18054539 | 1,88602E-15 | 2,81771E-13 |
| b3128 | 1572 | 0,53 | -6,057146868 | 11,7737481 | 62,68899799 | 2,42064E-15 | 3,49978E-13 |
| b3668 | 1503 | 0,57 | 2,171571256 | 4,109106644 | 62,06239079 | 3,32746E-15 | 4,66052E-13 |
| b3116 | 1332 | 0,5 | -5,399629649 | 8,028193133 | 59,80999791 | 1,04471E-14 | 1,41891E-12 |
| b2319 | 1014 | 0,55 | 1,51537061 | 6,558808261 | 59,35601824 | 1,31577E-14 | 1,715E-12 |
| b4466 | 4563 | 0,52 | -1,698544802 | 7,671725977 | 59,32122525 | 1,33924E-14 | 1,715E-12 |
| b1165 | 273 | 0,37 | 3,505606784 | 3,638624327 | 58,79723313 | 1,74787E-14 | 2,1761E-12 |
| b2761 | 2667 | 0,45 | -2,721426535 | 5,798336838 | 58,64579797 | 1,88771E-14 | 2,28668E-12 |
| b2728 | 273 | 0,55 | -3,4655386 | 5,206706812 | 57,86088825 | 2,81325E-14 | 3,31816E-12 |
| b3947 | 2502 | 0,55 | -2,064151022 | 5,452028482 | 56,31449615 | 6,17584E-14 | 7,09747E-12 |
| b2209 | 489 | 0,49 | -1,794799063 | 7,551339319 | 55,57736715 | 8,98522E-14 | 1,00679E-11 |
| b1743 | 486 | 0,51 | -2,20954724 | 6,136129208 | 55,05503802 | 1,17202E-13 | 1,28121E-11 |
| b0661 | 1425 | 0,54 | 1,486626088 | 7,067823291 | 54,57419957 | 1,49688E-13 | 1,59738E-11 |
| b3665 | 1767 | 0,55 | -1,609847529 | 5,969487345 | 54,02454345 | 1,98001E-13 | 2,06381E-11 |
| b1629 | 2223 | 0,55 | 2,031734726 | 5,650846806 | 53,6066942 | 2,44923E-13 | 2,49488E-11 |
| b4240 | 1422 | 0,54 | -2,291565711 | 9,010296015 | 53,41678139 | 2,69782E-13 | 2,68703E-11 |
| b0620 | 681 | 0,51 | -2,90319617 | 5,33140105 | 53,34136369 | 2,80341E-13 | 2,7164E-11 |
| b3336 | 477 | 0,43 | -2,110878336 | 10,85461303 | 53,31000626 | 2,84852E-13 | 2,7164E-11 |
| b0621 | 1386 | 0,51 | -6,03266962 | 9,122073977 | 53,14516029 | 3,09788E-13 | 2,89264E-11 |
| b2500 | 639 | 0,54 | 2,365260134 | 6,255293716 | 52,35162166 | 4,64015E-13 | 4,24432E-11 |
| b3074 | 333 | 0,53 | -3,092723373 | 7,390181714 | 52,23901582 | 4,914E-13 | 4,40491E-11 |
| b1207 | 948 | 0,53 | 2,433353164 | 8,557352135 | 52,10196165 | 5,26923E-13 | 4,63072E-11 |
| b4305 | 1122 | 0,52 | -1,863851357 | 5,984488962 | 52,06319561 | 5,37429E-13 | 4,63222E-11 |
| b1876 | 1734 | 0,53 | 1,463727807 | 7,433041801 | 51,94717772 | 5,70139E-13 | 4,82144E-11 |
| b1064 | 648 | 0,49 | -1,821634149 | 7,954482761 | 51,59897786 | 6,8077E-13 | 5,65039E-11 |
| b1441 | 1014 | 0,54 | -1,64077537 | 7,857538007 | 51,54614889 | 6,99337E-13 | 5,69896E-11 |
| b0458 | 471 | 0,47 | 1,931275507 | 6,202703914 | 51,36816273 | 7,65703E-13 | 6,0236E-11 |
| b0920 | 780 | 0,52 | -2,988872718 | 6,268079799 | 51,36726278 | 7,66054E-13 | 6,0236E-11 |
| b1406 | 861 | 0,53 | -1,364739216 | 7,100344188 | 50,95440984 | 9,45364E-13 | 7,30538E-11 |
| b2965 | 2136 | 0,52 | 1,764877726 | 5,668554589 | 50,78702182 | 1,02952E-12 | 7,82088E-11 |
| b0522 | 1068 | 0,56 | 3,062264652 | 7,086467525 | 49,87986656 | 1,63453E-12 | 1,221E-10 |
| b3942 | 2181 | 0,56 | -2,605990447 | 11,06026994 | 49,83312297 | 1,67394E-12 | 1,22993E-10 |
| b2727 | 873 | 0,55 | -3,835014918 | 8,061563227 | 49,78195936 | 1,71817E-12 | 1,24207E-10 |
| b3570 | 825 | 0,48 | 1,847918877 | 7,768565648 | 49,68320244 | 1,80686E-12 | 1,28545E-10 |
| b1020 | 1065 | 0,52 | 3,255715159 | 7,110406683 | 49,56469414 | 1,91937E-12 | 1,34416E-10 |
| b3386 | 678 | 0,51 | 1,414308645 | 7,287036529 | 49,40717318 | 2,07984E-12 | 1,41415E-10 |
| b4375 | 1590 | 0,54 | 1,906093081 | 7,027777328 | 49,40473867 | 2,08242E-12 | 1,41415E-10 |
| b2789 | 1353 | 0,52 | -4,527208683 | 9,479890381 | 48,74490194 | 2,91515E-12 | 1,95011E-10 |
| b4379 | 864 | 0,56 | -3,604934465 | 5,694961555 | 48,5232174 | 3,26402E-12 | 2,15137E-10 |
| b1900 | 1515 | 0,52 | -2,064004463 | 5,97770165 | 48,03843048 | 4,17946E-12 | 2,71484E-10 |
| b4335 | 1152 | 0,54 | -2,070118179 | 6,808574997 | 47,9612053 | 4,34736E-12 | 2,78356E-10 |
| b3746 | 1497 | 0,5 | -2,291960236 | 8,278710589 | 47,62115549 | 5,17075E-12 | 3,26412E-10 |
| b4208 | 1413 | 0,53 | 2,588406859 | 7,688726805 | 47,50104977 | 5,49745E-12 | 3,38638E-10 |
| b4079 | 2148 | 0,54 | -3,297564799 | 7,509895708 | 47,49461284 | 5,51553E-12 | 3,38638E-10 |
| b2746 | 480 | 0,54 | 1,62360859 | 5,865721371 | 47,14707766 | 6,58548E-12 | 3,9773E-10 |
| b1761 | 1344 | 0,53 | 1,780363276 | 6,586044529 | 47,12635774 | 6,65546E-12 | 3,9773E-10 |
| b0894 | 2445 | 0,53 | -4,410199711 | 9,100491403 | 47,07885425 | 6,81874E-12 | 4,02126E-10 |
| b3189 | 1260 | 0,53 | 1,950037263 | 7,216877273 | 46,54689355 | 8,94519E-12 | 5,2068E-10 |
| b1667 | 306 | 0,45 | 1,488893523 | 7,549268064 | 46,48349114 | 9,23936E-12 | 5,27029E-10 |
| b3566 | 993 | 0,44 | -2,072126332 | 5,675170221 | 46,47289636 | 9,28945E-12 | 5,27029E-10 |
| b2312 | 1518 | 0,53 | 2,639903123 | 7,78265447 | 46,4290697 | 9,49957E-12 | 5,32213E-10 |
| b1778 | 414 | 0,49 | 2,136767582 | 7,440168167 | 46,15122683 | 1,09468E-11 | 6,05725E-10 |
| b3567 | 1542 | 0,47 | -1,86381491 | 5,248759197 | 46,02515809 | 1,16744E-11 | 6,38106E-10 |
| b2206 | 2487 | 0,56 | -4,043481674 | 10,56045528 | 45,93323357 | 1,22353E-11 | 6,60704E-10 |
| b2875 | 1626 | 0,52 | -2,906798548 | 6,618311518 | 45,48440406 | 1,53859E-11 | 8,20948E-10 |
| b0030 | 915 | 0,56 | -2,033096381 | 7,534791372 | 45,32195748 | 1,67164E-11 | 8,81448E-10 |
| b3518 | 1398 | 0,48 | -2,963193844 | 5,906111691 | 45,04240081 | 1,92814E-11 | 1,00488E-09 |
| b1675 | 210 | 0,47 | -1,390080966 | 6,026836884 | 45,01897337 | 1,95135E-11 | 1,00528E-09 |
| b4304 | 1314 | 0,52 | -2,362620271 | 5,470973877 | 44,56025173 | 2,46649E-11 | 1,25623E-09 |
| b1658 | 1026 | 0,54 | 2,205150699 | 6,982384726 | 44,51138633 | 2,52882E-11 | 1,26131E-09 |
| b3092 | 1413 | 0,54 | -1,871164694 | 6,878181511 | 44,50834923 | 2,53275E-11 | 1,26131E-09 |

| | | | | | | | |
|---|---|---|---|---|---|---|---|
| b3871 | 1824 | 0,53 | 1,943440446 | 8,612790271 | 44,26283756 | 2,87115E-11 | 1,41412E-09 |
| b3536 | 1572 | 0,49 | -1,452738071 | 7,985708692 | 44,18810454 | 2,98288E-11 | 1,45318E-09 |
| b1056 | 576 | 0,47 | -2,012408211 | 5,391923543 | 44,0659838 | 3,17491E-11 | 1,5301E-09 |
| b1285 | 1986 | 0,48 | -1,453895685 | 8,360885706 | 44,01759871 | 3,25437E-11 | 1,55171E-09 |
| b3159 | 879 | 0,56 | -2,059815859 | 4,605559872 | 43,90984501 | 3,43855E-11 | 1,60923E-09 |
| b4128 | 297 | 0,35 | -2,470082362 | 4,193345582 | 43,90515116 | 3,44681E-11 | 1,60923E-09 |
| b2207 | 264 | 0,55 | -2,697392618 | 6,100326111 | 43,8796102 | 3,49208E-11 | 1,61356E-09 |
| b1189 | 1299 | 0,56 | 3,25920448 | 10,06079499 | 43,68530636 | 3,85656E-11 | 1,76379E-09 |
| b4071 | 567 | 0,54 | -5,045622373 | 6,866197502 | 43,48936678 | 4,26269E-11 | 1,92926E-09 |
| b0759 | 1017 | 0,54 | -1,491291723 | 8,537661288 | 43,47028672 | 4,30446E-11 | 1,92926E-09 |
| b2980 | 765 | 0,55 | 2,593150821 | 5,280045439 | 43,34139549 | 4,59754E-11 | 2,04021E-09 |
| b0074 | 1572 | 0,53 | 1,963433764 | 4,814588556 | 43,29234788 | 4,71424E-11 | 2,07149E-09 |
| b1521 | 1452 | 0,52 | -2,47796116 | 5,478715614 | 43,14957316 | 5,0711E-11 | 2,20667E-09 |
| b1474 | 3048 | 0,54 | -4,525226815 | 9,461838879 | 42,95930361 | 5,58905E-11 | 2,40866E-09 |
| b2568 | 975 | 0,52 | 1,313738357 | 7,082521973 | 42,46189139 | 7,20729E-11 | 3,07648E-09 |
| b1656 | 582 | 0,5 | -1,993925898 | 11,02072294 | 42,39426038 | 7,46087E-11 | 3,15468E-09 |
| b2310 | 783 | 0,5 | 2,670368536 | 6,806004984 | 42,33014472 | 7,70951E-11 | 3,22935E-09 |
| b3919 | 768 | 0,53 | -1,711176686 | 10,44337994 | 42,30832807 | 7,79599E-11 | 3,23533E-09 |
| b2537 | 891 | 0,5 | 3,093504257 | 5,718374991 | 42,19091713 | 8,27836E-11 | 3,404E-09 |
| b1777 | 273 | 0,45 | 2,093524092 | 7,385226909 | 41,92602073 | 9,47928E-11 | 3,85218E-09 |
| b0812 | 504 | 0,49 | -2,037203324 | 12,74911534 | 41,91349489 | 9,54019E-11 | 3,85218E-09 |
| b2730 | 1011 | 0,57 | -2,233655776 | 6,629332477 | 41,78331196 | 1,0197E-10 | 4,08062E-09 |
| b1498 | 1683 | 0,44 | -2,244841543 | 6,836791573 | 41,684772 | 1,07241E-10 | 4,25358E-09 |
| b2330 | 933 | 0,53 | 1,655120645 | 6,817333176 | 41,65162904 | 1,09074E-10 | 4,28835E-09 |
| b3764 | 339 | 0,48 | 1,364423203 | 7,704378361 | 41,47798702 | 1,19206E-10 | 4,64591E-09 |
| b3127 | 1335 | 0,51 | -5,422992407 | 11,00322504 | 41,40304643 | 1,23864E-10 | 4,78586E-09 |
| b2318 | 813 | 0,55 | 1,146939811 | 6,536416747 | 41,16578239 | 1,39849E-10 | 5,35731E-09 |
| b0034 | 396 | 0,46 | -1,868438758 | 4,780083944 | 41,03800123 | 1,49298E-10 | 5,67079E-09 |
| b3011 | 1164 | 0,55 | -2,064241502 | 7,20668396 | 40,62411243 | 1,84516E-10 | 6,94957E-09 |
| b1623 | 1002 | 0,54 | -1,450357584 | 6,351564941 | 40,56106433 | 1,90566E-10 | 7,11765E-09 |
| b0742 | 792 | 0,49 | 1,439658621 | 8,240053119 | 40,3115769 | 2,16522E-10 | 8,02027E-09 |
| b1475 | 885 | 0,55 | -4,4409693 | 7,066268928 | 40,27453685 | 2,20666E-10 | 8,10678E-09 |
| b4072 | 672 | 0,58 | -5,537780473 | 6,268057951 | 40,17557063 | 2,32133E-10 | 8,4587E-09 |
| b2677 | 1203 | 0,49 | 2,384922004 | 4,760179395 | 40,14838506 | 2,35386E-10 | 8,50806E-09 |
| b2499 | 1038 | 0,53 | 3,077550547 | 7,810102852 | 39,79313822 | 2,82335E-10 | 1,01234E-08 |
| b1190 | 1071 | 0,58 | 2,735578338 | 9,291576727 | 39,76076691 | 2,87054E-10 | 1,01544E-08 |
| b1829 | 882 | 0,54 | 1,424082922 | 8,067120787 | 39,75617316 | 2,8773E-10 | 1,01544E-08 |
| b4380 | 1551 | 0,53 | -3,697749836 | 8,38163891 | 39,7160462 | 2,93702E-10 | 1,02842E-08 |
| b0312 | 1473 | 0,58 | 2,065947608 | 6,489104126 | 39,67763797 | 2,99536E-10 | 1,04071E-08 |
| b0104 | 1044 | 0,51 | 1,772930076 | 7,421266832 | 39,62948023 | 3,07014E-10 | 1,05849E-08 |
| b2869 | 1779 | 0,46 | -2,941868199 | 9,165419121 | 39,59800986 | 3,12001E-10 | 1,06632E-08 |
| b3745 | 1452 | 0,53 | -1,634736217 | 6,826015355 | 39,58527717 | 3,14042E-10 | 1,06632E-08 |
| b3831 | 762 | 0,54 | -1,803038982 | 9,626487273 | 39,51840005 | 3,24983E-10 | 1,09517E-08 |
| b2552 | 1191 | 0,52 | -2,94150101 | 7,839493807 | 39,20275296 | 3,82001E-10 | 1,27771E-08 |
| b0143 | 1398 | 0,56 | 1,351884427 | 7,337738071 | 39,14538306 | 3,93392E-10 | 1,30606E-08 |
| b1739 | 339 | 0,5 | -1,840197547 | 10,66813495 | 39,10068128 | 4,02504E-10 | 1,32649E-08 |
| b2557 | 3888 | 0,57 | 2,315968496 | 9,926261831 | 39,01577842 | 4,20394E-10 | 1,37533E-08 |
| b4064 | 1350 | 0,56 | 1,930445304 | 6,23755281 | 38,95765664 | 4,33098E-10 | 1,40663E-08 |
| b1286 | 1935 | 0,53 | 1,642751198 | 7,060659441 | 38,89503159 | 4,47217E-10 | 1,44203E-08 |
| b0073 | 1092 | 0,55 | 2,244944157 | 4,9110525 | 38,81312654 | 4,66381E-10 | 1,49309E-08 |
| b1182 | 912 | 0,41 | -2,755769808 | 4,65285162 | 38,75789263 | 4,79766E-10 | 1,52504E-08 |
| b2981 | 1101 | 0,51 | 1,980980563 | 3,079139391 | 38,70248033 | 4,93581E-10 | 1,55791E-08 |
| b0888 | 966 | 0,54 | 2,325992194 | 7,401431913 | 38,45071125 | 5,61541E-10 | 1,76002E-08 |
| b2219 | 1827 | 0,49 | -1,772394384 | 6,245546429 | 38,34849904 | 5,91736E-10 | 1,84178E-08 |
| b2678 | 1065 | 0,58 | 2,225036926 | 4,736133025 | 38,03995734 | 6,93105E-10 | 2,14241E-08 |
| b2905 | 1095 | 0,55 | 1,823283386 | 10,28186658 | 37,70020144 | 8,2496E-10 | 2,53252E-08 |
| b1088 | 522 | 0,5 | 1,76891473 | 9,08855988 | 37,43738833 | 9,43951E-10 | 2,87809E-08 |
| b0876 | 1659 | 0,53 | -1,510429367 | 6,979599263 | 37,27388768 | 1,0265E-09 | 3,10863E-08 |
| b2400 | 1416 | 0,53 | 1,654104118 | 8,425606969 | 37,13496015 | 1,1023E-09 | 3,31577E-08 |
| b1687 | 3057 | 0,54 | 1,44646587 | 8,726002234 | 36,85327088 | 1,27362E-09 | 3,80558E-08 |
| b3714 | 1338 | 0,54 | 1,430887178 | 6,396265899 | 36,78880905 | 1,31644E-09 | 3,90747E-08 |
| b3209 | 654 | 0,53 | -1,435369034 | 7,831736694 | 36,70206926 | 1,37633E-09 | 4,05837E-08 |
| b3588 | 1539 | 0,55 | -1,90817334 | 10,06233491 | 36,30474673 | 1,68752E-09 | 4,94241E-08 |
| b3267 | 222 | 0,54 | 4,313923145 | 2,710448455 | 36,29246165 | 1,69819E-09 | 4,94241E-08 |
| b1440 | 1146 | 0,53 | -1,791582934 | 9,650793043 | 36,11130009 | 1,86363E-09 | 5,3889E-08 |
| b4120 | 1422 | 0,46 | -1,631473357 | 5,281742065 | 36,01738123 | 1,95565E-09 | 5,61874E-08 |
| b0480 | 1653 | 0,51 | -1,400501898 | 8,735978679 | 35,9517649 | 2,02263E-09 | 5,77416E-08 |
| b3685 | 1662 | 0,53 | -1,274082691 | 7,052018681 | 35,87827249 | 2,10038E-09 | 5,95817E-08 |
| b2292 | 1833 | 0,52 | -2,325470829 | 7,400577491 | 35,72317473 | 2,27442E-09 | 6,38604E-08 |
| b1095 | 1242 | 0,53 | 1,712912081 | 9,102843096 | 35,71864954 | 2,27971E-09 | 6,38604E-08 |
| b0121 | 867 | 0,51 | 1,252123048 | 6,612593631 | 35,60740184 | 2,41369E-09 | 6,69499E-08 |
| b3565 | 1323 | 0,51 | -1,297196682 | 6,32388892 | 35,60241413 | 2,41987E-09 | 6,69499E-08 |
| b4656 | 261 | 0,51 | 1,513512166 | 4,928091858 | 35,56501991 | 2,46678E-09 | 6,78288E-08 |
| b0839 | 1203 | 0,52 | -1,412752973 | 9,25995311 | 35,44804024 | 2,61946E-09 | 7,15879E-08 |
| b0175 | 858 | 0,51 | 1,029287946 | 7,19909671 | 35,29335277 | 2,83598E-09 | 7,70356E-08 |
| b1164 | 237 | 0,42 | 4,09630562 | 3,454558537 | 35,15679121 | 3,04199E-09 | 8,21337E-08 |
| b0896 | 864 | 0,55 | -3,388963797 | 6,493276067 | 35,00591684 | 3,28705E-09 | 8,82189E-08 |
| b2320 | 1137 | 0,53 | 1,139576136 | 7,180240352 | 34,59210185 | 4,06553E-09 | 1,07927E-07 |
| b3352 | 1914 | 0,55 | 1,387774011 | 6,298319834 | 34,57929249 | 4,09237E-09 | 1,07927E-07 |
| b1628 | 579 | 0,55 | 1,997563845 | 3,866485364 | 34,57869329 | 4,09363E-09 | 1,07927E-07 |
| b0564 | 750 | 0,32 | -2,74922685 | 6,246693599 | 34,39981453 | 4,48766E-09 | 1,16955E-07 |
| b2287 | 663 | 0,52 | 1,586180773 | 9,035000692 | 34,39956976 | 4,48822E-09 | 1,16955E-07 |
| b0820 | 1593 | 0,53 | 1,183505084 | 6,571681969 | 34,27771613 | 4,77822E-09 | 1,23359E-07 |
| b3783 | 1260 | 0,5 | 1,712797134 | 8,590115287 | 34,2733151 | 4,78904E-09 | 1,23359E-07 |
| b0895 | 618 | 0,52 | -4,231764352 | 6,190401811 | 34,23391979 | 4,88697E-09 | 1,24519E-07 |
| b2838 | 1263 | 0,53 | 1,512918005 | 4,63750111 | 34,23286042 | 4,88963E-09 | 1,24519E-07 |
| b3926 | 1509 | 0,54 | -1,255387422 | 7,622237545 | 34,17489625 | 5,03746E-09 | 1,27559E-07 |
| b3528 | 1287 | 0,52 | 2,981706374 | 9,917292249 | 34,10983271 | 5,20874E-09 | 1,31155E-07 |
| b0733 | 1569 | 0,53 | -2,271628004 | 11,25813257 | 34,09164597 | 5,25765E-09 | 1,31647E-07 |
| b1786 | 1491 | 0,46 | 1,492518598 | 5,140640325 | 34,02038912 | 5,45376E-09 | 1,35799E-07 |
| b4239 | 1656 | 0,53 | -2,125903629 | 7,735817406 | 33,99744687 | 5,51844E-09 | 1,3665E-07 |
| b0644 | 978 | 0,47 | -1,198438012 | 5,83774128 | 33,95237112 | 5,64778E-09 | 1,39084E-07 |
| b0313 | 588 | 0,57 | 2,47207329 | 5,286474074 | 33,91736883 | 5,75031E-09 | 1,40835E-07 |
| b3737 | 240 | 0,54 | 1,914332026 | 9,049505636 | 33,83477297 | 5,99968E-09 | 1,46144E-07 |

| | | | | | | | |
|---|---|---|---|---|---|---|---|
| b3251 | 1044 | 0,54 | 1,471236659 | 7,949945182 | 33,63580041 | 6,64582E-09 | 1,60202E-07 |
| b1178 | 402 | 0,43 | -1,69734334 | 5,17587848 | 33,62517078 | 6,68224E-09 | 1,60202E-07 |
| b2313 | 489 | 0,49 | 2,495676781 | 6,450262277 | 33,62464893 | 6,68403E-09 | 1,60202E-07 |
| b1004 | 597 | 0,54 | -1,964433896 | 11,24557193 | 33,56989778 | 6,87485E-09 | 1,63899E-07 |
| b0325 | 1050 | 0,53 | -1,388559037 | 8,568704262 | 33,48160199 | 7,19413E-09 | 1,7048E-07 |
| b3019 | 2259 | 0,55 | 1,111888956 | 7,553683376 | 33,47274876 | 7,22695E-09 | 1,7048E-07 |
| b0523 | 510 | 0,59 | 2,829148218 | 5,729706917 | 33,39666957 | 7,51526E-09 | 1,76353E-07 |
| b4119 | 1356 | 0,52 | -1,687536235 | 6,262013874 | 33,36450183 | 7,64059E-09 | 1,7836E-07 |
| b0967 | 1191 | 0,52 | 1,302794626 | 5,245288011 | 33,33261631 | 7,7669E-09 | 1,80369E-07 |
| b3003 | 885 | 0,54 | -1,819436222 | 7,91530377 | 33,29338298 | 7,92518E-09 | 1,83096E-07 |
| b1283 | 219 | 0,52 | -2,217624774 | 8,105232783 | 33,19985389 | 8,31568E-09 | 1,91133E-07 |
| b3087 | 987 | 0,53 | -1,699541565 | 8,512698865 | 33,11210796 | 8,69953E-09 | 1,98935E-07 |
| b3612 | 1545 | 0,53 | -1,53985057 | 8,79586655 | 33,05357066 | 8,96542E-09 | 2,03975E-07 |
| b1757 | 1308 | 0,54 | -1,780388481 | 6,883881716 | 33,04297257 | 9,01443E-09 | 2,04054E-07 |
| b1630 | 1059 | 0,53 | 2,031942993 | 4,546522058 | 33,0256929 | 9,0949E-09 | 2,04841E-07 |
| b1378 | 3525 | 0,53 | -1,679563271 | 9,770421686 | 32,98719051 | 9,27681E-09 | 2,07893E-07 |
| b1422 | 924 | 0,51 | 3,699575985 | 6,036386974 | 32,91847225 | 9,61057E-09 | 2,14301E-07 |
| b2205 | 696 | 0,59 | -3,3331325 | 7,421449079 | 32,85998876 | 9,90409E-09 | 2,19753E-07 |
| b0304 | 1326 | 0,46 | -1,312026752 | 6,040018317 | 32,76357606 | 1,04077E-08 | 2,29789E-07 |
| b1132 | 642 | 0,57 | 1,377411591 | 5,76552635 | 32,72116279 | 1,06373E-08 | 2,33707E-07 |
| b3945 | 1104 | 0,55 | -3,332911263 | 8,067647234 | 32,61320513 | 1,12448E-08 | 2,4585E-07 |
| b2513 | 621 | 0,5 | 1,140281908 | 7,24744341 | 32,5937839 | 1,13577E-08 | 2,47114E-07 |
| b0872 | 969 | 0,55 | -3,388394137 | 5,825685741 | 32,49171901 | 1,19702E-08 | 2,58333E-07 |
| b2286 | 1791 | 0,56 | 1,560553121 | 10,21265101 | 32,48871181 | 1,19887E-08 | 2,58333E-07 |
| b0526 | 1386 | 0,54 | 1,25540086 | 6,726306594 | 32,35201662 | 1,28624E-08 | 2,75833E-07 |
| b3385 | 759 | 0,53 | 1,294803277 | 7,1631241 | 32,09958188 | 1,46469E-08 | 3,12393E-07 |
| b3030 | 1893 | 0,54 | 1,157849865 | 7,188637413 | 32,09168123 | 1,47066E-08 | 3,12393E-07 |
| b3256 | 1350 | 0,53 | 1,82662802 | 8,691988665 | 31,90527593 | 1,61877E-08 | 3,42232E-07 |
| b1476 | 654 | 0,5 | -3,54035058 | 6,261069992 | 31,8893503 | 1,6321E-08 | 3,4343E-07 |
| b4050 | 243 | 0,5 | 2,483935986 | 2,437446203 | 31,87626447 | 1,64313E-08 | 3,44135E-07 |
| b3255 | 471 | 0,52 | 1,646809192 | 7,621616308 | 31,8495489 | 1,66588E-08 | 3,47279E-07 |
| b1512 | 954 | 0,51 | 1,43858207 | 6,857883546 | 31,79093727 | 1,71692E-08 | 3,56261E-07 |
| b1039 | 390 | 0,43 | 3,303586932 | 2,186603408 | 31,77112318 | 1,73453E-08 | 3,57064E-07 |
| b1631 | 621 | 0,53 | 1,753536388 | 4,444759714 | 31,76866329 | 1,73672E-08 | 3,57064E-07 |
| b1138 | 705 | 0,31 | -1,520507441 | 4,130473287 | 31,75586749 | 1,7482E-08 | 3,57783E-07 |
| b4049 | 993 | 0,54 | 1,45236987 | 4,941661399 | 31,60410757 | 1,89029E-08 | 3,8437E-07 |
| b2955 | 1137 | 0,53 | 1,586313179 | 5,346420016 | 31,5990087 | 1,89526E-08 | 3,8437E-07 |
| b2143 | 885 | 0,55 | -1,343605821 | 6,960661553 | 31,56393636 | 1,9298E-08 | 3,89612E-07 |
| b2703 | 960 | 0,55 | -2,287216755 | 5,235923908 | 31,46435075 | 2,03136E-08 | 4,08275E-07 |
| b3060 | 933 | 0,5 | -2,643847972 | 5,782731041 | 31,44003846 | 2,05695E-08 | 4,10188E-07 |
| b1167 | 249 | 0,36 | 3,660605481 | 2,42468535 | 31,43793798 | 2,05918E-08 | 4,10188E-07 |
| b0758 | 1047 | 0,56 | -1,365997691 | 8,100562015 | 31,41111637 | 2,08782E-08 | 4,14054E-07 |
| b3001 | 1041 | 0,52 | -1,479991499 | 8,911142069 | 31,30286404 | 2,20754E-08 | 4,35867E-07 |
| b0211 | 1359 | 0,52 | 1,347600121 | 8,184287229 | 31,25270389 | 2,26532E-08 | 4,45314E-07 |
| b4133 | 1539 | 0,42 | -2,083107024 | 4,341107731 | 31,13549368 | 2,40631E-08 | 4,70871E-07 |
| b1380 | 990 | 0,5 | -1,685644201 | 8,091781446 | 31,12663747 | 2,41731E-08 | 4,70871E-07 |
| b0855 | 1134 | 0,55 | 1,952866097 | 4,498490348 | 31,11899793 | 2,42685E-08 | 4,70871E-07 |
| b3738 | 816 | 0,5 | 2,05466648 | 9,063115932 | 31,07837126 | 2,47817E-08 | 4,78758E-07 |
| b2535 | 1281 | 0,52 | -1,885811578 | 10,14697179 | 30,9663919 | 2,62535E-08 | 5,05014E-07 |
| b3846 | 2190 | 0,55 | 3,923391419 | 7,29112466 | 30,74833349 | 2,93756E-08 | 5,62656E-07 |
| b0906 | 693 | 0,48 | -1,091594113 | 5,949910076 | 30,71167848 | 2,99358E-08 | 5,70946E-07 |
| b1003 | 228 | 0,47 | -1,826022984 | 9,99263474 | 30,67219146 | 3,05512E-08 | 5,79998E-07 |
| b0652 | 726 | 0,52 | 1,715632986 | 6,160307778 | 30,6647122 | 3,06692E-08 | 5,79998E-07 |
| b1747 | 1035 | 0,56 | 3,200562823 | 7,311079116 | 30,57711427 | 3,20856E-08 | 6,04234E-07 |
| b2366 | 1329 | 0,51 | -1,368594888 | 5,535746031 | 30,48380925 | 3,36665E-08 | 6,30626E-07 |
| b4122 | 1647 | 0,55 | -2,541020057 | 6,098913732 | 30,47793914 | 3,37685E-08 | 6,30626E-07 |
| b4252 | 453 | 0,5 | -1,427844304 | 5,997351729 | 30,44403015 | 3,43639E-08 | 6,39083E-07 |
| b3641 | 597 | 0,53 | 1,790696514 | 5,570799603 | 30,31598462 | 3,67088E-08 | 6,79872E-07 |
| b1038 | 417 | 0,42 | 2,747377294 | 2,268388546 | 30,28336894 | 3,73313E-08 | 6,87389E-07 |
| b3713 | 567 | 0,53 | -1,139907697 | 7,135364229 | 30,27869215 | 3,74214E-08 | 6,87389E-07 |
| b3423 | 758 | 0,55 | 1,550627793 | 5,309962745 | 30,25998007 | 3,77842E-08 | 6,9122E-07 |
| b2673 | 246 | 0,53 | 3,487085377 | 2,227090059 | 30,19284716 | 3,91149E-08 | 7,12655E-07 |
| b0903 | 2283 | 0,51 | -1,693175761 | 12,35673177 | 30,1580065 | 3,9824E-08 | 7,22636E-07 |
| b4073 | 957 | 0,58 | -5,217047245 | 6,315042471 | 30,07395875 | 4,15879E-08 | 7,50947E-07 |
| b3185 | 258 | 0,5 | 1,951228612 | 8,208785962 | 30,06784049 | 4,17193E-08 | 7,50947E-07 |
| b2996 | 987 | 0,55 | -2,840093515 | 8,150080437 | 30,05365207 | 4,20256E-08 | 7,53436E-07 |
| b4188 | 276 | 0,51 | -2,295106466 | 9,153663587 | 30,00068871 | 4,31893E-08 | 7,71213E-07 |
| b0307 | 1428 | 0,5 | -2,772832042 | 7,173282336 | 29,96972472 | 4,38845E-08 | 7,80517E-07 |
| b0865 | 516 | 0,54 | -1,45934957 | 8,966938693 | 29,89050495 | 4,57146E-08 | 8,09853E-07 |
| b1748 | 1221 | 0,55 | 3,725304487 | 7,621265365 | 29,87247169 | 4,61418E-08 | 8,14203E-07 |
| b2351 | 921 | 0,4 | 2,322608982 | 5,860537904 | 29,822824 | 4,73386E-08 | 8,32046E-07 |
| b0432 | 948 | 0,51 | 3,388340028 | 9,540231598 | 29,8067798 | 4,7732E-08 | 8,35683E-07 |
| b0346 | 834 | 0,56 | 2,576708116 | 5,960138549 | 29,67795924 | 5,10115E-08 | 8,89625E-07 |
| b3736 | 471 | 0,53 | 1,946947564 | 10,12804936 | 29,6666224 | 5,13107E-08 | 8,91375E-07 |
| b4070 | 1437 | 0,52 | -4,70680465 | 9,25510194 | 29,5949368 | 5,32438E-08 | 9,21386E-07 |
| b0722 | 348 | 0,51 | 3,148252733 | 8,300492119 | 29,56878123 | 5,39672E-08 | 9,29293E-07 |
| b1783 | 1935 | 0,5 | -1,885839457 | 11,86996311 | 29,56346236 | 5,41155E-08 | 9,29293E-07 |
| b3396 | 2553 | 0,56 | 1,260522423 | 5,679368771 | 29,54464476 | 5,46434E-08 | 9,34778E-07 |
| b2766 | 1272 | 0,55 | -1,631136817 | 5,50789789 | 29,53070547 | 5,50378E-08 | 9,37945E-07 |
| b0185 | 960 | 0,52 | 1,342539014 | 8,078742507 | 29,42037529 | 5,82619E-08 | 9,89128E-07 |
| b2587 | 1299 | 0,49 | 2,234028887 | 9,213232976 | 29,37786335 | 5,9554E-08 | 1,00481E-06 |
| b4592 | 93 | 0,46 | 2,363980105 | 2,248735059 | 29,37525767 | 5,96342E-08 | 1,00481E-06 |
| b2845 | 1230 | 0,44 | 1,649125821 | 4,907478263 | 29,3176069 | 6,14349E-08 | 1,03128E-06 |
| b0281 | 1401 | 0,44 | 1,077319194 | 6,266196333 | 29,29826245 | 6,20513E-08 | 1,03774E-06 |
| b4470 | 1311 | 0,57 | 1,811844774 | 6,640733075 | 29,28245872 | 6,25595E-08 | 1,04235E-06 |
| b0450 | 339 | 0,51 | 3,228429566 | 1,345462008 | 29,25309348 | 6,35147E-08 | 1,05434E-06 |
| b1587 | 2427 | 0,51 | -2,535875386 | 6,352536904 | 29,24467956 | 6,37911E-08 | 1,05503E-06 |
| b1113 | 963 | 0,53 | 1,650765203 | 4,892593131 | 29,03135223 | 7,12163E-08 | 1,1735E-06 |
| b2204 | 864 | 0,56 | -3,183535328 | 6,922008258 | 28,99490022 | 7,25691E-08 | 1,19141E-06 |
| b2114 | 2034 | 0,53 | 1,200780965 | 8,099601449 | 28,69128962 | 8,48847E-08 | 1,3877E-06 |
| b3253 | 975 | 0,55 | -1,331776566 | 8,107739426 | 28,6853666 | 8,51447E-08 | 1,3877E-06 |
| b3828 | 954 | 0,56 | 2,72951541 | 4,143700305 | 28,6163972 | 8,82319E-08 | 1,43281E-06 |
| b1619 | 768 | 0,47 | -1,406707346 | 9,189995903 | 28,5840755 | 8,9717E-08 | 1,45167E-06 |
| b0063 | 1701 | 0,58 | -1,496441385 | 5,351817639 | 28,55257503 | 9,11885E-08 | 1,46821E-06 |

| | | | | | | | |
|---|---|---|---|---|---|---|---|
| b1462 | 570 | 0,51 | -1,236603773 | 5,340129956 | 28,54820405 | 9,13946E-08 | 1,46821E-06 |
| b3024 | 393 | 0,52 | -1,540627675 | 10,117277 | 28,51391864 | 9,30273E-08 | 1,4891E-06 |
| b0314 | 2034 | 0,55 | 1,523128973 | 5,92121651 | 28,49664817 | 9,38608E-08 | 1,4971E-06 |
| b1276 | 2676 | 0,53 | -1,615119706 | 11,52955077 | 28,45546482 | 9,58788E-08 | 1,52386E-06 |
| b1674 | 627 | 0,49 | -2,491680842 | 4,431943145 | 28,32346924 | 1,02644E-07 | 1,62562E-06 |
| b1131 | 1371 | 0,52 | 2,040845016 | 8,104292294 | 28,3081191 | 1,03461E-07 | 1,63279E-06 |
| b1127 | 1227 | 0,5 | -1,625542301 | 9,213342358 | 28,17901633 | 1,10597E-07 | 1,73929E-06 |
| b0329 | 276 | 0,39 | -1,649064697 | 8,889065544 | 28,15610227 | 1,11914E-07 | 1,75385E-06 |
| b2675 | 2145 | 0,52 | 1,545604929 | 4,926422996 | 28,1001141 | 1,15199E-07 | 1,79903E-06 |
| b4460 | 987 | 0,51 | -1,256320573 | 5,424275576 | 28,08690245 | 1,15988E-07 | 1,80507E-06 |
| b2288 | 444 | 0,53 | 1,349624115 | 7,979300632 | 28,03590832 | 1,19085E-07 | 1,84685E-06 |
| b1225 | 1539 | 0,54 | -4,819206808 | 10,86895997 | 27,90010254 | 1,27743E-07 | 1,97429E-06 |
| b0721 | 390 | 0,49 | 3,480400118 | 7,908558539 | 27,8757076 | 1,29364E-07 | 1,99247E-06 |
| b1234 | 906 | 0,5 | -1,064463436 | 7,332143334 | 27,82520865 | 1,32785E-07 | 2,03815E-06 |
| b0992 | 1074 | 0,49 | -3,570237101 | 5,550500672 | 27,75803929 | 1,37476E-07 | 2,10296E-06 |
| b4154 | 1809 | 0,56 | -2,076656427 | 11,25347464 | 27,74280364 | 1,38563E-07 | 2,11237E-06 |
| b1673 | 2103 | 0,48 | -2,635568672 | 5,493628364 | 27,7354121 | 1,39093E-07 | 2,11327E-06 |
| b2802 | 1776 | 0,55 | -1,916680752 | 6,131753918 | 27,72172986 | 1,4008E-07 | 2,12108E-06 |
| b2890 | 1518 | 0,53 | 1,501309801 | 8,744562139 | 27,64414661 | 1,45813E-07 | 2,20045E-06 |
| b3744 | 993 | 0,56 | 1,449858244 | 6,329769829 | 27,59347292 | 1,49683E-07 | 2,25127E-06 |
| b1299 | 558 | 0,51 | 2,307292864 | 5,220281475 | 27,35145527 | 1,69637E-07 | 2,54285E-06 |
| b1451 | 2103 | 0,51 | 1,92759731 | 4,682077249 | 27,23298138 | 1,80356E-07 | 2,69451E-06 |
| b0423 | 1449 | 0,52 | 1,221652679 | 5,690263587 | 27,20396295 | 1,83083E-07 | 2,72617E-06 |
| b1442 | 942 | 0,53 | -1,159455665 | 6,474451923 | 27,12692027 | 1,90526E-07 | 2,82761E-06 |
| b2813 | 1098 | 0,53 | 1,091852442 | 5,881738224 | 27,09641136 | 1,93557E-07 | 2,86311E-06 |
| b3931 | 1332 | 0,53 | 1,485250334 | 8,573845337 | 27,07942483 | 1,95265E-07 | 2,87888E-06 |
| b0948 | 2109 | 0,54 | 1,135523732 | 6,774398869 | 27,01899541 | 2,01466E-07 | 2,95191E-06 |
| b2431 | 900 | 0,56 | -1,603310486 | 8,71766915 | 27,01832248 | 2,01536E-07 | 2,95191E-06 |
| b4566 | 258 | 0,5 | 1,975944282 | 3,314672879 | 26,95177343 | 2,08596E-07 | 3,04536E-06 |
| b3335 | 678 | 0,5 | -1,486236329 | 4,8281543 | 26,88242962 | 2,16216E-07 | 3,14636E-06 |
| b3640 | 456 | 0,55 | 1,238340349 | 5,962459481 | 26,86961944 | 2,17654E-07 | 3,15703E-06 |
| b0931 | 1203 | 0,51 | 1,358718541 | 6,370927734 | 26,80676653 | 2,24849E-07 | 3,25088E-06 |
| b0194 | 1719 | 0,55 | 1,337554486 | 9,035058128 | 26,77410321 | 2,28681E-07 | 3,28521E-06 |
| b3118 | 939 | 0,42 | -3,790521188 | 8,719753546 | 26,77403449 | 2,2869E-07 | 3,28521E-06 |
| b1634 | 1503 | 0,52 | 1,292334262 | 6,311233557 | 26,71289979 | 2,3604E-07 | 3,37998E-06 |
| b2365 | 1338 | 0,49 | -1,972394365 | 4,598413147 | 26,67806249 | 2,40335E-07 | 3,43051E-06 |
| b4342 | 1503 | 0,44 | -1,470908518 | 7,372298752 | 26,61425002 | 2,48405E-07 | 3,53445E-06 |
| b2234 | 2286 | 0,54 | 1,706603537 | 8,2611288 | 26,5879118 | 2,51814E-07 | 3,57162E-06 |
| b0740 | 1293 | 0,53 | 1,189161061 | 9,195662405 | 26,47336394 | 2,67197E-07 | 3,77785E-06 |
| b3012 | 828 | 0,51 | -1,704714534 | 9,199183325 | 26,43354808 | 2,72762E-07 | 3,8444E-06 |
| b0120 | 795 | 0,46 | 1,240220999 | 6,408882683 | 26,35443922 | 2,84165E-07 | 3,99256E-06 |
| b0653 | 675 | 0,5 | 1,566168198 | 5,230645665 | 26,3025141 | 2,91908E-07 | 4,08854E-06 |
| b2569 | 1800 | 0,54 | 1,204485216 | 8,113333582 | 26,29365982 | 2,9325E-07 | 4,09453E-06 |
| b0970 | 660 | 0,51 | 1,47081143 | 8,419576871 | 26,279841 | 2,95356E-07 | 4,11113E-06 |
| b2511 | 1473 | 0,52 | 0,983568399 | 7,851864151 | 26,18349107 | 3,10465E-07 | 4,30807E-06 |
| b4116 | 762 | 0,41 | -3,723947948 | 4,546750209 | 26,17716348 | 3,11484E-07 | 4,30887E-06 |
| b3735 | 534 | 0,52 | 2,047610578 | 9,653149457 | 26,03664838 | 3,34998E-07 | 4,61987E-06 |
| b1636 | 864 | 0,53 | 1,120262171 | 5,770090698 | 25,91702747 | 3,56411E-07 | 4,89558E-06 |
| b0766 | 819 | 0,52 | 1,379979115 | 4,90458924 | 25,91289885 | 3,57174E-07 | 4,89558E-06 |
| b4334 | 768 | 0,57 | -2,012720321 | 4,984852343 | 25,89155497 | 3,61146E-07 | 4,93492E-06 |
| b0772 | 1284 | 0,53 | 1,428343526 | 7,098489281 | 25,79586903 | 3,795E-07 | 5,16996E-06 |
| b2153 | 669 | 0,51 | 1,390252963 | 6,594870686 | 25,6986255 | 3,99111E-07 | 5,42066E-06 |
| b1902 | 504 | 0,48 | -1,281047341 | 6,958826939 | 25,60691682 | 4,18536E-07 | 5,66731E-06 |
| b0809 | 723 | 0,54 | 1,650124979 | 6,436261304 | 25,46227669 | 4,51118E-07 | 6,09009E-06 |
| b1830 | 2049 | 0,5 | 1,265611497 | 8,574491489 | 25,44283317 | 4,55687E-07 | 6,13331E-06 |
| b1784 | 1284 | 0,49 | -1,727844312 | 9,529170872 | 25,42499255 | 4,59921E-07 | 6,17175E-06 |
| b2964 | 1257 | 0,5 | -1,40805035 | 7,378930151 | 25,3382044 | 4,81085E-07 | 6,43649E-06 |
| b2628 | 1617 | 0,49 | -1,108441595 | 6,963830263 | 25,29267437 | 4,92576E-07 | 6,57061E-06 |
| b0627 | 204 | 0,5 | 1,333967745 | 6,062006909 | 25,28444028 | 4,94683E-07 | 6,57914E-06 |
| b0126 | 663 | 0,51 | 1,791866841 | 7,304582685 | 25,27812364 | 4,96306E-07 | 6,58119E-06 |
| b3941 | 891 | 0,52 | 2,944354752 | 4,088123693 | 25,22086068 | 5,11262E-07 | 6,7515E-06 |
| b0734 | 1140 | 0,54 | -2,098175889 | 10,49751586 | 25,21746958 | 5,12162E-07 | 6,7515E-06 |
| b2997 | 1119 | 0,54 | -2,871285464 | 9,002792567 | 25,17780179 | 5,22805E-07 | 6,84716E-06 |
| b0240 | 1178 | 0,5 | 1,731761364 | 7,432264996 | 25,17389841 | 5,23864E-07 | 6,84716E-06 |
| b3542 | 903 | 0,59 | 1,719941373 | 7,876069647 | 25,17339309 | 5,24002E-07 | 6,84716E-06 |
| b0197 | 816 | 0,49 | 0,944534737 | 7,325252635 | 25,11220818 | 5,40893E-07 | 7,04734E-06 |
| b3591 | 1392 | 0,56 | -1,269866927 | 7,125533911 | 25,10647573 | 5,42504E-07 | 7,04783E-06 |
| b1055 | 1053 | 0,5 | 1,964429949 | 4,973868363 | 25,0567303 | 5,56681E-07 | 7,21111E-06 |
| b1535 | 891 | 0,45 | 1,702126749 | 4,073886944 | 25,03686988 | 5,62445E-07 | 7,26477E-06 |
| b2521 | 846 | 0,53 | -1,313840252 | 9,361091204 | 25,00886659 | 5,70673E-07 | 7,34987E-06 |
| b3845 | 1164 | 0,58 | 2,645951182 | 6,710397151 | 24,98390723 | 5,78108E-07 | 7,4243E-06 |
| b3962 | 1401 | 0,52 | 1,971627205 | 9,082424586 | 24,96604522 | 5,83489E-07 | 7,45527E-06 |
| b0999 | 306 | 0,54 | -1,575578395 | 7,934791916 | 24,96486371 | 5,83847E-07 | 7,45527E-06 |
| b1015 | 1509 | 0,54 | 3,001163365 | 8,826965965 | 24,87706855 | 6,1105E-07 | 7,78047E-06 |
| b1046 | 1422 | 0,51 | -1,099966458 | 6,776727552 | 24,84073859 | 6,22675E-07 | 7,90603E-06 |
| b4149 | 534 | 0,53 | -1,529799073 | 7,882831546 | 24,76529524 | 6,47529E-07 | 8,19838E-06 |
| b1461 | 234 | 0,43 | -1,562865837 | 4,304152267 | 24,72185478 | 6,62289E-07 | 8,34261E-06 |
| b4349 | 1590 | 0,55 | 1,226923595 | 6,206489917 | 24,72082323 | 6,62644E-07 | 8,34261E-06 |
| b2536 | 1140 | 0,56 | -1,399155575 | 6,382301391 | 24,71106043 | 6,66009E-07 | 8,36149E-06 |
| b2957 | 1047 | 0,53 | -4,212608485 | 10,93586382 | 24,66606827 | 6,8174E-07 | 8,53508E-06 |
| b3407 | 2322 | 0,57 | 1,389916473 | 7,287058268 | 24,62004336 | 6,98217E-07 | 8,71702E-06 |
| b3107 | 165 | 0,52 | 1,371022406 | 4,235212662 | 24,60670985 | 7,03065E-07 | 8,75316E-06 |
| b3365 | 2544 | 0,54 | -3,322632848 | 8,732288366 | 24,57324755 | 7,1538E-07 | 8,88181E-06 |
| b1297 | 1419 | 0,53 | 2,761699522 | 6,69901334 | 24,55396991 | 7,22573E-07 | 8,94633E-06 |
| b2796 | 1290 | 0,52 | 1,835408836 | 9,170934356 | 24,53619438 | 7,2927E-07 | 9,00437E-06 |
| b4051 | 984 | 0,53 | -1,602109062 | 9,217276733 | 24,50597521 | 7,40797E-07 | 9,12158E-06 |
| b1342 | 984 | 0,52 | -1,076828576 | 7,336186982 | 24,4786252 | 7,51388E-07 | 9,22663E-06 |
| b2040 | 900 | 0,47 | 1,051744257 | 6,829590744 | 24,46130746 | 7,58172E-07 | 9,2845E-06 |
| b2317 | 660 | 0,49 | 1,36353031 | 5,556177088 | 24,420564 | 7,74377E-07 | 9,4571E-06 |
| b3972 | 1029 | 0,46 | 0,949347595 | 6,497256246 | 24,40916752 | 7,78971E-07 | 9,48736E-06 |
| b1298 | 765 | 0,52 | 2,364888835 | 5,65792718 | 24,32923362 | 8,11974E-07 | 9,86251E-06 |
| b1651 | 408 | 0,51 | -1,027406033 | 7,730219911 | 24,30482871 | 8,22327E-07 | 9,96126E-06 |
| b2455 | 1404 | 0,57 | 1,6268965 | 3,048736258 | 24,21878633 | 8,59894E-07 | 1,03883E-05 |
| b3115 | 1209 | 0,48 | -4,095396861 | 5,943170087 | 24,19822233 | 8,69125E-07 | 1,04716E-05 |

| | | | | | | | |
|---|---|---|---|---|---|---|---|
| b2525 | 336 | 0,52 | 1,307092246 | 5,701733989 | 24,18858028 | 8,73487E-07 | 1,04959E-05 |
| b3211 | 930 | 0,56 | -1,87968208 | 5,108362369 | 24,17099826 | 8,81498E-07 | 1,05438E-05 |
| b2306 | 774 | 0,52 | 1,918536214 | 4,460930137 | 24,16950638 | 8,82181E-07 | 1,05438E-05 |
| b2329 | 1086 | 0,57 | 1,130943811 | 7,019246214 | 24,13700214 | 8,97197E-07 | 1,06948E-05 |
| b2827 | 795 | 0,51 | 1,088882131 | 6,441329161 | 24,10663731 | 9,11456E-07 | 1,08359E-05 |
| b0336 | 1260 | 0,54 | 2,166976654 | 4,968002144 | 24,09545961 | 9,16762E-07 | 1,08568E-05 |
| b0426 | 492 | 0,5 | 0,97905434 | 8,572563902 | 24,09274532 | 9,18055E-07 | 1,08568E-05 |
| b1805 | 1686 | 0,51 | 1,609872107 | 6,287200579 | 24,06179233 | 9,32932E-07 | 1,10037E-05 |
| b2247 | 1206 | 0,52 | -1,555493543 | 4,806003872 | 24,0232796 | 9,51779E-07 | 1,11965E-05 |
| b2008 | 1059 | 0,51 | -0,911597612 | 6,488195362 | 23,97360529 | 9,76655E-07 | 1,14591E-05 |
| b1226 | 711 | 0,56 | -3,95689538 | 8,884629244 | 23,96136724 | 9,82883E-07 | 1,1502E-05 |
| b1586 | 306 | 0,56 | -1,723786808 | 6,023254687 | 23,88639704 | 1,02191E-06 | 1,19276E-05 |
| b3922 | 300 | 0,46 | -1,199639507 | 6,787420079 | 23,85227251 | 1,04019E-06 | 1,21025E-05 |
| b2584 | 2661 | 0,54 | -1,52975106 | 9,987420774 | 23,84837807 | 1,0423E-06 | 1,21025E-05 |
| b0409 | 972 | 0,52 | 0,982863299 | 7,765866449 | 23,83542155 | 1,04934E-06 | 1,21528E-05 |
| b2201 | 624 | 0,56 | -1,901623762 | 6,014738073 | 23,8209802 | 1,05724E-06 | 1,22127E-05 |
| b3169 | 1488 | 0,53 | 1,073995857 | 9,068459937 | 23,73278851 | 1,10681E-06 | 1,27525E-05 |
| b2878 | 3099 | 0,52 | -1,232745144 | 4,869090792 | 23,72428097 | 1,11171E-06 | 1,27649E-05 |
| b1208 | 852 | 0,53 | 0,986255439 | 6,734204616 | 23,72104536 | 1,11358E-06 | 1,27649E-05 |
| b1333 | 951 | 0,51 | -1,7959976 | 10,57055897 | 23,6729729 | 1,14175E-06 | 1,30487E-05 |
| b4003 | 1398 | 0,54 | -1,356526686 | 5,859074975 | 23,66890762 | 1,14416E-06 | 1,30487E-05 |
| b2891 | 1099 | 0,54 | 1,377124789 | 8,022579897 | 23,62359576 | 1,17142E-06 | 1,33257E-05 |
| b3955 | 1734 | 0,5 | 1,252704481 | 5,754078223 | 23,57607875 | 1,20071E-06 | 1,36243E-05 |
| b3611 | 432 | 0,5 | 1,423116928 | 6,974946569 | 23,5673226 | 1,20619E-06 | 1,36518E-05 |
| b0212 | 756 | 0,4 | -0,978078148 | 6,208329962 | 23,56058127 | 1,21042E-06 | 1,36652E-05 |
| b4138 | 1302 | 0,53 | -1,880704732 | 10,12486228 | 23,55372135 | 1,21474E-06 | 1,36796E-05 |
| b2896 | 408 | 0,53 | -0,94595339 | 6,554752109 | 23,5288471 | 1,23055E-06 | 1,38229E-05 |
| b3939 | 1161 | 0,55 | 1,293259177 | 5,29698621 | 23,3715804 | 1,33537E-06 | 1,49628E-05 |
| b3091 | 1488 | 0,54 | -1,482289518 | 6,244114845 | 23,33058048 | 1,36414E-06 | 1,52471E-05 |
| b3424 | 831 | 0,54 | 1,546419675 | 5,198931031 | 23,31970088 | 1,37188E-06 | 1,52815E-05 |
| b4034 | 1191 | 0,51 | -2,357541991 | 10,87158646 | 23,31667132 | 1,37404E-06 | 1,52815E-05 |
| b1062 | 1047 | 0,53 | 2,095355185 | 6,25390999 | 23,20963773 | 1,45267E-06 | 1,61161E-05 |
| b2315 | 1269 | 0,56 | 0,854013427 | 6,558567922 | 23,19137877 | 1,46653E-06 | 1,62296E-05 |
| b1668 | 1605 | 0,48 | -1,137143124 | 7,671300371 | 23,17090566 | 1,48223E-06 | 1,63629E-05 |
| b1045 | 534 | 0,52 | -1,271562458 | 6,217637823 | 23,1530599 | 1,49605E-06 | 1,64749E-05 |
| b4381 | 780 | 0,55 | -1,385967775 | 7,726510007 | 23,14346623 | 1,50353E-06 | 1,65167E-05 |
| b0007 | 1431 | 0,53 | -1,433738051 | 5,887737382 | 23,07830333 | 1,55536E-06 | 1,70443E-05 |
| b2899 | 660 | 0,52 | -1,461190045 | 7,085626428 | 23,07273418 | 1,55987E-06 | 1,70521E-05 |
| b0643 | 483 | 0,53 | -1,379800517 | 10,08212218 | 23,0523017 | 1,57654E-06 | 1,71923E-05 |
| b3572 | 1254 | 0,52 | 1,271839984 | 6,279284836 | 22,90005664 | 1,70647E-06 | 1,85641E-05 |
| b3384 | 1005 | 0,52 | 1,025142826 | 8,234571429 | 22,89297923 | 1,71276E-06 | 1,85874E-05 |
| b0945 | 1011 | 0,47 | 1,986922057 | 3,897401872 | 22,84660007 | 1,75459E-06 | 1,89954E-05 |
| b0867 | 831 | 0,56 | -1,278628507 | 7,72476841 | 22,81011817 | 1,78822E-06 | 1,93128E-05 |
| b1224 | 3744 | 0,55 | -4,005366139 | 13,49970437 | 22,78019807 | 1,81628E-06 | 1,95686E-05 |
| b4469 | 2220 | 0,55 | 1,301919229 | 5,410539622 | 22,73046672 | 1,86389E-06 | 2,00335E-05 |
| b1478 | 1011 | 0,52 | -1,460105808 | 8,279975337 | 22,72433469 | 1,86985E-06 | 2,00494E-05 |
| b2614 | 594 | 0,5 | 1,678012183 | 8,504481652 | 22,66749476 | 1,92599E-06 | 2,06021E-05 |
| b0925 | 1848 | 0,53 | -1,313979716 | 8,809016518 | 22,66071674 | 1,93279E-06 | 2,06257E-05 |
| b4565 | 279 | 0,48 | -1,7225517 | 3,761061474 | 22,64015119 | 1,95359E-06 | 2,07981E-05 |
| b3351 | 555 | 0,57 | 1,779062611 | 4,108621144 | 22,62370311 | 1,97039E-06 | 2,09272E-05 |
| b2014 | 1359 | 0,53 | 1,567694473 | 5,15730483 | 22,61761007 | 1,97665E-06 | 2,0944E-05 |
| b1089 | 174 | 0,54 | 1,133920633 | 7,870029625 | 22,60520119 | 1,98945E-06 | 2,103E-05 |
| b3816 | 951 | 0,53 | 1,492021049 | 6,793819727 | 22,55796937 | 2,03897E-06 | 2,14538E-05 |
| b0723 | 1767 | 0,56 | 2,307301094 | 11,40339559 | 22,55783209 | 2,03912E-06 | 2,14538E-05 |
| b1929 | 1206 | 0,55 | 1,543891487 | 7,493123404 | 22,46821738 | 2,1365E-06 | 2,24257E-05 |
| b4123 | 1341 | 0,52 | -2,167766347 | 5,75309337 | 22,4575252 | 2,14842E-06 | 2,24982E-05 |
| b0819 | 921 | 0,53 | 1,207100591 | 6,758650192 | 22,37179825 | 2,24649E-06 | 2,34703E-05 |
| b1037 | 834 | 0,5 | 1,694645949 | 4,255576514 | 22,36030034 | 2,25998E-06 | 2,35563E-05 |
| b0632 | 1212 | 0,49 | 1,423551287 | 6,957186472 | 22,34825692 | 2,27419E-06 | 2,36495E-05 |
| b0475 | 963 | 0,54 | -1,228579141 | 7,015145539 | 22,33566259 | 2,28916E-06 | 2,375E-05 |
| b0739 | 1266 | 0,52 | 0,891169467 | 6,511647951 | 22,29845246 | 2,33395E-06 | 2,41588E-05 |
| b1716 | 357 | 0,48 | 1,253957915 | 10,00538121 | 22,24823279 | 2,39579E-06 | 2,47418E-05 |
| b2995 | 1179 | 0,54 | -2,48218805 | 7,480370954 | 22,21039651 | 2,44347E-06 | 2,51762E-05 |
| b2602 | 366 | 0,46 | -1,358546189 | 5,025636699 | 22,17988273 | 2,48262E-06 | 2,55208E-05 |
| b3200 | 558 | 0,5 | 0,878706918 | 7,280137951 | 22,13984991 | 2,53493E-06 | 2,5999E-05 |
| b0002 | 2463 | 0,53 | 1,555463511 | 9,05383418 | 22,12136163 | 2,55946E-06 | 2,61906E-05 |
| b4523 | 168 | 0,46 | -1,450559182 | 5,550498541 | 22,10689576 | 2,57882E-06 | 2,63286E-05 |
| b0581 | 1119 | 0,52 | -1,357053398 | 8,338620965 | 22,06645998 | 2,63372E-06 | 2,67985E-05 |
| b3066 | 1746 | 0,52 | 0,989687337 | 7,620718324 | 22,06421441 | 2,6368E-06 | 2,67985E-05 |
| b0914 | 1749 | 0,51 | 0,971930964 | 6,810812561 | 22,04439196 | 2,66417E-06 | 2,70154E-05 |
| b2147 | 1236 | 0,51 | -2,152895969 | 8,604103901 | 22,03446343 | 2,67799E-06 | 2,70504E-05 |
| b3946 | 663 | 0,53 | -1,539522133 | 4,142903773 | 22,0332423 | 2,67969E-06 | 2,70504E-05 |
| b4331 | 555 | 0,51 | 1,525380498 | 3,490820729 | 21,96369452 | 2,77857E-06 | 2,79855E-05 |
| b4006 | 1590 | 0,56 | 2,333560685 | 8,0911931 | 21,94619341 | 2,80403E-06 | 2,81786E-05 |
| b3156 | 504 | 0,56 | -1,70661173 | 7,970172137 | 21,90107852 | 2,87073E-06 | 2,87843E-05 |
| b0168 | 795 | 0,5 | 1,119296403 | 6,840221308 | 21,87832726 | 2,90496E-06 | 2,90626E-05 |
| b0419 | 975 | 0,53 | -1,265706821 | 9,022985625 | 21,86208463 | 2,92966E-06 | 2,92444E-05 |
| b0919 | 894 | 0,52 | -2,807043736 | 9,084760392 | 21,77949067 | 3,05852E-06 | 3,04629E-05 |
| b3201 | 726 | 0,54 | 0,852888668 | 7,191860837 | 21,6746682 | 3,23029E-06 | 3,21023E-05 |
| b0420 | 1863 | 0,54 | 1,0259631 | 7,568863326 | 21,66587513 | 3,24513E-06 | 3,21785E-05 |
| b2844 | 1182 | 0,54 | 1,860206399 | 7,035790636 | 21,56783225 | 3,41532E-06 | 3,37913E-05 |
| b0271 | 1611 | 0,64 | -1,394770124 | 5,246667655 | 21,55431314 | 3,43948E-06 | 3,39554E-05 |
| b0306 | 720 | 0,51 | -3,293936009 | 6,201246144 | 21,54070236 | 3,46398E-06 | 3,406E-05 |
| b4132 | 1335 | 0,49 | -3,284662462 | 5,828282101 | 21,53998398 | 3,46528E-06 | 3,406E-05 |
| b4217 | 207 | 0,43 | 1,029318686 | 8,696675378 | 21,49536466 | 3,54685E-06 | 3,47855E-05 |
| b3669 | 591 | 0,59 | 1,533820241 | 5,002337023 | 21,46787758 | 3,59806E-06 | 3,52107E-05 |
| b3349 | 591 | 0,55 | 1,107025345 | 9,447171684 | 21,42707647 | 3,67544E-06 | 3,58896E-05 |
| b0431 | 1992 | 0,53 | 2,255721828 | 11,11239408 | 21,37943129 | 3,76792E-06 | 3,67126E-05 |
| b1211 | 1083 | 0,53 | 1,10138431 | 6,014281494 | 21,33574855 | 3,85476E-06 | 3,74773E-05 |
| b1914 | 657 | 0,5 | -0,844499259 | 7,483848419 | 21,28395942 | 3,96032E-06 | 3,84202E-05 |
| b0814 | 516 | 0,51 | 1,39685268 | 10,29043284 | 21,24613167 | 4,03925E-06 | 3,90392E-05 |
| b0873 | 1653 | 0,55 | -4,329867323 | 7,66096822 | 21,24151693 | 4,04898E-06 | 3,90392E-05 |
| b3341 | 540 | 0,5 | 1,772160987 | 10,1008732 | 21,24091546 | 4,05025E-06 | 3,90392E-05 |
| b0848 | 378 | 0,5 | -1,623993174 | 3,917304929 | 21,22493736 | 4,08416E-06 | 3,92815E-05 |

| | | | | | | | |
|---|---|---|---|---|---|---|---|
| b4391 | 1668 | 0,55 | 1,226383624 | 9,38223288 | 21,19363964 | 4,1514E-06 | 3,98428E-05 |
| b3932 | 531 | 0,53 | 1,395994161 | 6,831509428 | 21,17418892 | 4,19375E-06 | 4,01632E-05 |
| b2801 | 1317 | 0,48 | -1,534467885 | 5,064263705 | 21,16275472 | 4,21884E-06 | 4,02763E-05 |
| b0451 | 1287 | 0,56 | 1,172002496 | 4,694559208 | 21,16062678 | 4,22353E-06 | 4,02763E-05 |
| b2009 | 474 | 0,47 | -1,249612571 | 8,040437958 | 21,13446645 | 4,28158E-06 | 4,07226E-05 |
| b2779 | 1299 | 0,5 | -1,348101986 | 12,22550402 | 21,13136847 | 4,2885E-06 | 4,07226E-05 |
| b3925 | 1011 | 0,57 | -1,03229258 | 6,036620236 | 21,12587803 | 4,30081E-06 | 4,07531E-05 |
| b0162 | 1158 | 0,53 | -2,839646055 | 9,589629787 | 21,11891989 | 4,31645E-06 | 4,08151E-05 |
| b1414 | 801 | 0,51 | 1,038883779 | 6,195118773 | 21,08115243 | 4,40237E-06 | 4,14671E-05 |
| b4189 | 330 | 0,49 | -2,001624198 | 9,800577235 | 21,08047851 | 4,40392E-06 | 4,14671E-05 |
| b3250 | 1104 | 0,56 | 1,121089227 | 5,934144311 | 21,04665202 | 4,48235E-06 | 4,2067E-05 |
| b0033 | 3222 | 0,56 | 2,323858987 | 8,066253655 | 21,04492433 | 4,4864E-06 | 4,2067E-05 |
| b1443 | 795 | 0,54 | -1,247703772 | 7,240807462 | 21,03308452 | 4,51421E-06 | 4,22394E-05 |
| b4279 | 1218 | 0,36 | 1,798291726 | 3,798666191 | 20,97706272 | 4,64815E-06 | 4,34021E-05 |
| b0124 | 2391 | 0,55 | 1,867942533 | 7,705737167 | 20,94063357 | 4,73738E-06 | 4,41434E-05 |
| b3451 | 846 | 0,54 | 2,30784736 | 4,683677176 | 20,91505824 | 4,80106E-06 | 4,46438E-05 |
| b4522 | 129 | 0,5 | -1,394339624 | 4,992320157 | 20,88833105 | 4,86851E-06 | 4,51546E-05 |
| b0125 | 537 | 0,51 | 1,093647695 | 6,185613602 | 20,88533474 | 4,87614E-06 | 4,51546E-05 |
| b0823 | 2433 | 0,55 | -1,94877951 | 5,386326625 | 20,86028023 | 4,94034E-06 | 4,56548E-05 |
| b0907 | 1089 | 0,5 | 1,147311148 | 7,810281755 | 20,8369237 | 5,00095E-06 | 4,61199E-05 |
| b3599 | 1914 | 0,54 | -1,018977081 | 9,280657572 | 20,83271307 | 5,01195E-06 | 4,61265E-05 |
| b3296 | 621 | 0,51 | 1,481240656 | 11,11068219 | 20,80567083 | 5,08322E-06 | 4,66865E-05 |
| b1040 | 651 | 0,42 | 2,539972283 | 3,671624743 | 20,77558971 | 5,1637E-06 | 4,73286E-05 |
| b1227 | 678 | 0,56 | -3,374545807 | 8,848587224 | 20,75291423 | 5,2252E-06 | 4,77946E-05 |
| b3788 | 1068 | 0,54 | 0,886112635 | 6,26999209 | 20,74689565 | 5,24165E-06 | 4,78474E-05 |
| b1188 | 1533 | 0,5 | -1,680910476 | 9,816139804 | 20,73315686 | 5,27939E-06 | 4,80939E-05 |
| b0180 | 456 | 0,5 | 0,962273338 | 7,62872686 | 20,67527161 | 5,44142E-06 | 4,94695E-05 |
| b3075 | 984 | 0,52 | -1,159366965 | 5,575417696 | 20,64746571 | 5,52102E-06 | 5,00915E-05 |
| b1274 | 2598 | 0,52 | 1,145283739 | 8,987925069 | 20,59766069 | 5,66653E-06 | 5,12064E-05 |
| b3496 | 1470 | 0,52 | -1,0817719 | 9,038539224 | 20,59758705 | 5,66675E-06 | 5,12064E-05 |
| b0835 | 1326 | 0,53 | 1,445553175 | 4,727503864 | 20,59006334 | 5,68906E-06 | 5,13046E-05 |
| b0184 | 3483 | 0,55 | 1,121879071 | 7,992178846 | 20,5622966 | 5,77218E-06 | 5,19496E-05 |
| b4211 | 861 | 0,54 | -1,625663169 | 4,441059625 | 20,55033777 | 5,80835E-06 | 5,21704E-05 |
| b2795 | 1365 | 0,51 | -1,096883531 | 9,465255531 | 20,45176554 | 6,11531E-06 | 5,48177E-05 |
| b3654 | 1392 | 0,55 | 1,717974771 | 6,756836098 | 20,4451154 | 6,1366E-06 | 5,48987E-05 |
| b3320 | 630 | 0,51 | 1,779173598 | 10,70419117 | 20,41005697 | 6,25005E-06 | 5,58022E-05 |
| b2937 | 921 | 0,55 | -1,219436366 | 8,070462037 | 20,37273128 | 6,37315E-06 | 5,67882E-05 |
| b0127 | 927 | 0,5 | 1,016447483 | 5,75762411 | 20,3634849 | 6,40402E-06 | 5,695E-05 |
| b0113 | 765 | 0,56 | 1,88284885 | 4,96876055 | 20,33095309 | 6,51383E-06 | 5,78118E-05 |
| b2146 | 1239 | 0,51 | -2,130507625 | 8,900591771 | 20,27817255 | 6,69601E-06 | 5,93113E-05 |
| b4441 | 184 | 0,5 | 1,615937449 | 8,820956132 | 20,24006727 | 6,83072E-06 | 6,03491E-05 |
| b3832 | 1428 | 0,53 | -0,889326036 | 6,468863674 | 20,23730528 | 6,84059E-06 | 6,03491E-05 |
| b1531 | 384 | 0,46 | 1,506212709 | 3,688597067 | 20,23367849 | 6,85357E-06 | 6,03491E-05 |
| b2526 | 1851 | 0,58 | 1,180553803 | 7,459026338 | 20,22978338 | 6,86754E-06 | 6,03535E-05 |
| b0799 | 2151 | 0,54 | -0,913193253 | 6,085562384 | 20,22287533 | 6,89238E-06 | 6,04533E-05 |
| b0638 | 612 | 0,51 | 1,275171803 | 3,92248418 | 20,21219561 | 6,93097E-06 | 6,0673E-05 |
| b1050 | 228 | 0,5 | -1,43867338 | 8,588368097 | 20,19467298 | 6,99474E-06 | 6,10973E-05 |
| b2705 | 780 | 0,53 | -1,59745339 | 5,845050671 | 20,19140736 | 7,0067E-06 | 6,10973E-05 |
| b0439 | 2355 | 0,52 | 1,267638617 | 9,542230552 | 20,18449872 | 7,03205E-06 | 6,11993E-05 |
| b3170 | 453 | 0,49 | 1,16693946 | 6,333930228 | 20,17829702 | 7,05488E-06 | 6,1279E-05 |
| b2235 | 1131 | 0,51 | 1,121534849 | 7,669392312 | 20,16260444 | 7,113E-06 | 6,16643E-05 |
| b2575 | 738 | 0,52 | 1,488108102 | 3,599686856 | 20,10443335 | 7,33265E-06 | 6,34458E-05 |
| b2244 | 900 | 0,48 | 1,176598147 | 4,346556526 | 20,05597765 | 7,52081E-06 | 6,49485E-05 |
| b1282 | 327 | 0,5 | 1,817015753 | 2,999222932 | 20,04165162 | 7,57736E-06 | 6,5311E-05 |
| b3477 | 945 | 0,58 | -3,294795898 | 5,000491161 | 20,00377288 | 7,72895E-06 | 6,64898E-05 |
| b0338 | 900 | 0,55 | 1,338396217 | 3,626689249 | 19,97836513 | 7,83234E-06 | 6,72501E-05 |
| b3294 | 384 | 0,54 | 1,450270396 | 9,485771675 | 19,93289107 | 8,02085E-06 | 6,8737E-05 |
| b3366 | 327 | 0,56 | -3,208649341 | 5,150401041 | 19,91930591 | 8,07805E-06 | 6,90131E-05 |
| b2203 | 450 | 0,56 | -2,641386807 | 5,978772316 | 19,91792912 | 8,08387E-06 | 6,90131E-05 |
| b1332 | 258 | 0,45 | 1,302756898 | 6,187902325 | 19,90036056 | 8,15849E-06 | 6,95178E-05 |
| b2316 | 915 | 0,55 | 1,29259674 | 8,47959503 | 19,89164169 | 8,19579E-06 | 6,97031E-05 |
| b2157 | 882 | 0,54 | -0,982632226 | 6,812300983 | 19,82340109 | 8,49364E-06 | 7,20995E-05 |
| b2902 | 744 | 0,55 | 0,965252065 | 5,853007795 | 19,78804226 | 8,65223E-06 | 7,32924E-05 |
| b4322 | 1185 | 0,54 | -0,966110001 | 6,389537022 | 19,78480771 | 8,66688E-06 | 7,32924E-05 |
| b0529 | 867 | 0,54 | 1,024554464 | 6,823838257 | 19,74999725 | 8,82618E-06 | 7,439E-05 |
| b2524 | 201 | 0,5 | 1,435092586 | 4,47043483 | 19,7489493 | 8,83102E-06 | 7,439E-05 |
| b2272 | 504 | 0,46 | -1,748473925 | 3,606712008 | 19,7456089 | 8,84647E-06 | 7,439E-05 |
| b0854 | 1113 | 0,51 | 1,817987975 | 5,534524111 | 19,73263734 | 8,90672E-06 | 7,47564E-05 |
| b0331 | 891 | 0,56 | 1,717969895 | 2,8334571 | 19,62477197 | 9,42394E-06 | 7,89497E-05 |
| b1117 | 702 | 0,55 | 1,233428469 | 5,487708165 | 19,59130778 | 9,59045E-06 | 8,01493E-05 |
| b3077 | 450 | 0,46 | -2,150258213 | 4,536877927 | 19,58883163 | 9,60289E-06 | 8,01493E-05 |
| b0406 | 1128 | 0,51 | 1,052429263 | 7,177553495 | 19,5747082 | 9,67414E-06 | 8,0511E-05 |
| b1018 | 1128 | 0,51 | 2,734703752 | 3,497243431 | 19,57312633 | 9,68216E-06 | 8,0511E-05 |
| b1212 | 834 | 0,55 | 1,088541867 | 4,592791447 | 19,56908503 | 9,70266E-06 | 8,05321E-05 |
| b1751 | 678 | 0,48 | -1,768531484 | 4,815148264 | 19,55582201 | 9,77025E-06 | 8,09432E-05 |
| b2608 | 549 | 0,49 | 1,498469695 | 10,18819593 | 19,50249784 | 1,00468E-05 | 8,29975E-05 |
| b0949 | 1908 | 0,52 | 0,868454827 | 7,176753301 | 19,50089642 | 1,00552E-05 | 8,29975E-05 |
| b0634 | 1113 | 0,52 | 0,981939269 | 5,961413483 | 19,47875216 | 1,01725E-05 | 8,38109E-05 |
| b1496 | 1686 | 0,44 | 2,907240139 | 1,468450155 | 19,46402329 | 1,02512E-05 | 8,43047E-05 |
| b2480 | 471 | 0,49 | 1,294943648 | 7,300492408 | 19,42781645 | 1,04474E-05 | 8,56293E-05 |
| b2897 | 267 | 0,49 | -0,964257584 | 6,821088017 | 19,42725213 | 1,04505E-05 | 8,56293E-05 |
| b3631 | 1125 | 0,45 | 0,931672534 | 6,033903549 | 19,41972743 | 1,04918E-05 | 8,58104E-05 |
| b0866 | 324 | 0,54 | -1,238475422 | 6,932676531 | 19,39993734 | 1,06011E-05 | 8,65463E-05 |
| b1906 | 240 | 0,47 | -1,509242781 | 5,613282995 | 19,39205643 | 1,06449E-05 | 8,67462E-05 |
| b0157 | 624 | 0,54 | 1,33618241 | 3,66145827 | 19,36812075 | 1,07792E-05 | 8,76809E-05 |
| b3186 | 312 | 0,48 | 1,736135797 | 8,918091964 | 19,35861493 | 1,08329E-05 | 8,79588E-05 |
| b1334 | 753 | 0,52 | 1,05573022 | 8,167327463 | 19,33691769 | 1,09567E-05 | 8,8803E-05 |
| b3261 | 297 | 0,47 | 1,809118372 | 5,195153256 | 19,31539911 | 1,10809E-05 | 8,96473E-05 |
| b3208 | 729 | 0,52 | -0,898036662 | 6,445473206 | 19,27323555 | 1,13283E-05 | 9,14839E-05 |
| b2797 | 1368 | 0,55 | 2,435972267 | 7,666028335 | 19,26633355 | 1,13694E-05 | 9,16501E-05 |
| b3705 | 1647 | 0,54 | 1,513041955 | 8,268798906 | 19,25855385 | 1,14158E-05 | 9,18591E-05 |
| b2968 | 537 | 0,53 | 2,368114978 | 2,057617514 | 19,20358703 | 1,17492E-05 | 9,43729E-05 |
| b2704 | 372 | 0,55 | -1,724050525 | 3,956364538 | 19,19223827 | 1,18193E-05 | 9,47658E-05 |
| b2747 | 711 | 0,55 | 1,389624383 | 5,693495506 | 19,18642986 | 1,18553E-05 | 9,48849E-05 |

| | | | | | | | |
|---|---|---|---|---|---|---|---|
| b3387 | 837 | 0,5 | 0,93326707 | 6,223470471 | 19,13964913 | 1,21495E-05 | 9,70657E-05 |
| b0440 | 273 | 0,49 | 1,112919691 | 10,00654427 | 19,12078197 | 1,22702E-05 | 9,78556E-05 |
| b2702 | 564 | 0,5 | -1,829414601 | 4,517453231 | 19,1071243 | 1,23583E-05 | 9,83833E-05 |
| b3342 | 375 | 0,53 | 1,611880735 | 9,744744925 | 19,09506504 | 1,24366E-05 | 9,88313E-05 |
| b4004 | 1326 | 0,56 | -1,11013708 | 6,307630517 | 19,07252979 | 1,25843E-05 | 9,98282E-05 |
| b3636 | 168 | 0,4 | 1,734815558 | 6,658702215 | 19,06581392 | 1,26287E-05 | 0,000100003 |
| b1444 | 1425 | 0,53 | -1,519903725 | 9,804566896 | 18,99888699 | 1,30795E-05 | 0,000103346 |
| b2966 | 708 | 0,52 | -1,764163241 | 5,062785259 | 18,99634115 | 1,30969E-05 | 0,000103346 |
| b3321 | 312 | 0,55 | 1,581027148 | 9,270384752 | 18,99219966 | 1,31254E-05 | 0,000103388 |
| b2293 | 651 | 0,57 | -0,938040377 | 7,608053385 | 18,98145876 | 1,31995E-05 | 0,00010379 |
| b2220 | 1386 | 0,49 | -1,503792097 | 5,892710515 | 18,91611197 | 1,36594E-05 | 0,000107218 |
| b2456 | 288 | 0,53 | 2,617887749 | 1,328836562 | 18,89227413 | 1,38312E-05 | 0,000108376 |
| b2202 | 603 | 0,52 | -2,080376169 | 6,592740585 | 18,87875056 | 1,39296E-05 | 0,000108957 |
| b0062 | 1503 | 0,55 | -1,463257402 | 5,787555299 | 18,83562599 | 1,42481E-05 | 0,000111254 |
| b1919 | 987 | 0,54 | -0,879013307 | 7,003849505 | 18,76602808 | 1,47776E-05 | 0,000115188 |
| b3910 | 675 | 0,53 | -1,136732971 | 7,144584397 | 18,75155786 | 1,48902E-05 | 0,000115864 |
| b0308 | 696 | 0,52 | -1,909599959 | 5,978995101 | 18,69482538 | 1,53398E-05 | 0,000119156 |
| b0065 | 765 | 0,54 | -0,760420517 | 6,177677251 | 18,67957354 | 1,5463E-05 | 0,000119905 |
| b0071 | 606 | 0,51 | 1,434121278 | 5,319310884 | 18,64954737 | 1,57085E-05 | 0,000121598 |
| b3645 | 825 | 0,46 | -1,132766766 | 4,780002219 | 18,63713602 | 1,5811E-05 | 0,000122181 |
| b4243 | 387 | 0,53 | -1,132033503 | 9,048244357 | 18,59026306 | 1,62046E-05 | 0,000125007 |
| b1385 | 1500 | 0,53 | -0,981828734 | 6,104240947 | 18,56594602 | 1,64126E-05 | 0,000126394 |
| b1684 | 369 | 0,53 | -1,525444572 | 6,77644578 | 18,50238584 | 1,69692E-05 | 0,000130357 |
| b0470 | 1932 | 0,58 | 1,188791915 | 6,812192324 | 18,5005591 | 1,69854E-05 | 0,000130357 |
| b0513 | 1302 | 0,51 | 1,932324425 | 2,82444362 | 18,48849777 | 1,70933E-05 | 0,000130961 |
| b0476 | 960 | 0,52 | -1,019768986 | 4,950235024 | 18,4364772 | 1,75663E-05 | 0,000134355 |
| b3573 | 474 | 0,54 | -1,70678571 | 5,974282965 | 18,43254585 | 1,76026E-05 | 0,000134374 |
| b0465 | 3363 | 0,52 | 0,797619844 | 6,907580369 | 18,42972375 | 1,76287E-05 | 0,000134374 |
| b3130 | 465 | 0,44 | -1,01275381 | 5,882132232 | 18,41185067 | 1,77948E-05 | 0,00013541 |
| b3478 | 834 | 0,61 | -2,913136423 | 4,344575841 | 18,3903091 | 1,79971E-05 | 0,000136717 |
| b0741 | 522 | 0,51 | 1,171983522 | 10,10212776 | 18,36738582 | 1,82149E-05 | 0,000138138 |
| b2949 | 417 | 0,53 | 1,333187398 | 4,446191742 | 18,34431709 | 1,84368E-05 | 0,000139584 |
| b2066 | 642 | 0,49 | 1,459356446 | 4,66296532 | 18,30670339 | 1,88044E-05 | 0,000142127 |
| b0633 | 1089 | 0,57 | 0,914624786 | 7,393609254 | 18,23305448 | 1,95457E-05 | 0,000147481 |
| b0174 | 762 | 0,5 | 0,997847798 | 7,133132023 | 18,22112348 | 1,96685E-05 | 0,000148159 |
| b0964 | 663 | 0,51 | -1,450357125 | 4,908210777 | 18,21698076 | 1,97114E-05 | 0,000148232 |
| b0591 | 1251 | 0,58 | 2,617717218 | 2,209745733 | 18,1830824 | 2,00653E-05 | 0,000150641 |
| b4581 | 788 | 0,54 | -1,230971681 | 7,081853365 | 18,1450701 | 2,04699E-05 | 0,000153421 |
| b1133 | 1107 | 0,54 | 1,067618085 | 6,780278353 | 18,08601521 | 2,11147E-05 | 0,00015799 |
| b3295 | 990 | 0,52 | 1,433443769 | 11,73014565 | 18,03080856 | 2,17359E-05 | 0,000162367 |
| b4040 | 873 | 0,52 | 1,022321615 | 6,382712926 | 17,99883832 | 2,2104E-05 | 0,000164842 |
| b0639 | 642 | 0,5 | 1,028757986 | 5,348163469 | 17,95890239 | 2,25726E-05 | 0,000168057 |
| b2712 | 2253 | 0,58 | -1,167283594 | 5,73112296 | 17,91282364 | 2,31258E-05 | 0,000171643 |
| b3538 | 1680 | 0,53 | -0,866673306 | 6,64121229 | 17,91241065 | 2,31308E-05 | 0,000171643 |
| b1488 | 582 | 0,53 | 2,731123456 | 3,161026761 | 17,89731767 | 2,33149E-05 | 0,000172723 |
| b0067 | 1611 | 0,58 | 1,484873698 | 3,503672272 | 17,86035164 | 2,37723E-05 | 0,000175821 |
| b2942 | 1155 | 0,53 | 2,265487978 | 7,748871697 | 17,83688497 | 2,40672E-05 | 0,000177512 |
| b4328 | 1173 | 0,57 | 1,049602023 | 5,627619939 | 17,83586199 | 2,40802E-05 | 0,000177512 |
| b1511 | 1593 | 0,52 | 1,329681729 | 7,12267256 | 17,82476634 | 2,4221E-05 | 0,000178257 |
| b2713 | 528 | 0,52 | 2,039909496 | 1,807703369 | 17,80401982 | 2,44865E-05 | 0,000179916 |
| b3453 | 1317 | 0,54 | 1,852163913 | 7,32838999 | 17,78592659 | 2,47205E-05 | 0,000181338 |
| b4364 | 771 | 0,52 | -1,639985074 | 4,965107353 | 17,77393367 | 2,48768E-05 | 0,000182186 |
| b4317 | 2637 | 0,49 | 1,297058361 | 5,056008374 | 17,76551596 | 2,49871E-05 | 0,000182574 |
| b0025 | 942 | 0,54 | 1,001425977 | 7,129915368 | 17,76367933 | 2,50113E-05 | 0,000182574 |
| b2041 | 1086 | 0,44 | 1,08854339 | 7,001836771 | 17,74852481 | 2,52113E-05 | 0,000183683 |
| b2398 | 345 | 0,5 | -1,067558752 | 6,28006593 | 17,74597166 | 2,52451E-05 | 0,000183683 |
| b0818 | 1119 | 0,52 | 1,088454254 | 4,834237325 | 17,58890424 | 2,74183E-05 | 0,000199172 |
| b2913 | 1233 | 0,53 | 1,112763862 | 7,405959612 | 17,57576255 | 2,76085E-05 | 0,000200229 |
| b3786 | 1131 | 0,53 | 1,142428676 | 5,90327685 | 17,53420155 | 2,82186E-05 | 0,000204322 |
| b2517 | 1155 | 0,53 | 1,070633988 | 6,433980037 | 17,51364227 | 2,85254E-05 | 0,000206211 |
| b3297 | 390 | 0,52 | 1,292935832 | 10,24836835 | 17,50206402 | 2,86996E-05 | 0,000207136 |
| b4686 | 111 | 0,51 | 1,35500487 | 5,009912748 | 17,49853962 | 2,87528E-05 | 0,000207187 |
| b3827 | 900 | 0,56 | 1,261208222 | 3,716993541 | 17,46449827 | 2,92723E-05 | 0,000210592 |
| b2697 | 2631 | 0,54 | 1,247870786 | 9,897349828 | 17,42080104 | 2,9953E-05 | 0,000215143 |
| b4377 | 1074 | 0,54 | 0,913856886 | 7,080163002 | 17,35328514 | 3,10361E-05 | 0,000222427 |
| b3242 | 204 | 0,48 | 1,799894358 | 2,50470342 | 17,35143375 | 3,10664E-05 | 0,000222427 |
| b0159 | 699 | 0,52 | 0,813757249 | 7,564801607 | 17,30133121 | 3,18963E-05 | 0,000228005 |
| b2741 | 993 | 0,52 | 1,237031628 | 12,03370605 | 17,27796209 | 3,2291E-05 | 0,000230459 |
| b0457 | 1551 | 0,48 | -0,977691761 | 6,441122412 | 17,25662815 | 3,26556E-05 | 0,000232691 |
| b3318 | 303 | 0,48 | 1,609527912 | 9,603744858 | 17,24330924 | 3,28854E-05 | 0,000233956 |
| b1126 | 1137 | 0,51 | 0,900351003 | 6,096273032 | 17,20584526 | 3,35403E-05 | 0,000238237 |
| b3701 | 1101 | 0,54 | 0,76569131 | 7,348768372 | 17,18070828 | 3,3987E-05 | 0,000241028 |
| b1241 | 2676 | 0,51 | -1,088795996 | 11,66601905 | 17,17569547 | 3,40768E-05 | 0,000241283 |
| b1717 | 198 | 0,5 | 1,06928414 | 8,838531481 | 17,16977475 | 3,41832E-05 | 0,000241655 |
| b3450 | 1071 | 0,58 | 1,288056568 | 6,657282202 | 17,10495033 | 3,537E-05 | 0,000249651 |
| b4013 | 930 | 0,5 | 2,332698139 | 4,588090309 | 17,0680352 | 3,60642E-05 | 0,000254151 |
| b3637 | 237 | 0,53 | 1,493646126 | 8,344958938 | 16,99837649 | 3,74118E-05 | 0,000263223 |
| b2299 | 543 | 0,53 | 0,788312493 | 6,507501543 | 16,99547256 | 3,74691E-05 | 0,000263223 |
| b0463 | 1194 | 0,54 | 0,877827623 | 7,926467069 | 16,9846282 | 3,76837E-05 | 0,000264317 |
| b4057 | 357 | 0,5 | -1,066441877 | 7,434498286 | 16,97039821 | 3,79672E-05 | 0,000265889 |
| b0469 | 552 | 0,54 | 1,794741575 | 5,594901691 | 16,94900132 | 3,83975E-05 | 0,000268483 |
| b3773 | 894 | 0,58 | -1,317359168 | 5,946410292 | 16,91166443 | 3,91602E-05 | 0,000273103 |
| b0923 | 705 | 0,52 | 0,7504868 | 6,359810417 | 16,91070055 | 3,91801E-05 | 0,000273103 |
| b2627 | 2190 | 0,49 | -0,725914117 | 7,042683639 | 16,88370185 | 3,97414E-05 | 0,000276585 |
| b1222 | 1797 | 0,53 | -1,478253893 | 7,935051578 | 16,85429694 | 4,03618E-05 | 0,000280468 |
| b3741 | 1890 | 0,54 | 0,838966538 | 6,852014125 | 16,84658355 | 4,05262E-05 | 0,000281174 |
| b3260 | 966 | 0,52 | 1,43040863 | 6,853012915 | 16,83572411 | 4,07588E-05 | 0,000282351 |
| b3144 | 2517 | 0,48 | -1,070935487 | 6,327993015 | 16,82356934 | 4,10207E-05 | 0,000283726 |
| b2512 | 1179 | 0,53 | 0,913701912 | 8,672567991 | 16,81785873 | 4,11443E-05 | 0,000284143 |
| b2395 | 2190 | 0,49 | 0,881343907 | 5,553032966 | 16,80163485 | 4,14975E-05 | 0,000286141 |
| b1217 | 231 | 0,45 | -1,106845346 | 5,119650706 | 16,79229817 | 4,17022E-05 | 0,000287111 |
| b0142 | 480 | 0,5 | 1,743445288 | 3,754250785 | 16,78685961 | 4,18219E-05 | 0,000287494 |
| b2107 | 339 | 0,5 | 1,227537182 | 4,992977775 | 16,78250482 | 4,1918E-05 | 0,000287713 |
| b2586 | 324 | 0,54 | 1,720642663 | 4,470158801 | 16,7276202 | 4,31482E-05 | 0,000295601 |

| | | | | | | | |
|---|---|---|---|---|---|---|---|
| b3820 | 468 | 0,53 | 2,960030746 | 4,034633551 | 16,72168474 | 4,32834E-05 | 0,000295601 |
| b0755 | 753 | 0,51 | -1,05076057 | 10,79708721 | 16,7213467 | 4,32911E-05 | 0,000295601 |
| b3973 | 966 | 0,49 | 1,022585083 | 5,513890672 | 16,71959558 | 4,33311E-05 | 0,000295601 |
| b4318 | 531 | 0,5 | 1,533051698 | 3,348478248 | 16,71611459 | 4,34107E-05 | 0,000295694 |
| b2285 | 501 | 0,55 | 1,138447202 | 9,03065662 | 16,69898535 | 4,38044E-05 | 0,000297923 |
| b2972 | 810 | 0,52 | -1,359006085 | 4,159219432 | 16,59471325 | 4,62798E-05 | 0,000314282 |
| b2817 | 1254 | 0,54 | 1,096733809 | 6,108927706 | 16,54931677 | 4,74011E-05 | 0,000321409 |
| b3703 | 141 | 0,52 | 1,659505889 | 5,383139625 | 16,54490206 | 4,75115E-05 | 0,000321672 |
| b3319 | 606 | 0,53 | 1,627502531 | 10,43499936 | 16,52861418 | 4,79214E-05 | 0,000323957 |
| b3603 | 1656 | 0,55 | 3,342976251 | 6,300721173 | 16,52252527 | 4,80755E-05 | 0,00032451 |
| b3168 | 2673 | 0,53 | 0,971032704 | 10,76672303 | 16,46357369 | 4,95938E-05 | 0,000334255 |
| b1614 | 1509 | 0,49 | -1,043569859 | 10,01576976 | 16,45399614 | 4,9845E-05 | 0,000335043 |
| b4515 | 114 | 0,52 | -1,762638935 | 6,22410889 | 16,4534177 | 4,98602E-05 | 0,000335043 |
| b2137 | 762 | 0,55 | -0,978849133 | 6,510382938 | 16,43659692 | 5,03046E-05 | 0,000337523 |
| b0724 | 717 | 0,52 | 1,810427914 | 10,34604763 | 16,42035551 | 5,07374E-05 | 0,000339873 |
| b1012 | 1149 | 0,55 | 2,678017554 | 1,295343514 | 16,41777825 | 5,08065E-05 | 0,000339873 |
| b4175 | 1005 | 0,53 | 0,885245002 | 8,93082291 | 16,37886032 | 5,18604E-05 | 0,000346406 |
| b1436 | 231 | 0,49 | 2,321806918 | 2,34177184 | 16,36457483 | 5,22528E-05 | 0,000348507 |
| b0072 | 1401 | 0,56 | 1,478590059 | 5,549313883 | 16,32039747 | 5,34851E-05 | 0,000356197 |
| b1888 | 1965 | 0,53 | 1,629297541 | 2,844406625 | 16,297658 | 5,41308E-05 | 0,000359962 |
| b1124 | 795 | 0,5 | 1,08115566 | 5,397949121 | 16,25725341 | 5,52975E-05 | 0,000367175 |
| b2944 | 498 | 0,55 | 1,865957152 | 2,669689545 | 16,23879292 | 5,58389E-05 | 0,000370222 |
| b1714 | 984 | 0,54 | 0,94218133 | 7,328474203 | 16,22462477 | 5,62581E-05 | 0,00037245 |
| b3162 | 1890 | 0,56 | 1,068221375 | 7,358171538 | 16,07570858 | 6,08597E-05 | 0,00040232 |
| b1609 | 1302 | 0,51 | 0,920345562 | 4,782426098 | 16,01895356 | 6,27115E-05 | 0,000413951 |
| b1221 | 651 | 0,52 | -1,267297205 | 8,215825258 | 16,00821824 | 6,30681E-05 | 0,000415265 |
| b1768 | 642 | 0,53 | 0,934167138 | 5,503525869 | 16,00564454 | 6,31539E-05 | 0,000415265 |
| b2745 | 1050 | 0,52 | 0,72567156 | 7,829736128 | 16,00460724 | 6,31885E-05 | 0,000415265 |
| b0128 | 771 | 0,51 | 0,863731931 | 5,828269096 | 15,99969038 | 6,33528E-05 | 0,000415736 |
| b4620 | 279 | 0,49 | 2,306618534 | 1,649833399 | 15,95545126 | 6,48508E-05 | 0,000424943 |
| b3155 | 303 | 0,54 | -1,162239574 | 4,525701411 | 15,9371944 | 6,54793E-05 | 0,000428435 |
| b3257 | 243 | 0,54 | 2,028630779 | 1,543199163 | 15,91856344 | 6,6127E-05 | 0,000432042 |
| b4402 | 141 | 0,43 | 0,896847149 | 6,791089588 | 15,90344429 | 6,66573E-05 | 0,000434307 |
| b3543 | 1020 | 0,56 | 1,644723063 | 7,96976143 | 15,90315589 | 6,66675E-05 | 0,000434307 |
| b2762 | 735 | 0,51 | 1,502738874 | 3,21811147 | 15,89876015 | 6,68225E-05 | 0,000434686 |
| b4039 | 498 | 0,53 | 0,841041209 | 6,065620724 | 15,85872468 | 6,82511E-05 | 0,000443336 |
| b1281 | 738 | 0,53 | 1,268392931 | 4,93710497 | 15,83089503 | 6,92623E-05 | 0,000449253 |
| b2717 | 471 | 0,54 | -1,034249383 | 5,106684334 | 15,82689885 | 6,94087E-05 | 0,000449552 |
| b3078 | 1434 | 0,5 | -2,154367005 | 5,372419859 | 15,77394118 | 7,13789E-05 | 0,000461645 |
| b3414 | 576 | 0,53 | 1,324152981 | 6,701662315 | 15,76060301 | 7,18839E-05 | 0,000464242 |
| b1831 | 699 | 0,53 | 1,29255314 | 7,991577088 | 15,74756453 | 7,23811E-05 | 0,00046678 |
| b0181 | 789 | 0,53 | 0,764667988 | 8,366675278 | 15,69178332 | 7,45473E-05 | 0,000480059 |
| b3389 | 1089 | 0,54 | 0,783022676 | 8,036701094 | 15,68299646 | 7,48944E-05 | 0,000481602 |
| b2294 | 495 | 0,48 | -0,789501827 | 7,618488091 | 15,67306494 | 7,52888E-05 | 0,000483444 |
| b3020 | 1608 | 0,52 | 1,128339625 | 6,030050185 | 15,6673781 | 7,55155E-05 | 0,000484207 |
| b1507 | 1323 | 0,48 | -0,960100301 | 6,223514194 | 15,60859436 | 7,78997E-05 | 0,000498781 |
| b0171 | 726 | 0,53 | 0,901799095 | 7,24428175 | 15,60045658 | 7,82357E-05 | 0,000500217 |
| b3281 | 819 | 0,49 | 0,882022258 | 6,22934302 | 15,59212836 | 7,8581E-05 | 0,00050171 |
| b0397 | 3147 | 0,54 | 0,749118504 | 6,350436928 | 15,57653119 | 7,9232E-05 | 0,000505146 |
| b2989 | 867 | 0,53 | -0,713946904 | 7,934675749 | 15,57146658 | 7,94445E-05 | 0,000505781 |
| b4348 | 1395 | 0,4 | 1,384562012 | 4,783273482 | 15,55286821 | 8,02298E-05 | 0,000510057 |
| b2477 | 1035 | 0,55 | 0,845031146 | 8,394928893 | 15,5072622 | 8,21889E-05 | 0,000521506 |
| b2607 | 768 | 0,53 | 1,261829793 | 10,37115226 | 15,50554965 | 8,22634E-05 | 0,000521506 |
| b3905 | 837 | 0,52 | -1,25976932 | 4,603498164 | 15,48706322 | 8,30719E-05 | 0,000525887 |
| b3348 | 219 | 0,53 | 1,02159391 | 4,583978177 | 15,47922163 | 8,34172E-05 | 0,000527329 |
| b1924 | 1407 | 0,52 | 1,917118356 | 2,35462327 | 15,47070616 | 8,37939E-05 | 0,000528964 |
| b0379 | 309 | 0,52 | 1,816648075 | 2,717130648 | 15,44237906 | 8,50592E-05 | 0,000536196 |
| b3183 | 1173 | 0,53 | 0,946898561 | 6,98755523 | 15,40190504 | 8,69006E-05 | 0,000547035 |
| b2742 | 1140 | 0,52 | 1,071097081 | 11,40925614 | 15,39295731 | 8,73131E-05 | 0,00054886 |
| b2104 | 789 | 0,54 | 1,549258056 | 2,696036653 | 15,3825407 | 8,77958E-05 | 0,000551121 |
| b3165 | 270 | 0,52 | 1,097091034 | 8,275182773 | 15,3793353 | 8,79448E-05 | 0,000551285 |
| b3447 | 1743 | 0,55 | -0,921972153 | 7,944178583 | 15,37626537 | 8,80878E-05 | 0,00055141 |
| b0810 | 660 | 0,53 | 1,365955192 | 6,147307935 | 15,35730006 | 8,89765E-05 | 0,000556196 |
| b3158 | 996 | 0,57 | -1,856875358 | 5,710600468 | 15,30460351 | 9,14933E-05 | 0,000571132 |
| b3951 | 2298 | 0,53 | -1,049929416 | 4,961075004 | 15,28881625 | 9,22611E-05 | 0,000575124 |
| b0393 | 912 | 0,52 | 1,123600233 | 5,761812037 | 15,25284867 | 9,40348E-05 | 0,000585367 |
| b3522 | 1014 | 0,53 | -1,102499455 | 7,52686396 | 15,23365715 | 9,49952E-05 | 0,000590525 |
| b4340 | 1413 | 0,55 | 1,077600986 | 4,758514658 | 15,19043496 | 9,71945E-05 | 0,00060336 |
| b3298 | 357 | 0,52 | 1,186619187 | 10,27997973 | 15,18074145 | 9,76947E-05 | 0,000604794 |
| b4151 | 360 | 0,54 | -1,416423416 | 7,843088639 | 15,18072741 | 9,76954E-05 | 0,000604794 |
| b3670 | 291 | 0,48 | 1,599129137 | 4,963189332 | 15,1520034 | 9,9193E-05 | 0,000613218 |
| b2582 | 420 | 0,5 | 1,142659586 | 4,782976323 | 15,13714283 | 9,99768E-05 | 0,000617212 |
| b1752 | 708 | 0,52 | -1,321074009 | 4,511725669 | 15,13395948 | 0,000100146 | 0,000617403 |
| b3114 | 2295 | 0,53 | -2,558969998 | 5,856421438 | 15,11723954 | 0,000101036 | 0,00062204 |
| b1627 | 582 | 0,52 | 1,349545214 | 3,984648124 | 15,08404875 | 0,000102829 | 0,000632205 |
| b1971 | 1005 | 0,52 | -1,079293138 | 6,560041845 | 15,07318814 | 0,000103422 | 0,000634982 |
| b2994 | 1704 | 0,53 | -1,604986707 | 8,412212899 | 15,07044466 | 0,000103572 | 0,000635036 |
| b2454 | 837 | 0,59 | 2,177964537 | 2,322724939 | 15,05918723 | 0,000104192 | 0,000637962 |
| b4014 | 1602 | 0,52 | 1,461888665 | 12,90858033 | 15,05442519 | 0,000104455 | 0,000638701 |
| b3452 | 888 | 0,56 | 2,317402976 | 4,207237761 | 15,05127934 | 0,000104629 | 0,000638895 |
| b0954 | 519 | 0,53 | 1,076926421 | 8,210163325 | 15,04412166 | 0,000105027 | 0,000640449 |
| b3494 | 336 | 0,47 | -1,295293214 | 8,16243495 | 15,03724231 | 0,00010541 | 0,000641153 |
| b2529 | 387 | 0,5 | 1,316694691 | 7,043577302 | 15,03692111 | 0,000105428 | 0,000641153 |
| b0474 | 645 | 0,52 | 1,339373596 | 7,027167557 | 15,03396292 | 0,000105594 | 0,000641288 |
| b3739 | 381 | 0,52 | 1,224622267 | 6,750705431 | 15,01737555 | 0,000106526 | 0,000646074 |
| b3072 | 1521 | 0,55 | 1,066173084 | 4,668155329 | 14,99525652 | 0,000107782 | 0,000651947 |
| b4074 | 1659 | 0,57 | -1,860777602 | 4,202836107 | 14,99519894 | 0,000107785 | 0,000651947 |
| b1583 | 342 | 0,51 | -0,844516834 | 5,951902443 | 14,9871069 | 0,000108248 | 0,000653866 |
| b0328 | 672 | 0,49 | -1,311018016 | 4,383786267 | 14,93796547 | 0,000111104 | 0,000670216 |
| b0676 | 1221 | 0,53 | -0,742548431 | 7,362587331 | 14,93396732 | 0,00011134 | 0,000670735 |
| b2450 | 810 | 0,5 | 1,129268944 | 6,665123481 | 14,9060341 | 0,000113001 | 0,000679826 |
| b0176 | 1353 | 0,51 | 0,777337041 | 8,108231501 | 14,90329959 | 0,000113165 | 0,000679899 |
| b2541 | 813 | 0,56 | 1,875015534 | 3,131889189 | 14,86077623 | 0,000115745 | 0,000694469 |
| b3787 | 1263 | 0,54 | 0,903338824 | 6,303865413 | 14,85298044 | 0,000116224 | 0,000696413 |

| | | | | | | | |
|---|---|---|---|---|---|---|---|
| b2421 | 912 | 0,56 | 1,265219405 | 4,794744788 | 14,84508451 | 0,000116712 | 0,000698401 |
| b2606 | 348 | 0,48 | 1,176157927 | 9,199663772 | 14,82403346 | 0,000118022 | 0,000705298 |
| b1415 | 1440 | 0,51 | 1,898954005 | 10,98753515 | 14,81520241 | 0,000118576 | 0,000707665 |
| b3597 | 1137 | 0,53 | -1,19492744 | 6,644646724 | 14,80804928 | 0,000119026 | 0,00070941 |
| b1338 | 1311 | 0,55 | 1,336387378 | 3,097107832 | 14,79728875 | 0,000119707 | 0,000712522 |
| b0905 | 1761 | 0,52 | 1,068698236 | 5,456532482 | 14,78143511 | 0,000120718 | 0,000717584 |
| b0436 | 1299 | 0,51 | 1,387130134 | 9,607527158 | 14,77358248 | 0,000121222 | 0,000719625 |
| b4293 | 522 | 0,55 | 2,280740218 | 2,743104194 | 14,76650967 | 0,000121677 | 0,000721256 |
| b0628 | 966 | 0,53 | 0,901334149 | 7,599646738 | 14,76432284 | 0,000121819 | 0,000721256 |
| b2171 | 573 | 0,5 | 1,02139206 | 5,167294103 | 14,75341434 | 0,000122525 | 0,000724484 |
| b2936 | 759 | 0,52 | -1,037820599 | 8,220474876 | 14,74375932 | 0,000123154 | 0,000726347 |
| b1537 | 519 | 0,49 | 1,475835136 | 3,951932354 | 14,74360547 | 0,000123165 | 0,000726347 |
| b4153 | 735 | 0,54 | -1,479550987 | 9,336757785 | 14,73517651 | 0,000123716 | 0,000728642 |
| b3476 | 1575 | 0,53 | -2,690539264 | 6,86467463 | 14,72067955 | 0,000124671 | 0,000733303 |
| b3401 | 879 | 0,53 | 0,719713888 | 6,930711438 | 14,70182834 | 0,000125924 | 0,000739702 |
| b3356 | 405 | 0,52 | -0,673230431 | 6,811035495 | 14,69347781 | 0,000126483 | 0,000742013 |
| b4005 | 1290 | 0,58 | 1,856411773 | 8,304589952 | 14,68956226 | 0,000126746 | 0,000742584 |
| b2828 | 876 | 0,53 | 0,879774379 | 5,925782083 | 14,65443662 | 0,00012913 | 0,000755564 |
| b1524 | 927 | 0,52 | 1,317481461 | 4,333200915 | 14,63639878 | 0,000130372 | 0,000761835 |
| b0871 | 1719 | 0,54 | -1,072954225 | 9,683433045 | 14,6194379 | 0,000131551 | 0,000767721 |
| b4486 | 2937 | 0,5 | -0,793082378 | 6,359956315 | 14,60509899 | 0,000132555 | 0,000772046 |
| b4388 | 969 | 0,57 | 0,921764847 | 5,675703802 | 14,60395126 | 0,000132636 | 0,000772046 |
| b3161 | 1245 | 0,53 | -4,169847034 | 10,05184537 | 14,56974478 | 0,000135066 | 0,000785169 |
| b1021 | 414 | 0,44 | 1,779611266 | 2,617362348 | 14,5284587 | 0,000138058 | 0,000801524 |
| b2877 | 579 | 0,43 | -1,908174691 | 6,636901834 | 14,52245484 | 0,000138499 | 0,000803042 |
| b1999 | 552 | 0,58 | -1,409501263 | 3,558898163 | 14,48558546 | 0,000141236 | 0,000817856 |
| b3506 | 567 | 0,46 | -1,392064625 | 10,65807876 | 14,46582923 | 0,000142725 | 0,000825413 |
| b0478 | 1677 | 0,55 | -1,193789739 | 8,56270276 | 14,46270113 | 0,000142963 | 0,000825719 |
| b1849 | 1179 | 0,54 | 1,769646964 | 7,305481507 | 14,43700643 | 0,000144926 | 0,000835984 |
| b2302 | 648 | 0,51 | -0,872325374 | 6,613997244 | 14,39801226 | 0,000147958 | 0,000852377 |
| b1767 | 1017 | 0,51 | 1,05620243 | 5,775945722 | 14,36289491 | 0,000150744 | 0,000867309 |
| b2609 | 249 | 0,51 | 1,16881128 | 8,139814507 | 14,35696354 | 0,000151219 | 0,00086893 |
| b0270 | 1383 | 0,62 | -1,200132709 | 4,336934151 | 14,34767389 | 0,000151967 | 0,00087211 |
| b1725 | 861 | 0,52 | -1,186668887 | 9,666894865 | 14,34331588 | 0,00015232 | 0,000873013 |
| b1963 | 366 | 0,5 | 2,540434377 | 3,02956863 | 14,30874322 | 0,000155143 | 0,000886877 |
| b0825 | 663 | 0,54 | -1,14076602 | 4,638512114 | 14,30659123 | 0,00015532 | 0,000886877 |
| b3838 | 516 | 0,55 | -0,822687306 | 7,521507661 | 14,30644643 | 0,000155332 | 0,000886877 |
| b0822 | 816 | 0,47 | 1,550106213 | 4,503410846 | 14,30345173 | 0,000155579 | 0,000887158 |
| b1871 | 972 | 0,52 | 0,919888737 | 5,993936336 | 14,28689571 | 0,000156954 | 0,000893859 |
| b4158 | 315 | 0,48 | 1,628254095 | 2,338502467 | 14,25893011 | 0,000159303 | 0,000906087 |
| b3734 | 1542 | 0,53 | 1,499339899 | 11,76746477 | 14,25545416 | 0,000159598 | 0,000906612 |
| b0808 | 2226 | 0,53 | -0,84101317 | 6,482890498 | 14,25259729 | 0,00015984 | 0,00090684 |
| b1257 | 507 | 0,41 | -1,782870545 | 5,875504933 | 14,24470442 | 0,000160512 | 0,000909499 |
| b2733 | 2562 | 0,56 | 0,903837252 | 6,483544846 | 14,21521856 | 0,000163047 | 0,000922695 |
| b1136 | 1251 | 0,5 | -1,10341355 | 11,83672726 | 14,17489939 | 0,000166578 | 0,000941491 |
| b0956 | 453 | 0,46 | -0,845242591 | 5,956227122 | 14,1696978 | 0,000167039 | 0,000942908 |
| b2538 | 1362 | 0,53 | 1,047212816 | 4,801537204 | 14,1206523 | 0,000171451 | 0,000965733 |
| b1060 | 255 | 0,45 | -1,761489975 | 8,032976987 | 14,119967 | 0,000171514 | 0,000965733 |
| b1959 | 921 | 0,54 | 1,79714365 | 2,465619288 | 14,08631592 | 0,000174609 | 0,000980844 |
| b1707 | 714 | 0,42 | -1,201010994 | 4,697971127 | 14,08604201 | 0,000174635 | 0,000980844 |
| b3362 | 168 | 0,51 | -1,298820133 | 6,140895203 | 14,06811146 | 0,000176308 | 0,000989 |
| b2893 | 711 | 0,48 | 0,721195239 | 6,554607768 | 14,05790887 | 0,000177267 | 0,000993137 |
| b3479 | 765 | 0,59 | -2,661479729 | 4,274634459 | 14,04176454 | 0,000178795 | 0,001000449 |
| b0492 | 855 | 0,56 | 0,778693354 | 7,43958207 | 14,03531372 | 0,000179409 | 0,001002635 |
| b1125 | 828 | 0,51 | 1,043985326 | 5,053788205 | 14,00102708 | 0,000182711 | 0,001019813 |
| b1637 | 1275 | 0,52 | 0,753832862 | 8,259224847 | 13,98913267 | 0,00018387 | 0,001025008 |
| b3306 | 393 | 0,5 | 1,28669831 | 9,873600233 | 13,98192895 | 0,000184576 | 0,001027665 |
| b1452 | 1062 | 0,51 | 2,332116016 | 4,811431288 | 13,96100169 | 0,000186642 | 0,001037879 |
| b4203 | 450 | 0,5 | 1,105168155 | 9,570985424 | 13,93326516 | 0,000189416 | 0,001052 |
| b1495 | 2373 | 0,46 | 1,36187728 | 3,36468204 | 13,92064003 | 0,000190693 | 0,001057779 |
| b3733 | 864 | 0,53 | 1,345970757 | 10,32184835 | 13,91005124 | 0,00019177 | 0,00106244 |
| b2328 | 825 | 0,55 | 0,892988024 | 5,456465134 | 13,90745907 | 0,000192035 | 0,001062593 |
| b2106 | 825 | 0,52 | 2,016386941 | 2,029929298 | 13,89422912 | 0,000193391 | 0,001068779 |
| b4086 | 981 | 0,52 | 1,42581351 | 2,661801257 | 13,87705999 | 0,000195166 | 0,001077259 |
| b4127 | 273 | 0,41 | -0,881288285 | 7,004700877 | 13,84118045 | 0,000198928 | 0,001096674 |
| b2540 | 321 | 0,55 | 1,781126621 | 1,92170569 | 13,80999116 | 0,000202258 | 0,00111366 |
| b2343 | 285 | 0,52 | -1,308525965 | 7,98246176 | 13,8054236 | 0,00020275 | 0,001115001 |
| b2573 | 576 | 0,49 | -0,873417036 | 8,303136905 | 13,7918858 | 0,000204216 | 0,001121688 |
| b2174 | 714 | 0,44 | 1,528561233 | 3,107423507 | 13,78173205 | 0,000205323 | 0,001126387 |
| b2186 | 1008 | 0,52 | 0,84614379 | 6,01342072 | 13,76706398 | 0,000206932 | 0,001133828 |
| b2960 | 720 | 0,53 | 0,732108255 | 7,104533558 | 13,73893156 | 0,000210055 | 0,001149531 |
| b4220 | 1734 | 0,53 | -0,673867398 | 7,235615947 | 13,73591106 | 0,000210393 | 0,001149977 |
| b1718 | 543 | 0,49 | 0,878063685 | 10,5321823 | 13,7116556 | 0,000213128 | 0,001163506 |
| b2938 | 1977 | 0,54 | 0,883007357 | 9,382747373 | 13,69054351 | 0,000215537 | 0,001175228 |
| b1019 | 1272 | 0,54 | 1,777466025 | 3,826881256 | 13,68626579 | 0,000216029 | 0,001176477 |
| b0154 | 1281 | 0,56 | 0,657035416 | 7,679183408 | 13,65420018 | 0,000219749 | 0,001195286 |
| b4152 | 396 | 0,49 | -1,355195114 | 7,890586783 | 13,59768593 | 0,000226465 | 0,00123032 |
| b0910 | 684 | 0,54 | 0,892902245 | 6,643433788 | 13,59219951 | 0,000227127 | 0,00123137 |
| b0199 | 1032 | 0,53 | 1,917108573 | 5,826166132 | 13,59153876 | 0,000227207 | 0,00123137 |
| b4609 | 143 | 0,41 | -0,990127881 | 5,351399478 | 13,58411074 | 0,000228108 | 0,001234759 |
| b2217 | 651 | 0,48 | 0,644793807 | 7,992667586 | 13,57147344 | 0,000229649 | 0,001241601 |
| b1612 | 1647 | 0,52 | 1,824910185 | 9,562212484 | 13,56888937 | 0,000229965 | 0,001241813 |
| b3390 | 522 | 0,49 | 0,903342227 | 7,155828411 | 13,56185898 | 0,000230828 | 0,001244973 |
| b3457 | 927 | 0,56 | 2,199735496 | 1,767095734 | 13,53630862 | 0,000233992 | 0,00126052 |
| b0610 | 411 | 0,55 | 0,872073098 | 6,189941407 | 13,52367753 | 0,000235572 | 0,001267509 |
| b4414 | 136 | 0,51 | 1,112026933 | 6,931524077 | 13,51636336 | 0,000236492 | 0,001270933 |
| b1269 | 876 | 0,54 | 0,726674726 | 6,089922056 | 13,5125474 | 0,000236974 | 0,001271995 |
| b4021 | 690 | 0,54 | -0,991586764 | 6,12423201 | 13,46731138 | 0,000242756 | 0,001301474 |
| b2666 | 159 | 0,51 | -1,082588312 | 6,236361312 | 13,40764695 | 0,000250601 | 0,001341926 |
| b0985 | 747 | 0,52 | 2,044231469 | 1,652262376 | 13,40105508 | 0,000251483 | 0,001345043 |
| b4201 | 315 | 0,52 | 1,009341747 | 8,949562897 | 13,39340448 | 0,000252511 | 0,001348931 |
| b4200 | 396 | 0,53 | 1,067158943 | 9,380109788 | 13,38500461 | 0,000253644 | 0,001353372 |
| b2045 | 1281 | 0,54 | 1,36151518 | 2,63426861 | 13,36979349 | 0,00025571 | 0,001362771 |
| b4449 | 174 | 0,49 | 1,740707657 | 2,420405632 | 13,36199792 | 0,000256775 | 0,001366822 |

| | | | | | | | |
|---|---|---|---|---|---|---|---|
| b3153 | 519 | 0,5 | -0,829347052 | 6,357161435 | 13,35049459 | 0,000258354 | 0,0013736 |
| b0048 | 480 | 0,53 | -0,953234224 | 6,146007104 | 13,32802593 | 0,000261469 | 0,00138851 |
| b1000 | 921 | 0,53 | -1,088169788 | 9,889968666 | 13,26224068 | 0,000270806 | 0,001436392 |
| b0369 | 975 | 0,54 | 0,728754712 | 7,071747088 | 13,22329012 | 0,000276492 | 0,001464818 |
| b0738 | 429 | 0,52 | 1,107591228 | 4,365378757 | 13,21781554 | 0,000277301 | 0,001467369 |
| b1955 | 816 | 0,52 | -0,916743065 | 7,545693676 | 13,21182403 | 0,000278189 | 0,001470331 |
| b2380 | 1698 | 0,51 | -0,870968834 | 5,751552403 | 13,20423775 | 0,000279317 | 0,001474556 |
| b4234 | 552 | 0,53 | -0,828785777 | 7,786416153 | 13,19670162 | 0,000280442 | 0,001477387 |
| b4255 | 417 | 0,49 | 0,761531037 | 8,323490874 | 13,19533883 | 0,000280646 | 0,001477387 |
| b0186 | 2142 | 0,51 | -0,815131445 | 8,458696042 | 13,19403291 | 0,000280842 | 0,001477387 |
| b0757 | 1149 | 0,53 | -0,969385043 | 8,817229554 | 13,19130816 | 0,000281251 | 0,001477802 |
| b4177 | 1299 | 0,54 | -1,038837562 | 11,01229932 | 13,17170309 | 0,000284208 | 0,001491594 |
| b3299 | 117 | 0,48 | 1,059882189 | 7,793940715 | 13,16830532 | 0,000284724 | 0,001492554 |
| b1746 | 1479 | 0,58 | 1,76572748 | 7,693250741 | 13,16031751 | 0,00028594 | 0,001497179 |
| b2749 | 324 | 0,48 | 1,676931782 | 3,240170906 | 13,15450993 | 0,000286828 | 0,001500074 |
| b3474 | 1050 | 0,51 | -0,920544785 | 6,121977884 | 13,12069458 | 0,000292052 | 0,001525613 |
| b0641 | 582 | 0,52 | 0,723263626 | 7,666618955 | 13,10850192 | 0,000293959 | 0,001533787 |
| b2231 | 2628 | 0,54 | 0,988979933 | 9,357255585 | 13,09360019 | 0,000296306 | 0,001544238 |
| b3317 | 822 | 0,53 | 1,363038618 | 11,1427602 | 13,08147587 | 0,00029823 | 0,001552461 |
| b2476 | 714 | 0,52 | 1,455583344 | 8,458513998 | 13,06839446 | 0,00030032 | 0,001561527 |
| b2347 | 933 | 0,49 | -0,935135122 | 5,720460053 | 13,05984234 | 0,000301695 | 0,001566855 |
| b3492 | 1203 | 0,55 | -1,155240503 | 6,78764042 | 13,05348287 | 0,000302721 | 0,001570365 |
| b4157 | 315 | 0,5 | 2,065799025 | 1,855992845 | 13,03410433 | 0,000305869 | 0,001584137 |
| b1323 | 1542 | 0,52 | -0,835105086 | 8,072801171 | 13,03279885 | 0,000306083 | 0,001584137 |
| b2111 | 543 | 0,41 | -2,047738405 | 3,459130519 | 13,00710381 | 0,000310312 | 0,001604171 |
| b2307 | 717 | 0,5 | 1,581582365 | 3,94172949 | 13,00431739 | 0,000310774 | 0,001604709 |
| b2392 | 1239 | 0,53 | 1,113533734 | 5,802413553 | 12,98849603 | 0,000313411 | 0,001616463 |
| b4376 | 606 | 0,52 | -1,080098028 | 11,55308333 | 12,97328072 | 0,000315968 | 0,001627779 |
| b3700 | 1074 | 0,55 | 1,070430267 | 4,821979298 | 12,95565905 | 0,000318956 | 0,001641286 |
| b0622 | 561 | 0,47 | -1,542410089 | 4,339319822 | 12,94906064 | 0,000320082 | 0,001645192 |
| b0183 | 597 | 0,57 | 0,865041584 | 4,857097977 | 12,91156433 | 0,000326558 | 0,001676557 |
| b4494 | 1900 | 0,39 | 2,011273293 | 1,62154013 | 12,90444768 | 0,000327802 | 0,001681018 |
| b0715 | 1047 | 0,56 | -1,418356846 | 4,722718484 | 12,86929646 | 0,000334017 | 0,001709798 |
| b2530 | 1215 | 0,53 | 1,650433629 | 8,382458135 | 12,8683989 | 0,000334177 | 0,001709798 |
| b1223 | 1392 | 0,54 | -3,279996022 | 11,59984629 | 12,86412846 | 0,000334941 | 0,00171175 |
| b0731 | 1977 | 0,55 | 1,907842286 | 1,953367711 | 12,84761622 | 0,000337909 | 0,001724955 |
| b0198 | 654 | 0,52 | 1,113801358 | 4,994486176 | 12,8328711 | 0,000340583 | 0,001736623 |
| b1547 | 963 | 0,53 | 1,940872297 | 2,126908264 | 12,82886239 | 0,000341313 | 0,00173837 |
| b3416 | 2085 | 0,55 | -0,837097323 | 8,836818993 | 12,82090495 | 0,000342768 | 0,001743798 |
| b3315 | 333 | 0,5 | 1,304213157 | 10,02346205 | 12,81535404 | 0,000343786 | 0,001746996 |
| b3982 | 546 | 0,52 | 0,841847283 | 7,95770143 | 12,8086965 | 0,000345012 | 0,001751238 |
| b0408 | 1848 | 0,53 | 0,801257042 | 8,280280421 | 12,78196498 | 0,000349977 | 0,001774431 |
| b4488 | 1645 | 0,55 | 0,918503672 | 6,307192423 | 12,77851585 | 0,000350623 | 0,001775697 |
| b2210 | 1647 | 0,54 | 1,446574766 | 4,616495498 | 12,73133962 | 0,000359579 | 0,001818998 |
| b0123 | 1551 | 0,55 | -0,752018306 | 7,190540346 | 12,69927504 | 0,000365797 | 0,00184837 |
| b0170 | 852 | 0,5 | 1,221342989 | 10,0721586 | 12,69600242 | 0,000366438 | 0,001849522 |
| b2436 | 900 | 0,55 | 0,820768775 | 5,017811587 | 12,6860785 | 0,000368388 | 0,001857272 |
| b3434 | 594 | 0,5 | 1,105153836 | 3,793094629 | 12,65249691 | 0,000375064 | 0,001888808 |
| b0654 | 741 | 0,52 | 1,282789597 | 5,496853615 | 12,60118884 | 0,000385501 | 0,001939189 |
| b0856 | 954 | 0,51 | 1,764812816 | 3,329217944 | 12,58005526 | 0,000389885 | 0,001959042 |
| b1732 | 2262 | 0,52 | -0,971095102 | 10,30895004 | 12,57665619 | 0,000390595 | 0,00196041 |
| b3232 | 1128 | 0,52 | 0,902116683 | 5,228622997 | 12,56711879 | 0,000392593 | 0,001966334 |
| b3404 | 1353 | 0,57 | -0,604206384 | 7,238339611 | 12,56683716 | 0,000392653 | 0,001966334 |
| b2411 | 2016 | 0,54 | 0,673207161 | 7,403665479 | 12,55678631 | 0,00039477 | 0,001974731 |
| b4147 | 567 | 0,49 | 1,028542407 | 7,991899121 | 12,55032005 | 0,000396138 | 0,001979367 |
| b4292 | 954 | 0,59 | 1,641927103 | 2,491376169 | 12,53188756 | 0,000400065 | 0,001996763 |
| b3615 | 1035 | 0,46 | 1,616751126 | 2,168265926 | 12,5261394 | 0,000401298 | 0,002000688 |
| b1601 | 1035 | 0,5 | -1,09543767 | 4,764405411 | 12,51413109 | 0,000403886 | 0,00201135 |
| b3769 | 264 | 0,49 | 2,694376345 | 1,120451073 | 12,51095133 | 0,000404574 | 0,00201254 |
| b3564 | 1455 | 0,55 | -0,785594687 | 6,415012637 | 12,50806971 | 0,000405198 | 0,002013412 |
| b2764 | 1800 | 0,56 | 1,325482381 | 3,323772603 | 12,45497991 | 0,000416879 | 0,00206916 |
| b1068 | 924 | 0,55 | -0,894155311 | 7,22327815 | 12,45123639 | 0,000417715 | 0,002071017 |
| b1310 | 1293 | 0,5 | 1,707407803 | 1,785444408 | 12,44615117 | 0,000418854 | 0,002074369 |
| b2382 | 858 | 0,54 | -0,970814946 | 4,740438524 | 12,43599061 | 0,000421139 | 0,002083382 |
| b0927 | 648 | 0,52 | 0,910786842 | 6,924311696 | 12,37178814 | 0,00043587 | 0,00215388 |
| b1308 | 315 | 0,51 | -1,064675797 | 7,853107695 | 12,36475457 | 0,000437515 | 0,002159628 |
| b0619 | 1659 | 0,5 | -0,927548732 | 5,582351588 | 12,36149931 | 0,000438279 | 0,002161017 |
| b4490 | 830 | 0,54 | 2,081987611 | 2,722542714 | 12,35694805 | 0,000439348 | 0,002163911 |
| b4036 | 1341 | 0,52 | -1,92241078 | 10,94087993 | 12,3377091 | 0,000443899 | 0,002183927 |
| b3754 | 1428 | 0,49 | -1,314433888 | 5,45987742 | 12,32938825 | 0,000445883 | 0,002189137 |
| b0060 | 2352 | 0,55 | -0,700436872 | 6,678316295 | 12,3279894 | 0,000446217 | 0,002189137 |
| b1967 | 852 | 0,49 | -1,085729759 | 10,42949258 | 12,32712399 | 0,000446424 | 0,002189137 |
| b3530 | 3474 | 0,57 | -0,7302745 | 7,200495024 | 12,31904826 | 0,000448359 | 0,002196225 |
| b0527 | 522 | 0,53 | 1,571833152 | 2,19532896 | 12,30377881 | 0,000452042 | 0,002209879 |
| b0398 | 1203 | 0,53 | 0,950306135 | 4,901781496 | 12,29988737 | 0,000452986 | 0,002209879 |
| b1889 | 927 | 0,53 | 2,421584986 | 1,797769317 | 12,2993909 | 0,000453106 | 0,002209879 |
| b1780 | 885 | 0,5 | -1,155300887 | 7,319805011 | 12,29933852 | 0,000453119 | 0,002209879 |
| b0612 | 1464 | 0,51 | 1,692559321 | 2,557890852 | 12,29370478 | 0,000454489 | 0,002213134 |
| b3604 | 777 | 0,54 | 2,520399949 | 5,392404001 | 12,29253402 | 0,000454774 | 0,002213134 |
| b3417 | 2394 | 0,52 | -0,848428355 | 9,681284562 | 12,28567998 | 0,000456447 | 0,002217326 |
| b3539 | 1272 | 0,45 | 1,105138612 | 5,986377114 | 12,28315288 | 0,000457066 | 0,002217326 |
| b1187 | 720 | 0,51 | -0,708717197 | 6,649688376 | 12,28293311 | 0,000457119 | 0,002217326 |
| b4461 | 1287 | 0,51 | 0,701691208 | 5,69713292 | 12,27608747 | 0,000458799 | 0,002223068 |
| b0984 | 2097 | 0,52 | 1,181420332 | 3,696629412 | 12,26212295 | 0,000462245 | 0,002235027 |
| b3743 | 459 | 0,49 | 1,472219096 | 2,981330692 | 12,26038138 | 0,000462677 | 0,002235027 |
| b3182 | 1434 | 0,53 | 1,005924402 | 5,015642761 | 12,26003313 | 0,000462763 | 0,002235027 |
| b0166 | 825 | 0,53 | 0,983797077 | 8,230554769 | 12,25486764 | 0,000464046 | 0,00223881 |
| b3054 | 1302 | 0,55 | 0,617669114 | 7,067136152 | 12,23214436 | 0,000469732 | 0,002263804 |
| b3987 | 4029 | 0,53 | 1,098551552 | 11,96639742 | 12,22031062 | 0,000472721 | 0,002275761 |
| b3755 | 693 | 0,49 | -0,932343034 | 6,956529269 | 12,21815199 | 0,000473268 | 0,002275951 |
| b2798 | 756 | 0,53 | -0,735952417 | 5,085393588 | 12,20972891 | 0,000475409 | 0,0022838 |
| b2665 | 450 | 0,48 | -1,020619162 | 9,913628513 | 12,19884311 | 0,000478192 | 0,002294705 |
| b3936 | 213 | 0,49 | 0,96577379 | 8,627159094 | 12,19382497 | 0,00047948 | 0,002298426 |
| b1920 | 801 | 0,5 | -0,931835013 | 8,831591294 | 12,1533284 | 0,000490003 | 0,002346362 |

| | | | | | | | |
|---|---|---|---|---|---|---|---|
| b0678 | 801 | 0,49 | -0,642878154 | 7,709815506 | 12,13418048 | 0,00049506 | 0,002367194 |
| b0228 | 498 | 0,45 | -0,915069614 | 4,295181061 | 12,13286294 | 0,00049541 | 0,002367194 |
| b3316 | 279 | 0,49 | 1,259369513 | 9,200286188 | 12,12698188 | 0,000496975 | 0,002372142 |
| b4354 | 2151 | 0,56 | -1,003194918 | 5,610820673 | 12,11541824 | 0,000500066 | 0,002384358 |
| b4577 | 227 | 0,48 | -1,493799277 | 3,723957714 | 12,10683621 | 0,000502373 | 0,002392812 |
| b2490 | 414 | 0,53 | 1,713664899 | 1,880425401 | 12,08128568 | 0,000509305 | 0,002423252 |
| b0576 | 1377 | 0,53 | 0,872307133 | 5,12643269 | 12,07018201 | 0,000512347 | 0,002433324 |
| b0829 | 1872 | 0,54 | -0,59738655 | 7,066445482 | 12,06959873 | 0,000512507 | 0,002433324 |
| b2954 | 594 | 0,56 | 1,235734829 | 5,092899094 | 12,03837215 | 0,000521165 | 0,002469302 |
| b2952 | 567 | 0,51 | 0,819359798 | 5,465388677 | 12,03829229 | 0,000521187 | 0,002469302 |
| b1779 | 996 | 0,5 | -0,982112681 | 12,30147782 | 12,00966426 | 0,000529254 | 0,002504874 |
| b0800 | 963 | 0,56 | -0,885944297 | 8,113554845 | 11,99883414 | 0,000532338 | 0,002516815 |
| b3740 | 624 | 0,49 | 0,977809259 | 4,713638473 | 11,98237946 | 0,00053706 | 0,002536461 |
| b0119 | 363 | 0,52 | -1,045875801 | 7,542768255 | 11,97800062 | 0,000538323 | 0,002539752 |
| b1264 | 1563 | 0,54 | -5,44258606 | 9,341414367 | 11,9721442 | 0,000540018 | 0,002545067 |
| b3909 | 984 | 0,53 | -0,955197655 | 4,087624481 | 11,95362641 | 0,000545411 | 0,002565003 |
| b4214 | 741 | 0,55 | -0,991438638 | 8,174657876 | 11,95184795 | 0,000545932 | 0,002565003 |
| b1538 | 2046 | 0,5 | -0,850279788 | 8,269807944 | 11,9517372 | 0,000545965 | 0,002565003 |
| b4162 | 546 | 0,54 | -0,63854674 | 6,041213061 | 11,93973421 | 0,000549493 | 0,002578878 |
| b4367 | 789 | 0,53 | 2,961872145 | 4,220294968 | 11,92964684 | 0,000552476 | 0,002590167 |
| b4382 | 1323 | 0,55 | -0,877037768 | 8,625847282 | 11,92584206 | 0,000553606 | 0,00259275 |
| b1309 | 1680 | 0,44 | 1,586689136 | 1,845153153 | 11,91698421 | 0,000556245 | 0,002602389 |
| b3421 | 1227 | 0,51 | -1,045953917 | 5,2560189 | 11,9139386 | 0,000557155 | 0,002603929 |
| b2039 | 882 | 0,43 | 0,749170297 | 7,290912467 | 11,90366809 | 0,000560235 | 0,002615598 |
| b0593 | 1176 | 0,56 | 2,624383777 | 2,334972056 | 11,8962199 | 0,00056248 | 0,002623345 |
| b2199 | 738 | 0,55 | -1,31735329 | 5,39110054 | 11,85346343 | 0,000575542 | 0,002681476 |
| b3093 | 1419 | 0,54 | -0,770491897 | 5,888102213 | 11,78978921 | 0,000595565 | 0,002771881 |
| b2435 | 870 | 0,5 | 0,990792001 | 5,62072587 | 11,78006725 | 0,000598683 | 0,002783504 |
| b2998 | 288 | 0,45 | 2,118173409 | 1,534800938 | 11,77231533 | 0,000601182 | 0,002792224 |
| b0698 | 1674 | 0,55 | 2,642972146 | 4,971286493 | 11,73939432 | 0,000611909 | 0,002839107 |
| b0427 | 1365 | 0,53 | 0,961061588 | 5,04570731 | 11,73662415 | 0,000612821 | 0,002840396 |
| b0005 | 297 | 0,54 | 1,387238347 | 2,782404582 | 11,73230873 | 0,000614243 | 0,002844049 |
| b1116 | 1200 | 0,54 | 1,076216259 | 5,236419011 | 11,72109409 | 0,000617956 | 0,002858286 |
| b0655 | 909 | 0,49 | 0,910864364 | 8,935418507 | 11,7182374 | 0,000618905 | 0,002859726 |
| b3403 | 1623 | 0,54 | -1,158022341 | 11,76036563 | 11,71056892 | 0,000621461 | 0,002868578 |
| b2948 | 564 | 0,51 | 0,831776716 | 5,738804351 | 11,69658142 | 0,00062615 | 0,002887249 |
| b0850 | 288 | 0,5 | 0,916763169 | 4,17150171 | 11,67055462 | 0,000634971 | 0,002924912 |
| b1858 | 756 | 0,49 | -0,888714756 | 5,299556611 | 11,65397624 | 0,000640655 | 0,002948063 |
| b4483 | 783 | 0,54 | 1,018351293 | 3,980120608 | 11,63951865 | 0,000645653 | 0,002968018 |
| b0145 | 456 | 0,53 | 0,711238922 | 9,559413376 | 11,60781134 | 0,000656754 | 0,003015954 |
| b3105 | 897 | 0,55 | 0,962663246 | 5,37031077 | 11,5975534 | 0,000660386 | 0,003026712 |
| b0947 | 1110 | 0,5 | 0,816284569 | 5,670481816 | 11,59738211 | 0,000660447 | 0,003026712 |
| b3360 | 564 | 0,53 | -1,09440655 | 5,62558562 | 11,59170664 | 0,000662466 | 0,003032862 |
| b3448 | 441 | 0,51 | -1,206942198 | 7,89318194 | 11,57696301 | 0,000667739 | 0,003053884 |
| b2542 | 1203 | 0,54 | 1,175278344 | 4,134264275 | 11,57461635 | 0,000668582 | 0,003054624 |
| b1114 | 3447 | 0,55 | 0,794079359 | 7,901943713 | 11,56181844 | 0,0006732 | 0,003072588 |
| b3923 | 429 | 0,43 | -0,954636541 | 8,168879079 | 11,55091663 | 0,000677159 | 0,003087513 |
| b0679 | 1947 | 0,55 | -0,929819629 | 9,317017038 | 11,54628678 | 0,000678847 | 0,003092065 |
| b3308 | 540 | 0,5 | 1,208583679 | 10,82953774 | 11,51796374 | 0,000689269 | 0,003136347 |
| b2722 | 924 | 0,58 | 2,184840326 | 1,457705854 | 11,50266271 | 0,000694966 | 0,003159063 |
| b2812 | 807 | 0,55 | 0,889186734 | 5,263273165 | 11,49006712 | 0,000699691 | 0,003177321 |
| b3309 | 315 | 0,46 | 1,204645034 | 9,599309475 | 11,48210725 | 0,000702694 | 0,003187728 |
| b2482 | 2019 | 0,55 | 1,244422845 | 2,706946132 | 11,46136809 | 0,00071058 | 0,003220241 |
| b3310 | 372 | 0,51 | 1,109393303 | 10,2927366 | 11,45362362 | 0,000713547 | 0,003230423 |
| b0889 | 495 | 0,48 | 0,735369629 | 7,738794094 | 11,44719072 | 0,000716022 | 0,003238354 |
| b2840 | 693 | 0,51 | 0,946380486 | 4,904264336 | 11,42968217 | 0,0007228 | 0,003265718 |
| b0345 | 1083 | 0,56 | 1,112770827 | 5,496779667 | 11,42540764 | 0,000724465 | 0,003269943 |
| b1533 | 900 | 0,5 | 0,988097635 | 5,268910407 | 11,42251779 | 0,000725593 | 0,003271738 |
| b0517 | 1050 | 0,52 | 1,851152176 | 1,794045988 | 11,41677399 | 0,00072784 | 0,00327857 |
| b0781 | 990 | 0,54 | -1,229086836 | 8,244808785 | 11,41228825 | 0,000729599 | 0,003283196 |
| b1792 | 348 | 0,53 | -1,02040176 | 5,969075299 | 11,38195391 | 0,000741611 | 0,003333901 |
| b1824 | 144 | 0,42 | 1,092591091 | 9,208413032 | 11,37329683 | 0,000745075 | 0,003346119 |
| b3598 | 363 | 0,52 | -1,427012382 | 5,823335007 | 11,36704704 | 0,000747586 | 0,003354036 |
| b2081 | 1362 | 0,54 | 1,071273486 | 6,005795717 | 11,35846927 | 0,000751047 | 0,003366193 |
| b2472 | 1128 | 0,53 | -0,761683109 | 6,974730547 | 11,34288138 | 0,000757377 | 0,003391174 |
| b0023 | 264 | 0,47 | 1,698044778 | 7,366113661 | 11,33762108 | 0,000759526 | 0,003394032 |
| b3812 | 717 | 0,56 | 1,018045204 | 5,145909209 | 11,33760971 | 0,00075953 | 0,003394032 |
| b3927 | 846 | 0,51 | -0,641097266 | 5,96467183 | 11,32366984 | 0,000765253 | 0,0034162 |
| b1022 | 1326 | 0,47 | 1,714204592 | 3,024097329 | 11,31036842 | 0,000770755 | 0,003437336 |
| b2284 | 1338 | 0,56 | 1,118254625 | 10,21966362 | 11,29893148 | 0,000775517 | 0,003455137 |
| b2776 | 1479 | 0,49 | -1,158227668 | 4,039092833 | 11,26317242 | 0,0007906 | 0,003518836 |
| b3391 | 1239 | 0,54 | 1,314951491 | 3,113061501 | 11,25061361 | 0,000795967 | 0,00353921 |
| b1258 | 501 | 0,43 | -1,552475216 | 5,513731656 | 11,24867844 | 0,000796797 | 0,003539391 |
| b2700 | 498 | 0,57 | 1,10943142 | 5,123423398 | 11,2441766 | 0,000798732 | 0,003544473 |
| b0026 | 2817 | 0,56 | 0,811806877 | 10,0391352 | 11,23387585 | 0,000803177 | 0,003560674 |
| b1339 | 909 | 0,5 | 1,246521801 | 3,70210651 | 11,22654111 | 0,000806358 | 0,003571241 |
| b2303 | 363 | 0,45 | 0,734254246 | 6,46157306 | 11,17931885 | 0,000827141 | 0,003659672 |
| b3548 | 699 | 0,5 | -0,709094655 | 7,418770232 | 11,16020885 | 0,000835705 | 0,003693916 |
| b4393 | 327 | 0,54 | -1,033479768 | 5,850110681 | 11,15458426 | 0,000838243 | 0,003701482 |
| b0588 | 816 | 0,54 | 1,991309735 | 2,418295686 | 11,13725551 | 0,00084611 | 0,003732544 |
| b0311 | 1671 | 0,57 | 1,049817202 | 5,816664183 | 11,13451248 | 0,000847362 | 0,003734392 |
| b0880 | 225 | 0,48 | 0,952296582 | 10,19633214 | 11,13162167 | 0,000848684 | 0,003736542 |
| b0884 | 219 | 0,48 | 1,435168652 | 5,620822134 | 11,09112274 | 0,00086742 | 0,003815284 |
| b0532 | 2604 | 0,48 | 1,876826969 | 2,281426243 | 11,07102602 | 0,000876871 | 0,003853076 |
| b3102 | 987 | 0,51 | -0,806116051 | 7,788424808 | 11,06541839 | 0,000879527 | 0,003860962 |
| b2880 | 780 | 0,54 | 1,596748939 | 2,361713198 | 11,06314128 | 0,000880608 | 0,003861924 |
| b2468 | 1980 | 0,56 | -0,908496267 | 4,826167661 | 11,05432172 | 0,000884807 | 0,003876545 |
| b1534 | 1188 | 0,5 | 1,042286287 | 4,716187426 | 11,05145711 | 0,000886175 | 0,003878748 |
| b3540 | 1005 | 0,56 | 0,971616937 | 8,763879188 | 11,04877239 | 0,000887459 | 0,003880579 |
| b3210 | 2337 | 0,51 | -0,679704104 | 8,193723252 | 11,04458299 | 0,000889467 | 0,003884973 |
| b2993 | 495 | 0,55 | -1,286698536 | 6,091408535 | 11,04306042 | 0,000890198 | 0,003884973 |
| b4233 | 1374 | 0,55 | 0,613179495 | 7,491423365 | 11,03245802 | 0,000895304 | 0,003903454 |
| b2059 | 840 | 0,52 | 1,868191291 | 1,854533186 | 10,98952801 | 0,000916281 | 0,003991033 |
| b3361 | 603 | 0,53 | -1,11613418 | 8,48175002 | 10,97680076 | 0,000922596 | 0,004013073 |

| | | | | | | | |
|---|---|---|---|---|---|---|---|
| b0246 | 318 | 0,57 | 1,735687275 | 1,685593894 | 10,97572321 | 0,000923132 | 0,004013073 |
| b2622 | 1242 | 0,48 | 0,693202539 | 6,441139493 | 10,96863483 | 0,00092667 | 0,004024549 |
| b3225 | 894 | 0,52 | 0,998118508 | 5,425396098 | 10,96636514 | 0,000927806 | 0,004025581 |
| b2044 | 1221 | 0,55 | 1,307196458 | 2,645930508 | 10,96438938 | 0,000928795 | 0,004025978 |
| b0241 | 1056 | 0,45 | 1,664470981 | 2,368926174 | 10,95782868 | 0,00093209 | 0,004036354 |
| b4179 | 2442 | 0,55 | -0,826039545 | 9,756909368 | 10,93316383 | 0,000944581 | 0,004086496 |
| b2308 | 687 | 0,53 | 1,175694933 | 5,067326465 | 10,92952401 | 0,000946438 | 0,004090584 |
| b1504 | 531 | 0,45 | 2,964855313 | 0,727073523 | 10,91046737 | 0,000956224 | 0,004127915 |
| b1166 | 267 | 0,35 | 1,588348928 | 4,260817322 | 10,90912445 | 0,000956917 | 0,004127915 |
| b3516 | 825 | 0,41 | -0,814624044 | 9,675041832 | 10,88605735 | 0,000968909 | 0,004175623 |
| b2182 | 1191 | 0,53 | 0,755702369 | 5,808775786 | 10,8539768 | 0,000985838 | 0,004244454 |
| b2707 | 774 | 0,49 | -0,980922246 | 6,474192482 | 10,85221979 | 0,000986774 | 0,004244454 |
| b0029 | 951 | 0,55 | 0,662173431 | 6,838546525 | 10,82350447 | 0,001002196 | 0,004306657 |
| b3541 | 984 | 0,57 | 1,020602062 | 8,657416715 | 10,81897051 | 0,001004653 | 0,004312039 |
| b2681 | 1178 | 0,54 | 1,003267776 | 3,315500238 | 10,81764513 | 0,001005373 | 0,004312039 |
| b0484 | 2505 | 0,58 | -0,927871089 | 9,264836823 | 10,81474121 | 0,001006951 | 0,004314679 |
| b0688 | 1641 | 0,55 | 0,789437505 | 8,983737998 | 10,80238355 | 0,001013695 | 0,004339428 |
| b2708 | 966 | 0,57 | -1,006107117 | 6,319060405 | 10,79854478 | 0,001015799 | 0,004344286 |
| b1840 | 873 | 0,53 | 0,88693365 | 6,121310812 | 10,78699772 | 0,001022155 | 0,00436388 |
| b1263 | 1596 | 0,56 | -5,27863927 | 10,19397485 | 10,78668509 | 0,001022328 | 0,00436388 |
| b1750 | 711 | 0,48 | -1,157170266 | 3,882622609 | 10,7617207 | 0,00103621 | 0,004418928 |
| b3157 | 525 | 0,52 | -1,358963579 | 8,68837713 | 10,75049313 | 0,001042515 | 0,004441591 |
| b2800 | 648 | 0,49 | -0,986594211 | 4,445253735 | 10,73750548 | 0,001049858 | 0,004468625 |
| b4492 | 8622 | 0,5 | 0,6071357 | 6,990215883 | 10,72175259 | 0,001058833 | 0,004502553 |
| b4641 | 159 | 0,4 | 1,64236959 | 2,346096234 | 10,70611373 | 0,001067821 | 0,004533912 |
| b1839 | 342 | 0,46 | 0,883323443 | 5,23114792 | 10,70540335 | 0,001068231 | 0,004533912 |
| b1516 | 1023 | 0,5 | 1,732295535 | 7,308881906 | 10,69279564 | 0,001075536 | 0,004560596 |
| b0720 | 1284 | 0,51 | -0,979002908 | 11,99355945 | 10,68414254 | 0,001080578 | 0,004577647 |
| b2321 | 996 | 0,52 | 0,953900537 | 4,586467814 | 10,67491562 | 0,001085981 | 0,004596193 |
| b1365 | 264 | 0,48 | 2,871607172 | 0,890028206 | 10,67269323 | 0,001087287 | 0,004597377 |
| b1529 | 666 | 0,51 | -0,696929348 | 5,67183836 | 10,64622885 | 0,001102956 | 0,004659234 |
| b4027 | 639 | 0,55 | 2,526075513 | 1,000592966 | 10,62528626 | 0,001115517 | 0,00470786 |
| b4347 | 342 | 0,56 | 2,038803184 | 2,011983018 | 10,61700067 | 0,001120527 | 0,004724553 |
| b0078 | 492 | 0,5 | 1,273738205 | 3,188766237 | 10,61025422 | 0,001124622 | 0,004737366 |
| b2027 | 981 | 0,46 | 1,065279225 | 7,557554653 | 10,60013429 | 0,001130794 | 0,004758893 |
| b2053 | 1122 | 0,55 | 1,726259108 | 2,46714775 | 10,56713993 | 0,001151157 | 0,004835547 |
| b4694 | 339 | 0,5 | 1,30389134 | 2,597547241 | 10,56571949 | 0,001152042 | 0,004835547 |
| b1530 | 435 | 0,49 | 1,514294707 | 2,622576395 | 10,56539271 | 0,001152246 | 0,004835547 |
| b1818 | 801 | 0,52 | -0,923148414 | 10,37614273 | 10,56281575 | 0,001153853 | 0,004837763 |
| b3199 | 576 | 0,46 | 0,694680416 | 6,572124576 | 10,55718958 | 0,00115737 | 0,004845117 |
| b3590 | 1845 | 0,56 | -0,702990695 | 7,563813595 | 10,55655309 | 0,001157769 | 0,004845117 |
| b1705 | 192 | 0,51 | 1,152420503 | 2,727746111 | 10,48969994 | 0,001200419 | 0,005018915 |
| b0737 | 693 | 0,51 | 0,993830421 | 5,339658405 | 10,46475522 | 0,001216737 | 0,005082399 |
| b1865 | 453 | 0,53 | 0,643786241 | 6,326693043 | 10,41183554 | 0,001252102 | 0,005225254 |
| b0248 | 459 | 0,56 | 0,957819747 | 3,989238785 | 10,40604512 | 0,001256035 | 0,005236788 |
| b2465 | 2004 | 0,55 | -0,998763415 | 10,78373257 | 10,40290434 | 0,001258173 | 0,005240827 |
| b3607 | 822 | 0,54 | 0,693690604 | 7,064064071 | 10,39928647 | 0,00126064 | 0,00524623 |
| b0156 | 345 | 0,51 | 0,834140992 | 6,549939438 | 10,36964407 | 0,001281042 | 0,005326189 |
| b3571 | 2031 | 0,53 | -0,833983244 | 4,975170415 | 10,36185954 | 0,001286456 | 0,005343738 |
| b2660 | 1269 | 0,58 | 1,050028372 | 9,121869145 | 10,33671728 | 0,001304097 | 0,005409411 |
| b1588 | 2424 | 0,51 | -1,220911495 | 5,659509765 | 10,33589371 | 0,001304679 | 0,005409411 |
| b2190 | 3791 | 0,48 | -0,572131683 | 6,069227265 | 10,33091336 | 0,001308205 | 0,005419014 |
| b4316 | 726 | 0,44 | 1,728179036 | 3,574802302 | 10,32880175 | 0,001309702 | 0,005420207 |
| b4131 | 2148 | 0,48 | -1,788183885 | 5,771522838 | 10,32615364 | 0,001311583 | 0,005422982 |
| b0219 | 771 | 0,55 | -0,660033971 | 6,542612429 | 10,29428559 | 0,001334428 | 0,005512356 |
| b1804 | 1128 | 0,54 | 0,980740615 | 4,697757415 | 10,28852512 | 0,0013386 | 0,0055245 |
| b4311 | 717 | 0,43 | 1,498105629 | 2,124027352 | 10,261241 | 0,001358543 | 0,005601645 |
| b2976 | 2172 | 0,53 | -2,590730821 | 12,67856685 | 10,25786987 | 0,001361027 | 0,005606732 |
| b2462 | 336 | 0,51 | 1,823722999 | 1,801662573 | 10,23023429 | 0,001381571 | 0,005686136 |
| b3089 | 1245 | 0,55 | 0,74428985 | 6,807119574 | 10,22316295 | 0,001386878 | 0,005702741 |
| b2242 | 1260 | 0,56 | 1,645009865 | 2,24830739 | 10,20998059 | 0,001396826 | 0,005736872 |
| b3427 | 219 | 0,44 | 1,771413264 | 1,848390201 | 10,20877666 | 0,001397739 | 0,005736872 |
| b4126 | 231 | 0,49 | -0,904269862 | 6,497591858 | 10,18418942 | 0,0014165 | 0,005808556 |
| b2177 | 1815 | 0,51 | -0,719927817 | 6,203356994 | 10,17466561 | 0,001423835 | 0,005830997 |
| b0456 | 354 | 0,49 | 1,165941445 | 5,125799362 | 10,17215346 | 0,001425776 | 0,005830997 |
| b3088 | 966 | 0,54 | 1,627120576 | 3,061283991 | 10,17202572 | 0,001425875 | 0,005830997 |
| b1855 | 972 | 0,52 | 0,632251206 | 6,415228397 | 10,1644563 | 0,001431741 | 0,005849649 |
| b0998 | 600 | 0,53 | -1,217989946 | 4,850460353 | 10,16066927 | 0,001434685 | 0,005856338 |
| b2701 | 1086 | 0,55 | 0,988199318 | 5,575368009 | 10,15632077 | 0,001438073 | 0,005864827 |
| b0262 | 1047 | 0,56 | 1,432475002 | 2,410330091 | 10,15399667 | 0,001439887 | 0,005866887 |
| b1094 | 237 | 0,48 | 0,893378812 | 10,51639528 | 10,14900867 | 0,001443789 | 0,005877439 |
| b2344 | 1341 | 0,5 | 1,365650854 | 8,001658325 | 10,13368587 | 0,00145584 | 0,00592112 |
| b1817 | 972 | 0,5 | -0,911675859 | 10,50581922 | 10,12626412 | 0,001461714 | 0,00593962 |
| b4148 | 318 | 0,53 | -0,960339931 | 5,277586554 | 10,12150267 | 0,001465495 | 0,00594959 |
| b0683 | 447 | 0,48 | 0,873674766 | 7,640254299 | 10,11961039 | 0,001467 | 0,005950312 |
| b1423 | 1344 | 0,53 | -0,837147959 | 8,423513388 | 10,09927368 | 0,001483278 | 0,006008123 |
| b3519 | 1650 | 0,54 | -1,004901238 | 7,578434306 | 10,0984586 | 0,001483934 | 0,006008123 |
| b0752 | 942 | 0,53 | -0,759927016 | 5,83326841 | 10,08547137 | 0,001494429 | 0,006045155 |
| b2520 | 4962 | 0,53 | -0,789569989 | 9,485637294 | 10,06989352 | 0,001507117 | 0,006090983 |
| b0243 | 1254 | 0,54 | 0,66766391 | 7,979696177 | 10,03914337 | 0,001532485 | 0,0061773 |
| b0493 | 810 | 0,54 | 0,732258021 | 5,297841665 | 10,03904005 | 0,00153257 | 0,0061773 |
| b2699 | 1062 | 0,54 | 0,677200931 | 9,155679725 | 10,03899278 | 0,00153261 | 0,0061773 |
| b2200 | 663 | 0,56 | -1,347761838 | 4,487500007 | 9,996967269 | 0,001567982 | 0,006314193 |
| b4111 | 1503 | 0,5 | -0,737288202 | 9,145122512 | 9,983483701 | 0,001579506 | 0,006354887 |
| b2049 | 1437 | 0,55 | 1,438825759 | 2,350299628 | 9,966205931 | 0,001594397 | 0,006409047 |
| b3651 | 690 | 0,55 | 0,832779577 | 5,32777461 | 9,96375186 | 0,001596524 | 0,006411505 |
| b2373 | 1695 | 0,47 | 1,780910608 | 2,149080003 | 9,962200462 | 0,00159787 | 0,006411505 |
| b2889 | 549 | 0,5 | -0,860402605 | 5,940938675 | 9,943491933 | 0,001614191 | 0,0064712 |
| b4557 | 258 | 0,55 | 1,833151778 | 3,598548127 | 9,896202855 | 0,001656202 | 0,006633687 |
| b3290 | 1377 | 0,5 | 0,596737211 | 7,247211376 | 9,891017055 | 0,001660875 | 0,006646468 |
| b4326 | 393 | 0,49 | -1,686337827 | 3,714775396 | 9,886103204 | 0,001665317 | 0,006658295 |
| b4069 | 1959 | 0,57 | 1,64432993 | 10,06493691 | 9,835057344 | 0,001712169 | 0,006839522 |
| b2823 | 324 | 0,55 | 1,366531176 | 2,701084642 | 9,802925629 | 0,001742345 | 0,006952123 |
| b3859 | 987 | 0,49 | 0,899219929 | 5,971701188 | 9,801748944 | 0,00174346 | 0,006952123 |

| | | | | | | | |
|---|---|---|---|---|---|---|---|
| b3480 | 807 | 0,56 | -2,149189151 | 4,095061454 | 9,776210381 | 0,001767843 | 0,007043087 |
| b1186 | 1542 | 0,53 | 0,677585424 | 6,034800246 | 9,769515169 | 0,001774292 | 0,007062503 |
| b0859 | 1128 | 0,55 | 0,960652012 | 3,864539462 | 9,761836928 | 0,001781718 | 0,007084217 |
| b2208 | 495 | 0,55 | -1,929511667 | 7,870333985 | 9,760608674 | 0,001782909 | 0,007084217 |
| b2623 | 957 | 0,37 | 1,298845209 | 3,391799169 | 9,756935961 | 0,001786474 | 0,007092097 |
| b1435 | 1962 | 0,51 | 0,738630164 | 6,008565712 | 9,744241809 | 0,001798853 | 0,00713492 |
| b2527 | 516 | 0,52 | 1,498112382 | 4,938919928 | 9,714209049 | 0,001828488 | 0,007246049 |
| b3070 | 765 | 0,56 | 1,449104865 | 3,441090975 | 9,687875662 | 0,001854879 | 0,00734414 |
| b0462 | 3150 | 0,54 | 0,704443351 | 9,651379778 | 9,685265779 | 0,001857515 | 0,007348087 |
| b1479 | 1698 | 0,52 | 0,809716693 | 8,45824785 | 9,671906298 | 0,001871071 | 0,007395184 |
| b2771 | 1338 | 0,53 | 1,449061736 | 2,286572948 | 9,656116806 | 0,001887221 | 0,007452446 |
| b2154 | 837 | 0,54 | 0,680928343 | 6,561256968 | 9,651181448 | 0,001892298 | 0,007465917 |
| b0567 | 891 | 0,52 | 1,164877549 | 3,896621847 | 9,641102453 | 0,00190271 | 0,007500393 |
| b0913 | 2265 | 0,49 | -0,791959807 | 4,955615679 | 9,614781447 | 0,001930174 | 0,00760197 |
| b2139 | 1436 | 0,55 | 1,575183749 | 1,785110323 | 9,610592314 | 0,001934582 | 0,007610251 |
| b2304 | 894 | 0,55 | -0,66778631 | 8,021077313 | 9,609557251 | 0,001935673 | 0,007610251 |
| b3303 | 504 | 0,5 | 1,084296186 | 10,59264305 | 9,590958688 | 0,001955378 | 0,007680986 |
| b0348 | 945 | 0,58 | 1,68792449 | 1,956254056 | 9,585928699 | 0,001960742 | 0,007695313 |
| b4090 | 450 | 0,5 | 0,946915533 | 3,432137144 | 9,566230206 | 0,001981893 | 0,007771519 |
| b3574 | 849 | 0,52 | 0,843611813 | 4,995226473 | 9,536239173 | 0,002014541 | 0,007892634 |
| b0843 | 285 | 0,53 | 1,023899574 | 3,175987295 | 9,533333099 | 0,002017733 | 0,007898237 |
| b4052 | 1416 | 0,54 | 0,654839697 | 6,768055383 | 9,502216735 | 0,002052238 | 0,008026293 |
| b2243 | 1191 | 0,54 | 1,225021878 | 2,870087059 | 9,493566999 | 0,002061936 | 0,008057189 |
| b1749 | 807 | 0,53 | 0,97207329 | 5,767584829 | 9,487325878 | 0,002068962 | 0,008077601 |
| b4389 | 1383 | 0,58 | 0,648269395 | 6,72335276 | 9,478935123 | 0,002078446 | 0,008107566 |
| b2342 | 1311 | 0,56 | 1,073891424 | 6,072803881 | 9,460884259 | 0,002098999 | 0,008180619 |
| b2223 | 1323 | 0,53 | 1,897435665 | 2,033187289 | 9,454263182 | 0,002106589 | 0,008203069 |
| b0847 | 1686 | 0,52 | -0,617771444 | 6,108505591 | 9,449671977 | 0,002111869 | 0,008216489 |
| b1449 | 1038 | 0,52 | -0,622319832 | 8,086552855 | 9,439730239 | 0,002123347 | 0,008247039 |
| b0767 | 996 | 0,55 | -0,644874537 | 9,059259394 | 9,439683823 | 0,002123401 | 0,008247039 |
| b0261 | 933 | 0,6 | -0,713513633 | 5,89613621 | 9,431487665 | 0,002132912 | 0,008276806 |
| b1559 | 753 | 0,48 | 1,283385077 | 2,87338149 | 9,423806822 | 0,002141864 | 0,008304356 |
| b4384 | 720 | 0,52 | -0,710954777 | 9,902935653 | 9,419829282 | 0,002146515 | 0,008315195 |
| b1487 | 1551 | 0,5 | 1,579913071 | 5,481615835 | 9,406914497 | 0,002161687 | 0,008366736 |
| b4418 | 169 | 0,38 | 1,02965019 | 3,877885941 | 9,400799035 | 0,002168909 | 0,008387446 |
| b2000 | 3120 | 0,58 | -1,101562194 | 9,022315531 | 9,359425519 | 0,002218417 | 0,008571503 |
| b2824 | 408 | 0,53 | 1,183458863 | 2,735502609 | 9,328696134 | 0,002255928 | 0,008708932 |
| b0102 | 744 | 0,51 | 0,697270417 | 5,896725313 | 9,314205295 | 0,00227384 | 0,008770525 |
| b4192 | 1065 | 0,52 | -1,09924744 | 4,116750096 | 9,296668364 | 0,00229571 | 0,008847267 |
| b1384 | 906 | 0,49 | 1,457195992 | 4,538504594 | 9,278479156 | 0,002318619 | 0,008927877 |
| b4020 | 1632 | 0,55 | -0,563792437 | 6,844052419 | 9,267837933 | 0,002332128 | 0,008972188 |
| b0236 | 501 | 0,54 | 1,76566444 | 1,382432691 | 9,258769451 | 0,002343704 | 0,009008991 |
| b2539 | 519 | 0,52 | 1,477913632 | 2,646636905 | 9,2559131 | 0,002347363 | 0,009015321 |
| b3425 | 327 | 0,54 | 1,742980119 | 3,534392423 | 9,251747411 | 0,002352708 | 0,009028115 |
| b0330 | 1587 | 0,55 | 1,235456952 | 5,363069507 | 9,237484592 | 0,002371104 | 0,009090924 |
| b1262 | 1359 | 0,53 | -4,594717592 | 10,31569177 | 9,233911981 | 0,002375735 | 0,009100894 |
| b0064 | 879 | 0,53 | -0,777127615 | 8,006113419 | 9,231357341 | 0,002379052 | 0,009105817 |
| b0080 | 1005 | 0,55 | -0,674756416 | 8,141999629 | 9,226861302 | 0,002384901 | 0,009120416 |
| b2004 | 369 | 0,57 | 1,867740112 | 1,709601903 | 9,216900427 | 0,002397912 | 0,009162353 |
| b0524 | 723 | 0,54 | 0,972614839 | 4,600446488 | 9,207907571 | 0,00240972 | 0,009199628 |
| b0817 | 468 | 0,54 | 0,681943209 | 5,240086332 | 9,202788674 | 0,002416467 | 0,009211709 |
| b1638 | 657 | 0,52 | 0,854459229 | 5,708877762 | 9,202389172 | 0,002416995 | 0,009211709 |
| b1621 | 1593 | 0,53 | -0,880196572 | 5,879223058 | 9,195052566 | 0,002426701 | 0,009239712 |
| b4226 | 531 | 0,51 | 0,996531968 | 9,172735448 | 9,193722472 | 0,002428465 | 0,009239712 |
| b0908 | 1284 | 0,51 | 1,013114518 | 6,230310145 | 9,19043506 | 0,002432831 | 0,00924847 |
| b2457 | 294 | 0,58 | 1,122824201 | 3,711997002 | 9,187185882 | 0,002437153 | 0,009257051 |
| b2322 | 1179 | 0,57 | 1,433080041 | 2,958073446 | 9,18324807 | 0,002442402 | 0,009269133 |
| b1067 | 648 | 0,52 | -0,659419272 | 7,276516701 | 9,180238177 | 0,002446422 | 0,009276534 |
| b3302 | 180 | 0,5 | 0,94009057 | 8,879153763 | 9,162535766 | 0,0024702 | 0,009358781 |
| b0381 | 1095 | 0,51 | 0,712300123 | 7,414030957 | 9,149536873 | 0,00248781 | 0,009417538 |
| b3041 | 654 | 0,52 | 0,8424904 | 8,519529803 | 9,141489923 | 0,002498775 | 0,009451064 |
| b0528 | 213 | 0,55 | 1,149371345 | 3,730443759 | 9,132821703 | 0,002510642 | 0,009487941 |
| b4258 | 2856 | 0,55 | 0,736481217 | 9,798924112 | 9,114655694 | 0,002535697 | 0,009574552 |
| b1233 | 459 | 0,46 | 0,852861375 | 4,712704509 | 9,113088091 | 0,002537871 | 0,009574694 |
| b3811 | 897 | 0,54 | 0,838474016 | 6,303853936 | 9,102190139 | 0,002553036 | 0,009623808 |
| b4225 | 351 | 0,54 | -0,79152925 | 4,961522609 | 9,08779456 | 0,00257321 | 0,009691703 |
| b1654 | 348 | 0,53 | 0,770065772 | 7,203601781 | 9,084698801 | 0,002577569 | 0,009699971 |
| b1203 | 1092 | 0,51 | 0,583913073 | 7,642577795 | 9,063526844 | 0,002607585 | 0,009804696 |
| b0267 | 1155 | 0,62 | 1,966836549 | 1,530682806 | 9,061245641 | 0,002610841 | 0,009805649 |
| b3924 | 747 | 0,54 | -0,530195804 | 6,442081921 | 9,060283782 | 0,002612214 | 0,009805649 |
| b1272 | 1050 | 0,51 | 0,544643647 | 7,22997387 | 9,055283182 | 0,002619368 | 0,009824275 |
| b1360 | 747 | 0,53 | 1,364135018 | 2,602869484 | 9,044507797 | 0,002634852 | 0,009874084 |
| b4227 | 957 | 0,5 | -0,92166566 | 8,431784374 | 9,037268749 | 0,002645306 | 0,009904979 |
| b3906 | 849 | 0,5 | -0,759504392 | 4,034856432 | 9,023852257 | 0,002664792 | 0,009969614 |
| b1857 | 933 | 0,49 | -0,693334731 | 6,186525555 | 9,017573445 | 0,002673961 | 0,009995576 |
| b0963 | 459 | 0,53 | -0,602566101 | 7,902254078 | 9,015364419 | 0,002677195 | 0,009999324 |
| b3617 | 1197 | 0,54 | -0,772981066 | 10,19079119 | 9,01086589 | 0,002683792 | 0,010015618 |
| b2258 | 387 | 0,49 | -0,779973628 | 4,564427061 | 8,997123713 | 0,002704049 | 0,010082817 |
| b1295 | 246 | 0,48 | 1,50860606 | 2,794362667 | 8,989545017 | 0,002715286 | 0,010114082 |
| b4136 | 1698 | 0,54 | 0,720644321 | 6,048417595 | 8,988427755 | 0,002716947 | 0,010114082 |
| b2943 | 1395 | 0,53 | 0,89329049 | 6,286944279 | 8,981815188 | 0,002726796 | 0,010142325 |
| b2987 | 1500 | 0,52 | 1,461858305 | 1,923246617 | 8,972000526 | 0,002741483 | 0,010188495 |
| b3438 | 996 | 0,54 | -0,662193387 | 7,104755195 | 8,955867572 | 0,002765798 | 0,010270346 |
| b0155 | 1422 | 0,54 | -0,665535849 | 7,158035994 | 8,935912162 | 0,002796178 | 0,010374561 |
| b3789 | 882 | 0,55 | 0,603087594 | 6,260475975 | 8,929587456 | 0,002805877 | 0,010401937 |
| b1014 | 3963 | 0,57 | 1,637414534 | 11,70851826 | 8,917608261 | 0,002824342 | 0,010461734 |
| b3184 | 966 | 0,53 | 0,890034438 | 5,419491455 | 8,905979042 | 0,002842384 | 0,010519874 |
| b3552 | 660 | 0,58 | 1,141091643 | 3,404656638 | 8,900867704 | 0,002850352 | 0,010539837 |
| b2349 | 1158 | 0,48 | 0,865295113 | 4,109237553 | 8,898537304 | 0,002853992 | 0,010539837 |
| b2554 | 1335 | 0,55 | 0,818739159 | 5,344328726 | 8,897999172 | 0,002854833 | 0,010539837 |
| b3188 | 279 | 0,5 | 1,258365658 | 2,707332059 | 8,893534753 | 0,002861822 | 0,010556942 |
| b0027 | 495 | 0,54 | 0,611245921 | 6,673693959 | 8,882001603 | 0,002879956 | 0,010615102 |
| b4378 | 780 | 0,56 | 0,693513078 | 6,60496173 | 8,875002301 | 0,002891019 | 0,010647121 |
| b2399 | 393 | 0,46 | -0,799017994 | 5,471265461 | 8,872513361 | 0,002894963 | 0,010652893 |

| | | | | | | | |
|---|---|---|---|---|---|---|---|
| b1312 | 843 | 0,53 | 1,365047853 | 1,988432425 | 8,863778521 | 0,002908848 | 0,010689832 |
| b0093 | 831 | 0,53 | -0,70765622 | 8,424199311 | 8,863199402 | 0,002909771 | 0,010689832 |
| b4202 | 228 | 0,53 | 0,833301122 | 8,318769738 | 8,859119177 | 0,002916283 | 0,010704979 |
| b1593 | 696 | 0,52 | -1,702152275 | 8,246478847 | 8,855776899 | 0,002921627 | 0,010715822 |
| b2236 | 255 | 0,56 | 1,093346468 | 4,542478802 | 8,846909773 | 0,002935855 | 0,010759202 |
| b1683 | 1488 | 0,52 | -1,010975163 | 8,878311922 | 8,841917319 | 0,002943897 | 0,010779859 |
| b1632 | 696 | 0,53 | 1,063725937 | 4,269973396 | 8,835304764 | 0,002954583 | 0,010810156 |
| b0337 | 1284 | 0,53 | 0,93719163 | 6,572983288 | 8,82854433 | 0,002965548 | 0,010840556 |
| b1968 | 1359 | 0,4 | 0,921838269 | 4,310710362 | 8,827202845 | 0,002967729 | 0,010840556 |
| b1237 | 414 | 0,47 | 0,691798176 | 10,01916362 | 8,81999889 | 0,002979468 | 0,010874575 |
| b2393 | 1203 | 0,5 | 0,84327147 | 7,008578388 | 8,818177986 | 0,002982443 | 0,010876575 |
| b1841 | 375 | 0,5 | 0,76630753 | 5,453810876 | 8,803475016 | 0,003006573 | 0,01095566 |
| b3314 | 702 | 0,51 | 1,058526878 | 11,16555484 | 8,787436114 | 0,003033122 | 0,011043424 |
| b1883 | 1050 | 0,54 | 1,721943086 | 1,671096012 | 8,784668468 | 0,003037728 | 0,011051214 |
| b4465 | 1278 | 0,51 | 1,383900906 | 2,488675461 | 8,767039202 | 0,00306723 | 0,011149491 |
| b2685 | 1173 | 0,53 | 0,882627268 | 4,788354375 | 8,765198861 | 0,003070326 | 0,011151703 |
| b1204 | 585 | 0,5 | 1,009943951 | 4,529948782 | 8,758328348 | 0,003081914 | 0,011184728 |
| b1517 | 876 | 0,5 | 1,636868374 | 7,11135962 | 8,748397344 | 0,003098743 | 0,011236703 |
| b3940 | 2433 | 0,58 | 0,567562039 | 6,850410564 | 8,744284661 | 0,003105739 | 0,011252969 |
| b3817 | 381 | 0,35 | 2,01211587 | 1,63954647 | 8,73960689 | 0,003113716 | 0,01127276 |
| b2172 | 1467 | 0,52 | 0,674511004 | 4,662790645 | 8,732691519 | 0,003125548 | 0,011306461 |
| b2183 | 696 | 0,53 | 0,873696918 | 5,907454334 | 8,728620114 | 0,003132535 | 0,011322598 |
| b1735 | 843 | 0,42 | 0,851123333 | 3,779841518 | 8,719697739 | 0,003147902 | 0,011359441 |
| b4543 | 186 | 0,51 | 1,250981756 | 3,189642966 | 8,719086236 | 0,003148958 | 0,011359441 |
| b1086 | 960 | 0,51 | 0,735426384 | 5,618811289 | 8,7182914 | 0,003150331 | 0,011359441 |
| b0600 | 1161 | 0,54 | 1,591682323 | 4,02555104 | 8,71020377 | 0,003164338 | 0,011400775 |
| b4186 | 1164 | 0,53 | 1,369253419 | 2,49271216 | 8,700314332 | 0,003181552 | 0,011453587 |
| b2426 | 792 | 0,55 | -0,994315749 | 9,099874961 | 8,683219947 | 0,003211531 | 0,011552232 |
| b1646 | 522 | 0,53 | -0,964047087 | 7,848829133 | 8,679632519 | 0,003217859 | 0,011565712 |
| b1822 | 810 | 0,51 | 0,994934798 | 4,345480831 | 8,67253964 | 0,003230407 | 0,011601509 |
| b3393 | 441 | 0,55 | 1,785399592 | 1,274054697 | 8,670302525 | 0,003234375 | 0,01160646 |
| b1598 | 822 | 0,52 | -1,025106249 | 8,013374577 | 8,665919447 | 0,003242164 | 0,011625102 |
| b0842 | 1233 | 0,52 | -0,768450485 | 5,317478918 | 8,662433756 | 0,003248371 | 0,01163805 |
| b1845 | 2061 | 0,5 | -0,548840226 | 7,264226075 | 8,657161803 | 0,003257783 | 0,011662448 |
| b0430 | 615 | 0,53 | 1,159718126 | 9,526949745 | 8,654403786 | 0,003262718 | 0,011670793 |
| b1092 | 930 | 0,55 | 0,604008653 | 9,069889549 | 8,643887581 | 0,003281604 | 0,011720084 |
| b0834 | 1329 | 0,43 | 1,469861912 | 2,576828234 | 8,643818915 | 0,003281728 | 0,011720084 |
| b3600 | 1149 | 0,52 | -0,60675694 | 8,61974383 | 8,640683012 | 0,003287382 | 0,011730927 |
| b1238 | 618 | 0,42 | 1,299881335 | 4,328326585 | 8,622939381 | 0,003319557 | 0,011836322 |
| b2833 | 219 | 0,43 | -0,576750463 | 6,483725733 | 8,612636185 | 0,003338388 | 0,011894001 |
| b0169 | 726 | 0,51 | 0,988145775 | 10,60060907 | 8,600644301 | 0,003360441 | 0,011960402 |
| b3305 | 534 | 0,52 | 1,094991328 | 10,68127636 | 8,599603481 | 0,003362362 | 0,011960402 |
| b3440 | 1038 | 0,51 | -0,645274294 | 7,698921327 | 8,590234145 | 0,003379705 | 0,012012561 |
| b1180 | 660 | 0,52 | 0,66455371 | 6,157386423 | 8,588412134 | 0,003383089 | 0,012015058 |
| b1079 | 699 | 0,54 | 1,854928352 | 1,684278634 | 8,586145942 | 0,003387302 | 0,012020495 |
| b0488 | 459 | 0,57 | -0,929827189 | 6,391116905 | 8,583937093 | 0,003391413 | 0,012025564 |
| b3449 | 744 | 0,56 | 0,710485765 | 6,501222632 | 8,562892937 | 0,003430838 | 0,012155744 |
| b2724 | 612 | 0,57 | 2,005554901 | 1,066285174 | 8,556902067 | 0,003442147 | 0,012186178 |
| b1503 | 504 | 0,45 | 2,43883633 | 0,988106488 | 8,549193266 | 0,003456754 | 0,012228233 |
| b3860 | 627 | 0,48 | 0,728462502 | 7,846279785 | 8,547367258 | 0,003460223 | 0,012230852 |
| b3289 | 1290 | 0,5 | 0,648275617 | 7,003055045 | 8,537071859 | 0,00347985 | 0,012290534 |
| b2514 | 1275 | 0,55 | 0,60188812 | 8,442003239 | 8,52750814 | 0,003498183 | 0,012345557 |
| b4159 | 3324 | 0,56 | 0,56829526 | 6,51075454 | 8,523138704 | 0,003506592 | 0,012365496 |
| b3392 | 405 | 0,52 | 1,77128344 | 1,238520741 | 8,520548496 | 0,003511586 | 0,012373373 |
| b2478 | 879 | 0,52 | 0,594846066 | 8,631011742 | 8,509246535 | 0,003533463 | 0,012440678 |
| b3485 | 1125 | 0,55 | -0,708122455 | 6,809740836 | 8,504431054 | 0,003542827 | 0,012463853 |
| b4319 | 504 | 0,52 | 1,160500064 | 3,359862015 | 8,497003591 | 0,003557318 | 0,01250502 |
| b2471 | 357 | 0,47 | -0,780834816 | 5,272461149 | 8,486423126 | 0,003578066 | 0,012568096 |
| b1129 | 1461 | 0,51 | -0,539370844 | 7,032842431 | 8,467435106 | 0,003615609 | 0,012690023 |
| b3381 | 1164 | 0,55 | 1,349933584 | 2,027925193 | 8,456406545 | 0,003637598 | 0,012757211 |
| b3002 | 495 | 0,47 | 0,837553103 | 5,715489752 | 8,437821229 | 0,003674963 | 0,012878173 |
| b1686 | 411 | 0,52 | 0,7842313 | 5,984537563 | 8,434524845 | 0,003681631 | 0,01289146 |
| b4302 | 432 | 0,54 | -0,959933501 | 3,674238804 | 8,42253168 | 0,003705994 | 0,01296664 |
| b0937 | 576 | 0,54 | 1,576308535 | 2,261093362 | 8,417474615 | 0,003716316 | 0,012992613 |
| b2986 | 693 | 0,47 | 2,415812179 | 0,872108468 | 8,409414517 | 0,003732829 | 0,01304017 |
| b3304 | 354 | 0,54 | 0,928712294 | 9,799002724 | 8,389289347 | 0,003774384 | 0,013175071 |
| b2690 | 567 | 0,53 | -0,566187211 | 6,034400663 | 8,352965029 | 0,003850585 | 0,0134306 |
| b3632 | 1035 | 0,45 | 0,616754839 | 6,087045691 | 8,345789122 | 0,003865822 | 0,013473262 |
| b1428 | 981 | 0,48 | -0,855943876 | 6,822904694 | 8,34308635 | 0,003871577 | 0,013482836 |
| b0441 | 1872 | 0,52 | 0,687398137 | 8,572167031 | 8,341546209 | 0,003874861 | 0,013483793 |
| b4658 | 836 | 0,53 | -0,693317421 | 6,650702991 | 8,324891558 | 0,003910547 | 0,013597419 |
| b0163 | 387 | 0,44 | -0,576934611 | 8,293967567 | 8,321888518 | 0,003917017 | 0,013609358 |
| b1008 | 591 | 0,57 | 1,839849628 | 1,480471895 | 8,304426177 | 0,003954858 | 0,013730187 |
| b4315 | 540 | 0,49 | 0,979071543 | 4,967379356 | 8,301532235 | 0,003961164 | 0,013733228 |
| b4263 | 1503 | 0,54 | -0,537854462 | 7,84520171 | 8,301212656 | 0,003961862 | 0,013733228 |
| b1080 | 1098 | 0,54 | 1,758558043 | 1,877864238 | 8,286986292 | 0,00399302 | 0,013830539 |
| b1252 | 720 | 0,54 | 1,695394049 | 4,462865279 | 8,279861375 | 0,004008719 | 0,013874192 |
| b1762 | 1041 | 0,41 | 1,479430107 | 2,108372319 | 8,275104475 | 0,004019235 | 0,013899854 |
| b0101 | 198 | 0,52 | 0,847754785 | 4,274818848 | 8,269399914 | 0,004031883 | 0,013932845 |
| b2659 | 978 | 0,54 | 0,997025346 | 9,469444469 | 8,263891725 | 0,004044134 | 0,013964414 |
| b0407 | 333 | 0,5 | 0,66293701 | 7,530783333 | 8,250996495 | 0,004072964 | 0,014053135 |
| b1009 | 801 | 0,58 | 2,10544685 | 1,557179477 | 8,247595028 | 0,004080603 | 0,014068663 |
| b2047 | 1395 | 0,55 | 1,438105641 | 1,983862279 | 8,243736409 | 0,004089287 | 0,014087765 |
| b1819 | 852 | 0,53 | -0,811535393 | 10,5550099 | 8,226020994 | 0,004129397 | 0,01421502 |
| b3190 | 255 | 0,51 | 1,428877093 | 4,429129768 | 8,223902568 | 0,00413422 | 0,014220701 |
| b1620 | 1029 | 0,54 | -0,73437102 | 4,991119039 | 8,216963749 | 0,004150058 | 0,014255607 |
| b0779 | 2022 | 0,54 | 0,542899427 | 8,344130346 | 8,216616723 | 0,004150852 | 0,014255607 |
| b3998 | 672 | 0,59 | 0,617174562 | 6,11976977 | 8,215280423 | 0,00415391 | 0,014255607 |
| b1759 | 408 | 0,55 | 1,194361487 | 3,031260017 | 8,19817912 | 0,004193245 | 0,014375914 |
| b0685 | 294 | 0,54 | 1,359766108 | 2,479702509 | 8,19725552 | 0,00419538 | 0,014375914 |
| b3014 | 258 | 0,48 | 2,104998423 | 1,719420409 | 8,190568425 | 0,004210873 | 0,014417977 |
| b2561 | 849 | 0,52 | 0,889139797 | 3,990811129 | 8,175736444 | 0,004245443 | 0,014525248 |
| b1661 | 1149 | 0,48 | 0,755525939 | 9,783024107 | 8,159351688 | 0,004283968 | 0,014645878 |
| b0680 | 1665 | 0,52 | 0,756524195 | 8,323173855 | 8,146025946 | 0,004315563 | 0,014742648 |

| | | | | | | | |
|---|---|---|---|---|---|---|---|
| b3103 | 366 | 0,52 | -0,651098513 | 7,285691506 | 8,143546818 | 0,004321467 | 0,014751574 |
| b0231 | 1056 | 0,54 | -0,734550406 | 5,770721523 | 8,121624557 | 0,004374035 | 0,014919652 |
| b4097 | 759 | 0,61 | 1,727981769 | 1,533088968 | 8,114515699 | 0,00439122 | 0,014966882 |
| b0791 | 411 | 0,55 | -0,790528813 | 7,627486334 | 8,107505736 | 0,004408234 | 0,015013454 |
| b3715 | 666 | 0,48 | 0,755859703 | 4,111267445 | 8,099796274 | 0,004427023 | 0,015065998 |
| b3164 | 2136 | 0,54 | 0,664554779 | 10,48199194 | 8,087336681 | 0,004457562 | 0,015149635 |
| b2188 | 1761 | 0,52 | 0,506302873 | 6,75113549 | 8,087012338 | 0,00445836 | 0,015149635 |
| b3639 | 1221 | 0,54 | 0,647915137 | 6,613154753 | 8,076190102 | 0,004485064 | 0,015228832 |
| b0401 | 1320 | 0,56 | -0,692562954 | 7,391284808 | 8,065103685 | 0,004512589 | 0,015310693 |
| b1460 | 1137 | 0,42 | 2,368603923 | 1,081872346 | 8,049450801 | 0,004551745 | 0,015431862 |
| b3823 | 621 | 0,52 | 0,901593095 | 5,181405114 | 8,043333815 | 0,004567141 | 0,015472354 |
| b3032 | 828 | 0,57 | -0,675887954 | 8,436568519 | 8,029108647 | 0,004603149 | 0,015582562 |
| b1243 | 1632 | 0,48 | -0,79378396 | 12,54392148 | 8,020795823 | 0,004624325 | 0,015634321 |
| b4176 | 198 | 0,49 | 1,672025358 | 1,692697382 | 8,020368804 | 0,004625415 | 0,015634321 |
| b2136 | 579 | 0,54 | -0,622823722 | 5,692436215 | 8,002015549 | 0,004672531 | 0,015781676 |
| b1693 | 759 | 0,5 | 0,799496506 | 4,943779098 | 7,996969213 | 0,004685571 | 0,015813803 |
| b0605 | 564 | 0,5 | -0,737740872 | 11,58779597 | 7,994191151 | 0,004692766 | 0,015825486 |
| b3379 | 879 | 0,53 | 1,196418343 | 2,473157835 | 7,992907349 | 0,004696095 | 0,015825486 |
| b1812 | 1362 | 0,51 | 0,653334443 | 5,311521023 | 7,98640838 | 0,004712982 | 0,015870462 |
| b1597 | 309 | 0,51 | -1,359520366 | 3,309168262 | 7,979469171 | 0,004731081 | 0,015919449 |
| b4397 | 474 | 0,49 | 0,755248059 | 5,271957831 | 7,9766032 | 0,004738577 | 0,01593271 |
| b3706 | 1365 | 0,56 | 0,568424342 | 6,725191348 | 7,970162926 | 0,004755466 | 0,01597751 |
| b2064 | 1854 | 0,53 | -0,462356574 | 7,28243993 | 7,961445595 | 0,004778423 | 0,016042617 |
| b2103 | 801 | 0,55 | 1,112455327 | 3,202965485 | 7,942706053 | 0,004828158 | 0,016197459 |
| b0347 | 1665 | 0,57 | 1,189007574 | 2,392932354 | 7,940972349 | 0,004832786 | 0,016200858 |
| b3031 | 582 | 0,54 | -0,544931713 | 6,228468805 | 7,9326547 | 0,004855051 | 0,016263334 |
| b0367 | 828 | 0,57 | 1,595976284 | 1,574892643 | 7,930570828 | 0,004860646 | 0,016269914 |
| b1870 | 744 | 0,51 | 0,776841536 | 5,656440251 | 7,917949119 | 0,004894673 | 0,016361583 |
| b1882 | 390 | 0,49 | 1,19558994 | 2,694844341 | 7,9175628 | 0,004895718 | 0,016361583 |
| b2977 | 405 | 0,55 | -2,375966545 | 9,850468809 | 7,916356374 | 0,004898984 | 0,016361583 |
| b1807 | 696 | 0,56 | 1,020276181 | 4,858444709 | 7,883153629 | 0,004989739 | 0,01665228 |
| b0285 | 957 | 0,61 | 1,086855794 | 4,212872631 | 7,878121625 | 0,005003642 | 0,016686254 |
| b2377 | 243 | 0,48 | -1,060132904 | 5,792452512 | 7,873091948 | 0,005017579 | 0,016720288 |
| b1990 | 933 | 0,5 | -0,831336313 | 7,913822355 | 7,870578014 | 0,005024559 | 0,016731109 |
| b2028 | 1167 | 0,44 | 0,79301068 | 4,270596614 | 7,865169323 | 0,005039611 | 0,016768771 |
| b4333 | 861 | 0,43 | -1,135143296 | 3,297320706 | 7,845335246 | 0,005095201 | 0,016941165 |
| b2213 | 1065 | 0,57 | -0,868207574 | 6,418521943 | 7,842366666 | 0,005103575 | 0,016956428 |
| b2166 | 942 | 0,47 | 1,087868699 | 2,56287899 | 7,83419347 | 0,005126702 | 0,017020652 |
| b2714 | 1011 | 0,53 | 0,784806278 | 5,146699991 | 7,816353928 | 0,005177554 | 0,017176757 |
| b0466 | 162 | 0,48 | 1,27055207 | 2,796995893 | 7,81288275 | 0,005187509 | 0,017197052 |
| b1273 | 252 | 0,47 | 0,574085905 | 6,543732922 | 7,809095949 | 0,00519839 | 0,017220389 |
| b2175 | 567 | 0,47 | 1,157394608 | 8,44194978 | 7,801575776 | 0,005220069 | 0,017279431 |
| b2486 | 1581 | 0,54 | 0,910994956 | 3,149101436 | 7,782839689 | 0,005274482 | 0,017446664 |
| b0058 | 660 | 0,54 | 0,703878651 | 5,533955686 | 7,776180784 | 0,00529396 | 0,017498178 |
| b1071 | 294 | 0,53 | 1,383865011 | 2,095633666 | 7,751258075 | 0,005367514 | 0,017726333 |
| b1388 | 930 | 0,52 | 1,753410726 | 4,583842607 | 7,750120049 | 0,005370897 | 0,017726333 |
| b4056 | 417 | 0,52 | -0,776284711 | 8,185492591 | 7,740023069 | 0,005401011 | 0,017812606 |
| b1880 | 1149 | 0,55 | 1,75205599 | 1,581180234 | 7,738476959 | 0,005405638 | 0,017814755 |
| b2073 | 660 | 0,51 | 1,487316188 | 1,920630518 | 7,733970509 | 0,005419145 | 0,017846149 |
| b1248 | 330 | 0,47 | 0,686020256 | 5,489378419 | 7,730776299 | 0,00542874 | 0,017864621 |
| b0911 | 1674 | 0,51 | 1,481482067 | 11,97811223 | 7,717612321 | 0,005468467 | 0,01798215 |
| b0365 | 963 | 0,56 | 2,262582623 | 1,42026865 | 7,714696801 | 0,005477306 | 0,017998009 |
| b0068 | 984 | 0,53 | 0,96325679 | 4,03756849 | 7,708876289 | 0,005494995 | 0,018042906 |
| b0349 | 867 | 0,55 | 1,082640776 | 2,616132524 | 7,706722772 | 0,005501554 | 0,018051219 |
| b3300 | 1332 | 0,5 | 0,868325518 | 12,0874849 | 7,703217523 | 0,005512248 | 0,018073076 |
| b2711 | 1134 | 0,54 | 1,189059305 | 2,788219792 | 7,68937246 | 0,005554694 | 0,018198931 |
| b0965 | 414 | 0,54 | -0,714193241 | 7,622509113 | 7,666672381 | 0,00562501 | 0,018404406 |
| b3819 | 891 | 0,51 | 1,485618735 | 3,387839676 | 7,666476112 | 0,005625622 | 0,018404406 |
| b0662 | 1176 | 0,57 | -0,902611464 | 8,442787558 | 7,662911224 | 0,005636748 | 0,018427355 |
| b4042 | 369 | 0,52 | -0,600935302 | 5,302154695 | 7,660545376 | 0,005644144 | 0,018438086 |
| b0433 | 1476 | 0,54 | 0,919582811 | 4,776867113 | 7,645783506 | 0,005690517 | 0,018576037 |
| b0131 | 381 | 0,52 | 0,766547569 | 6,482848381 | 7,637380923 | 0,005717087 | 0,018641363 |
| b2445 | 369 | 0,47 | 1,771402945 | 1,484917059 | 7,636825563 | 0,005718847 | 0,018641363 |
| b1069 | 1536 | 0,54 | 0,667202527 | 4,883670651 | 7,619832723 | 0,005772984 | 0,018804154 |
| b2060 | 2163 | 0,53 | 1,299694537 | 2,38133212 | 7,615496398 | 0,005786883 | 0,018835736 |
| b0629 | 954 | 0,45 | 0,926510084 | 3,512362159 | 7,60880036 | 0,005808411 | 0,018883583 |
| b4356 | 1362 | 0,52 | -0,701823947 | 4,673846526 | 7,608304443 | 0,005810009 | 0,018883583 |
| b3558 | 852 | 0,43 | 1,673720014 | 1,826447386 | 7,594499657 | 0,005854664 | 0,019014931 |
| b1280 | 1170 | 0,52 | -0,539719757 | 6,528172017 | 7,588802851 | 0,005873194 | 0,019061299 |
| b3231 | 429 | 0,51 | 0,952779764 | 9,583296903 | 7,58013257 | 0,00590151 | 0,019130183 |
| b3510 | 333 | 0,43 | -1,041934202 | 12,37645367 | 7,578915251 | 0,005905497 | 0,019130183 |
| b2603 | 519 | 0,46 | -0,734735491 | 4,478679993 | 7,578388469 | 0,005907223 | 0,019130183 |
| b3509 | 327 | 0,4 | -1,102272177 | 11,36556386 | 7,569598368 | 0,005936101 | 0,019209823 |
| b3057 | 822 | 0,52 | 0,627976717 | 5,266040314 | 7,568175221 | 0,00594079 | 0,019211126 |
| b3646 | 618 | 0,53 | 1,002874747 | 3,982794913 | 7,531715759 | 0,006062215 | 0,019589651 |
| b3967 | 858 | 0,52 | -0,543883424 | 6,378925348 | 7,529548278 | 0,006069512 | 0,019599103 |
| b1851 | 1812 | 0,54 | -0,524777471 | 6,385398075 | 7,526973257 | 0,006078194 | 0,019613006 |
| b4098 | 846 | 0,58 | 2,107779411 | 1,326559188 | 7,507304484 | 0,006144927 | 0,019809848 |
| b4485 | 1503 | 0,56 | -0,915979783 | 6,80895906 | 7,506393221 | 0,006148036 | 0,019809848 |
| b1878 | 393 | 0,53 | 2,056726414 | 1,22436253 | 7,48758878 | 0,006212569 | 0,020003401 |
| b0587 | 1134 | 0,45 | 2,279036137 | 1,04891031 | 7,48333084 | 0,006227277 | 0,020036364 |
| b1519 | 759 | 0,54 | -0,536543144 | 7,129399963 | 7,481172726 | 0,006234745 | 0,020046002 |
| b0520 | 816 | 0,57 | 1,413616094 | 2,0208475 | 7,46453656 | 0,006292623 | 0,020217588 |
| b3488 | 1623 | 0,37 | -0,997263062 | 3,560698816 | 7,455445137 | 0,006324484 | 0,020305398 |
| b3493 | 1500 | 0,54 | 0,535800939 | 7,889609972 | 7,447923046 | 0,006350969 | 0,020375837 |
| b2484 | 1440 | 0,55 | 1,435682372 | 2,133732962 | 7,432427016 | 0,006405889 | 0,020537334 |
| b0677 | 1149 | 0,52 | -0,528505339 | 7,735950437 | 7,420852408 | 0,006447227 | 0,020655089 |
| b3307 | 306 | 0,53 | 0,970393598 | 10,12395859 | 7,399273631 | 0,006525022 | 0,020889392 |
| b2142 | 696 | 0,53 | -0,910752368 | 5,204524958 | 7,396816155 | 0,006533942 | 0,020903018 |
| b1051 | 375 | 0,5 | -0,855885833 | 9,917815497 | 7,395007555 | 0,006540515 | 0,020909121 |
| b3067 | 1842 | 0,53 | 0,69122984 | 9,697933313 | 7,392761737 | 0,006548686 | 0,020920322 |
| b4395 | 648 | 0,56 | 0,514966804 | 5,715338932 | 7,389733262 | 0,006559722 | 0,02094065 |
| b3230 | 393 | 0,52 | 0,917873609 | 9,732217512 | 7,38764559 | 0,00656734 | 0,020950048 |
| b2128 | 732 | 0,58 | 0,803179615 | 4,960160541 | 7,376512122 | 0,00660812 | 0,021065146 |

| | | | | | | | |
|---|---|---|---|---|---|---|---|
| b1307 | 222 | 0,55 | 0,976590536 | 3,184369866 | 7,369481651 | 0,006634005 | 0,021132631 |
| b1091 | 954 | 0,55 | 0,584543763 | 8,360645834 | 7,367444097 | 0,006641527 | 0,0211383 |
| b0097 | 513 | 0,54 | -0,575052574 | 6,492931654 | 7,366445076 | 0,006645217 | 0,0211383 |
| b2963 | 1080 | 0,51 | 0,749847039 | 4,909937386 | 7,363442669 | 0,006656322 | 0,021158608 |
| b0987 | 306 | 0,58 | 1,450671452 | 2,196718738 | 7,359289693 | 0,006671714 | 0,021192504 |
| b0708 | 1419 | 0,53 | -0,550965679 | 6,513023183 | 7,353376552 | 0,006693692 | 0,021247259 |
| b2372 | 945 | 0,45 | 1,74077866 | 1,336914019 | 7,347560964 | 0,00671538 | 0,021301014 |
| b4561 | 267 | 0,46 | 1,675304498 | 1,878583746 | 7,340506343 | 0,006741785 | 0,021369646 |
| b1645 | 2013 | 0,53 | -0,73663381 | 5,874793833 | 7,337297108 | 0,006753832 | 0,021379294 |
| b4303 | 807 | 0,53 | -0,868815047 | 4,839723332 | 7,337154178 | 0,006754369 | 0,021379294 |
| b0590 | 1005 | 0,61 | 1,580756083 | 2,132689783 | 7,331949883 | 0,006773953 | 0,021426152 |
| b3866 | 510 | 0,52 | -0,562638027 | 7,39692116 | 7,330608383 | 0,00677901 | 0,021427027 |
| b2113 | 1110 | 0,54 | 0,518068259 | 8,478562461 | 7,322656071 | 0,006809071 | 0,021492007 |
| b0059 | 2907 | 0,55 | 0,613961449 | 7,714487508 | 7,322632808 | 0,006809159 | 0,021492007 |
| b3180 | 294 | 0,49 | 0,656174126 | 6,72900523 | 7,310684733 | 0,00685458 | 0,021620147 |
| b1866 | 1773 | 0,54 | 0,609551689 | 9,084374924 | 7,288117545 | 0,006941217 | 0,021878013 |
| b2950 | 981 | 0,55 | 0,991072191 | 3,206635983 | 7,28517468 | 0,006952597 | 0,021897472 |
| b3996 | 774 | 0,53 | 0,733327707 | 6,124690449 | 7,283995552 | 0,006957162 | 0,021897472 |
| b3858 | 270 | 0,46 | -0,681395474 | 6,371870196 | 7,265609987 | 0,007028739 | 0,022107235 |
| b2407 | 834 | 0,52 | 1,559633422 | 1,795707879 | 7,259768114 | 0,00705164 | 0,022163709 |
| b3523 | 1323 | 0,56 | 0,74994786 | 3,954718745 | 7,258505138 | 0,0070566 | 0,022163758 |
| b4573 | 897 | 0,55 | 1,047348032 | 2,705172838 | 7,246857254 | 0,007102521 | 0,022292367 |
| b0968 | 279 | 0,52 | -0,740433225 | 5,902449161 | 7,245560194 | 0,007107654 | 0,022292865 |
| b0489 | 918 | 0,53 | -1,059232361 | 7,767758238 | 7,229077401 | 0,007173206 | 0,022478439 |
| b3652 | 2082 | 0,57 | 0,547255466 | 6,58060379 | 7,228165437 | 0,007176851 | 0,022478439 |
| b2414 | 972 | 0,5 | 0,721857572 | 8,277459147 | 7,218417024 | 0,007215932 | 0,022563061 |
| b3586 | 1137 | 0,51 | 1,108165425 | 2,913918411 | 7,218173118 | 0,007216912 | 0,022563061 |
| b0735 | 294 | 0,5 | -0,75011712 | 6,402334328 | 7,217660991 | 0,007218971 | 0,022563061 |
| b2531 | 489 | 0,54 | 1,296240165 | 5,9282479 | 7,213558805 | 0,007235489 | 0,022581067 |
| b2259 | 267 | 0,47 | -0,825154876 | 5,439085079 | 7,212523554 | 0,007239663 | 0,022581067 |
| b4087 | 1533 | 0,48 | 0,978899681 | 2,566439659 | 7,212138529 | 0,007241216 | 0,022581067 |
| b1216 | 1101 | 0,49 | 0,77274286 | 4,782879166 | 7,21122943 | 0,007244885 | 0,022581067 |
| b3916 | 963 | 0,53 | -0,637666414 | 9,600136265 | 7,209506374 | 0,007251844 | 0,022587049 |
| b1801 | 1446 | 0,51 | 1,003274789 | 2,732733156 | 7,20586905 | 0,007266555 | 0,022617154 |
| b0635 | 1902 | 0,53 | 0,734883064 | 5,418346575 | 7,195395521 | 0,007309088 | 0,022733749 |
| b1011 | 693 | 0,55 | 1,555649979 | 1,718557742 | 7,19342479 | 0,007317119 | 0,022741283 |
| b0321 | 1419 | 0,58 | -0,708871023 | 4,021622086 | 7,191434421 | 0,00732524 | 0,022741283 |
| b1962 | 696 | 0,54 | 0,835359368 | 4,83997915 | 7,191068974 | 0,007326732 | 0,022741283 |
| b3606 | 474 | 0,55 | 0,833839586 | 3,429078589 | 7,18801151 | 0,007339227 | 0,02275223 |
| b0288 | 330 | 0,5 | 1,342180251 | 2,332072299 | 7,187721893 | 0,007340412 | 0,02275223 |
| b0631 | 264 | 0,46 | 1,037160993 | 6,194896627 | 7,172443396 | 0,007403187 | 0,022930951 |
| b1875 | 567 | 0,51 | 0,804972904 | 4,724098777 | 7,165119608 | 0,007433473 | 0,023008859 |
| b0613 | 879 | 0,55 | 1,594589625 | 1,747391671 | 7,159960414 | 0,007454884 | 0,023051026 |
| b0585 | 1125 | 0,55 | 2,01894582 | 1,637938414 | 7,159359436 | 0,007457382 | 0,023051026 |
| b1828 | 1374 | 0,52 | 1,425160933 | 2,614338869 | 7,155785321 | 0,007472257 | 0,023081086 |
| b1756 | 654 | 0,51 | 1,158077976 | 3,341311833 | 7,148919823 | 0,007500915 | 0,023153651 |
| b1163 | 1212 | 0,44 | 0,851077931 | 3,905093017 | 7,139619843 | 0,007539914 | 0,023258015 |
| b1953 | 228 | 0,49 | -0,905350022 | 7,367038812 | 7,132630985 | 0,007569358 | 0,023327372 |
| b3034 | 630 | 0,51 | 0,705117506 | 5,76630787 | 7,1318139 | 0,007572808 | 0,023327372 |
| b1939 | 996 | 0,56 | 1,285378795 | 2,254057646 | 7,115307076 | 0,007642851 | 0,023526963 |
| b1477 | 285 | 0,51 | 0,857643579 | 4,011821193 | 7,110357564 | 0,007663982 | 0,023575819 |
| b2583 | 699 | 0,52 | 0,931879384 | 3,198765305 | 7,107152279 | 0,007677698 | 0,023601813 |
| b1232 | 843 | 0,47 | 0,690370057 | 6,295219646 | 7,092042411 | 0,007742696 | 0,023785307 |
| b3469 | 2199 | 0,58 | -0,586152319 | 7,424838993 | 7,087896137 | 0,00776063 | 0,023824071 |
| b3468 | 627 | 0,57 | -0,621037559 | 6,301324552 | 7,084100457 | 0,007777085 | 0,023858244 |
| b3340 | 2115 | 0,51 | 0,886124259 | 13,06153466 | 7,081310842 | 0,007789201 | 0,023866069 |
| b3750 | 966 | 0,53 | 0,596907681 | 7,140652679 | 7,081061429 | 0,007790285 | 0,023866069 |
| b2698 | 501 | 0,52 | 1,328085316 | 2,75524911 | 7,06045659 | 0,007880391 | 0,024125623 |
| b2688 | 1557 | 0,51 | 0,563909749 | 8,86109326 | 7,057556581 | 0,007893158 | 0,024148215 |
| b2636 | 444 | 0,51 | 1,770014453 | 1,312416138 | 7,045561587 | 0,007946191 | 0,024293879 |
| b2324 | 2007 | 0,53 | 0,545135023 | 5,915788659 | 7,036297676 | 0,007987398 | 0,024403215 |
| b1175 | 813 | 0,5 | 0,639438619 | 8,607226416 | 7,034572766 | 0,007995094 | 0,024410091 |
| b3313 | 411 | 0,52 | 0,895101484 | 10,34880157 | 7,03008777 | 0,008015142 | 0,024454642 |
| b1652 | 648 | 0,54 | 0,815964274 | 4,116794694 | 7,027411474 | 0,00802713 | 0,024474556 |
| b0687 | 546 | 0,5 | 0,599669087 | 7,045354716 | 7,024278495 | 0,008041186 | 0,024500746 |
| b2676 | 960 | 0,48 | 0,99668206 | 3,047635912 | 7,020132222 | 0,008059828 | 0,024519369 |
| b4481 | 1080 | 0,53 | 0,522907101 | 5,504709169 | 7,018735324 | 0,008066118 | 0,024519369 |
| b4277 | 330 | 0,58 | 2,469863914 | 0,763740132 | 7,018102976 | 0,008068967 | 0,024519369 |
| b3984 | 705 | 0,52 | 0,929098758 | 10,66794625 | 7,01805557 | 0,008069181 | 0,024519369 |
| b0938 | 540 | 0,42 | 1,316828651 | 2,482729578 | 7,007305744 | 0,008117775 | 0,024650318 |
| b2926 | 1164 | 0,52 | -0,723212662 | 11,23594593 | 6,996474872 | 0,008167039 | 0,02478312 |
| b1969 | 672 | 0,45 | 0,631486418 | 4,7054904 | 6,98949593 | 0,008198944 | 0,024863104 |
| b3187 | 972 | 0,53 | 0,56337418 | 7,643423545 | 6,983284204 | 0,008227449 | 0,024932675 |
| b0301 | 594 | 0,45 | 1,631632465 | 1,800690739 | 6,982067311 | 0,008233045 | 0,024932776 |
| b1505 | 1227 | 0,45 | 2,204032937 | 1,167119405 | 6,958729243 | 0,008341125 | 0,025243028 |
| b0558 | 411 | 0,32 | -2,031589194 | 1,78338217 | 6,954754474 | 0,008359677 | 0,0252821 |
| b2518 | 432 | 0,52 | 1,595005765 | 6,54545287 | 6,951927883 | 0,008372895 | 0,025305002 |
| b1195 | 255 | 0,53 | -0,904232012 | 6,377778027 | 6,945549522 | 0,008402802 | 0,025378274 |
| b4059 | 537 | 0,58 | -0,587951689 | 8,545646564 | 6,927491617 | 0,008488065 | 0,025618525 |
| b1296 | 1386 | 0,53 | 1,292282264 | 5,178360837 | 6,918835617 | 0,008529249 | 0,025725502 |
| b1581 | 1215 | 0,51 | 0,937734937 | 3,465920105 | 6,908257874 | 0,008579855 | 0,025860732 |
| b0561 | 438 | 0,49 | 2,019638408 | 1,334182608 | 6,902970996 | 0,008605263 | 0,025919885 |
| b2140 | 948 | 0,54 | 0,812634834 | 3,573864724 | 6,886176897 | 0,008686486 | 0,026146963 |
| b3671 | 1689 | 0,56 | 0,768589543 | 7,239046014 | 6,87513159 | 0,008740332 | 0,026291388 |
| b3333 | 1164 | 0,49 | -0,803841755 | 4,317488984 | 6,872951492 | 0,008751001 | 0,026305825 |
| b2605 | 483 | 0,51 | -0,750401124 | 4,210860121 | 6,85910559 | 0,008819068 | 0,02649267 |
| b4392 | 1938 | 0,55 | -0,547770013 | 8,330111115 | 6,855042692 | 0,008839144 | 0,026535195 |
| b2551 | 1254 | 0,53 | 1,254934394 | 10,23602255 | 6,849106346 | 0,008868562 | 0,026605687 |
| b1251 | 297 | 0,49 | 0,62626881 | 6,246134434 | 6,838869918 | 0,008919525 | 0,026740242 |
| b2121 | 1137 | 0,54 | 1,462534875 | 1,887762226 | 6,837704434 | 0,008925346 | 0,026740242 |
| b3388 | 1287 | 0,55 | 0,540174396 | 8,614474756 | 6,833467119 | 0,008946544 | 0,026785844 |
| b1311 | 882 | 0,54 | 1,378453774 | 1,787554464 | 6,813951045 | 0,009044842 | 0,027037898 |
| b2768 | 576 | 0,47 | -1,016826631 | 3,04905482 | 6,813783035 | 0,009045693 | 0,027037898 |
| b3813 | 2163 | 0,58 | 0,564060985 | 7,13345073 | 6,813164182 | 0,009048828 | 0,027037898 |

| | | | | | | | |
|---|---|---|---|---|---|---|---|
| b2892 | 1734 | 0,57 | 0,511325385 | 7,007187423 | 6,810838466 | 0,009060621 | 0,02705019 |
| b1872 | 2430 | 0,51 | -0,761072403 | 4,856908464 | 6,809973209 | 0,009065012 | 0,02705019 |
| b0152 | 891 | 0,55 | 1,266048429 | 3,295845307 | 6,796135997 | 0,009135538 | 0,027242501 |
| b1823 | 210 | 0,47 | 0,732684007 | 9,735767938 | 6,793228045 | 0,00915043 | 0,027268768 |
| b2709 | 1515 | 0,57 | -0,708803934 | 6,323114858 | 6,772350193 | 0,009258084 | 0,027571251 |
| b3650 | 2109 | 0,54 | 0,623104345 | 8,087708758 | 6,768225125 | 0,009279507 | 0,027616701 |
| b0179 | 1026 | 0,53 | 0,596858725 | 9,062711263 | 6,765424948 | 0,009294079 | 0,027641713 |
| b2216 | 2673 | 0,49 | 0,474917579 | 7,892911389 | 6,751986499 | 0,009364337 | 0,0278322 |
| b0249 | 240 | 0,59 | 1,31274616 | 2,201784972 | 6,748764469 | 0,009381263 | 0,027864028 |
| b1202 | 2868 | 0,5 | -0,650896347 | 5,093886404 | 6,744523993 | 0,009403586 | 0,027911837 |
| b0961 | 447 | 0,48 | 0,939613138 | 3,547411189 | 6,742296411 | 0,009415335 | 0,027928214 |
| b2046 | 1479 | 0,54 | 1,519009385 | 1,996710368 | 6,728161729 | 0,009490236 | 0,02813177 |
| b1493 | 1401 | 0,53 | -1,463146506 | 11,61063792 | 6,721098327 | 0,009527893 | 0,028220925 |
| b0389 | 192 | 0,48 | -0,625528656 | 6,4181121 | 6,720160473 | 0,009532905 | 0,028220925 |
| b2109 | 2481 | 0,46 | 1,150583139 | 2,682157737 | 6,681447741 | 0,009742147 | 0,028821321 |
| b4401 | 717 | 0,51 | 0,746591352 | 10,34626147 | 6,675423035 | 0,009775131 | 0,028899826 |
| b1832 | 498 | 0,49 | 0,746087136 | 6,97532133 | 6,665704319 | 0,00982858 | 0,029038693 |
| b2475 | 864 | 0,54 | -0,721999897 | 7,775048819 | 6,656592031 | 0,009878967 | 0,029168333 |
| b1319 | 906 | 0,5 | 2,21671701 | 0,932046231 | 6,654582376 | 0,009890115 | 0,029182024 |
| b4456 | 207 | 0,47 | -0,543474556 | 8,518784542 | 6,646968489 | 0,009932468 | 0,029287711 |
| b4475 | 1017 | 0,6 | -0,85082811 | 5,121914065 | 6,64240233 | 0,009957956 | 0,029343564 |
| b2992 | 489 | 0,54 | -0,854289537 | 5,528944485 | 6,628676112 | 0,010034982 | 0,02955111 |
| b3400 | 402 | 0,53 | 0,6150447 | 5,410542763 | 6,624858644 | 0,010056512 | 0,029595068 |
| b3529 | 1989 | 0,53 | -0,476316252 | 6,505203644 | 6,623669971 | 0,010063226 | 0,029595393 |
| b2510 | 216 | 0,48 | 0,774065306 | 3,566331063 | 6,614803554 | 0,010113449 | 0,029705161 |
| b0693 | 2199 | 0,49 | 0,960440244 | 2,995816877 | 6,614740869 | 0,010113805 | 0,029705161 |
| b2290 | 1218 | 0,5 | 0,504903066 | 6,065114666 | 6,611950322 | 0,010129666 | 0,029732261 |
| b1633 | 636 | 0,49 | 0,955808001 | 4,053139139 | 6,607188741 | 0,010156788 | 0,029792359 |
| b0539 | 279 | 0,5 | 2,149317644 | 0,948764816 | 6,604143551 | 0,010174173 | 0,029823834 |
| b2808 | 918 | 0,49 | 0,657110242 | 4,997798149 | 6,597157966 | 0,010214168 | 0,029912508 |
| b2238 | 243 | 0,41 | 1,846144074 | 1,220520055 | 6,596529999 | 0,010217771 | 0,029912508 |
| b2769 | 861 | 0,56 | 1,120509381 | 2,607465218 | 6,590391019 | 0,010253065 | 0,029984623 |
| b3667 | 1320 | 0,55 | 0,879698109 | 3,325828746 | 6,589918831 | 0,010255785 | 0,029984623 |
| b3693 | 879 | 0,54 | -0,68189364 | 4,374790629 | 6,587059352 | 0,010272271 | 0,030013247 |
| b0316 | 933 | 0,49 | -0,726362383 | 4,4418746 | 6,572193447 | 0,010358421 | 0,030231551 |
| b0986 | 645 | 0,48 | 1,540840725 | 1,792996602 | 6,571840104 | 0,010360478 | 0,030231551 |
| b1532 | 219 | 0,5 | 1,234872931 | 2,73628563 | 6,551833984 | 0,01047761 | 0,030553448 |
| b2852 | 1377 | 0,35 | 1,764461608 | 1,380739401 | 6,532306425 | 0,010593251 | 0,030861751 |
| b2270 | 1728 | 0,47 | 0,875684245 | 3,358559871 | 6,531659002 | 0,010597107 | 0,030861751 |
| b0642 | 2583 | 0,53 | 0,588045795 | 9,929683208 | 6,49912045 | 0,010792787 | 0,031411215 |
| b2580 | 690 | 0,52 | 0,492779921 | 5,525674079 | 6,489401323 | 0,010851952 | 0,031562912 |
| b4409 | 126 | 0,43 | -1,033986176 | 3,867674367 | 6,479015946 | 0,01091554 | 0,03172727 |
| b0018 | 210 | 0,51 | -0,974670468 | 3,493923309 | 6,461631174 | 0,011022841 | 0,032018389 |
| b2355 | 579 | 0,51 | 2,437777383 | 0,833646772 | 6,45548256 | 0,01106105 | 0,032108566 |
| b2214 | 1056 | 0,53 | 1,074507765 | 3,757510533 | 6,448955325 | 0,01110176 | 0,032205883 |
| b0556 | 462 | 0,51 | 1,37155684 | 2,432730282 | 6,447632661 | 0,011110028 | 0,032209021 |
| b0263 | 348 | 0,56 | 1,542508294 | 1,457773336 | 6,435638462 | 0,011185295 | 0,032406264 |
| b0233 | 399 | 0,44 | 0,853413267 | 3,240072858 | 6,433497349 | 0,011198786 | 0,032424391 |
| b1001 | 1257 | 0,34 | 1,468864485 | 1,77072871 | 6,420703553 | 0,011279747 | 0,032637717 |
| b1713 | 2388 | 0,54 | 0,567611027 | 10,19940956 | 6,413249281 | 0,011327195 | 0,032753863 |
| b2062 | 1140 | 0,54 | 2,113927776 | 1,335648124 | 6,40318 | 0,011391614 | 0,0329189 |
| b3219 | 717 | 0,44 | -0,637430686 | 4,655119366 | 6,400792436 | 0,011406944 | 0,03294196 |
| b3282 | 573 | 0,52 | 0,580119355 | 5,47245571 | 6,39818128 | 0,011423733 | 0,032969203 |
| b2453 | 1188 | 0,59 | 0,922910265 | 2,985779659 | 6,395403448 | 0,011441622 | 0,032999583 |
| b1235 | 1014 | 0,49 | -0,592007246 | 7,80683214 | 6,387428007 | 0,011493144 | 0,033126862 |
| b3589 | 1152 | 0,51 | 1,636518618 | 1,583560677 | 6,371713422 | 0,011595358 | 0,033399998 |
| b2985 | 714 | 0,5 | 1,786654352 | 1,210475094 | 6,349050436 | 0,011744414 | 0,033807621 |
| b4477 | 618 | 0,59 | -1,050821422 | 3,045088151 | 6,341331366 | 0,011795631 | 0,03393326 |
| b3167 | 402 | 0,53 | 0,460533733 | 6,63470114 | 6,339572022 | 0,011807337 | 0,033945146 |
| b0245 | 342 | 0,51 | 1,266528661 | 1,973344124 | 6,335057048 | 0,011837431 | 0,03400985 |
| b1245 | 909 | 0,52 | 0,721869611 | 9,451615662 | 6,322596786 | 0,011920893 | 0,034227702 |
| b1613 | 1176 | 0,51 | -0,543620493 | 7,744072598 | 6,303798701 | 0,012047953 | 0,034570374 |
| b1569 | 231 | 0,47 | 1,98371291 | 1,064150299 | 6,302510132 | 0,012056713 | 0,034573377 |
| b3999 | 591 | 0,49 | 0,520624548 | 6,040965856 | 6,299681436 | 0,012075967 | 0,034606448 |
| b4542 | 108 | 0,49 | 1,39452564 | 2,173109851 | 6,293378111 | 0,012118986 | 0,034707536 |
| b2390 | 327 | 0,54 | 0,822578352 | 3,973216866 | 6,29003665 | 0,012141854 | 0,034729261 |
| b2634 | 702 | 0,57 | 1,295207925 | 2,246232975 | 6,290005319 | 0,012142069 | 0,034729261 |
| b0821 | 1266 | 0,56 | -1,182978877 | 5,659050019 | 6,280571293 | 0,012206874 | 0,034892354 |
| b1833 | 1284 | 0,5 | -0,502164605 | 5,65654074 | 6,259442968 | 0,012353304 | 0,035288406 |
| b2873 | 1386 | 0,53 | 0,901530295 | 3,031812954 | 6,249291762 | 0,012424297 | 0,035468599 |
| b3983 | 429 | 0,52 | 0,857661136 | 9,561116436 | 6,243244294 | 0,01246679 | 0,035558166 |
| b1194 | 735 | 0,51 | 1,519939143 | 1,713170657 | 6,242569766 | 0,012471539 | 0,035558166 |
| b0486 | 1293 | 0,53 | -0,74763107 | 9,446925552 | 6,237805682 | 0,012505132 | 0,035624993 |
| b4325 | 831 | 0,4 | 2,400888329 | 0,834143942 | 6,236221902 | 0,01251632 | 0,035624993 |
| b2983 | 1068 | 0,53 | 1,059231142 | 2,298907328 | 6,235867849 | 0,012518823 | 0,035624993 |
| b2752 | 909 | 0,53 | 1,161569712 | 3,006290466 | 6,232614646 | 0,012541842 | 0,035650347 |
| b4076 | 597 | 0,52 | 1,248522738 | 2,138633319 | 6,232360648 | 0,012543641 | 0,035650347 |
| b2723 | 1827 | 0,58 | 1,007715081 | 2,312070064 | 6,219991745 | 0,01263157 | 0,035877502 |
| b0177 | 2433 | 0,51 | 0,589126447 | 10,10545554 | 6,202555712 | 0,012756598 | 0,036209674 |
| b0146 | 705 | 0,49 | -0,592574891 | 8,491048799 | 6,20053869 | 0,012771144 | 0,036228017 |
| b0578 | 654 | 0,52 | 0,542684262 | 7,077614818 | 6,199339033 | 0,012779803 | 0,03622965 |
| b2984 | 759 | 0,53 | 1,827015452 | 1,239995307 | 6,196660405 | 0,012799159 | 0,036261587 |
| b3399 | 669 | 0,52 | 0,461287794 | 6,133220372 | 6,193042737 | 0,012825349 | 0,036312832 |
| b1515 | 993 | 0,53 | 1,219000381 | 5,688206677 | 6,171927306 | 0,012979313 | 0,0367047 |
| b0512 | 1362 | 0,52 | -0,892364739 | 4,834626923 | 6,171336703 | 0,012983647 | 0,0367047 |
| b4366 | 678 | 0,45 | 1,325097947 | 1,966094774 | 6,170699974 | 0,012988321 | 0,0367047 |
| b2336 | 753 | 0,51 | 1,821741252 | 1,288819324 | 6,153142921 | 0,013117878 | 0,037047466 |
| b4113 | 669 | 0,53 | -0,492844664 | 6,245857489 | 6,14770434 | 0,013158279 | 0,037138166 |
| b0869 | 1431 | 0,55 | 0,500292347 | 6,330688966 | 6,124247376 | 0,013334004 | 0,037610451 |
| b3690 | 1065 | 0,51 | 1,295333913 | 2,123847413 | 6,11520085 | 0,013402418 | 0,037779647 |
| b0385 | 1116 | 0,54 | 0,997004991 | 3,014510195 | 6,108827605 | 0,013450832 | 0,037892288 |
| b0129 | 441 | 0,49 | -0,78853805 | 5,434949686 | 6,104794733 | 0,013481561 | 0,037954997 |
| b2325 | 279 | 0,53 | 0,856739714 | 4,131102091 | 6,097897203 | 0,013534284 | 0,038079511 |
| b1842 | 231 | 0,48 | 0,731330238 | 3,86852241 | 6,09466006 | 0,013559101 | 0,038125402 |

| | | | | | | | |
|---|---|---|---|---|---|---|---|
| b3921 | 441 | 0,49 | 0,797153669 | 3,853682291 | 6,091725955 | 0,013581635 | 0,038141311 |
| b0592 | 957 | 0,55 | 1,196079252 | 2,927409552 | 6,091707276 | 0,013581779 | 0,038141311 |
| b3622 | 1260 | 0,32 | 0,623847415 | 5,738307792 | 6,089783144 | 0,013596577 | 0,03815896 |
| b3555 | 291 | 0,44 | -0,623880673 | 8,713940535 | 6,085081379 | 0,013632809 | 0,038236701 |
| b2846 | 633 | 0,37 | 1,818597181 | 1,761918702 | 6,066669848 | 0,013775645 | 0,038613158 |
| b2327 | 810 | 0,53 | 0,617582029 | 4,76723161 | 6,060410001 | 0,013824559 | 0,038696533 |
| b3568 | 1182 | 0,54 | -0,995545083 | 4,499535553 | 6,058729704 | 0,013837719 | 0,038696533 |
| b0846 | 537 | 0,52 | -0,658248106 | 4,680469707 | 6,058538715 | 0,013839215 | 0,038696533 |
| b4213 | 1944 | 0,49 | -0,710170661 | 7,713274314 | 6,058448188 | 0,013839925 | 0,038696533 |
| b0217 | 786 | 0,43 | 1,426231581 | 1,527731621 | 6,053233947 | 0,013880851 | 0,038786767 |
| b1915 | 225 | 0,48 | 1,05660508 | 3,733124144 | 6,050068608 | 0,013905757 | 0,038825822 |
| b4360 | 495 | 0,51 | 0,71739618 | 5,030892439 | 6,049256579 | 0,013912153 | 0,038825822 |
| b4538 | 186 | 0,43 | 1,532818815 | 1,481632624 | 6,040595435 | 0,013980568 | 0,038974691 |
| b3902 | 825 | 0,56 | 1,380863698 | 2,051436777 | 6,040302533 | 0,013982888 | 0,038974691 |
| b3280 | 258 | 0,49 | 0,823976625 | 4,467715682 | 6,022644319 | 0,01412347 | 0,03934207 |
| b3268 | 1025 | 0,5 | -0,834912861 | 4,693817028 | 6,019447391 | 0,014149076 | 0,03938892 |
| b3644 | 864 | 0,54 | 0,569463875 | 7,142380197 | 6,018183264 | 0,014159215 | 0,039392676 |
| b3249 | 489 | 0,51 | 0,560462494 | 4,883874074 | 6,014264602 | 0,014190691 | 0,039455755 |
| b4256 | 504 | 0,52 | 0,756398892 | 4,183091454 | 5,971278684 | 0,014540733 | 0,040403947 |
| b1518 | 291 | 0,46 | 1,387067152 | 5,287311103 | 5,957967045 | 0,014650925 | 0,040684911 |
| b4058 | 2823 | 0,56 | 0,501983333 | 8,431951206 | 5,955213398 | 0,014673827 | 0,040723276 |
| b0689 | 495 | 0,45 | 1,495597712 | 1,684197338 | 5,950160857 | 0,014715943 | 0,040814887 |
| b0943 | 516 | 0,47 | 1,053862196 | 2,197620545 | 5,931675881 | 0,014871093 | 0,041219689 |
| b3197 | 987 | 0,52 | 0,407421976 | 7,092245864 | 5,902940137 | 0,015115632 | 0,041871608 |
| b0836 | 384 | 0,49 | -1,527939855 | 8,564019202 | 5,894453825 | 0,015188639 | 0,042047857 |
| b0230 | 756 | 0,53 | 1,149578896 | 2,573380906 | 5,879493878 | 0,015318224 | 0,042380421 |
| b1682 | 747 | 0,5 | -0,647149868 | 7,175292361 | 5,859827477 | 0,015490312 | 0,042830092 |
| b2940 | 216 | 0,48 | 1,076999847 | 5,378836195 | 5,851166815 | 0,015566727 | 0,042974663 |
| b0630 | 642 | 0,48 | 0,760818715 | 4,82597873 | 5,85116079 | 0,01556678 | 0,042974663 |
| b3778 | 2022 | 0,53 | 0,461233176 | 5,965175763 | 5,850642638 | 0,015571364 | 0,042974663 |
| b1010 | 387 | 0,54 | 1,730387217 | 1,251498585 | 5,844798568 | 0,015623162 | 0,043091085 |
| b0139 | 2598 | 0,43 | 1,26967201 | 2,623226064 | 5,835318696 | 0,015707564 | 0,043297234 |
| b1814 | 1365 | 0,54 | 0,551964846 | 8,729827468 | 5,829868016 | 0,015756306 | 0,043404893 |
| b3775 | 282 | 0,52 | 0,67963557 | 4,549243765 | 5,821267606 | 0,01583353 | 0,043590836 |
| b0238 | 459 | 0,52 | 0,88918346 | 5,009988352 | 5,818977851 | 0,015854156 | 0,043610383 |
| b2169 | 1131 | 0,56 | 0,483138786 | 6,671876777 | 5,818157111 | 0,015861556 | 0,043610383 |
| b1678 | 1005 | 0,52 | -0,866395645 | 9,384082738 | 5,817240888 | 0,01586982 | 0,043610383 |
| b3683 | 1617 | 0,52 | 1,039339853 | 2,661683283 | 5,804787905 | 0,015982595 | 0,043893378 |
| b3347 | 813 | 0,48 | 0,719966378 | 7,675963842 | 5,803110959 | 0,015997845 | 0,043908353 |
| b2405 | 885 | 0,51 | 0,672669852 | 4,376672283 | 5,799564879 | 0,016030141 | 0,043970069 |
| b0930 | 1401 | 0,52 | 0,520723826 | 8,689069917 | 5,771747172 | 0,016285835 | 0,044644106 |
| b1539 | 747 | 0,51 | -0,504550425 | 8,093201092 | 5,769618699 | 0,016305572 | 0,044670889 |
| b3394 | 540 | 0,55 | 1,145745818 | 1,737263185 | 5,755812034 | 0,016434198 | 0,04496952 |
| b1769 | 1359 | 0,45 | 1,133611068 | 2,56029869 | 5,75576439 | 0,016434644 | 0,04496952 |
| b4025 | 1650 | 0,51 | -0,650587751 | 10,29104153 | 5,754028082 | 0,016450894 | 0,04498652 |
| b3525 | 768 | 0,53 | 1,524994042 | 1,562724689 | 5,749601096 | 0,016492401 | 0,045072526 |
| b3835 | 1641 | 0,5 | -0,437219274 | 7,057175424 | 5,748208428 | 0,016505481 | 0,045080784 |
| b2959 | 327 | 0,49 | 0,608557179 | 8,186429761 | 5,742693761 | 0,01655738 | 0,045194993 |
| b0445 | 1701 | 0,53 | -0,69972643 | 5,926959529 | 5,733998656 | 0,016639553 | 0,045387819 |
| b2461 | 480 | 0,5 | 1,021341606 | 2,798767378 | 5,732681087 | 0,016652041 | 0,045387819 |
| b1608 | 720 | 0,49 | 0,816246428 | 4,66725717 | 5,731727311 | 0,016661087 | 0,045387819 |
| b1482 | 432 | 0,5 | -0,740097174 | 10,20300823 | 5,730763082 | 0,016670237 | 0,045387819 |
| b0251 | 444 | 0,51 | 0,998848516 | 2,399591008 | 5,729876418 | 0,016678656 | 0,045387819 |
| b0472 | 606 | 0,58 | 0,557905445 | 6,899363912 | 5,711398868 | 0,016855103 | 0,045840153 |
| b0959 | 630 | 0,48 | -0,510498747 | 5,373323023 | 5,698413169 | 0,016980258 | 0,046152526 |
| b2953 | 291 | 0,48 | 0,691667324 | 3,62940222 | 5,689618079 | 0,017065568 | 0,046356289 |
| b1961 | 1419 | 0,52 | 0,575398416 | 5,847890157 | 5,686561851 | 0,017095316 | 0,046362212 |
| b4407 | 201 | 0,56 | 2,001577945 | 1,348319566 | 5,686385122 | 0,017097038 | 0,046362212 |
| b3732 | 1383 | 0,55 | 0,952071441 | 11,59678094 | 5,686206255 | 0,017098781 | 0,046362212 |
| b4323 | 1461 | 0,58 | -0,857668409 | 6,504338861 | 5,683858215 | 0,017121677 | 0,046396226 |
| b3829 | 2262 | 0,56 | -0,897946429 | 6,641321155 | 5,680402449 | 0,017155432 | 0,046445369 |
| b4043 | 609 | 0,52 | -0,524430725 | 7,681761898 | 5,679880346 | 0,017160538 | 0,046445369 |
| b4547 | 201 | 0,48 | -0,765794639 | 5,611425866 | 5,677881149 | 0,017180103 | 0,046470261 |
| b3649 | 276 | 0,53 | 0,471779362 | 6,842774438 | 5,675695745 | 0,017201517 | 0,04650012 |
| b0421 | 900 | 0,54 | 0,764668447 | 6,586972236 | 5,66981217 | 0,017259304 | 0,04662821 |
| b3560 | 912 | 0,56 | 0,576808156 | 8,427730106 | 5,665038534 | 0,017306337 | 0,046727109 |
| b0222 | 579 | 0,51 | 0,470824389 | 7,754026926 | 5,661985155 | 0,01733649 | 0,046769078 |
| b2911 | 183 | 0,55 | -1,021116175 | 11,6235428 | 5,661351863 | 0,017342751 | 0,046769078 |
| b4633 | 222 | 0,53 | 0,90858877 | 2,838506367 | 5,657530452 | 0,017380579 | 0,046842907 |
| b2152 | 1158 | 0,54 | 0,768736303 | 4,457148918 | 5,651184746 | 0,017443583 | 0,046984459 |
| b2939 | 132 | 0,41 | 1,325775826 | 2,58977356 | 5,630641443 | 0,017649172 | 0,047509663 |
| b2522 | 777 | 0,47 | 0,629043267 | 5,660554871 | 5,61921602 | 0,017764594 | 0,047791662 |
| b2528 | 324 | 0,52 | 0,7550338 | 7,160781602 | 5,604373055 | 0,017915704 | 0,048169279 |
| b4478 | 1149 | 0,53 | -0,708742013 | 4,145538817 | 5,602905096 | 0,017930721 | 0,048180751 |
| b0178 | 486 | 0,5 | 0,530183509 | 9,003844555 | 5,581781749 | 0,01814825 | 0,048736043 |
| b1726 | 537 | 0,44 | -0,495771476 | 5,926883052 | 5,580571136 | 0,018160799 | 0,04874054 |
| b0286 | 690 | 0,55 | 0,898699848 | 3,843590364 | 5,57586659 | 0,018209652 | 0,048820779 |
| b1736 | 351 | 0,5 | 1,202877623 | 2,874905826 | 5,57542005 | 0,018214295 | 0,048820779 |
| b2257 | 1653 | 0,51 | -0,6143865 | 5,068844121 | 5,574547416 | 0,018223374 | 0,048820779 |
| b0535 | 633 | 0,37 | 2,276353005 | 0,884420457 | 5,572170069 | 0,018248132 | 0,048857901 |
| b0324 | 1383 | 0,56 | 0,52000493 | 5,315959044 | 5,569758799 | 0,018273278 | 0,048896018 |
| b4041 | 2424 | 0,55 | -0,558759176 | 8,736793924 | 5,557870075 | 0,018397785 | 0,049177956 |
| b2562 | 261 | 0,45 | 1,23315427 | 1,897342579 | 5,557603418 | 0,018400587 | 0,049177956 |
| b4567 | 237 | 0,48 | 2,030875993 | 1,096203434 | 5,55250558 | 0,018454252 | 0,049291988 |
| b3821 | 870 | 0,51 | 0,679850162 | 5,256218129 | 5,546967764 | 0,018512731 | 0,049393303 |
| b1789 | 447 | 0,55 | 1,050789107 | 2,685994231 | 5,546826672 | 0,018514223 | 0,049393303 |
| b2017 | 252 | 0,48 | 0,561696534 | 5,349437713 | 5,543089355 | 0,018553801 | 0,049469444 |
| b3236 | 939 | 0,52 | -0,603892588 | 12,08113307 | 5,534347966 | 0,018646712 | 0,049687613 |
| b3052 | 1434 | 0,56 | 0,459618345 | 7,842463723 | 5,527047823 | 0,018724673 | 0,049865707 |
| b0862 | 717 | 0,55 | -0,415701817 | 7,68512025 | 5,52355811 | 0,01876206 | 0,049935601 |
| b4278 | 1329 | 0,54 | 0,571080379 | 4,466642818 | 5,522153925 | 0,018777125 | 0,049946039 |
| b1973 | 651 | 0,38 | -1,179710447 | 2,329264262 | 5,516960423 | 0,018832954 | 0,050064829 |
| b3152 | 636 | 0,55 | -0,651392332 | 5,682667548 | 5,501624851 | 0,018998812 | 0,050475801 |
| b0221 | 2445 | 0,57 | 0,762381692 | 8,246923303 | 5,484703389 | 0,019183575 | 0,050936482 |

| | | | | | | | |
|---|---|---|---|---|---|---|---|
| b2975 | 1683 | 0,53 | -2,375023397 | 8,408179175 | 5,482145936 | 0,01921166 | 0,050974925 |
| b3301 | 435 | 0,54 | 0,75436545 | 10,9314355 | 5,481314544 | 0,019220799 | 0,050974925 |
| b3138 | 477 | 0,44 | 2,330987302 | 0,816767825 | 5,464443953 | 0,019407228 | 0,051429677 |
| b2547 | 1512 | 0,56 | -0,693818172 | 3,509609589 | 5,463724567 | 0,019415219 | 0,051429677 |
| b1130 | 672 | 0,48 | -0,456667225 | 7,325709188 | 5,441727072 | 0,01966122 | 0,052050553 |
| b1523 | 360 | 0,53 | 0,925737615 | 3,340138018 | 5,433699047 | 0,019751798 | 0,052252444 |
| b4190 | 750 | 0,53 | -0,648223372 | 5,192460724 | 5,432903562 | 0,019760797 | 0,052252444 |
| b0494 | 627 | 0,53 | 0,467782761 | 5,81321847 | 5,431375646 | 0,019778093 | 0,052267343 |
| b0039 | 1143 | 0,54 | -0,775121613 | 5,312519305 | 5,430280476 | 0,0197905 | 0,052269312 |
| b0066 | 699 | 0,57 | 0,786382164 | 3,135675659 | 5,420261673 | 0,019904377 | 0,052539114 |
| b0982 | 447 | 0,52 | 0,924382029 | 2,863130924 | 5,417379886 | 0,019937257 | 0,05259493 |
| b3964 | 360 | 0,49 | -0,501147464 | 6,810418802 | 5,409344954 | 0,020029231 | 0,052806477 |
| b4516 | 276 | 0,53 | 2,23803848 | 0,863557416 | 5,403396388 | 0,020097605 | 0,052955593 |
| b0699 | 207 | 0,5 | -1,22581487 | 5,841639118 | 5,397000073 | 0,020171395 | 0,053116908 |
| b3244 | 1446 | 0,55 | -0,513252763 | 8,154434174 | 5,396037004 | 0,020182529 | 0,053116908 |
| b0009 | 588 | 0,54 | -0,531790276 | 6,900772553 | 5,392402576 | 0,020224606 | 0,05319641 |
| b1344 | 936 | 0,49 | 0,477554057 | 5,69149116 | 5,369639442 | 0,020490213 | 0,053863421 |
| b0694 | 678 | 0,57 | -0,613432967 | 4,847855631 | 5,36397273 | 0,020556893 | 0,05400703 |
| b0922 | 1323 | 0,53 | 0,441422882 | 6,863597348 | 5,35003754 | 0,020721825 | 0,054408446 |
| b2474 | 2016 | 0,55 | -0,461789543 | 6,615758905 | 5,348199388 | 0,020743682 | 0,054433948 |
| b2350 | 363 | 0,46 | 1,372935781 | 4,101400981 | 5,345039241 | 0,020781316 | 0,054490939 |
| b4207 | 621 | 0,56 | 0,721476753 | 7,153875046 | 5,34433468 | 0,020789716 | 0,054490939 |
| b1278 | 765 | 0,51 | -0,608678623 | 6,260979118 | 5,330178135 | 0,020959243 | 0,054903172 |
| b0278 | 699 | 0,37 | 1,43527826 | 1,792694425 | 5,323220742 | 0,021043083 | 0,055090594 |
| b4691 | 161 | 0,45 | 1,153254608 | 2,605714397 | 5,313736602 | 0,02115793 | 0,055358928 |
| b2069 | 1353 | 0,56 | 0,680013748 | 3,961132063 | 5,310321686 | 0,021199442 | 0,055435179 |
| b0589 | 993 | 0,58 | 1,049871281 | 2,679701297 | 5,308282727 | 0,021224267 | 0,055467735 |
| b1253 | 399 | 0,48 | 1,251067704 | 3,622628996 | 5,306393587 | 0,021247295 | 0,055495559 |
| b3181 | 477 | 0,48 | 0,858757574 | 5,536977558 | 5,305300606 | 0,021260631 | 0,055498047 |
| b3702 | 1404 | 0,54 | 0,478562643 | 7,732399094 | 5,300479382 | 0,021319556 | 0,05561947 |
| b2785 | 1302 | 0,51 | -0,420855058 | 7,101104229 | 5,299379177 | 0,021333027 | 0,055622237 |
| b3709 | 1248 | 0,46 | 1,87060877 | 8,393691199 | 5,298327417 | 0,021345912 | 0,055623476 |
| b1853 | 870 | 0,49 | -0,484628471 | 6,712239897 | 5,276905556 | 0,021610122 | 0,056279237 |
| b2215 | 1104 | 0,51 | -0,662691943 | 14,73654399 | 5,269080221 | 0,021707479 | 0,056499955 |
| b2803 | 1449 | 0,51 | -0,818871723 | 5,402864179 | 5,2658697 | 0,021747554 | 0,056571408 |
| b4598 | 96 | 0,33 | 0,69804407 | 5,330454157 | 5,26423247 | 0,02176802 | 0,0565918 |
| b2806 | 1101 | 0,52 | 0,456051481 | 6,044572843 | 5,259273727 | 0,021830127 | 0,056720364 |
| b4160 | 969 | 0,53 | 0,426755745 | 7,07172528 | 5,257326829 | 0,021854561 | 0,056750952 |
| b3486 | 2736 | 0,57 | -0,568950167 | 8,278434983 | 5,255381023 | 0,02187901 | 0,056754197 |
| b3656 | 2319 | 0,52 | -0,60354974 | 4,775378824 | 5,255211942 | 0,021881136 | 0,056754197 |
| b3050 | 630 | 0,43 | 1,041888422 | 2,154316872 | 5,247046442 | 0,021984056 | 0,056969881 |
| b2572 | 651 | 0,52 | -0,589127385 | 9,06257174 | 5,246598767 | 0,021989713 | 0,056969881 |
| b2232 | 723 | 0,54 | 0,675772572 | 5,677676518 | 5,241174702 | 0,022058375 | 0,057114751 |
| b0258 | 1108 | 0,55 | 0,657763284 | 4,536453133 | 5,238487945 | 0,022092468 | 0,057166882 |
| b0405 | 1071 | 0,54 | 0,951664298 | 4,644687172 | 5,237578694 | 0,022104018 | 0,057166882 |
| b1697 | 765 | 0,51 | 1,35422469 | 2,103022769 | 5,23258329 | 0,022167585 | 0,057298222 |
| b0402 | 1374 | 0,53 | 0,619707369 | 5,156640864 | 5,230718371 | 0,022191365 | 0,057324036 |
| b3793 | 1353 | 0,52 | 0,508227536 | 5,054561423 | 5,229794794 | 0,022203152 | 0,057324036 |
| b2384 | 1038 | 0,58 | 1,193108168 | 1,997103559 | 5,227253067 | 0,022235622 | 0,057374818 |
| b1256 | 639 | 0,5 | -1,126117794 | 8,282133834 | 5,223934454 | 0,022278092 | 0,057451327 |
| b3698 | 399 | 0,51 | -0,597255353 | 5,343805534 | 5,212652329 | 0,022423102 | 0,057792032 |
| b2063 | 1584 | 0,53 | -0,549918389 | 5,96356021 | 5,209556552 | 0,022463063 | 0,057861753 |
| b3748 | 420 | 0,48 | 0,641423981 | 8,276909724 | 5,187458457 | 0,022750464 | 0,058568397 |
| b4496 | 1193 | 0,46 | -0,802096241 | 6,150842627 | 5,175593775 | 0,022906342 | 0,058935834 |
| b1034 | 738 | 0,48 | 0,450599656 | 6,857808933 | 5,149814454 | 0,023248857 | 0,059782776 |
| b3346 | 723 | 0,47 | 0,442425908 | 6,950662745 | 5,14709587 | 0,023285286 | 0,059842116 |
| b3398 | 2136 | 0,53 | 0,480628937 | 6,144637449 | 5,142282444 | 0,02334993 | 0,05997386 |
| b2149 | 1521 | 0,45 | 2,049339532 | 7,57299067 | 5,13966791 | 0,023385121 | 0,060029847 |
| b1492 | 1536 | 0,48 | -1,2025037 | 12,4752756 | 5,138336163 | 0,023403067 | 0,060041527 |
| b0077 | 1725 | 0,53 | 0,577905183 | 4,493002373 | 5,136137681 | 0,023432725 | 0,060083222 |
| b3009 | 660 | 0,51 | -0,582632175 | 6,624774233 | 5,126486231 | 0,023563383 | 0,060383696 |
| b0242 | 1104 | 0,56 | 0,428930851 | 7,451340255 | 5,119143161 | 0,023663298 | 0,060605087 |
| b1261 | 1194 | 0,55 | -2,939896761 | 11,21404181 | 5,117783399 | 0,023681848 | 0,060617957 |
| b4141 | 1257 | 0,52 | 0,553084813 | 4,361215844 | 5,113672413 | 0,023738022 | 0,060727063 |
| b0424 | 591 | 0,57 | -0,529739858 | 5,917864209 | 5,112515026 | 0,023753862 | 0,06073292 |
| b3810 | 708 | 0,56 | 0,495079672 | 6,294917624 | 5,105161761 | 0,023854755 | 0,060956106 |
| b1930 | 234 | 0,48 | 0,934283441 | 5,371625569 | 5,097856027 | 0,023955435 | 0,061178496 |
| b0602 | 1221 | 0,48 | 1,227513412 | 2,719381893 | 5,095267924 | 0,023991208 | 0,061234962 |
| b0110 | 552 | 0,54 | -0,478289723 | 5,572116233 | 5,09148652 | 0,024043574 | 0,061333692 |
| b0368 | 852 | 0,54 | 0,895305535 | 2,867037806 | 5,082387148 | 0,02417007 | 0,061621306 |
| b2051 | 480 | 0,55 | 1,775975374 | 1,217731061 | 5,0767956 | 0,024248145 | 0,061765889 |
| b1584 | 561 | 0,47 | -0,477563011 | 6,076964273 | 5,076352511 | 0,024254343 | 0,061765889 |
| b2151 | 1041 | 0,52 | 1,063574393 | 5,518525506 | 5,066026377 | 0,024399253 | 0,062095321 |
| b4676 | 99 | 0,42 | 1,521323212 | 1,344444834 | 5,06424627 | 0,024424324 | 0,062095321 |
| b0530 | 543 | 0,45 | 1,250956458 | 1,810859458 | 5,06417932 | 0,024425268 | 0,062095321 |
| b1447 | 450 | 0,52 | 0,832268996 | 4,094601272 | 5,062411687 | 0,024450191 | 0,062123445 |
| b4332 | 1179 | 0,54 | 0,745931187 | 4,00280238 | 5,056716424 | 0,024530673 | 0,062292621 |
| b3876 | 1404 | 0,45 | 1,098319524 | 2,116269001 | 5,031016681 | 0,024897279 | 0,063187772 |
| b1363 | 1458 | 0,38 | 0,747190468 | 3,924719808 | 5,022010229 | 0,025027098 | 0,063481297 |
| b2015 | 930 | 0,5 | -0,769953597 | 5,982860121 | 4,98571218 | 0,025557463 | 0,064733115 |
| b0507 | 1782 | 0,53 | -1,278837294 | 5,024844232 | 4,985639642 | 0,025558535 | 0,064733115 |
| b3353 | 1023 | 0,54 | 0,471756761 | 5,860707769 | 4,985273228 | 0,025563948 | 0,064733115 |
| b0915 | 987 | 0,55 | 0,438279298 | 5,826942451 | 4,97945767 | 0,025650023 | 0,0649144 |
| b3609 | 468 | 0,51 | 0,485277653 | 9,161758111 | 4,975915921 | 0,025702591 | 0,06501073 |
| b2593 | 732 | 0,55 | 0,450316844 | 6,064515683 | 4,955479948 | 0,026008105 | 0,065746376 |
| b2874 | 933 | 0,49 | -0,815038541 | 3,85433139 | 4,953445756 | 0,026038721 | 0,065786667 |
| b4274 | 336 | 0,46 | -0,783724818 | 5,872726342 | 4,952050059 | 0,026059749 | 0,065802702 |
| b3312 | 192 | 0,54 | 0,734677336 | 9,145676251 | 4,948039266 | 0,026120276 | 0,065918399 |
| b2159 | 858 | 0,52 | -0,40751136 | 6,612866653 | 4,941500432 | 0,026219267 | 0,066093963 |
| b3160 | 1008 | 0,56 | 0,574925524 | 6,447314226 | 4,941495836 | 0,026219337 | 0,066093963 |
| b3149 | 591 | 0,5 | 0,4503518 | 6,200789005 | 4,935612973 | 0,02630873 | 0,066282028 |
| b3311 | 255 | 0,47 | 0,781936147 | 9,511542953 | 4,932599578 | 0,026354643 | 0,066360398 |
| b0926 | 549 | 0,5 | 0,73865181 | 6,962512287 | 4,917576555 | 0,026584783 | 0,066867015 |
| b3836 | 270 | 0,46 | -0,495786464 | 7,427388323 | 4,916579931 | 0,026600124 | 0,066867015 |

| | | | | | | | |
|---|---|---|---|---|---|---|---|
| b1214 | 810 | 0,46 | 0,436297075 | 6,29293686 | 4,91654905 | 0,0266006 | 0,066867015 |
| b1873 | 1101 | 0,47 | 1,367365871 | 1,802067845 | 4,914395908 | 0,026633775 | 0,066912882 |
| b1155 | 414 | 0,42 | 1,579432843 | 1,330814219 | 4,912123336 | 0,026668838 | 0,066963435 |
| b1917 | 753 | 0,54 | -0,503611111 | 5,747408062 | 4,88773938 | 0,02704808 | 0,067877657 |
| b2196 | 1944 | 0,57 | -1,015299511 | 6,063304367 | 4,880500453 | 0,027161743 | 0,068124752 |
| b2241 | 1629 | 0,57 | 1,031960611 | 2,110590666 | 4,867906408 | 0,027360675 | 0,068585316 |
| b1541 | 204 | 0,52 | -1,174720678 | 4,135699587 | 4,86624481 | 0,027387034 | 0,068613017 |
| b0551 | 384 | 0,43 | 1,798282412 | 1,131662791 | 4,860996179 | 0,02747047 | 0,068783602 |
| b2908 | 1326 | 0,54 | -0,563427779 | 9,114416746 | 4,858325315 | 0,02751303 | 0,068851703 |
| b2799 | 1149 | 0,54 | -0,615617013 | 7,069822349 | 4,849122192 | 0,027660205 | 0,069181384 |
| b4033 | 1545 | 0,53 | -1,207504667 | 8,216047271 | 4,843010206 | 0,0277584 | 0,069388259 |
| b4411 | 147 | 0,48 | 0,673162783 | 9,518575704 | 4,839009303 | 0,027822875 | 0,069499062 |
| b1885 | 1602 | 0,54 | 1,095064767 | 2,040057454 | 4,838336091 | 0,027833739 | 0,069499062 |
| b2825 | 564 | 0,52 | 0,83475651 | 2,781467532 | 4,82933105 | 0,027979485 | 0,069824082 |
| b1708 | 465 | 0,5 | 0,43770633 | 6,612195374 | 4,822905263 | 0,028083971 | 0,070045831 |
| b0207 | 804 | 0,46 | -0,52681492 | 5,336167602 | 4,812521137 | 0,028253681 | 0,070429921 |
| b2007 | 330 | 0,48 | -0,445086384 | 8,143147617 | 4,808039324 | 0,028327258 | 0,070574079 |
| b4118 | 909 | 0,5 | -0,754698523 | 6,653257491 | 4,804335943 | 0,028388205 | 0,070686631 |
| b2311 | 570 | 0,53 | 0,888004255 | 3,903082153 | 4,801641604 | 0,028432633 | 0,070757946 |
| b3192 | 636 | 0,52 | 0,441498379 | 7,749782935 | 4,798873445 | 0,028478353 | 0,070832396 |
| b2432 | 576 | 0,51 | 0,596316626 | 5,672621742 | 4,793242996 | 0,028571584 | 0,071024869 |
| b2565 | 729 | 0,54 | 0,506285607 | 5,004699687 | 4,791315378 | 0,028603575 | 0,071064979 |
| b1100 | 798 | 0,51 | -0,525208712 | 7,265741747 | 4,78085475 | 0,028777833 | 0,071458309 |
| b0784 | 246 | 0,56 | -0,921066961 | 6,005837475 | 4,778012761 | 0,028825367 | 0,071536708 |
| b3870 | 1410 | 0,53 | 0,852848603 | 7,636436096 | 4,772539128 | 0,028917147 | 0,071697837 |
| b0036 | 786 | 0,54 | -1,072686172 | 2,320528736 | 4,772233143 | 0,028922287 | 0,071697837 |
| b3635 | 810 | 0,54 | 0,894611042 | 4,25252992 | 4,769702785 | 0,028964826 | 0,071763598 |
| b2386 | 1248 | 0,54 | 1,382438376 | 1,911104599 | 4,757473281 | 0,029171341 | 0,072235332 |
| b2246 | 1290 | 0,53 | 0,802383899 | 3,109179362 | 4,743152028 | 0,02941513 | 0,072769794 |
| b0195 | 708 | 0,55 | 0,701547807 | 4,105864069 | 4,742887755 | 0,029419649 | 0,072769794 |
| b0732 | 2634 | 0,49 | -0,465567511 | 5,039572369 | 4,737995805 | 0,029503423 | 0,072936757 |
| b1526 | 882 | 0,54 | 0,679445963 | 4,302023752 | 4,731719386 | 0,02961127 | 0,073163016 |
| b0040 | 1515 | 0,52 | 1,161337008 | 1,749672015 | 4,72528035 | 0,029722338 | 0,073396979 |
| b0095 | 1152 | 0,54 | -0,508229192 | 10,46702141 | 4,722997095 | 0,029761826 | 0,073454022 |
| b0485 | 933 | 0,53 | -0,589637748 | 9,895052885 | 4,72050975 | 0,029804906 | 0,073494127 |
| b1947 | 366 | 0,55 | 1,710248994 | 0,82323717 | 4,720165685 | 0,029810871 | 0,073494127 |
| b0234 | 453 | 0,43 | 1,092786093 | 2,252052792 | 4,703521218 | 0,030100887 | 0,074168321 |
| b0760 | 1473 | 0,53 | 0,522578442 | 5,35574069 | 4,688701892 | 0,030361576 | 0,074769552 |
| b0797 | 1365 | 0,57 | 0,678748717 | 3,881812045 | 4,682688975 | 0,030468021 | 0,074990483 |
| b2058 | 489 | 0,54 | 1,289952415 | 1,629533626 | 4,673326553 | 0,030634535 | 0,075358939 |
| b1624 | 1041 | 0,53 | 0,402400445 | 6,930248059 | 4,671643092 | 0,030664577 | 0,075391461 |
| b1797 | 360 | 0,48 | -1,053442291 | 3,570402032 | 4,665843944 | 0,030768298 | 0,075569378 |
| b3584 | 741 | 0,49 | 0,801949692 | 3,519314988 | 4,665711945 | 0,030770664 | 0,075569378 |
| b1502 | 915 | 0,45 | 2,001208027 | 1,033743904 | 4,654977243 | 0,030963643 | 0,076001669 |
| b2082 | 219 | 0,51 | 0,655610159 | 3,908758147 | 4,64513392 | 0,031141707 | 0,076396897 |
| b2951 | 705 | 0,51 | 0,457309388 | 5,303923661 | 4,643782949 | 0,031166229 | 0,07641523 |
| b3504 | 2423 | 0,34 | 1,822917054 | 1,025264079 | 4,641196179 | 0,031213239 | 0,076488648 |
| b3233 | 399 | 0,54 | 0,423150059 | 7,530342378 | 4,639769986 | 0,031239189 | 0,076510408 |
| b2907 | 1179 | 0,6 | -0,524082871 | 7,94860104 | 4,636468756 | 0,031299343 | 0,076581465 |
| b0636 | 468 | 0,58 | 0,660993149 | 4,690775716 | 4,636302529 | 0,031302375 | 0,076581465 |
| b1553 | 498 | 0,46 | 2,248617738 | 0,773724827 | 4,633563757 | 0,031352376 | 0,076661947 |
| b1943 | 1128 | 0,55 | 1,094098855 | 2,148196808 | 4,621591983 | 0,031571923 | 0,077156684 |
| b1890 | 888 | 0,51 | 1,146020065 | 1,683543054 | 4,62057374 | 0,03159067 | 0,077160426 |
| b2503 | 2244 | 0,47 | -0,6255005 | 4,004087055 | 4,617241853 | 0,031652095 | 0,07726835 |
| b3613 | 1260 | 0,57 | 0,591275208 | 5,68899626 | 4,613289402 | 0,031725123 | 0,077404464 |
| b2364 | 936 | 0,48 | -0,467582553 | 4,85764985 | 4,60482898 | 0,031882033 | 0,07774498 |
| b4099 | 1065 | 0,63 | 1,401939381 | 1,366633107 | 4,599274872 | 0,031985482 | 0,077954829 |
| b1913 | 1833 | 0,52 | -0,370396143 | 7,34917587 | 4,596307601 | 0,032040893 | 0,078047436 |
| b1121 | 789 | 0,43 | 1,069684296 | 1,581832527 | 4,58854583 | 0,032186311 | 0,078359068 |
| b1268 | 1896 | 0,47 | -0,503742064 | 5,304484255 | 4,574815119 | 0,032445247 | 0,078946578 |
| b2759 | 483 | 0,46 | 1,138798258 | 2,198682468 | 4,568690839 | 0,03256144 | 0,079166536 |
| b0247 | 477 | 0,58 | 1,114262953 | 2,052265847 | 4,56818948 | 0,032570971 | 0,079166536 |
| b4199 | 276 | 0,46 | 1,067597491 | 2,543080388 | 4,564885325 | 0,032633859 | 0,079258584 |
| b3903 | 1260 | 0,56 | 1,083532664 | 2,12651615 | 4,564342142 | 0,032644209 | 0,079258584 |
| b1198 | 1419 | 0,5 | -0,535192108 | 6,726384559 | 4,560453623 | 0,032718406 | 0,079360526 |
| b0434 | 579 | 0,51 | 0,799522069 | 5,500640551 | 4,560285987 | 0,032721609 | 0,079360526 |
| b0497 | 4281 | 0,6 | -0,563788034 | 5,240603433 | 4,55889311 | 0,032748232 | 0,07938214 |
| b0108 | 441 | 0,53 | 2,128734319 | 0,865387284 | 4,55667237 | 0,032790725 | 0,079442179 |
| b3334 | 462 | 0,48 | 1,193657283 | 2,201191898 | 4,548180676 | 0,032953744 | 0,079793992 |
| b4559 | 174 | 0,35 | -1,344133052 | 2,478734952 | 4,546259442 | 0,032990744 | 0,07984045 |
| b0874 | 900 | 0,48 | 0,960953755 | 2,713076919 | 4,544727269 | 0,033020282 | 0,07986881 |
| b4351 | 915 | 0,51 | -0,582746353 | 6,782504376 | 4,54223611 | 0,033068367 | 0,079941976 |
| b0717 | 729 | 0,48 | 1,124869648 | 2,033404506 | 4,526611178 | 0,033371639 | 0,080631636 |
| b2616 | 1662 | 0,52 | -0,53027527 | 6,866949194 | 4,520376655 | 0,033493457 | 0,080882367 |
| b3408 | 228 | 0,49 | 1,314548599 | 4,043737156 | 4,507052904 | 0,033755354 | 0,081470918 |
| b2721 | 1710 | 0,57 | 0,764434839 | 3,085762616 | 4,504366284 | 0,033808422 | 0,081515757 |
| b0780 | 909 | 0,51 | 0,515605113 | 5,355968403 | 4,504270961 | 0,033810306 | 0,081515757 |
| b0863 | 732 | 0,5 | -0,489167708 | 9,557777359 | 4,481288135 | 0,034267884 | 0,082574546 |
| b3855 | 120 | 0,62 | -0,643898279 | 10,27783023 | 4,477760767 | 0,034338683 | 0,082700687 |
| b4520 | 219 | 0,4 | -0,903690053 | 2,752723587 | 4,465159362 | 0,034592863 | 0,083240078 |
| b3986 | 366 | 0,46 | 0,752898256 | 10,57012713 | 4,464817208 | 0,034599792 | 0,083240078 |
| b2505 | 519 | 0,47 | 1,539324938 | 1,347911103 | 4,442313804 | 0,035058703 | 0,084265008 |
| b4018 | 825 | 0,54 | -0,466116766 | 7,108586155 | 4,44208418 | 0,035063418 | 0,084265008 |
| b3475 | 588 | 0,56 | -0,57515739 | 5,026132401 | 4,432774341 | 0,035255158 | 0,084680394 |
| b0435 | 318 | 0,48 | -0,6578686 | 9,489702852 | 4,429623324 | 0,035320302 | 0,084772345 |
| b1139 | 894 | 0,37 | -0,817646701 | 4,494874674 | 4,429093526 | 0,035331267 | 0,084772345 |
| b0429 | 330 | 0,5 | 0,720823502 | 9,334364349 | 4,425467094 | 0,035406421 | 0,084907212 |
| b3716 | 468 | 0,44 | 1,018643356 | 2,524806191 | 4,424147645 | 0,035433807 | 0,084925699 |
| b3672 | 99 | 0,59 | 0,524779996 | 5,332352379 | 4,423270394 | 0,035452026 | 0,084925699 |
| b4146 | 1029 | 0,53 | 0,568871547 | 5,582373075 | 4,415204029 | 0,035620019 | 0,085257036 |
| b2991 | 342 | 0,55 | -0,847245681 | 3,742606562 | 4,414803286 | 0,035628386 | 0,085257036 |
| b1785 | 1476 | 0,38 | -1,052061398 | 3,074128515 | 4,405156679 | 0,035830433 | 0,085688483 |
| b0182 | 1149 | 0,54 | 0,422098583 | 7,216697793 | 4,404371935 | 0,035846922 | 0,085688483 |
| b3193 | 552 | 0,5 | 0,381607596 | 5,93525731 | 4,403360349 | 0,035868189 | 0,085693617 |

| | | | | | | | |
|---|---|---|---|---|---|---|---|
| b1546 | 576 | 0,48 | 1,941666161 | 0,734949631 | 4,401741673 | 0,035902247 | 0,085729287 |
| b1459 | 1054 | 0,38 | 1,203510821 | 2,001036601 | 4,398496173 | 0,035970636 | 0,085846853 |
| b4030 | 411 | 0,49 | 1,103731873 | 2,676893099 | 4,393091581 | 0,036084824 | 0,086031904 |
| b4232 | 999 | 0,51 | 0,421541577 | 8,224457415 | 4,393009358 | 0,036086564 | 0,086031904 |
| b2918 | 996 | 0,54 | 0,909464707 | 2,450913681 | 4,38940796 | 0,036162869 | 0,086163339 |
| b1887 | 504 | 0,52 | 0,809573952 | 2,589002659 | 4,388593711 | 0,036180144 | 0,086163339 |
| b3363 | 573 | 0,51 | 0,590944863 | 6,617309136 | 4,381512726 | 0,03633074 | 0,086476037 |
| b3431 | 1974 | 0,54 | -0,554022695 | 9,302414566 | 4,380357333 | 0,036355375 | 0,086488742 |
| b2125 | 720 | 0,49 | 0,53482381 | 5,091174412 | 4,377325819 | 0,036420095 | 0,086596745 |
| b3801 | 1656 | 0,53 | -0,553965729 | 4,487231879 | 4,370941866 | 0,03655678 | 0,086875657 |
| b2424 | 834 | 0,56 | 0,955252263 | 2,409113277 | 4,369704457 | 0,036583336 | 0,086892694 |
| b3780 | 1266 | 0,53 | 0,416968903 | 8,081851319 | 4,360031916 | 0,036791616 | 0,087341114 |
| b3326 | 1482 | 0,52 | 0,879860413 | 2,62782854 | 4,358261723 | 0,036829868 | 0,087385637 |
| b0815 | 1584 | 0,48 | 0,63728013 | 3,977569718 | 4,357196289 | 0,036852911 | 0,087394046 |
| b3047 | 750 | 0,39 | 1,210645654 | 1,578456176 | 4,351994954 | 0,036965621 | 0,087614972 |
| b1610 | 930 | 0,51 | 0,545556199 | 5,449179483 | 4,340897328 | 0,037207309 | 0,088141204 |
| b3018 | 738 | 0,49 | 0,42737013 | 5,660171806 | 4,337597491 | 0,037279493 | 0,08826555 |
| b1373 | 576 | 0,48 | 1,754426636 | 1,105249358 | 4,33262192 | 0,037388611 | 0,088431816 |
| b1712 | 300 | 0,5 | 0,511750071 | 9,806352034 | 4,332596133 | 0,037389177 | 0,088431816 |
| b0684 | 531 | 0,49 | 0,477794642 | 7,375200781 | 4,32813882 | 0,037487215 | 0,088616929 |
| b2035 | 1167 | 0,32 | -0,556994878 | 6,869571551 | 4,322500717 | 0,03761161 | 0,08886412 |
| b1904 | 324 | 0,5 | 0,993522662 | 1,989886975 | 4,317469083 | 0,037722989 | 0,089080315 |
| b1453 | 1500 | 0,52 | 0,775012041 | 3,009282494 | 4,309716211 | 0,037895283 | 0,089440051 |
| b0391 | 285 | 0,52 | 0,505598615 | 6,300787571 | 4,307951719 | 0,037934611 | 0,08948575 |
| b2909 | 579 | 0,51 | 0,365578665 | 7,665449814 | 4,30546956 | 0,037990006 | 0,089563184 |
| b3397 | 561 | 0,51 | 0,791452524 | 5,293588203 | 4,30469088 | 0,038007402 | 0,089563184 |
| b2563 | 381 | 0,51 | -0,599574545 | 4,677112769 | 4,294438243 | 0,038237226 | 0,090057408 |
| b3898 | 1071 | 0,53 | 0,750884301 | 3,032912092 | 4,291050013 | 0,038313497 | 0,09018965 |
| b4556 | 815 | 0,53 | 0,82319239 | 2,474581554 | 4,288085384 | 0,038380363 | 0,090299626 |
| b2778 | 873 | 0,41 | 1,683936588 | 1,038694837 | 4,286017696 | 0,038427071 | 0,090362085 |
| b1081 | 942 | 0,55 | 1,063066195 | 1,967110502 | 4,283732348 | 0,038478766 | 0,090436197 |
| b2362 | 363 | 0,47 | 1,743734722 | 0,773057423 | 4,280920832 | 0,038542462 | 0,090508728 |
| b2185 | 285 | 0,51 | 0,777200621 | 7,816699767 | 4,280587842 | 0,038550014 | 0,090508728 |
| b2057 | 1218 | 0,54 | 1,014981006 | 1,985961821 | 4,279162669 | 0,038582351 | 0,090537223 |
| b3616 | 1026 | 0,51 | -0,533009602 | 10,19699539 | 4,277372692 | 0,038623005 | 0,090585196 |
| b0444 | 696 | 0,52 | 0,428077742 | 5,692438786 | 4,274931594 | 0,038678521 | 0,090667955 |
| b1753 | 549 | 0,56 | -0,777710303 | 4,266902001 | 4,270375948 | 0,038782349 | 0,090829791 |
| b2494 | 1464 | 0,54 | 0,400957901 | 7,159382956 | 4,269560504 | 0,038800965 | 0,090829791 |
| b3517 | 1401 | 0,54 | -1,121168137 | 11,22477758 | 4,269236866 | 0,038808356 | 0,090829791 |
| b1491 | 1320 | 0,52 | 0,436260648 | 5,554174025 | 4,267280848 | 0,038853057 | 0,090886952 |
| b4106 | 789 | 0,59 | 2,063006074 | 0,994659239 | 4,259410883 | 0,039033456 | 0,091261319 |
| b0382 | 261 | 0,46 | -0,40460541 | 7,120889489 | 4,246373775 | 0,039334234 | 0,091916599 |
| b1548 | 528 | 0,53 | 1,672221644 | 1,083363538 | 4,242391183 | 0,0394266 | 0,092084431 |
| b3953 | 342 | 0,56 | 0,932929872 | 2,517232763 | 4,233194659 | 0,039640761 | 0,092536401 |
| b3643 | 716 | 0,57 | 0,741677029 | 4,645989616 | 4,231757455 | 0,039674339 | 0,092566574 |
| b2160 | 1089 | 0,52 | -0,457790396 | 5,620788398 | 4,228939767 | 0,039740258 | 0,092672131 |
| b0697 | 2049 | 0,56 | 1,229664567 | 6,25408794 | 4,222798538 | 0,039884327 | 0,092959726 |
| b1941 | 1374 | 0,59 | 1,190067289 | 1,642457166 | 4,213784583 | 0,040096782 | 0,09340633 |
| b3382 | 363 | 0,52 | 0,736870386 | 3,162096862 | 4,207556572 | 0,040244268 | 0,093691068 |
| b2273 | 717 | 0,39 | 1,362595011 | 1,288695704 | 4,206859072 | 0,04026082 | 0,093691068 |
| b2888 | 1449 | 0,49 | 0,929751747 | 2,247170955 | 4,197463929 | 0,040484481 | 0,09416266 |
| b0052 | 990 | 0,56 | 0,415853265 | 6,790145931 | 4,194769913 | 0,040548855 | 0,09426347 |
| b2604 | 1227 | 0,48 | -0,488072266 | 4,552406115 | 4,192811554 | 0,040595718 | 0,094323489 |
| b4053 | 1080 | 0,56 | 0,442401538 | 5,926041721 | 4,190295938 | 0,040656 | 0,094414606 |
| b0981 | 2181 | 0,51 | 0,458291395 | 5,295305925 | 4,185519512 | 0,040770715 | 0,094631976 |
| b1329 | 1614 | 0,48 | -0,491469711 | 7,619852679 | 4,176435206 | 0,040989832 | 0,095091318 |
| b3166 | 945 | 0,52 | 0,424006956 | 7,203381778 | 4,169049261 | 0,041168895 | 0,095457314 |
| b0983 | 1140 | 0,51 | 0,719079582 | 3,434822701 | 4,161027582 | 0,041364302 | 0,095860807 |
| b0849 | 258 | 0,45 | 0,581289388 | 4,067772906 | 4,15868075 | 0,041421654 | 0,095944111 |
| b3673 | 1185 | 0,57 | 0,472291247 | 5,866715696 | 4,156472446 | 0,041475698 | 0,096019668 |
| b1672 | 648 | 0,52 | -0,649100146 | 3,453231408 | 4,15080038 | 0,041614849 | 0,096292077 |
| b0543 | 333 | 0,41 | 0,94466416 | 2,808758606 | 4,149129026 | 0,041655945 | 0,096337434 |
| b4600 | 21837 | 0,47 | 0,794328377 | 2,842117325 | 4,143607675 | 0,041792011 | 0,096602266 |
| b0703 | 1521 | 0,55 | 1,152588097 | 2,196876676 | 4,13488076 | 0,042008027 | 0,097051534 |
| b1886 | 1662 | 0,53 | 1,000125937 | 2,312137749 | 4,132401924 | 0,042069599 | 0,09714371 |
| b2805 | 732 | 0,48 | 0,478754835 | 6,581444407 | 4,130569252 | 0,042115182 | 0,09719889 |
| b1316 | 2268 | 0,54 | 0,787857326 | 3,463349593 | 4,117138952 | 0,042450813 | 0,097923079 |
| b3058 | 369 | 0,51 | 0,943489256 | 2,789759644 | 4,105127379 | 0,042753371 | 0,098570272 |
| b2832 | 714 | 0,5 | 0,695548613 | 4,229446908 | 4,102605591 | 0,042817179 | 0,098666631 |
| b2935 | 1992 | 0,56 | 0,637341086 | 10,18706215 | 4,096322936 | 0,042976585 | 0,098983069 |
| b4297 | 1968 | 0,55 | -0,517532711 | 5,869694571 | 4,094944922 | 0,043011631 | 0,099012908 |
| b3535 | 189 | 0,46 | -0,788860393 | 4,291221191 | 4,092853352 | 0,043064883 | 0,099084603 |
| b1744 | 969 | 0,53 | 0,834298656 | 6,438979029 | 4,085755502 | 0,043246114 | 0,099412499 |
| b1835 | 1440 | 0,52 | 0,716106985 | 4,478386982 | 4,085535 | 0,043251757 | 0,099412499 |
| b3410 | 237 | 0,55 | 0,827687802 | 3,545213188 | 4,084048878 | 0,043289809 | 0,099448961 |
| b3943 | 906 | 0,54 | -0,692608152 | 3,549493581 | 4,076850072 | 0,043474633 | 0,099781544 |
| b3618 | 858 | 0,37 | 0,604424564 | 5,521128561 | 4,076676216 | 0,043479107 | 0,099781544 |
| b4182 | 699 | 0,51 | 1,200461479 | 1,582053066 | 4,074982988 | 0,043522704 | 0,099830481 |
| b0509 | 879 | 0,51 | -1,681745432 | 4,270595559 | 4,064910423 | 0,043783007 | 0,100376183 |
| b4487 | 330 | 0,56 | 0,745152076 | 3,305343833 | 4,052845663 | 0,044096951 | 0,101044242 |
| b2326 | 549 | 0,5 | 0,560019729 | 4,81699714 | 4,050991254 | 0,044145416 | 0,101103604 |
| b4209 | 663 | 0,53 | -1,199210776 | 6,012966398 | 4,049195291 | 0,044192406 | 0,101159532 |
| b2533 | 804 | 0,54 | 0,731795252 | 5,362521213 | 4,045239953 | 0,044296081 | 0,101345092 |
| b2389 | 1257 | 0,54 | -0,457957319 | 5,616625048 | 4,04383508 | 0,044332966 | 0,101377732 |
| b3993 | 636 | 0,58 | 1,130368109 | 2,663498803 | 4,041906399 | 0,044383657 | 0,101441892 |
| b4139 | 1437 | 0,49 | -0,797747802 | 12,53720448 | 4,039465558 | 0,044447896 | 0,101536936 |
| b4642 | 129 | 0,53 | 1,836115669 | 2,28658406 | 4,038198219 | 0,044481289 | 0,101549195 |
| b1701 | 1647 | 0,5 | 0,774549585 | 3,150743986 | 4,0375425 | 0,044498576 | 0,101549195 |
| b3507 | 531 | 0,4 | -0,563791655 | 5,67324816 | 4,034414273 | 0,044581148 | 0,101685856 |
| b4501 | 201 | 0,44 | 0,972576386 | 2,296134933 | 4,033369368 | 0,044608765 | 0,101697094 |
| b1676 | 1413 | 0,5 | -0,541701006 | 10,12461437 | 4,028654214 | 0,044733612 | 0,101929867 |
| b2906 | 1203 | 0,55 | -0,394817263 | 6,84529453 | 4,027099255 | 0,044774864 | 0,101972023 |
| b0763 | 774 | 0,51 | -0,479580785 | 5,733870365 | 4,025084504 | 0,044828374 | 0,102042038 |
| b1486 | 1023 | 0,55 | 1,169744968 | 3,913628104 | 4,023494431 | 0,044870652 | 0,102086429 |

| | | | | | | | |
|---|---|---|---|---|---|---|---|
| b4399 | 1425 | 0,54 | 0,511331907 | 4,893447193 | 4,022275113 | 0,044903101 | 0,102108422 |
| b4281 | 231 | 0,48 | 1,201185499 | 1,590560044 | 4,01688423 | 0,045046862 | 0,102358861 |
| b4000 | 273 | 0,48 | 0,486612349 | 9,165310407 | 4,016433272 | 0,04505891 | 0,102358861 |
| b3795 | 1386 | 0,53 | 0,563632593 | 5,432056495 | 4,013269453 | 0,04514353 | 0,102414971 |
| b0384 | 321 | 0,48 | 0,43590007 | 6,682716443 | 4,013033552 | 0,045149846 | 0,102414971 |
| b0607 | 429 | 0,5 | -0,710855682 | 9,781436363 | 4,012947112 | 0,04515216 | 0,102414971 |
| b1844 | 663 | 0,52 | 0,453004847 | 5,491596025 | 4,009984531 | 0,045231566 | 0,102543186 |
| b2359 | 363 | 0,5 | 1,522425663 | 1,338378334 | 4,004216415 | 0,04538659 | 0,102842616 |
| b0674 | 1665 | 0,52 | 0,510569226 | 7,052428135 | 3,999905348 | 0,045502819 | 0,103053883 |
| b2599 | 1161 | 0,49 | -0,489920482 | 6,609499983 | 3,994293872 | 0,045654579 | 0,103345364 |
| b3752 | 930 | 0,52 | 0,519350061 | 7,279781493 | 3,981147724 | 0,046012201 | 0,104102315 |
| b4218 | 1344 | 0,51 | -0,489348484 | 5,93780125 | 3,978104368 | 0,046095411 | 0,104237959 |
| b2275 | 849 | 0,43 | 0,772005111 | 3,496811627 | 3,973072096 | 0,04623335 | 0,104497163 |
| b0973 | 1794 | 0,57 | -0,854448168 | 6,140751583 | 3,971005583 | 0,046290121 | 0,104551811 |
| b0918 | 747 | 0,53 | 0,334742384 | 7,342352066 | 3,970494143 | 0,046304182 | 0,104551811 |
| b4571 | 1994 | 0,35 | -0,669843688 | 6,806000962 | 3,958826683 | 0,046626192 | 0,105225878 |
| b3657 | 1383 | 0,49 | 0,742091411 | 2,948961117 | 3,95322073 | 0,04678175 | 0,105471628 |
| b1368 | 933 | 0,46 | -0,726797834 | 3,408512001 | 3,953206321 | 0,04678215 | 0,105471628 |
| b1520 | 915 | 0,48 | -0,478706139 | 5,508753547 | 3,951102481 | 0,04684067 | 0,10555047 |
| b0707 | 510 | 0,5 | -0,485457413 | 5,586471923 | 3,949807026 | 0,046876743 | 0,105578675 |
| b1988 | 918 | 0,48 | 0,680795134 | 3,444126523 | 3,946821726 | 0,046959982 | 0,105713029 |
| b1809 | 345 | 0,52 | -0,479288804 | 6,735421918 | 3,944859961 | 0,047014767 | 0,105783225 |
| b0172 | 558 | 0,51 | 0,41700486 | 9,054468118 | 3,942961188 | 0,047067856 | 0,10584954 |
| b0098 | 2706 | 0,52 | -0,437479459 | 9,495341106 | 3,941494021 | 0,047108922 | 0,105872649 |
| b1618 | 591 | 0,51 | -0,466181824 | 6,141583858 | 3,940906533 | 0,047125376 | 0,105872649 |
| b2927 | 1020 | 0,51 | 0,398181025 | 8,116773748 | 3,939588449 | 0,047162314 | 0,105902551 |
| b1242 | 648 | 0,48 | 0,547730993 | 4,145819002 | 3,935959139 | 0,047264181 | 0,106078147 |
| b0929 | 1089 | 0,48 | -0,737417253 | 10,72841959 | 3,934895792 | 0,047294071 | 0,106092104 |
| b1615 | 1266 | 0,47 | 1,417016438 | 1,972907065 | 3,923159853 | 0,047625285 | 0,10675897 |
| b0726 | 2802 | 0,56 | 0,850456471 | 12,25930049 | 3,922676196 | 0,047638987 | 0,10675897 |
| b3455 | 768 | 0,55 | 0,997389988 | 2,424839191 | 3,91939519 | 0,047732049 | 0,106914066 |
| b1179 | 294 | 0,44 | 0,588511085 | 4,678361193 | 3,916145244 | 0,04782442 | 0,107067458 |
| b3608 | 1020 | 0,57 | 0,412303947 | 8,886401788 | 3,898834322 | 0,048319623 | 0,108090707 |
| b2148 | 1011 | 0,5 | 1,71259458 | 7,3334746 | 3,898483519 | 0,048329714 | 0,108090707 |
| b2543 | 423 | 0,5 | 0,486454705 | 6,533974258 | 3,896826611 | 0,048377405 | 0,108143407 |
| b1884 | 861 | 0,54 | 1,228997582 | 1,747804196 | 3,895693752 | 0,048410042 | 0,108162416 |
| b3691 | 1293 | 0,53 | 0,959718001 | 2,80666878 | 3,892985535 | 0,048488156 | 0,108272091 |
| b0437 | 624 | 0,51 | 0,395272994 | 8,855515035 | 3,892317576 | 0,048507443 | 0,108272091 |
| b0705 | 1137 | 0,41 | 2,060946747 | 1,019693711 | 3,886015628 | 0,048689805 | 0,108625041 |
| b2156 | 1470 | 0,53 | -0,353679259 | 7,009870233 | 3,880459418 | 0,048851188 | 0,108930858 |
| b3712 | 750 | 0,52 | -0,457998579 | 5,540747598 | 3,879329222 | 0,048884084 | 0,108950008 |
| b2904 | 390 | 0,53 | 0,450287498 | 10,13119397 | 3,877995666 | 0,04892293 | 0,108982391 |
| b4699 | 122 | 0,44 | 1,820090205 | 0,939721141 | 3,873524941 | 0,049053396 | 0,109218739 |
| b1745 | 1344 | 0,56 | 0,932623379 | 7,332819027 | 3,86536494 | 0,049292473 | 0,109654099 |
| b0521 | 894 | 0,57 | 0,834263157 | 2,565768499 | 3,865181546 | 0,04929786 | 0,109654099 |
| b3710 | 1176 | 0,53 | 0,890067063 | 2,809653723 | 3,861796277 | 0,049397416 | 0,109821041 |
| b0640 | 1032 | 0,52 | 0,444163591 | 6,582177994 | 3,859702449 | 0,049459099 | 0,10990366 |
| b3883 | 897 | 0,56 | -0,667795689 | 4,270384512 | 3,85879707 | 0,049485796 | 0,109908492 |
| b3198 | 567 | 0,53 | 0,388300477 | 6,550565817 | 3,851171324 | 0,049711263 | 0,110354571 |
| b2487 | 1668 | 0,54 | -0,55629519 | 4,263007946 | 3,85012703 | 0,049742223 | 0,110368636 |
| b1987 | 951 | 0,48 | 0,672584631 | 3,313025942 | 3,848634005 | 0,049786523 | 0,110412269 |
| b1942 | 444 | 0,52 | 1,816849005 | 0,891939326 | 3,845831523 | 0,049869788 | 0,11054223 |
| b3981 | 384 | 0,55 | 0,438282311 | 7,355334469 | 3,843791401 | 0,049930495 | 0,110622085 |
| b1439 | 1407 | 0,51 | 0,4971624 | 5,265200674 | 3,84049535 | 0,050028739 | 0,110784984 |
| b3674 | 498 | 0,54 | -0,480204089 | 4,835671786 | 3,837168955 | 0,050128095 | 0,110950183 |
| b0936 | 960 | 0,52 | 1,309755382 | 1,475145249 | 3,834919198 | 0,05019541 | 0,110990637 |
| b1430 | 594 | 0,46 | -0,485695063 | 5,33038084 | 3,834902864 | 0,0501959 | 0,110990637 |
| b2597 | 342 | 0,46 | -0,685617431 | 12,18040722 | 3,832355668 | 0,050272231 | 0,111104606 |
| b0716 | 1062 | 0,45 | -0,736676701 | 3,843140175 | 3,831385447 | 0,050301338 | 0,111114144 |
| b1099 | 1005 | 0,54 | 0,403317065 | 5,585013045 | 3,827621628 | 0,050414422 | 0,111309084 |
| b1029 | 987 | 0,41 | 1,813819141 | 1,133733513 | 3,824090021 | 0,050520774 | 0,111488975 |
| b1254 | 540 | 0,46 | 0,467682576 | 5,084281893 | 3,808741052 | 0,050985759 | 0,112459731 |
| b0544 | 1527 | 0,36 | 1,15950506 | 1,755127978 | 3,804879603 | 0,05110345 | 0,112624057 |
| b2633 | 822 | 0,56 | 1,018798168 | 2,35443237 | 3,804648054 | 0,051110516 | 0,112624057 |
| b2268 | 918 | 0,51 | 0,63896836 | 4,230443548 | 3,803157233 | 0,051156037 | 0,112668972 |
| b3022 | 297 | 0,4 | 0,456018693 | 5,589187515 | 3,793693283 | 0,051446014 | 0,113230315 |
| b3614 | 960 | 0,53 | 0,672287778 | 4,43995582 | 3,792501251 | 0,051482662 | 0,113230315 |
| b1400 | 591 | 0,51 | 0,374589177 | 6,636538129 | 3,792370008 | 0,051486698 | 0,113230315 |
| b4060 | 282 | 0,52 | 0,907920447 | 2,768161221 | 3,784731704 | 0,051722202 | 0,113692451 |
| b1043 | 333 | 0,43 | 2,034627136 | 0,762358857 | 3,782261297 | 0,051798613 | 0,113804599 |
| b4023 | 273 | 0,49 | -0,576694438 | 3,637608072 | 3,777138132 | 0,051957456 | 0,114097657 |
| b3141 | 756 | 0,5 | 1,112638484 | 1,626386454 | 3,776015321 | 0,051992337 | 0,114118343 |
| b2558 | 1557 | 0,5 | 0,619296206 | 3,946788368 | 3,768504521 | 0,052226307 | 0,114544066 |
| b4396 | 870 | 0,53 | -0,472866919 | 9,140436806 | 3,767735247 | 0,052250334 | 0,114544066 |
| b3355 | 870 | 0,53 | -0,419279161 | 6,35986078 | 3,767330937 | 0,052262966 | 0,114544066 |
| b3880 | 1242 | 0,53 | -0,591087003 | 4,27438605 | 3,759166019 | 0,052518767 | 0,115048443 |
| b1184 | 1269 | 0,51 | -0,582833387 | 4,06683399 | 3,757249232 | 0,052579011 | 0,115091186 |
| b3337 | 195 | 0,46 | 1,804149909 | 1,890298166 | 3,756911392 | 0,052589636 | 0,115091186 |
| b3655 | 1710 | 0,54 | -0,408985316 | 7,174906153 | 3,750097593 | 0,052804428 | 0,115504855 |
| b3266 | 3105 | 0,52 | -0,483678121 | 4,557804282 | 3,747268932 | 0,052893869 | 0,115644059 |
| b0305 | 855 | 0,51 | -0,644551947 | 4,149213429 | 3,741807843 | 0,053067 | 0,115966013 |
| b2281 | 543 | 0,54 | 0,849340614 | 8,847788552 | 3,726845145 | 0,053544438 | 0,116952324 |
| b2898 | 981 | 0,54 | -0,359905546 | 8,690800285 | 3,723953178 | 0,05363724 | 0,117097958 |
| b3928 | 246 | 0,48 | 0,400772982 | 7,635078861 | 3,714874453 | 0,05392968 | 0,117679078 |
| b0719 | 567 | 0,47 | 1,360426135 | 1,1797869 | 3,712410746 | 0,054009331 | 0,117795535 |
| b1940 | 687 | 0,57 | 1,057364599 | 1,859761388 | 3,708531419 | 0,054135002 | 0,118012198 |
| b2119 | 1089 | 0,54 | 1,449641235 | 1,220187778 | 3,706741966 | 0,054193076 | 0,118081364 |
| b2423 | 876 | 0,57 | 0,842673192 | 2,588079667 | 3,698433623 | 0,054463575 | 0,118591667 |
| b0934 | 792 | 0,56 | 1,231635822 | 1,563875088 | 3,697924504 | 0,054480197 | 0,118591667 |
| b0448 | 1773 | 0,54 | 0,503467882 | 5,067834372 | 3,68710829 | 0,054834606 | 0,119289145 |
| b3395 | 780 | 0,58 | 0,64534323 | 3,234208399 | 3,686196171 | 0,054864605 | 0,119289145 |
| b1259 | 180 | 0,51 | 0,918128347 | 4,896170921 | 3,685714331 | 0,054880459 | 0,119289145 |
| b4544 | 336 | 0,54 | -0,665918959 | 3,824677079 | 3,684218496 | 0,054929708 | 0,119338318 |
| b1567 | 183 | 0,44 | 2,00493692 | 0,877802184 | 3,683375051 | 0,054957498 | 0,119340846 |

| | | | | | | | |
|---|---|---|---|---|---|---|---|
| b2129 | 927 | 0,55 | 0,446782766 | 5,766062518 | 3,682147444 | 0,054997972 | 0,119370902 |
| b3863 | 2787 | 0,52 | -0,418360423 | 9,319377827 | 3,675143106 | 0,055229512 | 0,119815428 |
| b1485 | 897 | 0,53 | 0,982661365 | 3,844517201 | 3,673058098 | 0,055298635 | 0,119907345 |
| b1688 | 1113 | 0,55 | -0,395855004 | 7,020044797 | 3,667456645 | 0,055484791 | 0,120200776 |
| b3005 | 426 | 0,5 | 0,813559749 | 4,384621835 | 3,667372387 | 0,055487596 | 0,120200776 |
| b1775 | 1380 | 0,49 | 0,967766488 | 2,615282908 | 3,657159507 | 0,055828737 | 0,120820248 |
| b0276 | 828 | 0,49 | -0,798034841 | 4,843274094 | 3,656652635 | 0,055845726 | 0,120820248 |
| b1945 | 1005 | 0,53 | 0,793138588 | 2,647653404 | 3,65612981 | 0,055863255 | 0,120820248 |
| b0899 | 1431 | 0,47 | -0,654709421 | 4,650734981 | 3,6555892 | 0,055881387 | 0,120820248 |
| b4398 | 690 | 0,55 | 0,470026775 | 5,431608574 | 3,654561355 | 0,055915877 | 0,120836529 |
| b3992 | 756 | 0,58 | 0,949802183 | 2,812825048 | 3,652248387 | 0,055993574 | 0,12094612 |
| b4385 | 1332 | 0,54 | -0,461339627 | 6,291743524 | 3,64836048 | 0,056124434 | 0,121170383 |
| b2458 | 1017 | 0,58 | 0,610594658 | 3,776338054 | 3,629796213 | 0,056753765 | 0,12247009 |
| b2367 | 1539 | 0,42 | 1,487095584 | 1,38208051 | 3,625415232 | 0,056903371 | 0,122733834 |
| b1907 | 1212 | 0,51 | 0,68099182 | 4,106629579 | 3,62184463 | 0,057025612 | 0,122928988 |
| b4660 | 1607 | 0,35 | 1,346782668 | 1,593296981 | 3,620646482 | 0,057066694 | 0,122928988 |
| b2566 | 906 | 0,52 | 0,410862774 | 7,322221182 | 3,620371349 | 0,057076132 | 0,122928988 |
| b4269 | 1020 | 0,56 | -0,527537288 | 8,088073526 | 3,609618019 | 0,057446313 | 0,123666846 |
| b2807 | 396 | 0,55 | 0,517686306 | 4,521886802 | 3,605224331 | 0,057598297 | 0,123905681 |
| b2289 | 939 | 0,5 | -0,405178194 | 7,506484853 | 3,604336953 | 0,057629045 | 0,123905681 |
| b0115 | 1893 | 0,55 | -0,63036895 | 9,768464933 | 3,604015348 | 0,057640193 | 0,123905681 |
| b3897 | 1749 | 0,5 | 0,87281027 | 2,438093649 | 3,601542891 | 0,057725973 | 0,12403059 |
| b2443 | 642 | 0,44 | 0,599873085 | 3,908630343 | 3,595551515 | 0,057934402 | 0,124418779 |
| b2300 | 555 | 0,52 | 0,42040968 | 6,684653911 | 3,588134968 | 0,058193519 | 0,124915398 |
| b2507 | 1578 | 0,54 | 0,691040397 | 9,152003868 | 3,584854182 | 0,058308534 | 0,125102369 |
| b4336 | 1281 | 0,53 | -0,446684658 | 6,183633736 | 3,580621112 | 0,058457291 | 0,125331724 |
| b4174 | 1260 | 0,56 | 0,430942629 | 9,311388338 | 3,580221342 | 0,05847136 | 0,125331724 |
| b2870 | 1191 | 0,5 | -0,956800262 | 2,614821791 | 3,575317286 | 0,058644245 | 0,125642211 |
| b4088 | 936 | 0,49 | -0,585720304 | 4,187564587 | 3,562259979 | 0,059107214 | 0,126518149 |
| b2839 | 936 | 0,53 | 0,492670746 | 4,375725626 | 3,562194362 | 0,05910955 | 0,126518149 |
| b0577 | 1248 | 0,48 | 0,42964147 | 5,575754963 | 3,558043388 | 0,059257549 | 0,126774384 |
| b0452 | 861 | 0,52 | 0,511850653 | 6,06093585 | 3,548467594 | 0,059600469 | 0,127447186 |
| b3028 | 582 | 0,51 | 0,506720355 | 5,639000984 | 3,545215285 | 0,059717417 | 0,127636368 |
| b3822 | 1830 | 0,55 | 0,63704422 | 5,453485763 | 3,531469083 | 0,060214414 | 0,128613218 |
| b0769 | 1053 | 0,49 | 1,954231971 | 0,689297873 | 3,530989053 | 0,060231848 | 0,128613218 |
| b0446 | 819 | 0,51 | 0,481335159 | 4,035998442 | 3,530008869 | 0,060267466 | 0,128627991 |
| b0841 | 597 | 0,52 | 0,691330612 | 3,921444437 | 3,523732911 | 0,06049605 | 0,1290544 |
| b2011 | 1428 | 0,5 | -0,435993389 | 6,653039394 | 3,520647409 | 0,060608768 | 0,129233349 |
| b0916 | 1233 | 0,52 | 0,494572697 | 3,938588854 | 3,517045127 | 0,060740648 | 0,129405108 |
| b2492 | 849 | 0,51 | 0,563563276 | 3,482384868 | 3,516303118 | 0,060767851 | 0,129405108 |
| b2791 | 783 | 0,54 | 0,583160021 | 4,362620792 | 3,516082624 | 0,060775938 | 0,129405108 |
| b4012 | 444 | 0,55 | -0,593772507 | 4,570100401 | 3,510668404 | 0,060974851 | 0,129766991 |
| b4463 | 1455 | 0,51 | 0,764289222 | 3,133964861 | 3,508703502 | 0,061047211 | 0,129859326 |
| b1681 | 1272 | 0,53 | -0,588158461 | 8,199580767 | 3,506629234 | 0,061123698 | 0,129960348 |
| b3064 | 1014 | 0,58 | 0,360304741 | 6,121239794 | 3,502815111 | 0,061264606 | 0,130188282 |
| b0495 | 687 | 0,57 | 0,417628026 | 4,977700372 | 3,502155909 | 0,061288995 | 0,130188282 |
| b1218 | 696 | 0,55 | -0,380643168 | 6,297217074 | 3,497961433 | 0,06144442 | 0,130456604 |
| b4372 | 414 | 0,58 | 0,696062947 | 4,540394213 | 3,485757716 | 0,061899018 | 0,131344771 |
| b3731 | 420 | 0,55 | 0,718969879 | 9,713664534 | 3,485093204 | 0,061923874 | 0,131344771 |
| b2945 | 708 | 0,55 | -0,801189324 | 2,959342047 | 3,484377498 | 0,061950657 | 0,131344771 |
| b1527 | 1116 | 0,4 | 1,572331043 | 1,143945064 | 3,482931865 | 0,062004793 | 0,131397391 |
| b4068 | 315 | 0,49 | 1,125236379 | 5,844273956 | 3,47665645 | 0,062240378 | 0,131834299 |
| b1279 | 309 | 0,5 | -0,46823119 | 6,006852278 | 3,475128078 | 0,062297899 | 0,131893805 |
| b2720 | 543 | 0,56 | 0,990163395 | 1,74041694 | 3,47263286 | 0,06239193 | 0,132022342 |
| b4313 | 597 | 0,47 | 1,00325266 | 2,119228719 | 3,471954368 | 0,062417524 | 0,132022342 |
| b2786 | 2757 | 0,5 | -0,325386137 | 6,499041742 | 3,469372277 | 0,06251503 | 0,132166209 |
| b2647 | 4581 | 0,49 | -0,365946604 | 6,885114133 | 3,464929762 | 0,06268317 | 0,132421928 |
| b2635 | 237 | 0,53 | 1,51195278 | 0,87377153 | 3,464615651 | 0,062695076 | 0,132421928 |
| b0563 | 183 | 0,47 | 1,249984827 | 1,611628608 | 3,462234669 | 0,062785408 | 0,132550258 |
| b4371 | 1032 | 0,56 | 0,442245939 | 5,462570592 | 3,45473151 | 0,063070977 | 0,13305273 |
| b4246 | 135 | 0,5 | 1,933719139 | 0,884366313 | 3,454054707 | 0,063096805 | 0,13305273 |
| b0939 | 702 | 0,45 | 1,179340891 | 1,231353096 | 3,453219088 | 0,063128708 | 0,13305273 |
| b3081 | 2019 | 0,57 | 0,585730514 | 6,682154619 | 3,452866898 | 0,063142159 | 0,13305273 |
| b0830 | 1539 | 0,52 | -0,368782213 | 6,96525996 | 3,451659791 | 0,063188286 | 0,133082755 |
| b2671 | 345 | 0,47 | -0,597485996 | 4,311460718 | 3,450374016 | 0,063237458 | 0,133082755 |
| b1023 | 2019 | 0,44 | 0,861780448 | 3,492153192 | 3,450164218 | 0,063245486 | 0,133082755 |
| b0392 | 342 | 0,47 | 0,909713525 | 2,930667401 | 3,448387816 | 0,063313498 | 0,133159788 |
| b1868 | 819 | 0,51 | 0,498421562 | 4,870941767 | 3,447656646 | 0,063341514 | 0,133159788 |
| b0958 | 510 | 0,48 | -0,488727638 | 6,865760306 | 3,437315333 | 0,063739186 | 0,133932973 |
| b4554 | 210 | 0,49 | -0,686923837 | 7,928247734 | 3,434584723 | 0,063844634 | 0,134037316 |
| b1635 | 606 | 0,5 | 0,427759642 | 7,973063145 | 3,434125381 | 0,063862391 | 0,134037316 |
| b1850 | 642 | 0,56 | -0,418344433 | 7,84983348 | 3,433707221 | 0,06387856 | 0,134037316 |
| b3380 | 1227 | 0,54 | 0,801656027 | 2,466338641 | 3,430871673 | 0,06398832 | 0,134204796 |
| b0351 | 951 | 0,54 | 0,655147056 | 3,129156541 | 3,426384202 | 0,064162434 | 0,13450703 |
| b2780 | 1638 | 0,52 | 0,408517723 | 9,48098391 | 3,423485799 | 0,064275161 | 0,134680351 |
| b4298 | 906 | 0,53 | -0,44194867 | 4,438080146 | 3,422037522 | 0,064331568 | 0,134735554 |
| b1617 | 1812 | 0,52 | -0,690599458 | 4,330346808 | 3,41969024 | 0,0644231 | 0,134864239 |
| b0498 | 369 | 0,31 | 1,067681986 | 1,739585229 | 3,417077719 | 0,06452514 | 0,135014788 |
| b1209 | 624 | 0,51 | 0,543978833 | 6,245835216 | 3,398423753 | 0,065258752 | 0,136486106 |
| b0783 | 486 | 0,56 | -0,73806789 | 7,583903237 | 3,393583937 | 0,06545054 | 0,136823377 |
| b4473 | 1125 | 0,51 | -0,403684135 | 7,024444683 | 3,387715103 | 0,065683913 | 0,137247225 |
| b1892 | 351 | 0,46 | 0,742025229 | 3,358332947 | 3,386444274 | 0,065734564 | 0,137289057 |
| b2629 | 264 | 0,45 | 0,970637143 | 1,792262118 | 3,383426545 | 0,065855008 | 0,137476548 |
| b3997 | 1065 | 0,56 | 0,338548883 | 7,203163773 | 3,381137904 | 0,06594651 | 0,13751459 |
| b2132 | 2298 | 0,54 | 0,358975353 | 7,20780943 | 3,380863389 | 0,065957495 | 0,13751459 |
| b1644 | 858 | 0,5 | 0,85647546 | 3,219009925 | 3,380668941 | 0,065965276 | 0,13751459 |
| b3886 | 873 | 0,51 | -0,339293356 | 7,229436019 | 3,378237873 | 0,066062651 | 0,137586918 |
| b2071 | 462 | 0,41 | 1,12264473 | 1,32547886 | 3,377942234 | 0,066074503 | 0,137586918 |
| b2048 | 1371 | 0,55 | 0,932279246 | 2,811811521 | 3,377128076 | 0,066107154 | 0,137586918 |
| b3444 | 276 | 0,53 | -0,585152406 | 3,891240537 | 3,376739023 | 0,066122763 | 0,137586918 |
| b1859 | 786 | 0,52 | -0,398675358 | 4,663660784 | 3,365196483 | 0,066587641 | 0,138489933 |
| b0710 | 744 | 0,53 | 0,413724319 | 8,042061881 | 3,36147754 | 0,066738164 | 0,138738614 |
| b3292 | 426 | 0,49 | 0,452747059 | 5,531751322 | 3,359801767 | 0,06680611 | 0,138815477 |
| b4300 | 783 | 0,5 | -0,535293731 | 4,52392198 | 3,356561243 | 0,066937709 | 0,139024473 |

| | | | | | | | |
|---|---|---|---|---|---|---|---|
| b4105 | 1017 | 0,55 | 1,345313794 | 1,451955332 | 3,355005984 | 0,067000967 | 0,139091401 |
| b1932 | 480 | 0,47 | 0,541209673 | 3,504472482 | 3,351784511 | 0,0671322 | 0,139299315 |
| b2794 | 849 | 0,49 | 0,352799367 | 5,878445223 | 3,349426141 | 0,067228447 | 0,139434474 |
| b4061 | 1587 | 0,45 | -0,435795449 | 5,87374105 | 3,347145444 | 0,067321664 | 0,139563227 |
| b3501 | 354 | 0,51 | 1,014834791 | 1,821306931 | 3,340701542 | 0,067585787 | 0,139990009 |
| b4512 | 198 | 0,52 | -0,440043153 | 5,741941616 | 3,340598984 | 0,06759 | 0,139990009 |
| b2133 | 1716 | 0,52 | -0,390478154 | 9,050190852 | 3,334866255 | 0,067825921 | 0,140413754 |
| b3106 | 702 | 0,53 | 0,549750232 | 3,898534846 | 3,330825797 | 0,067992728 | 0,140694094 |
| b1260 | 807 | 0,54 | -2,244878643 | 10,64536199 | 3,329151332 | 0,068061986 | 0,140772413 |
| b3023 | 483 | 0,53 | -0,465754734 | 6,010497377 | 3,324497404 | 0,068254873 | 0,141106246 |
| b0571 | 684 | 0,54 | 0,530802288 | 4,767028092 | 3,317349722 | 0,068552257 | 0,141655701 |
| b3084 | 1137 | 0,54 | 0,524270877 | 4,03507279 | 3,314638997 | 0,0686654 | 0,141824112 |
| b0718 | 2448 | 0,5 | 0,817547793 | 2,34132169 | 3,306361904 | 0,069012118 | 0,142474579 |
| b1565 | 306 | 0,46 | 0,995807871 | 2,047201678 | 3,302345745 | 0,069181024 | 0,142757528 |
| b1148 | 339 | 0,5 | 1,155338488 | 1,657365469 | 3,291972614 | 0,069619335 | 0,143595885 |
| b3550 | 441 | 0,52 | 0,589616393 | 3,665531013 | 3,289567417 | 0,069721389 | 0,143740232 |
| b2425 | 1017 | 0,54 | 0,703852831 | 3,189227648 | 3,284725657 | 0,069927314 | 0,144098492 |
| b4016 | 1737 | 0,53 | -0,598265606 | 10,81141346 | 3,279879669 | 0,070134071 | 0,144458137 |
| b0070 | 1179 | 0,49 | -0,393019105 | 5,249773389 | 3,272891081 | 0,070433397 | 0,145008032 |
| b2194 | 1053 | 0,54 | -0,850687383 | 5,504474637 | 3,269794016 | 0,070566484 | 0,145155428 |
| b0727 | 1218 | 0,56 | 0,824052715 | 11,65757003 | 3,269717777 | 0,070569763 | 0,145155428 |
| b2381 | 735 | 0,47 | -0,381892848 | 5,856759289 | 3,267736038 | 0,070655068 | 0,145264226 |
| b1246 | 1014 | 0,52 | 0,687775754 | 9,999212738 | 3,264801914 | 0,070781571 | 0,145392278 |
| b3370 | 1338 | 0,55 | 0,776788508 | 2,600208609 | 3,264786664 | 0,070782229 | 0,145392278 |
| b1326 | 729 | 0,55 | -0,392223906 | 6,605729065 | 3,259909717 | 0,070993035 | 0,14575849 |
| b2495 | 360 | 0,52 | 0,449122681 | 5,880586894 | 3,25716251 | 0,071112079 | 0,145936053 |
| b1200 | 1071 | 0,53 | -0,51483671 | 6,675858825 | 3,253571457 | 0,071268012 | 0,146189122 |
| b2859 | 399 | 0,36 | 1,470382263 | 1,225453711 | 3,250992742 | 0,071380213 | 0,146352294 |
| b2559 | 504 | 0,55 | -0,486289623 | 4,578433534 | 3,249874243 | 0,071428938 | 0,146385231 |
| b0158 | 801 | 0,53 | 0,595765085 | 4,445706782 | 3,247102966 | 0,071549816 | 0,14656594 |
| b3770 | 930 | 0,55 | 0,453532537 | 6,01886506 | 3,245255259 | 0,071630532 | 0,146664251 |
| b2831 | 690 | 0,54 | 0,53328444 | 4,134220073 | 3,238180072 | 0,07194051 | 0,147231674 |
| b0394 | 909 | 0,56 | -0,346565358 | 7,609665606 | 3,231508945 | 0,072234103 | 0,147738591 |
| b0187 | 390 | 0,51 | -0,4708793 | 5,615908432 | 3,230536246 | 0,072277018 | 0,147738591 |
| b4095 | 1137 | 0,61 | 0,948960225 | 2,002457468 | 3,230308084 | 0,072287088 | 0,147738591 |
| b3965 | 1101 | 0,51 | -0,362196781 | 7,072951457 | 3,22566722 | 0,072492249 | 0,148090365 |
| b0383 | 1416 | 0,53 | -0,454588726 | 5,62749173 | 3,222851605 | 0,072617025 | 0,148277679 |
| b1294 | 1644 | 0,54 | -0,391588047 | 6,928918414 | 3,221404182 | 0,072681258 | 0,148341256 |
| b3350 | 1806 | 0,55 | 0,429437886 | 5,76836372 | 3,215575359 | 0,072940544 | 0,148802693 |
| b1006 | 1329 | 0,54 | 0,550821511 | 4,018421984 | 3,20545471 | 0,073393105 | 0,149657824 |
| b1110 | 540 | 0,54 | 0,667120753 | 4,035567809 | 3,202983567 | 0,073504063 | 0,149815922 |
| b1960 | 471 | 0,56 | 0,714431634 | 3,248185797 | 3,201156425 | 0,073586221 | 0,149910136 |
| b2485 | 651 | 0,53 | 1,501528023 | 1,561333306 | 3,200468433 | 0,073617182 | 0,149910136 |
| b4390 | 1233 | 0,51 | -0,453860604 | 7,689184511 | 3,184400756 | 0,074344251 | 0,15132195 |
| b0572 | 1374 | 0,51 | 0,952957809 | 2,080994749 | 3,182388984 | 0,074435827 | 0,151439571 |
| b2085 | 318 | 0,36 | -1,048158544 | 2,206344639 | 3,177090621 | 0,074677587 | 0,151797878 |
| b2131 | 918 | 0,54 | -0,382945065 | 7,356848504 | 3,177044842 | 0,074679679 | 0,151797878 |
| b1480 | 138 | 0,44 | 0,474505208 | 8,14241633 | 3,173794336 | 0,074828419 | 0,151947337 |
| b0326 | 816 | 0,39 | 1,649027007 | 0,908231328 | 3,173264732 | 0,074852683 | 0,151947337 |
| b2222 | 651 | 0,54 | 1,640673337 | 0,842146059 | 3,173216067 | 0,074854913 | 0,151947337 |
| b2760 | 1509 | 0,44 | 0,741859613 | 2,882962142 | 3,170291616 | 0,074989056 | 0,152150724 |
| b2919 | 786 | 0,49 | 0,737715363 | 2,135278146 | 3,165013932 | 0,075231794 | 0,152574163 |
| b1065 | 1209 | 0,55 | -0,642065137 | 3,44042464 | 3,161727737 | 0,075383363 | 0,152812408 |
| b3610 | 252 | 0,52 | 0,318526848 | 7,066249244 | 3,155339429 | 0,075678951 | 0,153342251 |
| b1910 | 74 | 0,57 | 1,837296913 | 0,611356134 | 3,151826796 | 0,075842012 | 0,153603207 |
| b0557 | 294 | 0,41 | 0,620073502 | 5,280431938 | 3,150348495 | 0,075910749 | 0,15367298 |
| b3094 | 777 | 0,51 | -0,463639564 | 7,001137744 | 3,145627891 | 0,076130694 | 0,154048655 |
| b1247 | 1005 | 0,52 | 0,874079765 | 10,03633785 | 3,141604013 | 0,076318718 | 0,154359429 |
| b0148 | 2430 | 0,57 | -0,327161282 | 6,709205866 | 3,1364326 | 0,076561096 | 0,154779807 |
| b2369 | 615 | 0,4 | -0,67039175 | 6,161453147 | 3,126882629 | 0,077010871 | 0,155618901 |
| b1305 | 225 | 0,48 | 0,521390986 | 3,837810532 | 3,125076051 | 0,077096274 | 0,155703931 |
| b2052 | 966 | 0,56 | 1,037660068 | 2,232708677 | 3,124337615 | 0,077131212 | 0,155703931 |
| b2212 | 651 | 0,53 | -0,528550664 | 4,340968048 | 3,123789238 | 0,077157169 | 0,155703931 |
| b1419 | 174 | 0,44 | -0,554423467 | 3,817695461 | 3,122250264 | 0,077230064 | 0,155780894 |
| b4329 | 462 | 0,53 | 0,867190251 | 2,432918385 | 3,121284885 | 0,077275828 | 0,155803086 |
| b1199 | 633 | 0,54 | -0,514144546 | 5,726652873 | 3,10166028 | 0,078212492 | 0,157620678 |
| b0164 | 813 | 0,51 | 0,701575102 | 3,045692596 | 3,09792445 | 0,07839218 | 0,157911798 |
| b3293 | 369 | 0,48 | 0,415693628 | 5,661135697 | 3,091764575 | 0,078689433 | 0,15843937 |
| b0554 | 216 | 0,48 | 1,20755746 | 1,448385101 | 3,083112618 | 0,079108996 | 0,159212626 |
| b2502 | 1542 | 0,51 | 0,31990205 | 7,243716535 | 3,079942854 | 0,079263311 | 0,159451598 |
| b1463 | 846 | 0,51 | -0,471020975 | 4,455210657 | 3,077511774 | 0,079381884 | 0,159581146 |
| b3359 | 1221 | 0,55 | 0,449589314 | 5,091497096 | 3,076007544 | 0,079455347 | 0,159581146 |
| b4032 | 891 | 0,55 | -0,9084078 | 7,648073741 | 3,075467777 | 0,079481726 | 0,159581146 |
| b4502 | 255 | 0,56 | 0,876221469 | 2,831465914 | 3,07528566 | 0,079490628 | 0,159581146 |
| b2135 | 588 | 0,54 | -0,769648986 | 6,908347119 | 3,074976691 | 0,079505734 | 0,159581146 |
| b2269 | 1212 | 0,35 | 1,121218717 | 1,868635129 | 3,07178783 | 0,079661819 | 0,159822861 |
| b3497 | 753 | 0,58 | 0,448123756 | 5,681350073 | 3,0647045 | 0,080009708 | 0,160448999 |
| b1992 | 744 | 0,53 | 0,373274908 | 5,26884156 | 3,058447981 | 0,080318352 | 0,16099591 |
| b3465 | 597 | 0,54 | 0,412958105 | 4,76990164 | 3,055588507 | 0,080459842 | 0,161207426 |
| b1177 | 369 | 0,42 | 0,479052422 | 4,906299951 | 3,052787943 | 0,080598677 | 0,161413436 |
| b4363 | 474 | 0,51 | -0,67823396 | 3,265889849 | 3,052007918 | 0,080637392 | 0,161418843 |
| b2644 | 483 | 0,54 | 1,269086411 | 1,318516229 | 3,045223608 | 0,080974964 | 0,162022227 |
| b3383 | 906 | 0,52 | 0,52668233 | 5,064975053 | 3,038688935 | 0,081301556 | 0,162593787 |
| b3848 | 615 | 0,56 | -0,414124645 | 6,217955862 | 3,038057868 | 0,08133317 | 0,162593787 |
| b0252 | 822 | 0,58 | 0,770791903 | 2,840202761 | 3,028779076 | 0,081799548 | 0,163453221 |
| b1508 | 267 | 0,45 | -0,615955584 | 3,99251547 | 3,025129265 | 0,081983788 | 0,163748367 |
| b1957 | 183 | 0,53 | 0,405489412 | 7,564791905 | 3,010555011 | 0,082723962 | 0,165153139 |
| b1591 | 615 | 0,53 | 0,541529417 | 4,234160088 | 3,008531159 | 0,082827315 | 0,165285853 |
| b3514 | 3114 | 0,53 | 0,760615501 | 9,324100439 | 3,006804464 | 0,082915604 | 0,1653884 |
| b3048 | 1065 | 0,4 | 0,6255806 | 3,641629694 | 2,997487846 | 0,083393733 | 0,16626811 |
| b2168 | 939 | 0,53 | 0,29333943 | 7,206996634 | 2,97822569 | 0,08439174 | 0,168183094 |
| b2646 | 330 | 0,48 | 0,962532175 | 1,984204591 | 2,974006406 | 0,084612068 | 0,168487428 |
| b3980 | 1185 | 0,53 | 0,620129646 | 10,80835909 | 2,973861719 | 0,084619634 | 0,168487428 |
| b2553 | 339 | 0,51 | 0,361268581 | 6,041442885 | 2,969795032 | 0,084832602 | 0,168836467 |

| | | | | | | | |
|---|---|---|---|---|---|---|---|
| b0333 | 1170 | 0,56 | 0,713815384 | 2,78681092 | 2,968102542 | 0,084921407 | 0,168847562 |
| b2050 | 1224 | 0,56 | 0,739210924 | 2,56737855 | 2,96793608 | 0,084930147 | 0,168847562 |
| b1340 | 564 | 0,54 | 0,389923265 | 4,996798454 | 2,967535267 | 0,084951194 | 0,168847562 |
| b2361 | 537 | 0,46 | 1,507822018 | 0,850711241 | 2,96354421 | 0,085161082 | 0,169189703 |
| b4312 | 603 | 0,43 | 1,273150931 | 4,870884771 | 2,958892113 | 0,085406442 | 0,169601982 |
| b0090 | 1068 | 0,56 | -0,354736576 | 7,504682017 | 2,956691904 | 0,085522751 | 0,169686942 |
| b1727 | 669 | 0,52 | 0,466264809 | 4,921417257 | 2,956650441 | 0,085524945 | 0,169686942 |
| b0024 | 219 | 0,49 | 0,705911399 | 2,690928714 | 2,954931062 | 0,085615958 | 0,169792355 |
| b0112 | 1374 | 0,53 | -0,41009979 | 5,786348992 | 2,951935888 | 0,085774753 | 0,170032041 |
| b2496 | 702 | 0,54 | -0,370517143 | 5,601686006 | 2,950687131 | 0,085841053 | 0,17008824 |
| b1864 | 741 | 0,52 | 0,378628464 | 7,99569642 | 2,948401477 | 0,085962548 | 0,170253707 |
| b1206 | 1680 | 0,55 | -0,499831945 | 5,564323008 | 2,947360932 | 0,08601792 | 0,170288126 |
| b3033 | 423 | 0,48 | 0,415578365 | 5,557392996 | 2,943500331 | 0,086223698 | 0,170620138 |
| b0342 | 612 | 0,43 | 0,830728041 | 2,21088617 | 2,938804804 | 0,086474697 | 0,170963973 |
| b0681 | 1407 | 0,51 | 0,569482835 | 3,728348996 | 2,938244514 | 0,0865047 | 0,170963973 |
| b0594 | 1611 | 0,56 | 1,047827045 | 3,245724683 | 2,938110278 | 0,08651189 | 0,170963973 |
| b2418 | 852 | 0,52 | -0,401646363 | 6,358029634 | 2,932918848 | 0,086790449 | 0,171438867 |
| b0824 | 900 | 0,54 | -0,704124736 | 2,810149492 | 2,925720509 | 0,0871783 | 0,172129137 |
| b2198 | 210 | 0,57 | -0,844343374 | 3,257496979 | 2,900365152 | 0,088559489 | 0,17477923 |
| b0533 | 984 | 0,46 | 1,371041242 | 1,103386869 | 2,898426439 | 0,088666069 | 0,174912553 |
| b0785 | 453 | 0,57 | -0,655622368 | 7,07806388 | 2,88794197 | 0,089244861 | 0,175976888 |
| b3035 | 1482 | 0,51 | -0,374831332 | 9,831615109 | 2,882869263 | 0,089526368 | 0,176454344 |
| b0388 | 525 | 0,52 | -0,531501014 | 5,963672961 | 2,880398071 | 0,089663853 | 0,176647645 |
| b1813 | 579 | 0,55 | 0,400430046 | 4,839280147 | 2,87761626 | 0,089818895 | 0,176875346 |
| b3323 | 1470 | 0,49 | 0,857913548 | 2,600218198 | 2,874323587 | 0,090002785 | 0,177159632 |
| b0132 | 903 | 0,49 | 0,457920559 | 4,718018456 | 2,87165792 | 0,090151957 | 0,17737536 |
| b3264 | 663 | 0,44 | 1,033344401 | 1,538045895 | 2,863960293 | 0,090584227 | 0,178147655 |
| b2229 | 624 | 0,51 | 1,361986444 | 1,084506272 | 2,858870721 | 0,090871274 | 0,178633794 |
| b1287 | 1128 | 0,51 | 0,6168524 | 2,915181774 | 2,856154537 | 0,091024868 | 0,178857281 |
| b2005 | 375 | 0,53 | 1,098064075 | 1,277320323 | 2,853717483 | 0,091162918 | 0,179050042 |
| b1811 | 180 | 0,56 | 0,597756415 | 4,067180486 | 2,851320948 | 0,091298894 | 0,179209848 |
| b0418 | 519 | 0,53 | -0,411626935 | 6,371981862 | 2,850699545 | 0,091334187 | 0,179209848 |
| b4493 | 1001 | 0,48 | 0,484197294 | 8,382354563 | 2,849950218 | 0,091376766 | 0,179209848 |
| b3642 | 642 | 0,53 | 0,733931583 | 2,618571122 | 2,849467276 | 0,09140422 | 0,179209848 |
| b0977 | 858 | 0,55 | 0,603065536 | 3,969766726 | 2,845807439 | 0,09161256 | 0,179539787 |
| b4373 | 447 | 0,52 | 0,474788395 | 5,396690112 | 2,845093818 | 0,091653244 | 0,179541013 |
| b1931 | 669 | 0,52 | -0,539268299 | 4,862749202 | 2,839000185 | 0,092001443 | 0,180059976 |
| b3259 | 882 | 0,54 | 0,381718606 | 5,7522289 | 2,837852057 | 0,09206721 | 0,180059976 |
| b3415 | 1317 | 0,53 | -0,447341748 | 5,605198199 | 2,837761106 | 0,092072422 | 0,180059976 |
| b3202 | 1434 | 0,53 | 0,357214343 | 8,907637378 | 2,837648706 | 0,092078863 | 0,180059976 |
| b4046 | 516 | 0,52 | -0,578385495 | 6,300226099 | 2,833342603 | 0,092326013 | 0,180420154 |
| b1394 | 789 | 0,54 | -1,274502315 | 4,737908996 | 2,832890855 | 0,092351983 | 0,180420154 |
| b0013 | 405 | 0,49 | 0,897702733 | 1,719680841 | 2,832337349 | 0,092383814 | 0,180420154 |
| b2732 | 354 | 0,51 | -1,051564302 | 3,918179232 | 2,831235864 | 0,092447192 | 0,180465295 |
| b3994 | 1896 | 0,56 | 0,496912004 | 4,186531103 | 2,829550338 | 0,092544268 | 0,180576147 |
| b3985 | 498 | 0,52 | 0,562238061 | 10,81523709 | 2,827929638 | 0,092637715 | 0,180679824 |
| b1185 | 531 | 0,53 | 0,474617861 | 5,038732166 | 2,825881408 | 0,092755958 | 0,180831755 |
| b1410 | 1758 | 0,51 | 0,637074355 | 3,461617483 | 2,823030672 | 0,092920804 | 0,181074366 |
| b0130 | 1230 | 0,5 | -0,499878259 | 4,726754896 | 2,821620779 | 0,093002449 | 0,181154706 |
| b1288 | 789 | 0,53 | 0,418603755 | 9,276257175 | 2,818050414 | 0,093209555 | 0,181479246 |
| b3877 | 1386 | 0,52 | 0,799777686 | 2,765055618 | 2,814992968 | 0,093387306 | 0,181746376 |
| b0250 | 468 | 0,53 | -0,462449334 | 5,537381652 | 2,807751826 | 0,093809757 | 0,182430784 |
| b1395 | 1428 | 0,53 | -1,274756501 | 5,533367247 | 2,807570145 | 0,093820383 | 0,182430784 |
| b0946 | 543 | 0,49 | -0,416963121 | 6,234294696 | 2,806845053 | 0,093862805 | 0,182434125 |
| b2886 | 489 | 0,55 | 0,829168422 | 2,280950383 | 2,805799501 | 0,093924012 | 0,182473958 |
| b0525 | 495 | 0,48 | 0,422194361 | 8,685750774 | 2,799756756 | 0,094278609 | 0,183083503 |
| b2544 | 873 | 0,55 | 0,49469127 | 4,077715724 | 2,796003192 | 0,094499606 | 0,18343319 |
| b3484 | 1137 | 0,42 | 0,994553956 | 1,752192288 | 2,793991521 | 0,094618279 | 0,183584037 |
| b4296 | 1350 | 0,49 | 0,525132256 | 3,837111676 | 2,788494348 | 0,094943396 | 0,184095588 |
| b3025 | 660 | 0,52 | 0,472765745 | 4,084116633 | 2,788145348 | 0,094964078 | 0,184095588 |
| b1215 | 855 | 0,52 | 0,360854918 | 8,138276916 | 2,786554539 | 0,095058412 | 0,184198791 |
| b4339 | 921 | 0,49 | -0,469908488 | 4,496109348 | 2,782190983 | 0,095317692 | 0,184621389 |
| b0597 | 414 | 0,57 | 0,98326543 | 2,565134194 | 2,775665924 | 0,095706844 | 0,185295064 |
| b3110 | 1332 | 0,51 | 0,59224507 | 6,009028656 | 2,770300781 | 0,096028115 | 0,185836793 |
| b0496 | 2415 | 0,58 | 0,355129366 | 5,917093323 | 2,768880486 | 0,096113361 | 0,185921486 |
| b4155 | 978 | 0,53 | -0,478841955 | 4,559597966 | 2,760044399 | 0,096645555 | 0,18687031 |
| b0140 | 741 | 0,44 | 1,402094492 | 1,090598565 | 2,759258286 | 0,096693058 | 0,186881537 |
| b4537 | 252 | 0,48 | 0,915428449 | 2,565621825 | 2,752308208 | 0,09711414 | 0,187614472 |
| b3546 | 1692 | 0,5 | -0,402979296 | 5,561688082 | 2,751122296 | 0,09718619 | 0,187672772 |
| b2368 | 1164 | 0,42 | 0,878648646 | 2,414175757 | 2,747361196 | 0,097415081 | 0,187958995 |
| b2464 | 951 | 0,5 | -0,46047475 | 9,527491711 | 2,747308644 | 0,097418283 | 0,187958995 |
| b3557 | 522 | 0,46 | 0,852319264 | 2,441242157 | 2,741630097 | 0,097764991 | 0,188546769 |
| b4525 | 138 | 0,41 | 0,983158806 | 1,403981781 | 2,74077261 | 0,097817463 | 0,188566825 |
| b2757 | 675 | 0,48 | 0,540355866 | 3,604375159 | 2,736813479 | 0,098060129 | 0,188953353 |
| b3466 | 270 | 0,46 | 0,691556676 | 3,150682777 | 2,732842236 | 0,098304197 | 0,189342249 |
| b2055 | 747 | 0,46 | 1,600514667 | 0,809848627 | 2,73209894 | 0,098349953 | 0,189349008 |
| b4661 | 2646 | 0,52 | -0,478288211 | 5,016866831 | 2,730716804 | 0,098435096 | 0,189431559 |
| b1174 | 267 | 0,43 | 0,391805236 | 6,601738976 | 2,727270713 | 0,098647735 | 0,189707601 |
| b1826 | 144 | 0,47 | 0,692086485 | 3,044479062 | 2,727020555 | 0,09866319 | 0,189707601 |
| b2341 | 2145 | 0,52 | 0,386018889 | 7,767254864 | 2,721858975 | 0,098982675 | 0,190240286 |
| b0924 | 4461 | 0,56 | 0,38831341 | 9,507527838 | 2,719349445 | 0,099138414 | 0,190414945 |
| b3495 | 435 | 0,49 | -0,594351027 | 10,72450649 | 2,719025793 | 0,099158519 | 0,190414945 |
| b1568 | 279 | 0,51 | 1,388185233 | 1,197053981 | 2,716826328 | 0,099295266 | 0,190595881 |
| b2038 | 558 | 0,39 | 0,433594024 | 6,014295591 | 2,714951883 | 0,099411968 | 0,190669027 |
| b1109 | 1305 | 0,53 | -0,484940427 | 5,850822557 | 2,71484776 | 0,099418455 | 0,190669027 |
| b2070 | 1947 | 0,51 | -0,483762324 | 4,338395606 | 2,710626794 | 0,099681818 | 0,191092348 |
| b2296 | 1203 | 0,52 | -0,424323435 | 8,866679324 | 2,709340693 | 0,099762215 | 0,191164706 |
| b3729 | 1830 | 0,54 | 0,365369162 | 9,996332625 | 2,70847491 | 0,099816376 | 0,191186751 |
| b2388 | 966 | 0,52 | 0,300027937 | 7,414722897 | 2,707469339 | 0,099879323 | 0,191225598 |
| b2750 | 606 | 0,52 | -0,564419347 | 3,074919245 | 2,705503833 | 0,100002484 | 0,191260803 |
| b4221 | 3780 | 0,55 | -0,380230426 | 8,334968435 | 2,70542184 | 0,100007626 | 0,191260803 |
| b0570 | 1443 | 0,52 | 0,435437114 | 4,578236373 | 2,70513316 | 0,10002573 | 0,191260803 |
| b1320 | 999 | 0,45 | 0,738398723 | 3,302882142 | 2,701292797 | 0,100266913 | 0,191570877 |
| b2763 | 1713 | 0,56 | 0,613621423 | 3,613204055 | 2,701190007 | 0,100273377 | 0,191570877 |

| | | | | | | | |
|---|---|---|---|---|---|---|---|
| b3803 | 1182 | 0,53 | -0,325271242 | 8,377519983 | 2,700508306 | 0,100316259 | 0,191571143 |
| b2882 | 1401 | 0,49 | -0,505835021 | 3,911491889 | 2,692133525 | 0,100844704 | 0,192490904 |
| b2819 | 1827 | 0,55 | -0,375596273 | 6,475653832 | 2,691516043 | 0,100883787 | 0,192490904 |
| b3473 | 1218 | 0,57 | -0,447395342 | 4,427088027 | 2,682454898 | 0,101459213 | 0,193506466 |
| b3601 | 588 | 0,51 | -0,438267178 | 7,590717927 | 2,681600939 | 0,101513629 | 0,193527896 |
| b4702 | 54 | 0,5 | 1,79392624 | 0,443827977 | 2,67935036 | 0,101657191 | 0,193719188 |
| b4310 | 1107 | 0,44 | 0,53017016 | 5,367827375 | 2,675168788 | 0,101924519 | 0,194146067 |
| b1881 | 645 | 0,53 | 0,682412957 | 3,178550588 | 2,673932744 | 0,102003687 | 0,194214327 |
| b1641 | 468 | 0,51 | -0,381309098 | 9,697099834 | 2,672375821 | 0,102103502 | 0,194321825 |
| b0972 | 1119 | 0,56 | -0,665766741 | 5,792629944 | 2,665830419 | 0,102524302 | 0,195039865 |
| b3864 | 109 | 0,42 | 1,79533679 | 0,728032521 | 2,661975118 | 0,102773045 | 0,195430118 |
| b4180 | 732 | 0,56 | -0,319234821 | 6,336151564 | 2,652519392 | 0,103385926 | 0,196403608 |
| b1343 | 1374 | 0,52 | 0,354378418 | 5,628990952 | 2,652315336 | 0,103399196 | 0,196403608 |
| b2375 | 636 | 0,46 | 1,11813712 | 1,278566862 | 2,652050107 | 0,103416447 | 0,196403608 |
| b2036 | 1104 | 0,36 | -0,393015048 | 7,876501193 | 2,649617565 | 0,103574811 | 0,196621052 |
| b4196 | 651 | 0,57 | -0,5572434 | 3,925748648 | 2,64568179 | 0,103831603 | 0,197025082 |
| b4100 | 585 | 0,61 | 1,502558532 | 1,0959482 | 2,638384125 | 0,104309589 | 0,197848318 |
| b2765 | 366 | 0,49 | 0,894410875 | 2,700957127 | 2,635105351 | 0,104525128 | 0,198173275 |
| b1781 | 855 | 0,51 | -0,377445344 | 5,159067022 | 2,633204039 | 0,104650339 | 0,198326773 |
| b0295 | 228 | 0,44 | 1,148360248 | 1,332243584 | 2,632426147 | 0,104701614 | 0,198340083 |
| b1758 | 621 | 0,53 | -0,470951618 | 4,288406382 | 2,627392913 | 0,105034052 | 0,198885771 |
| b0460 | 219 | 0,41 | -0,613337574 | 5,60745493 | 2,624173815 | 0,105247274 | 0,199205356 |
| b2416 | 1728 | 0,5 | -0,393951678 | 11,11542489 | 2,622209392 | 0,105377624 | 0,199367882 |
| b4513 | 90 | 0,49 | 1,771357471 | 0,661053533 | 2,616878082 | 0,105732278 | 0,19995446 |
| b2434 | 426 | 0,52 | 0,393774304 | 5,971641467 | 2,615112752 | 0,105850001 | 0,200092663 |
| b1561 | 252 | 0,4 | 0,848764071 | 2,005128858 | 2,614140869 | 0,105914874 | 0,200130887 |
| b2371 | 1146 | 0,45 | -0,696495222 | 3,65382269 | 2,611864112 | 0,106067016 | 0,200333909 |
| b1314 | 789 | 0,54 | 0,783542307 | 2,738766795 | 2,610742312 | 0,106142068 | 0,200391216 |
| b4266 | 765 | 0,49 | -0,60456702 | 3,410590187 | 2,609034984 | 0,106256404 | 0,200515044 |
| b1096 | 810 | 0,51 | 0,340318989 | 5,325970087 | 2,608427311 | 0,106297132 | 0,200515044 |
| b2118 | 3633 | 0,51 | 0,457746095 | 4,192869763 | 2,604832389 | 0,106538421 | 0,200885655 |
| b0051 | 822 | 0,53 | 0,318108103 | 7,388726629 | 2,600262685 | 0,106846004 | 0,201315092 |
| b3073 | 1380 | 0,55 | -0,388457988 | 8,320526311 | 2,600114382 | 0,106856003 | 0,201315092 |
| b3935 | 2199 | 0,57 | 0,358594289 | 5,450360102 | 2,59515232 | 0,107191134 | 0,201861623 |
| b2181 | 345 | 0,49 | -0,417970443 | 5,623379882 | 2,58703534 | 0,107741833 | 0,202739987 |
| b1944 | 465 | 0,53 | 0,98237191 | 1,577891465 | 2,586944339 | 0,107748025 | 0,202739987 |
| b0932 | 2613 | 0,53 | 0,433079889 | 9,946408958 | 2,57994363 | 0,108225516 | 0,203552985 |
| b1244 | 921 | 0,46 | 0,411942681 | 9,127816936 | 2,570779398 | 0,108854088 | 0,204567399 |
| b2567 | 681 | 0,51 | -0,368018442 | 7,16161985 | 2,569957776 | 0,108910639 | 0,204567399 |
| b4472 | 3801 | 0,53 | -0,316491653 | 7,468111957 | 2,569647386 | 0,108932011 | 0,204567399 |
| b0303 | 237 | 0,35 | 1,48831622 | 0,897706758 | 2,569423486 | 0,10894743 | 0,204567399 |
| b3662 | 1191 | 0,53 | 0,826979607 | 2,381798669 | 2,568080333 | 0,10903998 | 0,20465544 |
| b2225 | 777 | 0,53 | 0,70375266 | 2,445348643 | 2,565810192 | 0,109196601 | 0,204855629 |
| b0404 | 582 | 0,53 | 0,457060042 | 4,231493981 | 2,565209966 | 0,109238053 | 0,204855629 |
| b3082 | 417 | 0,48 | 0,469416331 | 4,054860603 | 2,561504278 | 0,109494353 | 0,205227158 |
| b3418 | 2706 | 0,54 | -0,473057098 | 10,6872312 | 2,561022159 | 0,109527747 | 0,205227158 |
| b3272 | 120 | 0,62 | -0,514432405 | 9,03816011 | 2,558081495 | 0,109731673 | 0,20547928 |
| b3721 | 1413 | 0,49 | 0,906671487 | 1,908295807 | 2,557554677 | 0,109768251 | 0,20547928 |
| b1123 | 1047 | 0,51 | -0,478234092 | 8,948729203 | 2,557099887 | 0,109799838 | 0,20547928 |
| b0996 | 1173 | 0,51 | 0,928135533 | 2,012406034 | 2,555394564 | 0,10991837 | 0,205597398 |
| b4251 | 594 | 0,49 | 0,485828794 | 3,651891469 | 2,554872283 | 0,1099547 | 0,205597398 |
| b2736 | 909 | 0,53 | -0,557440621 | 3,885423672 | 2,550210358 | 0,110279571 | 0,206118865 |
| b0882 | 2277 | 0,53 | -0,553292923 | 11,47573688 | 2,547251307 | 0,110486324 | 0,206419218 |
| b2826 | 471 | 0,54 | 0,690401829 | 1,996512968 | 2,542153744 | 0,110843497 | 0,207000231 |
| b3563 | 342 | 0,42 | 1,196437061 | 1,148462004 | 2,53833224 | 0,111112094 | 0,207376639 |
| b4484 | 501 | 0,5 | 0,351690162 | 7,451931097 | 2,537969986 | 0,111137592 | 0,207376639 |
| b3338 | 2694 | 0,5 | -0,310624783 | 5,777607331 | 2,529966211 | 0,111702608 | 0,208344191 |
| b1093 | 735 | 0,52 | 0,343759184 | 9,940594572 | 2,528339853 | 0,111817805 | 0,208472297 |
| b1084 | 3186 | 0,56 | 0,384239045 | 9,689308636 | 2,526302175 | 0,111962321 | 0,208654936 |
| b0803 | 267 | 0,52 | -0,696659837 | 4,80740523 | 2,524365849 | 0,112099839 | 0,208824388 |
| b4185 | 639 | 0,49 | 1,389982201 | 1,146261342 | 2,52254187 | 0,112229548 | 0,208979158 |
| b3882 | 897 | 0,55 | 0,655473936 | 2,815334554 | 2,521420726 | 0,112309358 | 0,209040924 |
| b2740 | 1365 | 0,52 | 0,614803592 | 3,36328308 | 2,519284828 | 0,112461579 | 0,209237358 |
| b3930 | 927 | 0,53 | 0,597441619 | 4,753652421 | 2,51419017 | 0,112825581 | 0,209763315 |
| b1154 | 630 | 0,5 | 1,27477773 | 1,19411108 | 2,514018418 | 0,112837874 | 0,209763315 |
| b2716 | 1425 | 0,52 | -0,406808665 | 4,660435336 | 2,508184056 | 0,113256372 | 0,210454005 |
| b2663 | 1401 | 0,51 | 0,614741874 | 8,04252483 | 2,507408137 | 0,113312157 | 0,210470406 |
| b0794 | 1737 | 0,56 | -0,283586633 | 7,099804214 | 2,505794415 | 0,113428274 | 0,210598809 |
| b0387 | 459 | 0,56 | -0,477052355 | 4,501064907 | 2,504931254 | 0,113490437 | 0,210626973 |
| b4080 | 1467 | 0,56 | 0,830833607 | 1,786189376 | 2,503860831 | 0,113567579 | 0,210682902 |
| b4644 | 272 | 0,55 | 1,458406681 | 0,651648342 | 2,499318199 | 0,113895596 | 0,211203998 |
| b3283 | 543 | 0,5 | -0,346458967 | 7,234446088 | 2,497194949 | 0,114049271 | 0,211401503 |
| b4570 | 1596 | 0,57 | 0,861128751 | 1,635370068 | 2,48786064 | 0,114727581 | 0,212570904 |
| b2099 | 1005 | 0,56 | 0,470677973 | 4,09521378 | 2,486940551 | 0,114794682 | 0,212583819 |
| b1413 | 3903 | 0,53 | -0,339719684 | 8,68780842 | 2,485853048 | 0,11487405 | 0,212583819 |
| b3704 | 360 | 0,56 | 0,700047926 | 5,163428996 | 2,485367997 | 0,114909469 | 0,212583819 |
| b2668 | 525 | 0,46 | -0,608844322 | 4,584480562 | 2,484877203 | 0,114945319 | 0,212583819 |
| b0133 | 852 | 0,53 | 0,293875083 | 8,257191887 | 2,484516109 | 0,114971704 | 0,212583819 |
| b2298 | 1521 | 0,52 | -0,669660647 | 3,569266076 | 2,483684049 | 0,115032526 | 0,212608571 |
| b3234 | 1368 | 0,52 | -0,328220125 | 7,661429399 | 2,478669474 | 0,115399838 | 0,213199536 |
| b3934 | 1026 | 0,52 | 0,392391127 | 6,368039228 | 2,474433661 | 0,115711115 | 0,213686534 |
| b0751 | 720 | 0,48 | -0,360452331 | 5,947936084 | 2,471863102 | 0,11590047 | 0,213948066 |
| b0857 | 846 | 0,55 | 0,517095179 | 3,693779607 | 2,470316906 | 0,116014531 | 0,214047692 |
| b2255 | 1983 | 0,51 | 0,407211866 | 6,400513597 | 2,469837073 | 0,116049953 | 0,214047692 |
| b2271 | 972 | 0,45 | 1,021220566 | 1,632206815 | 2,466800133 | 0,116274423 | 0,214209778 |
| b2108 | 1035 | 0,39 | 1,324304029 | 1,142345105 | 2,466725802 | 0,116279923 | 0,214209778 |
| b1028 | 342 | 0,44 | 1,734458609 | 0,582543843 | 2,466708388 | 0,116281211 | 0,214209778 |
| b1607 | 336 | 0,47 | 0,658257488 | 3,31093393 | 2,461650662 | 0,116656132 | 0,214812155 |
| b2197 | 480 | 0,54 | -0,71053405 | 4,681105095 | 2,460127991 | 0,116769267 | 0,214932178 |
| b1555 | 312 | 0,44 | 1,075085219 | 1,090278152 | 2,456753186 | 0,117020447 | 0,215306094 |
| b1066 | 585 | 0,49 | -0,289299836 | 6,639244159 | 2,453112762 | 0,117292066 | 0,215702868 |
| b3247 | 1470 | 0,52 | 0,277828267 | 7,372932899 | 2,452573647 | 0,11733235 | 0,215702868 |
| b3150 | 576 | 0,54 | 0,301984698 | 6,673987528 | 2,451027887 | 0,117447937 | 0,21577888 |
| b0028 | 450 | 0,54 | 0,359002981 | 5,916492974 | 2,45073324 | 0,117469984 | 0,21577888 |

| | | | | | | | |
|---|---|---|---|---|---|---|---|
| b3949 | 1080 | 0,54 | -0,549233366 | 3,323156094 | 2,446537542 | 0,117784424 | 0,216267836 |
| b2280 | 555 | 0,54 | 0,6945 | 9,089930214 | 2,44354071 | 0,118009586 | 0,216561265 |
| b1925 | 411 | 0,5 | 1,098726574 | 1,345301038 | 2,443124857 | 0,118040868 | 0,216561265 |
| b4181 | 402 | 0,39 | 1,169966482 | 0,932488963 | 2,439028651 | 0,118349491 | 0,217038633 |
| b1044 | 312 | 0,44 | 0,967272201 | 1,486774427 | 2,435368827 | 0,118625991 | 0,217456724 |
| b1271 | 759 | 0,52 | 0,345657455 | 5,547549665 | 2,431457713 | 0,118922265 | 0,217910707 |
| b3405 | 720 | 0,55 | -0,29309094 | 8,503762459 | 2,429959485 | 0,119035975 | 0,218029931 |
| b1582 | 327 | 0,52 | 0,686653609 | 3,266165093 | 2,427815229 | 0,119198926 | 0,21823921 |
| b2810 | 1206 | 0,56 | 0,301992327 | 5,899956801 | 2,426185137 | 0,119322969 | 0,21831368 |
| b1972 | 636 | 0,48 | -0,505266236 | 4,425764861 | 2,42600062 | 0,119337018 | 0,21831368 |
| b2123 | 462 | 0,42 | 0,892300676 | 1,762057176 | 2,424531681 | 0,119448935 | 0,218429264 |
| b2555 | 714 | 0,54 | 0,415768441 | 4,660627926 | 2,415579156 | 0,120133533 | 0,219454259 |
| b4425 | 171 | 0,58 | 0,825650662 | 2,726702906 | 2,415420877 | 0,120145675 | 0,219454259 |
| b0624 | 384 | 0,48 | -0,465353219 | 4,965601317 | 2,415040527 | 0,12017486 | 0,219454259 |
| b4262 | 1083 | 0,54 | 0,285957153 | 7,228238784 | 2,414643775 | 0,120205311 | 0,219454259 |
| b1754 | 1167 | 0,56 | -0,642080354 | 5,032968783 | 2,412099276 | 0,12040081 | 0,219721673 |
| b3995 | 477 | 0,49 | 0,40562899 | 6,694445735 | 2,406826063 | 0,120807082 | 0,220275625 |
| b0991 | 231 | 0,42 | 1,248752983 | 0,753379523 | 2,406008542 | 0,120870204 | 0,220275625 |
| b0838 | 627 | 0,52 | -0,487230271 | 7,319365824 | 2,40585988 | 0,120881686 | 0,220275625 |
| b0229 | 1713 | 0,58 | -0,447539319 | 3,994190139 | 2,405610558 | 0,120900946 | 0,220275625 |
| b1236 | 909 | 0,47 | 0,319936091 | 8,725450302 | 2,403095263 | 0,121095437 | 0,220540329 |
| b0792 | 1107 | 0,52 | -0,363413562 | 5,407573697 | 2,398209672 | 0,121474201 | 0,221140279 |
| b4400 | 1353 | 0,5 | 0,480678861 | 3,391741521 | 2,397422888 | 0,12153532 | 0,221161715 |
| b4320 | 903 | 0,5 | 0,411317821 | 4,587379577 | 2,396111465 | 0,12163727 | 0,221257405 |
| b2374 | 1251 | 0,47 | 0,741002266 | 2,176188688 | 2,391902579 | 0,121965111 | 0,221752613 |
| b1827 | 792 | 0,5 | -0,305281358 | 7,704407388 | 2,391346918 | 0,122008466 | 0,221752613 |
| b4436 | 143 | 0,48 | -0,462227491 | 4,146047579 | 2,38387258 | 0,122593308 | 0,222725256 |
| b1837 | 192 | 0,46 | -0,663430278 | 4,404431809 | 2,382178919 | 0,122726263 | 0,22280503 |
| b1710 | 552 | 0,49 | 0,461506547 | 6,620543594 | 2,382046823 | 0,12273664 | 0,22280503 |
| b1592 | 1257 | 0,54 | -0,515050339 | 3,967996814 | 2,381211832 | 0,122802253 | 0,222833885 |
| b3875 | 693 | 0,41 | 1,155117931 | 1,07695031 | 2,37903477 | 0,122973508 | 0,223054335 |
| b3258 | 1452 | 0,54 | 0,340555602 | 4,895063948 | 2,377673268 | 0,123080743 | 0,223158532 |
| b0334 | 1452 | 0,57 | 0,65968454 | 3,331289605 | 2,376779837 | 0,123151168 | 0,22319593 |
| b4428 | 150 | 0,53 | -0,576250781 | 3,480426385 | 2,374913983 | 0,123298389 | 0,223372426 |
| b4572 | 1260 | 0,57 | 0,603783234 | 2,756147924 | 2,373047769 | 0,123445834 | 0,223549183 |
| b1205 | 279 | 0,53 | -0,820004396 | 10,04947724 | 2,371770867 | 0,123546832 | 0,22364172 |
| b0149 | 2535 | 0,55 | 0,291226841 | 7,506957737 | 2,371070325 | 0,12360228 | 0,223651765 |
| b2933 | 1389 | 0,5 | 0,821941504 | 1,895492448 | 2,364292496 | 0,124140184 | 0,224534424 |
| b0617 | 297 | 0,56 | 1,288489352 | 0,901680034 | 2,358973525 | 0,124564129 | 0,225210337 |
| b2615 | 879 | 0,5 | -0,295512377 | 6,100236253 | 2,353660771 | 0,124989184 | 0,225846933 |
| b2413 | 762 | 0,5 | 0,396543264 | 4,908848025 | 2,353313669 | 0,125017011 | 0,225846933 |
| b2564 | 732 | 0,56 | -0,292943666 | 6,874118708 | 2,351363653 | 0,125173467 | 0,226035726 |
| b2127 | 732 | 0,53 | -0,323420961 | 6,685792553 | 2,350754578 | 0,12522238 | 0,226035726 |
| b3537 | 192 | 0,48 | -0,527800602 | 3,558576254 | 2,349922497 | 0,125289236 | 0,226065361 |
| b1147 | 558 | 0,5 | -0,539243769 | 3,351562767 | 2,347553728 | 0,125479779 | 0,226238419 |
| b1396 | 423 | 0,52 | -1,222591087 | 3,830333562 | 2,347102529 | 0,12551611 | 0,226238419 |
| b0356 | 1110 | 0,54 | -0,316263113 | 7,184890981 | 2,346848385 | 0,125536579 | 0,226238419 |
| b0209 | 801 | 0,53 | -0,317255016 | 7,168643648 | 2,346205396 | 0,125588383 | 0,226240809 |
| b2581 | 1038 | 0,56 | 0,314671854 | 7,140069375 | 2,345003898 | 0,125685247 | 0,226324338 |
| b3747 | 1869 | 0,51 | -0,322002778 | 6,179166223 | 2,339391425 | 0,126138824 | 0,226919567 |
| b2842 | 762 | 0,52 | -0,379876991 | 4,919711696 | 2,339084332 | 0,126163695 | 0,226919567 |
| b4130 | 1458 | 0,51 | 0,527547746 | 3,303563242 | 2,33903508 | 0,126167684 | 0,226919567 |
| b1514 | 1029 | 0,54 | 0,614248466 | 6,237058958 | 2,337288054 | 0,126309279 | 0,227083108 |
| b0088 | 1317 | 0,55 | -0,339770382 | 8,403218853 | 2,333417947 | 0,126623577 | 0,227556885 |
| b1220 | 1395 | 0,51 | -0,384126292 | 4,793040766 | 2,331881442 | 0,1267486 | 0,227664108 |
| b2479 | 573 | 0,5 | 0,309574969 | 6,274847822 | 2,331436482 | 0,126784831 | 0,227664108 |
| b2452 | 1227 | 0,55 | 0,442915786 | 3,767202418 | 2,323844954 | 0,127404759 | 0,228685675 |
| b3063 | 1464 | 0,55 | 0,841245281 | 3,006580846 | 2,320048555 | 0,127716039 | 0,229116474 |
| b0921 | 786 | 0,52 | 0,312567317 | 6,186342607 | 2,319671482 | 0,127747003 | 0,229116474 |
| b2173 | 987 | 0,55 | 0,513009576 | 4,705973897 | 2,309730503 | 0,128566339 | 0,230404727 |
| b1994 | 1017 | 0,55 | 0,678926525 | 2,711010745 | 2,309709219 | 0,128568099 | 0,230404727 |
| b2734 | 657 | 0,42 | 1,054520725 | 1,712928537 | 2,308429322 | 0,128674015 | 0,230502372 |
| b4066 | 1293 | 0,33 | 0,863821573 | 1,561334135 | 2,29707102 | 0,129618218 | 0,232101021 |
| b2230 | 1689 | 0,51 | -0,452475178 | 3,485105519 | 2,29553736 | 0,1297463 | 0,232237587 |
| b1134 | 462 | 0,5 | 0,389890501 | 4,492892892 | 2,29179395 | 0,13005952 | 0,232705297 |
| b2331 | 552 | 0,53 | -0,343028023 | 5,687060354 | 2,28452556 | 0,130670094 | 0,233704454 |
| b1481 | 216 | 0,45 | 1,331479868 | 1,992861899 | 2,281354919 | 0,130937441 | 0,234033812 |
| b1361 | 500 | 0,48 | 0,722320249 | 2,497957945 | 2,281103357 | 0,130958679 | 0,234033812 |
| b1776 | 1077 | 0,5 | 0,514494826 | 3,233388362 | 2,275208385 | 0,131457455 | 0,234831531 |
| b1193 | 612 | 0,52 | 0,392370779 | 5,182880938 | 2,271191394 | 0,131798548 | 0,235347049 |
| b4038 | 1581 | 0,33 | 0,985656021 | 1,25597849 | 2,269929849 | 0,131905873 | 0,235444891 |
| b2466 | 1044 | 0,55 | 0,535433665 | 4,445879475 | 2,266104647 | 0,132231896 | 0,235932865 |
| b4504 | 222 | 0,56 | 1,261648636 | 1,097779127 | 2,254403506 | 0,133234785 | 0,237599654 |
| b3139 | 804 | 0,51 | 1,313119288 | 1,144446555 | 2,253970097 | 0,133272095 | 0,237599654 |
| b0091 | 1476 | 0,55 | -0,302309523 | 8,087564071 | 2,250216108 | 0,133595744 | 0,238081958 |
| b3653 | 1206 | 0,54 | -0,340195272 | 6,917464996 | 2,249565367 | 0,133651937 | 0,238087433 |
| b1554 | 534 | 0,51 | 1,649006 | 0,815103739 | 2,246875715 | 0,133884474 | 0,238406918 |
| b2274 | 423 | 0,4 | 0,858540565 | 1,46340006 | 2,245902375 | 0,133968736 | 0,238462223 |
| b1330 | 1032 | 0,43 | 0,342696659 | 5,34511495 | 2,24383377 | 0,134148014 | 0,238686541 |
| b4352 | 957 | 0,55 | 0,2637374 | 6,329952957 | 2,240635867 | 0,134425691 | 0,239085693 |
| b4089 | 891 | 0,47 | 0,325403097 | 6,033652372 | 2,239755884 | 0,134502213 | 0,239126902 |
| b0604 | 747 | 0,47 | 0,340815645 | 5,593056589 | 2,23490924 | 0,134924547 | 0,239725625 |
| b3779 | 1485 | 0,54 | 0,262488925 | 6,963539317 | 2,234664059 | 0,134945951 | 0,239725625 |
| b1571 | 156 | 0,48 | 1,640645745 | 0,933005088 | 2,228011164 | 0,135528201 | 0,240664579 |
| b0658 | 879 | 0,52 | -0,358633637 | 8,200647956 | 2,225216654 | 0,13577361 | 0,24100488 |
| b2837 | 1032 | 0,53 | -0,378400478 | 5,601544051 | 2,222307629 | 0,136029604 | 0,24119687 |
| b2363 | 306 | 0,43 | 0,917075584 | 1,411733735 | 2,221773588 | 0,136076658 | 0,24119687 |
| b1077 | 756 | 0,59 | 0,738587018 | 2,069097286 | 2,221695552 | 0,136083536 | 0,24119687 |
| b0015 | 1131 | 0,55 | 0,645923399 | 8,235919164 | 2,220620464 | 0,136178322 | 0,24119687 |
| b3974 | 951 | 0,44 | 0,548370007 | 5,281304565 | 2,220542304 | 0,136185216 | 0,24119687 |
| b4187 | 1626 | 0,54 | -0,562880316 | 9,931910069 | 2,220321906 | 0,136204658 | 0,24119687 |
| b4137 | 339 | 0,5 | 0,409260269 | 4,220065041 | 2,218631685 | 0,13635386 | 0,241365719 |
| b0813 | 888 | 0,53 | 0,749144257 | 3,071506611 | 2,213093924 | 0,136843983 | 0,242137675 |
| b1350 | 2601 | 0,49 | -0,458397485 | 4,105866488 | 2,210667843 | 0,137059326 | 0,242423006 |

| | | | | | | | |
|---|---|---|---|---|---|---|---|
| b1950 | 786 | 0,47 | 0,716974635 | 1,788289255 | 2,202722495 | 0,13776723 | 0,243578985 |
| b2843 | 837 | 0,5 | -0,377851595 | 4,506173762 | 2,200848311 | 0,137934811 | 0,243779109 |
| b2504 | 192 | 0,49 | -0,516366003 | 4,038998518 | 2,197000142 | 0,138279612 | 0,244292164 |
| b2631 | 372 | 0,46 | -0,385730052 | 5,527073167 | 2,191876954 | 0,138740158 | 0,244906482 |
| b2161 | 1251 | 0,5 | 0,642044406 | 2,611198632 | 2,191694131 | 0,138756624 | 0,244906482 |
| b3271 | 759 | 0,47 | 0,52679243 | 3,26619427 | 2,191309564 | 0,138791268 | 0,244906482 |
| b2493 | 1062 | 0,54 | -0,332328116 | 5,493889971 | 2,189043618 | 0,138995597 | 0,24517051 |
| b3944 | 618 | 0,53 | 0,959525483 | 1,612828549 | 2,187968707 | 0,139092644 | 0,245245173 |
| b1836 | 237 | 0,49 | 0,399357219 | 7,412506928 | 2,186152899 | 0,139256754 | 0,245418198 |
| b0962 | 2055 | 0,53 | -0,293421493 | 7,76734246 | 2,185671602 | 0,139300289 | 0,245418198 |
| b1958 | 918 | 0,53 | 0,310467437 | 6,507510661 | 2,18326383 | 0,139518312 | 0,245705726 |
| b1542 | 1461 | 0,49 | 0,580285539 | 3,10190283 | 2,180075462 | 0,139807607 | 0,246093647 |
| b2314 | 663 | 0,57 | 0,254360061 | 6,276288321 | 2,179626492 | 0,139848398 | 0,246093647 |
| b2961 | 1053 | 0,55 | 0,320499616 | 5,569872019 | 2,174343349 | 0,140329402 | 0,246843163 |
| b4206 | 639 | 0,56 | -0,325210029 | 7,214508679 | 2,168953551 | 0,140822032 | 0,247612533 |
| b3867 | 1374 | 0,53 | 0,249746764 | 7,432969003 | 2,165399192 | 0,141147964 | 0,248088304 |
| b2619 | 477 | 0,47 | 0,329633593 | 5,935148243 | 2,160053274 | 0,141639779 | 0,248855152 |
| b3240 | 1968 | 0,53 | 0,392598305 | 3,996322708 | 2,159176069 | 0,141720665 | 0,248899694 |
| b3630 | 798 | 0,43 | 0,344794019 | 5,269527154 | 2,156599568 | 0,141958538 | 0,249219808 |
| b2624 | 213 | 0,47 | 1,280953865 | 0,955965534 | 2,147920683 | 0,14276312 | 0,250534183 |
| b4532 | 177 | 0,49 | 0,615744012 | 2,874670216 | 2,147268727 | 0,142823767 | 0,250542514 |
| b2687 | 516 | 0,52 | 0,329766909 | 9,021920652 | 2,140102414 | 0,143492312 | 0,2516168 |
| b4527 | 156 | 0,49 | 1,276230893 | 0,954620004 | 2,139257333 | 0,143571381 | 0,251656993 |
| b2437 | 1053 | 0,53 | -0,439898617 | 5,24074437 | 2,134819594 | 0,143987401 | 0,252218901 |
| b3602 | 363 | 0,51 | 0,391950288 | 4,944160577 | 2,134637509 | 0,144004499 | 0,252218901 |
| b1545 | 591 | 0,45 | 0,938118875 | 1,715555633 | 2,132099378 | 0,144243081 | 0,252382489 |
| b3369 | 168 | 0,42 | 1,003657531 | 1,308469091 | 2,131166283 | 0,144330902 | 0,252382489 |
| b0765 | 1059 | 0,54 | 0,325486703 | 5,556490532 | 2,131028669 | 0,144343859 | 0,252382489 |
| b1007 | 495 | 0,59 | 0,864931659 | 1,755645076 | 2,130661463 | 0,14437844 | 0,252382489 |
| b4495 | 1129 | 0,34 | 1,227802553 | 0,940474755 | 2,130166353 | 0,144425081 | 0,252382489 |
| b4048 | 708 | 0,37 | 1,417068759 | 1,061282385 | 2,130052999 | 0,144435762 | 0,252382489 |
| b1552 | 213 | 0,4 | 1,41527404 | 0,918635112 | 2,126900344 | 0,144733174 | 0,25280362 |
| b1033 | 939 | 0,51 | 0,345968772 | 7,200892756 | 2,118719206 | 0,145508183 | 0,254058309 |
| b2095 | 1263 | 0,49 | 0,415048196 | 13,07188015 | 2,111050971 | 0,146238854 | 0,255234635 |
| b0350 | 810 | 0,55 | 0,663076388 | 2,384751058 | 2,108463763 | 0,146486309 | 0,255539536 |
| b2641 | 1016 | 0,55 | 0,42928793 | 3,806104409 | 2,108032759 | 0,146527579 | 0,255539536 |
| b2853 | 219 | 0,34 | 1,349199894 | 1,018015445 | 2,106972859 | 0,146629122 | 0,255617163 |
| b4579 | 2029 | 0,45 | 1,176655959 | 1,630703026 | 2,103407777 | 0,146971258 | 0,256113988 |
| b2254 | 969 | 0,5 | 0,549469015 | 4,800234026 | 2,101757193 | 0,147129966 | 0,256224041 |
| b4358 | 1023 | 0,51 | 0,808103619 | 2,073145565 | 2,101562013 | 0,147148746 | 0,256224041 |
| b2672 | 330 | 0,54 | -0,445175526 | 9,459886473 | 2,099032731 | 0,147392355 | 0,256548558 |
| b4101 | 453 | 0,63 | 1,405065714 | 0,956470812 | 2,097392325 | 0,147550595 | 0,256724289 |
| b4457 | 245 | 0,49 | -0,445287368 | 11,29099423 | 2,096715406 | 0,147615949 | 0,256738333 |
| b0853 | 477 | 0,48 | 0,370127748 | 5,623806417 | 2,090346719 | 0,148232425 | 0,257710524 |
| b0686 | 765 | 0,51 | 0,371225442 | 5,204656041 | 2,088511593 | 0,148410601 | 0,257920246 |
| b4383 | 1224 | 0,54 | -0,385351384 | 10,4051098 | 2,087299631 | 0,148528405 | 0,258024927 |
| b3377 | 1305 | 0,53 | 0,668080239 | 3,075090152 | 2,084278938 | 0,14882248 | 0,258435628 |
| b1035 | 555 | 0,54 | 0,359284649 | 6,36733279 | 2,083552749 | 0,148893275 | 0,258458427 |
| b0548 | 171 | 0,49 | 1,584984526 | 0,582927828 | 2,078243743 | 0,149412 | 0,25917487 |
| b0047 | 1863 | 0,56 | 0,29468406 | 6,491751858 | 2,078145106 | 0,149421657 | 0,25917487 |
| b4081 | 2052 | 0,55 | 0,56797021 | 3,124681327 | 2,077186376 | 0,149515555 | 0,259237415 |
| b3766 | 99 | 0,54 | 0,717571891 | 2,830795143 | 2,070773821 | 0,150145323 | 0,260228669 |
| b2427 | 858 | 0,52 | -0,463449375 | 3,915370627 | 2,06911024 | 0,15030919 | 0,26041198 |
| b4257 | 1197 | 0,37 | 1,019523841 | 1,556514852 | 2,068146641 | 0,1504042 | 0,260475898 |
| b0940 | 2601 | 0,48 | 0,483462158 | 3,645030944 | 2,064014736 | 0,150812372 | 0,261002839 |
| b3375 | 732 | 0,51 | -0,397344426 | 5,101593233 | 2,063887778 | 0,150824934 | 0,261002839 |
| b0482 | 795 | 0,54 | -0,336807434 | 4,970850393 | 2,060195807 | 0,151190741 | 0,261534891 |
| b0111 | 855 | 0,52 | -0,277288053 | 6,321616295 | 2,055509752 | 0,151656492 | 0,262239351 |
| b4593 | 174 | 0,32 | 1,501485757 | 0,763520589 | 2,045960099 | 0,152610677 | 0,263787526 |
| b0574 | 1224 | 0,56 | 0,69152004 | 2,433406763 | 2,042739747 | 0,152933981 | 0,264143755 |
| b4309 | 981 | 0,49 | 0,617675494 | 3,346265391 | 2,042733218 | 0,152934637 | 0,264143755 |
| b1366 | 453 | 0,49 | 0,962046661 | 1,577572992 | 2,041154842 | 0,153093381 | 0,264316076 |
| b0531 | 693 | 0,43 | 1,473443978 | 0,839333586 | 2,03712138 | 0,153499891 | 0,264895 |
| b0344 | 3075 | 0,56 | -0,408538544 | 4,878541339 | 2,036655727 | 0,153546901 | 0,264895 |
| b4359 | 2292 | 0,52 | -0,269354752 | 7,406575618 | 2,025393175 | 0,154688883 | 0,266689881 |
| b0933 | 768 | 0,56 | 0,435896696 | 3,612917171 | 2,025222019 | 0,154706312 | 0,266689881 |
| b3799 | 77 | 0,62 | 1,557608308 | 0,443499419 | 2,023726798 | 0,154858665 | 0,26675554 |
| b3697 | 813 | 0,51 | 0,288703782 | 5,846604178 | 2,023503718 | 0,15488141 | 0,26675554 |
| b1120 | 729 | 0,52 | 0,281044174 | 6,76263751 | 2,023096374 | 0,154922952 | 0,26675554 |
| b1760 | 273 | 0,51 | 0,659277488 | 2,717338309 | 2,022056874 | 0,15502902 | 0,266835663 |
| b0366 | 768 | 0,56 | 0,862362757 | 1,620796237 | 2,020281977 | 0,155210317 | 0,267045159 |
| b1806 | 582 | 0,54 | 0,348218012 | 5,770579063 | 2,016096109 | 0,155638836 | 0,267679687 |
| b0135 | 1239 | 0,4 | 0,634679087 | 2,577583595 | 2,015238799 | 0,155726767 | 0,267728182 |
| b2105 | 273 | 0,48 | 0,625466371 | 2,804251327 | 2,014065733 | 0,155847176 | 0,267830401 |
| b3221 | 465 | 0,47 | 0,326300966 | 5,357927127 | 2,013495568 | 0,155905738 | 0,267830401 |
| b1090 | 1071 | 0,52 | 0,303257262 | 7,060262243 | 2,012243467 | 0,15603443 | 0,26794878 |
| b0454 | 390 | 0,54 | -0,43250584 | 4,269557294 | 2,009381739 | 0,156329015 | 0,268280204 |
| b2739 | 777 | 0,51 | 0,495186305 | 3,276537244 | 2,009205837 | 0,156347143 | 0,268280204 |
| b2481 | 618 | 0,56 | 1,134860177 | 1,222645466 | 2,003138811 | 0,156973854 | 0,269252512 |
| b0728 | 1167 | 0,54 | 0,611372617 | 11,88029305 | 2,000927822 | 0,157202954 | 0,269542326 |
| b3194 | 783 | 0,55 | 0,272552465 | 6,19778217 | 1,999617472 | 0,15733891 | 0,269672274 |
| b4357 | 915 | 0,47 | 0,445141463 | 5,427696749 | 1,99776593 | 0,157531246 | 0,269896276 |
| b2385 | 1086 | 0,57 | 0,637071874 | 2,227618072 | 1,997200468 | 0,157590039 | 0,269896276 |
| b2625 | 1410 | 0,38 | 1,056888585 | 1,238026558 | 1,9949008 | 0,1578294 | 0,270202967 |
| b4453 | 108 | 0,53 | 0,853629112 | 1,675705838 | 1,988886082 | 0,158457398 | 0,271174516 |
| b0260 | 1404 | 0,55 | 0,461196499 | 4,208658267 | 1,988243972 | 0,158524609 | 0,271185991 |
| b0031 | 822 | 0,53 | 0,303081307 | 6,63617783 | 1,984338267 | 0,158934124 | 0,27178281 |
| b3824 | 621 | 0,52 | 0,595689712 | 3,291230288 | 1,982513924 | 0,15912582 | 0,272006837 |
| b2595 | 738 | 0,51 | 0,35231808 | 7,731288674 | 1,978319441 | 0,159567562 | 0,272657954 |
| b0649 | 1452 | 0,48 | 0,517680457 | 3,553601007 | 1,972005157 | 0,160235187 | 0,2736944 |
| b2295 | 456 | 0,51 | -0,332340424 | 6,304671753 | 1,966468379 | 0,160823223 | 0,274577177 |
| b0279 | 855 | 0,36 | 0,908036642 | 1,246242471 | 1,965986336 | 0,160874535 | 0,274577177 |
| b2460 | 702 | 0,56 | 0,430745521 | 3,625748043 | 1,960517543 | 0,161457979 | 0,275468086 |
| b2571 | 957 | 0,51 | -0,382057022 | 7,513450025 | 1,952152294 | 0,162355109 | 0,276893303 |

| | | | | | | | |
|---|---|---|---|---|---|---|---|
| b0782 | 513 | 0,52 | -0,529562163 | 8,184618623 | 1,949595142 | 0,162630485 | 0,277257449 |
| b2387 | 327 | 0,51 | 1,119006101 | 1,001473966 | 1,940048779 | 0,163663238 | 0,278912027 |
| b4019 | 3684 | 0,56 | -0,295397212 | 8,22484542 | 1,933090524 | 0,164420722 | 0,280096419 |
| b4508 | 312 | 0,51 | 1,530527143 | 0,706820788 | 1,930817069 | 0,164669081 | 0,280412926 |
| b2218 | 2850 | 0,51 | 0,281600479 | 6,69480728 | 1,928252347 | 0,164949774 | 0,280784233 |
| b2611 | 792 | 0,53 | 0,413376686 | 5,105109115 | 1,927532156 | 0,165028692 | 0,280811921 |
| b3826 | 801 | 0,52 | 0,294279953 | 5,743810834 | 1,924589358 | 0,165351614 | 0,281254624 |
| b4362 | 540 | 0,57 | 0,408066069 | 5,152530207 | 1,923324767 | 0,165490603 | 0,28138425 |
| b3437 | 528 | 0,5 | -0,473591952 | 3,786659395 | 1,921953015 | 0,165641521 | 0,281396712 |
| b4238 | 2139 | 0,54 | -0,556825874 | 8,371245554 | 1,921690052 | 0,16567047 | 0,281396712 |
| b0542 | 1564 | 0,5 | 0,703956866 | 2,681414635 | 1,921450316 | 0,165696867 | 0,281396712 |
| b0935 | 1146 | 0,57 | 0,624455749 | 2,378216691 | 1,920976355 | 0,165749067 | 0,281396712 |
| b1770 | 759 | 0,42 | -0,369619082 | 5,670606116 | 1,919257713 | 0,165938513 | 0,281611668 |
| b0762 | 150 | 0,41 | 0,313777962 | 5,575507568 | 1,918018528 | 0,166075261 | 0,281737063 |
| b4037 | 921 | 0,53 | -0,794641847 | 8,491603058 | 1,914725965 | 0,166439234 | 0,282247692 |
| b0254 | 894 | 0,48 | 0,477094321 | 3,355710787 | 1,912353502 | 0,166702062 | 0,282585729 |
| b3332 | 984 | 0,5 | -0,482914886 | 3,481930382 | 1,911788987 | 0,16676467 | 0,282585729 |
| b2266 | 306 | 0,52 | -0,351788877 | 10,36244186 | 1,910360614 | 0,166923207 | 0,282747472 |
| b4324 | 774 | 0,54 | -0,336538676 | 6,292365365 | 1,909666472 | 0,167000312 | 0,282771213 |
| b0046 | 531 | 0,55 | 0,448520931 | 4,309528486 | 1,907083631 | 0,167287574 | 0,283150644 |
| b3707 | 75 | 0,35 | 1,516245613 | 0,530974521 | 1,904988408 | 0,167521019 | 0,283348805 |
| b4439 | 93 | 0,35 | 1,519386374 | 0,601003774 | 1,904898117 | 0,167531087 | 0,283348805 |
| b3095 | 663 | 0,51 | -0,292013049 | 5,934235919 | 1,901086545 | 0,16795675 | 0,283928078 |
| b1796 | 183 | 0,46 | 0,802873821 | 2,169112567 | 1,900697373 | 0,168000282 | 0,283928078 |
| b3526 | 930 | 0,57 | -0,335822286 | 6,385810419 | 1,895964606 | 0,168530704 | 0,284717157 |
| b0875 | 696 | 0,52 | -0,503581861 | 4,131513512 | 1,888270664 | 0,169397103 | 0,286073027 |
| b3378 | 354 | 0,47 | 0,641718207 | 2,320368363 | 1,879696319 | 0,170368667 | 0,287605411 |
| b1663 | 1374 | 0,53 | -0,289077784 | 6,822692414 | 1,876419673 | 0,170741634 | 0,288126507 |
| b2061 | 444 | 0,55 | 1,190302784 | 0,698755622 | 1,871627934 | 0,171288747 | 0,288940972 |
| b0283 | 957 | 0,6 | 0,477287164 | 4,473273292 | 1,866788892 | 0,171843305 | 0,289767379 |
| b3065 | 216 | 0,52 | 0,565527693 | 7,219729254 | 1,860373873 | 0,172581656 | 0,290902965 |
| b2990 | 249 | 0,52 | -0,496891241 | 3,652437825 | 1,856664873 | 0,173010216 | 0,291515709 |
| b3430 | 1296 | 0,53 | -0,410993703 | 9,899625283 | 1,85199227 | 0,173551859 | 0,292318464 |
| b1981 | 1317 | 0,5 | 0,386186544 | 5,278160004 | 1,850636101 | 0,17370943 | 0,292380948 |
| b0235 | 207 | 0,51 | 1,498310017 | 0,561069634 | 1,850550124 | 0,173719425 | 0,292380948 |
| b3328 | 438 | 0,49 | 0,866332377 | 1,245211996 | 1,849588619 | 0,173831248 | 0,292388707 |
| b2446 | 417 | 0,49 | 0,827006602 | 2,392345175 | 1,849206169 | 0,17387575 | 0,292388707 |
| b2279 | 303 | 0,55 | 0,617856346 | 7,405798231 | 1,848828193 | 0,173919744 | 0,292388707 |
| b3146 | 861 | 0,55 | 0,386104396 | 4,871048335 | 1,843088454 | 0,174589393 | 0,293382582 |
| b3402 | 1725 | 0,5 | -0,277921678 | 5,685884164 | 1,842639978 | 0,174641841 | 0,293382582 |
| b3487 | 1068 | 0,58 | 0,308132965 | 8,036124088 | 1,840995676 | 0,174834293 | 0,293546968 |
| b3432 | 2187 | 0,53 | -0,3483091 | 9,981565206 | 1,840684986 | 0,174870684 | 0,293546968 |
| b4506 | 141 | 0,42 | 1,394702036 | 0,670264745 | 1,835723272 | 0,175453034 | 0,294402258 |
| b0269 | 1968 | 0,67 | 0,308570803 | 6,036892814 | 1,835088374 | 0,175527713 | 0,294402258 |
| b1926 | 366 | 0,52 | -1,137400458 | 1,253316261 | 1,834667388 | 0,17557725 | 0,294402258 |
| b2211 | 1644 | 0,53 | 0,551975111 | 4,149374039 | 1,833024156 | 0,175770765 | 0,294512434 |
| b0386 | 810 | 0,55 | 0,270691516 | 7,101266668 | 1,832993496 | 0,175774377 | 0,294512434 |
| b0614 | 552 | 0,58 | 1,179409651 | 0,987000186 | 1,829192368 | 0,176222969 | 0,295096341 |
| b2282 | 978 | 0,54 | 0,618039649 | 9,791934984 | 1,828925165 | 0,176254552 | 0,295096341 |
| b3753 | 993 | 0,51 | 0,310318574 | 5,672448193 | 1,822318241 | 0,177037578 | 0,296296648 |
| b0893 | 1293 | 0,53 | -0,336841384 | 9,869385214 | 1,821682109 | 0,177113181 | 0,296312534 |
| b3918 | 756 | 0,49 | 0,489677214 | 3,272567202 | 1,814253007 | 0,177998883 | 0,297633137 |
| b2969 | 963 | 0,57 | 0,346005045 | 4,377837587 | 1,813948039 | 0,17803535 | 0,297633137 |
| b3915 | 903 | 0,52 | -0,231363382 | 6,771122675 | 1,811872137 | 0,178283812 | 0,297937378 |
| b2195 | 558 | 0,54 | -0,735301459 | 4,570117574 | 1,810377472 | 0,178462955 | 0,298125593 |
| b0811 | 747 | 0,49 | 0,32130805 | 8,660532613 | 1,806453976 | 0,178934195 | 0,298801439 |
| b4651 | 365 | 0,36 | 1,301442535 | 0,886264261 | 1,801313573 | 0,179553771 | 0,299724396 |
| b1255 | 744 | 0,49 | 0,343845094 | 5,431279177 | 1,800142989 | 0,179695209 | 0,299810231 |
| b2412 | 987 | 0,55 | -0,304927017 | 8,766603222 | 1,799780975 | 0,179738976 | 0,299810231 |
| b1622 | 1173 | 0,51 | -0,37501936 | 5,83956399 | 1,79294426 | 0,180567845 | 0,30108076 |
| b1862 | 603 | 0,44 | 0,875475958 | 1,850465154 | 1,788749069 | 0,181078651 | 0,301769638 |
| b4471 | 1365 | 0,53 | -0,718185349 | 4,069271061 | 1,78844576 | 0,181115646 | 0,301769638 |
| b0256 | 1152 | 0,46 | -0,408586774 | 3,96366879 | 1,787721768 | 0,181203989 | 0,301804638 |
| b1303 | 978 | 0,52 | -0,315454861 | 4,827445698 | 1,786522122 | 0,181350483 | 0,301936428 |
| b2560 | 636 | 0,52 | -0,266804031 | 6,029216247 | 1,785113133 | 0,181522715 | 0,302110957 |
| b1118 | 1245 | 0,52 | 0,237495027 | 7,030644018 | 1,782754956 | 0,181811397 | 0,302479095 |
| b1424 | 1656 | 0,51 | 0,263231739 | 6,852649765 | 1,771791507 | 0,183160521 | 0,304532411 |
| b0161 | 1425 | 0,54 | -0,473834095 | 8,623809947 | 1,771621953 | 0,183181477 | 0,304532411 |
| b2756 | 600 | 0,45 | 0,459649811 | 3,199564539 | 1,770482 | 0,183322439 | 0,304653753 |
| b0805 | 2283 | 0,54 | 0,600055183 | 3,680437972 | 1,768801232 | 0,183530506 | 0,304886481 |
| b0092 | 921 | 0,52 | -0,302853925 | 8,123280176 | 1,762364652 | 0,184329843 | 0,306100911 |
| b3134 | 375 | 0,58 | -0,53852665 | 2,646280356 | 1,760630709 | 0,184545866 | 0,306346138 |
| b0952 | 564 | 0,52 | 0,336663803 | 5,681646048 | 1,753996628 | 0,185375088 | 0,307608718 |
| b4637 | 87 | 0,4 | 1,571991106 | 0,582591734 | 1,7529756 | 0,185503095 | 0,307707206 |
| b1156 | 603 | 0,48 | 0,760030414 | 1,868532056 | 1,751294437 | 0,185714086 | 0,307943224 |
| b2775 | 1278 | 0,5 | 0,457191275 | 3,005033998 | 1,746941328 | 0,186261712 | 0,308737054 |
| b0327 | 246 | 0,47 | 0,905200773 | 1,417160527 | 1,743988834 | 0,186634208 | 0,309240118 |
| b0416 | 420 | 0,54 | 0,260773006 | 6,927080824 | 1,742238097 | 0,186855495 | 0,309492361 |
| b3358 | 2103 | 0,53 | 0,287294245 | 6,471019428 | 1,736268565 | 0,18761232 | 0,310631111 |
| b1722 | 759 | 0,49 | 0,671239811 | 3,1631141 | 1,732398876 | 0,188104829 | 0,311331552 |
| b2784 | 2235 | 0,54 | -0,252496898 | 8,780141041 | 1,731433105 | 0,18822798 | 0,311420379 |
| b3686 | 429 | 0,49 | 1,320986771 | 5,150069585 | 1,72501093 | 0,189049304 | 0,31266383 |
| b4115 | 1338 | 0,54 | 0,42148979 | 3,540299105 | 1,721948464 | 0,189442428 | 0,313198437 |
| b3577 | 474 | 0,44 | 1,199900078 | 0,727399424 | 1,710730916 | 0,190890565 | 0,315476222 |
| b1685 | 189 | 0,44 | 0,504854876 | 3,281655793 | 1,710038126 | 0,190980424 | 0,315508389 |
| b1790 | 822 | 0,5 | 0,365410023 | 4,287760349 | 1,700245109 | 0,192255925 | 0,317498547 |
| b0501 | 491 | 0,33 | 1,049384099 | 1,016657229 | 1,699151956 | 0,19239892 | 0,317593742 |
| b2804 | 423 | 0,49 | 0,321380618 | 5,652618414 | 1,698721295 | 0,192455289 | 0,317593742 |
| b4125 | 1632 | 0,5 | -0,276184206 | 5,94675934 | 1,697060544 | 0,192672843 | 0,317835731 |
| b2078 | 1404 | 0,54 | 0,384893389 | 4,070952846 | 1,693629808 | 0,193123171 | 0,318461388 |
| b3133 | 474 | 0,51 | -0,539730622 | 3,520911998 | 1,690505798 | 0,193534307 | 0,319021981 |
| b0928 | 1191 | 0,52 | 0,402721908 | 9,428113594 | 1,6791354 | 0,195039377 | 0,321384738 |
| b1426 | 225 | 0,41 | 0,328478893 | 7,450419047 | 1,677791791 | 0,19521813 | 0,321503677 |
| b0318 | 606 | 0,45 | -0,474691066 | 3,363627065 | 1,67751467 | 0,195255022 | 0,321503677 |

| | | | | | | | |
|---|---|---|---|---|---|---|---|
| b1639 | 330 | 0,46 | 0,398896876 | 3,468588315 | 1,676607177 | 0,19537589 | 0,321584553 |
| b3765 | 1521 | 0,51 | -0,378414197 | 3,521477302 | 1,675890048 | 0,195471465 | 0,321623754 |
| b2679 | 993 | 0,53 | -0,425917597 | 6,106801417 | 1,673432959 | 0,195799348 | 0,32204502 |
| b3140 | 792 | 0,49 | 0,689186287 | 1,908718957 | 1,670999454 | 0,196124719 | 0,322461846 |
| b1070 | 417 | 0,48 | 0,515948258 | 2,61399412 | 1,659047267 | 0,197732009 | 0,324985282 |
| b3658 | 95 | 0,64 | 1,235991513 | 0,642083604 | 1,652253652 | 0,198652474 | 0,326317854 |
| b3098 | 306 | 0,55 | -0,306808617 | 10,01517067 | 1,651989223 | 0,198688403 | 0,326317854 |
| b2754 | 285 | 0,46 | 0,522765034 | 2,644497913 | 1,649484929 | 0,19902905 | 0,326757582 |
| b1974 | 531 | 0,42 | 0,698197978 | 1,95703941 | 1,644928748 | 0,199650563 | 0,327541075 |
| b2162 | 942 | 0,52 | -0,680480793 | 1,8904813 | 1,64491505 | 0,199652435 | 0,327541075 |
| b1874 | 747 | 0,52 | 0,323762665 | 6,012984016 | 1,643612984 | 0,199830472 | 0,3277132 |
| b2470 | 3114 | 0,54 | 0,327270369 | 4,956742359 | 1,642620186 | 0,199966347 | 0,327816082 |
| b0942 | 543 | 0,47 | 0,668132808 | 1,953539114 | 1,639641057 | 0,200374724 | 0,328365452 |
| b3895 | 834 | 0,53 | 0,330046488 | 5,359061284 | 1,630714469 | 0,201604256 | 0,330200558 |
| b1816 | 1557 | 0,54 | -0,2686962 | 6,796424665 | 1,630442288 | 0,201641885 | 0,330200558 |
| b3544 | 1608 | 0,52 | 0,462343568 | 11,61094679 | 1,627137405 | 0,202099443 | 0,330828965 |
| b3131 | 810 | 0,52 | 0,288642886 | 6,375180146 | 1,625072313 | 0,202385974 | 0,331098603 |
| b1723 | 930 | 0,54 | 0,26088016 | 8,414399909 | 1,624885568 | 0,202411908 | 0,331098603 |
| b4394 | 513 | 0,56 | 0,417252032 | 4,192290475 | 1,621579097 | 0,202871746 | 0,33172972 |
| b1464 | 894 | 0,52 | 0,276764433 | 5,729296233 | 1,607030749 | 0,20490969 | 0,334939909 |
| b3481 | 402 | 0,57 | -0,425876077 | 4,356495557 | 1,604858496 | 0,205216048 | 0,335318384 |
| b4596 | 174 | 0,48 | 0,421540239 | 3,147347294 | 1,602288853 | 0,205579148 | 0,335789265 |
| b2962 | 276 | 0,47 | -0,365452383 | 6,05977206 | 1,600426145 | 0,20584283 | 0,336089589 |
| b2879 | 1329 | 0,53 | 0,839237305 | 2,343459213 | 1,599592813 | 0,205960925 | 0,336089589 |
| b4178 | 426 | 0,48 | -0,282029508 | 6,305171396 | 1,599402015 | 0,205987975 | 0,336089589 |
| b0603 | 903 | 0,39 | 0,762026697 | 1,907227249 | 1,594975514 | 0,20661671 | 0,336992756 |
| b0598 | 2106 | 0,57 | 0,457321676 | 10,39429409 | 1,590332077 | 0,207278696 | 0,337843853 |
| b0546 | 798 | 0,36 | -0,355302613 | 4,733376521 | 1,589961293 | 0,207331664 | 0,337843853 |
| b2339 | 564 | 0,46 | 1,291571951 | 0,772133512 | 1,589730324 | 0,207364667 | 0,337843853 |
| b1494 | 2796 | 0,47 | 0,422261784 | 4,215851698 | 1,585041833 | 0,20803595 | 0,338807155 |
| b3086 | 693 | 0,52 | 0,764562022 | 1,712213757 | 1,584545827 | 0,208107117 | 0,338807155 |
| b1563 | 288 | 0,47 | 0,277571232 | 6,213724342 | 1,582989919 | 0,208330545 | 0,33904775 |
| b2903 | 2874 | 0,56 | 0,353411307 | 11,76111308 | 1,579692183 | 0,208805038 | 0,339696616 |
| b2305 | 891 | 0,46 | 0,463951312 | 3,987405761 | 1,574881192 | 0,20949956 | 0,34070284 |
| b0764 | 690 | 0,54 | 0,288671188 | 4,838146496 | 1,569710798 | 0,210249018 | 0,341770192 |
| b2865 | 756 | 0,51 | -0,228993953 | 6,239632038 | 1,569302154 | 0,210308387 | 0,341770192 |
| b1432 | 1149 | 0,52 | -0,288986903 | 5,883312047 | 1,564898498 | 0,210949425 | 0,342569997 |
| b1604 | 945 | 0,51 | 0,309006157 | 7,56987236 | 1,564871161 | 0,210953412 | 0,342569997 |
| b4067 | 1650 | 0,56 | 0,745902962 | 8,814363544 | 1,564098657 | 0,211066106 | 0,342628861 |
| b4464 | 1368 | 0,53 | -0,333849459 | 4,769386229 | 1,563525913 | 0,211149705 | 0,342640469 |
| b0038 | 1218 | 0,53 | 0,347607658 | 3,744871777 | 1,561077088 | 0,211507584 | 0,343096992 |
| b3178 | 1935 | 0,54 | 0,298159085 | 10,40493022 | 1,552175947 | 0,212814499 | 0,345085007 |
| b4265 | 1320 | 0,51 | -0,381451688 | 4,219684611 | 1,551683291 | 0,212887114 | 0,345085007 |
| b4599 | 96 | 0,39 | 0,565604615 | 3,948996488 | 1,549929838 | 0,213145799 | 0,345328286 |
| b4028 | 738 | 0,59 | 0,928207819 | 1,09751133 | 1,549621739 | 0,213191291 | 0,345328286 |
| b0646 | 1428 | 0,47 | 0,646728441 | 2,268894264 | 1,547735145 | 0,213470106 | 0,345654992 |
| b0153 | 1983 | 0,6 | 0,498380298 | 4,129531778 | 1,546446111 | 0,213660859 | 0,345805843 |
| b3684 | 717 | 0,51 | 0,76880783 | 1,844445649 | 1,546063088 | 0,213717578 | 0,345805843 |
| b4029 | 2097 | 0,54 | 0,425329303 | 4,441244251 | 1,544032863 | 0,214018518 | 0,346167809 |
| b1456 | 2037 | 0,58 | 0,537115795 | 2,328180428 | 1,543080627 | 0,214159842 | 0,346271433 |
| b0272 | 759 | 0,63 | 0,390565849 | 4,718899433 | 1,530913435 | 0,215975406 | 0,349081057 |
| b0770 | 1434 | 0,5 | 0,717749301 | 1,61079253 | 1,529297263 | 0,216217944 | 0,349170531 |
| b3892 | 636 | 0,53 | -0,408492352 | 9,117048851 | 1,529138787 | 0,216241743 | 0,349170531 |
| b1467 | 1545 | 0,54 | -0,477219483 | 7,989944136 | 1,528987419 | 0,216264479 | 0,349170531 |
| b2914 | 660 | 0,52 | -0,279735986 | 8,534281955 | 1,524420546 | 0,216951757 | 0,350154041 |
| b1764 | 1044 | 0,55 | -0,268600932 | 8,735668858 | 1,52182323 | 0,217343793 | 0,350660503 |
| b0144 | 927 | 0,54 | 0,307922937 | 5,101255243 | 1,52033883 | 0,217568225 | 0,350809621 |
| b0210 | 624 | 0,49 | -0,463314543 | 4,626586749 | 1,520176676 | 0,217592759 | 0,350809621 |
| b3456 | 1278 | 0,57 | 0,516188701 | 2,844885001 | 1,518735532 | 0,217810945 | 0,351035116 |
| b2753 | 1038 | 0,49 | 0,344359234 | 4,305752783 | 1,517084734 | 0,218061194 | 0,351312103 |
| b4606 | 60 | 0,38 | 1,259772206 | 0,487890955 | 1,516407226 | 0,218163998 | 0,351351433 |
| b3345 | 387 | 0,55 | -0,253725778 | 5,412648249 | 1,515283752 | 0,2183346 | 0,351499883 |
| b3429 | 1434 | 0,55 | -0,326667137 | 9,589656741 | 1,513146847 | 0,218659533 | 0,351896598 |
| b4445 | 82 | 0,48 | 1,444340787 | 0,454834111 | 1,511591016 | 0,218896472 | 0,352130911 |
| b2170 | 1182 | 0,53 | 0,497937852 | 3,114821827 | 1,511159383 | 0,21896226 | 0,352130911 |
| b2067 | 3318 | 0,52 | 0,273222614 | 6,604753622 | 1,510550398 | 0,21905512 | 0,352153891 |
| b4204 | 795 | 0,42 | 0,696286811 | 1,790136765 | 1,507851868 | 0,219467165 | 0,352689794 |
| b4184 | 399 | 0,44 | 1,256704627 | 0,860536901 | 1,499756278 | 0,220708866 | 0,354558114 |
| b3534 | 753 | 0,54 | -0,445754926 | 4,237043122 | 1,494091476 | 0,221582728 | 0,355834391 |
| b3512 | 528 | 0,33 | -0,519788176 | 9,569141951 | 1,491209805 | 0,222028847 | 0,356423098 |
| b3524 | 2061 | 0,53 | -0,311179689 | 8,112125853 | 1,490486122 | 0,222141051 | 0,356475542 |
| b4017 | 2187 | 0,38 | 0,472346194 | 4,776701171 | 1,488914865 | 0,222384901 | 0,356739129 |
| b4015 | 1305 | 0,55 | -0,36133334 | 13,99158794 | 1,484627864 | 0,223051851 | 0,357477536 |
| b3976 | 76 | 0,51 | 1,242632283 | 0,7277354 | 1,483685472 | 0,223198785 | 0,357477536 |
| b0695 | 2685 | 0,58 | -0,312498671 | 7,489016562 | 1,483618567 | 0,223209221 | 0,357477536 |
| b4432 | 249 | 0,46 | 0,472657017 | 6,725646663 | 1,483504376 | 0,223227034 | 0,357477536 |
| b0768 | 954 | 0,45 | 0,51598602 | 2,898174987 | 1,483054463 | 0,223297234 | 0,357477536 |
| b0623 | 210 | 0,44 | 0,384770741 | 10,25180498 | 1,482884491 | 0,223323762 | 0,357477536 |
| b1157 | 501 | 0,47 | 1,245164296 | 0,735811882 | 1,482082359 | 0,223449002 | 0,357522042 |
| b1765 | 552 | 0,56 | 0,267038598 | 7,46146624 | 1,481684819 | 0,223511103 | 0,357522042 |
| b4467 | 1224 | 0,56 | -1,070717968 | 9,062609332 | 1,481120906 | 0,223599228 | 0,357535406 |
| b4001 | 696 | 0,53 | 0,293558697 | 5,433450632 | 1,474586381 | 0,224623458 | 0,359045057 |
| b1695 | 1152 | 0,52 | 0,734002042 | 2,008561096 | 1,473096095 | 0,224857834 | 0,359291555 |
| b1810 | 300 | 0,45 | 0,297366915 | 6,194664739 | 1,468031045 | 0,225656608 | 0,360439385 |
| b2097 | 1053 | 0,51 | -0,312262101 | 10,54212423 | 1,465408571 | 0,226071517 | 0,36092397 |
| b2346 | 756 | 0,52 | 0,222938819 | 7,403948525 | 1,464787219 | 0,226169957 | 0,36092397 |
| b3216 | 2382 | 0,46 | -0,328187062 | 4,886755725 | 1,464587757 | 0,226201569 | 0,36092397 |
| b3331 | 588 | 0,5 | 0,713696474 | 1,898250968 | 1,460716541 | 0,226816146 | 0,361775789 |
| b3113 | 390 | 0,53 | -0,635943028 | 3,157339898 | 1,456599916 | 0,227471885 | 0,362692633 |
| b1358 | 423 | 0,51 | 0,611096241 | 2,372464241 | 1,453904388 | 0,227902492 | 0,363131426 |
| b1446 | 234 | 0,54 | 0,260533751 | 5,755289367 | 1,453862911 | 0,227909126 | 0,363131426 |
| b0691 | 651 | 0,44 | 1,233743687 | 0,867732076 | 1,451905294 | 0,228222478 | 0,363153144 |
| b3708 | 1416 | 0,51 | 0,650228816 | 12,34398307 | 1,451473061 | 0,228291734 | 0,363153144 |
| b4065 | 1650 | 0,56 | -0,223803309 | 6,77019617 | 1,451433989 | 0,228297996 | 0,363153144 |

| | | | | | | | |
|---|---|---|---|---|---|---|---|
| b0160 | 1518 | 0,48 | 0,283997733 | 6,110440893 | 1,451269315 | 0,228324389 | 0,363153144 |
| b0403 | 1815 | 0,55 | 0,30309737 | 5,577255963 | 1,450856869 | 0,22839051 | 0,363153144 |
| b3825 | 1023 | 0,54 | -0,232708056 | 6,757209208 | 1,450742151 | 0,228408905 | 0,363153144 |
| b0289 | 711 | 0,53 | 0,440613115 | 3,415945764 | 1,436573238 | 0,230694671 | 0,366657275 |
| b0645 | 708 | 0,46 | 1,414157895 | 0,908266736 | 1,434024862 | 0,2311087 | 0,36718511 |
| b0438 | 1275 | 0,52 | 0,279686457 | 10,23221554 | 1,431562011 | 0,231509687 | 0,367691855 |
| b1468 | 3741 | 0,55 | -0,401996763 | 9,256980982 | 1,42770859 | 0,232138762 | 0,36852636 |
| b0412 | 540 | 0,48 | 0,467008141 | 3,64926744 | 1,427055575 | 0,232245572 | 0,36852636 |
| b1381 | 2640 | 0,52 | -0,233444601 | 6,355702473 | 1,426834247 | 0,232281787 | 0,36852636 |
| b3120 | 540 | 0,36 | 0,791449901 | 1,159233945 | 1,422853382 | 0,232934321 | 0,369430865 |
| b0188 | 1299 | 0,58 | 0,272029314 | 5,919416395 | 1,419949565 | 0,233411705 | 0,369933094 |
| b1289 | 354 | 0,5 | -0,374175337 | 3,391468933 | 1,419923095 | 0,233416062 | 0,369933094 |
| b0682 | 327 | 0,48 | 0,516434681 | 2,716507449 | 1,419063764 | 0,233557564 | 0,370026513 |
| b3678 | 1494 | 0,52 | 0,391834841 | 3,079785573 | 1,413938296 | 0,234403712 | 0,371235844 |
| b1392 | 1071 | 0,53 | -0,809232878 | 5,658784769 | 1,412174683 | 0,234695719 | 0,371500867 |
| b2574 | 1623 | 0,53 | 0,400896322 | 3,296852038 | 1,41192662 | 0,234736827 | 0,371500867 |
| b2546 | 999 | 0,56 | 0,515052282 | 2,604507656 | 1,411001125 | 0,234890272 | 0,371612495 |
| b2245 | 804 | 0,54 | 0,441581663 | 3,327213927 | 1,408362391 | 0,235328436 | 0,372109471 |
| b3920 | 600 | 0,49 | 0,429385555 | 4,506890749 | 1,407776208 | 0,235425906 | 0,372109471 |
| b0534 | 516 | 0,5 | 1,084417855 | 0,868654985 | 1,407331317 | 0,235499915 | 0,372109471 |
| b1183 | 420 | 0,48 | -0,482880838 | 3,298956901 | 1,40711149 | 0,235536495 | 0,372109471 |
| b0615 | 1533 | 0,56 | 0,608426872 | 1,931115201 | 1,404206367 | 0,236020555 | 0,372742822 |
| b1098 | 642 | 0,54 | 0,237715834 | 5,394093719 | 1,400135443 | 0,236700894 | 0,373685597 |
| b0191 | 423 | 0,54 | 0,310202865 | 4,557742809 | 1,39858367 | 0,236960854 | 0,373964278 |
| b0297 | 872 | 0,48 | 1,206482506 | 0,735205744 | 1,396098945 | 0,237377828 | 0,37437187 |
| b0736 | 405 | 0,5 | 0,345886024 | 5,239772591 | 1,396049233 | 0,237386179 | 0,37437187 |
| b1550 | 174 | 0,3 | -0,495762183 | 4,895427669 | 1,395284942 | 0,237514623 | 0,37444268 |
| b1664 | 1257 | 0,51 | 0,247222961 | 9,206310189 | 1,394685569 | 0,23761541 | 0,374469855 |
| b4580 | 4168 | 0,45 | -0,277201409 | 6,033013925 | 1,393386201 | 0,237834083 | 0,374530708 |
| b3061 | 912 | 0,54 | -0,736064103 | 4,188543819 | 1,393124275 | 0,237878192 | 0,374530708 |
| b3051 | 1662 | 0,5 | 0,431248408 | 3,068199616 | 1,392966819 | 0,237904713 | 0,374530708 |
| b4249 | 714 | 0,54 | 0,501010952 | 2,764083582 | 1,392263384 | 0,23802324 | 0,374585731 |
| b3228 | 498 | 0,55 | 0,238303441 | 7,378129707 | 1,387871816 | 0,238764828 | 0,375510489 |
| b2250 | 543 | 0,53 | 0,459873114 | 4,075311917 | 1,387392689 | 0,238845906 | 0,375510489 |
| b4555 | 294 | 0,43 | 0,528306134 | 2,929846567 | 1,387296394 | 0,238862205 | 0,375510489 |
| b3830 | 816 | 0,55 | -0,30417736 | 7,661179406 | 1,38525828 | 0,239207498 | 0,37592146 |
| b1306 | 360 | 0,5 | 0,390189688 | 4,487591072 | 1,38357757 | 0,239492696 | 0,376237737 |
| b1856 | 1323 | 0,52 | 0,262282935 | 6,746239311 | 1,380644119 | 0,239991461 | 0,376889183 |
| b1159 | 834 | 0,38 | 0,571706394 | 2,491472426 | 1,377737135 | 0,240486972 | 0,377391441 |
| b2774 | 786 | 0,46 | 0,812634704 | 1,525170045 | 1,377486896 | 0,240529685 | 0,377391441 |
| b0266 | 309 | 0,52 | 0,425282706 | 3,486014905 | 1,377286549 | 0,240563888 | 0,377391441 |
| b0147 | 531 | 0,56 | 0,452527868 | 3,673788298 | 1,37254608 | 0,241374913 | 0,378531267 |
| b2830 | 531 | 0,49 | 0,229964741 | 7,603456077 | 1,369501343 | 0,241897578 | 0,379192103 |
| b4588 | 102 | 0,49 | 1,37335258 | 0,488116829 | 1,369106203 | 0,24196551 | 0,379192103 |
| b3804 | 741 | 0,53 | -0,200076054 | 7,119454944 | 1,367888278 | 0,242175038 | 0,379387809 |
| b0944 | 711 | 0,52 | 0,357428855 | 3,464071124 | 1,363772572 | 0,24288473 | 0,380366653 |
| b3627 | 1020 | 0,36 | -0,256799125 | 5,788052574 | 1,36170227 | 0,243242681 | 0,380787073 |
| b1418 | 531 | 0,48 | 0,323137522 | 5,360056023 | 1,361237603 | 0,243323109 | 0,380787073 |
| b0508 | 777 | 0,45 | -1,135289538 | 3,443364985 | 1,360554979 | 0,243441322 | 0,380839094 |
| b0043 | 1287 | 0,55 | 0,622292522 | 2,494908663 | 1,357911276 | 0,243899804 | 0,381339322 |
| b1798 | 639 | 0,45 | 0,513342578 | 3,753503259 | 1,357540969 | 0,243964108 | 0,381339322 |
| b4140 | 477 | 0,47 | 0,536913971 | 5,590254511 | 1,357240349 | 0,244016326 | 0,381339322 |
| b2577 | 882 | 0,5 | -0,334573516 | 4,257510349 | 1,355782744 | 0,244269708 | 0,381602242 |
| b3322 | 420 | 0,46 | 0,490912694 | 2,767440146 | 1,353782892 | 0,244617874 | 0,382013 |
| b3719 | 1170 | 0,5 | 0,555278357 | 2,588266084 | 1,351389069 | 0,245035425 | 0,382531791 |
| b0377 | 1221 | 0,51 | -0,320353863 | 4,988910525 | 1,350011692 | 0,245276073 | 0,38277415 |
| b0019 | 1167 | 0,51 | -0,277328753 | 8,473991089 | 1,348454355 | 0,24554851 | 0,383065932 |
| b1059 | 1119 | 0,52 | 0,238070167 | 6,032161985 | 1,342810761 | 0,246538892 | 0,384477145 |
| b4205 | 402 | 0,39 | -0,450143319 | 3,24854438 | 1,342265993 | 0,246634751 | 0,384492853 |
| b4216 | 555 | 0,52 | -0,229472541 | 6,777902978 | 1,341442997 | 0,246779653 | 0,384584981 |
| b1642 | 435 | 0,48 | 0,216835226 | 6,947862111 | 1,340187161 | 0,247000965 | 0,384796081 |
| b0479 | 1221 | 0,52 | 0,346378202 | 4,180864236 | 1,33966423 | 0,247093191 | 0,384806004 |
| b0069 | 1656 | 0,55 | -0,243832975 | 5,244120674 | 1,339053887 | 0,247200886 | 0,384840004 |
| b2515 | 1119 | 0,53 | 0,240270933 | 8,46681329 | 1,337532565 | 0,247469574 | 0,385124525 |
| b3135 | 555 | 0,58 | -0,345181723 | 3,537964462 | 1,331868386 | 0,248473102 | 0,386552045 |
| b1196 | 441 | 0,41 | -0,526168655 | 2,778739599 | 1,330018962 | 0,248801844 | 0,38692917 |
| b4172 | 309 | 0,49 | 0,231168079 | 8,961042853 | 1,326647475 | 0,249402512 | 0,387728775 |
| b1393 | 768 | 0,55 | -0,872328643 | 5,019735302 | 1,320624712 | 0,250479965 | 0,389172466 |
| b3207 | 633 | 0,51 | 0,279493056 | 8,984352309 | 1,3204862 | 0,250504812 | 0,389172466 |
| b0167 | 2673 | 0,54 | 0,21012343 | 6,938486184 | 1,318659141 | 0,250832835 | 0,389547043 |
| b3521 | 900 | 0,53 | 0,4357668 | 3,782463948 | 1,317806339 | 0,250986124 | 0,389562903 |
| b0449 | 1782 | 0,56 | 0,291095209 | 4,878081748 | 1,317302362 | 0,251076767 | 0,389562903 |
| b4171 | 951 | 0,53 | 0,285938674 | 9,943046646 | 1,317152106 | 0,251103799 | 0,389562903 |
| b2578 | 588 | 0,51 | 0,409366349 | 3,221994596 | 1,316468292 | 0,251226867 | 0,389618968 |
| b4236 | 366 | 0,49 | 0,266083873 | 4,912395817 | 1,315171533 | 0,251460451 | 0,389846331 |
| b3857 | 585 | 0,5 | -0,309787425 | 4,7710006 | 1,313935602 | 0,251683328 | 0,390026659 |
| b4697 | 143 | 0,42 | -0,590923342 | 2,279421784 | 1,313561659 | 0,251750809 | 0,390026659 |
| b3579 | 987 | 0,51 | 0,51623074 | 2,365321887 | 1,308343844 | 0,25269473 | 0,391247936 |
| b2054 | 549 | 0,48 | 1,345792233 | 0,709595694 | 1,308239266 | 0,252713694 | 0,391247936 |
| b4458 | 110 | 0,49 | 1,341572101 | 0,509330308 | 1,306445327 | 0,253039258 | 0,391616697 |
| b2519 | 2313 | 0,57 | -0,279573392 | 5,018565799 | 1,305402231 | 0,253228797 | 0,391774756 |
| b2072 | 762 | 0,52 | 0,693135175 | 1,426485484 | 1,301922702 | 0,253862318 | 0,392619362 |
| b2068 | 849 | 0,56 | -0,312568567 | 4,783507093 | 1,300533199 | 0,254115852 | 0,392757756 |
| b2251 | 426 | 0,48 | -0,426796347 | 3,364865259 | 1,300210234 | 0,254174826 | 0,392757756 |
| b2473 | 699 | 0,54 | -0,244501894 | 6,460672762 | 1,299700707 | 0,2542679 | 0,392757756 |
| b0503 | 1095 | 0,56 | -0,323943095 | 4,257089902 | 1,299512325 | 0,254302322 | 0,392757756 |
| b2033 | 591 | 0,41 | 0,365930389 | 6,479287386 | 1,298317762 | 0,254520732 | 0,39295967 |
| b4517 | 174 | 0,4 | 0,517852055 | 3,322568024 | 1,296428097 | 0,254866705 | 0,393358324 |
| b4259 | 444 | 0,54 | 0,212677635 | 6,24506186 | 1,291390578 | 0,255791843 | 0,394650272 |
| b1877 | 489 | 0,39 | 0,823390724 | 1,387227058 | 1,288138072 | 0,256391364 | 0,395360169 |
| b1738 | 321 | 0,44 | 0,302989271 | 4,264290126 | 1,287937547 | 0,256428383 | 0,395360169 |
| b4474 | 831 | 0,55 | 0,436823306 | 2,792491609 | 1,286066326 | 0,256774144 | 0,395757123 |
| b1454 | 618 | 0,47 | -0,288782505 | 5,219997381 | 1,285053854 | 0,256961467 | 0,395909693 |
| b4108 | 336 | 0,46 | 0,27276901 | 4,833925457 | 1,284217619 | 0,257116311 | 0,396012132 |

| | | | | | | | |
|---|---|---|---|---|---|---|---|
| b2042 | 894 | 0,52 | -0,288407601 | 8,638977388 | 1,282711168 | 0,257395547 | 0,396306027 |
| b2083 | 213 | 0,48 | 0,948798159 | 1,271726786 | 1,277804863 | 0,258307583 | 0,397491043 |
| b0358 | 774 | 0,5 | -0,445756085 | 3,531035987 | 1,277617068 | 0,258342572 | 0,397491043 |
| b0094 | 1263 | 0,52 | -0,240675395 | 8,997959652 | 1,275011541 | 0,258828619 | 0,398102221 |
| b0750 | 1044 | 0,5 | -0,302996419 | 4,719857953 | 1,272994147 | 0,259205731 | 0,398545484 |
| b0490 | 678 | 0,45 | -0,264388706 | 5,012509358 | 1,270860163 | 0,259605376 | 0,399023078 |
| b1860 | 1011 | 0,53 | -0,202917362 | 6,385278147 | 1,268102693 | 0,260122915 | 0,39968149 |
| b1928 | 414 | 0,57 | 0,246452427 | 6,0680922 | 1,267343385 | 0,260265651 | 0,399701457 |
| b3689 | 1248 | 0,55 | -0,25706751 | 5,92881873 | 1,267084872 | 0,260314269 | 0,399701457 |
| b3878 | 2037 | 0,54 | 0,538040811 | 2,94257946 | 1,266218906 | 0,260477211 | 0,399728774 |
| b3147 | 2037 | 0,54 | 0,20259495 | 7,74046947 | 1,265967495 | 0,26052454 | 0,399728774 |
| b1370 | 981 | 0,55 | -0,389776536 | 4,320965083 | 1,265153613 | 0,260677832 | 0,399728774 |
| b3575 | 999 | 0,53 | 0,440018729 | 2,829536428 | 1,265095396 | 0,260688801 | 0,399728774 |
| b4145 | 870 | 0,45 | 0,344492323 | 4,145017546 | 1,263494959 | 0,260990578 | 0,400054641 |
| b2301 | 645 | 0,52 | 0,252178767 | 5,48873259 | 1,261391414 | 0,261387879 | 0,400526658 |
| b2335 | 513 | 0,49 | 0,713603403 | 1,577960724 | 1,260166401 | 0,261619596 | 0,400744712 |
| b0364 | 558 | 0,43 | 1,098487797 | 0,908702253 | 1,259397834 | 0,261765103 | 0,400830609 |
| b3100 | 300 | 0,57 | -0,273776088 | 9,861186903 | 1,257688531 | 0,262089074 | 0,401182207 |
| b4244 | 462 | 0,48 | 0,568220071 | 6,287674575 | 1,257242279 | 0,262173736 | 0,401182207 |
| b0390 | 678 | 0,46 | 0,393995849 | 4,470132844 | 1,256167494 | 0,26237778 | 0,40135741 |
| b0355 | 834 | 0,49 | 0,316782153 | 4,602490648 | 1,254411585 | 0,262711558 | 0,401610972 |
| b1322 | 1062 | 0,56 | 0,425492898 | 5,889666407 | 1,254352749 | 0,262722751 | 0,401610972 |
| b2352 | 1332 | 0,28 | 0,494918914 | 5,041234105 | 1,252156111 | 0,263141071 | 0,40211329 |
| b2821 | 2889 | 0,51 | 0,217653663 | 6,890385824 | 1,246767017 | 0,264170859 | 0,403549349 |
| b3137 | 861 | 0,52 | -0,70219177 | 2,017517839 | 1,244490701 | 0,264607338 | 0,404078395 |
| b1376 | 435 | 0,49 | -0,368498184 | 9,661592915 | 1,243149207 | 0,264864987 | 0,404334084 |
| b4093 | 435 | 0,58 | 0,377821706 | 4,412079987 | 1,242218853 | 0,265043855 | 0,404469376 |
| b0879 | 1947 | 0,54 | -0,224318005 | 6,484799355 | 1,240649931 | 0,265345833 | 0,404792384 |
| b0599 | 1089 | 0,55 | -0,30985888 | 6,682260198 | 1,236747222 | 0,266098866 | 0,405803033 |
| b3800 | 1236 | 0,53 | 0,319892203 | 3,396013896 | 1,234016676 | 0,26662731 | 0,406470613 |
| b1742 | 576 | 0,52 | 0,522596261 | 2,138331268 | 1,232618035 | 0,266898496 | 0,406745685 |
| b1106 | 825 | 0,53 | 0,23154063 | 5,535278719 | 1,231975599 | 0,267023175 | 0,406797373 |
| b2638 | 153 | 0,44 | 0,713749992 | 1,541107001 | 1,230053142 | 0,267396704 | 0,407133551 |
| b2408 | 765 | 0,45 | 0,410155716 | 3,686318926 | 1,22970572 | 0,267464276 | 0,407133551 |
| b4518 | 174 | 0,51 | 0,388616857 | 7,861811402 | 1,229048611 | 0,26759214 | 0,407133551 |
| b4546 | 219 | 0,52 | 0,441160172 | 3,509432264 | 1,228971278 | 0,267607193 | 0,407133551 |
| b0200 | 576 | 0,5 | -0,266093397 | 6,221074824 | 1,220830507 | 0,269197726 | 0,409301802 |
| b3979 | 76 | 0,54 | 1,288821331 | 0,509185644 | 1,220742332 | 0,269215018 | 0,409301802 |
| b3727 | 960 | 0,55 | 0,457047656 | 4,686348419 | 1,218868747 | 0,269582775 | 0,409721939 |
| b3900 | 447 | 0,5 | 1,125748114 | 0,868310776 | 1,215685578 | 0,270209024 | 0,410534524 |
| b3911 | 1374 | 0,55 | -0,217879495 | 7,84574084 | 1,213699602 | 0,270600661 | 0,410990228 |
| b2333 | 540 | 0,49 | 0,761717311 | 1,573444848 | 1,213015152 | 0,2707358 | 0,411056184 |
| b3513 | 1158 | 0,55 | 0,437592111 | 8,140225351 | 1,209332382 | 0,271464382 | 0,412022811 |
| b2770 | 780 | 0,56 | 0,462130886 | 2,335226774 | 1,207780071 | 0,271772219 | 0,412261109 |
| b3834 | 606 | 0,56 | -0,198565409 | 6,300104403 | 1,207613138 | 0,27180535 | 0,412261109 |
| b2006 | 168 | 0,49 | 1,00211005 | 1,112195891 | 1,201411851 | 0,273039677 | 0,413993178 |
| b3677 | 450 | 0,48 | 0,4739946 | 2,83872589 | 1,198118218 | 0,273698111 | 0,414669525 |
| b0595 | 858 | 0,55 | 0,794109379 | 3,147531728 | 1,198025258 | 0,273716723 | 0,414669525 |
| b1270 | 591 | 0,5 | 0,28751757 | 4,873718882 | 1,197792652 | 0,273763303 | 0,414669525 |
| b2988 | 1860 | 0,52 | 0,230774316 | 7,254133006 | 1,193569859 | 0,274610654 | 0,415732883 |
| b1249 | 1461 | 0,52 | 0,216800845 | 6,995068543 | 1,193190912 | 0,274686855 | 0,415732883 |
| b2755 | 918 | 0,51 | 0,353604872 | 4,134720489 | 1,192908824 | 0,274743596 | 0,415732883 |
| b1336 | 1527 | 0,49 | 0,375261545 | 3,037354208 | 1,190913853 | 0,275145299 | 0,416033094 |
| b0511 | 1455 | 0,47 | -0,783285982 | 1,956885507 | 1,19088293 | 0,275151531 | 0,416033094 |
| b1891 | 579 | 0,49 | 0,392230121 | 3,698343144 | 1,190540962 | 0,275220465 | 0,416033094 |
| b0637 | 318 | 0,51 | 0,339343863 | 5,084763931 | 1,18933727 | 0,275463277 | 0,416259747 |
| b3887 | 438 | 0,55 | -0,199613178 | 6,864835885 | 1,187888039 | 0,275755978 | 0,416561609 |
| b1250 | 1254 | 0,42 | -0,273458299 | 5,699444969 | 1,186170926 | 0,276103289 | 0,416813847 |
| b3596 | 462 | 0,32 | 1,054895528 | 0,859535428 | 1,18614296 | 0,27610895 | 0,416813847 |
| b4688 | 205 | 0,43 | 1,287700006 | 0,454907773 | 1,184702179 | 0,2764008 | 0,417031433 |
| b1993 | 546 | 0,51 | 0,307763724 | 4,453723604 | 1,184512871 | 0,276439176 | 0,417031433 |
| b4509 | 141 | 0,48 | 1,278865215 | 0,455283754 | 1,183779733 | 0,276587858 | 0,417115337 |
| b1389 | 288 | 0,5 | 0,89811855 | 3,736108468 | 1,182973675 | 0,276751445 | 0,417221653 |
| b0339 | 660 | 0,57 | 0,463906691 | 2,498822572 | 1,180485519 | 0,277257176 | 0,417843531 |
| b2226 | 1650 | 0,55 | 0,396241748 | 3,069671876 | 1,178008606 | 0,277761777 | 0,418463289 |
| b1803 | 966 | 0,5 | 0,458382804 | 2,992188569 | 1,17624687 | 0,278121385 | 0,418864263 |
| b2283 | 2727 | 0,56 | 0,459676202 | 11,39881105 | 1,174478774 | 0,27848288 | 0,41926781 |
| b1057 | 567 | 0,51 | 0,499025652 | 2,790441957 | 1,169706724 | 0,279461509 | 0,420599894 |
| b1731 | 243 | 0,52 | 0,579568121 | 3,308872766 | 1,166342642 | 0,280154005 | 0,421500588 |
| b0831 | 921 | 0,54 | -0,263391343 | 5,098701617 | 1,165061884 | 0,280418219 | 0,421756529 |
| b2620 | 483 | 0,52 | -0,224680014 | 6,51637082 | 1,162265852 | 0,280996118 | 0,422483932 |
| b2080 | 333 | 0,48 | -0,368716571 | 8,044016285 | 1,160564697 | 0,281348458 | 0,422871827 |
| b0273 | 1005 | 0,59 | 0,466783804 | 4,566361945 | 1,159557623 | 0,281557304 | 0,423043861 |
| b0459 | 552 | 0,48 | 0,364965699 | 5,480693849 | 1,153342453 | 0,282850549 | 0,424844557 |
| b3461 | 855 | 0,54 | 0,218305546 | 9,011025903 | 1,152191878 | 0,283090783 | 0,42492923 |
| b4690 | 75 | 0,56 | 1,252317092 | 0,444137069 | 1,152164329 | 0,283096538 | 0,42492923 |
| b3104 | 357 | 0,45 | 0,709667214 | 1,856901618 | 1,146096061 | 0,284367874 | 0,426694614 |
| b1922 | 720 | 0,54 | 0,722668074 | 1,900376506 | 1,144840339 | 0,284631858 | 0,426947787 |
| b1861 | 612 | 0,53 | -0,228564363 | 5,703117711 | 1,142880123 | 0,285044565 | 0,4274238 |
| b4595 | 174 | 0,51 | 0,349673242 | 4,559309441 | 1,141592201 | 0,285316139 | 0,427662509 |
| b3809 | 825 | 0,55 | 0,212995045 | 7,235324776 | 1,141220428 | 0,285394593 | 0,427662509 |
| b4193 | 1398 | 0,53 | -0,364175216 | 3,739383056 | 1,140633036 | 0,285518604 | 0,427705341 |
| b1327 | 921 | 0,48 | -0,343548146 | 4,335003442 | 1,133615854 | 0,287005383 | 0,429788883 |
| b2420 | 735 | 0,39 | -0,348308636 | 3,863108624 | 1,126999745 | 0,288416205 | 0,431701717 |
| b1284 | 750 | 0,49 | 0,299045636 | 5,012250163 | 1,12598457 | 0,288633461 | 0,431701717 |
| b2684 | 531 | 0,49 | 0,330395825 | 6,596399431 | 1,125563289 | 0,28872368 | 0,431701717 |
| b3174 | 87 | 0,57 | 1,238549689 | 0,488249675 | 1,125390289 | 0,288760739 | 0,431701717 |
| b3978 | 75 | 0,57 | 1,239691129 | 0,582092028 | 1,125373503 | 0,288764335 | 0,431701717 |
| b3884 | 786 | 0,52 | -0,268618344 | 5,794865062 | 1,123974189 | 0,289064315 | 0,432006088 |
| b4659 | 796 | 0,43 | 0,2911179 | 4,883445636 | 1,122080951 | 0,289470812 | 0,432469393 |
| b0010 | 567 | 0,53 | -0,245853702 | 5,335041043 | 1,117613784 | 0,290432849 | 0,433762089 |
| b1469 | 1389 | 0,49 | 0,394740967 | 6,987640176 | 1,116728813 | 0,290623917 | 0,433902863 |
| b0704 | 570 | 0,37 | 0,589561553 | 2,093878987 | 1,115420998 | 0,290906572 | 0,434180239 |
| b2440 | 888 | 0,57 | -0,306041736 | 5,71897362 | 1,114751207 | 0,291051469 | 0,434251891 |

| | | | | | | | |
|---|---|---|---|---|---|---|---|
| b4237 | 465 | 0,51 | -0,458410072 | 3,956947653 | 1,111720661 | 0,291708221 | 0,435086937 |
| b0974 | 708 | 0,52 | -0,430902615 | 4,527510371 | 1,110255499 | 0,292026415 | 0,435304874 |
| b1679 | 417 | 0,49 | -0,351559307 | 5,013515073 | 1,110153496 | 0,292048584 | 0,435304874 |
| b2187 | 228 | 0,5 | 0,309304758 | 4,485530398 | 1,107485427 | 0,292629215 | 0,43598749 |
| b1058 | 141 | 0,38 | 1,242435195 | 0,651800074 | 1,107155549 | 0,292701106 | 0,43598749 |
| b1896 | 1425 | 0,49 | -0,285343019 | 9,207017544 | 1,106551758 | 0,29283275 | 0,436038666 |
| b4657 | 246 | 0,42 | 0,790117056 | 1,022910579 | 1,105554755 | 0,293050291 | 0,436217671 |
| b4350 | 3513 | 0,54 | -0,238734643 | 9,286395957 | 1,100153061 | 0,294232516 | 0,437832051 |
| b4622 | 276 | 0,47 | 1,234180931 | 0,55160108 | 1,096490676 | 0,295037546 | 0,438884262 |
| b1513 | 1536 | 0,53 | 0,370162373 | 7,241550378 | 1,095806014 | 0,295188355 | 0,438962908 |
| b3956 | 2652 | 0,56 | 0,245840263 | 9,052023789 | 1,09272452 | 0,295868331 | 0,439828145 |
| b0828 | 966 | 0,57 | -0,226117184 | 6,908940736 | 1,091788185 | 0,296075344 | 0,439950653 |
| b2383 | 2496 | 0,55 | 0,30143868 | 3,730527652 | 1,091464009 | 0,296147059 | 0,439950653 |
| b3357 | 633 | 0,49 | 0,225083301 | 9,524156209 | 1,090297939 | 0,296405205 | 0,440188246 |
| b0045 | 1332 | 0,53 | 0,617657418 | 1,963171097 | 1,088955478 | 0,296702757 | 0,440484186 |
| b2340 | 486 | 0,53 | 0,252593595 | 6,899404711 | 1,087907232 | 0,296935364 | 0,440631969 |
| b2947 | 951 | 0,53 | 0,198278927 | 7,232484561 | 1,087620982 | 0,296998924 | 0,440631969 |
| b1912 | 549 | 0,52 | -0,214297551 | 6,057432991 | 1,086308221 | 0,297290638 | 0,440918808 |
| b3647 | 1683 | 0,53 | -0,274853796 | 4,029936559 | 1,085208705 | 0,297535248 | 0,441135621 |
| b0224 | 741 | 0,48 | 0,287101467 | 6,173906281 | 1,083283904 | 0,297964084 | 0,441625339 |
| b3097 | 369 | 0,49 | 0,232335828 | 9,019576932 | 1,079551706 | 0,298797864 | 0,44260742 |
| b0713 | 735 | 0,58 | -0,284192671 | 6,730485113 | 1,07943405 | 0,298824197 | 0,44260742 |
| b1445 | 174 | 0,45 | 0,572131912 | 1,787464203 | 1,077043346 | 0,299359922 | 0,443254434 |
| b1421 | 1641 | 0,51 | 0,31328231 | 3,930405882 | 1,075270791 | 0,299757927 | 0,443697169 |
| b4230 | 1026 | 0,58 | -0,437539493 | 5,469311696 | 1,073711771 | 0,300108547 | 0,444069498 |
| b1153 | 585 | 0,57 | 0,945424482 | 1,098316828 | 1,072876876 | 0,300296531 | 0,444070236 |
| b1616 | 1374 | 0,52 | 0,650589033 | 2,575118932 | 1,072829494 | 0,300307204 | 0,444070236 |
| b4260 | 1512 | 0,55 | 0,220586309 | 9,421889618 | 1,071478205 | 0,300611793 | 0,444374029 |
| b1562 | 156 | 0,52 | 0,344899139 | 4,761754646 | 1,069593506 | 0,301037281 | 0,444856279 |
| b4083 | 1986 | 0,53 | 0,504406129 | 2,274908447 | 1,069037149 | 0,301163032 | 0,444895422 |
| b3963 | 648 | 0,53 | -0,181466049 | 7,03820165 | 1,067243564 | 0,30156889 | 0,445348193 |
| b0579 | 369 | 0,47 | -0,307889135 | 4,110538328 | 1,064322466 | 0,302231397 | 0,446179553 |
| b2179 | 1026 | 0,53 | 0,362192319 | 4,325456534 | 1,062864271 | 0,302562819 | 0,44652175 |
| b1528 | 1191 | 0,51 | 0,391916776 | 3,510774653 | 1,058323048 | 0,303597971 | 0,447901944 |
| b3215 | 675 | 0,42 | 0,808231037 | 1,16215952 | 1,052981214 | 0,304821484 | 0,44955903 |
| b0284 | 2199 | 0,59 | 0,286760296 | 5,713223908 | 1,051440286 | 0,305175609 | 0,44993325 |
| b1122 | 462 | 0,44 | 0,906171339 | 1,184989624 | 1,050668844 | 0,305353096 | 0,450046885 |
| b3872 | 711 | 0,49 | 0,393505059 | 3,361701367 | 1,047686824 | 0,306040434 | 0,450911646 |
| b0994 | 1029 | 0,53 | -0,288529468 | 4,344167208 | 1,044895737 | 0,30668558 | 0,451713694 |
| b3862 | 933 | 0,45 | 0,408023353 | 2,841390025 | 1,043312711 | 0,307052273 | 0,452095797 |
| b1838 | 657 | 0,48 | 0,373488048 | 4,283129082 | 1,042905275 | 0,307146743 | 0,452095797 |
| b4437 | 136 | 0,54 | 0,549560702 | 2,277390157 | 1,041840571 | 0,30739379 | 0,452240888 |
| b1895 | 429 | 0,45 | -0,269357381 | 5,355832677 | 1,041610885 | 0,307447118 | 0,452240888 |
| b4632 | 93 | 0,66 | 1,0357416 | 0,509980899 | 1,036928124 | 0,308536987 | 0,45366351 |
| b3688 | 333 | 0,54 | -0,24637333 | 6,70710693 | 1,036586476 | 0,308616698 | 0,45366351 |
| b3711 | 960 | 0,52 | -0,298444541 | 4,457822792 | 1,034383678 | 0,309131287 | 0,45427096 |
| b1952 | 189 | 0,51 | -0,272319683 | 5,002561798 | 1,032327459 | 0,309612641 | 0,454829189 |
| b0993 | 2745 | 0,54 | 0,252461388 | 4,56297516 | 1,029736911 | 0,310220468 | 0,455479327 |
| b0802 | 261 | 0,51 | 0,327458182 | 3,770880375 | 1,029277008 | 0,310328538 | 0,455479327 |
| b3354 | 219 | 0,5 | 0,279533629 | 4,445168085 | 1,028990978 | 0,310395776 | 0,455479327 |
| b4524 | 1082 | 0,49 | 0,622059484 | 1,468905929 | 1,02871061 | 0,310461701 | 0,455479327 |
| b3463 | 669 | 0,56 | 0,18270534 | 6,789456089 | 1,022986749 | 0,31181159 | 0,457310061 |
| b4321 | 1344 | 0,53 | -0,294520885 | 4,793607211 | 1,018500078 | 0,312875059 | 0,458719665 |
| b4166 | 1140 | 0,53 | -0,209919681 | 7,092783604 | 1,016553996 | 0,313337808 | 0,459247892 |
| b1362 | 444 | 0,52 | 0,606676669 | 1,812035546 | 1,014354554 | 0,313861878 | 0,459865622 |
| b0487 | 408 | 0,59 | -0,296500953 | 7,003337369 | 1,005983993 | 0,315866876 | 0,462652071 |
| b2735 | 768 | 0,52 | -0,24714196 | 5,999149602 | 1,003835155 | 0,316384289 | 0,463181031 |
| b3080 | 2352 | 0,54 | -0,407910528 | 4,271721386 | 1,003626049 | 0,316434698 | 0,463181031 |
| b1936 | 162 | 0,5 | 1,175891421 | 0,593512072 | 1,001520913 | 0,316942771 | 0,463773262 |
| b1337 | 1446 | 0,53 | 0,245273889 | 4,009816669 | 0,999299115 | 0,317480161 | 0,46435633 |
| b4480 | 840 | 0,48 | 0,250867553 | 5,252608028 | 0,999017135 | 0,317548449 | 0,46435633 |
| b4498 | 2035 | 0,46 | 0,313911959 | 6,971746585 | 0,998205301 | 0,317745162 | 0,464492439 |
| b3291 | 411 | 0,45 | -0,22192838 | 8,623681238 | 0,99393271 | 0,318783082 | 0,465857767 |
| b4421 | 108 | 0,58 | 1,165825831 | 0,671649966 | 0,99314891 | 0,318973969 | 0,465984788 |
| b0076 | 945 | 0,46 | 0,397669073 | 3,234622594 | 0,991286291 | 0,319428197 | 0,466496311 |
| b0041 | 771 | 0,53 | 0,584320568 | 2,152513228 | 0,990824805 | 0,319540869 | 0,466508851 |
| b4408 | 369 | 0,51 | 0,28977693 | 15,07898474 | 0,990246071 | 0,31968224 | 0,466563269 |
| b2354 | 441 | 0,46 | 0,504047104 | 2,085807368 | 0,987946578 | 0,320244767 | 0,467084772 |
| b2958 | 720 | 0,52 | 0,237107445 | 6,15804923 | 0,987933407 | 0,320247993 | 0,467084772 |
| b2180 | 1590 | 0,51 | 0,217160119 | 6,063072034 | 0,987070759 | 0,320459362 | 0,467164035 |
| b0714 | 792 | 0,52 | -0,335580518 | 4,635398289 | 0,986860939 | 0,320510801 | 0,467164035 |
| b1937 | 315 | 0,54 | 0,826583394 | 1,169860285 | 0,98414452 | 0,321177729 | 0,467983933 |
| b0004 | 1287 | 0,53 | 0,257012975 | 8,665248679 | 0,983213551 | 0,321406718 | 0,468109262 |
| b3593 | 4134 | 0,6 | -0,394804182 | 3,660329884 | 0,982563597 | 0,321566714 | 0,468109262 |
| b0559 | 207 | 0,4 | -0,570367945 | 2,452819496 | 0,982399608 | 0,321607099 | 0,468109262 |
| b2488 | 546 | 0,55 | 0,423662809 | 2,359551454 | 0,982097522 | 0,32168151 | 0,468109262 |
| b2448 | 378 | 0,43 | 0,517619796 | 2,369320137 | 0,977486742 | 0,322820078 | 0,469613628 |
| b3890 | 219 | 0,46 | 0,457424362 | 2,347148433 | 0,975794287 | 0,323239339 | 0,470070966 |
| b2792 | 330 | 0,52 | 0,47037978 | 3,050184064 | 0,972596129 | 0,324033564 | 0,471073122 |
| b3742 | 444 | 0,5 | 0,293280944 | 6,080218056 | 0,970809601 | 0,32447835 | 0,471566785 |
| b1596 | 1254 | 0,53 | 0,324556668 | 4,903455769 | 0,970310441 | 0,324602768 | 0,471594686 |
| b3511 | 573 | 0,47 | -0,405131666 | 9,851899267 | 0,967761892 | 0,325238992 | 0,472329551 |
| b0550 | 363 | 0,53 | 1,001570407 | 0,727597516 | 0,967440468 | 0,325319349 | 0,472329551 |
| b1854 | 1443 | 0,53 | -0,279423478 | 9,928270337 | 0,966374006 | 0,325586159 | 0,472549342 |
| b2056 | 1218 | 0,42 | 0,77569317 | 1,518282893 | 0,965992814 | 0,325681597 | 0,472549342 |
| b1438 | 417 | 0,46 | 0,282741326 | 4,091924178 | 0,964871957 | 0,325962437 | 0,472785008 |
| b1847 | 357 | 0,49 | -0,257590044 | 8,567415429 | 0,964502852 | 0,326054989 | 0,472785008 |
| b4055 | 714 | 0,49 | -0,304692174 | 6,455989061 | 0,963677916 | 0,326261966 | 0,472932125 |
| b4044 | 1380 | 0,56 | -0,269258238 | 4,818321605 | 0,961514209 | 0,326805668 | 0,473567089 |
| b2094 | 453 | 0,43 | 0,343320067 | 10,98514974 | 0,960994713 | 0,326936388 | 0,47360339 |
| b3413 | 684 | 0,56 | 0,229053936 | 5,445536439 | 0,955137553 | 0,328415016 | 0,475591632 |
| b1640 | 1110 | 0,55 | -0,207230385 | 6,015165595 | 0,952436039 | 0,329099999 | 0,476429649 |
| b0773 | 477 | 0,52 | 0,245008436 | 7,510992709 | 0,951485564 | 0,329341448 | 0,47662524 |
| b0553 | 2297 | 0,44 | 0,380343789 | 10,76771852 | 0,950820491 | 0,329510536 | 0,476716018 |

| | | | | | | | |
|---|---|---|---|---|---|---|---|
| b2696 | 186 | 0,49 | -0,227488447 | 8,622825811 | 0,948847237 | 0,330012897 | 0,47728874 |
| b0086 | 1359 | 0,55 | -0,215360136 | 8,017211865 | 0,947173758 | 0,330439738 | 0,477751905 |
| b1115 | 1074 | 0,5 | 0,614103897 | 2,173205012 | 0,946382243 | 0,330641879 | 0,477890004 |
| b2291 | 600 | 0,49 | 0,26349797 | 4,306909543 | 0,941768147 | 0,331823534 | 0,479443288 |
| b0428 | 891 | 0,54 | 0,366149292 | 10,43142282 | 0,940957937 | 0,332031606 | 0,47958932 |
| b0505 | 483 | 0,51 | -0,431879594 | 3,387201845 | 0,935652736 | 0,33339836 | 0,481320846 |
| b0353 | 1212 | 0,57 | 0,376272037 | 3,716777292 | 0,935471573 | 0,333445165 | 0,481320846 |
| b1146 | 201 | 0,43 | 0,531523239 | 2,505095927 | 0,933836796 | 0,333867917 | 0,481630097 |
| b4078 | 690 | 0,5 | 0,201695606 | 5,943942111 | 0,933812048 | 0,333874322 | 0,481630097 |
| b4161 | 1053 | 0,54 | 0,19213009 | 7,019585103 | 0,931355514 | 0,334510943 | 0,482393194 |
| b1540 | 687 | 0,5 | 0,2772425 | 5,09084026 | 0,929116991 | 0,33509248 | 0,483033499 |
| b0340 | 471 | 0,51 | -0,424123717 | 2,5309864 | 0,928817059 | 0,3351705 | 0,483033499 |
| b3500 | 1353 | 0,54 | -0,1887442 | 8,068301601 | 0,92814993 | 0,335344126 | 0,483128375 |
| b0659 | 468 | 0,52 | -0,219418992 | 7,312665653 | 0,926681422 | 0,335726743 | 0,483524184 |
| b3893 | 903 | 0,55 | -0,329930813 | 10,01346792 | 0,925202165 | 0,336112751 | 0,483924622 |
| b4026 | 243 | 0,6 | 0,47379384 | 2,33599809 | 0,924674355 | 0,336250625 | 0,483967663 |
| b1210 | 1257 | 0,54 | 0,213769242 | 6,7816948 | 0,919439245 | 0,337622251 | 0,485785852 |
| b2648 | 240 | 0,5 | 1,127763357 | 0,603859661 | 0,918605787 | 0,337841314 | 0,485945047 |
| b4539 | 255 | 0,44 | 0,26642486 | 4,486389417 | 0,917274327 | 0,338191664 | 0,486292922 |
| b3666 | 1392 | 0,53 | 0,487205855 | 2,8754814 | 0,914497892 | 0,338923806 | 0,487189383 |
| b1763 | 1962 | 0,55 | -0,167197992 | 7,525716042 | 0,910637116 | 0,33994543 | 0,488501256 |
| b0237 | 1458 | 0,51 | -0,241257819 | 9,546847737 | 0,909307414 | 0,340298249 | 0,488851523 |
| b2751 | 1428 | 0,55 | -0,245929129 | 5,069165494 | 0,908507794 | 0,340510655 | 0,488999922 |
| b3213 | 1419 | 0,56 | -0,50038644 | 7,178178867 | 0,907936162 | 0,340662609 | 0,48906144 |
| b0293 | 588 | 0,53 | -0,349911178 | 3,404649803 | 0,905925708 | 0,341197764 | 0,489665559 |
| b1304 | 669 | 0,49 | 0,229150337 | 6,569096631 | 0,905534913 | 0,34130192 | 0,489665559 |
| b4403 | 687 | 0,52 | 0,269674407 | 4,661629705 | 0,902227092 | 0,342185247 | 0,490775769 |
| b2924 | 861 | 0,5 | 0,410038106 | 8,241575744 | 0,900536606 | 0,342637868 | 0,491114066 |
| b4210 | 966 | 0,56 | 0,341436981 | 3,805391016 | 0,900527647 | 0,342640269 | 0,491114066 |
| b0573 | 333 | 0,45 | -0,831976527 | 1,612163784 | 0,898159474 | 0,343275704 | 0,491787896 |
| b2834 | 1041 | 0,54 | 0,183034962 | 7,973515164 | 0,897958004 | 0,343329836 | 0,491787896 |
| b3847 | 1332 | 0,54 | -0,215524457 | 8,806023404 | 0,896718022 | 0,343663257 | 0,492108217 |
| b2249 | 1203 | 0,54 | 0,180651598 | 6,471592865 | 0,895755498 | 0,343922373 | 0,492321967 |
| b4045 | 210 | 0,42 | 0,298378529 | 10,04243795 | 0,888382482 | 0,345916017 | 0,495017748 |
| b1302 | 1266 | 0,58 | -0,402160844 | 6,710696008 | 0,886946479 | 0,346306127 | 0,495265205 |
| b2483 | 948 | 0,54 | 0,558990225 | 1,619991572 | 0,886697364 | 0,346373863 | 0,495265205 |
| b1655 | 816 | 0,48 | 0,323489847 | 5,383417798 | 0,886225063 | 0,346502334 | 0,495265205 |
| b2021 | 1071 | 0,55 | -0,307677789 | 5,84524893 | 0,886119925 | 0,346530942 | 0,495265205 |
| b0417 | 978 | 0,54 | -0,162817051 | 6,174089895 | 0,883185631 | 0,347330643 | 0,496249902 |
| b1448 | 519 | 0,53 | 0,222271536 | 5,092124635 | 0,880886308 | 0,347959042 | 0,496914779 |
| b3559 | 2070 | 0,55 | 0,231100869 | 9,880832105 | 0,880671834 | 0,348017736 | 0,496914779 |
| b0461 | 375 | 0,41 | -0,394293492 | 6,77309165 | 0,879471594 | 0,348346446 | 0,497225724 |
| b3621 | 960 | 0,51 | 0,202133041 | 5,951019707 | 0,87224227 | 0,350335301 | 0,499905386 |
| b4446 | 140 | 0,47 | 0,408773464 | 2,692019674 | 0,869029513 | 0,351224121 | 0,500855644 |
| b1073 | 417 | 0,53 | -0,732404037 | 1,201780274 | 0,869027274 | 0,351224741 | 0,500855644 |
| b1991 | 1080 | 0,51 | 0,19826876 | 6,110890745 | 0,867905533 | 0,351535799 | 0,501139774 |
| b1317 | 660 | 0,57 | 0,553023063 | 1,891627327 | 0,864379203 | 0,352516097 | 0,502377472 |
| b2748 | 312 | 0,52 | 0,208442898 | 5,759116359 | 0,863767607 | 0,352686497 | 0,502460546 |
| b1042 | 456 | 0,51 | -0,322565628 | 3,472675445 | 0,859399953 | 0,353906665 | 0,504038663 |
| b1399 | 951 | 0,5 | -0,180288186 | 6,932844442 | 0,856374027 | 0,354755388 | 0,504980122 |
| b3720 | 1617 | 0,45 | 0,605922047 | 2,076944608 | 0,856240017 | 0,35479304 | 0,504980122 |
| b3406 | 477 | 0,5 | -0,214401633 | 4,895351573 | 0,853609857 | 0,355533129 | 0,505872852 |
| b4631 | 129 | 0,36 | 0,940868876 | 0,499178999 | 0,852968913 | 0,355713801 | 0,505969298 |
| b0055 | 816 | 0,53 | 0,201079895 | 5,211522915 | 0,848837896 | 0,356881299 | 0,507468903 |
| b4215 | 939 | 0,36 | -0,513726884 | 2,562306052 | 0,847019449 | 0,357396891 | 0,50804087 |
| b3687 | 414 | 0,47 | 0,650701855 | 7,045929275 | 0,846614802 | 0,357511761 | 0,508043029 |
| b0549 | 291 | 0,53 | 1,078576987 | 0,572881136 | 0,844191874 | 0,358200638 | 0,508860622 |
| b3123 | 377 | 0,62 | -0,26217045 | 12,88633405 | 0,843322744 | 0,35844819 | 0,509050947 |
| b3696 | 657 | 0,43 | 0,372127685 | 3,545278124 | 0,842903231 | 0,358567763 | 0,50905946 |
| b3781 | 330 | 0,51 | -0,204265328 | 8,882105621 | 0,8416481 | 0,358925839 | 0,509291075 |
| b4102 | 726 | 0,62 | 0,625471432 | 1,12542998 | 0,841534865 | 0,358958167 | 0,509291075 |
| b0886 | 1722 | 0,54 | -0,203245128 | 7,063109116 | 0,840376768 | 0,359289037 | 0,509599198 |
| b0700 | 4194 | 0,59 | -0,268274883 | 4,504846207 | 0,838402423 | 0,359854079 | 0,510239159 |
| b2921 | 897 | 0,45 | 0,318348046 | 3,229930279 | 0,837940116 | 0,359986564 | 0,510265585 |
| b3771 | 1851 | 0,56 | 0,199898602 | 6,381880779 | 0,83692148 | 0,360278716 | 0,510518244 |
| b2930 | 966 | 0,57 | 0,310387194 | 3,166735767 | 0,836407545 | 0,36042624 | 0,510565869 |
| b2867 | 879 | 0,54 | 0,296129328 | 3,742130237 | 0,835824513 | 0,360593699 | 0,510605927 |
| b0411 | 885 | 0,51 | -0,234228436 | 8,428883616 | 0,835515957 | 0,360682366 | 0,510605927 |
| b0352 | 1014 | 0,57 | 0,341538692 | 3,553857655 | 0,833421683 | 0,361284976 | 0,511297525 |
| b0878 | 1116 | 0,53 | 0,208556685 | 5,8613038 | 0,831584361 | 0,361814794 | 0,511885703 |
| b2545 | 1062 | 0,55 | 0,27252703 | 4,236586148 | 0,830790055 | 0,362044176 | 0,512011876 |
| b4696 | 5323 | 0,51 | -0,168895067 | 6,865482377 | 0,830484558 | 0,362132452 | 0,512011876 |
| b2360 | 825 | 0,46 | -0,640069131 | 2,318527572 | 0,826043701 | 0,363419035 | 0,51366891 |
| b1063 | 561 | 0,47 | -0,189387174 | 5,313771527 | 0,824987753 | 0,363725888 | 0,513929302 |
| b1794 | 1026 | 0,48 | 0,246624788 | 5,448786347 | 0,824341512 | 0,36391386 | 0,513929302 |
| b0795 | 999 | 0,57 | -0,184333976 | 7,091627324 | 0,824226743 | 0,363947257 | 0,513929302 |
| b2497 | 1290 | 0,52 | 0,423750133 | 4,329373537 | 0,823065069 | 0,364285533 | 0,514244964 |
| b1573 | 219 | 0,48 | 0,585038421 | 1,758046693 | 0,821891341 | 0,364627762 | 0,514566004 |
| b3806 | 2547 | 0,52 | 0,18690089 | 8,64797763 | 0,82123472 | 0,36481941 | 0,514674409 |
| b1741 | 888 | 0,52 | 0,262154851 | 4,108947047 | 0,819698332 | 0,365268381 | 0,514992332 |
| b1213 | 393 | 0,48 | 0,266162133 | 3,852219756 | 0,819677172 | 0,36527457 | 0,514992332 |
| b4222 | 342 | 0,51 | 0,193190598 | 6,27855383 | 0,816960007 | 0,36607049 | 0,515952181 |
| b4568 | 162 | 0,52 | -0,302103954 | 8,967608928 | 0,812941848 | 0,367251919 | 0,517454606 |
| b4341 | 165 | 0,56 | 0,913274633 | 0,583291903 | 0,809305405 | 0,368325688 | 0,518804441 |
| b0225 | 279 | 0,39 | -0,289799018 | 4,099528206 | 0,808588258 | 0,368537962 | 0,518940354 |
| b3774 | 1476 | 0,54 | -0,270977774 | 5,534015617 | 0,807592615 | 0,368832953 | 0,519192618 |
| b1501 | 2280 | 0,5 | 0,307270436 | 4,399163665 | 0,803704798 | 0,369987999 | 0,520655012 |
| b2790 | 450 | 0,53 | -0,204823469 | 6,404968698 | 0,802499881 | 0,370346996 | 0,520880767 |
| b3891 | 930 | 0,55 | -0,23915132 | 8,023259013 | 0,802386312 | 0,370380858 | 0,520880767 |
| b3344 | 360 | 0,5 | 0,279813376 | 4,153850327 | 0,800322303 | 0,370997024 | 0,521448242 |
| b3722 | 1878 | 0,51 | 0,304923669 | 4,358148099 | 0,800255281 | 0,371017056 | 0,521448242 |
| b1048 | 1536 | 0,5 | -0,202512687 | 8,363445884 | 0,796885512 | 0,372026184 | 0,522639488 |
| b4447 | 143 | 0,48 | 0,348228777 | 3,974048321 | 0,796646655 | 0,37209786 | 0,522639488 |
| b1036 | 492 | 0,54 | 0,255018321 | 4,950299311 | 0,796241328 | 0,372219532 | 0,522646598 |

| | | | | | | | |
|---|---|---|---|---|---|---|---|
| b1097 | 1023 | 0,51 | 0,252959975 | 5,06179291 | 0,795105417 | 0,37256081 | 0,522961964 |
| b0268 | 909 | 0,62 | 0,199871689 | 5,253888297 | 0,793298098 | 0,373104712 | 0,523561466 |
| b3583 | 696 | 0,57 | -0,353382553 | 3,028572464 | 0,786899161 | 0,375039401 | 0,525879233 |
| b1191 | 1737 | 0,52 | -0,211031185 | 5,038001255 | 0,786735618 | 0,375089032 | 0,525879233 |
| b1802 | 1125 | 0,52 | -0,37908184 | 2,921922668 | 0,786671761 | 0,375108413 | 0,525879233 |
| b2419 | 375 | 0,46 | 0,594944608 | 2,65481592 | 0,785792935 | 0,375375289 | 0,526088819 |
| b2221 | 663 | 0,54 | 0,714452974 | 1,284865975 | 0,785264258 | 0,375535962 | 0,526149479 |
| b1101 | 1434 | 0,53 | 0,226226814 | 9,784524133 | 0,781673921 | 0,376629684 | 0,527516951 |
| b4536 | 240 | 0,44 | -0,409673905 | 3,887553137 | 0,779981038 | 0,377146939 | 0,528076408 |
| b3894 | 3051 | 0,55 | -0,304592071 | 11,91365862 | 0,779432044 | 0,377314897 | 0,528146586 |
| b1800 | 1086 | 0,54 | -0,278167003 | 5,771732764 | 0,778525798 | 0,377592383 | 0,528369984 |
| b3988 | 4224 | 0,54 | 0,278270183 | 12,55801865 | 0,777220314 | 0,377992617 | 0,528764953 |
| b3907 | 1035 | 0,52 | 0,514887327 | 2,89143728 | 0,776477719 | 0,378220548 | 0,52891872 |
| b4173 | 1281 | 0,54 | 0,192996796 | 9,011789843 | 0,774863549 | 0,378716667 | 0,529447318 |
| b3839 | 777 | 0,52 | -0,305494848 | 5,937932207 | 0,774179398 | 0,37892722 | 0,529506353 |
| b1357 | 297 | 0,43 | 0,508491892 | 2,101600313 | 0,773958699 | 0,378995176 | 0,529506353 |
| b2970 | 849 | 0,51 | 0,543516096 | 1,582846819 | 0,772307194 | 0,379504248 | 0,530052366 |
| b2915 | 321 | 0,53 | -0,549719937 | 1,725079625 | 0,769817403 | 0,380273546 | 0,530560104 |
| b3143 | 696 | 0,44 | 0,823894541 | 1,049451216 | 0,769812349 | 0,38027511 | 0,530560104 |
| b1951 | 624 | 0,38 | -0,376693121 | 2,713716187 | 0,769616704 | 0,380335654 | 0,530560104 |
| b0292 | 669 | 0,55 | 0,641340234 | 1,500286766 | 0,769598528 | 0,380341279 | 0,530560104 |
| b3580 | 1497 | 0,57 | 0,457247437 | 2,533593404 | 0,768948899 | 0,380542415 | 0,530675515 |
| b4671 | 102 | 0,28 | -0,703684097 | 1,009207618 | 0,767510005 | 0,380988456 | 0,531132274 |
| b1450 | 666 | 0,44 | 0,363446857 | 4,86022252 | 0,766433367 | 0,381322685 | 0,531432922 |
| b2376 | 276 | 0,4 | 0,477498009 | 2,626577952 | 0,763939555 | 0,382098454 | 0,532348545 |
| b0957 | 1041 | 0,54 | 0,248992774 | 14,05413377 | 0,761968632 | 0,382713146 | 0,532880997 |
| b0287 | 615 | 0,45 | -0,331891283 | 5,47230614 | 0,761951761 | 0,382718413 | 0,532880997 |
| b1112 | 258 | 0,47 | 0,587566994 | 2,383715657 | 0,761158959 | 0,382966073 | 0,532972558 |
| b2725 | 462 | 0,5 | 0,762846576 | 0,91640815 | 0,760980022 | 0,383022001 | 0,532972558 |
| b4410 | 126 | 0,54 | 0,579546207 | 1,699063902 | 0,760329351 | 0,383225473 | 0,533090183 |
| b3439 | 696 | 0,52 | -0,192593832 | 6,177283868 | 0,75765896 | 0,38406214 | 0,534088275 |
| b1648 | 240 | 0,52 | -0,401768026 | 4,193949656 | 0,752976772 | 0,385535401 | 0,53597074 |
| b2498 | 627 | 0,53 | -0,239750723 | 6,756168045 | 0,751453621 | 0,386016396 | 0,536464635 |
| b1506 | 203 | 0,46 | 0,758499969 | 0,754962849 | 0,751094091 | 0,386130056 | 0,536464635 |
| b2165 | 939 | 0,5 | 0,341747968 | 3,811742118 | 0,747129062 | 0,387386712 | 0,538043769 |
| b4576 | 504 | 0,53 | 0,302874113 | 3,19716441 | 0,743715998 | 0,388473106 | 0,53934499 |
| b0753 | 381 | 0,5 | -0,324635408 | 8,702643764 | 0,743183446 | 0,388643011 | 0,53934499 |
| b2894 | 897 | 0,53 | 0,152900148 | 6,283855482 | 0,743053183 | 0,388684587 | 0,53934499 |
| b3626 | 1017 | 0,34 | -0,181858552 | 5,554438887 | 0,741808512 | 0,389082162 | 0,539729573 |
| b3578 | 1278 | 0,51 | 0,535497828 | 1,510497143 | 0,739201919 | 0,389916652 | 0,540719812 |
| b0596 | 747 | 0,58 | 0,381735505 | 3,669203532 | 0,736923845 | 0,390648066 | 0,541566542 |
| b0499 | 708 | 0,45 | 0,401354408 | 2,17217486 | 0,733000678 | 0,391912278 | 0,543151153 |
| b3343 | 288 | 0,5 | 0,302015673 | 4,143715178 | 0,732281418 | 0,39214469 | 0,543305256 |
| b3679 | 1716 | 0,53 | -0,293790805 | 2,837019089 | 0,731612667 | 0,392360959 | 0,543436903 |
| b0898 | 1149 | 0,52 | 0,223090937 | 4,687656503 | 0,729699472 | 0,392980619 | 0,54412701 |
| b1599 | 330 | 0,52 | -0,318379211 | 3,00151813 | 0,72689132 | 0,393892692 | 0,545221448 |
| b1570 | 408 | 0,44 | -0,203823177 | 5,444693565 | 0,722737544 | 0,395247411 | 0,546773963 |
| b4511 | 219 | 0,56 | 0,995015476 | 0,690381672 | 0,722548157 | 0,395309337 | 0,546773963 |
| b4440 | 304 | 0,53 | -0,267078502 | 4,356017406 | 0,722331251 | 0,39538028 | 0,546773963 |
| b2334 | 489 | 0,47 | 0,546468797 | 1,617765144 | 0,721481587 | 0,395658352 | 0,54697025 |
| b3220 | 1128 | 0,49 | 0,328978935 | 2,816445099 | 0,721152 | 0,395766292 | 0,54697025 |
| b4497 | 1053 | 0,39 | 0,502803826 | 1,715138206 | 0,719870416 | 0,396186418 | 0,547382098 |
| b4355 | 1656 | 0,55 | -0,264727103 | 4,354305604 | 0,718456521 | 0,396650665 | 0,547854632 |
| b3952 | 879 | 0,55 | 0,336411836 | 3,199185929 | 0,7174842 | 0,396970379 | 0,548127307 |
| b3442 | 1179 | 0,34 | 0,861260224 | 0,87587061 | 0,714053833 | 0,398101313 | 0,549519583 |
| b2353 | 291 | 0,54 | 0,33282493 | 3,708918875 | 0,712833548 | 0,398504745 | 0,549907101 |
| b0860 | 732 | 0,45 | -0,277733456 | 5,028130536 | 0,712357114 | 0,398662416 | 0,549955355 |
| b2120 | 2280 | 0,56 | 0,393286619 | 2,423158173 | 0,71003594 | 0,399431881 | 0,550716654 |
| b1176 | 696 | 0,51 | 0,167594227 | 7,334756735 | 0,709750537 | 0,39952664 | 0,550716654 |
| b4330 | 684 | 0,53 | 0,579346678 | 1,333601407 | 0,709581134 | 0,3995829 | 0,550716654 |
| b1834 | 2634 | 0,54 | -0,156561159 | 7,212262739 | 0,708989727 | 0,399779402 | 0,5508181 |
| b0491 | 780 | 0,46 | -0,225063073 | 4,665367782 | 0,707017269 | 0,400435788 | 0,55155292 |
| b1536 | 393 | 0,48 | -0,28082161 | 5,71042251 | 0,706581818 | 0,400580906 | 0,551583294 |
| b4628 | 192 | 0,58 | 0,854017002 | 0,709365343 | 0,704611197 | 0,401238589 | 0,552151113 |
| b1291 | 993 | 0,51 | -0,180839107 | 6,435764547 | 0,70460798 | 0,401239665 | 0,552151113 |
| b2818 | 1332 | 0,53 | 0,24422133 | 4,803937212 | 0,703698815 | 0,401543623 | 0,552307711 |
| b2858 | 707 | 0,31 | 0,85701267 | 0,80718238 | 0,703530542 | 0,401599918 | 0,552307711 |
| b0467 | 528 | 0,59 | 0,22046289 | 4,839398352 | 0,702944995 | 0,401795899 | 0,552407736 |
| b0902 | 741 | 0,5 | -0,171158312 | 7,082276281 | 0,699375967 | 0,402993458 | 0,55372332 |
| b0756 | 1041 | 0,56 | 0,187280373 | 9,011989665 | 0,699356864 | 0,402999882 | 0,55372332 |
| b3901 | 315 | 0,51 | 0,520966656 | 1,477662938 | 0,698551903 | 0,403270704 | 0,553925619 |
| b1698 | 939 | 0,54 | 0,351371392 | 3,20695984 | 0,696689833 | 0,403898199 | 0,554617563 |
| b0213 | 723 | 0,49 | -0,157573164 | 5,548766368 | 0,695037822 | 0,404456098 | 0,555213548 |
| b3472 | 558 | 0,52 | 0,163417163 | 7,467333512 | 0,693317801 | 0,405038161 | 0,555767796 |
| b1795 | 249 | 0,46 | 0,261675741 | 8,357466132 | 0,693109696 | 0,405108668 | 0,555767796 |
| b4314 | 549 | 0,52 | -0,208384224 | 9,340268856 | 0,692746237 | 0,405231851 | 0,555767796 |
| b2010 | 1167 | 0,49 | 0,222691743 | 4,494942569 | 0,69113423 | 0,405778855 | 0,55634776 |
| b2664 | 663 | 0,54 | 0,263957304 | 7,507051955 | 0,690548501 | 0,405977878 | 0,556450412 |
| b2946 | 732 | 0,53 | 0,187900827 | 5,693342294 | 0,689313754 | 0,406397896 | 0,556855815 |
| b4705 | 129 | 0,41 | -0,443509942 | 2,833415756 | 0,68863391 | 0,406629428 | 0,557002779 |
| b1420 | 168 | 0,51 | -0,283163898 | 3,577865334 | 0,687686908 | 0,406952265 | 0,557274687 |
| b1709 | 750 | 0,55 | 0,20052028 | 6,268286812 | 0,685808945 | 0,407593583 | 0,557982418 |
| b4529 | 267 | 0,55 | 0,171824218 | 6,501502719 | 0,68455644 | 0,408022132 | 0,558390565 |
| b4442 | 78 | 0,41 | 0,90948307 | 0,622200346 | 0,684209678 | 0,408140895 | 0,558390565 |
| b2642 | 1704 | 0,31 | 0,250932347 | 4,386174582 | 0,683109486 | 0,408518036 | 0,55869373 |
| b0858 | 489 | 0,52 | 0,321366331 | 3,575591851 | 0,682687016 | 0,408662993 | 0,55869373 |
| b1315 | 1056 | 0,55 | 0,342267257 | 2,810063327 | 0,68247303 | 0,408736444 | 0,55869373 |
| b1074 | 405 | 0,53 | 0,6631512 | 1,173728093 | 0,681421945 | 0,409097512 | 0,559016783 |
| b3214 | 765 | 0,45 | 0,405041423 | 2,647565498 | 0,679222538 | 0,409854569 | 0,559804642 |
| b1275 | 975 | 0,48 | -0,152877561 | 6,700430411 | 0,67902147 | 0,409923881 | 0,559804642 |
| b1734 | 1353 | 0,47 | 0,267030251 | 3,521945115 | 0,678091781 | 0,410244588 | 0,560071959 |
| b1041 | 456 | 0,42 | 0,683654857 | 1,347709083 | 0,676850969 | 0,410673195 | 0,560486377 |
| b0468 | 378 | 0,52 | 0,286839184 | 3,734503498 | 0,675735531 | 0,411059058 | 0,560842222 |
| b3027 | 333 | 0,34 | 0,839808555 | 0,680958034 | 0,675312768 | 0,411205443 | 0,56087121 |

| | | | | | | | |
|---|---|---|---|---|---|---|---|
| b1843 | 657 | 0,52 | -0,169029878 | 5,543270829 | 0,669730614 | 0,413145543 | 0,563039094 |
| b0885 | 705 | 0,54 | -0,211960801 | 5,128994644 | 0,669490105 | 0,413229436 | 0,563039094 |
| b1160 | 324 | 0,34 | 0,964003581 | 0,572203825 | 0,668593561 | 0,413542386 | 0,563039094 |
| b1265 | 45 | 0,44 | 1,068742269 | 0,410376926 | 0,66857585 | 0,413548572 | 0,563039094 |
| b4668 | 57 | 0,44 | 1,068742269 | 0,410376926 | 0,66857585 | 0,413548572 | 0,563039094 |
| b4678 | 63 | 0,44 | 1,068742269 | 0,410376926 | 0,66857585 | 0,413548572 | 0,563039094 |
| b0786 | 705 | 0,49 | -0,23862062 | 8,644012291 | 0,668162174 | 0,413693092 | 0,563064815 |
| b4500 | 4605 | 0,54 | -0,227220354 | 4,646363338 | 0,667674347 | 0,413863612 | 0,563125899 |
| b3717 | 588 | 0,45 | 0,32544525 | 3,328632828 | 0,66712563 | 0,414055542 | 0,563216066 |
| b1386 | 2274 | 0,51 | 0,314256828 | 3,741996276 | 0,666329432 | 0,414334269 | 0,563424209 |
| b2370 | 3594 | 0,41 | -0,231628595 | 6,340794435 | 0,665896239 | 0,414486034 | 0,563459632 |
| b0310 | 669 | 0,37 | -0,550618542 | 1,239347084 | 0,665407434 | 0,414657381 | 0,563521645 |
| b4261 | 1101 | 0,55 | -0,163311062 | 7,646036686 | 0,663290202 | 0,415400776 | 0,564360799 |
| b0084 | 1767 | 0,54 | -0,163139691 | 7,679260801 | 0,661375647 | 0,416074708 | 0,565105103 |
| b4224 | 252 | 0,5 | -0,268745668 | 4,773156277 | 0,651773139 | 0,419479449 | 0,569556767 |
| b0586 | 3882 | 0,57 | 0,370959588 | 4,730377911 | 0,650439213 | 0,419955693 | 0,570030714 |
| b4112 | 1092 | 0,54 | -0,202764717 | 5,704542045 | 0,64565488 | 0,42167047 | 0,572184998 |
| b4533 | 234 | 0,44 | 0,776551703 | 0,926357129 | 0,641886873 | 0,423028358 | 0,573853844 |
| b1740 | 828 | 0,5 | -0,15534845 | 7,405107466 | 0,638215054 | 0,424357892 | 0,575335805 |
| b3628 | 1080 | 0,4 | 0,165604031 | 6,025759335 | 0,638160898 | 0,424377548 | 0,575335805 |
| b0471 | 330 | 0,53 | 0,153685056 | 7,276236647 | 0,635943983 | 0,425183367 | 0,576253962 |
| b2793 | 546 | 0,48 | -0,181999264 | 5,337984531 | 0,633820334 | 0,425957443 | 0,577128555 |
| b3638 | 669 | 0,48 | -0,196833758 | 4,211618603 | 0,630003218 | 0,427354133 | 0,578672002 |
| b1719 | 1929 | 0,5 | -0,199677474 | 10,94909299 | 0,630001332 | 0,427354825 | 0,578672002 |
| b0354 | 540 | 0,48 | 0,183180139 | 5,581824458 | 0,629111985 | 0,427681231 | 0,578939075 |
| b4499 | 3796 | 0,5 | 0,298671071 | 3,819840067 | 0,628444753 | 0,427926363 | 0,579096003 |
| b4438 | 87 | 0,43 | 1,033208902 | 0,433445674 | 0,626040163 | 0,428811539 | 0,57959429 |
| b4675 | 75 | 0,43 | 1,033108329 | 0,421858274 | 0,626039761 | 0,428811688 | 0,57959429 |
| b2018 | 51 | 0,43 | 1,033108339 | 0,410376926 | 0,626039348 | 0,42881184 | 0,57959429 |
| b4601 | 84 | 0,43 | 1,033108339 | 0,410376926 | 0,626039348 | 0,42881184 | 0,57959429 |
| b1054 | 921 | 0,53 | -0,201711 | 6,024619767 | 0,624473539 | 0,429389732 | 0,580200416 |
| b1611 | 1404 | 0,54 | 0,236802254 | 7,590684476 | 0,623438865 | 0,429772246 | 0,580542256 |
| b2043 | 1395 | 0,47 | -0,257867551 | 3,731016392 | 0,62204866 | 0,430287008 | 0,58106248 |
| b1905 | 498 | 0,43 | -0,306718482 | 8,178151332 | 0,620786414 | 0,4307552 | 0,58151952 |
| b3879 | 927 | 0,48 | -0,375760671 | 2,669502683 | 0,619397269 | 0,431271353 | 0,582041013 |
| b2032 | 1119 | 0,32 | -0,248970607 | 6,730152972 | 0,619047274 | 0,431401545 | 0,582041459 |
| b1600 | 366 | 0,42 | 0,594670612 | 1,761039669 | 0,618616994 | 0,431561684 | 0,582082296 |
| b4482 | 765 | 0,48 | 0,345203549 | 2,396249397 | 0,616344123 | 0,432409086 | 0,582914974 |
| b2029 | 1407 | 0,5 | 0,255398211 | 10,44049934 | 0,616263599 | 0,432439154 | 0,582914974 |
| b2126 | 1686 | 0,51 | 0,178238286 | 5,831664698 | 0,615889141 | 0,432579022 | 0,582928195 |
| b3482 | 4236 | 0,59 | -0,338003756 | 3,021779011 | 0,612843386 | 0,433719229 | 0,584289025 |
| b4703 | 90 | 0,42 | -0,618177283 | 1,507428318 | 0,608547371 | 0,435335275 | 0,585958254 |
| b0220 | 474 | 0,5 | -0,18455376 | 8,799354768 | 0,608545386 | 0,435336024 | 0,585958254 |
| b0290 | 1644 | 0,52 | 0,217236539 | 4,692713126 | 0,608506987 | 0,43535051 | 0,585958254 |
| b0214 | 468 | 0,5 | 0,21567976 | 4,413681506 | 0,607477311 | 0,435739231 | 0,586305384 |
| b0887 | 1767 | 0,54 | -0,142821549 | 6,624547716 | 0,606182101 | 0,436228949 | 0,58678816 |
| b3977 | 85 | 0,59 | 0,504272934 | 1,239571988 | 0,6046072 | 0,43682555 | 0,587414376 |
| b1465 | 681 | 0,53 | -0,25861123 | 6,507238405 | 0,600635308 | 0,43833573 | 0,589071292 |
| b0053 | 1287 | 0,54 | 0,170003419 | 9,124900288 | 0,600544293 | 0,438370429 | 0,589071292 |
| b1923 | 1497 | 0,47 | -0,175846913 | 6,25305358 | 0,600330397 | 0,438451992 | 0,589071292 |
| b1956 | 1695 | 0,51 | -0,132845516 | 6,958550438 | 0,595947161 | 0,440128559 | 0,5911466 |
| b2410 | 999 | 0,52 | -0,195014759 | 6,046374157 | 0,593360678 | 0,4411225 | 0,592303465 |
| b3099 | 405 | 0,53 | -0,195100929 | 10,31095681 | 0,593018665 | 0,441254188 | 0,592303465 |
| b2669 | 405 | 0,48 | -0,362115704 | 4,809743046 | 0,590594689 | 0,442189248 | 0,593380901 |
| b0518 | 1668 | 0,56 | 0,483970019 | 1,779400431 | 0,589254352 | 0,442707601 | 0,593742147 |
| b0801 | 1086 | 0,56 | -0,193737052 | 7,205666441 | 0,589213552 | 0,442723395 | 0,593742147 |
| b2523 | 1284 | 0,57 | -0,179218871 | 8,10558709 | 0,588108871 | 0,44315134 | 0,594048357 |
| b3868 | 1410 | 0,57 | 0,178932747 | 5,551243454 | 0,587940034 | 0,443216802 | 0,594048357 |
| b3547 | 1209 | 0,51 | 0,276998179 | 3,392428632 | 0,586658821 | 0,443714048 | 0,594370948 |
| b3239 | 273 | 0,43 | 0,20515135 | 5,641179533 | 0,586636519 | 0,443722711 | 0,594370948 |
| b3171 | 77 | 0,65 | 0,997222794 | 0,478086535 | 0,583080741 | 0,445107296 | 0,594960077 |
| b4608 | 77 | 0,42 | 0,995676672 | 0,410376926 | 0,582737489 | 0,445241309 | 0,594960077 |
| b0616 | 909 | 0,58 | 0,392935953 | 2,572852678 | 0,582596866 | 0,445296229 | 0,594960077 |
| b3503 | 426 | 0,52 | 0,326324711 | 2,934637637 | 0,581891733 | 0,445571776 | 0,594960077 |
| b0706 | 762 | 0,4 | -0,790919876 | 1,011972915 | 0,581428302 | 0,445753016 | 0,594960077 |
| b4695 | 78 | 0,64 | 0,995440465 | 0,4220575 | 0,580323172 | 0,446185676 | 0,594960077 |
| b0206 | 77 | 0,64 | 0,995069183 | 0,410376926 | 0,580322776 | 0,446185831 | 0,594960077 |
| b0216 | 77 | 0,64 | 0,995069183 | 0,410376926 | 0,580322776 | 0,446185831 | 0,594960077 |
| b1229 | 90 | 0,64 | 0,995069183 | 0,410376926 | 0,580322776 | 0,446185831 | 0,594960077 |
| b2590 | 76 | 0,64 | 0,995069183 | 0,410376926 | 0,580322776 | 0,446185831 | 0,594960077 |
| b3757 | 76 | 0,64 | 0,995069183 | 0,410376926 | 0,580322776 | 0,446185831 | 0,594960077 |
| b3760 | 77 | 0,64 | 0,995069183 | 0,410376926 | 0,580322776 | 0,446185831 | 0,594960077 |
| b3969 | 76 | 0,64 | 0,995069183 | 0,410376926 | 0,580322776 | 0,446185831 | 0,594960077 |
| b4008 | 76 | 0,64 | 0,995069183 | 0,410376926 | 0,580322776 | 0,446185831 | 0,594960077 |
| b4635 | 45 | 0,64 | 0,995069183 | 0,410376926 | 0,580322776 | 0,446185831 | 0,594960077 |
| b3422 | 1599 | 0,56 | 0,210167864 | 3,950384299 | 0,579942677 | 0,446334791 | 0,594960077 |
| b3675 | 363 | 0,5 | 0,298860123 | 3,135529757 | 0,579727443 | 0,446419174 | 0,594960077 |
| b1970 | 414 | 0,42 | -0,349958773 | 3,009917656 | 0,577234837 | 0,447398222 | 0,596087643 |
| b4426 | 96 | 0,5 | 0,640817385 | 0,611506391 | 0,576493485 | 0,447690055 | 0,596240285 |
| b2887 | 1920 | 0,53 | 0,24741142 | 3,631691114 | 0,576268067 | 0,447778849 | 0,596240285 |
| b4479 | 690 | 0,55 | -0,16455676 | 5,039502079 | 0,575234626 | 0,448186281 | 0,596533897 |
| b4476 | 1341 | 0,55 | 0,215193002 | 3,93958419 | 0,575033748 | 0,448265544 | 0,596533897 |
| b2449 | 393 | 0,44 | 0,239825958 | 5,118404716 | 0,570379598 | 0,450108113 | 0,598808122 |
| b0672 | 85 | 0,6 | 0,824772747 | 0,680029614 | 0,568650697 | 0,450795592 | 0,599544761 |
| b0414 | 1104 | 0,55 | -0,157388635 | 7,544420094 | 0,565105926 | 0,452210272 | 0,601247831 |
| b3235 | 1068 | 0,53 | 0,17058323 | 5,481273416 | 0,563477719 | 0,452862401 | 0,601936323 |
| b2451 | 1404 | 0,59 | 0,269049048 | 2,899103447 | 0,561593011 | 0,453619106 | 0,602763365 |
| b4291 | 2325 | 0,59 | 0,361695247 | 5,455548097 | 0,559215735 | 0,454576408 | 0,603623227 |
| b0966 | 318 | 0,56 | -0,267400922 | 8,156208912 | 0,559100073 | 0,454623064 | 0,603623227 |
| b2019 | 900 | 0,54 | 0,217081862 | 5,317617981 | 0,558983143 | 0,45467024 | 0,603623227 |
| b0315 | 1089 | 0,46 | 0,21914008 | 4,001043857 | 0,555953559 | 0,455895225 | 0,605070173 |
| b0117 | 1854 | 0,54 | 0,21474277 | 4,362110929 | 0,5556207 | 0,45603013 | 0,605070173 |
| b0116 | 1425 | 0,53 | 0,242727688 | 11,08155679 | 0,55511644 | 0,456234623 | 0,60516235 |
| b3237 | 471 | 0,49 | -0,141531456 | 6,419434085 | 0,554289322 | 0,456570357 | 0,605428503 |

| | | | | | | | |
|---|---|---|---|---|---|---|---|
| b1694 | 1596 | 0,47 | 0,541155394 | 1,512156761 | 0,552999929 | 0,457094509 | 0,605944274 |
| b1908 | 666 | 0,53 | -0,164076912 | 7,942702944 | 0,552296323 | 0,457380933 | 0,606077616 |
| b1483 | 927 | 0,55 | -0,265742574 | 4,7175645 | 0,552088603 | 0,457465546 | 0,606077616 |
| b2579 | 384 | 0,48 | 0,301365786 | 7,844073954 | 0,551539831 | 0,457689202 | 0,60619474 |
| b4107 | 444 | 0,52 | 0,208292671 | 4,680919676 | 0,550326211 | 0,458184435 | 0,606671385 |
| b2737 | 1167 | 0,55 | -0,240612157 | 4,06897767 | 0,549725592 | 0,458429839 | 0,606817052 |
| b0562 | 655 | 0,41 | -0,290136486 | 3,354108887 | 0,548040192 | 0,459119578 | 0,60755062 |
| b0730 | 723 | 0,48 | 0,200002336 | 4,021793315 | 0,54720818 | 0,45946068 | 0,60782254 |
| b0232 | 294 | 0,42 | 0,260661968 | 3,770290769 | 0,546500989 | 0,459750923 | 0,607958821 |
| b3006 | 735 | 0,53 | 0,32942459 | 5,699741042 | 0,5462963 | 0,459834985 | 0,607958821 |
| b3592 | 609 | 0,53 | -0,218812436 | 5,598137451 | 0,544492205 | 0,460576951 | 0,608760216 |
| b3520 | 603 | 0,48 | -0,27541135 | 3,645939674 | 0,543700025 | 0,460903348 | 0,608938689 |
| b3205 | 855 | 0,52 | 0,138414265 | 7,790856454 | 0,543441353 | 0,461010007 | 0,608938689 |
| b3241 | 933 | 0,54 | 0,246835979 | 3,680651003 | 0,543175738 | 0,46111957 | 0,608938689 |
| b2233 | 3753 | 0,51 | 0,133215517 | 5,854824137 | 0,541530572 | 0,461799099 | 0,609656425 |
| b2922 | 741 | 0,53 | 0,16290665 | 8,353289177 | 0,54084417 | 0,462083085 | 0,609851705 |
| b1700 | 294 | 0,48 | 0,709960855 | 0,979319435 | 0,540114449 | 0,462385299 | 0,609963678 |
| b4002 | 426 | 0,54 | -0,355450975 | 6,755035152 | 0,539399676 | 0,462681627 | 0,609963678 |
| b4639 | 87 | 0,41 | 0,956509662 | 0,410376926 | 0,539325744 | 0,462712294 | 0,609963678 |
| b4670 | 54 | 0,41 | 0,956509662 | 0,410376926 | 0,539325744 | 0,462712294 | 0,609963678 |
| b3412 | 771 | 0,56 | -0,17545879 | 4,855494027 | 0,538984382 | 0,462853936 | 0,609970992 |
| b3533 | 2619 | 0,55 | 0,229091446 | 3,817092617 | 0,53861192 | 0,463008562 | 0,609995407 |
| b4082 | 1032 | 0,56 | 0,403090996 | 1,904652571 | 0,534025809 | 0,464919226 | 0,612290943 |
| b4648 | 102 | 0,44 | 0,949706479 | 0,531570847 | 0,533774764 | 0,465024179 | 0,612290943 |
| b3912 | 699 | 0,51 | -0,16051373 | 7,981678247 | 0,533381769 | 0,465188553 | 0,612327488 |
| b0294 | 591 | 0,39 | 0,68182687 | 0,77205985 | 0,532048258 | 0,465747 | 0,612882576 |
| b3676 | 348 | 0,52 | 0,26349671 | 3,741962441 | 0,53035196 | 0,466458926 | 0,613638182 |
| b2489 | 759 | 0,56 | 0,255802295 | 2,98935926 | 0,530021191 | 0,46659795 | 0,613638182 |
| b2910 | 330 | 0,47 | 0,171939919 | 7,838713234 | 0,529702547 | 0,466731942 | 0,613638182 |
| b0136 | 597 | 0,41 | 0,389891879 | 1,786394362 | 0,528903994 | 0,467068008 | 0,613832995 |
| b4634 | 309 | 0,53 | 0,481600239 | 2,621449979 | 0,528699747 | 0,467154026 | 0,613832995 |
| b0861 | 669 | 0,52 | 0,148491358 | 7,032702152 | 0,527925576 | 0,467480298 | 0,614081681 |
| b4194 | 306 | 0,51 | 0,299908872 | 2,660974731 | 0,527242685 | 0,467768404 | 0,614103648 |
| b1918 | 669 | 0,53 | -0,170253417 | 5,480953548 | 0,527236413 | 0,467771052 | 0,614103648 |
| b2532 | 741 | 0,55 | -0,215808439 | 7,210432934 | 0,526421466 | 0,468115247 | 0,614140655 |
| b4427 | 109 | 0,39 | 0,947350971 | 0,421896389 | 0,526399708 | 0,468124442 | 0,614140655 |
| b3991 | 771 | 0,58 | -0,335158069 | 3,333029818 | 0,526009515 | 0,468289391 | 0,614140655 |
| b2469 | 1701 | 0,52 | -0,192428495 | 5,431865755 | 0,525872496 | 0,468347336 | 0,614140655 |
| b3605 | 1191 | 0,56 | 0,475933468 | 7,550368336 | 0,52489976 | 0,468759035 | 0,61450073 |
| b1579 | 1158 | 0,42 | 0,268608088 | 2,846227733 | 0,523599938 | 0,469310078 | 0,615043207 |
| b0380 | 213 | 0,49 | -0,179432084 | 6,067559957 | 0,521391603 | 0,470248667 | 0,616002608 |
| b0953 | 168 | 0,54 | 0,233585516 | 10,82856788 | 0,521231036 | 0,470317029 | 0,616002608 |
| b4569 | 1745 | 0,46 | -0,292443085 | 2,820030462 | 0,520791588 | 0,470504208 | 0,616067737 |
| b3549 | 564 | 0,5 | 0,180884415 | 5,118858079 | 0,520277402 | 0,470723374 | 0,616171537 |
| b1398 | 1314 | 0,52 | 0,316404515 | 6,65159522 | 0,519960724 | 0,470858437 | 0,616171537 |
| b4693 | 1134 | 0,54 | -0,347372916 | 2,620126921 | 0,518860133 | 0,471328322 | 0,616606404 |
| b0537 | 1164 | 0,47 | -0,137242319 | 6,110970348 | 0,517709208 | 0,471820508 | 0,617000743 |
| b1692 | 867 | 0,48 | 0,448693384 | 1,827597047 | 0,517327166 | 0,471984069 | 0,617000743 |
| b0864 | 729 | 0,53 | -0,170655891 | 8,116974514 | 0,516879357 | 0,472175904 | 0,617000743 |
| b4413 | 55 | 0,47 | 0,928750157 | 0,432982471 | 0,516227406 | 0,472455415 | 0,617000743 |
| b0075 | 87 | 0,47 | 0,928433949 | 0,421833148 | 0,516227058 | 0,472455564 | 0,617000743 |
| b4545 | 51 | 0,47 | 0,928750111 | 0,410376926 | 0,516226688 | 0,472455723 | 0,617000743 |
| b2024 | 738 | 0,57 | 0,212896481 | 5,680832992 | 0,5156384 | 0,47270817 | 0,617150603 |
| b4431 | 106 | 0,39 | 0,936703844 | 0,410376926 | 0,515212146 | 0,472891221 | 0,6172098 |
| b1192 | 915 | 0,53 | -0,168315706 | 5,307717959 | 0,513802383 | 0,47349745 | 0,617821127 |
| b2447 | 594 | 0,52 | 0,280271866 | 3,073816434 | 0,512119107 | 0,474222947 | 0,618587674 |
| b3284 | 474 | 0,46 | -0,167631798 | 8,917687699 | 0,510922377 | 0,474739838 | 0,619007332 |
| b2323 | 1221 | 0,57 | 0,210401362 | 10,215598 | 0,510734926 | 0,474820885 | 0,619007332 |
| b1104 | 378 | 0,49 | 0,139553376 | 7,231827004 | 0,507913732 | 0,476043383 | 0,620124935 |
| b3467 | 360 | 0,51 | 0,243102872 | 4,153751183 | 0,506908022 | 0,476480422 | 0,620124935 |
| b2189 | 77 | 0,66 | 0,927556907 | 0,410376926 | 0,506843881 | 0,476508317 | 0,620124935 |
| b2814 | 77 | 0,66 | 0,927556907 | 0,410376926 | 0,506843881 | 0,476508317 | 0,620124935 |
| b2815 | 77 | 0,66 | 0,927556907 | 0,410376926 | 0,506843881 | 0,476508317 | 0,620124935 |
| b2816 | 77 | 0,66 | 0,927556907 | 0,410376926 | 0,506843881 | 0,476508317 | 0,620124935 |
| b3873 | 981 | 0,44 | 0,28468093 | 3,953778949 | 0,506310928 | 0,476740203 | 0,620246615 |
| b4135 | 576 | 0,53 | 0,151305283 | 7,764353539 | 0,505990243 | 0,47687982 | 0,620248216 |
| b3515 | 729 | 0,42 | 0,157163261 | 7,996866896 | 0,504725236 | 0,47743122 | 0,620785242 |
| b4387 | 645 | 0,56 | 0,170741549 | 5,293332976 | 0,503009789 | 0,478180624 | 0,621579338 |
| b4586 | 480 | 0,31 | 0,832855916 | 0,571074127 | 0,501376826 | 0,478895781 | 0,622328469 |
| b3726 | 891 | 0,56 | 0,417649173 | 4,744552341 | 0,498630182 | 0,480102628 | 0,623544287 |
| b4623 | 2597 | 0,51 | 0,271487968 | 3,384695771 | 0,498614292 | 0,480109624 | 0,623544287 |
| b4685 | 81 | 0,4 | 0,916620837 | 0,4442836 | 0,496314241 | 0,481124105 | 0,62450048 |
| b4667 | 60 | 0,4 | 0,916467304 | 0,410376926 | 0,496313272 | 0,481124533 | 0,62450048 |
| b2847 | 810 | 0,4 | -0,3616633 | 2,551135379 | 0,495360083 | 0,481545987 | 0,624866565 |
| b2265 | 1296 | 0,53 | 0,163848041 | 6,885332588 | 0,493172547 | 0,482515507 | 0,625937237 |
| b0087 | 1083 | 0,53 | -0,153577177 | 7,279567158 | 0,49283568 | 0,482665092 | 0,625937237 |
| b1472 | 279 | 0,42 | 0,772084521 | 0,488017012 | 0,492554393 | 0,482790056 | 0,625937237 |
| b4361 | 738 | 0,51 | 0,135902827 | 5,585389473 | 0,491016056 | 0,483474415 | 0,626643241 |
| b4590 | 258 | 0,44 | 0,768825513 | 0,68022837 | 0,489595468 | 0,484107813 | 0,627282803 |
| b1181 | 462 | 0,48 | -0,188318209 | 4,679222324 | 0,48837325 | 0,484653859 | 0,627808843 |
| b2016 | 825 | 0,52 | 0,131917813 | 7,558792198 | 0,487514869 | 0,485037964 | 0,628124864 |
| b3849 | 1452 | 0,53 | -0,136313456 | 6,647901898 | 0,486615539 | 0,485440932 | 0,628465123 |
| b2576 | 1335 | 0,55 | 0,121953248 | 6,484544044 | 0,485103613 | 0,486119639 | 0,629162062 |
| b4643 | 15 | 0,67 | 0,907490402 | 0,410376926 | 0,484424906 | 0,486424823 | 0,629193667 |
| b4645 | 14 | 0,79 | 0,907490402 | 0,410376926 | 0,484424906 | 0,486424823 | 0,629193667 |
| b0415 | 471 | 0,52 | 0,142662783 | 6,806322632 | 0,483428687 | 0,486873353 | 0,629592144 |
| b2829 | 2247 | 0,54 | 0,135806958 | 8,551219968 | 0,482957924 | 0,487085545 | 0,62968486 |
| b2900 | 312 | 0,44 | 0,165933294 | 5,007918107 | 0,481992824 | 0,487521033 | 0,630066111 |
| b4129 | 1518 | 0,47 | -0,188916318 | 10,45267457 | 0,480453191 | 0,488217112 | 0,630622974 |
| b2637 | 468 | 0,51 | -0,217287107 | 3,879426114 | 0,480417394 | 0,488233315 | 0,630622974 |
| b0798 | 483 | 0,5 | -0,234970047 | 3,91384465 | 0,479788083 | 0,488518322 | 0,630649952 |
| b0138 | 570 | 0,43 | 0,34768781 | 2,213921007 | 0,479749915 | 0,488535617 | 0,630649952 |
| b3010 | 957 | 0,5 | 0,199215903 | 4,7850323 | 0,478795672 | 0,488968335 | 0,6310268 |
| b3059 | 618 | 0,54 | -0,154545695 | 4,925559997 | 0,477079879 | 0,489747997 | 0,631781133 |

| | | | | | | | |
|---|---|---|---|---|---|---|---|
| b1825 | 288 | 0,43 | 0,188340667 | 5,061085899 | 0,476889202 | 0,489834769 | 0,631781133 |
| b1699 | 1290 | 0,55 | 0,24186452 | 2,826527619 | 0,475162816 | 0,490621571 | 0,63250959 |
| b4374 | 678 | 0,54 | 0,148905994 | 6,030210956 | 0,475030844 | 0,490681804 | 0,63250959 |
| b1140 | 1128 | 0,45 | -0,211248949 | 4,034392395 | 0,472550115 | 0,49181633 | 0,633789761 |
| b4253 | 1815 | 0,32 | -0,282859243 | 3,136874892 | 0,471585581 | 0,49225863 | 0,634177402 |
| b2176 | 1557 | 0,51 | 0,153149431 | 4,885671494 | 0,470991655 | 0,492531314 | 0,634269853 |
| b2184 | 1761 | 0,53 | -0,185772707 | 4,704216153 | 0,470546091 | 0,492736048 | 0,634269853 |
| b4267 | 1032 | 0,47 | -0,21198551 | 3,66659702 | 0,470504999 | 0,492754937 | 0,634269853 |
| b1729 | 1392 | 0,52 | 0,168211142 | 5,252795262 | 0,468446724 | 0,493702618 | 0,635307244 |
| b0107 | 1386 | 0,53 | -0,251739559 | 4,071142751 | 0,467308992 | 0,49422777 | 0,635800478 |
| b2277 | 1530 | 0,55 | 0,312510073 | 9,984974692 | 0,466893379 | 0,494419842 | 0,635865059 |
| b1151 | 412 | 0,56 | 0,596492762 | 1,326769129 | 0,466433431 | 0,494632549 | 0,635956135 |
| b1348 | 195 | 0,41 | 0,748003629 | 0,582020033 | 0,4628949 | 0,496274137 | 0,637883763 |
| b2076 | 3078 | 0,56 | -0,188916647 | 5,135918468 | 0,460963647 | 0,497173955 | 0,63885713 |
| b3374 | 786 | 0,57 | -0,248418686 | 3,728136786 | 0,459381492 | 0,497913176 | 0,639596357 |
| b3954 | 852 | 0,53 | 0,184708515 | 4,273507486 | 0,458930637 | 0,498124166 | 0,639596357 |
| b2260 | 1356 | 0,56 | -0,146698332 | 6,562498678 | 0,458631938 | 0,498264033 | 0,639596357 |
| b0729 | 870 | 0,54 | 0,274517847 | 11,38589116 | 0,458512344 | 0,498320053 | 0,639596357 |
| b0422 | 243 | 0,55 | -0,215659315 | 5,323480932 | 0,4579506 | 0,498583323 | 0,639751061 |
| b0089 | 1245 | 0,53 | -0,141696689 | 7,749058358 | 0,457568734 | 0,498762426 | 0,639797708 |
| b2710 | 1440 | 0,53 | 0,225656924 | 4,20816282 | 0,4571824 | 0,498943735 | 0,639847159 |
| b0560 | 546 | 0,53 | -0,543731069 | 1,334627424 | 0,456864268 | 0,49909312 | 0,639851249 |
| b0173 | 1197 | 0,53 | -0,145449899 | 6,873706491 | 0,455795695 | 0,499595444 | 0,639851249 |
| b3248 | 594 | 0,54 | 0,152349123 | 5,836999071 | 0,455555126 | 0,499708651 | 0,639851249 |
| b3958 | 1005 | 0,54 | 0,224493305 | 4,17823652 | 0,454641567 | 0,50013895 | 0,639851249 |
| b4704 | 69 | 0,39 | 0,87552857 | 0,433172695 | 0,454088554 | 0,500399733 | 0,639851249 |
| b4630 | 90 | 0,39 | 0,874908501 | 0,421875775 | 0,454088257 | 0,500399873 | 0,639851249 |
| b4663 | 87 | 0,39 | 0,875330592 | 0,410376926 | 0,454087943 | 0,500400021 | 0,639851249 |
| b4680 | 72 | 0,39 | 0,875330592 | 0,410376926 | 0,454087943 | 0,500400021 | 0,639851249 |
| b4424 | 68 | 0,49 | 0,868589432 | 0,421994664 | 0,454040551 | 0,500422381 | 0,639851249 |
| b2348 | 75 | 0,49 | 0,868589432 | 0,421875775 | 0,454040547 | 0,500422382 | 0,639851249 |
| b1626 | 441 | 0,49 | 0,301648541 | 4,707534503 | 0,45383943 | 0,500517287 | 0,639851249 |
| b3961 | 918 | 0,55 | -0,133494222 | 7,653569566 | 0,453197539 | 0,500820392 | 0,640056173 |
| b3017 | 1413 | 0,56 | -0,117512198 | 7,158176671 | 0,451584925 | 0,501583258 | 0,640848393 |
| b2145 | 240 | 0,43 | 0,39912576 | 2,002334448 | 0,451080739 | 0,501822175 | 0,640970929 |
| b3499 | 843 | 0,54 | 0,235714318 | 5,373904829 | 0,450660107 | 0,502021647 | 0,641043026 |
| b1429 | 993 | 0,53 | -0,171014662 | 5,26365105 | 0,449717002 | 0,502469376 | 0,641048679 |
| b3660 | 924 | 0,49 | -0,166332497 | 5,173992266 | 0,449572976 | 0,502537811 | 0,641048679 |
| b3874 | 1266 | 0,45 | 0,460011112 | 3,436603384 | 0,449455114 | 0,502593826 | 0,641048679 |
| b2278 | 1842 | 0,57 | 0,33774266 | 10,6393466 | 0,449445946 | 0,502598183 | 0,641048679 |
| b4369 | 87 | 0,6 | 0,567079692 | 1,261694515 | 0,448918112 | 0,502849174 | 0,641186344 |
| b0844 | 816 | 0,46 | 0,177397727 | 5,071313458 | 0,448241818 | 0,503171072 | 0,641414318 |
| b2715 | 1458 | 0,56 | 0,213527914 | 4,222841648 | 0,445920892 | 0,50427845 | 0,642643165 |
| b3889 | 213 | 0,46 | 0,731373969 | 0,727665819 | 0,445480426 | 0,504489079 | 0,642728838 |
| b1061 | 246 | 0,48 | 0,254959493 | 3,859067124 | 0,443436759 | 0,505468326 | 0,643793417 |
| b1733 | 750 | 0,51 | -0,188656147 | 4,294495942 | 0,442247417 | 0,506039713 | 0,644338066 |
| b0793 | 1134 | 0,55 | -0,157925175 | 5,26586742 | 0,441117087 | 0,506583778 | 0,644847626 |
| b0702 | 327 | 0,31 | 0,478563051 | 0,972123685 | 0,439034545 | 0,507588808 | 0,645943508 |
| b3899 | 1452 | 0,56 | 0,401168508 | 1,957849068 | 0,437103527 | 0,508523783 | 0,646949644 |
| b3038 | 1161 | 0,5 | 0,13075837 | 8,162641203 | 0,436380368 | 0,508874691 | 0,647212363 |
| b2626 | 627 | 0,4 | 0,517050154 | 1,361869615 | 0,435187023 | 0,50945467 | 0,647556252 |
| b4365 | 726 | 0,45 | 0,613193146 | 0,806656899 | 0,434952569 | 0,509568751 | 0,647556252 |
| b0806 | 261 | 0,52 | 0,19672234 | 4,429177601 | 0,434932508 | 0,509578514 | 0,647556252 |
| b2856 | 633 | 0,36 | 0,637030605 | 0,736669423 | 0,433484033 | 0,51028432 | 0,648184448 |
| b0042 | 942 | 0,57 | -0,251200967 | 2,400592092 | 0,433324629 | 0,510362097 | 0,648184448 |
| b2820 | 3543 | 0,55 | 0,120199078 | 7,340706031 | 0,429963389 | 0,512006913 | 0,649926047 |
| b3079 | 1071 | 0,52 | 0,395244689 | 2,4759283 | 0,429929796 | 0,512023398 | 0,649926047 |
| b4433 | 121 | 0,46 | 0,847333192 | 0,410376926 | 0,429094091 | 0,512433799 | 0,650262822 |
| b4602 | 90 | 0,5 | 0,314985779 | 2,347871209 | 0,427002178 | 0,513463612 | 0,650400393 |
| b3950 | 321 | 0,49 | 0,312782916 | 2,27099773 | 0,42595698 | 0,513979494 | 0,650400393 |
| b0743 | 76 | 0,5 | 0,839929347 | 0,410376926 | 0,425341752 | 0,514283577 | 0,650400393 |
| b0745 | 76 | 0,5 | 0,839929347 | 0,410376926 | 0,425341752 | 0,514283577 | 0,650400393 |
| b0747 | 76 | 0,5 | 0,839929347 | 0,410376926 | 0,425341752 | 0,514283577 | 0,650400393 |
| b0748 | 76 | 0,5 | 0,839929347 | 0,410376926 | 0,425341752 | 0,514283577 | 0,650400393 |
| b0749 | 76 | 0,5 | 0,839929347 | 0,410376926 | 0,425341752 | 0,514283577 | 0,650400393 |
| b2404 | 76 | 0,5 | 0,839929347 | 0,410376926 | 0,425341752 | 0,514283577 | 0,650400393 |
| b4422 | 66 | 0,5 | 0,839929347 | 0,410376926 | 0,425341752 | 0,514283577 | 0,650400393 |
| b4444 | 88 | 0,5 | 0,839929347 | 0,410376926 | 0,425341752 | 0,514283577 | 0,650400393 |
| b4597 | 64 | 0,5 | 0,839929347 | 0,410376926 | 0,425341752 | 0,514283577 | 0,650400393 |
| b4706 | 66 | 0,5 | 0,839929347 | 0,410376926 | 0,425341752 | 0,514283577 | 0,650400393 |
| b2267 | 462 | 0,48 | 0,142358635 | 5,457850392 | 0,42475064 | 0,514576036 | 0,650586683 |
| b4578 | 432 | 0,46 | 0,233194677 | 3,725249444 | 0,424397023 | 0,51475113 | 0,650624524 |
| b1787 | 504 | 0,51 | 0,165711418 | 5,485844558 | 0,421781608 | 0,516049393 | 0,652039115 |
| b1680 | 1221 | 0,55 | -0,206321808 | 7,612221011 | 0,421556778 | 0,516161263 | 0,652039115 |
| b0335 | 1887 | 0,58 | 0,192142145 | 3,96351899 | 0,421124879 | 0,516376284 | 0,652126939 |
| b2096 | 855 | 0,53 | 0,168251356 | 12,32258535 | 0,419995174 | 0,516939453 | 0,652559476 |
| b4327 | 912 | 0,51 | -0,158245855 | 4,681890546 | 0,419853862 | 0,517009973 | 0,652559476 |
| b1025 | 1359 | 0,39 | 0,236523936 | 3,836257152 | 0,417413608 | 0,518230433 | 0,653641709 |
| b0584 | 2241 | 0,54 | -0,287031739 | 4,482847378 | 0,417254681 | 0,518310093 | 0,653641709 |
| b1595 | 894 | 0,53 | 0,196091234 | 4,275010238 | 0,416989089 | 0,518443266 | 0,653641709 |
| b0473 | 1875 | 0,53 | 0,323814973 | 9,657962218 | 0,416974162 | 0,518450753 | 0,653641709 |
| b1602 | 1389 | 0,53 | -0,154837112 | 9,097732695 | 0,415060814 | 0,519411948 | 0,65466939 |
| b1321 | 1398 | 0,54 | 0,275000157 | 6,112201052 | 0,414170768 | 0,519860143 | 0,655050087 |
| b2598 | 48 | 0,38 | 0,833203677 | 0,410376926 | 0,412940967 | 0,520480549 | 0,655375283 |
| b4666 | 60 | 0,38 | 0,833203677 | 0,410376926 | 0,412940967 | 0,520480549 | 0,655375283 |
| b2592 | 2574 | 0,51 | 0,323410267 | 10,70226429 | 0,412789806 | 0,520556896 | 0,655375283 |
| b3441 | 489 | 0,52 | 0,188319116 | 4,017961301 | 0,41113681 | 0,521393069 | 0,656243677 |
| b4558 | 204 | 0,52 | -0,152250291 | 5,563512521 | 0,410705717 | 0,521611529 | 0,656334327 |
| b1696 | 912 | 0,42 | -0,177696993 | 4,164314411 | 0,409601088 | 0,522172047 | 0,65685521 |
| b0425 | 912 | 0,52 | -0,142335795 | 6,482792661 | 0,408560614 | 0,522700986 | 0,657336089 |
| b0868 | 1014 | 0,54 | -0,137931046 | 5,853796647 | 0,406867737 | 0,523563615 | 0,658028562 |
| b0218 | 357 | 0,38 | 0,700523777 | 0,641947053 | 0,406650855 | 0,523674313 | 0,658028562 |
| b2155 | 1992 | 0,53 | 0,301704007 | 4,227802133 | 0,406616062 | 0,523692075 | 0,658028562 |
| b4183 | 660 | 0,47 | 0,524220318 | 1,290781287 | 0,404835879 | 0,524602316 | 0,658857464 |

| | | | | | | | |
|---|---|---|---|---|---|---|---|
| b1846 | 660 | 0,53 | -0,171503912 | 5,64922782 | 0,404751054 | 0,524645759 | 0,658857464 |
| b2438 | 501 | 0,58 | -0,208533771 | 5,093919146 | 0,404295181 | 0,524879342 | 0,658966165 |
| b0141 | 585 | 0,45 | 0,665577954 | 0,747050641 | 0,401874718 | 0,526122659 | 0,65997556 |
| b0651 | 936 | 0,51 | -0,161365919 | 7,407786331 | 0,401834401 | 0,526143413 | 0,65997556 |
| b1689 | 357 | 0,46 | -0,280035823 | 2,079912426 | 0,401824368 | 0,526148578 | 0,65997556 |
| b0826 | 750 | 0,55 | -0,155861619 | 6,162142797 | 0,401383999 | 0,526375366 | 0,65997556 |
| b0569 | 2238 | 0,53 | -0,154301957 | 5,349861237 | 0,401088192 | 0,526527804 | 0,65997556 |
| b4629 | 49 | 0,51 | 0,81590104 | 0,410376926 | 0,401012456 | 0,526566845 | 0,65997556 |
| b3206 | 273 | 0,49 | 0,17845972 | 4,38077151 | 0,400606565 | 0,526776167 | 0,660053336 |
| b2098 | 1278 | 0,5 | -0,21988039 | 3,596725498 | 0,399612535 | 0,527289428 | 0,660511798 |
| b0037 | 1554 | 0,51 | 0,214670975 | 3,843005265 | 0,399228569 | 0,527487925 | 0,660563968 |
| b2013 | 1059 | 0,5 | 0,245620218 | 2,887734064 | 0,398961947 | 0,527625838 | 0,660563968 |
| b0003 | 933 | 0,56 | 0,15300495 | 8,244227085 | 0,398660447 | 0,52778187 | 0,660574795 |
| b4198 | 687 | 0,57 | -0,202294106 | 3,242296555 | 0,397906399 | 0,528172465 | 0,660734385 |
| b4640 | 533 | 0,41 | -0,245707205 | 2,952598867 | 0,397845145 | 0,528204217 | 0,660734385 |
| b1594 | 1221 | 0,52 | -0,161864409 | 7,278057456 | 0,3966925 | 0,52880235 | 0,661298028 |
| b4550 | 219 | 0,43 | 0,212075464 | 3,87338179 | 0,39610223 | 0,529109124 | 0,661497098 |
| b4054 | 1194 | 0,53 | 0,168916865 | 6,44955779 | 0,39493105 | 0,529718754 | 0,662074583 |
| b2164 | 1251 | 0,51 | -0,187489248 | 4,424882393 | 0,394151889 | 0,530125026 | 0,662237314 |
| b4286 | 330 | 0,54 | 0,282071125 | 2,380017988 | 0,393834996 | 0,530290422 | 0,662237314 |
| b1219 | 354 | 0,52 | 0,122084185 | 6,867765481 | 0,393831556 | 0,530292218 | 0,662237314 |
| b3728 | 1041 | 0,51 | 0,230171813 | 5,749888332 | 0,393317536 | 0,530560698 | 0,662388036 |
| b3661 | 819 | 0,47 | -0,152592931 | 5,253071982 | 0,392585843 | 0,530943295 | 0,662681105 |
| b2744 | 762 | 0,55 | 0,105442495 | 7,372174947 | 0,391912641 | 0,531295746 | 0,662936396 |
| b0909 | 765 | 0,5 | 0,258728051 | 2,898091387 | 0,391140209 | 0,531700668 | 0,663256997 |
| b3046 | 3805 | 0,42 | 0,262925125 | 2,933672633 | 0,38860246 | 0,533034924 | 0,664733375 |
| b0442 | 372 | 0,55 | 0,195075856 | 3,735418176 | 0,388325643 | 0,533180831 | 0,664733375 |
| b3881 | 879 | 0,54 | -0,220698603 | 3,528351687 | 0,385242466 | 0,534810825 | 0,666580122 |
| b2252 | 603 | 0,43 | 0,357358107 | 1,835047348 | 0,384338263 | 0,535290567 | 0,666992584 |
| b0106 | 1203 | 0,52 | 0,212275793 | 3,189617022 | 0,384039917 | 0,535449032 | 0,667004603 |
| b0323 | 951 | 0,56 | 0,272009984 | 2,28214386 | 0,38370486 | 0,535627097 | 0,667041025 |
| b4117 | 2268 | 0,53 | -0,166955575 | 4,458148132 | 0,383146871 | 0,535923877 | 0,667078872 |
| b2491 | 2013 | 0,53 | 0,187038384 | 3,695215679 | 0,382832898 | 0,536091003 | 0,667078872 |
| b4450 | 121 | 0,52 | -0,24559203 | 2,557311161 | 0,382341635 | 0,53635269 | 0,667078872 |
| b3648 | 624 | 0,49 | 0,104282166 | 7,22708058 | 0,381916753 | 0,536579204 | 0,667078872 |
| b2276 | 1458 | 0,57 | 0,274302145 | 10,06242525 | 0,381460203 | 0,536822795 | 0,667078872 |
| b2508 | 1467 | 0,55 | 0,245671201 | 8,32942852 | 0,380062751 | 0,537569655 | 0,667078872 |
| b0001 | 66 | 0,52 | 0,793031537 | 0,477334582 | 0,380021807 | 0,537591566 | 0,667078872 |
| b4420 | 67 | 0,52 | 0,793279855 | 0,433254521 | 0,380020887 | 0,537592058 | 0,667078872 |
| b4429 | 56 | 0,52 | 0,793050448 | 0,421554886 | 0,380020619 | 0,537592202 | 0,667078872 |
| b0666 | 77 | 0,52 | 0,793279822 | 0,410376926 | 0,380020353 | 0,537592344 | 0,667078872 |
| b0673 | 77 | 0,52 | 0,793279822 | 0,410376926 | 0,380020353 | 0,537592344 | 0,667078872 |
| b4625 | 77 | 0,52 | 0,793279822 | 0,410376926 | 0,380020353 | 0,537592344 | 0,667078872 |
| b4701 | 56 | 0,52 | 0,793279822 | 0,410376926 | 0,380020353 | 0,537592344 | 0,667078872 |
| b2297 | 2145 | 0,56 | 0,175167403 | 9,339587603 | 0,37940644 | 0,537921071 | 0,667302031 |
| b4197 | 855 | 0,57 | -0,230031328 | 3,716276271 | 0,378761578 | 0,538266765 | 0,66754611 |
| b4124 | 720 | 0,49 | -0,131573737 | 5,631675159 | 0,376663989 | 0,539394043 | 0,668759087 |
| b3861 | 1431 | 0,42 | 0,276412389 | 2,663384863 | 0,376332735 | 0,539572459 | 0,668795288 |
| b1551 | 156 | 0,44 | 0,793801027 | 0,455059768 | 0,374279037 | 0,540681015 | 0,669301545 |
| b1975 | 90 | 0,63 | -0,270623917 | 2,647470529 | 0,374167491 | 0,540741346 | 0,669301545 |
| b1982 | 1455 | 0,5 | 0,157545768 | 7,534895571 | 0,373973265 | 0,540846424 | 0,669301545 |
| b4662 | 132 | 0,47 | 0,788680493 | 0,410376926 | 0,373104388 | 0,541316954 | 0,669301545 |
| b4669 | 51 | 0,37 | 0,790235079 | 0,421787673 | 0,373090024 | 0,541324739 | 0,669301545 |
| b4585 | 84 | 0,37 | 0,790235079 | 0,410376926 | 0,373089769 | 0,541324878 | 0,669301545 |
| b4614 | 30 | 0,37 | 0,790235079 | 0,410376926 | 0,373089769 | 0,541324878 | 0,669301545 |
| b4664 | 60 | 0,37 | 0,790235079 | 0,410376926 | 0,373089769 | 0,541324878 | 0,669301545 |
| b4687 | 81 | 0,37 | 0,790235079 | 0,410376926 | 0,373089769 | 0,541324878 | 0,669301545 |
| b1978 | 7077 | 0,5 | 0,168770821 | 6,197936287 | 0,372579841 | 0,541601381 | 0,66945874 |
| b4191 | 756 | 0,51 | -0,174104268 | 4,82113624 | 0,372046144 | 0,541891052 | 0,669600664 |
| b4415 | 153 | 0,48 | 0,513674915 | 0,917936749 | 0,371817946 | 0,542014996 | 0,669600664 |
| b1649 | 600 | 0,52 | 0,226279608 | 3,683743679 | 0,370634236 | 0,542658758 | 0,67021123 |
| b2868 | 480 | 0,52 | -0,196860468 | 3,673033827 | 0,369563069 | 0,54324253 | 0,670747388 |
| b2777 | 672 | 0,52 | 0,147593438 | 6,703004864 | 0,368994069 | 0,543553098 | 0,670790277 |
| b4589 | 195 | 0,44 | 0,457441404 | 1,513305521 | 0,36895105 | 0,543576592 | 0,670790277 |
| b4063 | 465 | 0,52 | 0,217272336 | 3,391076963 | 0,368537113 | 0,543802749 | 0,670884646 |
| b0410 | 348 | 0,47 | 0,186664195 | 4,446429191 | 0,36645172 | 0,544944766 | 0,672108542 |
| b2034 | 993 | 0,36 | 0,171525065 | 6,921159766 | 0,36488495 | 0,545805697 | 0,672974709 |
| b1404 | 1152 | 0,46 | 0,778537492 | 0,410376926 | 0,364353442 | 0,54609833 | 0,672974709 |
| b4284 | 1152 | 0,46 | 0,778537492 | 0,410376926 | 0,364353442 | 0,54609833 | 0,672974709 |
| b4156 | 1503 | 0,47 | 0,212821955 | 4,298571237 | 0,362553999 | 0,54709122 | 0,672974709 |
| b3772 | 1545 | 0,59 | -0,150872944 | 6,080618661 | 0,362105126 | 0,547339421 | 0,672974709 |
| b1580 | 1020 | 0,45 | 0,239501475 | 2,814005602 | 0,361530293 | 0,547657576 | 0,672974709 |
| b3797 | 77 | 0,53 | 0,770366578 | 0,563142894 | 0,360948532 | 0,547979916 | 0,672974709 |
| b4451 | 90 | 0,53 | 0,770442591 | 0,44467613 | 0,360946551 | 0,547981014 | 0,672974709 |
| b1665 | 77 | 0,53 | 0,770442535 | 0,410376926 | 0,360945794 | 0,547981434 | 0,672974709 |
| b1977 | 76 | 0,53 | 0,770442535 | 0,410376926 | 0,360945794 | 0,547981434 | 0,672974709 |
| b1984 | 76 | 0,53 | 0,770442535 | 0,410376926 | 0,360945794 | 0,547981434 | 0,672974709 |
| b1986 | 76 | 0,53 | 0,770442535 | 0,410376926 | 0,360945794 | 0,547981434 | 0,672974709 |
| b1989 | 76 | 0,53 | 0,770442535 | 0,410376926 | 0,360945794 | 0,547981434 | 0,672974709 |
| b4430 | 68 | 0,53 | 0,770442535 | 0,410376926 | 0,360945794 | 0,547981434 | 0,672974709 |
| b4700 | 59 | 0,53 | 0,770442535 | 0,410376926 | 0,360945794 | 0,547981434 | 0,672974709 |
| b3508 | 648 | 0,46 | -0,200091158 | 6,992334134 | 0,360644065 | 0,548148756 | 0,672974709 |
| b0833 | 2349 | 0,42 | 0,214217215 | 3,427704638 | 0,360552389 | 0,548199613 | 0,672974709 |
| b3751 | 891 | 0,53 | -0,186607136 | 9,798330286 | 0,359036582 | 0,549041781 | 0,673710405 |
| b2917 | 2145 | 0,55 | 0,182005963 | 3,590421907 | 0,358578248 | 0,549296902 | 0,673710405 |
| b3013 | 927 | 0,47 | 0,209662083 | 3,564208386 | 0,358508661 | 0,549335656 | 0,673710405 |
| b2441 | 1362 | 0,56 | -0,175782563 | 8,054262651 | 0,358392851 | 0,549400163 | 0,673710405 |
| b3179 | 630 | 0,5 | -0,140765924 | 8,627966086 | 0,354934846 | 0,551332839 | 0,675895455 |
| b0035 | 591 | 0,55 | -0,281721967 | 2,572903229 | 0,352964591 | 0,552439725 | 0,677067227 |
| b0611 | 807 | 0,51 | -0,117076906 | 6,421730604 | 0,351791326 | 0,553100851 | 0,677692186 |
| b0807 | 927 | 0,54 | 0,166913978 | 4,530032552 | 0,350940017 | 0,553581491 | 0,678095721 |
| b2020 | 1305 | 0,58 | -0,188249127 | 5,977973734 | 0,350641498 | 0,553750218 | 0,67811707 |
| b3068 | 507 | 0,54 | -0,109713887 | 7,167234063 | 0,350353762 | 0,553912942 | 0,678131059 |
| b4346 | 1380 | 0,4 | -0,137510053 | 5,176748105 | 0,349018052 | 0,554669514 | 0,678871863 |

| | | | | | | | |
|---|---|---|---|---|---|---|---|
| b2783 | 249 | 0,49 | -0,150921176 | 5,012937539 | 0,34752644 | 0,555516703 | 0,67972314 |
| b4121 | 630 | 0,55 | -0,202225123 | 3,365164325 | 0,346282678 | 0,556224997 | 0,680253794 |
| b2158 | 1050 | 0,53 | 0,153992462 | 4,993902137 | 0,346148432 | 0,556301549 | 0,680253794 |
| b1082 | 1644 | 0,52 | 0,184668039 | 3,911358138 | 0,345965819 | 0,556405714 | 0,680253794 |
| b0969 | 330 | 0,47 | 0,164176017 | 4,661482221 | 0,345064777 | 0,556920224 | 0,680697149 |
| b0696 | 573 | 0,54 | 0,338906193 | 4,719721519 | 0,34380854 | 0,557639067 | 0,681389939 |
| b4270 | 85 | 0,54 | 0,748810933 | 0,421843572 | 0,343368727 | 0,557891153 | 0,681512169 |
| b2003 | 222 | 0,53 | 0,400998185 | 1,456608912 | 0,342046412 | 0,558650369 | 0,682246904 |
| b3270 | 1104 | 0,52 | 0,237572155 | 2,587674476 | 0,34155672 | 0,558932029 | 0,682246904 |
| b0008 | 954 | 0,52 | 0,145608096 | 10,53301056 | 0,341526759 | 0,558949271 | 0,682246904 |
| b3699 | 2415 | 0,55 | 0,118820822 | 8,954618385 | 0,339436822 | 0,560154479 | 0,68353182 |
| b3502 | 1290 | 0,54 | -0,219701921 | 3,575309481 | 0,338132978 | 0,56090889 | 0,684266098 |
| b1715 | 45 | 0,36 | 0,7472337 | 0,410376926 | 0,334698325 | 0,562905553 | 0,686328262 |
| b4673 | 66 | 0,36 | 0,7472337 | 0,410376926 | 0,334698325 | 0,562905553 | 0,686328262 |
| b4636 | 150 | 0,47 | 0,746262608 | 0,455242862 | 0,334205378 | 0,563193239 | 0,686373788 |
| b0575 | 3144 | 0,56 | 0,152272688 | 4,473815204 | 0,334109592 | 0,563249173 | 0,686373788 |
| b2001 | 1533 | 0,5 | 0,246455039 | 4,54613417 | 0,333092474 | 0,563843777 | 0,686911608 |
| b1657 | 1170 | 0,53 | 0,179133824 | 3,497134308 | 0,33107399 | 0,565027367 | 0,688166483 |
| b2695 | 93 | 0,65 | -0,221105765 | 4,613841816 | 0,32890935 | 0,566302009 | 0,689323778 |
| b1158 | 555 | 0,49 | -0,519200851 | 1,053407592 | 0,328724453 | 0,566411143 | 0,689323778 |
| b0545 | 552 | 0,42 | -0,203246311 | 3,529721 | 0,328481443 | 0,566554641 | 0,689323778 |
| b3724 | 726 | 0,53 | -0,223527062 | 6,648167578 | 0,328253981 | 0,566689021 | 0,689323778 |
| b4231 | 996 | 0,52 | -0,229022442 | 5,381611235 | 0,328156603 | 0,566746569 | 0,689323778 |
| b0787 | 714 | 0,42 | -0,271373216 | 2,610523907 | 0,327457503 | 0,567160052 | 0,689639542 |
| b4454 | 66 | 0,55 | 0,729295747 | 0,421896389 | 0,326592782 | 0,567672303 | 0,689888262 |
| b1574 | 53 | 0,55 | 0,729295723 | 0,410376926 | 0,326592538 | 0,567672447 | 0,689888262 |
| b4708 | 174 | 0,41 | 0,741757129 | 0,410376926 | 0,326217663 | 0,567894799 | 0,689971399 |
| b0900 | 909 | 0,47 | 0,198851224 | 3,571758123 | 0,324290098 | 0,569040788 | 0,691176372 |
| b0277 | 627 | 0,45 | 0,166643628 | 3,541186104 | 0,320969634 | 0,571025512 | 0,693399172 |
| b4653 | 132 | 0,55 | 0,613699995 | 0,541362752 | 0,320236838 | 0,571465349 | 0,693745312 |
| b2872 | 1212 | 0,51 | -0,207242383 | 3,02572191 | 0,319410467 | 0,571962148 | 0,694160398 |
| b4468 | 1053 | 0,58 | -0,637798067 | 8,636836606 | 0,317422358 | 0,573160847 | 0,695426885 |
| b0502 | 422 | 0,49 | 0,213733461 | 2,90572635 | 0,316425575 | 0,573763704 | 0,695732273 |
| b1927 | 1488 | 0,5 | 0,124592679 | 8,974267117 | 0,316396496 | 0,573781309 | 0,695732273 |
| b1171 | 330 | 0,45 | 0,19812736 | 3,737178859 | 0,316236451 | 0,573878227 | 0,695732273 |
| b0447 | 459 | 0,52 | 0,157481223 | 4,240883203 | 0,315389022 | 0,57439194 | 0,69616676 |
| b0192 | 711 | 0,52 | -0,132346322 | 5,991037998 | 0,314995395 | 0,574630867 | 0,696268058 |
| b2738 | 639 | 0,54 | 0,216013632 | 3,016099478 | 0,314588517 | 0,574878043 | 0,696372871 |
| b1349 | 810 | 0,47 | 0,236876065 | 3,249831863 | 0,314341657 | 0,575028111 | 0,696372871 |
| b3629 | 936 | 0,27 | -0,182601476 | 4,901063997 | 0,313428254 | 0,575584052 | 0,696857839 |
| b0137 | 606 | 0,43 | 0,283503639 | 2,446688606 | 0,313049439 | 0,575814929 | 0,696949098 |
| b4519 | 165 | 0,47 | 0,719928253 | 0,583237089 | 0,310972935 | 0,577083766 | 0,698081614 |
| b3587 | 324 | 0,47 | 0,720478324 | 0,700393069 | 0,310717507 | 0,577240228 | 0,698081614 |
| b3794 | 741 | 0,53 | 0,131534835 | 5,308498689 | 0,310699889 | 0,577251023 | 0,698081614 |
| b3055 | 621 | 0,53 | 0,252212364 | 5,397958013 | 0,310499856 | 0,577373615 | 0,698081614 |
| b0565 | 954 | 0,43 | 0,180663944 | 7,692189566 | 0,309594963 | 0,577928838 | 0,69856447 |
| b0375 | 624 | 0,39 | -0,401718394 | 1,536617337 | 0,308839634 | 0,578393107 | 0,698937154 |
| b2144 | 720 | 0,48 | -0,142546015 | 4,11164429 | 0,307648421 | 0,579126806 | 0,699635133 |
| b2077 | 1416 | 0,56 | -0,176389871 | 4,231356152 | 0,30678375 | 0,579660544 | 0,700091231 |
| b3459 | 384 | 0,57 | -0,153757293 | 4,460642206 | 0,305421177 | 0,580503621 | 0,70092059 |
| b2901 | 1440 | 0,51 | -0,118101545 | 7,867094996 | 0,30515165 | 0,580670679 | 0,700933472 |
| b0975 | 588 | 0,59 | -0,225090526 | 4,463214227 | 0,304238632 | 0,5812373 | 0,701428535 |
| b0050 | 378 | 0,52 | -0,116834545 | 6,364420178 | 0,303466728 | 0,581717212 | 0,701818719 |
| b3411 | 879 | 0,48 | 0,163445387 | 3,694398362 | 0,302796435 | 0,582134595 | 0,702133276 |
| b1848 | 291 | 0,49 | 0,129475488 | 5,707226351 | 0,300598738 | 0,583507309 | 0,703272275 |
| b3273 | 76 | 0,57 | 0,69897531 | 0,478185471 | 0,299031221 | 0,584490396 | 0,703272275 |
| b0202 | 77 | 0,57 | 0,699003993 | 0,410376926 | 0,29903006 | 0,584491126 | 0,703272275 |
| b0668 | 75 | 0,57 | 0,699003993 | 0,410376926 | 0,29903006 | 0,584491126 | 0,703272275 |
| b0670 | 75 | 0,57 | 0,699003993 | 0,410376926 | 0,29903006 | 0,584491126 | 0,703272275 |
| b2652 | 76 | 0,57 | 0,699003993 | 0,410376926 | 0,29903006 | 0,584491126 | 0,703272275 |
| b2864 | 74 | 0,57 | 0,699003993 | 0,410376926 | 0,29903006 | 0,584491126 | 0,703272275 |
| b3277 | 77 | 0,57 | 0,699003993 | 0,410376926 | 0,29903006 | 0,584491126 | 0,703272275 |
| b3852 | 77 | 0,57 | 0,699003993 | 0,410376926 | 0,29903006 | 0,584491126 | 0,703272275 |
| b4603 | 91 | 0,35 | 0,705061547 | 0,421808067 | 0,297894703 | 0,585205273 | 0,703462733 |
| b4665 | 60 | 0,35 | 0,704487841 | 0,410376926 | 0,297894502 | 0,5852054 | 0,703462733 |
| b1643 | 237 | 0,49 | -0,182201538 | 3,586049367 | 0,297888078 | 0,585209445 | 0,703462733 |
| b4212 | 381 | 0,56 | -0,204404168 | 3,985962669 | 0,297593709 | 0,585394896 | 0,703462733 |
| b2617 | 342 | 0,49 | 0,122036289 | 6,938332182 | 0,297531378 | 0,58543418 | 0,703462733 |
| b0477 | 1305 | 0,52 | -0,142575286 | 5,752115943 | 0,296998651 | 0,585770144 | 0,703677777 |
| b2781 | 792 | 0,51 | 0,101843681 | 6,825760577 | 0,2955565 | 0,5866816 | 0,704102995 |
| b3136 | 1155 | 0,57 | 0,244096115 | 2,632024093 | 0,295185919 | 0,586916277 | 0,704102995 |
| b4655 | 126 | 0,31 | -0,607865781 | 0,499644527 | 0,295050502 | 0,58700208 | 0,704102995 |
| b0203 | 76 | 0,63 | 0,697207996 | 0,410376926 | 0,294948562 | 0,587066688 | 0,704102995 |
| b3276 | 76 | 0,63 | 0,697207996 | 0,410376926 | 0,294948562 | 0,587066688 | 0,704102995 |
| b3853 | 76 | 0,63 | 0,697207996 | 0,410376926 | 0,294948562 | 0,587066688 | 0,704102995 |
| b4195 | 465 | 0,54 | 0,235556749 | 2,97262061 | 0,294255652 | 0,587506226 | 0,704139205 |
| b1589 | 618 | 0,53 | -0,2766977 | 3,115296235 | 0,294197246 | 0,587543306 | 0,704139205 |
| b2358 | 708 | 0,52 | 0,699231038 | 0,573539868 | 0,294158055 | 0,58756819 | 0,704139205 |
| b2240 | 1359 | 0,53 | -0,134893082 | 4,873764286 | 0,293595284 | 0,587925746 | 0,704276868 |
| b3619 | 933 | 0,51 | -0,101047646 | 8,467880535 | 0,293185513 | 0,588186373 | 0,704276868 |
| b0744 | 76 | 0,62 | 0,689969976 | 0,410376926 | 0,289092808 | 0,590802468 | 0,704276868 |
| b0746 | 76 | 0,62 | 0,689969976 | 0,410376926 | 0,289092808 | 0,590802468 | 0,704276868 |
| b2401 | 76 | 0,62 | 0,689969976 | 0,410376926 | 0,289092808 | 0,590802468 | 0,704276868 |
| b2402 | 76 | 0,62 | 0,689969976 | 0,410376926 | 0,289092808 | 0,590802468 | 0,704276868 |
| b2403 | 76 | 0,62 | 0,689969976 | 0,410376926 | 0,289092808 | 0,590802468 | 0,704276868 |
| b2409 | 927 | 0,54 | 0,183970303 | 3,867990992 | 0,288011914 | 0,591497368 | 0,704276868 |
| b2079 | 723 | 0,54 | 0,123182544 | 5,210864474 | 0,287957874 | 0,591532155 | 0,704276868 |
| b4548 | 201 | 0,55 | 0,514215824 | 0,900747212 | 0,287466222 | 0,591848829 | 0,704276868 |
| b3761 | 76 | 0,59 | 0,684518555 | 0,531478586 | 0,285793339 | 0,592928955 | 0,704276868 |
| b0664 | 75 | 0,59 | 0,684833526 | 0,410376926 | 0,285791583 | 0,592930092 | 0,704276868 |
| b0665 | 75 | 0,59 | 0,684833526 | 0,410376926 | 0,285791583 | 0,592930092 | 0,704276868 |
| b1230 | 85 | 0,59 | 0,684833526 | 0,410376926 | 0,285791583 | 0,592930092 | 0,704276868 |
| b1231 | 85 | 0,59 | 0,684833526 | 0,410376926 | 0,285791583 | 0,592930092 | 0,704276868 |
| b2967 | 76 | 0,59 | 0,684833526 | 0,410376926 | 0,285791583 | 0,592930092 | 0,704276868 |

| | | | | | | | |
|---|---|---|---|---|---|---|---|
| b3069 | 76 | 0,59 | 0,684833526 | 0,410376926 | 0,285791583 | 0,592930092 | 0,704276868 |
| b4134 | 76 | 0,59 | 0,684833526 | 0,410376926 | 0,285791583 | 0,592930092 | 0,704276868 |
| b3869 | 1050 | 0,55 | 0,136297403 | 4,885431997 | 0,285453525 | 0,593148859 | 0,704276868 |
| b3545 | 77 | 0,61 | 0,684456921 | 0,444415631 | 0,285402254 | 0,593182052 | 0,704276868 |
| b0536 | 77 | 0,61 | 0,684839994 | 0,410376926 | 0,285401689 | 0,593182418 | 0,704276868 |
| b0883 | 88 | 0,61 | 0,684839994 | 0,410376926 | 0,285401689 | 0,593182418 | 0,704276868 |
| b1032 | 88 | 0,61 | 0,684839994 | 0,410376926 | 0,285401689 | 0,593182418 | 0,704276868 |
| b1666 | 77 | 0,61 | 0,684839994 | 0,410376926 | 0,285401689 | 0,593182418 | 0,704276868 |
| b1911 | 76 | 0,61 | 0,684839994 | 0,410376926 | 0,285401689 | 0,593182418 | 0,704276868 |
| b2396 | 76 | 0,61 | 0,684839994 | 0,410376926 | 0,285401689 | 0,593182418 | 0,704276868 |
| b2397 | 76 | 0,61 | 0,684839994 | 0,410376926 | 0,285401689 | 0,593182418 | 0,704276868 |
| b3798 | 87 | 0,61 | 0,684839994 | 0,410376926 | 0,285401689 | 0,593182418 | 0,704276868 |
| b4163 | 76 | 0,61 | 0,684839994 | 0,410376926 | 0,285401689 | 0,593182418 | 0,704276868 |
| b4164 | 76 | 0,61 | 0,684839994 | 0,410376926 | 0,285401689 | 0,593182418 | 0,704276868 |
| b4165 | 76 | 0,61 | 0,684839994 | 0,410376926 | 0,285401689 | 0,593182418 | 0,704276868 |
| b4368 | 87 | 0,61 | 0,684839994 | 0,410376926 | 0,285401689 | 0,593182418 | 0,704276868 |
| b4370 | 87 | 0,61 | 0,684839994 | 0,410376926 | 0,285401689 | 0,593182418 | 0,704276868 |
| b1356 | 477 | 0,42 | -0,128627812 | 5,510991873 | 0,28483841 | 0,593547346 | 0,704276868 |
| b0960 | 2154 | 0,52 | 0,103131148 | 6,017194786 | 0,284477518 | 0,593781398 | 0,704276868 |
| b0971 | 88 | 0,6 | 0,683598014 | 0,410376926 | 0,28418932 | 0,593968443 | 0,704276868 |
| b2691 | 77 | 0,6 | 0,683598014 | 0,410376926 | 0,28418932 | 0,593968443 | 0,704276868 |
| b2692 | 77 | 0,6 | 0,683598014 | 0,410376926 | 0,28418932 | 0,593968443 | 0,704276868 |
| b2693 | 77 | 0,6 | 0,683598014 | 0,410376926 | 0,28418932 | 0,593968443 | 0,704276868 |
| b2694 | 77 | 0,6 | 0,683598014 | 0,410376926 | 0,28418932 | 0,593968443 | 0,704276868 |
| b1821 | 567 | 0,52 | 0,288987236 | 2,687704476 | 0,283926198 | 0,594139319 | 0,704293157 |
| b4075 | 384 | 0,52 | 0,287301507 | 2,045062723 | 0,282638588 | 0,594976985 | 0,705099643 |
| b2091 | 1041 | 0,49 | -0,182275712 | 11,64728036 | 0,28113256 | 0,595959856 | 0,706077736 |
| b4552 | 351 | 0,35 | 0,462150113 | 1,080148335 | 0,280506718 | 0,596369288 | 0,706376096 |
| b3569 | 1179 | 0,49 | 0,130006753 | 4,885106633 | 0,277914395 | 0,598071466 | 0,708205102 |
| b0647 | 555 | 0,43 | 0,550711597 | 0,971341199 | 0,277238592 | 0,59851688 | 0,708545339 |
| b2632 | 864 | 0,54 | 0,185249604 | 3,782090672 | 0,276784694 | 0,598816428 | 0,708712762 |
| b0239 | 1245 | 0,54 | -0,108906548 | 7,093506618 | 0,276367507 | 0,599092026 | 0,708851758 |
| b1053 | 1227 | 0,52 | -0,161878359 | 4,004285773 | 0,275697412 | 0,599535254 | 0,70918897 |
| b4092 | 759 | 0,59 | 0,162958741 | 4,172260891 | 0,274526317 | 0,600311515 | 0,709919845 |
| b1773 | 837 | 0,45 | 0,26579324 | 2,837132976 | 0,272422839 | 0,601711123 | 0,711387299 |
| b4337 | 1233 | 0,54 | 0,147781137 | 4,096896768 | 0,272026006 | 0,601975937 | 0,711512698 |
| b4077 | 1314 | 0,53 | 0,116091744 | 6,383024995 | 0,27116162 | 0,60255361 | 0,711843748 |
| b1954 | 87 | 0,44 | 0,469222964 | 0,868265292 | 0,271131639 | 0,602573668 | 0,711843748 |
| b1407 | 2307 | 0,43 | 0,208491856 | 2,931497047 | 0,268977721 | 0,604018351 | 0,713362385 |
| b0506 | 816 | 0,53 | -0,142351042 | 9,028601985 | 0,264894744 | 0,606777166 | 0,716431839 |
| b0300 | 684 | 0,43 | -0,180957524 | 3,471567096 | 0,264192199 | 0,607254576 | 0,716806692 |
| b3327 | 1197 | 0,51 | 0,201300249 | 3,523611266 | 0,259917823 | 0,610176617 | 0,720066245 |
| b4419 | 108 | 0,56 | 0,649707611 | 0,410376926 | 0,259153173 | 0,610702534 | 0,720307568 |
| b4423 | 108 | 0,56 | 0,649707611 | 0,410376926 | 0,259153173 | 0,610702534 | 0,720307568 |
| b2912 | 549 | 0,52 | 0,194840462 | 4,038483431 | 0,258602712 | 0,61108174 | 0,720565208 |
| b4616 | 140 | 0,51 | 0,653785112 | 0,642638323 | 0,258308134 | 0,611284879 | 0,720615157 |
| b2112 | 282 | 0,44 | -0,111315471 | 5,360269669 | 0,256609391 | 0,612459177 | 0,721778336 |
| b2662 | 1281 | 0,59 | -0,177151972 | 10,46964411 | 0,256189968 | 0,612749865 | 0,721778336 |
| b3330 | 378 | 0,48 | 0,469001894 | 1,189085766 | 0,256182993 | 0,612754701 | 0,721778336 |
| b0892 | 1344 | 0,54 | 0,102441155 | 6,287170244 | 0,255606193 | 0,613154958 | 0,721942443 |
| b1434 | 537 | 0,49 | 0,149895758 | 3,476872401 | 0,255518051 | 0,613216172 | 0,721942443 |
| b1390 | 747 | 0,53 | 0,371992409 | 5,100303421 | 0,253546459 | 0,614588903 | 0,723368556 |
| b3777 | 449 | 0,43 | -0,188166707 | 3,933967772 | 0,252894861 | 0,615044053 | 0,723670401 |
| b2102 | 819 | 0,48 | -0,154353867 | 3,477555025 | 0,252717199 | 0,615168279 | 0,723670401 |
| b4290 | 903 | 0,58 | -0,225214157 | 4,257217425 | 0,252291426 | 0,615466215 | 0,723703682 |
| b1938 | 1659 | 0,55 | -0,300422362 | 1,80720272 | 0,252215315 | 0,615519508 | 0,723703682 |
| b1457 | 483 | 0,27 | 0,36500055 | 1,471687468 | 0,251180059 | 0,616245385 | 0,724367116 |
| b0912 | 285 | 0,49 | -0,112528504 | 9,141384401 | 0,250930623 | 0,616420558 | 0,724383047 |
| b1024 | 2424 | 0,47 | -0,156288531 | 4,770697952 | 0,249843517 | 0,617185284 | 0,725091597 |
| b1578 | 693 | 0,55 | 0,637479711 | 0,410376926 | 0,248456945 | 0,618163692 | 0,726050751 |
| b3957 | 1152 | 0,52 | -0,110221089 | 6,48721797 | 0,247364195 | 0,618937174 | 0,726768775 |
| b4144 | 354 | 0,55 | 0,109175539 | 7,4662727 | 0,247019144 | 0,619181855 | 0,726865656 |
| b1078 | 783 | 0,54 | 0,266484353 | 2,392087657 | 0,246683517 | 0,619420057 | 0,726954883 |
| b2506 | 540 | 0,49 | 0,210054258 | 2,91771122 | 0,24557117 | 0,620210963 | 0,727692549 |
| b3470 | 246 | 0,54 | 0,149868909 | 3,947784315 | 0,245255287 | 0,620435971 | 0,727713028 |
| b4646 | 394 | 0,44 | 0,222726794 | 2,489069387 | 0,245090889 | 0,620553145 | 0,727713028 |
| b3833 | 756 | 0,52 | -0,081522161 | 7,385762203 | 0,243823116 | 0,621458393 | 0,72858397 |
| b3908 | 621 | 0,53 | -0,217419214 | 8,687912174 | 0,240924812 | 0,623538963 | 0,730832017 |
| b1724 | 291 | 0,43 | 0,115378722 | 7,373194828 | 0,238907056 | 0,624996613 | 0,732348972 |
| b1625 | 216 | 0,47 | 0,230615829 | 3,060565895 | 0,237823488 | 0,625782544 | 0,732970203 |
| b3226 | 792 | 0,52 | 0,116545168 | 6,671872867 | 0,237725326 | 0,625853853 | 0,732970203 |
| b1433 | 1176 | 0,53 | -0,143427361 | 3,735003029 | 0,236917726 | 0,626441213 | 0,733466436 |
| b0606 | 1566 | 0,53 | 0,106959871 | 9,092480777 | 0,236191218 | 0,626970654 | 0,733894613 |
| b2661 | 1449 | 0,58 | 0,168992268 | 10,15618334 | 0,235213347 | 0,627684868 | 0,734255684 |
| b2429 | 1425 | 0,57 | -0,137757238 | 5,193739757 | 0,234996725 | 0,627843331 | 0,734255684 |
| b2643 | 459 | 0,54 | 0,282022836 | 2,651891283 | 0,234255088 | 0,628386537 | 0,734255684 |
| b0021 | 504 | 0,55 | 0,61779095 | 0,410376926 | 0,233324588 | 0,629069579 | 0,734255684 |
| b0264 | 504 | 0,55 | 0,61779095 | 0,410376926 | 0,233324588 | 0,629069579 | 0,734255684 |
| b0274 | 504 | 0,55 | 0,61779095 | 0,410376926 | 0,233324588 | 0,629069579 | 0,734255684 |
| b1893 | 504 | 0,55 | 0,61779095 | 0,410376926 | 0,233324588 | 0,629069579 | 0,734255684 |
| b3445 | 504 | 0,55 | 0,61779095 | 0,410376926 | 0,233324588 | 0,629069579 | 0,734255684 |
| b0016 | 1113 | 0,52 | 0,619816944 | 0,410376926 | 0,233296252 | 0,629090406 | 0,734255684 |
| b0582 | 1113 | 0,52 | 0,619816944 | 0,410376926 | 0,233296252 | 0,629090406 | 0,734255684 |
| b2394 | 1113 | 0,52 | 0,619816944 | 0,410376926 | 0,233296252 | 0,629090406 | 0,734255684 |
| b0196 | 405 | 0,53 | 0,145692382 | 5,100233056 | 0,233085985 | 0,629244998 | 0,734255684 |
| b0789 | 1242 | 0,54 | -0,137049209 | 6,275003114 | 0,232250189 | 0,629860344 | 0,734782421 |
| b4689 | 255 | 0,49 | 0,620018503 | 0,410376926 | 0,231605715 | 0,630335763 | 0,735145691 |
| b0840 | 759 | 0,51 | 0,117943954 | 4,954516099 | 0,231204952 | 0,630631811 | 0,735299629 |
| b3663 | 453 | 0,53 | 0,159363619 | 3,735320065 | 0,230777932 | 0,630947602 | 0,735419909 |
| b1087 | 585 | 0,5 | -0,093620776 | 6,977457266 | 0,23040105 | 0,631226615 | 0,735419909 |
| b4626 | 63 | 0,33 | 0,617045042 | 0,410376926 | 0,229462436 | 0,631922711 | 0,735419909 |
| b3664 | 1335 | 0,48 | -0,206651098 | 2,693454022 | 0,22932851 | 0,632022176 | 0,735419909 |
| b0299 | 867 | 0,55 | 0,610730993 | 0,410376926 | 0,228518158 | 0,632624776 | 0,735419909 |
| b0372 | 867 | 0,55 | 0,610730993 | 0,410376926 | 0,228518158 | 0,632624776 | 0,735419909 |

| | | | | | | | |
|---|---|---|---|---|---|---|---|
| b0541 | 867 | 0,55 | 0,610730993 | 0,410376926 | 0,228518158 | 0,632624776 | 0,735419909 |
| b1026 | 867 | 0,55 | 0,610730993 | 0,410376926 | 0,228518158 | 0,632624776 | 0,735419909 |
| b2089 | 867 | 0,55 | 0,610730993 | 0,410376926 | 0,228518158 | 0,632624776 | 0,735419909 |
| b0483 | 342 | 0,45 | -0,137232854 | 5,185659087 | 0,228168099 | 0,632885495 | 0,735419909 |
| b1379 | 423 | 0,47 | 0,096291891 | 6,057941668 | 0,227810577 | 0,633152026 | 0,735419909 |
| b0357 | 276 | 0,53 | -0,120973544 | 4,782113083 | 0,227460251 | 0,633413442 | 0,735419909 |
| b0298 | 300 | 0,5 | 0,614135929 | 0,410376926 | 0,227208222 | 0,633601661 | 0,735419909 |
| b0373 | 300 | 0,5 | 0,614135929 | 0,410376926 | 0,227208222 | 0,633601661 | 0,735419909 |
| b0540 | 300 | 0,5 | 0,614135929 | 0,410376926 | 0,227208222 | 0,633601661 | 0,735419909 |
| b1027 | 300 | 0,5 | 0,614135929 | 0,410376926 | 0,227208222 | 0,633601661 | 0,735419909 |
| b2088 | 300 | 0,5 | 0,614135929 | 0,410376926 | 0,227208222 | 0,633601661 | 0,735419909 |
| b1408 | 606 | 0,47 | 0,495627329 | 0,562121748 | 0,22673367 | 0,633956411 | 0,735419909 |
| b2550 | 1194 | 0,51 | 0,148663018 | 4,66338153 | 0,226696639 | 0,633984113 | 0,735419909 |
| b2645 | 318 | 0,54 | 0,370567082 | 1,382113995 | 0,226653178 | 0,634016628 | 0,735419909 |
| b0481 | 480 | 0,51 | 0,120347173 | 4,429971996 | 0,226340469 | 0,634250693 | 0,735501062 |
| b3262 | 885 | 0,47 | 0,209193045 | 2,401764979 | 0,22561085 | 0,63479759 | 0,735944852 |
| b4535 | 108 | 0,48 | 0,492293826 | 0,890989461 | 0,225168303 | 0,635129836 | 0,736139624 |
| b0361 | 906 | 0,55 | 0,602655421 | 0,410376926 | 0,222727463 | 0,636969541 | 0,73712819 |
| b1402 | 906 | 0,55 | 0,602655421 | 0,410376926 | 0,222727463 | 0,636969541 | 0,73712819 |
| b1996 | 906 | 0,55 | 0,602655421 | 0,410376926 | 0,222727463 | 0,636969541 | 0,73712819 |
| b2860 | 906 | 0,55 | 0,602655421 | 0,410376926 | 0,222727463 | 0,636969541 | 0,73712819 |
| b3045 | 906 | 0,55 | 0,602655421 | 0,410376926 | 0,222727463 | 0,636969541 | 0,73712819 |
| b4273 | 906 | 0,55 | 0,602655421 | 0,410376926 | 0,222727463 | 0,636969541 | 0,73712819 |
| b4514 | 240 | 0,38 | 0,609229094 | 0,520185518 | 0,220960564 | 0,63830899 | 0,738487582 |
| b3553 | 975 | 0,52 | -0,142052165 | 7,592281449 | 0,219574465 | 0,639364346 | 0,739517677 |
| b2516 | 1014 | 0,55 | -0,12173508 | 8,296450252 | 0,219178265 | 0,639666754 | 0,739533946 |
| b1590 | 855 | 0,53 | -0,151407285 | 4,414515776 | 0,219123719 | 0,639708413 | 0,739533946 |
| b0205 | 120 | 0,64 | 0,599807758 | 0,410376926 | 0,218151683 | 0,640451866 | 0,739630318 |
| b2588 | 120 | 0,64 | 0,599807758 | 0,410376926 | 0,218151683 | 0,640451866 | 0,739630318 |
| b3274 | 120 | 0,64 | 0,599807758 | 0,410376926 | 0,218151683 | 0,640451866 | 0,739630318 |
| b3759 | 120 | 0,64 | 0,599807758 | 0,410376926 | 0,218151683 | 0,640451866 | 0,739630318 |
| b4084 | 930 | 0,55 | 0,136197342 | 4,128272888 | 0,21608981 | 0,642035576 | 0,741268277 |
| b1111 | 633 | 0,51 | 0,117171234 | 5,79678408 | 0,215744037 | 0,64230206 | 0,741301855 |
| b2092 | 1358 | 0,51 | 0,144797409 | 13,2420452 | 0,215622941 | 0,64239545 | 0,741301855 |
| b2670 | 450 | 0,49 | 0,122977517 | 7,071741202 | 0,214929222 | 0,64293106 | 0,741611017 |
| b2101 | 747 | 0,51 | 0,133662428 | 4,314902984 | 0,214847416 | 0,642994291 | 0,741611017 |
| b3243 | 930 | 0,53 | -0,122107714 | 5,244065315 | 0,214590346 | 0,643193084 | 0,741649447 |
| b2332 | 822 | 0,45 | 0,107283992 | 5,87737814 | 0,212857816 | 0,644536639 | 0,743007514 |
| b1564 | 240 | 0,47 | 0,106682075 | 5,845165286 | 0,212125364 | 0,645106641 | 0,743189484 |
| b3576 | 468 | 0,45 | 0,377793622 | 0,963617165 | 0,211917908 | 0,645268303 | 0,743189484 |
| b3971 | 120 | 0,63 | 0,589927573 | 0,410376926 | 0,21150105 | 0,645593431 | 0,743189484 |
| b4010 | 120 | 0,63 | 0,589927573 | 0,410376926 | 0,21150105 | 0,645593431 | 0,743189484 |
| b1353 | 489 | 0,37 | -0,202133779 | 2,137882368 | 0,2110167 | 0,64597169 | 0,743189484 |
| b4047 | 255 | 0,29 | 0,540040143 | 0,443810318 | 0,210500416 | 0,646375465 | 0,743189484 |
| b0360 | 366 | 0,53 | 0,588533388 | 0,410376926 | 0,210106057 | 0,64668429 | 0,743189484 |
| b1403 | 366 | 0,53 | 0,588533388 | 0,410376926 | 0,210106057 | 0,64668429 | 0,743189484 |
| b1997 | 366 | 0,53 | 0,588533388 | 0,410376926 | 0,210106057 | 0,64668429 | 0,743189484 |
| b2861 | 366 | 0,53 | 0,588533388 | 0,410376926 | 0,210106057 | 0,64668429 | 0,743189484 |
| b3044 | 366 | 0,53 | 0,588533388 | 0,410376926 | 0,210106057 | 0,64668429 | 0,743189484 |
| b4272 | 366 | 0,53 | 0,588533388 | 0,410376926 | 0,210106057 | 0,64668429 | 0,743189484 |
| b3790 | 675 | 0,56 | 0,102555724 | 5,314231806 | 0,209287548 | 0,647326391 | 0,743736704 |
| b3227 | 1368 | 0,51 | 0,174174518 | 2,745838747 | 0,206802067 | 0,649285542 | 0,744364295 |
| b4412 | 153 | 0,53 | 0,581483942 | 0,410376926 | 0,20598343 | 0,649933933 | 0,744364295 |
| b0259 | 1017 | 0,55 | 0,578428418 | 0,410376926 | 0,205879327 | 0,650016497 | 0,744364295 |
| b0552 | 1017 | 0,55 | 0,578428418 | 0,410376926 | 0,205879327 | 0,650016497 | 0,744364295 |
| b0656 | 1017 | 0,55 | 0,578428418 | 0,410376926 | 0,205879327 | 0,650016497 | 0,744364295 |
| b1331 | 1017 | 0,55 | 0,578428418 | 0,410376926 | 0,205879327 | 0,650016497 | 0,744364295 |
| b2030 | 1017 | 0,55 | 0,578428418 | 0,410376926 | 0,205879327 | 0,650016497 | 0,744364295 |
| b2192 | 1017 | 0,55 | 0,578428418 | 0,410376926 | 0,205879327 | 0,650016497 | 0,744364295 |
| b2982 | 1017 | 0,55 | 0,578428418 | 0,410376926 | 0,205879327 | 0,650016497 | 0,744364295 |
| b3218 | 1017 | 0,55 | 0,578428418 | 0,410376926 | 0,205879327 | 0,650016497 | 0,744364295 |
| b3505 | 1017 | 0,55 | 0,578428418 | 0,410376926 | 0,205879327 | 0,650016497 | 0,744364295 |
| b2871 | 1197 | 0,54 | -0,157188474 | 3,26158905 | 0,205761919 | 0,650109644 | 0,744364295 |
| b3792 | 1251 | 0,54 | -0,101193589 | 6,086436496 | 0,205722072 | 0,650141264 | 0,744364295 |
| b0618 | 1059 | 0,51 | -0,240153394 | 3,132708843 | 0,205650931 | 0,650197728 | 0,744364295 |
| b0989 | 213 | 0,47 | 0,417347116 | 0,933561727 | 0,203862164 | 0,651621314 | 0,745803557 |
| b0516 | 1236 | 0,51 | 0,149509704 | 3,415536538 | 0,203010737 | 0,652301563 | 0,746391525 |
| b0547 | 456 | 0,5 | -0,309249446 | 1,377241453 | 0,202582758 | 0,652644145 | 0,746552483 |
| b0995 | 693 | 0,52 | 0,118952123 | 4,297045525 | 0,202418975 | 0,652775363 | 0,746552483 |
| b1704 | 1047 | 0,53 | -0,238290555 | 5,782006583 | 0,196244157 | 0,657769689 | 0,752072384 |
| b3904 | 1470 | 0,55 | -0,264510711 | 1,910439629 | 0,196036041 | 0,657939646 | 0,752074852 |
| b1650 | 1098 | 0,53 | 0,116201682 | 6,042920725 | 0,195488174 | 0,658387578 | 0,752394983 |
| b3119 | 219 | 0,29 | -0,519814725 | 0,530905159 | 0,19511233 | 0,658695299 | 0,752554762 |
| b1863 | 522 | 0,56 | -0,093097709 | 5,963485107 | 0,193995987 | 0,659611395 | 0,753409346 |
| b3990 | 1134 | 0,55 | -0,14005071 | 3,784945771 | 0,19287178 | 0,660537131 | 0,754274502 |
| b4114 | 1644 | 0,49 | -0,11832967 | 4,971501563 | 0,192183569 | 0,661105432 | 0,754731163 |
| b3428 | 2448 | 0,51 | -0,103823028 | 10,02237429 | 0,189826408 | 0,663061136 | 0,756485709 |
| b1566 | 333 | 0,43 | 0,183698868 | 2,674902672 | 0,189752749 | 0,663122482 | 0,756485709 |
| b2534 | 855 | 0,51 | 0,245563184 | 2,128191957 | 0,189622558 | 0,663230945 | 0,756485709 |
| b2650 | 1374 | 0,34 | -0,49015505 | 0,957815314 | 0,189518755 | 0,663317456 | 0,756485709 |
| b3191 | 294 | 0,53 | -0,103592537 | 6,169281752 | 0,188311857 | 0,664325374 | 0,757432253 |
| b0796 | 672 | 0,54 | 0,108012277 | 5,318244061 | 0,187921433 | 0,66465225 | 0,757432253 |
| b1572 | 129 | 0,47 | 0,450111395 | 0,894911129 | 0,18783255 | 0,664726723 | 0,757432253 |
| b1374 | 591 | 0,45 | -0,215930541 | 2,572319305 | 0,187717202 | 0,664823401 | 0,757432253 |
| b3173 | 1626 | 0,43 | 0,130890242 | 3,710353396 | 0,187449836 | 0,665047627 | 0,757495162 |
| b3196 | 978 | 0,52 | -0,092952246 | 5,757506541 | 0,185938582 | 0,666318619 | 0,758627698 |
| b2686 | 1539 | 0,57 | -0,093010738 | 5,27232457 | 0,18586523 | 0,666380465 | 0,758627698 |
| b3458 | 1110 | 0,54 | -0,187938449 | 3,033369423 | 0,185255423 | 0,666895179 | 0,758717318 |
| b4604 | 165 | 0,4 | -0,472453408 | 0,710121935 | 0,185251054 | 0,666898871 | 0,758717318 |
| b3026 | 1350 | 0,53 | 0,114570145 | 4,835051086 | 0,185170392 | 0,666967031 | 0,758717318 |
| b3287 | 510 | 0,46 | -0,092234079 | 7,932709072 | 0,183179031 | 0,668655347 | 0,760444878 |
| b1150 | 183 | 0,6 | 0,463298458 | 0,682239883 | 0,181354088 | 0,670212132 | 0,762022014 |
| b2406 | 1257 | 0,48 | -0,170009638 | 4,423932012 | 0,181063845 | 0,670460579 | 0,762111162 |
| b0226 | 261 | 0,47 | 0,102049932 | 5,634082473 | 0,180260845 | 0,67114917 | 0,762700452 |

| | | | | | | | |
|---|---|---|---|---|---|---|---|
| b3625 | 699 | 0,35 | -0,095675594 | 4,93024121 | 0,179286229 | 0,671987363 | 0,763459407 |
| b4615 | 439 | 0,33 | -0,476890593 | 0,661602276 | 0,178778902 | 0,672424739 | 0,763632169 |
| b3582 | 861 | 0,56 | -0,168938909 | 2,697958337 | 0,178602062 | 0,672577368 | 0,763632169 |
| b3595 | 945 | 0,48 | 0,16084129 | 3,19819563 | 0,178517299 | 0,672650559 | 0,763632169 |
| b4280 | 1119 | 0,39 | 0,163237009 | 5,831664525 | 0,177724419 | 0,673336177 | 0,763905577 |
| b3037 | 672 | 0,56 | 0,081628075 | 8,000898767 | 0,177692749 | 0,673363599 | 0,763905577 |
| b1662 | 642 | 0,48 | -0,091566629 | 6,829639831 | 0,17764759 | 0,673402708 | 0,763905577 |
| b1788 | 105 | 0,43 | 0,377093592 | 1,026051021 | 0,176815888 | 0,674124017 | 0,764530325 |
| b4085 | 696 | 0,49 | 0,153820613 | 2,895042491 | 0,174529634 | 0,676117159 | 0,766596789 |
| b0580 | 249 | 0,51 | -0,152725192 | 3,960844343 | 0,173319555 | 0,677178309 | 0,767605761 |
| b4698 | 98 | 0,42 | 0,507971638 | 0,488793819 | 0,172872296 | 0,677571622 | 0,767789702 |
| b3212 | 4461 | 0,57 | 0,2481681 | 8,441744403 | 0,172598509 | 0,677812681 | 0,767789702 |
| b3142 | 585 | 0,45 | 0,23970864 | 2,142991416 | 0,172551043 | 0,677854496 | 0,767789702 |
| b1976 | 798 | 0,46 | -0,105589983 | 8,388507271 | 0,172300792 | 0,678075064 | 0,767845487 |
| b0022 | 276 | 0,53 | 0,531033748 | 0,410376926 | 0,171326232 | 0,678935823 | 0,767850204 |
| b0265 | 276 | 0,53 | 0,531033748 | 0,410376926 | 0,171326232 | 0,678935823 | 0,767850204 |
| b0275 | 276 | 0,53 | 0,531033748 | 0,410376926 | 0,171326232 | 0,678935823 | 0,767850204 |
| b1894 | 276 | 0,53 | 0,531033748 | 0,410376926 | 0,171326232 | 0,678935823 | 0,767850204 |
| b4294 | 276 | 0,53 | 0,531033748 | 0,410376926 | 0,171326232 | 0,678935823 | 0,767850204 |
| b0464 | 648 | 0,45 | 0,078267757 | 5,607443957 | 0,17090778 | 0,679306292 | 0,768075378 |
| b1525 | 1389 | 0,53 | 0,111728819 | 5,51485864 | 0,169459963 | 0,680592203 | 0,769149336 |
| b2356 | 270 | 0,53 | 0,528025056 | 0,489143704 | 0,169411061 | 0,680635747 | 0,769149336 |
| b2706 | 360 | 0,55 | 0,240754344 | 2,492363766 | 0,168812767 | 0,681169102 | 0,769149336 |
| b4219 | 639 | 0,54 | -0,10237485 | 7,542223189 | 0,168731279 | 0,68124183 | 0,769149336 |
| b4638 | 51 | 0,31 | 0,528460109 | 0,433177521 | 0,168553896 | 0,681400217 | 0,769149336 |
| b4406 | 201 | 0,51 | 0,085335915 | 6,204630361 | 0,168502974 | 0,681445703 | 0,769149336 |
| b0453 | 573 | 0,54 | -0,138423682 | 9,885345091 | 0,168489895 | 0,681457388 | 0,769149336 |
| b1119 | 912 | 0,53 | -0,074299024 | 7,036471654 | 0,168098841 | 0,681806984 | 0,769350177 |
| b3680 | 894 | 0,44 | 0,154209351 | 3,054509874 | 0,166921418 | 0,682862461 | 0,770347231 |
| b3222 | 876 | 0,58 | -0,096181495 | 5,829519828 | 0,166486833 | 0,683253133 | 0,77052399 |
| b0827 | 1236 | 0,56 | -0,100132681 | 7,35121301 | 0,166364762 | 0,683362976 | 0,77052399 |
| b2854 | 503 | 0,34 | 0,525001947 | 0,561126135 | 0,165656047 | 0,68400163 | 0,771050127 |
| b3624 | 852 | 0,33 | -0,092151186 | 4,816221797 | 0,165052936 | 0,684546375 | 0,771390715 |
| b4143 | 1647 | 0,53 | -0,212239247 | 12,10022416 | 0,164940582 | 0,684647984 | 0,771390715 |
| b1484 | 987 | 0,54 | 0,247904104 | 4,355865334 | 0,163253692 | 0,686178418 | 0,772805251 |
| b3371 | 1023 | 0,54 | -0,162191056 | 3,243252171 | 0,163176902 | 0,686248305 | 0,772805251 |
| b0083 | 366 | 0,5 | 0,089571549 | 5,854958865 | 0,162793624 | 0,686597411 | 0,773004169 |
| b3085 | 504 | 0,51 | 0,130820367 | 3,98884219 | 0,160467324 | 0,688726627 | 0,775206616 |
| b0443 | 399 | 0,46 | 0,129969631 | 4,469785992 | 0,159615999 | 0,689510304 | 0,775734607 |
| b0976 | 399 | 0,57 | 0,173622352 | 3,379756322 | 0,15958176 | 0,689541873 | 0,775734607 |
| b4455 | 153 | 0,35 | 0,510913156 | 0,410376926 | 0,157227339 | 0,691722174 | 0,777992166 |
| b2022 | 1068 | 0,53 | -0,101953099 | 6,574043139 | 0,156378744 | 0,692512643 | 0,778579943 |
| b3368 | 1374 | 0,55 | -0,088100236 | 6,05286083 | 0,156293484 | 0,692592199 | 0,778579943 |
| b0950 | 1254 | 0,52 | -0,076571967 | 5,721849037 | 0,155889655 | 0,692969357 | 0,77880859 |
| b0917 | 183 | 0,48 | 0,102459977 | 5,16360834 | 0,154798976 | 0,693990832 | 0,77976107 |
| b3784 | 1104 | 0,49 | -0,081889273 | 6,851700133 | 0,154462472 | 0,694306823 | 0,779920596 |
| b1143 | 342 | 0,39 | -0,105477587 | 4,327796331 | 0,153220225 | 0,695476797 | 0,78084726 |
| b1375 | 234 | 0,36 | 0,505471012 | 0,632250521 | 0,153216618 | 0,695480201 | 0,78084726 |
| b1852 | 1476 | 0,52 | -0,081111338 | 8,252781443 | 0,152231884 | 0,69641155 | 0,781697111 |
| b2683 | 336 | 0,54 | 0,127354861 | 4,326797108 | 0,151652257 | 0,696961376 | 0,782090874 |
| b4594 | 186 | 0,38 | 0,360280839 | 0,996738367 | 0,151494383 | 0,697111344 | 0,782090874 |
| b4254 | 1005 | 0,52 | 0,192668157 | 3,114252018 | 0,15039548 | 0,69815771 | 0,782885686 |
| b2556 | 1428 | 0,53 | 0,079889957 | 5,767900297 | 0,150383501 | 0,69816914 | 0,782885686 |
| b1324 | 507 | 0,51 | -0,100059663 | 10,03638189 | 0,149876224 | 0,698653671 | 0,783233055 |
| b4521 | 344 | 0,5 | 0,150278177 | 2,819147409 | 0,147564695 | 0,700873571 | 0,785525217 |
| b4551 | 201 | 0,48 | 0,124358983 | 3,865181188 | 0,146705668 | 0,70170363 | 0,786258918 |
| b2731 | 2079 | 0,53 | 0,079957113 | 6,296971767 | 0,145645969 | 0,702731444 | 0,786829483 |
| b3446 | 285 | 0,46 | 0,312547837 | 1,323234068 | 0,145645546 | 0,702731854 | 0,786829483 |
| b0761 | 789 | 0,53 | -0,082337137 | 5,38635965 | 0,145637684 | 0,702739496 | 0,786829483 |
| b3562 | 438 | 0,41 | -0,227166485 | 1,464852782 | 0,144947543 | 0,703411197 | 0,787384862 |
| b3339 | 1185 | 0,53 | 0,099597759 | 11,60382575 | 0,144514295 | 0,703833806 | 0,787498779 |
| b3367 | 807 | 0,55 | -0,197484212 | 2,783262821 | 0,144482989 | 0,703864371 | 0,787498779 |
| b2501 | 2067 | 0,49 | -0,065673332 | 7,937538202 | 0,144186903 | 0,70415364 | 0,787625808 |
| b2979 | 1500 | 0,57 | -0,408831525 | 9,188328915 | 0,143762775 | 0,704568594 | 0,787715303 |
| b3029 | 315 | 0,5 | -0,078142354 | 7,867649148 | 0,143745865 | 0,704585152 | 0,787715303 |
| b3896 | 1056 | 0,46 | 0,181890306 | 2,002647051 | 0,142912221 | 0,705402865 | 0,788432828 |
| b2178 | 1095 | 0,51 | 0,08948518 | 4,303248054 | 0,142547596 | 0,705761378 | 0,788636873 |
| b3818 | 381 | 0,33 | 0,410111989 | 1,040351031 | 0,140921688 | 0,707366444 | 0,7902334 |
| b0081 | 459 | 0,52 | -0,067595269 | 7,331312505 | 0,138141556 | 0,710135592 | 0,793129261 |
| b1706 | 1437 | 0,53 | 0,071242652 | 6,708257537 | 0,13770796 | 0,710570327 | 0,793417091 |
| b3042 | 291 | 0,49 | 0,078651807 | 5,904120332 | 0,136549478 | 0,711735684 | 0,794520382 |
| b1135 | 654 | 0,51 | 0,139451044 | 4,410549283 | 0,136048045 | 0,712241835 | 0,794802916 |
| b3462 | 1059 | 0,53 | 0,073721237 | 7,244953106 | 0,13594751 | 0,712343443 | 0,794802916 |
| b2025 | 777 | 0,52 | 0,140908572 | 5,528625796 | 0,13540383 | 0,712893669 | 0,795032045 |
| b4417 | 79 | 0,46 | 0,445876421 | 0,443993366 | 0,135394061 | 0,712903568 | 0,795032045 |
| b0771 | 2262 | 0,51 | 0,103295549 | 3,707872605 | 0,134494482 | 0,713816777 | 0,795770236 |
| b1577 | 765 | 0,49 | 0,23492285 | 1,661378309 | 0,134392425 | 0,713920598 | 0,795770236 |
| b0061 | 696 | 0,55 | -0,16152952 | 3,101552543 | 0,133996772 | 0,714323516 | 0,796015582 |
| b1766 | 1857 | 0,52 | -0,062579508 | 6,374401764 | 0,133827685 | 0,714495914 | 0,796015582 |
| b0990 | 213 | 0,44 | -0,272281603 | 1,70055907 | 0,133257593 | 0,715078081 | 0,796466192 |
| b4510 | 183 | 0,57 | 0,46460933 | 0,410376926 | 0,132049165 | 0,716316785 | 0,79764766 |
| b0096 | 918 | 0,51 | -0,086160137 | 10,52255019 | 0,130812146 | 0,71759146 | 0,798683781 |
| b0020 | 906 | 0,47 | 0,068309922 | 6,048717944 | 0,130800342 | 0,717603656 | 0,798683781 |
| b4528 | 186 | 0,57 | 0,461650621 | 0,410376926 | 0,130362536 | 0,718056445 | 0,798989321 |
| b4505 | 249 | 0,47 | -0,109938356 | 3,914008471 | 0,130187094 | 0,718238132 | 0,798993127 |
| b3148 | 396 | 0,55 | 0,096636033 | 4,952594777 | 0,128955995 | 0,719516964 | 0,800217129 |
| b1047 | 1158 | 0,44 | -0,108174576 | 3,771882673 | 0,126754614 | 0,721820987 | 0,802580418 |
| b1702 | 2379 | 0,55 | 0,08660572 | 10,79946592 | 0,12645994 | 0,722131107 | 0,802726097 |
| b0400 | 1296 | 0,53 | -0,114531269 | 4,700758428 | 0,126173087 | 0,722433388 | 0,802862991 |
| b1300 | 1488 | 0,57 | 0,136691088 | 7,048297319 | 0,125847967 | 0,722776462 | 0,803045142 |
| b2459 | 804 | 0,59 | 0,102455133 | 3,620452422 | 0,125179072 | 0,723483867 | 0,803631894 |
| b0904 | 858 | 0,49 | -0,091298461 | 8,007002096 | 0,124962357 | 0,723713515 | 0,803687804 |
| b4295 | 987 | 0,5 | -0,077423596 | 7,600002843 | 0,12365029 | 0,725108689 | 0,805037688 |
| b2851 | 492 | 0,33 | 0,452542937 | 0,681867475 | 0,123114993 | 0,725680281 | 0,805322283 |

| | | | | | | | |
|---|---|---|---|---|---|---|---|
| b0852 | 903 | 0,56 | -0,063694795 | 5,821760859 | 0,123073742 | 0,725724387 | 0,805322283 |
| b0754 | 1053 | 0,52 | 0,067597837 | 7,369196647 | 0,122663868 | 0,726163078 | 0,805609633 |
| b0951 | 1641 | 0,5 | 0,069295495 | 6,644985591 | 0,121784311 | 0,727107261 | 0,806279472 |
| b1585 | 711 | 0,44 | 0,079723488 | 5,890379487 | 0,121766289 | 0,727126646 | 0,806279472 |
| b0150 | 2244 | 0,51 | -0,130711335 | 6,235643124 | 0,121560292 | 0,72734835 | 0,806325823 |
| b4168 | 462 | 0,52 | -0,084567587 | 5,765193816 | 0,12109874 | 0,72784586 | 0,806453932 |
| b2074 | 1248 | 0,55 | 0,110147657 | 4,461261725 | 0,121006203 | 0,727945733 | 0,806453932 |
| b4308 | 1241 | 0,44 | -0,142338469 | 2,958766141 | 0,120794049 | 0,72817487 | 0,806453932 |
| b1313 | 1053 | 0,56 | 0,13897251 | 2,890606462 | 0,120785935 | 0,728183638 | 0,806453932 |
| b1341 | 1233 | 0,48 | -0,072000241 | 6,368483076 | 0,12025512 | 0,728757956 | 0,806890602 |
| b1605 | 1383 | 0,54 | 0,106168536 | 4,259180541 | 0,11951859 | 0,729557205 | 0,80752399 |
| b1669 | 813 | 0,53 | -0,104378395 | 3,766317501 | 0,119396138 | 0,729690352 | 0,80752399 |
| b4096 | 681 | 0,6 | -0,14324236 | 3,796431036 | 0,118795104 | 0,730344988 | 0,807905236 |
| b3163 | 885 | 0,48 | -0,092146835 | 9,64767828 | 0,118748925 | 0,730395362 | 0,807905236 |
| b2422 | 1098 | 0,59 | -0,125048579 | 3,911006825 | 0,117894777 | 0,731329086 | 0,808516242 |
| b1897 | 801 | 0,51 | 0,084224628 | 7,908213216 | 0,11782216 | 0,731408642 | 0,808516242 |
| b4611 | 142 | 0,46 | 0,125163387 | 3,836589063 | 0,117748906 | 0,731488925 | 0,808516242 |
| b4301 | 633 | 0,55 | -0,122290962 | 4,163055643 | 0,116767431 | 0,732567265 | 0,809508502 |
| b2925 | 1080 | 0,51 | -0,092971432 | 11,34727256 | 0,115826056 | 0,733606316 | 0,810456867 |
| b4142 | 294 | 0,49 | 0,170199225 | 8,934161799 | 0,115587252 | 0,733870647 | 0,810549098 |
| b0997 | 2547 | 0,55 | 0,098432486 | 3,950533934 | 0,11537305 | 0,734108004 | 0,810611499 |
| b4707 | 252 | 0,58 | 0,434855579 | 0,410376926 | 0,115207179 | 0,734291975 | 0,810614934 |
| b3090 | 552 | 0,5 | 0,167415865 | 2,697097639 | 0,114829834 | 0,734711047 | 0,810877842 |
| b3172 | 1344 | 0,53 | -0,086139912 | 7,872174673 | 0,114493415 | 0,735085316 | 0,811091183 |
| b3083 | 315 | 0,38 | 0,210003638 | 2,115709292 | 0,113519228 | 0,736172574 | 0,811944341 |
| b0280 | 441 | 0,44 | 0,086286184 | 5,198865406 | 0,113455996 | 0,736243325 | 0,811944341 |
| b1128 | 1122 | 0,54 | 0,066053704 | 8,01056799 | 0,113314258 | 0,736401996 | 0,811944341 |
| b1137 | 666 | 0,35 | 0,148420619 | 3,04139619 | 0,113132008 | 0,736606181 | 0,811969726 |
| b3561 | 996 | 0,47 | -0,149385429 | 3,158636889 | 0,110175923 | 0,739943938 | 0,815448422 |
| b1677 | 237 | 0,51 | -0,092677223 | 13,13960455 | 0,10948421 | 0,740732121 | 0,816088484 |
| b1606 | 723 | 0,51 | -0,088505839 | 5,236527508 | 0,10934691 | 0,740888898 | 0,816088484 |
| b2971 | 411 | 0,44 | 0,261149924 | 1,788868827 | 0,108748317 | 0,741573683 | 0,816642076 |
| b1105 | 642 | 0,56 | 0,069125231 | 8,034152405 | 0,108072551 | 0,742349271 | 0,817295365 |
| b2509 | 1371 | 0,53 | 0,083537071 | 5,323948905 | 0,107073128 | 0,743501265 | 0,818362639 |
| b1948 | 738 | 0,53 | 0,269525311 | 1,280519542 | 0,106859865 | 0,743747853 | 0,818433066 |
| b4150 | 1134 | 0,5 | 0,101205486 | 4,468982682 | 0,106345964 | 0,744343183 | 0,818841686 |
| b1737 | 1359 | 0,49 | 0,087133233 | 4,083122066 | 0,106224111 | 0,744484576 | 0,818841686 |
| b1869 | 396 | 0,51 | 0,071342994 | 5,766915246 | 0,105276382 | 0,745587362 | 0,819853424 |
| b2895 | 522 | 0,51 | -0,058967768 | 6,247936746 | 0,104105324 | 0,746957622 | 0,82115871 |
| b0122 | 348 | 0,5 | 0,128558627 | 4,124611483 | 0,103695975 | 0,747438608 | 0,821485984 |
| b4541 | 318 | 0,39 | 0,334452912 | 0,700248254 | 0,103435188 | 0,74774558 | 0,82162189 |
| b3176 | 1338 | 0,54 | -0,060426324 | 8,446761313 | 0,103101869 | 0,748138552 | 0,821852204 |
| b0413 | 450 | 0,51 | -0,075432187 | 6,550793774 | 0,102259899 | 0,749134339 | 0,822744451 |
| b1549 | 411 | 0,32 | 0,278177589 | 1,055985126 | 0,10163367 | 0,749877905 | 0,823359326 |
| b2134 | 933 | 0,51 | 0,070904949 | 5,155376471 | 0,09982266 | 0,752042553 | 0,82535133 |
| b4627 | 222 | 0,55 | 0,211426083 | 1,336857368 | 0,099807781 | 0,752060427 | 0,82535133 |
| b3263 | 180 | 0,41 | 0,182286479 | 3,076551808 | 0,097948925 | 0,754305027 | 0,827612028 |
| b2122 | 2001 | 0,54 | 0,133885202 | 3,418267444 | 0,09778361 | 0,754505777 | 0,827614787 |
| b3040 | 774 | 0,53 | 0,069503524 | 6,654482859 | 0,097642856 | 0,754676848 | 0,827614787 |
| b4287 | 768 | 0,54 | -0,089435614 | 4,010703421 | 0,097487881 | 0,75486536 | 0,827619017 |
| b4459 | 140 | 0,52 | -0,125686564 | 3,636000985 | 0,095928151 | 0,756771838 | 0,829506329 |
| b2719 | 768 | 0,56 | -0,106507985 | 3,026249463 | 0,094773015 | 0,758194752 | 0,830862807 |
| b0504 | 927 | 0,52 | 0,128418226 | 2,758287266 | 0,09425328 | 0,758838066 | 0,831364511 |
| b1325 | 966 | 0,55 | -0,060069856 | 6,267444683 | 0,093941769 | 0,759224577 | 0,831385055 |
| b3096 | 384 | 0,54 | -0,099048276 | 4,471542269 | 0,093830312 | 0,759363039 | 0,831385055 |
| b2100 | 966 | 0,57 | 0,087549029 | 4,034231189 | 0,093789871 | 0,759413301 | 0,831385055 |
| b3269 | 1182 | 0,5 | -0,124393437 | 3,006377319 | 0,093139599 | 0,760223116 | 0,831928049 |
| b2883 | 1320 | 0,49 | 0,075379184 | 4,647881289 | 0,093093599 | 0,76028052 | 0,831928049 |
| b0870 | 1002 | 0,56 | -0,059038969 | 7,432470054 | 0,092331363 | 0,761233973 | 0,832768041 |
| b1455 | 213 | 0,31 | -0,358774312 | 0,572244338 | 0,092013428 | 0,761632936 | 0,833001176 |
| b1703 | 834 | 0,51 | 0,058171683 | 6,797183511 | 0,091259612 | 0,762581887 | 0,833835573 |
| b3039 | 789 | 0,54 | 0,091774724 | 3,859173721 | 0,08977567 | 0,764462538 | 0,835688072 |
| b2130 | 1158 | 0,57 | 0,079060688 | 5,793270697 | 0,089286808 | 0,765085796 | 0,836057774 |
| b3554 | 711 | 0,5 | -0,057974065 | 8,394262047 | 0,089217895 | 0,765173804 | 0,836057774 |
| b3229 | 639 | 0,54 | 0,061567511 | 8,426199633 | 0,088501835 | 0,766090473 | 0,83685535 |
| b1328 | 900 | 0,47 | -0,084241867 | 4,440293989 | 0,088290127 | 0,766362264 | 0,836948262 |
| b0955 | 1761 | 0,52 | -0,061671686 | 7,809943101 | 0,087790951 | 0,767004515 | 0,837445612 |
| b3807 | 321 | 0,54 | 0,100326368 | 4,915300737 | 0,087435237 | 0,767463399 | 0,837634864 |
| b0396 | 1185 | 0,51 | 0,074458426 | 4,547471768 | 0,087366936 | 0,767551626 | 0,837634864 |
| b2850 | 490 | 0,32 | 0,378010474 | 0,613437756 | 0,086518572 | 0,768650625 | 0,838508756 |
| b1351 | 276 | 0,39 | -0,247254957 | 0,957682519 | 0,086405239 | 0,768797883 | 0,838508756 |
| b1277 | 591 | 0,5 | 0,061127739 | 6,936152793 | 0,086316197 | 0,768913652 | 0,838508756 |
| b2379 | 1239 | 0,55 | 0,061812764 | 7,591272679 | 0,085679176 | 0,769743785 | 0,839011407 |
| b1409 | 897 | 0,44 | 0,355043631 | 0,613245279 | 0,085675201 | 0,769748975 | 0,839011407 |
| b0692 | 1320 | 0,51 | 0,082870142 | 4,567247415 | 0,085137658 | 0,7704521 | 0,839439372 |
| b1141 | 246 | 0,44 | 0,164671835 | 2,365321726 | 0,08508875 | 0,770516193 | 0,839439372 |
| b3409 | 2322 | 0,55 | -0,103613179 | 7,44487707 | 0,084796783 | 0,770899225 | 0,83965257 |
| b2167 | 1692 | 0,56 | 0,063206796 | 8,500983018 | 0,084503484 | 0,771284727 | 0,839868354 |
| b4612 | 156 | 0,33 | 0,37248562 | 0,499612806 | 0,084213734 | 0,771666276 | 0,839916581 |
| b1558 | 213 | 0,48 | 0,221653476 | 1,840698351 | 0,084185259 | 0,771703811 | 0,839916581 |
| b1161 | 405 | 0,37 | 0,300635941 | 0,955958833 | 0,083853665 | 0,772141415 | 0,840188838 |
| b3594 | 843 | 0,34 | -0,101693887 | 3,900485052 | 0,083567113 | 0,772520335 | 0,840397122 |
| b3498 | 2043 | 0,55 | 0,066777985 | 8,840076487 | 0,082681853 | 0,773695415 | 0,841471209 |
| b3471 | 666 | 0,51 | -0,107546045 | 2,977111592 | 0,082335302 | 0,774157277 | 0,841665853 |
| b2237 | 651 | 0,49 | 0,066575241 | 5,468544185 | 0,082265855 | 0,774249958 | 0,841665853 |
| b3850 | 546 | 0,52 | 0,057259624 | 5,592182731 | 0,080682253 | 0,776374963 | 0,843599189 |
| b4649 | 192 | 0,53 | 0,261251801 | 1,140179318 | 0,08066008 | 0,776404876 | 0,843599189 |
| b4288 | 957 | 0,6 | -0,09140078 | 3,315613901 | 0,079638094 | 0,777788455 | 0,844698055 |
| b2378 | 921 | 0,51 | 0,162746632 | 2,738802915 | 0,079634642 | 0,777793145 | 0,844698055 |
| b3071 | 624 | 0,54 | 0,086383173 | 5,065473995 | 0,079359008 | 0,778167949 | 0,844900375 |
| b3483 | 384 | 0,33 | -0,274330025 | 1,015579495 | 0,077701549 | 0,780436706 | 0,847158469 |
| b0648 | 708 | 0,48 | 0,161976706 | 1,840967183 | 0,076443633 | 0,782176026 | 0,848840908 |
| b1471 | 1331 | 0,33 | 0,352247929 | 0,7740629 | 0,075651046 | 0,783279859 | 0,84983305 |
| b4104 | 788 | 0,62 | 0,165719705 | 1,585378167 | 0,075510779 | 0,783475854 | 0,849839976 |

| | | | | | | | |
|---|---|---|---|---|---|---|---|
| b1359 | 858 | 0,48 | -0,102611131 | 2,933002647 | 0,075275089 | 0,783805628 | 0,849991973 |
| b2934 | 444 | 0,42 | 0,17748961 | 1,296481073 | 0,075073031 | 0,784088785 | 0,850093357 |
| b0837 | 1116 | 0,52 | -0,065027357 | 5,870144171 | 0,074338305 | 0,785121875 | 0,850936971 |
| b2037 | 1248 | 0,35 | -0,063766024 | 6,564048511 | 0,074249856 | 0,785246611 | 0,850936971 |
| b2439 | 660 | 0,56 | -0,081828697 | 5,558871211 | 0,073672087 | 0,786063388 | 0,851616173 |
| b1774 | 1044 | 0,47 | 0,12637646 | 2,720453217 | 0,073393181 | 0,786458901 | 0,851838761 |
| b1560 | 1050 | 0,52 | 0,220341227 | 1,433373859 | 0,072974173 | 0,78705461 | 0,852278029 |
| b4526 | 171 | 0,37 | -0,306854429 | 0,612157427 | 0,072731078 | 0,787401062 | 0,852447237 |
| b1292 | 891 | 0,57 | 0,065335813 | 5,140062772 | 0,072242054 | 0,788099894 | 0,85299776 |
| b3238 | 264 | 0,48 | -0,115063994 | 4,045383749 | 0,071501359 | 0,789163216 | 0,853942428 |
| b1085 | 321 | 0,45 | -0,059348281 | 6,294781267 | 0,071240242 | 0,789539476 | 0,854095185 |
| b2630 | 1074 | 0,44 | 0,051119386 | 7,089611727 | 0,071139036 | 0,789685508 | 0,854095185 |
| b2758 | 1092 | 0,43 | -0,1002877 | 3,874490524 | 0,07093879 | 0,789974776 | 0,854201916 |
| b2253 | 1140 | 0,51 | -0,097488235 | 5,515139964 | 0,070652027 | 0,790389785 | 0,854371934 |
| b2549 | 3282 | 0,55 | 0,074193471 | 3,880616742 | 0,070566832 | 0,790513255 | 0,854371934 |
| b1293 | 966 | 0,51 | 0,061716844 | 5,710367565 | 0,070403477 | 0,790750222 | 0,85440361 |
| b2417 | 510 | 0,47 | -0,063590479 | 10,76820591 | 0,070283932 | 0,790923824 | 0,85440361 |
| b1201 | 1920 | 0,51 | 0,065954638 | 5,107608129 | 0,06929804 | 0,792361588 | 0,855750515 |
| b1397 | 1206 | 0,56 | -0,140390649 | 6,128516085 | 0,068775301 | 0,793128357 | 0,856272485 |
| b4435 | 204 | 0,43 | 0,234779234 | 1,521812253 | 0,068708214 | 0,793226987 | 0,856272485 |
| b2841 | 1419 | 0,51 | -0,073655192 | 5,036968097 | 0,068288624 | 0,793845035 | 0,856733312 |
| b0712 | 933 | 0,56 | 0,0772021 | 7,962423668 | 0,067943385 | 0,794355087 | 0,857077395 |
| b0151 | 798 | 0,57 | 0,133665373 | 3,833633982 | 0,06751136 | 0,794995308 | 0,85739821 |
| b1782 | 747 | 0,52 | -0,088287368 | 8,331935238 | 0,067484611 | 0,79503502 | 0,85739821 |
| b2433 | 450 | 0,53 | 0,060471467 | 4,56840037 | 0,067228744 | 0,795415304 | 0,85760197 |
| b2585 | 1356 | 0,49 | -0,064396896 | 8,467003392 | 0,067038289 | 0,79569887 | 0,857701379 |
| b4109 | 2229 | 0,51 | 0,117107905 | 4,06586129 | 0,066623385 | 0,796318108 | 0,858162482 |
| b1345 | 1236 | 0,43 | 0,09570569 | 3,87554026 | 0,066402757 | 0,79664823 | 0,858311867 |
| b3933 | 960 | 0,57 | -0,053900568 | 7,217448593 | 0,065761567 | 0,797610965 | 0,858898678 |
| b3008 | 1188 | 0,51 | 0,064483058 | 5,439808317 | 0,065726596 | 0,797663615 | 0,858898678 |
| b3620 | 1047 | 0,54 | 0,048645605 | 7,093736943 | 0,065657439 | 0,797767782 | 0,858898678 |
| b0134 | 795 | 0,55 | -0,05069055 | 7,953792716 | 0,065227057 | 0,79841735 | 0,859391585 |
| b1411 | 1293 | 0,51 | -0,076371536 | 3,806398856 | 0,065026057 | 0,798721499 | 0,859512547 |
| b1983 | 717 | 0,4 | -0,083343095 | 5,955986999 | 0,064525819 | 0,799480626 | 0,860122939 |
| b4282 | 249 | 0,49 | 0,261685388 | 0,971014599 | 0,064068764 | 0,800176965 | 0,860468905 |
| b0244 | 76 | 0,51 | 0,30461365 | 0,478209824 | 0,064062737 | 0,800186166 | 0,860468905 |
| b2415 | 258 | 0,51 | -0,055212247 | 8,697561049 | 0,063428381 | 0,801157072 | 0,861306308 |
| b4170 | 1848 | 0,56 | 0,046664465 | 7,240621356 | 0,063287565 | 0,801373295 | 0,86133216 |
| b0988 | 504 | 0,55 | 0,072872193 | 4,24339363 | 0,063034485 | 0,801762546 | 0,861543929 |
| b3364 | 1182 | 0,52 | -0,090814591 | 3,568856349 | 0,062506043 | 0,802578002 | 0,862133627 |
| b4652 | 199 | 0,42 | -0,174986385 | 1,509241466 | 0,06242975 | 0,802696034 | 0,862133627 |
| b3723 | 837 | 0,43 | -0,152801066 | 1,798251224 | 0,061922735 | 0,803482389 | 0,862771459 |
| b3556 | 213 | 0,48 | 0,097123528 | 4,10759347 | 0,061481414 | 0,804169643 | 0,863302596 |
| b2601 | 1071 | 0,52 | -0,074355844 | 5,952647607 | 0,060858274 | 0,80514451 | 0,864131203 |
| b1808 | 1911 | 0,55 | 0,060076584 | 5,53260292 | 0,06074194 | 0,805327094 | 0,864131203 |
| b1152 | 780 | 0,58 | 0,140559999 | 1,576507453 | 0,060160814 | 0,806241947 | 0,864818514 |
| b3730 | 1371 | 0,53 | -0,051404046 | 9,098504806 | 0,059998059 | 0,806499008 | 0,864818514 |
| b1383 | 327 | 0,45 | 0,071628757 | 3,986051795 | 0,059968019 | 0,806546494 | 0,864818514 |
| b3464 | 1494 | 0,53 | 0,046418682 | 7,853701897 | 0,059673342 | 0,807012983 | 0,865111741 |
| b2594 | 981 | 0,53 | 0,056053254 | 7,063825902 | 0,059508656 | 0,807274222 | 0,865168195 |
| b4268 | 564 | 0,43 | -0,082840613 | 3,207478761 | 0,059300952 | 0,807604245 | 0,865168195 |
| b4035 | 1116 | 0,55 | -0,137067571 | 8,838909717 | 0,059275492 | 0,807644741 | 0,865168195 |
| b1076 | 1209 | 0,52 | 0,103516421 | 2,97305559 | 0,058883568 | 0,808269288 | 0,865630334 |
| b2086 | 900 | 0,5 | 0,058024611 | 6,684688205 | 0,058472755 | 0,808926302 | 0,866127015 |
| b4647 | 185 | 0,38 | -0,274725693 | 0,521047258 | 0,057997226 | 0,809689874 | 0,866737525 |
| b1075 | 696 | 0,55 | 0,132969497 | 1,97343146 | 0,057260883 | 0,810878808 | 0,867802965 |
| b1083 | 954 | 0,51 | -0,065990384 | 4,169328605 | 0,056815767 | 0,811601438 | 0,868368977 |
| b1791 | 1182 | 0,53 | 0,09116882 | 3,436628458 | 0,056396534 | 0,812284787 | 0,8688927 |
| b2262 | 858 | 0,56 | 0,053865739 | 8,216791054 | 0,056236726 | 0,81254598 | 0,868964706 |
| b2428 | 897 | 0,54 | 0,065232185 | 5,238706755 | 0,055970808 | 0,812981471 | 0,869223033 |
| b1691 | 1266 | 0,45 | 0,187758757 | 1,205744971 | 0,055339096 | 0,814020419 | 0,870126286 |
| b0082 | 942 | 0,55 | 0,044667504 | 7,702333111 | 0,054889075 | 0,814764371 | 0,870713856 |
| b1168 | 1524 | 0,45 | 0,110837093 | 3,087445171 | 0,054719625 | 0,81504533 | 0,870806476 |
| b3101 | 393 | 0,49 | -0,083831834 | 3,113916186 | 0,054118527 | 0,816045708 | 0,871667508 |
| b1949 | 270 | 0,5 | 0,239655491 | 0,849926179 | 0,053815031 | 0,816553028 | 0,87200159 |
| b1497 | 1158 | 0,39 | -0,122590929 | 2,514882494 | 0,053695404 | 0,816753409 | 0,872007808 |
| b3917 | 990 | 0,51 | 0,098376915 | 2,824070669 | 0,053536457 | 0,817020017 | 0,872084714 |
| b4245 | 936 | 0,54 | 0,125312099 | 6,976495195 | 0,05199853 | 0,819621471 | 0,874653198 |
| b2141 | 399 | 0,49 | 0,086937823 | 3,607491667 | 0,051381515 | 0,820676539 | 0,875470346 |
| b1290 | 807 | 0,51 | 0,057369552 | 5,507309899 | 0,051322461 | 0,820777866 | 0,875470346 |
| b1557 | 216 | 0,35 | -0,144419621 | 2,031226626 | 0,051108012 | 0,821146346 | 0,87565499 |
| b2682 | 738 | 0,53 | 0,047471001 | 6,109188933 | 0,049827258 | 0,823364131 | 0,877811141 |
| b1162 | 732 | 0,49 | -0,043187128 | 5,935145047 | 0,049428924 | 0,824059984 | 0,878079466 |
| b1102 | 2190 | 0,5 | -0,071592399 | 3,722439245 | 0,049347288 | 0,824202958 | 0,878079466 |
| b0223 | 768 | 0,54 | 0,042400797 | 6,286333909 | 0,049346877 | 0,824203678 | 0,878079466 |
| b3223 | 690 | 0,58 | -0,057526854 | 4,932099356 | 0,049235202 | 0,824399463 | 0,878079466 |
| b2743 | 627 | 0,53 | 0,037940395 | 6,986978037 | 0,048449272 | 0,825783966 | 0,87934515 |
| b4235 | 1353 | 0,54 | -0,043544078 | 8,062952724 | 0,048071643 | 0,826453389 | 0,879848953 |
| b1815 | 1599 | 0,51 | 0,05434448 | 5,143128065 | 0,047951317 | 0,826667269 | 0,879867656 |
| b4062 | 324 | 0,51 | 0,078426662 | 3,962875815 | 0,047703247 | 0,827109107 | 0,880128921 |
| b1879 | 2079 | 0,55 | -0,072383741 | 4,017846983 | 0,047510576 | 0,827453103 | 0,880285973 |
| b3062 | 606 | 0,54 | 0,158644906 | 3,444802975 | 0,047087911 | 0,828210301 | 0,880882432 |
| b1946 | 414 | 0,55 | 0,221493986 | 1,070931442 | 0,046346561 | 0,829547061 | 0,882094882 |
| b1473 | 882 | 0,5 | 0,071200492 | 3,522267094 | 0,046215555 | 0,829784444 | 0,882138017 |
| b1466 | 696 | 0,56 | -0,079230022 | 6,326002041 | 0,045845052 | 0,830457709 | 0,882644404 |
| b2345 | 1059 | 0,36 | -0,097397916 | 2,14038667 | 0,045289793 | 0,831472053 | 0,883182339 |
| b4654 | 163 | 0,42 | 0,257510925 | 0,488557894 | 0,045279913 | 0,831490162 | 0,883182339 |
| b0320 | 1548 | 0,56 | -0,084646568 | 2,87745445 | 0,045244549 | 0,831554992 | 0,883182339 |
| b0011 | 714 | 0,51 | 0,081168162 | 2,852819881 | 0,043388096 | 0,83499622 | 0,886627116 |
| b3454 | 714 | 0,57 | -0,071827089 | 2,774786524 | 0,041600464 | 0,838383259 | 0,889935853 |
| b3056 | 1239 | 0,56 | -0,046756377 | 7,189180042 | 0,041534648 | 0,838509394 | 0,889935853 |
| b2881 | 2871 | 0,53 | 0,048332771 | 4,889803409 | 0,040879091 | 0,839771486 | 0,891064347 |
| b1301 | 1281 | 0,58 | 0,077061319 | 6,751827631 | 0,040349296 | 0,840799178 | 0,891776171 |
| b1490 | 1383 | 0,44 | -0,06974046 | 5,749785032 | 0,040310121 | 0,840875445 | 0,891776171 |

| | | | | | | | |
|---|---|---|---|---|---|---|---|
| b2463 | 2280 | 0,54 | 0,059298867 | 10,20183416 | 0,040226056 | 0,841039241 | 0,891776171 |
| b2264 | 1671 | 0,58 | -0,040078718 | 7,081517572 | 0,040077873 | 0,841328399 | 0,891871779 |
| b0378 | 1095 | 0,53 | -0,044249041 | 5,871594578 | 0,039971924 | 0,841535485 | 0,89188036 |
| b3043 | 552 | 0,44 | 0,151871564 | 1,363575398 | 0,039524775 | 0,842412634 | 0,892598919 |
| b1266 | 882 | 0,54 | 0,0412812 | 6,183220473 | 0,039092269 | 0,84326598 | 0,893291922 |
| b0253 | 864 | 0,57 | 0,052721627 | 4,626115591 | 0,038589253 | 0,844264636 | 0,894136977 |
| b2849 | 426 | 0,33 | 0,203125277 | 0,689733074 | 0,038489892 | 0,8444627 | 0,894136977 |
| b1921 | 552 | 0,47 | -0,076020001 | 3,253106479 | 0,037690349 | 0,846066243 | 0,895151906 |
| b1522 | 948 | 0,5 | 0,065131324 | 3,704132737 | 0,037668477 | 0,846110355 | 0,895151906 |
| b1049 | 2544 | 0,55 | -0,040620258 | 8,21566362 | 0,037657498 | 0,846132504 | 0,895151906 |
| b3634 | 480 | 0,53 | 0,05292018 | 4,816186018 | 0,037562515 | 0,846324254 | 0,895151906 |
| b0845 | 1209 | 0,55 | 0,045628319 | 4,562216357 | 0,037309339 | 0,846836596 | 0,895151906 |
| b4672 | 105 | 0,3 | -0,228391476 | 0,467128363 | 0,037253951 | 0,846948923 | 0,895151906 |
| b2956 | 1008 | 0,45 | -0,087558343 | 2,591352162 | 0,037221245 | 0,847015292 | 0,895151906 |
| b0877 | 993 | 0,5 | -0,049470124 | 6,090546879 | 0,037219409 | 0,847019017 | 0,895151906 |
| b1576 | 192 | 0,43 | 0,231818728 | 0,603413059 | 0,036740323 | 0,84799471 | 0,895755174 |
| b1711 | 981 | 0,55 | 0,050906741 | 5,013572225 | 0,036646396 | 0,848186771 | 0,895755174 |
| b1499 | 762 | 0,4 | 0,192014516 | 1,176420917 | 0,036645104 | 0,848189415 | 0,895755174 |
| b4503 | 273 | 0,47 | 0,108004988 | 1,769420866 | 0,036239073 | 0,849022629 | 0,896423892 |
| b3585 | 975 | 0,48 | 0,047221022 | 4,861594707 | 0,035494135 | 0,850563984 | 0,897839797 |
| b4692 | 234 | 0,54 | 0,141106548 | 1,331754408 | 0,034903909 | 0,851797157 | 0,898929799 |
| b3842 | 489 | 0,5 | 0,054759973 | 4,439973689 | 0,0344193 | 0,85281775 | 0,899794999 |
| b0399 | 690 | 0,54 | -0,044445353 | 4,858423386 | 0,03414403 | 0,853400786 | 0,900198241 |
| b2923 | 636 | 0,51 | 0,070731576 | 3,629215272 | 0,033925807 | 0,853864723 | 0,900475691 |
| b1352 | 222 | 0,42 | -0,175896052 | 0,718533939 | 0,033549232 | 0,854668953 | 0,900608499 |
| b3279 | 555 | 0,49 | 0,032025022 | 6,437890985 | 0,03354053 | 0,854687591 | 0,900608499 |
| b2718 | 411 | 0,53 | 0,066867417 | 3,032847715 | 0,033508133 | 0,854757008 | 0,900608499 |
| b1799 | 924 | 0,52 | -0,051634908 | 6,559214084 | 0,033490682 | 0,854794412 | 0,900608499 |
| b3551 | 2334 | 0,54 | 0,030779758 | 6,616768942 | 0,033060033 | 0,855720706 | 0,901194534 |
| b2836 | 2160 | 0,53 | -0,032584595 | 6,908270643 | 0,033045176 | 0,855752774 | 0,901194534 |
| b4618 | 90 | 0,42 | 0,086476099 | 2,321782415 | 0,03293507 | 0,855990659 | 0,901233294 |
| b2023 | 591 | 0,57 | 0,051452394 | 5,500493526 | 0,032579105 | 0,856762545 | 0,90183413 |
| b1730 | 804 | 0,35 | -0,130251388 | 1,154067173 | 0,032454655 | 0,857033432 | 0,901907453 |
| b3132 | 1281 | 0,55 | -0,059283605 | 3,929708232 | 0,031728396 | 0,858625074 | 0,903370324 |
| b2782 | 336 | 0,46 | -0,046665289 | 4,760473685 | 0,031577893 | 0,858957252 | 0,903507722 |
| b4248 | 396 | 0,47 | 0,072740553 | 3,908337574 | 0,031385228 | 0,859383683 | 0,903744174 |
| b4443 | 206 | 0,43 | 0,048117093 | 11,62984544 | 0,031283617 | 0,859609127 | 0,903769202 |
| b0319 | 864 | 0,47 | 0,115858984 | 1,455638475 | 0,031077344 | 0,860067946 | 0,903810821 |
| b0343 | 1254 | 0,46 | -0,060996664 | 3,31831189 | 0,03101513 | 0,860206638 | 0,903810821 |
| b3888 | 990 | 0,55 | -0,031682264 | 7,605484774 | 0,030994045 | 0,860253673 | 0,903810821 |
| b4167 | 1548 | 0,57 | -0,040027321 | 8,12513768 | 0,030685476 | 0,860943933 | 0,904050608 |
| b2444 | 246 | 0,49 | -0,11040308 | 1,612102068 | 0,030586745 | 0,861165545 | 0,904050608 |
| b3049 | 201 | 0,45 | -0,065022661 | 9,19028205 | 0,030541297 | 0,861267682 | 0,904050608 |
| b4582 | 1840 | 0,46 | 0,049230689 | 4,179174862 | 0,030531935 | 0,861288732 | 0,904050608 |
| b3053 | 2841 | 0,56 | 0,04320809 | 8,39369776 | 0,030430478 | 0,861517058 | 0,904078543 |
| b2124 | 471 | 0,49 | 0,045701158 | 4,964040585 | 0,029725747 | 0,863113982 | 0,905542337 |
| b1372 | 3363 | 0,53 | -0,056724686 | 4,562919421 | 0,029379062 | 0,863906728 | 0,906161936 |
| b4650 | 252 | 0,44 | -0,163553769 | 0,809543651 | 0,028989291 | 0,864803766 | 0,906565526 |
| b4624 | 90 | 0,56 | 0,202790298 | 0,509944558 | 0,028931981 | 0,864936184 | 0,906565526 |
| b3725 | 774 | 0,52 | 0,072742083 | 6,278552761 | 0,028902967 | 0,865003273 | 0,906565526 |
| b1985 | 1488 | 0,49 | -0,046271354 | 5,02410246 | 0,028860917 | 0,86510057 | 0,906565526 |
| b3433 | 1104 | 0,54 | -0,038219902 | 8,989812884 | 0,028642781 | 0,865606467 | 0,906883634 |
| b3376 | 1086 | 0,57 | -0,056157715 | 3,821808935 | 0,028074883 | 0,866932893 | 0,908061048 |
| b2248 | 783 | 0,51 | 0,061009417 | 3,038016918 | 0,027964535 | 0,867192227 | 0,908120459 |
| b4264 | 999 | 0,47 | 0,047230185 | 4,333128916 | 0,027551229 | 0,868168259 | 0,908749527 |
| b3749 | 1506 | 0,51 | 0,050449081 | 7,092508339 | 0,027538493 | 0,868198455 | 0,908749527 |
| b2920 | 1479 | 0,53 | 0,050262366 | 3,300189517 | 0,027318882 | 0,868720257 | 0,909083398 |
| b1916 | 723 | 0,47 | -0,039306098 | 5,202675335 | 0,026633187 | 0,870363505 | 0,91059039 |
| b1771 | 981 | 0,48 | -0,056278436 | 3,346421984 | 0,026083759 | 0,871695933 | 0,911562888 |
| b3785 | 1047 | 0,53 | -0,027765869 | 6,666753792 | 0,02608217 | 0,871699807 | 0,911562888 |
| b1867 | 567 | 0,53 | 0,047213112 | 5,78784846 | 0,025993586 | 0,871915982 | 0,911576262 |
| b4613 | 129 | 0,47 | -0,069843407 | 3,536409403 | 0,025336119 | 0,87353239 | 0,913053212 |
| b3145 | 1092 | 0,48 | 0,052147602 | 4,034995113 | 0,025183766 | 0,873910015 | 0,913234947 |
| b4110 | 879 | 0,57 | 0,066098464 | 2,672922674 | 0,024315133 | 0,876085751 | 0,915295183 |
| b2263 | 759 | 0,56 | 0,0311435 | 5,422971524 | 0,024105038 | 0,876617947 | 0,915414409 |
| b0208 | 915 | 0,5 | 0,043686525 | 5,301409752 | 0,024054954 | 0,876745167 | 0,915414409 |
| b1556 | 216 | 0,46 | 0,186745169 | 0,652617069 | 0,024028429 | 0,876812596 | 0,915414409 |
| b0660 | 1041 | 0,54 | -0,038438286 | 8,709418159 | 0,023858811 | 0,8772447 | 0,915518492 |
| b4031 | 1476 | 0,5 | 0,053203149 | 3,800443093 | 0,023828995 | 0,877320822 | 0,915518492 |
| b2600 | 1122 | 0,52 | -0,042243177 | 6,019175923 | 0,023681279 | 0,877698654 | 0,915699574 |
| b2689 | 429 | 0,49 | 0,03134085 | 5,74001655 | 0,023313853 | 0,878643737 | 0,916472244 |
| b0363 | 1197 | 0,5 | 0,053247393 | 3,105833131 | 0,023193806 | 0,878954168 | 0,916582732 |
| b1346 | 216 | 0,45 | -0,17329428 | 0,642934573 | 0,022950604 | 0,879585595 | 0,916903799 |
| b3491 | 1053 | 0,47 | 0,061265222 | 7,703387681 | 0,022864173 | 0,879810822 | 0,916903799 |
| b2193 | 648 | 0,52 | 0,031090571 | 5,926748089 | 0,022839277 | 0,879875778 | 0,916903799 |
| b1412 | 606 | 0,52 | 0,043577428 | 4,944074638 | 0,022521932 | 0,880706951 | 0,917556614 |
| b1603 | 1533 | 0,52 | -0,034949071 | 9,149805568 | 0,022358098 | 0,881138401 | 0,917792776 |
| b2767 | 261 | 0,55 | 0,064221371 | 2,260822588 | 0,022192682 | 0,88157566 | 0,917872077 |
| b2667 | 300 | 0,53 | 0,088752898 | 3,081508202 | 0,022174389 | 0,881624117 | 0,917872077 |
| b4462 | 2253 | 0,37 | 0,113359727 | 1,427671372 | 0,022031728 | 0,882002725 | 0,918052999 |
| b1690 | 1215 | 0,45 | -0,113558695 | 1,606862189 | 0,021865444 | 0,882445611 | 0,918216414 |
| b3531 | 1107 | 0,55 | 0,050751825 | 3,413794002 | 0,02181906 | 0,882569458 | 0,918216414 |
| b1335 | 516 | 0,53 | 0,050004737 | 4,536067925 | 0,0216437 | 0,883038898 | 0,918491609 |
| b1103 | 360 | 0,51 | -0,030688951 | 7,607333812 | 0,02122422 | 0,884169791 | 0,919454525 |
| b1575 | 189 | 0,36 | -0,143457896 | 0,603672359 | 0,021099245 | 0,884508919 | 0,919471664 |
| b1660 | 1212 | 0,53 | -0,063751282 | 3,74661753 | 0,021067006 | 0,884596567 | 0,919471664 |
| b0891 | 612 | 0,51 | -0,026089019 | 6,95866851 | 0,020587226 | 0,885909136 | 0,920622478 |
| b2093 | 285 | 0,42 | -0,049864681 | 10,55208995 | 0,02048398 | 0,886193629 | 0,920704647 |
| b1909 | 87 | 0,6 | 0,170687207 | 0,572426975 | 0,02033519 | 0,886604906 | 0,920918468 |
| b1267 | 621 | 0,48 | 0,028493146 | 6,720575462 | 0,020011483 | 0,887505018 | 0,921639827 |
| b2110 | 720 | 0,39 | 0,137277042 | 1,108582787 | 0,018867514 | 0,890746983 | 0,924792212 |
| b0881 | 321 | 0,45 | -0,030444108 | 6,087217488 | 0,01859842 | 0,891524027 | 0,925372092 |
| b1659 | 933 | 0,54 | 0,03119749 | 4,674130881 | 0,018530198 | 0,891721933 | 0,925372092 |
| b1107 | 1026 | 0,53 | 0,023929106 | 7,1555178 | 0,018379082 | 0,892161639 | 0,925372092 |

| | | | | | | | |
|---|---|---|---|---|---|---|---|
| b2811 | 444 | 0,55 | 0,031239786 | 5,159635857 | 0,018379067 | 0,892161683 | 0,925372092 |
| b3718 | 723 | 0,5 | -0,048460259 | 3,170273829 | 0,018318704 | 0,892337836 | 0,925372092 |
| b2065 | 582 | 0,6 | 0,02392333 | 7,26357772 | 0,017812197 | 0,893827745 | 0,926470185 |
| b3659 | 1185 | 0,46 | 0,089994351 | 1,475765806 | 0,017787302 | 0,893901527 | 0,926470185 |
| b2430 | 1305 | 0,52 | -0,043561548 | 4,919194395 | 0,017748423 | 0,894016857 | 0,926470185 |
| b3265 | 1158 | 0,52 | -0,091016639 | 2,016109256 | 0,017477945 | 0,894822796 | 0,927091025 |
| b1377 | 1134 | 0,47 | 0,089590812 | 2,250007419 | 0,017255106 | 0,895491564 | 0,927521735 |
| b0049 | 843 | 0,56 | 0,022945844 | 6,478218276 | 0,017181446 | 0,895713594 | 0,927521735 |
| b0317 | 498 | 0,53 | 0,076650352 | 2,070179962 | 0,017133178 | 0,895859347 | 0,927521735 |
| b4024 | 1350 | 0,54 | -0,038274576 | 4,725087542 | 0,01704469 | 0,896127095 | 0,927584675 |
| b0291 | 2526 | 0,55 | -0,03757678 | 4,173091559 | 0,016806931 | 0,896850031 | 0,927640208 |
| b0376 | 1158 | 0,55 | -0,044275567 | 5,504883942 | 0,016774701 | 0,896948429 | 0,927640208 |
| b0114 | 2664 | 0,53 | -0,036446328 | 10,21142689 | 0,01677301 | 0,896953593 | 0,927640208 |
| b2224 | 1185 | 0,53 | -0,047049348 | 2,738549556 | 0,016754999 | 0,897008626 | 0,927640208 |
| b4169 | 1338 | 0,55 | -0,028892784 | 7,14339981 | 0,015806854 | 0,899949365 | 0,930466679 |
| b0583 | 621 | 0,48 | -0,06711342 | 2,066644423 | 0,015730904 | 0,900188758 | 0,930499542 |
| b2261 | 963 | 0,59 | -0,028512825 | 7,659720686 | 0,015602871 | 0,900593653 | 0,930703424 |
| b3805 | 942 | 0,56 | 0,022303436 | 7,833990871 | 0,015505859 | 0,900901568 | 0,930807015 |
| b2002 | 447 | 0,58 | 0,051243513 | 2,703514405 | 0,015055123 | 0,902345203 | 0,932083706 |
| b1427 | 540 | 0,43 | -0,031931264 | 5,432226568 | 0,014971505 | 0,902615419 | 0,932147997 |
| b2835 | 1194 | 0,56 | -0,02519255 | 5,988679536 | 0,01486669 | 0,90295522 | 0,932284104 |
| b0118 | 2598 | 0,56 | 0,040915236 | 12,30576546 | 0,01465004 | 0,903661451 | 0,932798394 |
| b0099 | 390 | 0,49 | 0,044880006 | 2,963496776 | 0,014367543 | 0,904590339 | 0,933542229 |
| b0941 | 1071 | 0,48 | -0,034428519 | 3,647030214 | 0,0142609 | 0,9049434 | 0,933691602 |
| b0601 | 630 | 0,47 | -0,044154346 | 3,541099185 | 0,014100356 | 0,905477448 | 0,9340276 |
| b2163 | 660 | 0,45 | -0,055858549 | 2,387885136 | 0,013961942 | 0,905940359 | 0,934290081 |
| b0514 | 1146 | 0,56 | -0,038018817 | 4,258674735 | 0,013449542 | 0,907674605 | 0,935863257 |
| b0890 | 3990 | 0,54 | -0,025338197 | 9,139575598 | 0,013330179 | 0,90808338 | 0,93606939 |
| b1793 | 255 | 0,53 | 0,030866411 | 3,867575093 | 0,012708425 | 0,910243307 | 0,938047334 |
| b3959 | 777 | 0,55 | -0,037599913 | 5,024415344 | 0,012630525 | 0,910517667 | 0,938047334 |
| b0189 | 255 | 0,48 | -0,021945205 | 6,4079888 | 0,012563375 | 0,910754851 | 0,938047334 |
| b4241 | 948 | 0,54 | -0,028760459 | 4,761071742 | 0,012539494 | 0,910839357 | 0,938047334 |
| b2309 | 783 | 0,49 | 0,026872259 | 7,308118912 | 0,012308726 | 0,9116602 | 0,938677008 |
| b0215 | 732 | 0,5 | 0,018861875 | 6,604485945 | 0,011465025 | 0,914729595 | 0,941621048 |
| b1172 | 345 | 0,52 | 0,03699354 | 3,332214232 | 0,011344579 | 0,91517698 | 0,941865264 |
| b0190 | 546 | 0,52 | 0,02466465 | 5,13363319 | 0,011106952 | 0,916066727 | 0,942533362 |
| b0657 | 1539 | 0,56 | 0,023550548 | 6,249079089 | 0,011040099 | 0,916318777 | 0,942533362 |
| b4353 | 204 | 0,5 | 0,033183064 | 3,423214462 | 0,011003516 | 0,916457026 | 0,942533362 |
| b0790 | 762 | 0,56 | -0,026616893 | 5,802460689 | 0,010892833 | 0,916876733 | 0,942748685 |
| b3843 | 1494 | 0,54 | -0,020050445 | 8,495446009 | 0,01066103 | 0,917762763 | 0,94344328 |
| b3426 | 1506 | 0,55 | 0,029875059 | 6,682176185 | 0,010571275 | 0,918108445 | 0,943582218 |
| b3989 | 540 | 0,42 | 0,039629115 | 3,374510947 | 0,010413385 | 0,918720167 | 0,94399445 |
| b2357 | 495 | 0,54 | 0,12142048 | 0,5530997 | 0,010354207 | 0,91895065 | 0,944014855 |
| b4094 | 558 | 0,6 | -0,056753969 | 2,174770733 | 0,010281021 | 0,91923661 | 0,944092229 |
| b2618 | 291 | 0,51 | 0,033389033 | 4,437195143 | 0,010173001 | 0,91966057 | 0,944311265 |
| b4452 | 105 | 0,5 | 0,048329326 | 2,559116473 | 0,010079347 | 0,920029984 | 0,944474207 |
| b0851 | 723 | 0,53 | 0,020554437 | 6,370935994 | 0,009989088 | 0,920387651 | 0,944625018 |
| b1391 | 498 | 0,56 | -0,073543077 | 4,36332828 | 0,00966658 | 0,921679176 | 0,94573399 |
| b1355 | 135 | 0,47 | -0,11116329 | 0,700377371 | 0,009455155 | 0,922537691 | 0,946398245 |
| b4289 | 999 | 0,61 | 0,045871584 | 3,283839432 | 0,009299623 | 0,923175451 | 0,946798746 |
| b3938 | 318 | 0,52 | 0,035275343 | 6,602672887 | 0,00925714 | 0,923350585 | 0,946798746 |
| b3802 | 1197 | 0,55 | -0,020424131 | 8,248888272 | 0,009176985 | 0,923682133 | 0,946922077 |
| b1431 | 669 | 0,45 | 0,020486268 | 7,663311466 | 0,008959875 | 0,92458758 | 0,947633554 |
| b3929 | 486 | 0,52 | 0,023786481 | 8,720308825 | 0,008747372 | 0,925484594 | 0,948336065 |
| b2929 | 510 | 0,48 | 0,019249475 | 5,222085828 | 0,008682732 | 0,925759625 | 0,94840106 |
| b0014 | 1917 | 0,51 | -0,047338687 | 11,66227831 | 0,008370969 | 0,927100866 | 0,949558063 |
| b3532 | 2340 | 0,55 | -0,024683069 | 4,559974764 | 0,00823994 | 0,927672079 | 0,949926035 |
| b2548 | 984 | 0,46 | 0,040106109 | 3,095903833 | 0,008146524 | 0,928082117 | 0,950128837 |
| b2150 | 999 | 0,51 | 0,060243618 | 9,841401037 | 0,008037542 | 0,928563491 | 0,950351697 |
| b0650 | 1671 | 0,5 | 0,026325199 | 3,92553662 | 0,008001394 | 0,928723881 | 0,950351697 |
| b0227 | 750 | 0,52 | -0,032946166 | 3,646540957 | 0,007889704 | 0,929221778 | 0,950644147 |
| b2928 | 714 | 0,5 | 0,020375708 | 5,203323212 | 0,007736482 | 0,929910633 | 0,951110515 |
| b4386 | 1017 | 0,58 | -0,022392852 | 6,022946232 | 0,007655851 | 0,930275896 | 0,951110515 |
| b0455 | 114 | 0,64 | 0,104517386 | 0,710329757 | 0,007647408 | 0,930314257 | 0,951110515 |
| b3195 | 810 | 0,55 | -0,016812553 | 6,469198386 | 0,007288356 | 0,931965758 | 0,952581648 |
| b1144 | 309 | 0,47 | 0,032657159 | 3,257918303 | 0,007167537 | 0,932530656 | 0,952941724 |
| b2467 | 576 | 0,49 | -0,019646762 | 4,764253925 | 0,007121602 | 0,932746689 | 0,952945216 |
| b4679 | 84 | 0,35 | 0,100322961 | 0,520473757 | 0,006955214 | 0,933535137 | 0,953533383 |
| b4674 | 66 | 0,14 | 0,104509628 | 0,410376926 | 0,006753979 | 0,934501514 | 0,95383983 |
| b4677 | 66 | 0,2 | 0,104509628 | 0,410376926 | 0,006753979 | 0,934501514 | 0,95383983 |
| b4683 | 81 | 0,26 | 0,104509628 | 0,410376926 | 0,006753979 | 0,934501514 | 0,95383983 |
| b2610 | 1362 | 0,54 | -0,018869924 | 8,379753678 | 0,006715814 | 0,93468642 | 0,95383983 |
| b0296 | 264 | 0,46 | 0,059052257 | 1,356611412 | 0,006595867 | 0,935271015 | 0,954219142 |
| b1347 | 210 | 0,35 | 0,082981697 | 0,61282542 | 0,006476176 | 0,935859725 | 0,954389756 |
| b3325 | 1953 | 0,5 | 0,029885121 | 3,533575356 | 0,006475287 | 0,935864118 | 0,954389756 |
| b0709 | 1482 | 0,52 | -0,023837126 | 3,964793215 | 0,006353982 | 0,936466422 | 0,954786739 |
| b4022 | 873 | 0,52 | -0,015297616 | 5,69893569 | 0,006127682 | 0,937605713 | 0,955612195 |
| b0832 | 912 | 0,53 | -0,020475274 | 4,36185407 | 0,006108655 | 0,937702462 | 0,955612195 |
| b3203 | 288 | 0,45 | 0,014252312 | 8,117894668 | 0,005766453 | 0,939469087 | 0,957194918 |
| b1755 | 1536 | 0,57 | -0,031293772 | 4,151040584 | 0,005405215 | 0,941392197 | 0,958936325 |
| b4011 | 384 | 0,45 | -0,029818697 | 2,804358666 | 0,005201076 | 0,942507617 | 0,959854383 |
| b3329 | 510 | 0,56 | 0,085602413 | 1,112063637 | 0,005157228 | 0,942750055 | 0,959883177 |
| b0054 | 2355 | 0,51 | 0,015454855 | 9,71617805 | 0,005102756 | 0,943052685 | 0,95997323 |
| b2866 | 2259 | 0,53 | 0,015937963 | 5,221229911 | 0,004922325 | 0,944066881 | 0,960787412 |
| b4553 | 300 | 0,48 | 0,033788588 | 3,068477374 | 0,00464772 | 0,945646972 | 0,96217701 |
| b3177 | 849 | 0,52 | -0,014149046 | 5,790470506 | 0,004183913 | 0,94842626 | 0,964680963 |
| b1544 | 234 | 0,35 | -0,077206699 | 0,603421754 | 0,004165717 | 0,948538377 | 0,964680963 |
| b3960 | 1374 | 0,56 | -0,015608229 | 6,766786432 | 0,003936107 | 0,949974815 | 0,965922668 |
| b3121 | 1188 | 0,32 | -0,062316504 | 0,901575944 | 0,003706844 | 0,951451706 | 0,967204932 |
| b3937 | 609 | 0,52 | 0,021194453 | 3,905380574 | 0,003461689 | 0,953082636 | 0,96852854 |
| b0711 | 657 | 0,58 | 0,016544858 | 7,583991567 | 0,003423481 | 0,953341985 | 0,96852854 |
| b0103 | 621 | 0,53 | -0,020418579 | 5,503475135 | 0,003414664 | 0,953402034 | 0,96852854 |
| b2239 | 1077 | 0,46 | -0,012715716 | 5,91561528 | 0,003263975 | 0,954440673 | 0,969363947 |
| b4299 | 789 | 0,41 | -0,022827881 | 3,243129811 | 0,003100105 | 0,95559786 | 0,970319349 |

| | | | | | | | |
|---|---|---|---|---|---|---|---|
| b0085 | 1488 | 0,57 | 0,011210409 | 8,332498377 | 0,002996335 | 0,956346565 | 0,970750736 |
| b0193 | 825 | 0,49 | -0,01878421 | 3,086143049 | 0,002981333 | 0,956455879 | 0,970750736 |
| b4621 | 204 | 0,28 | -0,067468792 | 0,432868771 | 0,00280739 | 0,957744008 | 0,971749771 |
| b2256 | 891 | 0,53 | -0,017144361 | 4,901552344 | 0,002790151 | 0,957873826 | 0,971749771 |
| b2075 | 3123 | 0,57 | 0,012314595 | 5,318180373 | 0,002706586 | 0,958508887 | 0,972173983 |
| b3796 | 77 | 0,68 | 0,041460302 | 1,401598026 | 0,002434446 | 0,960648261 | 0,974123418 |
| b4682 | 141 | 0,24 | -0,062034303 | 0,444471786 | 0,002385422 | 0,961046183 | 0,97430649 |
| b3885 | 600 | 0,5 | 0,010197907 | 7,727301831 | 0,002358134 | 0,961269456 | 0,97431246 |
| b2822 | 3369 | 0,54 | 0,008293571 | 7,107837895 | 0,00229836 | 0,961763096 | 0,974592402 |
| b1013 | 639 | 0,51 | 0,01238421 | 4,654466522 | 0,002211402 | 0,962492867 | 0,975111444 |
| b1820 | 459 | 0,45 | -0,018603866 | 3,844200881 | 0,002092176 | 0,963517236 | 0,975785001 |
| b1489 | 2400 | 0,5 | 0,015662968 | 5,521021962 | 0,002083474 | 0,963593132 | 0,975785001 |
| b1728 | 591 | 0,51 | 0,020565445 | 2,913867704 | 0,001991846 | 0,964402152 | 0,976383656 |
| b3224 | 1491 | 0,55 | -0,014987114 | 5,985733173 | 0,001941567 | 0,964854015 | 0,976555455 |
| b0044 | 288 | 0,57 | 0,035579699 | 1,133340627 | 0,001924623 | 0,96500761 | 0,976555455 |
| b3204 | 492 | 0,52 | -0,007461638 | 7,561593225 | 0,001839881 | 0,965786166 | 0,977122708 |
| b0341 | 1155 | 0,59 | 0,016132412 | 2,804686204 | 0,001741262 | 0,96671519 | 0,977841905 |
| b0788 | 957 | 0,52 | 0,01331044 | 5,133270225 | 0,001623767 | 0,967857146 | 0,978776112 |
| b0006 | 777 | 0,5 | -0,008650251 | 5,490482011 | 0,00159201 | 0,968172855 | 0,978874518 |
| b1500 | 198 | 0,37 | 0,047616648 | 0,444315403 | 0,001569427 | 0,968399273 | 0,978882621 |
| b2026 | 612 | 0,55 | 0,010826296 | 5,639983426 | 0,001524581 | 0,968853804 | 0,979121251 |
| b1653 | 4617 | 0,57 | -0,007567719 | 6,6193901 | 0,001245498 | 0,971847206 | 0,981924972 |
| b3844 | 702 | 0,53 | -0,006280858 | 7,944333831 | 0,00118934 | 0,972488955 | 0,982351926 |
| b0568 | 2973 | 0,55 | 0,006988866 | 6,303238971 | 0,00115004 | 0,97294713 | 0,982593294 |
| b1387 | 2046 | 0,54 | 0,020118152 | 3,872566733 | 0,001105452 | 0,973476555 | 0,982906493 |
| b3443 | 1272 | 0,28 | -0,028266332 | 0,859153919 | 0,001067522 | 0,973935389 | 0,982988672 |
| b1772 | 948 | 0,45 | 0,013946689 | 2,666747567 | 0,001046148 | 0,974197558 | 0,982988672 |
| b3154 | 444 | 0,54 | 0,010041644 | 4,203070314 | 0,001025489 | 0,974453509 | 0,982988672 |
| b0566 | 762 | 0,46 | 0,015111764 | 2,493468198 | 0,001015366 | 0,974579868 | 0,982988672 |
| b1072 | 660 | 0,52 | -0,012317019 | 2,756610585 | 0,001009407 | 0,974654542 | 0,982988672 |
| b3633 | 1278 | 0,54 | -0,006475825 | 6,020996813 | 0,000935037 | 0,975605788 | 0,98369977 |
| b4587 | 1912 | 0,52 | -0,008461806 | 4,801791813 | 0,000920312 | 0,975798567 | 0,98369977 |
| b1382 | 186 | 0,43 | -0,012474466 | 2,861581339 | 0,000903687 | 0,976018091 | 0,983699816 |
| b1108 | 543 | 0,47 | -0,005849713 | 8,274307155 | 0,000815625 | 0,977216194 | 0,984685922 |
| b1670 | 786 | 0,49 | 0,009302052 | 3,586127258 | 0,000785207 | 0,977644966 | 0,984740153 |
| b2012 | 228 | 0,5 | -0,011669657 | 2,901056953 | 0,000753733 | 0,978097474 | 0,984740153 |
| b3252 | 1941 | 0,51 | 0,005834414 | 6,84674245 | 0,000750488 | 0,978144664 | 0,984740153 |
| b0109 | 894 | 0,53 | -0,005516808 | 5,696186983 | 0,0007502 | 0,978148853 | 0,984740153 |
| b0675 | 753 | 0,53 | 0,005181039 | 6,417093131 | 0,000717364 | 0,978632292 | 0,985005599 |
| b3966 | 1845 | 0,5 | -0,005788444 | 6,883461719 | 0,000642328 | 0,979780419 | 0,985939793 |
| b0804 | 678 | 0,53 | -0,009222689 | 3,781984456 | 0,000587942 | 0,980655178 | 0,986598543 |
| b1671 | 669 | 0,5 | 0,009104962 | 3,211398326 | 0,000505173 | 0,982068205 | 0,987798406 |
| b2570 | 480 | 0,55 | -0,006299601 | 5,008135405 | 0,000491409 | 0,982314154 | 0,987824106 |
| b2442 | 1209 | 0,54 | -0,006029912 | 4,823784823 | 0,00046967 | 0,982709705 | 0,987829341 |
| b0608 | 1239 | 0,5 | -0,005823912 | 5,61982732 | 0,000455943 | 0,982964208 | 0,987829341 |
| b0901 | 591 | 0,43 | -0,009376826 | 2,615473877 | 0,000455068 | 0,982980558 | 0,987829341 |
| b4345 | 1047 | 0,37 | -0,007734947 | 3,06756815 | 0,000434072 | 0,983377754 | 0,98800697 |
| b3460 | 1104 | 0,54 | -0,006523964 | 4,246030319 | 0,000393439 | 0,984174752 | 0,988586114 |
| b3021 | 396 | 0,42 | 0,004438817 | 5,914213089 | 0,000331375 | 0,98547634 | 0,989671736 |
| b0515 | 786 | 0,46 | 0,005357725 | 4,484123522 | 0,000242337 | 0,987579701 | 0,991561877 |
| b1145 | 675 | 0,44 | 0,00367727 | 5,826609131 | 0,000212573 | 0,988367357 | 0,992130458 |
| b3791 | 1131 | 0,54 | 0,003012711 | 6,435872684 | 0,000199567 | 0,988728795 | 0,992271039 |
| b3175 | 333 | 0,5 | 0,005302951 | 7,506069006 | 0,00018975 | 0,989009505 | 0,992330558 |
| b3856 | 528 | 0,49 | 0,003945961 | 4,163225482 | 0,00016001 | 0,989907425 | 0,992950567 |
| b3151 | 1041 | 0,58 | 0,002842016 | 4,946193993 | 0,00015488 | 0,990070523 | 0,992950567 |
| b3288 | 948 | 0,53 | -0,002689268 | 8,367318464 | 0,000127821 | 0,990979455 | 0,993639803 |
| b3623 | 1074 | 0,31 | 0,002074938 | 5,33367 | 7,40213E-05 | 0,993135433 | 0,995578844 |
| b3324 | 816 | 0,42 | -0,003638005 | 2,287453605 | 4,33166E-05 | 0,994748731 | 0,996816875 |
| b2848 | 483 | 0,32 | 0,006490153 | 1,018642382 | 3,98984E-05 | 0,994960183 | 0,996816875 |
| b4684 | 126 | 0,34 | -0,007178246 | 0,583305618 | 3,61056E-05 | 0,995205704 | 0,996816875 |
| b0555 | 498 | 0,48 | 0,002278355 | 3,265236512 | 3,52918E-05 | 0,995260044 | 0,996816875 |
| b3527 | 1497 | 0,53 | -0,001227479 | 6,024848211 | 1,74837E-05 | 0,996663771 | 0,997999782 |
| b2863 | 1137 | 0,42 | -0,001187052 | 3,786177759 | 1,08822E-05 | 0,997367928 | 0,998481808 |
| b3865 | 633 | 0,52 | -0,000296325 | 6,71944735 | 2,78023E-06 | 0,998669607 | 0,999561674 |
| b4491 | 2648 | 0,43 | 0,000306882 | 3,957593087 | 6,44933E-07 | 0,999359237 | 0,999848557 |
| b4271 | 1266 | 0,46 | 0,000136601 | 6,03173071 | 4,64695E-07 | 0,999456094 | 0,999848557 |
| b2916 | 894 | 0,54 | -8,59757E-05 | 5,949782655 | 1,50569E-07 | 0,999690395 | 0,999848557 |
| b3581 | 663 | 0,59 | 2,90105E-05 | 2,344374608 | 3,6026E-08 | 0,999848557 | 0,999848557 |

*Automatically generated reactions*

The flat-files of the Pathway/Genome Data Base (PGDB) for *Escherichia coli* K-12 MG1655 were obtained from the BioCyc ftp site [71]. To automatically generate reactions, the information about reactions, metabolite identifiers and reaction stoichiometries were extracted from the flat-files. Reactions involved with non-metabolic species or generic compounds were removed as described by Hartman *et al* [67]. An atomic balance analysis was performed to ensure that carbon, nitrogen, phosphate and sulphur were correctly balanced in all the automatically generated reactions.

*Transport reactions*

All reactions involving transfer between the organism and the environment were defined in the transport module in the model, except for the transfer of protons, as described by Hartman *et al.* [67]. Briefly, transporters were defined for each of the nutrients in the media and for metabolic by-products ($CO_2$, lactate, acetate, succinate, ethanol, formate and methylthioribose (which is produced as a by-product of spermidine synthesis and has been shown to be exported by *E. coli* [72])). Similarly, transporters were created for all biomass components (individual amino acids, lipopolysaccharides (LPSs), peptidogycan, membrane, DNA and RNA (see [67] for details). The biomass composition of *E. coli* was based on the results from previous study of *E. coli* [34, 67]. An individual module was constructed for the electron transport chain, as the protons need to be balanced in these reactions, contrary to the rest of the model. As described by Hartmann et al. [67], the electron transport chain used in this model was defined by a system capable of synthesising ATP with the net stoichiometry of: $4NADH + 9H^+ + 2O_2 + 5ADP + 5P \rightarrow 4NAD + 9H_2O + 5ATP$.

*Other reactions*

A condensed reaction corresponding to the fatty acid synthetase producing palmitate, from acetyl-CoA, NADPH and ATP, was constructed as described [67]. Also, four reactions to produce each nucleotide were defined. Lastly, reactions required for the production of biomass, but not included in the BioCyc database, were also defined in this module after confirming the presence of the genes encoding the enzymes.

*Model analysis*

A small number of inconsistent enzyme subsets were present initially. These were dealt with by changing reaction directionality (provided this could be supported by experimental data) or simply removing unnecessary reactions (e.g. reactions involved with non-metabolic species or generic compounds). Stoichiometric consistency, enzyme subsets and energy consistency were analysed according to the methods used by Hartman et al. [67]. Stoichiometric consistency was ensured by identifying the un-conserved metabolites, using the method of Gevorgyan *et al.* [73]. Flux balance analysis was used to analyse the model according to Hartman *et al.*[67].

References.

34. Feist, A.M. *et al.* A genome-scale metabolic reconstruction for *Escherichia coli* K-12 MG1655 that accounts for 1260 ORFs and thermodynamic information. *Mol. Syst. Biol.* **3** (2007).

67. Hartman, H.B. *et al.* Identification of potential drug targets in *Salmonella enterica* sv. Typhimurium using metabolic modelling and experimental validation. *Microbiol.* **160**, 1252-1266 (2014).

71. Caspi, R. *et al.* The MetaCyc database of metabolic pathways and enzymes and the BioCyc collection of pathway/genome databases. *Nucl. Acids Res.* **40**, D742-D753 (2012).

72. Hughes, J.A. In vivo hydrolysis of S-adenosyl-L-methionine in *Escherichia coli* increases export of 5 -methylthioribose. *Can. J. Microbiol.* **52**, 599-602 (2006).

73. Gevorgyan, A., Poolman, M.G. & Fell, D.A. Detection of stoichiometric inconsistencies in biomolecular models. *Bioinform.* **24**, 2245-2251 (2008).
